# QCD and strongly coupled gauge theories: challenges and perspectives

**DOI:** 10.1140/epjc/s10052-014-2981-5

**Published:** 2014-10-21

**Authors:** N. Brambilla, S. Eidelman, P. Foka, S. Gardner, A. S. Kronfeld, M. G. Alford, R. Alkofer, M. Butenschoen, T. D. Cohen, J. Erdmenger, L. Fabbietti, M. Faber, J. L. Goity, B. Ketzer, H. W. Lin, F. J. Llanes-Estrada, H. B. Meyer, P. Pakhlov, E. Pallante, M. I. Polikarpov, H. Sazdjian, A. Schmitt, W. M. Snow, A. Vairo, R. Vogt, A. Vuorinen, H. Wittig, P. Arnold, P. Christakoglou, P. Di Nezza, Z. Fodor, X. Garcia i Tormo, R. Höllwieser, M. A. Janik, A. Kalweit, D. Keane, E. Kiritsis, A. Mischke, R. Mizuk, G. Odyniec, K. Papadodimas, A. Pich, R. Pittau, J.-W. Qiu, G. Ricciardi, C. A. Salgado, K. Schwenzer, N. G. Stefanis, G. M. von Hippel, V. I. Zakharov

**Affiliations:** 1 Physik Department, Technische Universität München, James-Franck-Straße 1, 85748 Garching, Germany; 2 Budker Institute of Nuclear Physics, SB RAS, Novosibirsk , 630090 Russia; 3 Novosibirsk State University, Novosibirsk , 630090 Russia; 4 GSI Helmholtzzentrum für Schwerionenforschung GmbH, Planckstraße 1, 64291 Darmstadt, Germany; 5 Department of Physics and Astronomy, University of Kentucky, Lexington, KY 40506-0055 USA; 6 Theoretical Physics Department, Fermi National Accelerator Laboratory, P.O. Box 500, Batavia, IL 60510-5011 USA; 7 Department of Physics, Washington University, St Louis, MO 63130 USA; 8 University of Graz, 8010 Graz, Austria; 9 Faculty of Physics, University of Vienna, Boltzmanngasse 5, 1090 Wien, Austria; 10 Maryland Center for Fundamental Physics and Department of Physics, University of Maryland, College Park, MD 20742-4111 USA; 11 Max-Planck-Institute for Physics, Föhringer Ring 6, 80805 Munich, Germany; 12 Excellence Cluster “Origin and Structure of the Universe”, Technische Universität München, 85748 Garching, Germany; 13 Atominstitut, Technische Universität Wien, 1040 Vienna, Austria; 14 Hampton University, Hampton, VA 23668 USA; 15 Jefferson Laboratory, Newport News, VA 23606 USA; 16 Department of Physics, University of Washington, Seattle, WA 98195-1560 USA; 17 Department Fisica Teorica I, Universidad Complutense de Madrid, 28040 Madrid, Spain; 18 PRISMA Cluster of Excellence, Institut für Kernphysik and Helmholtz Institut Mainz, Johannes Gutenberg-Universität Mainz, 55099 Mainz, Germany; 19 Institute of Theoretical and Experimental Physics, Moscow, 117218 Russia; 20 Moscow Institute for Physics and Technology, Dolgoprudny, 141700 Russia; 21 Centre for Theoretical Physics, University of Groningen, 9747 AG Groningen, The Netherlands; 22 Institut de Physique Nucléaire CNRS/IN2P3, Université Paris-Sud, 91405 Orsay, France; 23 Institut für Theoretische Physik, Technische Universität Wien, 1040 Vienna, Austria; 24 Center for Exploration of Energy and Matter and Department of Physics, Indiana University, Bloomington, IN 47408 USA; 25 Physics Division, Lawrence Livermore National Laboratory, Livermore, CA 94551 USA; 26 Physics Department, University of California, Davis, CA 95616 USA; 27 Department of Physics and Helsinki Institute of Physics, University of Helsinki, Helsinki, P.O. Box 64, 00014 Finland; 28 Department of Physics, University of Virginia, 382 McCormick Rd., P.O. Box 400714, Charlottesville, VA 22904-4714 USA; 29 NIKHEF, Science Park 105, 1098 XG Amsterdam, The Netherlands; 30 Istituto Nazionale di Fisica Nucleare (INFN), Via E. Fermi 40, 00044 Frascati, Italy; 31 Wuppertal University, 42119 Wuppertal, Germany; 32 Eötvös University, 1117 Budapest, Hungary; 33 Forschungszentrum Jülich, 52425 Jülich, Germany; 34 Albert Einstein Center for Fundamental Physics, Institut für Theoretische Physik, Universität Bern, Sidlerstraße 5, 3012 Bern, Switzerland; 35 Faculty of Physics, Warsaw University of Technology, 00-662 Warsaw, Poland; 36 European Organization for Nuclear Research (CERN), Geneva, Switzerland; 37 Department of Physics, Kent State University, Kent, OH 44242 USA; 38 Crete Center for Theoretical Physics, Department of Physics, University of Crete, 71003 Heraklion, Greece; 39 Laboratoire APC, Université Paris Diderot, Paris Cedex 13, Sorbonne Paris-Cité , 75205 France; 40 Theory Group, Physics Department, CERN, 1211 Geneva 23, Switzerland; 41 Faculty of Science, Utrecht University, Princetonplein 5, 3584 CC Utrecht, The Netherlands; 42 Moscow Physical Engineering Institute, Moscow, 115409 Russia; 43 Lawrence Berkeley National Laboratory, 1 Cyclotron Rd, Berkeley, CA 94720 USA; 44 IFIC, Universitat de València, CSIC, Apt. Correus 22085, 46071 València, Spain; 45 Departamento de Fisica Teorica y del Cosmos and CAFPE, Campus Fuentenueva s. n., Universidad de Granada, 18071 Granada, Spain; 46 Physics Department, Brookhaven National Laboratory, Upton, NY 11973 USA; 47 C. N. Yang Institute for Theoretical Physics and Department of Physics and Astronomy, Stony Brook University, Stony Brook, NY 11794 USA; 48 Dipartimento di Fisica, Università degli Studi di Napoli Federico II, 80126 Napoli, Italy; 49 INFN, Sezione di Napoli, 80126 Napoli, Italy; 50 Departamento de Fisica de Particulas y IGFAE, Universidade de Santiago de Compostela, 15782 Santiago de Compostela, Galicia, Spain; 51 Institut für Theoretische Physik II, Ruhr-Universität Bochum, 44780 Bochum, Germany; 52 School of Biomedicine, Far Eastern Federal University, Sukhanova str 8, Vladivostok, 690950 Russia; 53 Present Address: Helmholtz-Institut für Strahlen- und Kernphysik, Universität Bonn, 53115 Bonn, Germany

## Abstract

We highlight the progress, current status, and open challenges of QCD-driven physics, in theory and in experiment. We discuss how the strong interaction is intimately connected to a broad sweep of physical problems, in settings ranging from astrophysics and cosmology to strongly coupled, complex systems in particle and condensed-matter physics, as well as to searches for physics beyond the Standard Model. We also discuss how success in describing the strong interaction impacts other fields, and, in turn, how such subjects can impact studies of the strong interaction. In the course of the work we offer a perspective on the many research streams which flow into and out of QCD, as well as a vision for future developments.

## Overview 

1This document highlights the status and challenges of strong-interaction physics at the beginning of a new era initiated by the discovery of the Higgs particle at the Large Hadron Collider at CERN. It has been a concerted undertaking by many contributing authors, with a smaller group of conveners and editors to coordinate the effort. Together, we have sought to address a common set of questions: What are the latest achievements and highlights related to the strong interaction? What important open problems remain? What are the most promising avenues for further investigation? What do experiments need from theory? What does theory need from experiments? In addressing these questions, we aim to cast the challenges in quantum chromodynamics (QCD) and other strongly coupled physics in a way that spurs future developments.

A core portion of the scientific work discussed in this document was nurtured in the framework of the conference series on “Quark Confinement and the Hadron Spectrum,” which has served over the years as a discussion forum for people working in the field. The starting point of the current enterprise can be traced to its Xth edition (http://www.confx.de), held in Munich in October, 2012. Nearly 400 participants engaged in lively discussions spurred by its seven topical sessions. These discussions inspired the chapters that follow, and their organization is loosely connected to the topical sessions of the conference: Light Quarks; Heavy Quarks; QCD and New Physics; Deconfinement; Nuclear and Astroparticle Physics; Vacuum Structure and Confinement; and Strongly Coupled Theories. This document is an original, focused work that summarizes the current status of QCD, broadly interpreted, and provides a vision for future developments and further research. The document’s wide-angle, high-resolution picture of the field is current through March 15, 2014.

### Readers’ guide

We expect that this work will attract a broad readership, ranging from practitioners in one or more subfields of QCD, to particle or nuclear physicists working in fields other than QCD and the Standard Model (SM), to students starting research in QCD or elsewhere. We should note that the scope of QCD is so vast that it is impossible to cover absolutely everything. Any omissions stem from the need to create something useful despite the numerous, and sometimes rapid, advances in QCD research. To help the reader navigate the rest of the document, let us begin with a brief guide to the contents of and rationale for each chapter.

Section 2 is aimed at all readers and explains the aims of this undertaking in more detail by focusing on properties and characteristics that render QCD a unique part of the SM. We also highlight the broad array of problems for which the study of QCD is pertinent before turning to a description of the experiments and theoretical tools that appear throughout the remaining chapters. Section 2 concludes with a status report on the determination of the fundamental parameters of QCD, namely, the gauge coupling ${\alpha _{\mathrm{s}}}$ and the quark masses.

The wish to understand the properties of the lightest hadrons with the quark model, concomitant with the observation of partons in deep-inelastic electron scattering, sparked the emergence of QCD. We thus begin in Sect. 3 with this physics, discussing not only the current status of the parton distribution functions, but also delving into many aspects of the structure and dynamics of light-quark hadrons at low energies. Section 3 also reviews the hadron spectrum, including exotic states beyond the quark model, such as glueballs, as well as chiral dynamics, probed through low-energy observables. Certain new-physics searches for which control over light-quark dynamics is essential are also described.

Heavy-quark systems have played a crucial role in the development of the SM, QCD especially. Their large mass, compared to the QCD scale, leads to clean experimental signatures and opens up a new theoretical toolkit. Section 4 surveys these theoretical tools in systems such as quarkonium, i.e., bound states of a heavy quark and a heavy antiquark, and hadrons consisting of a heavy quark bound to light degrees of freedom. Highlights of the chapter include an up-to-date presentation of the exotic states $X$, $Y$, $Z$ that have been discovered in the charmonium and bottomonium regions, the state of the art of lattice-QCD calculations, and an extended discussion of the status of our theoretical understanding of quarkonium production at hadron and electron colliders. The latest results for $B$- and $D$-meson semileptonic decays, which are used to determine some SM parameters and to look for signs of new physics, are also discussed.

Control of QCD for both heavy and light quarks, and for gluons as well, is the key to many searches for physics beyond the SM. Section 5 reviews the possibilities and challenges of the searches realized through precision measurements, both at high energy through collider experiments and at low energy through accelerator, reactor, and table-top experiments. In many searches, a comparably precise theoretical calculation is required to separate SM from non-SM effects, and these are reviewed as well. This chapter has an extremely broad scope, ranging from experiments with multi-TeV $pp$ collisions to those with ultracold neutrons and atoms; ranging from top-quark physics to the determinations of the weak-mixing angle at low energies; ranging from searches for new phenomena in quark-flavor violation to searches for permanent electric dipole moments.

In Sect. 5, QCD is a tool to aid the discovery of exotic phenomena external to QCD. The next three chapters treat a rich array of as-yet unexplored phenomena that emerge from QCD in complex, many-hadron systems. Section 6 begins this theme with a discussion of deconfinement in the context of the quark–gluon plasma and heavy-ion collisions. We first give a description of this novel kind of matter and of our present knowledge of the QCD phase diagram, based on the most recent measurements. We then turn to describing near-equilibrium properties of the quark–gluon plasma and its approach to equilibrium. We explain theorists’ present understanding, focusing on ideas and techniques that are directly connected to QCD. Hard probes such as jet quenching and quarkonium suppression as methods to scrutinize the quark–gluon plasma properties are also discussed. The chapter ends with a parallel between thermal field theory calculations in QCD and cosmology and with a note on the chiral magnetic effect.

Section 7 covers cold, dense hadronic systems, including nuclear and hypernuclear physics and also the ultra-dense hadronic matter found in neutron stars, noting also the new phases that are expected to appear at even higher densities. These topics are informed not only by theory and terrestrial experiments but also by astrophysical observations.

At this point the reader finds Sect. 8, which focuses on the biggest question in QCD: the nature of confinement. No experiment has detected a colored object in isolation, suggesting that colored objects are trapped inside color-singlet hadrons. Section 8 focuses on theoretical aspects of confinement and the related phenomenon of chiral-symmetry breaking, and how they arise in non-Abelian gauge theories.

QCD provides a loose prototype of strongly coupled theories, which are reviewed in Sect. 9. Supersymmetry, string theory, and the AdS/CFT correspondence all play a role in this chapter. These ideas modify the dynamics of gauge theories profoundly. Non-supersymmetric theories are also described here, though they are most interesting when the fermion content is such that the dynamics differ markedly from those of QCD, because they then are candidate models of electroweak symmetry breaking. Conformal symmetry is also presented here, both to help understand the phase diagram of non-Abelian gauge theories and to develop additional models of new physics. New exact results in field theories, sometimes inspired by string theory, are put forward, and their connection to computations of scattering amplitudes in QCD, with many legs or at many loops, is discussed. Section 9 further discusses techniques devised for strongly coupled particle physics and their interplay with condensed-matter physics.

Sections 3–9 all contain a section on future directions discussing the most important open problems and challenges, as well as the most interesting avenues for further research. The Appendix provides a list of acronyms explaining the meaning of abbreviations used throughout the review for laboratories, accelerators, and scientific collaborations. Where available, we provide links to web sites with more information.

## The nature of QCD

2QCD is the sector of the Standard Model (SM) of particle physics that describes the strong interactions of quarks and gluons. From a modern perspective, both the SM and general relativity are thought to be effective field theories, describing the low-energy limit of a more fundamental framework emergent at high energies. To begin, we would like to focus on one specific theoretical aspect, because it shows how QCD plays a special role in the SM.

In quantum field theory, couplings are best understood as depending on an energy scale; roughly speaking, this is the scale at which the quantum field theory—understood to be an effective field theory—is defined. In some cases, such as that of the hypercharge coupling or the Higgs self-coupling in the SM, this energy dependence is such that the coupling increases with increasing energy. This behavior predicts the failure of the theory at the shortest distance scales. QCD, on the other hand, is asymptotically free, which means the following. The QCD Lagrangian in the zero-quark-mass limit is scale invariant, and the interactions of the quarks are determined by the dimensionless parameter ${\alpha _{\mathrm{s}}}$. The theory at the quantum (loop) level generates a fundamental, dimensionful scale $\Lambda _\mathrm{QCD}$ which controls the variation of the coupling constant ${\alpha _{\mathrm{s}}}$ with energy scale. In QCD (unlike QED), the coupling *decreases* with increasing energy—as spectacularly confirmed in the kinematic variation of cross-section measurements from high-precision, deep-inelastic scattering data. The decrease is just fast enough that QCD retains its self-consistency in all extreme energy regimes: high center-of-mass scattering energies, of course, but also high temperatures and large baryon chemical potentials, etc. In this way, QCD is the paradigm of a complete physical theory.

Asymptotic freedom allows accurate calculations at high energy with perturbation theory. The success of the technique does not remove the challenge of understanding the non-perturbative aspects of the theory. The two aspects are deeply intertwined. The Lagrangian of QCD is written in terms of quark and gluon degrees of freedom which become apparent at large energy but remain hidden inside hadrons in the low-energy regime. This confinement property is related to the increase of $\alpha _\mathrm{s}$ at low energy, but it has never been demonstrated analytically. We have clear indications of the confinement of quarks into hadrons from both experiments and lattice QCD. Computations of the heavy quark–antiquark potential, for example, display a linear behavior in the quark–antiquark distance, which cannot be obtained in pure perturbation theory. Indeed the two main characteristics of QCD: confinement and the appearance of nearly massless pseudoscalar mesons, emergent from the spontaneous breaking of chiral symmetry, are non-perturbative phenomena whose precise understanding continues to be a target of research. Even in the simpler case of gluodynamics in the absence of quarks, we do not have a precise understanding of how a gap in the spectrum is formed and the glueball spectrum is generated. Glueball states are predictions of QCD, and their mass spectrum can be obtained with lattice-QCD calculations. They have not, however, been unambiguously observed; their predicted mass and width can be significantly modified by $q\bar{q}$ mixing effects.

The vacuum of QCD is also difficult to characterize. One possibility is to characterize the vacuum in terms of several non-perturbative objects. Such a parameterization has been introduced first in the sum rules approach, yielding a separation of short- and long-distance physics based on techniques derived from the existence of asymptotic freedom in QCD. These ideas have proven to be of profound importance, though the specifics have been supplanted, broadly speaking, by effective field theories in QCD, which, as discussed further in Sect. 2.3, systematically separate the high- and low-energy contributions.

Once a low-energy (non-perturbative), gauge-invariant quantity has been defined, one could use it to investigate the low-energy degrees of freedom which could characterize it and their relation to the confinement mechanism. Even in the absence of quarks, there is a fascinating and complex landscape of different possible topological objects: monopoles, vortices, calorons, or dyons, which are investigated using different methods; either lattice-QCD calculations or QCD vacuum models can be used to this end. Some of the recent research in this sector is addressed in Sect. 8.

### Broader themes in QCD

Many of the most influential ideas in field theory have emerged while trying to understand QCD. The renormalization-group methods of Kenneth Wilson, where short-distance degrees of freedom are systematically removed, or “integrated out,” began with attempts to understand the scale invariance of the strong interaction. These ideas flourished in critical phenomena and statistical mechanics, before returning to particle physics after the asymptotic freedom of gauge theories was discovered. It is this view of renormalization that provides QCD the high-energy self-consistency we have discussed, and has also led to one of the two key facets of modern effective field theory. The other key lies in the work of Steven Weinberg, who argued on the grounds of unitarity and analyticity that the correct effective Lagrangian would consist of all the operators with the desired fields and symmetries. This idea is crucial to the analysis of QCD, because it allows the introduction of an effective theory whose fields differ from the original ones. For example, the chiral Lagrangian contains pions and, depending on the context, other hadron fields, but not quarks and gluons. Certainly, QCD has been at the heart of the development of most of our tools and ideas in the construction of the Standard Model.

QCD also has a distinguished pedigree as a description of experimental observations. It is a merger of two insightful ideas, the quark model and the parton model, which were introduced to explain, respectively, the discovery of the hadron “zoo” in the 1960s and then the deep-inelastic scattering events seen in the early 1970s. The acceptance of QCD was forced on us by several discoveries, such as the $J/\psi$ and other charmonium states in 1974, the analogous $\Upsilon$ and bottomonium states in 1977, and the first observation of three-jet events, evoking the gluon, in 1979.

Some themes in QCD recur often enough that they appear in many of the chapters to follow, so we list them here:

QCD gives rise to the visible mass of the Universe, including everyday objects—the confinement scale, $\Lambda _\mathrm{QCD}$, sets the mass of the proton and the neutron. Similar dynamics could, conceivably, play a role in generating the mass of other forms of matter. *Thus, the confinement mechanism pertains to the origin of mass.*

QCD controls many parameters of the SM—QCD is needed to determine ${\alpha _{\mathrm{s}}}$, the six masses of the quarks, and the strong CP-violating parameter, as well as the Cabibbo–Kobayashi–Maskawa (CKM) mixing matrix. These tally to 12 parameters, out of the 19 of the SM (or 26–28 with neutrino masses and mixing). The quark masses and CKM parameters stem from, and the strong-CP parameter is connected to, the poorly understood Yukawa couplings of quarks to the Higgs boson; furthermore, ${\alpha _{\mathrm{s}}}$ may unify with the other gauge couplings. *Thus, quark couplings play a direct role in the search for a more fundamental theory.*

QCD describes the SM background to non-SM physics—in the high-energy regime, where the coupling constant is small and perturbation theory is applicable, QCD predicts the calculable background to new phenomena precisely. For example, QCD calculations of the background were instrumental to the Higgs discovery, and, indeed, QCD is ubiquitous at hadron colliders where direct contributions of new physics are most actively sought. *Thus, QCD plays a fundamental role in our investigations at the high-energy frontier.*

In the low-energy regime, QCD is often the limiting factor in the indirect search for non-SM physics—this is true in all searches for new physics in hadronic systems, be it in the study of CP violation in $B$ decays, or in permanent electric dipole moment searches in hadrons and nuclei. In addition, QCD calculations of hadronic effects are also needed to understand the anomalous magnetic moment of the muon, as well as aspects of neutrino physics. * Thus, QCD also plays a fundamental role in searches for new physics at the intensity frontier.*

Nuclear matter has a fascinating phase diagram—at non-zero temperature and non-zero chemical potential, QCD exhibits a rich phase diagram, which we continue to explore. The QCD equation of state, the possibility of phase transitions and/or crossovers, and the experimental search for the existence of a critical point are all current topics of research. In lattice QCD one can also alter the number of fermions and the number of colors in order to study different scenarios. In addition to the hadronic phase, different states of QCD matter are predicted, such as the quark–gluon plasma, quarkyonic matter, and a color superconductor state with diquark matter. Experiments studying heavy-ion collisions have shown the quark–gluon plasma to be a surprising substance. For example, it seems to be a strongly coupled, nearly perfect liquid with a minimal ratio of shear viscosity to entropy density. *Thus, QCD matter in extreme conditions exhibits rich and sometimes unexpected behavior.*

QCD impacts cosmology—probing the region of the QCD phase diagrams at large temperature allows us to probe conditions which have not existed since the beginning of the universe. The new state of matter formed in heavy-ion collisions existed microseconds after the Big Bang, before hadrons emerged as the universe cooled. *Thus, characterizing the quark–gluon plasma provides information about the early universe.*

QCD is needed for astrophysics—the region of the QCD phase diagram at large chemical potential provides information on the system under conditions of high pressure and large density, as is the case for astrophysical objects such as compact stars. These stars could be neutron stars, quark stars, or hybrids somewhere in between these pure limits. Moreover, one can use astrometric observational data on such objects to help characterize the QCD equation of state. *Thus, terrestrial accelerator experiments and astrophysical observations are deeply connected.*

QCD is a prototype of strongly coupled theories—strongly coupled gauge theories have been proposed as alternatives to the SM Higgs mechanism. Strongly coupled mechanisms may also underlie new sectors of particle physics that could explain the origin of dark matter. Furthermore, the relation between gauge theories and string theories could shed light on the unification of forces. *Thus, QCD provides a launching pad for new models of particle physics.*

QCD inspires new computational techniques for strongly interacting systems—as the prototype of an extremely rich, strongly coupled system, the study of QCD requires a variety of analytical tools and computational techniques, without which progress would halt. These developments fertilize new work in allied fields; for example, QCD methods have helped elucidate the universal properties of ultracold atoms. Conversely, developments in other fields may shed light on QCD itself. For example, the possibility of designing arrays of cold atoms in optical lattices with the gauge symmetry and fermion content of QCD is under development. If successful, this work could yield a kind of quantum computer with QCD as its specific application. *Thus, the challenge of QCD cross-fertilizes other fields of science.*

### Experiments addressing QCD

In this section, we offer a brief overview of the experimental tools of QCD. We discuss $e^{+} e^{-}$ colliders, fixed-target machines, hadron colliders, and relativistic heavy-ion colliders from a QCD perspective.

From the 1960s to 1990s, $e^+e^{-}$ colliders evolved from low center-of-mass energies $\sqrt{s}\sim 1$ GeV with modest luminosity to the Large Electron Positron (LEP) collider with $\sqrt{s}$ up to $209$ GeV and a vastly greater luminosity. Along the way, the $e^+e^{-}$ colliders PETRA (at DESY) and PEP (at SLAC) saw the first three-jet events. A further breakthrough happened at the end of 1990s with the advent of the two $B$-factories at KEK and SLAC and the operation of lower-energy, high-intensity colliders in Beijing, Cornell, Frascati, and Novosibirsk. Experiments at these machines are particularly good for studies of quarkonium physics and decays of open charm and bottom mesons, in a way that spurred theoretical developments. The copious production of $\tau$ leptons at $e^+e^{-}$ colliders led to a way to measure ${\alpha _{\mathrm{s}}}$ via their hadronic decays. Measurements of the hadronic cross section at various energy ranges play a useful role in understanding the interplay of QCD and QED.

Experiments with electron, muon, neutrino, photon, or hadron beams impinging on a fixed target have been a cornerstone of QCD. Early studies of deep inelastic scattering at SLAC led to the parton model. This technique and the complementary production of charged lepton pairs (the so-called Drell–Yan production) have remained an important tool for understanding proton structure. Later, the Hadron–Elektron Ring Anlage (HERA) continued this theme with $e^{-}p$ and $e^+p$ colliding beams. In addition to nucleon structure, fixed-target experiments have made significant contributions to strangeness and charm physics, as well as to the spectroscopy of light mesons, and HERA searched for non-SM particles such as leptoquarks. This line of research continues to this day at Jefferson Lab, J-PARC, Mainz, Fermilab, and CERN; future, post-HERA $ep$ colliders are under discussion.

The history of hadron colliders started in 1971 with $pp$ collisions at CERN’s Intersecting Storage Rings (ISR), at a center-of-mass energy of 30 GeV. The ISR ran for more than 10 years with $pp$ and $p\bar{p}$ collisions, as well as with ion beams: $pd$, $dd$, $p\alpha$, and $\alpha \alpha$. During this time, its luminosity increased by three orders of magnitude. This machine paved the way for the successful operation of proton–antiproton colliders: the S$p\bar{p}$S at CERN with $\sqrt{s}=630$ GeV in the 1980s, and the $p\bar{p}$ Tevatron at Fermilab with $\sqrt{s}=1.96$ TeV, which ran until 2011. Currently, the Large Hadron Collider (LHC) collides $pp$ beams at the highest energies in history, with a design energy of 14 TeV and luminosity four orders of magnitude higher than the ISR. Physics at these machines started from studies of jets at the ISR and moved to diverse investigations including proton structure, precise measurements of the $W$ mass, searches for heavy fundamental particles leading to discoveries of the top quark and Higgs, production of quarkonia, and flavor physics.

At the same time, pioneering experiments with light ions (atomic number, $A$, around 14) at relativistic energies started in the 1970s at LBNL in the United States and at JINR in Russia. The program continued in the 1980s with fixed-target programs at the CERN SPS and BNL AGS. These first experiments employed light-ion beams ($A \sim 30$) on heavy targets ($A \sim 200$). In the 1990s, the search for the quark–gluon plasma continued with truly heavy-ion beams ($A \sim 200$). In this era, the maximum center of mass energy per nucleon was $\sqrt{s_{NN}} \sim 20$ GeV. With the new millennium the heavy-ion field entered the collider era, first with the Relativistic Heavy-Ion Collider (RHIC) at BNL at $\sqrt{s_{NN}}=200$ GeV and, in 2010, the LHC at CERN, reaching the highest currently available energy, $\sqrt{s_{NN}}=2.76$ TeV.

The goal of heavy-ion physics is to map out the nuclear-matter phase diagram, analogous to studies of phase transitions in other fields. Proton-proton collisions occur at zero temperature and baryon density, while heavy-ion collisions can quantify the state of matter of bulk macroscopic systems. The early fixed-target experiments probed moderate values of temperature and baryon density. The current collider experiments reach the zero baryon density, high-temperature regime, where the quark–gluon plasma can be studied under conditions that arose in the early universe.

While the phase structure observed in collider experiments suggests a smooth crossover from hadronic matter to the quark–gluon plasma, theoretical arguments, augmented by lattice QCD computations, suggest a first-order phase transition at non-zero baryon density. The critical point where the line of first-order transitions ends and the crossover regime begins is of great interest. To reach the needed temperature and baryon density, two new facilities—FAIR at GSI and NICA at JINR—are being built.

Work at all these facilities, from $e^+e^{-}$ machines to heavy-ion colliders, require the development of novel trigger systems and detector technologies. The sophisticated detectors used in these experiments, coupled to farms of computers for on-line data analysis, permit the study of unprecedentedly enormous data samples, thus enabling greater sensitivity in searches for rare processes.

### Theoretical tools for QCD

The theory toolkit to study QCD matter is quite diverse, as befits the rich set of phenomena it describes. It includes QCD perturbation theory in the vacuum, semiclassical gauge theory, and techniques derived from string theory. Here we provide a brief outline of some of the wider ranging techniques.

*a. Effective Field Theories (EFTs):* Effective field theories are important tools in modern quantum field theory. They grew out of the operator-product expansion (OPE) and the formalism of phenomenological Lagrangians and, thus, provide a standard way to analyze physical systems with many different energy scales. Such systems are very common from the high-energy domain of particle physics beyond the Standard Model to the low-energy domain of nuclear physics.

Crucial to the construction of an EFT is the notion of *factorization*, whereby the effects in a physical system are separated into a high-energy factor and a low-energy factor, with each factor susceptible to calculation by different techniques. The high-energy factor is typically calculated by making use of powerful analytic techniques, such as weak-coupling perturbation theory and the renormalization group, while the low-energy part may be amenable to lattice gauge theory or phenomenological methods. A key concept in factorization is the principle of *universality*, on the basis of which a low-energy factor can be determined from one theoretical or phenomenological calculation and can then be applied in a model-independent way to a number of different processes. Factorization appeared first in applications of the OPE to QCD, where a classification of operators revealed a leading (set of) operator(s), whose short-distance coefficients could be calculated in a power series in ${\alpha _{\mathrm{s}}}$.

Apart from their theoretical appeal, EFTs are an extremely practical tool. In many cases they allow one to obtain formally consistent and numerically reliable predictions for physical processes that are of direct relevance for experiments. The essential role of factorization was realized early on in the analysis of deep inelastic scattering data in QCD and is codified in the determination of parton distribution functions from experiment, allowing SM predictions in new energy regimes.

Several properties of EFTs are important: they have a power counting in a small parameter which permits rudimentary error assessment for each prediction; they can be more predictive if they have more symmetry; they admit an appropriate definition of physical quantities and supply a systematic calculational framework; finally, they permit the resummation of large logarithms in the ratio of physical scales. For example, an object of great interest, investigated since the inception of QCD, is the heavy quark–antiquark static energy, which can be properly defined only in an EFT and subsequently calculated with lattice gauge theory.

The oldest example is chiral EFT for *light-quark systems*, with roots stemming from the development of current algebra in the 1960s. Chiral EFT has supplied us with an increasingly accurate description of mesons and baryons, and it is an essential ingredient in flavor-physics studies. The EFT description of pion–pion scattering, together with the data on pionium formation, has given us a precise way to confirm the standard mechanism of spontaneous breaking of chiral symmetry in QCD. Chiral effective theory has also allowed lattice QCD to make contact with the physical region of light-quark masses from simulations with computationally less demanding quark masses. For more details, see Sects. 3 and 5.

In the case of the heavy quark–antiquark bound states known as *quarkonium*, the EFT known as Non-relativistic QCD (NRQCD) separates physics at the scale of the heavy-quark mass from those related to the dynamics of quarkonium binding. This separation has solved the problem of uncontrolled infrared divergences in theoretical calculations and has opened the door to a systematic improvement of theoretical predictions. It has given us the tools to understand the data on the quarkonium production cross section at high-energy colliders, such as the Tevatron, the $B$ factories, and the LHC. It has also made it clear that a complete understanding of quarkonium production and decay involves processes in which the quark–antiquark pairs are in a color-octet state, as well as processes in which the pairs are in a color-singlet state. New, lower-energy EFTs, such as potential NRQCD (pNRQCD) have given greater control over some technical aspects of theoretical calculations and have provided a detailed description of the spectrum, decays, and transitions of heavy quarkonia. These EFTs allow the precise extraction of the Standard Model parameters, which are relevant for new-physics searches, from the data of current and future experiments. See Sects. 3 and 4 for applications of NRQCD and pNRQCD.

In the case of strong-interaction processes that involve large momentum transfers and energetic, nearly massless particles, Soft Collinear Effective Field Theory (SCET) has been developed. It has clarified issues of factorization for high-energy processes and has proved to be a powerful tool for resumming large logarithms. SCET has produced applications over a wide range of topics, including heavy-meson decays, deep-inelastic scattering, exclusive reactions, quarkonium-production processes, jet event shapes, and jet quenching. Recent developments regarding these applications can be found in Sects. 3, 4, and 5.

In  *finite-temperature* and  *finite-density* physics, EFTs such as Hard Thermal Loop (HTL), Electric QCD, Magnetic QCD, $\mathrm{NRQCD }_\mathrm{HTL }$, or p$\mathrm{NRQCD }_\mathrm{HTL }$ have allowed progress on problems that are not accessible to standard lattice QCD, such as the evolution of heavy quarkonia in a hot medium, thermodynamical properties of QCD at the very high temperatures, the thermalization rate of non-equilibrium configurations generated in heavy-ion collision experiments, and the regime of asymptotic density. These developments are discussed in Sect. 6.

In *nuclear physics*, chiral perturbation theory has been generalized to provide a QCD foundation to nuclear structure and reactions. EFTs have allowed, among other things, a model-independent description of hadronic and nuclear interactions in terms of parameters that will eventually be determined in lattice calculations, new solutions of few-nucleon systems that show universality and striking similarities to atomic systems near Feshbach resonances, derivation of consistent currents for nuclear reactions, and new approaches to understanding heavier nuclei (such as halo systems) and nuclear matter. Some recent developments are discussed in Sect. 7.

*b. Lattice gauge theory:* In the past decade, numerical lattice QCD has made enormous strides. Computing power, combined with new algorithms, has allowed a systematic simulation of sea quarks for the first time. The most recently generated ensembles of lattice gauge fields now have 2+1+1 flavors of sea quark, corresponding to the up and down, strange, and charm quarks. Most of this work uses chiral EFT to guide an extrapolation of the lightest two quark masses to the physical values. In some ensembles, however, the (averaged) up and down mass is now as light as in nature, obviating this step. Many quantities now have sub-percent uncertainties, so that the next step will require electromagnetism and isospin breaking (in the sea).

Some of the highlights include baryon masses with errors of 2–4 %; pion, kaon, and $D$-meson matrix elements with total uncertainty of 1–2 %; $B$-meson matrix elements to within 5–8 %. The light quark masses are now known directly from QCD (with the chiral extrapolation), with few per cent errors. Several of the best determinations of ${\alpha _{\mathrm{s}}}$ combine perturbation theory (lattice or continuum) with non-perturbatively computed quantities; these are so precise because the key input from experiment is just the scale, upon which ${\alpha _{\mathrm{s}}}$ depends logarithmically. A similar set of analyses yield the charm- and bottom-quark masses with accuracy comparable to perturbative QCD plus experiment. Lattice QCD has also yielded a wealth of thermodynamic properties, not least showing that the deconfinement transition (at small chemical potential) is a crossover, and the crossover temperature has now been found reproducibly.

Vigorous research, both theoretical and computational, is extending the reach of this tool into more demanding areas. The computer calculations take place in a finite spatial box (because computers’ memories are finite), and two-body states require special care. In the elastic case of $K\rightarrow \pi \pi$ transitions, the required extra computing is now manageable, and long-sought calculations of direct CP violation among neutral kaons, and related decay rates, now appear on the horizon. This success has spurred theoretical work on inelastic, multi-body kinematics, which will be required before long-distance contributions to, say, $D$-meson mixing can be computed. Nonleptonic $B$ and $D$ decays will also need these advances, and possibly more. In the realm of QCD thermodynamics, the phase diagram at non-zero chemical potential suffers from a fermion sign problem, exactly as in many condensed-matter problems. This problem is difficult, and several new ideas for workarounds and algorithms are being investigated.

*c. Other non-perturbative approaches:* The theoretical evaluation of a non-perturbative contribution arising in QCD requires non-perturbative techniques. In addition to lattice QCD, many models and techniques have been developed to this end. Among the most used techniques are: the limit of the large number of colors, generalizations of the original Shifman–Vainshtein–Zakharov sum rules, QCD vacuum models and effective string models, the AdS/CFT conjecture, and Schwinger–Dyson equations. Every chapter reports many results obtained with these alternative techniques.

### Fundamental parameters of QCD

Precise determinations of the quark masses and of ${\alpha _{\mathrm{s}}}$ are crucial for many of the problems discussed in the chapters to come. As fundamental parameters of a physical theory, they require both experimental and theoretical input. Because experiments detect hadrons, inside which quarks and gluons are confined, the parameters cannot be directly measured. Instead, they must be determined from a set of relations of the form

2.1 $$\begin{aligned} \,[M_\mathrm{HAD}(\Lambda _\mathrm{QCD}, m_q)]^\mathrm{TH}=[M_\mathrm{HAD}]^\mathrm{EXP}. \end{aligned}$$

One such relation is needed to determine $\Lambda _\mathrm{QCD}$, the parameter which fixes the value of ${\alpha _{\mathrm{s}}}(Q^2)$, the running coupling constant, at a squared energy scale $Q^2$; another six are needed for the (known) quarks—and yet another for the CP-violating angle $\bar{\theta }$. The quark masses and ${\alpha _{\mathrm{s}}}$ depend on the renormalization scheme and scale, so that care is needed to ensure that a consistent set of definitions is used. Some technical aspects of these definitions (such as the one known as the renormalon ambiguity) are continuing objects of theoretical research and can set practical limitations on our ability to determine the fundamental parameters of the theory. In what follows, we have the running coupling and running masses in mind.

Measurements of ${\alpha _{\mathrm{s}}}$ at different energy scales provide a direct quantitative verification of asymptotic freedom in QCD. From the high-energy measurement of the hadronic width of the $Z$ boson, one obtains ${\alpha _{\mathrm{s}}}(M_Z)=0.1197\pm 0.0028$ [1]. From the lower-energy measurement of the hadronic branching fraction of the $\tau$ lepton, one obtains, after running to the $Z$ mass, ${\alpha _{\mathrm{s}}}(M_Z^2)=0.1197\pm 0.0016$ [1]. At intermediate energies, several analyses of quarkonium yield values of ${\alpha _{\mathrm{s}}}$ in agreement with these two; see Sect. 4.4. The scale of the $\tau$ mass is low enough that the error assigned to the latter value remains under discussion; see Sect. 3.5.3 for details. Whatever one makes of these issues, the agreement between these two determinations provides an undeniable experimental verification of the asymptotic freedom property of QCD.

One can combine ${\alpha _{\mathrm{s}}}$ extractions from different systems to try to obtain a precise and reliable “world-average” value. At present most (but not all) individual ${\alpha _{\mathrm{s}}}$ measurements are dominated by systematic uncertainties of theoretical origin, and, therefore, any such averaging is somewhat subjective. Several other physical systems, beyond those mentioned above, are suitable to determine ${\alpha _{\mathrm{s}}}$. Those involving heavy quarks are discussed in Sect. 4.4. Lattice QCD provides several different ${\alpha _{\mathrm{s}}}$ determinations. Recent ones include [2–5], in addition to those mentioned in Sect. 4.4. Some of these determinations quote small errors, because the non-perturbative part is handled cleanly. They therefore may have quite an impact in world-averages, depending on how those are done. For example, lattice determinations dominate the error of the current PDG world average [1]. Fits of parton-distribution functions (PDFs) to collider data also provide a good way to determine ${\alpha _{\mathrm{s}}}$. Current analyses involve sets of PDFs determined in next-to-next-to-leading order (NNLO) [6–9]. Effects from unknown higher-order perturbative corrections in those fits are difficult to assess, however, and have not been addressed in detail so far. They are typically estimated to be slightly larger than the assigned uncertainties of the NNLO extractions. Jet rates and event-shape observables in $e^+e^{-}$ collisions can also provide good sensitivity to ${\alpha _{\mathrm{s}}}$. Current state-of-the-art analyses involve NNLO fixed-order predictions [10–17], combined with the resummation of logarithmically enhanced terms. Resummation for the event-shape cross sections has been performed both in the traditional diagrammatic approach [18] and within the SCET framework [19–21]. One complication with those extractions is the precise treatment of hadronization effects. It is by now clear [22] that analyses that use Monte Carlo generators to estimate them [19, 20, 22–24] tend to obtain larger values of ${\alpha _{\mathrm{s}}}$ than those that incorporate power corrections analytically [25–29]. Moreover, it may not be appropriate to use Monte Carlo hadronization with higher-order resummed predictions [25–27]. We also mention that analyses employing jet rates may be less sensitive to hadronization corrections [30–33]. The SCET-based results of Refs. [26, 28] quote remarkably small errors; one might wonder if the systematics of the procedure have been properly assessed, since the extractions are based only on thrust. In that sense, we mention analogous analyses that employ heavy-jet mass, the $C$-parameter, and broadening are within reach and may appear in the near future. Note that if one were to exclude the event-shape ${\alpha _{\mathrm{s}}}$ extractions that employ Monte Carlo hadronization, the impact on the PDG average could be quite significant. Related analyses employing deep-inelastic scattering data can also be performed [34].

Light-quark masses are small enough that they do not have a significant impact on typical hadronic quantities. Nevertheless, the observed masses of the light, pseudoscalar mesons, which would vanish in the zero-quark-mass limit, are sensitive to them. Moreover, various technical methods are available in which to relate the quark and hadron masses in this case. We refer to Sects. 3.4.2 and 3.4.3 for discussions of the determination of the light-quark masses from lattice QCD and from chiral perturbation theory. To determine light-quark masses, one can take advantage of chiral perturbation theory, lattice-QCD computations, and QCD sum rule methods. Current progress in the light-quark mass determinations is largely driven by improvements in lattice QCD.

Earlier lattice simulations use $N_\mathrm{f}=2$ flavors of sea quark (recent results include Refs. [35, 36]), while present ones use $N_\mathrm{f}=2+1$ (recent results include Refs. [37–40]). The influence of charmed sea quarks will soon be studied [41, 42]. In addition, some ensembles no longer require chiral extrapolations to reach the physical mass values. The simulations are almost always performed in the isospin limit, $m_u=m_d{=:}m_{ud}$, $m_{ud}=(m_u+m_d)/2$, therefore what one can directly obtain from the lattice is $m_\mathrm{s}$, the average $m_{ud}$, and their ratio. We mention that there is a new strategy to determine the light-quark masses which consists in computing the ratio $m_\mathrm{c}/m_\mathrm{s}$, combined with a separate calculation for $m_\mathrm{c}$, to obtain $m_\mathrm{s}$ [2, 43]. The advantage of this method is that the issue of lattice renormalization is traded for a continuum renormalization in the determination of $m_\mathrm{c}$. With additional input regarding isospin-breaking effects, from the lattice results in the isospin limit one can obtain separate values for $m_u$ and $m_d$; see Sect. 3.4.2 for additional discussion. With the present results, one obtains that $m_u\ne 0$, so that the strong-CP problem is not solved by having a massless $u$ quark [1, 44, 45]; see Sect. 5.7 for further discussion of this issue.

In contrast, heavy-quark masses also affect several processes of interest; for instance, the $b$-quark mass enters in the Higgs decay rate for $H\rightarrow b\bar{b}$. Many studies of Higgs physics do not, however, use the latest, more precise determinations of $m_b$. The value of the top-quark mass is also necessary for precision electroweak fits. To study heavy-quark masses, $m_Q$, one can exploit the hierarchy $m_Q\gg \Lambda _\mathrm{QCD}$ to construct heavy-quark effective theories that simplify the dynamics; and additionally take advantage of high-order, perturbative calculations that are available for these systems; and of progress in lattice-QCD computations. One of the best ways to determine the $b$ and $c$ masses is through sum-rule analyses, that compare theoretical predictions for moments of the cross section for heavy-quark production in $e^+e^{-}$ collisions with experimental data (some analyses that appeared in recent years include [46–49]) or lattice QCD (e.g., [2]). In those analyses, for the case of $m_\mathrm{c}$, the approach with lattice QCD gives the most precise determination, and the errors are mainly driven by perturbative uncertainties. For $m_b$, the approach with $e^+e^{-}$ data still gives a better determination, but expected lattice-QCD progress in the next few years may bring the lattice determination to a similar level of precision. A complementary way to obtain the $c$-quark mass is to exploit DIS charm production measurements in PDF fits [50]. The best measurement of the top-quark mass could be performed at a future $e^+e^{-}$ collider, but improvements on the mass determination, with respect to the present precision, from LHC measurements are possible.

## Light quarks

### Introduction

3The study of light-quark physics is central to the understanding of QCD. Light quarks represent a particularly sensitive probe of the strong interactions, especially of non-perturbative effects.

In the two extreme regimes of QCD, namely, in the low-energy regime where the energies are (much) smaller than a typical strong interaction scale $\sim$ $m_\rho$, and in the high-energy regime where the energies are much higher than that scale, there are well-established theoretical methods, namely, Chiral Perturbation Theory (ChPT) and perturbative QCD, respectively, that allow for a discussion of the physics in a manner consistent with the fundamental theory, and thus permit in this way to define and quantify effects in a more or less rigorous way. The intermediate-energy regime is less developed as there are no analytic methods that need allow for a complete discussion of the physics, thus requiring the introduction of methods which that need require some degree of modeling. However, as discussed in this chapter, methods based fundamentally on QCD, such as those based on the framework of Schwinger–Dyson equations, have made great advances, and a promising future lies ahead. Advances in lattice QCD, in which the excited hadron spectrum can be analyzed, are opening new perspectives for understanding the intermediate-energy regime of QCD; and one should expect that this will result in new strategies, methods, and ideas. Progress on all of the mentioned fronts continues, and in this chapter a representative number of the most exciting developments are discussed.

Never before has the study of the strong interactions had as many sources of experimental results as today. Laboratories and experiments around the world, ranging from low- to high-energy accelerators, as well as in precision nonaccelerator physics, give unprecedented access to the different aspects of QCD, and to light-quark physics in particular. In this chapter a broad sample of experiments and results from these venues will be given.

The objective of this chapter is to present a selection of topics in light-quark physics: partonic structure of light hadrons, low-energy properties and structure, excited hadrons, the role of light-quark physics in extracting fundamental QCD parameters, such as $\alpha _\mathrm{s}$ at the GeV scale, and also of theoretical methods, namely, ChPT, perturbative QCD, Schwinger–Dyson equations, and lattice QCD.

This chapter is organized as follows: Sect. 3.2 is devoted to hadron structure and contains the following topics: parton distributions (also including their transverse momentum dependence), hadron form factors, and generalized parton distributions (GPDs), lattice QCD calculations of form factors and moments of the parton distributions, along with a discussion of the proton radius puzzle; finally, the light pseudoscalar meson form factors, the neutral pion lifetime, and the charged pion polarizabilities complete the section. Section 3.3 deals with hadron spectroscopy and summarizes lattice QCD and continuum methods and results, along with a detailed presentation of experimental results and perspectives. Section 3.4 addresses chiral dynamics, including studies based on ChPT and/or on lattice QCD. In Sect. 3.5 the role of light quarks in precision tests of the Standard Model is discussed, with the hadronic contributions to the muon’s anomalous magnetic moment as a particular focus. The running of the electroweak mixing angle, as studied through the weak charge of the proton, and the determination of the strong coupling ${\alpha _{\mathrm{s}}}$ from $\tau$ decay are also addressed. Finally, Sect. 3.6 presents some thoughts on future directions.

### Hadron structure

#### Parton distribution functions in QCD

The description of hadrons within QCD faces severe difficulties because the strength of the color forces becomes large at low energies and the confinement properties of quarks and gluons cannot be ignored. The main concepts and techniques for treating this non-perturbative QCD regime are discussed in Sect. 8, which is devoted to infrared QCD. Here, we focus on those quantities that enter the description of hadronic processes in which a large momentum scale is involved, thus enabling the application of factorization theorems. Factorization theorems provide the possibility (under certain assumptions) to compute the cross section for high-energy hadron scattering by separating short-distance from long-distance effects in a systematic way. The hard-scattering partonic processes are described within perturbative QCD, while the distribution of partons in a particular hadron—or of hadrons arising from a particular parton in the case of final-state hadrons—is encoded in universal parton distribution functions (PDFs) or parton fragmentation functions (PFFs), respectively. These quantities contain the dynamics of long-distance scales related to non-perturbative physics and thus are taken from experiment.

To see how factorization works, consider the measured cross section in deep-inelastic scattering (DIS) for the generic process lepton + hadron $A \rightarrow \mathrm{lepton^{\prime }}$ + anything else $X$:

3.1 $$\begin{aligned} \mathrm{d}\sigma = \frac{\mathrm{d}^{3}\mathbf {k}'}{2s |\mathbf {k}'|} \frac{1}{(q)^2} L_{\mu \nu }(k,q) W^{\mu \nu }(p,q) \, , \end{aligned}$$

where $k$ and $k'$ are the incoming and outgoing lepton momenta, $p$ is the momentum of the incoming nucleon (or other hadron), $s=(p+k)^2$, and $q$ is the momentum of the exchanged photon. The leptonic tensor $L_{\mu \nu }(k,q)$ is known from the electroweak Lagrangian, whereas the hadronic tensor $W^{\mu \nu }(p,q)$ may be expressed in terms of matrix elements of the electroweak currents to which the vector bosons couple, viz., [51]

3.2 $$\begin{aligned} W^{\mu \nu }= \frac{1}{4\pi } \int _{}^{}\mathrm{d}^4y e^{iq\cdot y} \sum _{X} \left\langle A|j^\mu (y)|X\right\rangle \left\langle X|j^\nu (0)|A\right\rangle \, .\nonumber \\ \end{aligned}$$

For $Q^2=-q^2$ large and Bjorken $x_B=Q^2/2p\cdot q$ fixed, $W^{\mu \nu }$ can be written in the form of a factorization theorem to read

3.3 $$\begin{aligned} W^{\mu \nu }(p,q)&= \sum _{a} \int _{x_B}^{1} \frac{\mathrm{d}x}{x}f_{a/A}(x, \mu ) \nonumber \\&\times H_{a}^{\mu \nu }(q,xp, \mu , \alpha _\mathrm{s}(\mu )) + \text{ remainder }, \end{aligned}$$

where $f_{a/A}(x, \mu )$ is the PDF for a parton $a$ (gluon, $u$, $\bar{u}$, $\ldots$) in a hadron $A$ carrying a fraction $x$ of its momentum and probed at a factorization scale $\mu$, $H^{\mu \nu }_a$ is the short-distance contribution of partonic scattering on the parton $a$, and the sum runs over all possible types of parton $a$. In (3.3), the (process-dependent) remainder is suppressed by a power of $Q$.

In DIS experiments, $lA \rightarrow l^{\prime }X$, we learn about the longitudinal distribution of partons inside hadron $A$, e.g., the nucleon. The PDF for a quark $q$ in a hadron $A$ can be defined in a gauge-invariant way (see [51] and references cited therein) in terms of the following matrix element:

3.4 $$\begin{aligned} f_{q/A}(x,\mu )&= \frac{1}{4\pi } \int _{}^{} dy^{-} e^{-i x p^{+} y^{-}} \langle p| \bar{\psi }(0^+,y^{-},\mathbf{{0}}_\mathrm{T}) \nonumber \\&\times \gamma ^{+} \mathcal {W}(0^{-},y^{-}) \psi (0^+,0^{-},\mathbf{{0}}_\mathrm{T}) |p \rangle \, , \end{aligned}$$

where the light-cone notation, $v^{\pm }=(v^0\pm v^3)/\sqrt{2}$ for any vector $v^\mu$, was used. Here, $\mathcal {W}$ is the Wilson line operator in the fundamental representation of $\mathrm{SU}(3)_\mathrm{c}$,

3.5 $$\begin{aligned} \mathcal {W}(0^{-},y^{-}) = \mathcal{P} \exp \left[ ig \int _{0^{-}}^{y^{-}} dz^{-} A_{a}^{+}(0^+, z^{-}, {\mathbf {0}}_\mathrm{T})t_a \right] \nonumber \\ \end{aligned}$$

along a lightlike contour from $0^{-}$ to $y^{-}$ with a gluon field $A_a^\mu$ and the generators $t_a$ for $a=1,2,\dots ,8$. Here, $g$ is the gauge coupling, such that ${\alpha _{\mathrm{s}}}=g^2/4\pi$. Analogous definitions hold for the antiquark and the gluon—the latter in the adjoint representation. These collinear PDFs (and also the fragmentation functions) represent light-cone correlators of leading twist in which gauge invariance is ensured by lightlike Wilson lines (gauge links). The factorization scale $\mu$ dependence of PDFs is controlled by the DGLAP (Dokshitzer–Gribov–Lipatov–Altarelli–Parisi) [52–54] evolution equation [55, 56]. The PDFs represent the universal part in the factorized cross section of a collinear process such as (3.3). They are independent of any specific process in which they are measured. It is just this universality of the PDFs that ensures the predictive power of the factorization theorem. For example, the PDFs for the Drell–Yan (DY) process [57] are the same as in DIS, so that one can measure them in a DIS experiment and then use them to predict the DY cross section [51, 58].

The predictive power of the QCD factorization theorem also relies on our ability to calculate the short-distance, process-specific partonic scattering part, such as $H_{a}^{\mu \nu }$ in (3.3), in addition to the universality of the PDFs. Since the short-distance partonic scattering part is insensitive to the long-distance hadron properties, the factorization formalism for scattering off a hadron in (3.3) should also be valid for scattering off a partonic state. Applying the factorization formalism to various partonic states $a$, instead of the hadron $A$, the short-distance partonic part, $H^{\mu \nu }_a$ in (3.3), can be systematically extracted by calculating the partonic scattering cross section on the left and the PDFs of a parton on the right of (3.3), order-by-order in powers of $\alpha _\mathrm{s}$ in perturbative QCD. The validity of the collinear factorization formalism ensures that any perturbative collinear divergence of the partonic scattering cross section on the left is completely absorbed into the PDFs of partons on the right. The Feynman rules for calculating PDFs and fragmentation functions have been derived in [55, 56] having recourse to the concept of eikonal lines and vertices. Proofs of factorization of DIS and the DY process can be found in [51] and the original works cited therein.

One of the most intriguing aspects of QCD is the relation between its fundamental degrees of freedom, quarks and gluons, and the observable hadrons, such as the proton. The PDFs are the most prominent non-perturbative quantities describing the relation between a hadron and the quarks and gluons within it. The collinear PDFs, $f(x\!,\mu )$, give the number density of partons with longitudinal momentum fraction $x$ in a fast-moving hadron, probed at the factorization scale $\mu$. Although they are not direct physical observables, as the cross sections of leptons and hadrons are, they are well defined in QCD and can be systematically extracted from data of cross sections, if the factorization formulas of the cross sections with perturbatively calculated short-distance partonic parts are used. Our knowledge of PDFs has been much improved throughout the years by many surprises and discoveries from measurements at low-energy, fixed-target experiments to those at the LHC—the highest energy hadron collider in the world. The excellent agreement between the theory and data on the factorization scale $\mu$-dependence of the PDFs has provided one of the most stringent tests for QCD as the theory of strong interaction. Many sets of PDFs have been extracted from the QCD global analysis of existing data, and a detailed discussion of the extraction of PDFs will be given in the next subsection.

**Fig. 1:**
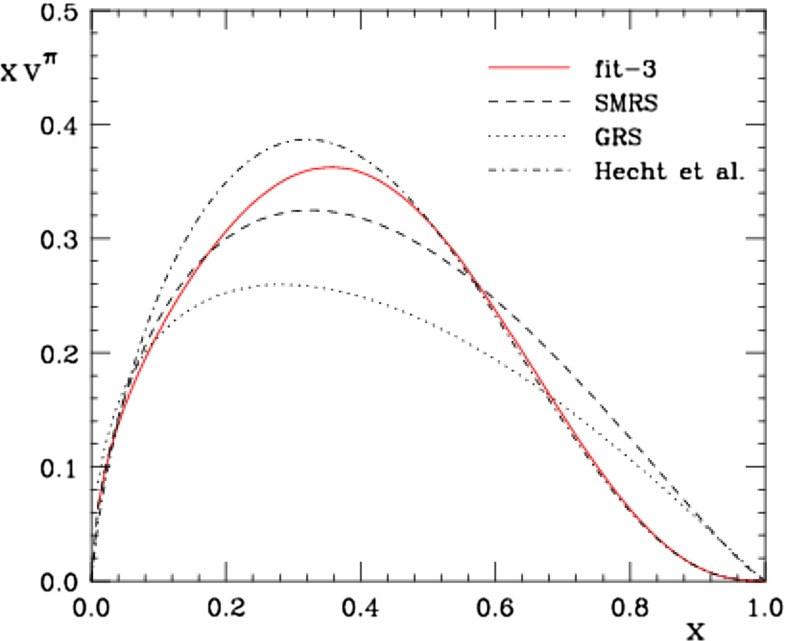
Valence distribution of the pion obtained in [59] from a fit to the E615 Drell–Yan data [60] at $Q=4$ GeV, compared to the NLO parameterizations of [61] Sutton–Martin–Roberts–Stirling (SMRS) and [62] Glück–Reya–Schienbein (GRS) and to the distribution obtained from Dyson–Schwinger equations by Hecht et al. [63]. From [59]

Understanding the characteristics and physics content of the extracted PDFs, such as the shape and the flavor dependence of the distributions, is a necessary step in searching for answers to the ultimate question in QCD: of how quarks and gluons are confined into hadrons. However, the extraction of PDFs depends on how well we can control the accuracy of the perturbatively calculated short-distance partonic parts. As an example, consider the pion PDF. Quite recently, Aicher, Schäfer, and Vogelsang [59] addressed the impact of threshold resummation effects on the pion’s valence distribution $v^\pi \equiv u_v^{\pi ^+}\!=\! \bar{d}_v^{\pi ^+}\!=\!d_v^{\pi ^{-}}\!=\! \bar{u}_v^{\pi ^{-}}$ using a fit to the pion–nucleon E615 DY data [60]. They found a fall-off much softer than linear, which is compatible with a valence distribution behaving as $xv^{\pi }=(1-x)^{2.34}$ (see Fig. 1). This softer behavior of the pion’s valence PDF is due to the resummation of large logarithmic higher-order corrections—“threshold logarithms”—that become particularly important in the kinematic regime accessed by the fixed-target DY data for which the ratio $Q^2/s$ is large. Here $Q$ and $\sqrt{s}$ denote the invariant mass of the lepton pair and the overall hadronic center-of-mass energy, respectively. Because threshold logarithms enhance the cross section near threshold, the fall-off of $v^\pi$ becomes softer relative to previous NLO analyses of the DY data. This finding is in agreement with predictions from perturbative QCD [61, 62] in the low-$x$ regime and from Dyson–Schwinger equation approaches [63] in the whole $x$ region. Moreover, it compares well with the CERN NA10 [64] DY data, which were not included in the fit shown in Fig. 1 (see [59] for details). Resummation effects on the PDFs in the context of DIS have been studied in [65].

Going beyond a purely longitudinal picture of hadron structure, one may keep the transverse (spacelike) degrees of freedom of the partons unintegrated and achieve in this way a three-dimensional image of the hadronic structure by means of transverse-momentum-($k_\mathrm{T}$)-dependent (TMD) distribution and fragmentation functions; see, e.g., [66] for a recent review. Such $x$- and $k_{T}$-dependent quantities provide a useful tool to study semi-inclusive deep inelastic scattering (SIDIS) $lH^{\uparrow } \rightarrow l^{\prime } h X$ (HERMES, COMPASS, JLab at 12 GeV experiments), the Drell–Yan (DY) process $H_{1}^{(\uparrow )} H_{2}^{\uparrow } \rightarrow l^{+} l^{-} X$ (COMPASS, PAX, GSI, RHIC experiments), or lepton-lepton annihilation to two almost back-to-back hadrons $e^{+}e^{-} \rightarrow h_{1} h_{2} X$ (Belle, BaBar experiments), in which events naturally have two very different momentum scales: $Q \gg q_\mathrm{T}$, where $Q$ is the invariant mass of the exchanged vector boson, e.g., $\gamma ^*$ or $Z^0$, and $q_\mathrm{T}$ is the transverse momentum of the observed hadron in SIDIS or the lepton-pair in DY, or the momentum imbalance of the two observed hadrons in $e^+e^{-}$ collisions. It is the two-scale nature of these scattering processes and corresponding TMD factorization formalisms [58, 67, 68] that enable us to explore the three-dimensional motion of partons inside a fast moving hadron. The large scale $Q$ localizes the hard collisions of partons, while the soft scale $q_\mathrm{T}$ provides the needed sensitivity to access the parton $k_\mathrm{T}$. Such a two-scale nature makes these observables most sensitive to both the soft and collinear regimes of QCD dynamics, and has led to the development of the soft-collinear effective theory approach in QCD (see Sect. 7.2.1 for more details and references).

In contrast to collinear PDFs which are related to collinear leading-twist correlators and involve only spin-spin densities, TMD PDFs (or simply, TMDs) parameterize spin-spin and momentum-spin correlations, and also single-spin and azimuthal asymmetries, such as the Sivers [69] and Collins [70, 71] effects in SIDIS. The first effect originates from the correlation of the distribution of unpolarized quarks in a nucleon with the transverse polarization vector $S_\mathrm{T}$. The second one stems from the similar correlation between $k_\mathrm{T}$ and $S_\mathrm{T}$ in the fragmentation function related to the quark polarization. The important point is that the Sivers asymmetry in the DY process flips sign relative to the SIDIS situation owing to the fact that the corresponding Wilson lines point in opposite time directions as a consequence of time reversal. This directional (path) dependence breaks the universality of the distribution functions in SIDIS, DY production, $e^{+} e^{-}$ annihilation [72], and other hadronic processes that contain more complicated Wilson lines [73], and lead to a breakdown of the TMD factorization [74–77]. On the other hand, the Collins function seems to possess universal properties in SIDIS and $e^{+} e^{-}$ processes [78]. Both asymmetries have been measured experimentally in the SIDIS experiments at HERMES, COMPASS, and JLab Hall A [79–83]. The experimental test of the breakdown of universality, i.e., a signal of process dependence, in terms of these asymmetries and their evolution effects is one of the top-priority tasks in present-day hadronic physics and is pursued by several collaborations.

**Fig. 2:**
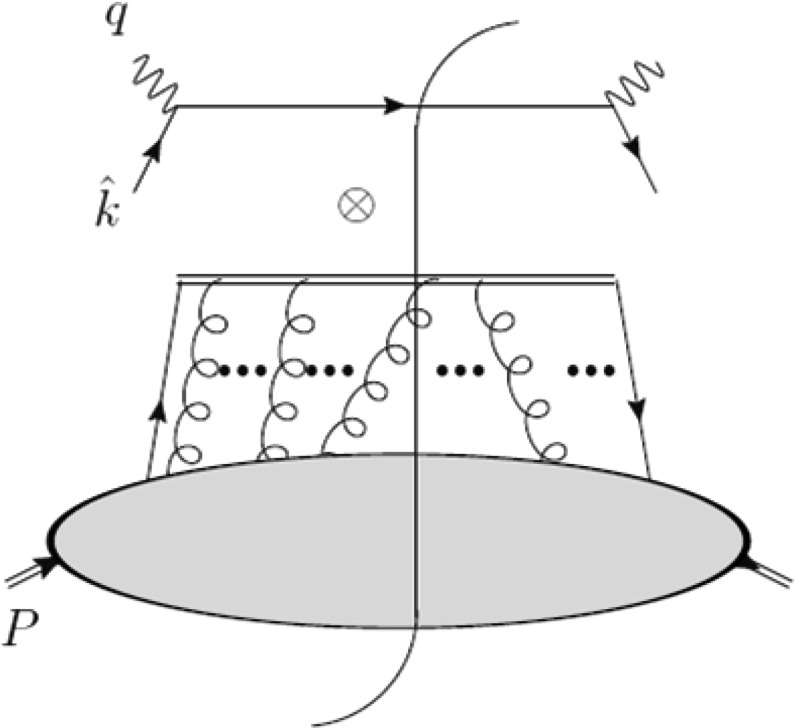
Factorization for SIDIS of extra gluons into gauge links (*double lines*). Figure from [66]

Theoretically, the effects described above arise because the TMD field correlators have a more complicated singularity structure than PDFs, which is related to the lightlike and transverse gauge links entering their operator definition [84–86]:

3.6 $$\begin{aligned}&\Phi _{ij}^{q[C]}(x, {\mathbf {k}}_{T};n) = \int \frac{d(y\cdot p) \, \mathrm{d}^2 \varvec{y}_{T}}{(2\pi )^3} e^{-ik \cdot y} \nonumber \\&\quad \times \left\langle p| \bar{\psi }_{j}(y)\mathcal {W}(0,y|C)\psi _{i}(0) |p\right\rangle _{y\cdot n=0}, \end{aligned}$$

where the contour $C$ in the Wilson line $\mathcal {W}(0,y|C)$ has to be taken along the color flow in each particular process. For instance, in the SIDIS case (see Fig. 2 for an illustration), the correlator contains a Wilson line at $\infty ^{-}$ that does not reduce to the unity operator by imposing the light-cone gauge $A^{+}=0$. This arises because in order to have a closed Wilson line, one needs in addition to the two eikonal attachments pointing in the minus direction on either side of the cut in Fig. 2, an additional detour in the transverse direction. This detour is related to the boundary terms that are needed as subtractions to make higher-twist contributions gauge invariant, see [66] for a discussion and references. Hence, the sign reversal between the SIDIS situation and the DY process is due to the change of a future-pointing Wilson line into a past-pointing Wilson line as a consequence of CP invariance (noting CPT is conserved in QCD) [71]. In terms of Feynman diagrams this means that the soft gluons from the Wilson line have “cross-talk” with the quark spectator (or the target remnant) after (before) the hard scattering took place, which emphasizes the importance of the color flow through the network of the eikonal lines and vertices. The contribution of the twist-three fragmentation function to the single transverse spin asymmetry in SIDIS within the framework of the $k_\mathrm{T}$ factorization is another open problem that deserves attention.

The imposition of the light-cone gauge $A^{+}=0$ in combination with different boundary conditions on the gluon propagator makes the proof of the TMD factorization difficult—already at the one-loop order—and demands the introduction of a soft renormalization factor to remove unphysical singularities [87–89]. One may classify the emerging divergences into three main categories: (i) ultraviolet (UV) poles stemming from large loop momenta that can be removed by dimensional regularization and minimal subtraction, (ii) rapidity divergences that can be resummed by means of the Collins–Soper–Sterman (CSS) [90] evolution equation in impact-parameter space, and (iii) overlapping UV and rapidity divergences that demand a generalized renormalization procedure to obtain a proper operator definition of the TMD PDFs. Rapidity divergences correspond to gluons moving with infinite rapidity in the opposite direction of their parent hadron and can persist even when infrared gluon mass regulators are included, in contrast to the collinear case in which rapidity divergences cancel in the sum of graphs. Their subtraction demands additional regularization parameters, beyond the usual renormalization scale $\mu$ of the modified-minimal-subtraction ($\overline{\mathrm{MS}}$) scheme.

Different theoretical schemes have been developed to deal with these problems and derive well-defined expressions for the TMD PDFs. Starting from the factorization formula for the semi-inclusive hadronic tensor, Collins [58] recently proposed a definition of the quark TMD PDF which absorbs all soft renormalization factors into the distribution and fragmentation functions, expressing them in the impact-parameter $b_\mathrm{T}$ space. Taking the limit $b_\mathrm{T}\rightarrow 0$, these semi-integrated PDFs reduce to the collinear case.

However, this framework has been formulated in the covariant Feynman gauge in which the transverse gauge links vanish so that it is not clear how to treat T-odd effects in axial gauges within this framework. Moreover, the CSS $b_\mathrm{T}$-space approach [90] to the evolution of the TMD PDFs requires an extrapolation to the non-perturbative large-$b_\mathrm{T}$ region in order to complete the Fourier transform in $b_\mathrm{T}$ and derive the TMDs in $k_\mathrm{T}$-space. Different treatments or approximations of the non-perturbative extrapolation could lead to uncertainties in the derived TMDs [91]. For example, the TMDs based on Collins’ definition predicts [92–94] asymmetries for DY processes that are a bit too small, while a more recent analysis [95, 96], which derives from the earlier work in [67, 68, 97] employing a different treatment on the extrapolation to the large $b_\mathrm{T}$ region, seems to describe the evolution of the TMD PDF for both the SIDIS and the DY process in the range $2$–$100$ GeV$^2$ reasonably well.

An alternative approach [98–100] to eliminate the overlapping UV-rapidity divergences employs the renormalization-group properties of the TMD PDFs to derive an appropriate soft renormalization factor composed of Wilson lines venturing off the light cone in the transverse direction along cusped contours. The soft factor encodes contributions from soft gluons with nearly zero center-of-mass rapidity. The presence of the soft factor in the approach of [98–100], entailed by cusp singularities in the Wilson lines, obscures the derivation of a correct factorization because it is not clear how to split and absorb it into the definition of the TMD PDFs to resemble the collinear factorization theorem. An extension of this approach, relevant for spin observables beyond leading twist, was given in [101].

Several different schemes to study TMD PDFs and their evolution have also been proposed [102–113], which are based on soft collinear effective theory (SCET). One such framework [108–110] has been shown in [114] to yield equivalent results to those obtained by Collins in [58]. A detailed comparison of the Ji-Ma-Yuan scheme [68, 97] with that of Collins [58] was given in [96]. The universality of quark and gluon TMDs has been studied in a recent work by Mulders and collaborators [115] in which it was pointed out that the whole process (i.e., the gauge link) dependence can be isolated in gluonic pole factors that multiply the universal TMDs of definite rank in the impact-parameter space. An analysis of non-perturbative contributions at the next-to-next-to-leading-logarithmic (NNLL) level to the transverse-momentum distribution of $Z/\gamma ^*$ bosons, produced at hadron colliders, has been presented in [116].

Last but not least, Sudakov resummation within $k_\mathrm{T}$ factorization of single and double logarithms is an important tool not only for Higgs boson production in $pA$ collisions, but also for heavy-quark pair production in DIS, used in the theoretical study of saturation phenomena that can be accessed experimentally at RHIC and the LHC (see, [117] for a recent comprehensive analysis). All these achievements notwithstanding, the TMD factorization formalism and the theoretical framework for calculating the evolution of TMD PDFs and radiative corrections to short-distance dynamics beyond one-loop order have not been fully developed. Complementary to these studies, exploratory calculations of TMD nucleon observables in dynamical lattice QCD have also been performed, which employ nonlocal operators with “staple-shaped,” process-dependent Wilson lines—see, for instance, [118].

#### PDFs in the DGLAP approach

The PDFs are essential objects in the phenomenology of hadronic colliders and the study of the hadron structure. In the collinear factorization framework, the PDFs are extracted from fits to experimental data for different processes—they are so-called global fits. The typical problem that a global fit solves is to find the set of parameters $\{p_i\}$ that determine the functional form of the PDFs at a given initial scale $Q_0^2$, $f_i(x,Q^2_0,\{p_i\})$ so that they minimize a quality criterion in comparison with the data, normally defined by the best $\chi ^2$. The calculation of the different observables involves i) the evolution of the PDFs to larger scales $Q^2>Q^2_0$ by means of the DGLAP evolution equations and ii) the computation of this observable by the factorized hard cross section at a given order in QCD. Several observables are known at next-to-next-to-leading order (NNLO) at present, and this order is needed for precision analyses. This conceptually simple procedure has been tremendously improved during the last years to cope with the stringent requirements of more and more precise analyses of the data in the search of either Standard Model or Beyond the Standard Model physics. For recent reviews on the topic we refer the readers to [119–122].

A standard choice of the initial parameterization, motivated by Regge theory, is

3.7 $$\begin{aligned} f_i(x,Q^2_0)=x^{\alpha _i}(1-x)^{\beta _i}g_i(x), \end{aligned}$$

where $g_i(x)$ is a function whose actual form differs from group to group. Typical modern sets involve of the order of 30 free parameters and the released results include not only the best fit (the central value PDFs) but also the set of *error* PDFs to be used to compute uncertainty bands. These uncertainties are based on Hessian error analyses which provide eigenvectors of the covariance matrix (ideally) determined by the one-sigma confidence level criterion or $\chi ^2=\chi ^2_\mathrm{min}+\Delta \chi ^2$, with $\Delta \chi ^2=1$. Notice, however, that when applied to a large set of experimental data from different sources it has long been realized that a more realistic treatment of the uncertainties requires the inclusion of a *tolerance* factor $T$ so that $\Delta \chi ^2=T^2$ [123, 124].

An alternative approach which naturally includes the study of the uncertainties is based on Monte Carlo [125], usually by constructing replicas of the experimental data which encode their covariance matrix. This approach is employed by the NNPDF Collaboration [125, 126], which also makes use of neural networks for the parameterizations of (3.7). In this case, the neural networks provide an unbiased set of basis functions in the functional space of the PDFs. The Monte Carlo procedure provides a number of PDF replicas $N_\mathrm{rep}$ and any observable is computed by averaging over these $N_\mathrm{rep}$ sets of PDFs. The main advantage of this method is that it does not require assumptions on the form of the probability distribution in parameter space (assumed to be a multi-dimensional Gaussian in the procedure explained in the previous paragraph). As a bonus, the method also provides a natural way of including new sets of data or checking the compatibility of new sets of data, without repeating the tedious and time-consuming procedure of a whole global fit. Indeed, in this approach, including a new set of data would change the relative weights of each of the $N_\mathrm{rep}$ sets of PDFs, so that a new observable can be computed by averaging over the $N_\mathrm{rep}$ sets now each one with a different weight [127–129]. This *Bayesian reweighing* procedure has also been adapted to the Hessian errors PDFs, where a Monte Carlo representation is possible by simply generating the PDF sets through a multi-Gaussian distribution in the parameter space [130].

**Fig. 3:**
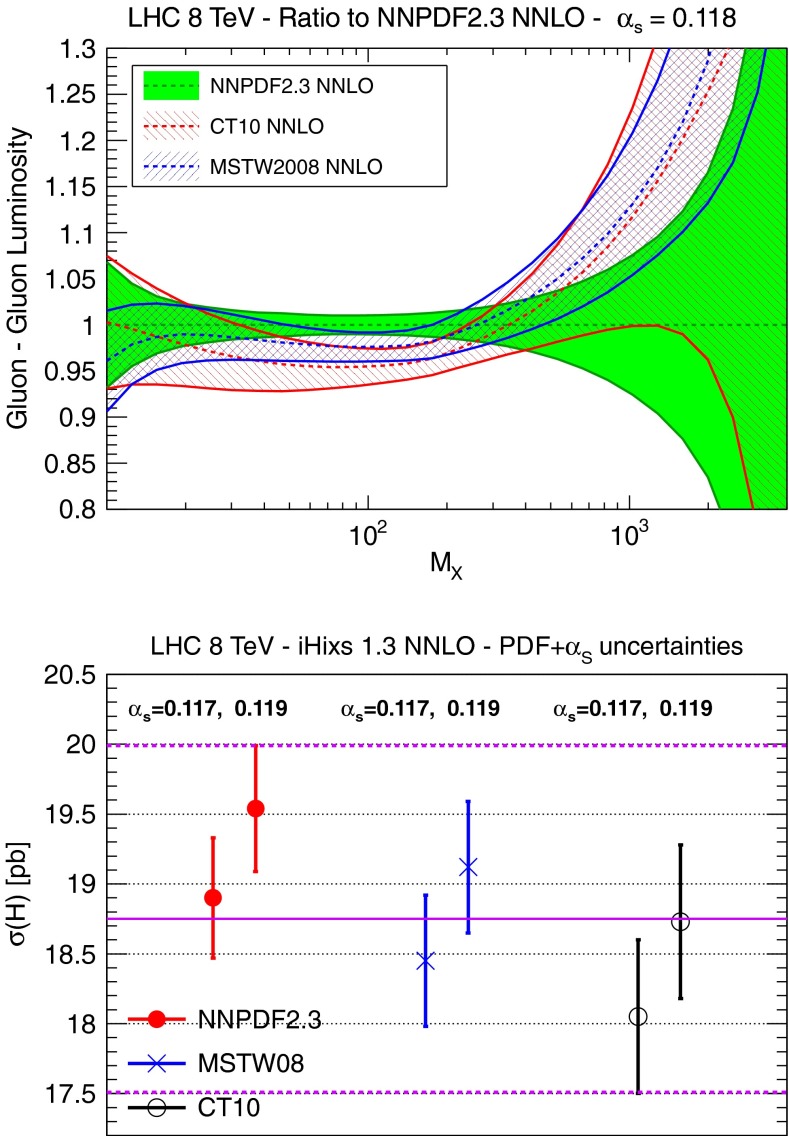
(*Upper figure*) Gluon–gluon luminosity to produce a resonance of mass $M_X$ for different PDFs normalized to that of NNPDF 2.3. (*Lower figure*) The corresponding uncertainties in the Higgs cross section from PDFs and $\alpha _\mathrm{s}(M_Z)$. Figures from [136]

Modern sets of unpolarized PDFs for the proton include MSTW08 [131], CT10 [132], NNPDF2.3 [133], HERAPDF [134], ABM11 [8], and CJ12 [135]. Comparison of some of these sets can be found in Fig. 3 as well as of their corresponding impact on the computation of the Higgs cross section at NNLO [136]. Following similar procedures, nuclear PDFs are also available, that is, nCTEQ [137], DSSZ [138], EPS09 [139], and HKN07 [140], as are polarized PDFs [141–145].

#### PDFs and nonlinear evolution equations

Linear evolution equations such as the DGLAP or the Balitsky–Fadin–Kuraev–Lipatov (BFKL) equations assume a branching process in which each parton in the hadronic wave function splits into two lower-energy ones. The divergence of this process in the infrared makes the distributions more and more populated in the small-$x$ region of the wave function. In this situation it was proposed long ago that a phenomenon of saturation of partonic densities should appear at small enough values of the fraction of momentum $x$ [146], or otherwise the unitarity of the scattering amplitudes would be violated. This idea has been further developed into a complete and coherent formalism known as the Color Glass Condensate (CGC, see, e.g., [147] for a recent review).

**Fig. 4:**
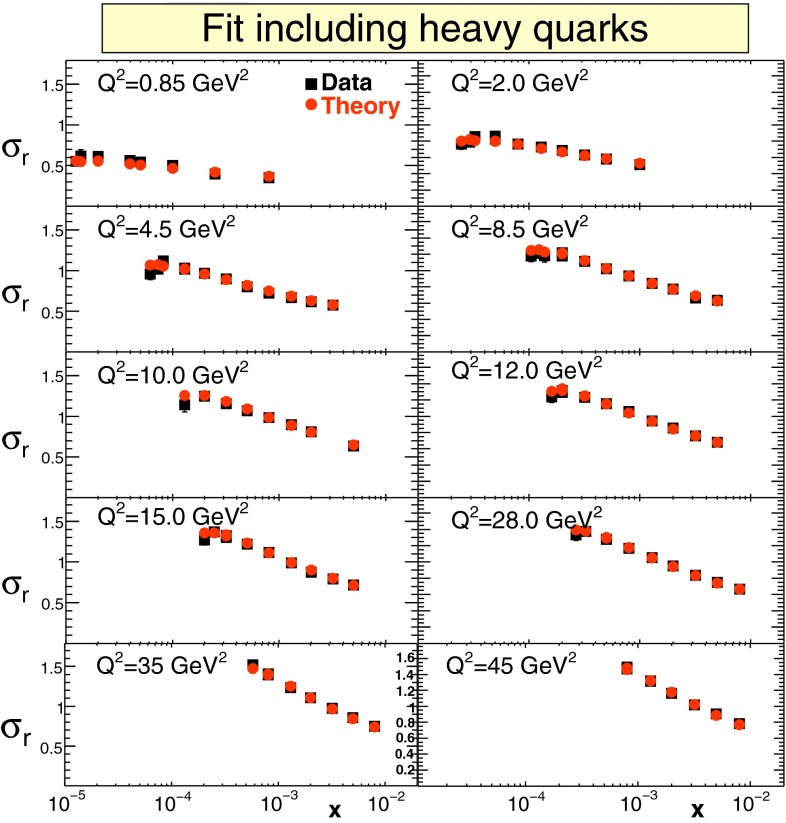
Fit using the NLO BK nonlinear evolution equations of the combined H1/ZEUS HERA data. Figure from [161]

The CGC formalism is usually formulated in terms of correlators of Wilson lines on the light cone in a color singlet state. The simplest one contains two Wilson lines and can be related to the dipole cross section; higher-order correlators can sometimes be simplified to the product of two-point correlators, especially in the large-$N_\mathrm{c}$ limit [148]. The nonlinear evolution equation of the dipole amplitudes is known in the large-$N_\mathrm{c}$ limit with NLO accuracy [149–152], and the LO version of it is termed the Balitsky–Kovchegov equation [153, 154]. The evolution equations at finite-$N_\mathrm{c}$ are known as the B-JIMWLK equations (using the acronyms of the authors in [153, 155–159]) and can be written as an infinite hierarchy of coupled nonlinear differential equations in the rapidity variable, $Y=\log (1/x)$, of the n-point correlators of the Wilson lines. These equations are very difficult to solve numerically. However, it has been checked that in the large-$N_\mathrm{c}$ approximation, the BK equations provide very accurate results [160]. The NLO BK equations (or rather their leading NLO contributions) provide a good description of the HERA and other small-$x$ physics data with a reduced number of free parameters [161] (Fig. 4).

One of the main interests of the CGC formalism is that it provides a general framework in which to address some of the fundamental questions in the theory of high-energy nucleus-nucleus collisions, in particular, with respect to the initial stages in the formation of a hot and dense QCD medium and how local thermal equilibrium is reached (see, e.g., [162] and references therein). The phenomenological analyses of different sets of data in such collisions deal with the multiplicities [163]; the ridge structure in the two-particle correlations in proton-nucleus collisions, which indicate very long-range rapidity correlations [164]; or the coupling of the CGC-initial conditions with a subsequent hydrodynamical evolution [165]. These are just examples of the potentialities of the formalism to provide a complete description of such complicated systems.

#### GPDs and tomography of the nucleon

Quarks and gluons carry color charge, and it is very natural to ask how color is distributed inside a bound and color neutral hadron. Knowing the color distribution in space might shed some light on how color is confined in QCD. Unlike the distribution of electromagnetic charge, which is given by the Fourier transform of the nucleon’s electromagnetic form factors (see the next subsection), it is very unlikely, if not impossible, to measure the spatial distribution of color in terms of scattering cross sections of color-neutral leptons and hadrons. This is because the gluon carries color, so that the nucleon cannot rebound back into a nucleon after absorbing a gluon. In other words, there is no elastic nucleon color form factor. Fortunately, in the last 20 years, remarkable progress has been made in both theory and experiment to make it possible to obtain spatial distributions of quarks and gluons inside the nucleons. These distributions, which are also known as tomographic images, are encoded in generalized parton distribution functions (GPDs) [166, 167].

GPDs are defined in terms of generalized parton form factors [168], e.g., for quarks,

3.8 $$\begin{aligned}&F_{q}(x,\xi ,t) \!=\!\! \int \!\frac{dy^{-}}{2\pi } e^{-i x p^{+} y^{-}} \langle p'| \bar{\psi }({\textstyle \frac{1}{2}}y^{-}){\textstyle \frac{1}{2}}\gamma ^{+} \psi (-{\textstyle \frac{1}{2}}y^{-}) |p \rangle \nonumber \\&\quad \equiv H_q(x,\xi ,t) \left[ \overline{\mathcal{U}}(p')\gamma ^\mu \mathcal{U}(p)\right] \frac{n_{\mu }}{p\cdot n} \nonumber \\&\quad \quad + E_q(x,\xi ,t) \left[ \overline{\mathcal{U}}(p') \frac{i\sigma ^{\mu \nu }(p'-p)_{\nu }}{2M} \mathcal{U}(p) \right] \frac{n_{\mu }}{p\cdot n}, \end{aligned}$$

where the gauge link between two quark field operators and the factorization scale dependence are suppressed, $\mathcal{U}$’s are hadron spinors, $\xi =(p'-p)\cdot n/2$ is the skewness, and $t=(p'-p)^2$ is the squared hadron momentum transfer. In (3.8), the factors $H_q(x,\xi ,t)$ and $E_q(x,\xi ,t)$ are the quark GPDs. Unlike PDFs and TMDs, which are defined in terms of forward hadronic matrix elements of quark and gluon correlators, like those in (3.4) and (3.6), GPDs are defined in terms of non-forward hadronic matrix elements, $p'\ne p$. Replacing the $\gamma ^\mu$ by $\gamma ^\mu \gamma _5$ in (3.8) then defines two additional quark GPDs, $\widetilde{H}_q(x,\xi ,t)$ and $\widetilde{E}_q(x,\xi ,t)$. Similarly, gluon GPDs are defined in terms of nonforward hadronic matrix elements of gluon correlators.

Taking the skewness $\xi \rightarrow 0$, the squared hadron momentum transfer $t$ becomes $-{\overrightarrow{\Delta }_{\perp }^2}$. Performing a Fourier transform of GPDs with respect to ${\overrightarrow{\Delta }}_\perp$ gives the joint distributions of quarks and gluons in their longitudinal momentum fraction $x$ and transverse position $b_\perp$, $f_a(x,b_\perp )$ with $a=q,\bar{q},g$, which are effectively equal to the tomographic images of quarks and gluons inside the hadron. Combining the GPDs and TMDs, one could obtain a comprehensive three-dimensional view of the hadron’s quark and gluon structure.

Taking the moments of GPDs, $\int \mathrm{d}x\, x^{n-1} H_a(x,\xi ,t)$ with $a=q,\bar{q},g$, gives generalized form factors for a large set of local operators that can be computed with lattice QCD, as discussed in the next subsection, although they cannot be directly measured in experiments. This connects the hadron structure to lattice QCD—one of the main tools for calculations in the non-perturbative sector of QCD. For example, the first moment of the quark GPD, $Hq(x, 0, t)$, with an appropriate sum over quark flavors, is equal to the electromagnetic Dirac form factor $F_1(t)$, which played a major historical role in exploring the internal structure of the proton.

GPDs also play a critical role in addressing the outstanding question of how the total spin of the proton is built up from the polarization and the orbital angular momentum of quarks, antiquarks, and gluons. After decades of theoretical and experimental effort following the European Muon Collaboration’s discovery [169], it has been established that the polarization of all quarks and antiquarks taken together can only account for about 30 % of the proton’s spin, while about 15 % of proton’s spin likely stems from gluons, as indicated by RHIC spin data [170]. Thus, after all existing measurements, about one half of the proton’s spin is still not explained, which is a puzzle. Other possible additional contributions from the polarization of quarks and gluons in unmeasured kinematic regions, related to the orbital momentum of quarks and gluons, could be the major source of the missing portion of the proton’s spin. In fact, some GPDs are intimately connected with the orbital angular momentum carried by quarks and gluons [171]. Ji’s sum rule is one of the examples that quantify this connection [172],

3.9 $$\begin{aligned} J_q = \frac{1}{2} \lim _{t\rightarrow 0} \int _0^1 \mathrm{d}x\, x \left[ H_q(x,\xi ,t) + E_q(x,\xi ,t) \right] , \end{aligned}$$

which represents the total angular momentum $J_q$ (including both helicity and orbital contributions) carried by quarks and antiquarks of flavor $q$. A similar relation holds for gluons. The $J_q$ in (3.9) is a generalized form factor at $t=0$ and could be computed in lattice QCD [173].

GPDs have been introduced independently in connection with the partonic description of deeply virtual Compton scattering (DVCS) by Müller et al. [174], Ji [175], and Radyushkin [176]. They have also been used to describe deeply virtual meson production (DVMP) [177, 178], and more recently timelike Compton scattering (TCS) [179]. Unlike PDFs and TMDs, GPDs are defined in terms of correlators of quarks and gluons at the amplitude level. This allows one to interpret them as an overlap of light-cone wave functions [180–182]. Like PDFs and TMDs, GPDs are not direct physical observables. Their extraction from experimental data relies upon QCD factorization, which has been derived at the leading twist-two level for transversely polarized photons in DVCS [178] and for longitudinally polarized photons in DVMP [183]. The NLO corrections to the quark and gluon contributions to the coefficient functions of the DVCS amplitude were first computed by Belitsky and Müller [184]. The NLO corrections to the crossed process, namely, TCS, have been derived by Pire et al. [185].

Initial experimental efforts to measure DVCS and DVMP have been carried out in recent years by collaborations at HERA and its fixed target experiment HERMES, as well as by collaborations at JLab and the COMPASS experiment at CERN. To help extract GPDs from cross-section data for exclusive processes, such as DVCS and DVMP, various functional forms or representations of GPDs have been proposed and used for comparing with existing data. Radyushkin’s double distribution ansatz (RDDA) [176, 186] has been employed in the Goloskokov–Kroll model [187–189] to investigate the consistency between the theoretical predictions and the data from DVMP measurements. More discussions and references on various representations of GPDs can be found in a recent article by Müller [168].

**Fig. 5:**
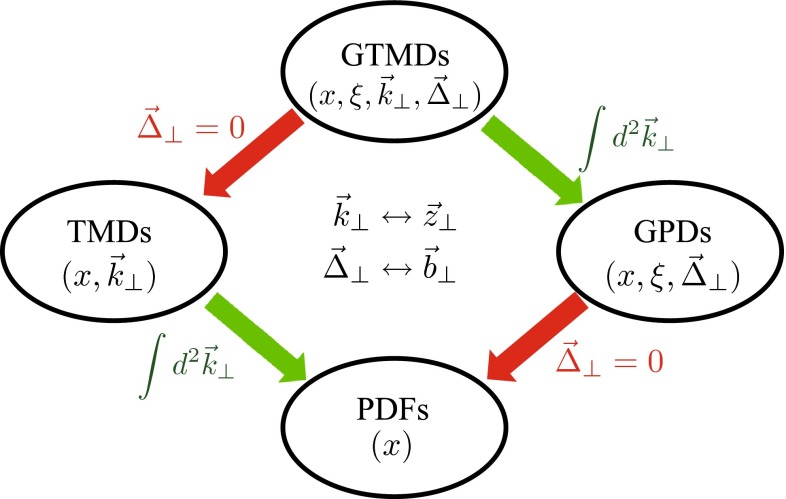
Connections among various partonic amplitudes in QCD. The abbreviations are explained in the text

The PDFs, TMDs, and GPDs represent various aspects of the same hadron’s quark and gluon structure probed in high-energy scattering. They are not completely independent and, actually, they are encoded in the so-called *mother distributions*, or the generalized TMDs (GTMDs), which are defined as TMDs with non-forward hadronic matrix elements [190, 191]. In addition to the momentum variables of the TMDs, $x$ and ${\overrightarrow{k}}\!\!_\perp$, GTMDs also depend on variables of GPDs, the skewness $\xi$ and the hadron momentum transfer $\Delta ^\mu =(p'-p)^\mu$ with $t=\Delta ^2$. The Fourier transform of GTMDs can be considered as Wigner distributions [192], the quantum-mechanical analog of classical phase-space distributions. The interrelationships between GTMDs and the PDFs, TMDs, and GPDs are illustrated in Fig. 5.

Comprehensive and dedicated reviews on the derivation and phenomenology of GPDs can be found in Refs. [168, 193–197]. More specific and recent reviews of the GPD phenomenology and global analysis of available data can be found in Ref. [198] for both the DVCS and DVMP processes, and in Ref. [199] for DVCS asymmetry measurements of different collaborations pertaining to the decomposition of the nucleon spin.

With its unprecedented luminosity, the updated 12 GeV program at JLab will provide good measurements of both DVCS and DVMP, which will be an excellent source of information on quark GPDs in the valence region. It is the future Electron–Ion Collider (EIC) that will provide the ultimate information on both quark and gluon GPDs, and the tomographic images of quarks and gluons inside a proton with its spin either polarized or unpolarized [200].

#### Hadron form factors

The internal structure of hadrons—most prominently of the nucleon—has been the subject of intense experimental and theoretical activities for decades. Many different experimental facilities have accumulated a wealth of data, mainly via electron–proton ($ep$) scattering. Electromagnetic form factors of the nucleon have been measured with high accuracy, e.g., at MAMI or MIT-Bates. These quantities encode information on the distribution of electric and magnetic charge inside the nucleon and also serve to determine the proton’s charge radius. The HERA experiments have significantly increased the kinematical range over which structure functions of the nucleon could be determined accurately. Polarized $ep$ and $\mu p/d$ scattering at HERMES, COMPASS, and JLab, provide the experimental basis for attempting to unravel the spin structure of the nucleon. Furthermore, a large experimental program is planned at future facilities (COMPASS-II, JLab at 12 GeV, PANDA@FAIR), designed to extract quantities such as GPDs, which provide rich information on the spatial distributions of quarks and gluons inside hadrons. This extensive experimental program requires equally intense theoretical activities, in order to gain a quantitative understanding of nucleon structure.

*a. Lattice-QCD calculations* Simulations of QCD on a space-time lattice are becoming increasingly important for the investigation of hadron structure. Form factors and structure functions of the nucleon have been the subject of lattice calculations for many years (see the recent reviews [201–204]), and more complex quantities such as GPDs have also been tackled recently [205–210], as reviewed in [211, 212]). Furthermore, several groups have reported lattice results on the strangeness content of the nucleon [213–222], as well as the strangeness contribution to the nucleon spin [223–229]. Although calculations of the latter quantities have not yet reached the same level of maturity concerning the overall accuracy compared to, say, electromagnetic form factors, they help to interpret experimental data from many experiments.

Lattice-QCD calculations of baryonic observables are technically more difficult than those of the corresponding quantities in the mesonic sector. This is largely due to the increased statistical noise which is intrinsic to baryonic correlation functions, and which scales as $\exp (m_\mathrm{N}-\frac{3}{2}m_\pi )$, where $m_\mathrm{N}$ and $m_\pi$ denote the nucleon and pion masses, respectively. As a consequence, statistically accurate lattice calculations are quite expensive. It is therefore more difficult to control the systematic effects related to lattice artifacts, finite-volume effects, and chiral extrapolations to the physical pion mass in these calculations. Statistical limitations may also be responsible for a systematic bias due to insufficient suppression of the contributions from higher excited states [230].

Many observables also require the evaluation of so-called “quark-disconnected” diagrams, which contain single quark propagators forming a loop. The evaluation of such diagrams in lattice QCD suffers from large statistical fluctuations, and specific methods must be employed to compute them with acceptable accuracy. In a lattice simulation, one typically considers isovector combinations of form factors and other quantities, for which the above-mentioned quark-disconnected diagrams cancel. It should be noted that hadronic matrix elements describing the ${\pi }N$ sigma term or the strangeness contribution to the nucleon are entirely based on quark-disconnected diagrams. With these complications in mind, it should not come as a surprise that lattice calculations of structural properties of baryons have often failed to reproduce some well-known experimental results.

In the following we summarize the current status of lattice investigations of structural properties of the nucleon. The Dirac and Pauli form factors, $F_1$ and $F_2$, are related to the hadronic matrix element of the electromagnetic current $V_{\mu }$ via

3.10 $$\begin{aligned}&\left\langle N(p^\prime ,s^\prime )| V_{\mu }(x) | N(p,s)\right\rangle \nonumber \\&\quad = \bar{u}(p^\prime ,s^\prime ) \left( \gamma _{\mu } F_1(Q^2) - \sigma _{\mu \nu }\frac{Q_\nu }{2m_\mathrm{N}}\, F_2(Q^2) \right) u(p,s),\nonumber \\ \end{aligned}$$

where $p,s$ and $p^\prime ,s^\prime$ denote the momenta and spins of the initial- and final-state nucleons, respectively, and $Q^2=-q^2$ is the negative squared momentum transfer. The Sachs electric and magnetic form factors, $G_\mathrm{E}$ and $G_\mathrm{M}$, which are related to the electron–proton scattering cross section via the Rosenbluth formula, are obtained from suitable linear combinations of $F_1$ and $F_2$, i.e.,

3.11 $$\begin{aligned}&G_\mathrm{E}(Q^2) = F_1(Q^2) + \frac{Q^2}{(2m_\mathrm{N})^2}F_2(Q^2),\nonumber \\&G_\mathrm{M}(Q^2)=F_1(Q^2)+F_2(Q^2). \end{aligned}$$

The charge radii associated with the form factors are then derived from

3.12 $$\begin{aligned} \left\langle r_i^2 \right\rangle = -6\left. \frac{d F_i(Q^2)}{d Q^2}\right| _{Q^2=0},\quad i=1,2 . \end{aligned}$$

Analogous relations hold for the electric and magnetic radii, $\langle {r_\mathrm{E}^2}\rangle$ and $\langle {r_\mathrm{M}^2}\rangle$.

Currently there is a large deviation between experimental determinations of $\langle r_\mathrm{E}^2 \rangle$ using muonic hydrogen and electronic systems that is called the “proton radius puzzle”, see Sect. 3.2.6 for further discussion.

**Fig. 6:**
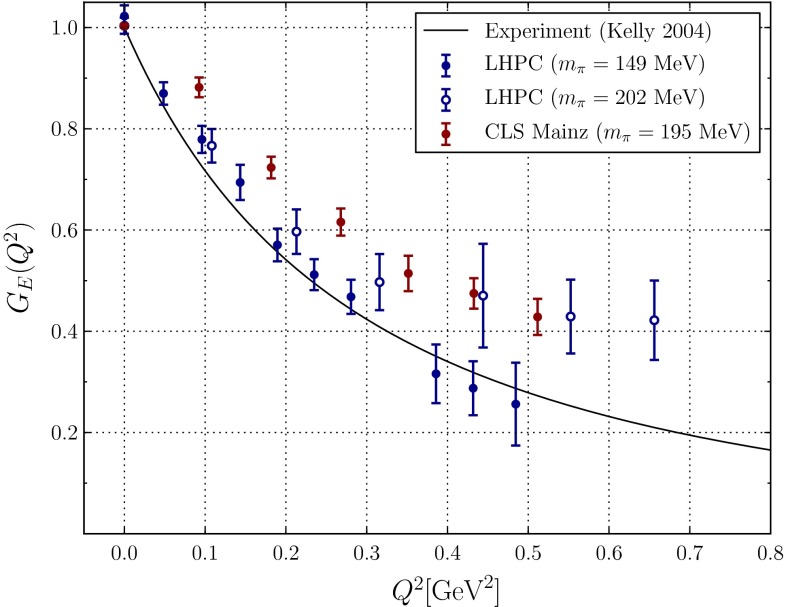
The dependence of the nucleon’s isovector electric form factor $G_\mathrm{E}$ on the Euclidean four-momentum transfer $Q^2=-q^2$ for near-physical pion masses, as reported by the LHP Collaboration [231] and the Mainz group [232]. The phenomenological parameterization of experimental data is from [233]

**Fig. 7:**
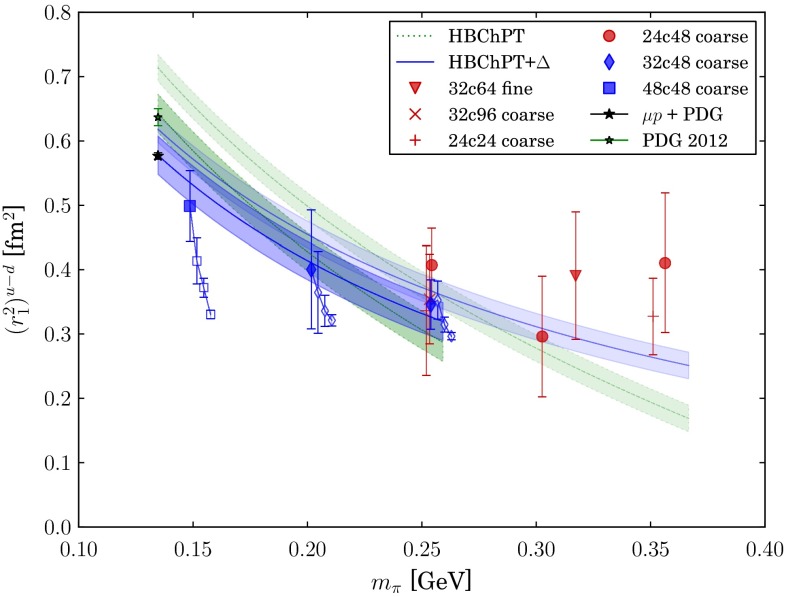
The dependence of the isovector Dirac radius $\langle r_1^2\rangle$ on the pion mass from [234]. *Filled blue symbols* denote results based on summed operator insertions, designed to suppress excited-state contamination

There are many cases in which lattice QCD calculations of observables that describe structural properties of the nucleon compare poorly to experiment. For instance, the dependence of nucleon form factors on $Q^2$ computed on the lattice is typically much flatter compared to phenomenological parameterizations of the experimental data, at least when the pion mass (i.e., the smallest mass in the pseudoscalar channel) is larger than about 250 MeV. It is then clear that the values of the associated charge radii are underestimated compared to experiment [206, 235–243]. The situation improved substantially after results from simulations with substantially smaller pion masses became available, combined with techniques designed to reduce or eliminate excited-state contamination. The data of [231] and [232] show a clear trend towards the $Q^2$-behavior seen in a fit of the experimental results as the pion mass is decreased from around 200 MeV to almost its physical value (see Fig. 6). Since different lattice actions are employed in the two calculations, the results are largely independent of the details of the fermionic discretization. A key ingredient in more recent calculations is the technique of summed operator insertions [244–247], for which excited state contributions are parametrically suppressed. Alternatively one can employ multi-exponential fits including the first excited state [231, 248] and solve the generalized eigenvalue problem for a matrix correlation function [249], or study the dependence of nucleon matrix elements for a wide range of source-sink separations [250]. Results for the pion mass dependence of the Dirac radius, $\langle r_1^2\rangle$, from [234] are shown in Fig. 7, demonstrating that good agreement with the PDG value [1] can be achieved. Similar observations also apply to the Pauli radius and the anomalous magnetic moment.

The axial charge of the nucleon, $g_A$, and the lowest moment of the isovector parton distribution function, $\langle x\rangle _{u-d}$ are both related to hadronic matrix elements with simple kinematics, since the initial and final nucleons are at rest. Furthermore, no quark-disconnected diagrams must be evaluated. If it can be demonstrated that lattice simulations accurately reproduce the experimental determinations of these quantities within the quoted statistical and systematic uncertainties, this would constitute a stringent test of lattice methods. In this sense $g_A$ and $\langle x\rangle _{u-d}$ may be considered benchmark observables for lattice QCD.

**Fig. 8:**
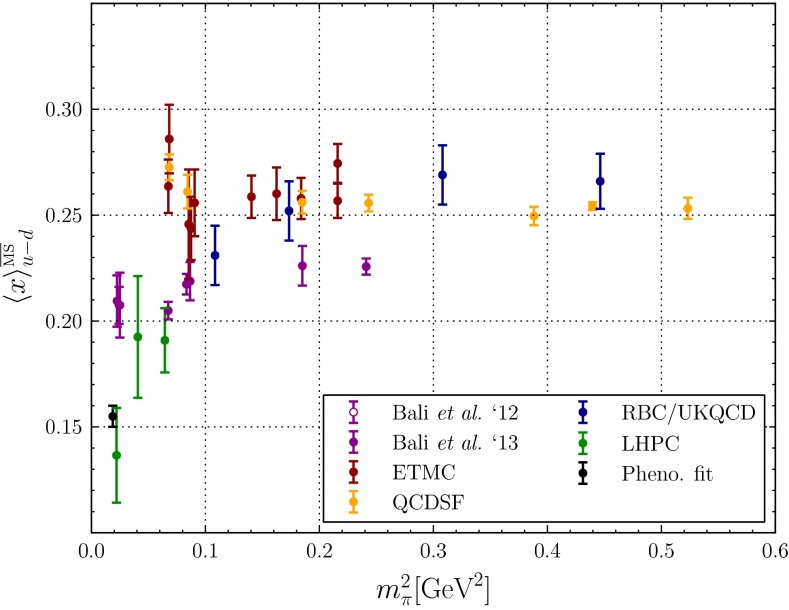
The dependence of the first moment of the isovector PDF plotted versus the pion mass. Lattice results are compiled from [207, 234, 240, 251–253]

Calculations based on relatively heavy pion masses have typically overestimated $\langle x\rangle _{u-d}$ [206–208, 239, 240, 251] by about 20 %. Moreover, it was found that $\langle x\rangle _{u-d}$ stays largely constant as a function of the pion mass (see Fig. 8). Lower values have been observed in [252, 253], but given that the overall pion mass dependence in that calculation is quite weak, it is still difficult to make contact with the phenomenological estimate. Other systematic errors, such as lattice artifacts or insufficient knowledge of renormalization factors, may well be relevant for this quantity. Recent calculations employing physical pion masses, as well as methods to suppress excited state contamination [234, 254], have reported a strong decrease of $\langle x\rangle _{u-d}$ near the physical value of $m_\pi$. Although the accuracy of the most recent estimates does not match the experimental precision, there are hints that lattice results for $\langle x\rangle _{u-d}$ can be reconciled with the phenomenological estimate.

**Fig. 9:**
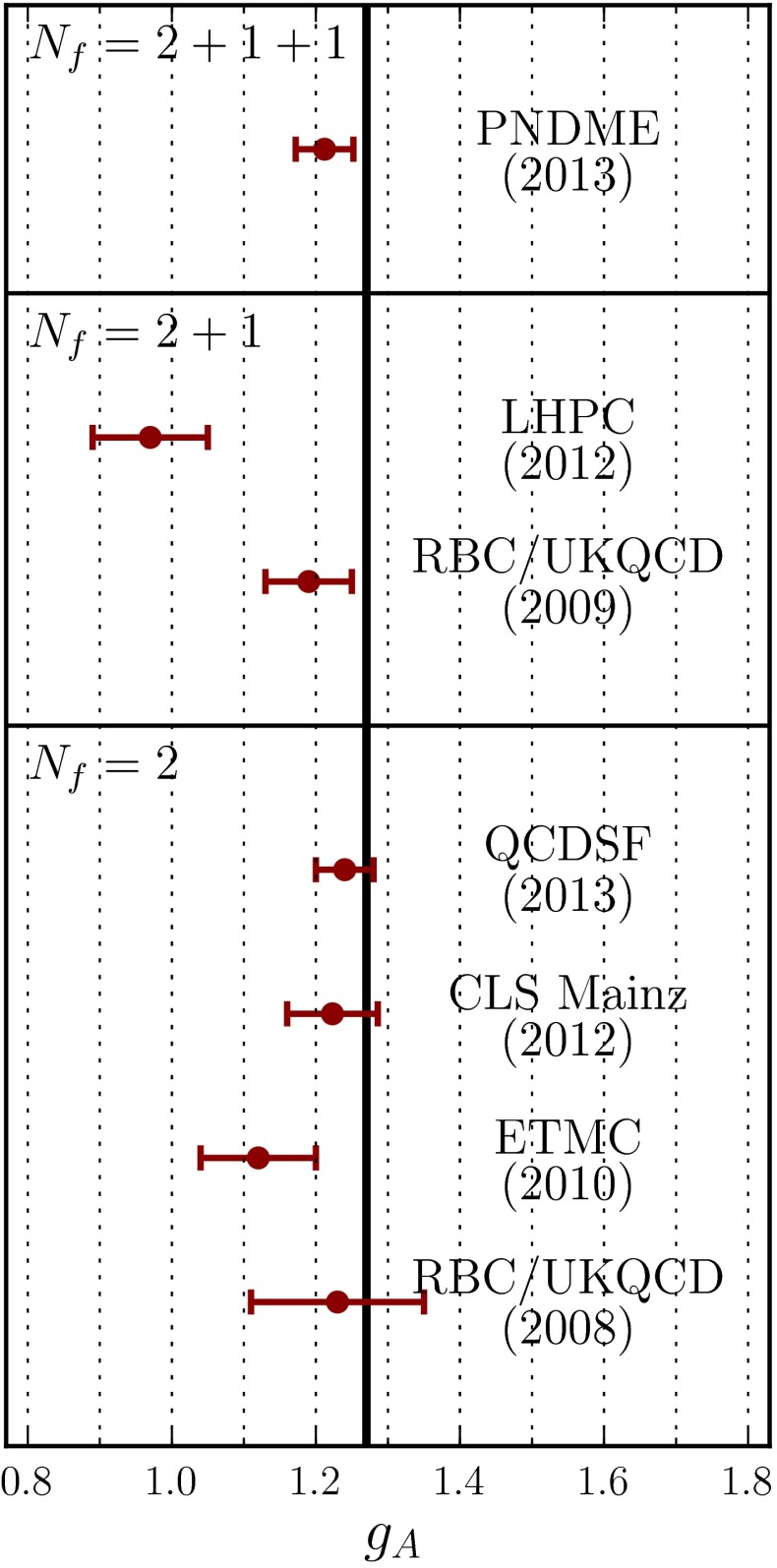
Compilation of recent published results for the axial charge in QCD with $N_\mathrm{f}=2+1+1$ dynamical quarks [248] (*upper panel*), $N_\mathrm{f}=2+1$ [234, 237] (*middle panel*), as well as two-flavor QCD [236, 247, 255, 256] (*lower panel*)

The strategy of controlling the bias from excited states and going towards the physical pion mass has also helped to make progress on $g_A$, which, compared to $\langle x\rangle _{u-d}$, is a simpler quantity. It is the matrix element of the axial current, i.e., a quark bilinear without derivatives, whose normalization factor is known with very good accuracy. Lattice simulations using pion masses $m_\pi > 250$ MeV typically underestimate $g_A$ by $10$–$15~\%$ [206, 236, 237, 239, 240, 242, 256–262]. Even more worrisome is the observation that the data from these simulations show little or no tendency to approach the physical value as the pion mass is decreased. However, although some of the most recent calculations using near-physical pion masses and addressing excited state contamination [247, 248, 255] produce estimates which agree with experiment (see Fig. 9), there are notable exceptions: the authors of [234] still find a very low result, despite using summed insertions which may be attributed to a particularly strong finite-size effect in $g_A$. The effects of finite volume have also been blamed for the low estimates reported in [263, 264].

The current status of lattice-QCD calculations of structural properties of the nucleon can be summarized by noting that various sources of systematic effects are now under much better control, which leads to a favorable comparison with experiment in many cases. Simulations employing near-physical pion masses and techniques designed to eliminate the bias from excited-state contributions have been crucial for this development. Further corroboration of these findings via additional simulations that are subject to different systematics is required. Also, the statistical accuracy in the baryonic sector must be improved.

*b. Poincaré-covariant Faddeev approach* The nucleons’ electromagnetic [265] as well as axial and pseudoscalar [266] form factors have been calculated in the Poincaré-covariant Faddeev framework based on Landau-gauge QCD Green’s functions. The latter are determined in a self-consistent manner from functional methods and, if available, compared to lattice results. Over the last decade, especially the results for corresponding propagators and some selected vertex functions have been established to an accuracy that they can serve as precise input to phenomenological calculations, see also the discussion in Sect. 8.2.

The main idea of the Poincaré-covariant Faddeev approach is to exploit the fact that baryons will appear as poles in the six-quark correlation function. Expanding around the pole one obtains (in a similar way as for the Bethe–Salpeter equation) a fully relativistic bound-state equation. The needed inputs for the latter equation are (i) the tensor structures of the bound-state amplitudes, which rest solely on Poincaré covariance and parity invariance and provide a partial-wave decomposition in the rest frame, see, e.g., [267, 268] and references therein for details; (ii) the fully dressed quark propagators for *complex* arguments; and (iii) the two- and three-particle irreducible interaction kernels. In case the three-particle kernel is neglected, the bound-state equation is then named the Poincaré-covariant Faddeev equation. The two-particle-irreducible interaction kernel is usually modeled within this approach, and mesons and baryons are then both considered in the so-called rainbow-ladder truncation, which is the simplest truncation that fully respects chiral symmetry and leads to a massless pion in the chiral limit.

In [265, 266] the general expression for the baryon’s electroweak currents in terms of three interacting dressed quarks has been derived. It turns out that in the rainbow-ladder truncation the only additional input needed is the fully dressed quark-photon vertex which is then also calculated in a consistent way. It is important to note that this vertex then contains the $\rho$-meson pole, a property which appears essential to obtaining the correct physics.

In the actual calculations a rainbow-ladder gluon-exchange kernel for the quark-quark interaction, which successfully reproduces properties of pseudoscalar and vector mesons, is employed. Then the nucleons’ Faddeev amplitudes and form factors are computed without any further truncations or model assumptions. Nevertheless, the resulting quark-quark interaction is flavor blind,^4^ and by assumption it is a vector-vector interaction and thus in contradiction to our current understanding of heavy-quark scalar confinement, cf. Sect. 8.2. References [269, 270] lays out an alternative description of the phenomenology of confinement, based on the interconnections of light-front QCD, holography, and conformal invariance, with wide-ranging implications for the description of hadron structure and dynamics.

**Fig. 10:**
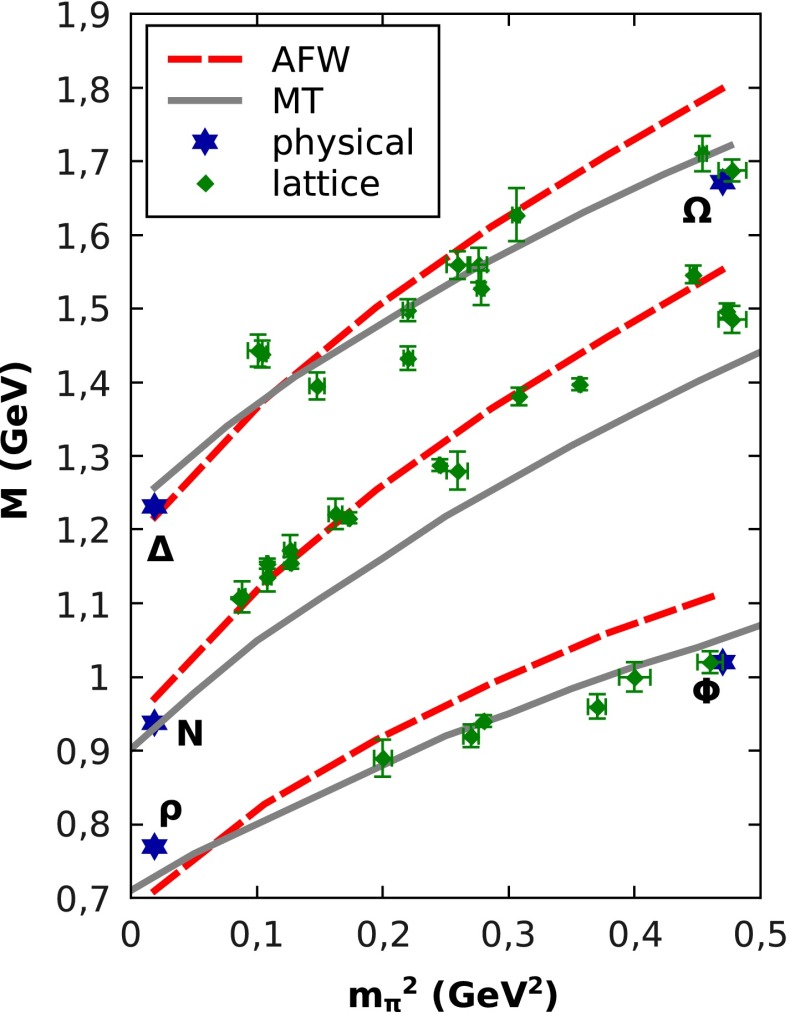
The vector meson, nucleon, and $\Delta$/$\Omega$ masses as a function of the pion mass squared in the Poincaré-covariant Faddeev approach (adapted from [278])

**Fig. 11:**
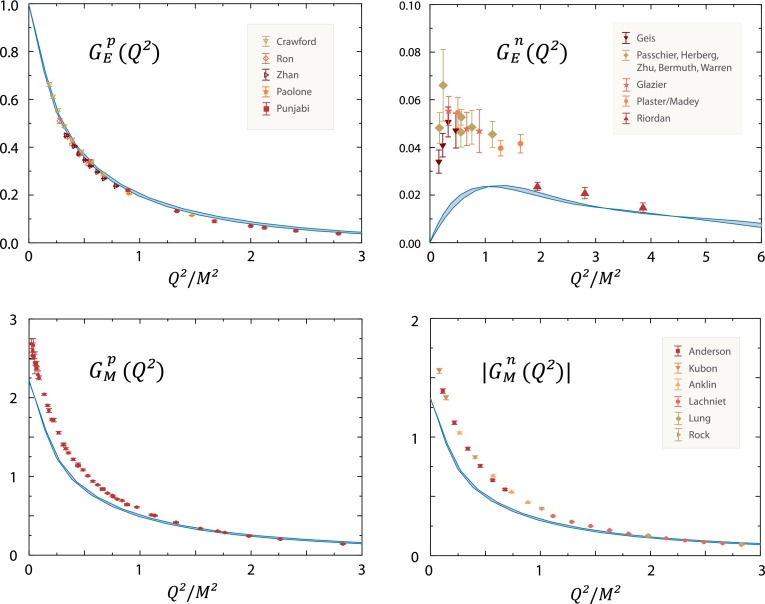
The nucleons’ electromagnetic form factors in the Poincaré-covariant Faddeev approach (adapted from [265])

Therefore the challenge posed to the Poincaré-covariant Faddeev approach is to extend in a systematically controlled way beyond the rainbow-ladder and the Faddeev truncations. Given the fact that non-perturbative calculations of the full quark–gluon vertex and three-gluon vertex have been published recently and are currently improved, this will become feasible in the near future. Nevertheless, already the available results provide valuable insight, and, as can be inferred from the results presented below, in many observables the effects beyond rainbow-ladder seem to be on the one hand surprisingly small and on the other hand in its physical nature clearly identifiable.

Figure 10 shows the results for some selected hadron masses using two different interaction models, see [271] for the MT and [272] for the AFW model. (The main phenomenological difference between these two models is that the AFW model reproduces the $\eta ^\prime$ mass via the Kogut–Susskind mechanism beyond rainbow-ladder whereas the (older) MT model does not take this issue into account.) As one can see, both model calculations compare favorably with lattice results [206, 235, 237, 238, 273–277]. Given the fact that the baryon masses are predictions (with parameters fixed from the meson sector) and that a rainbow-ladder model kernel has been used instead of a calculated one, the agreement is even somewhat better than expected.

**Fig. 12:**
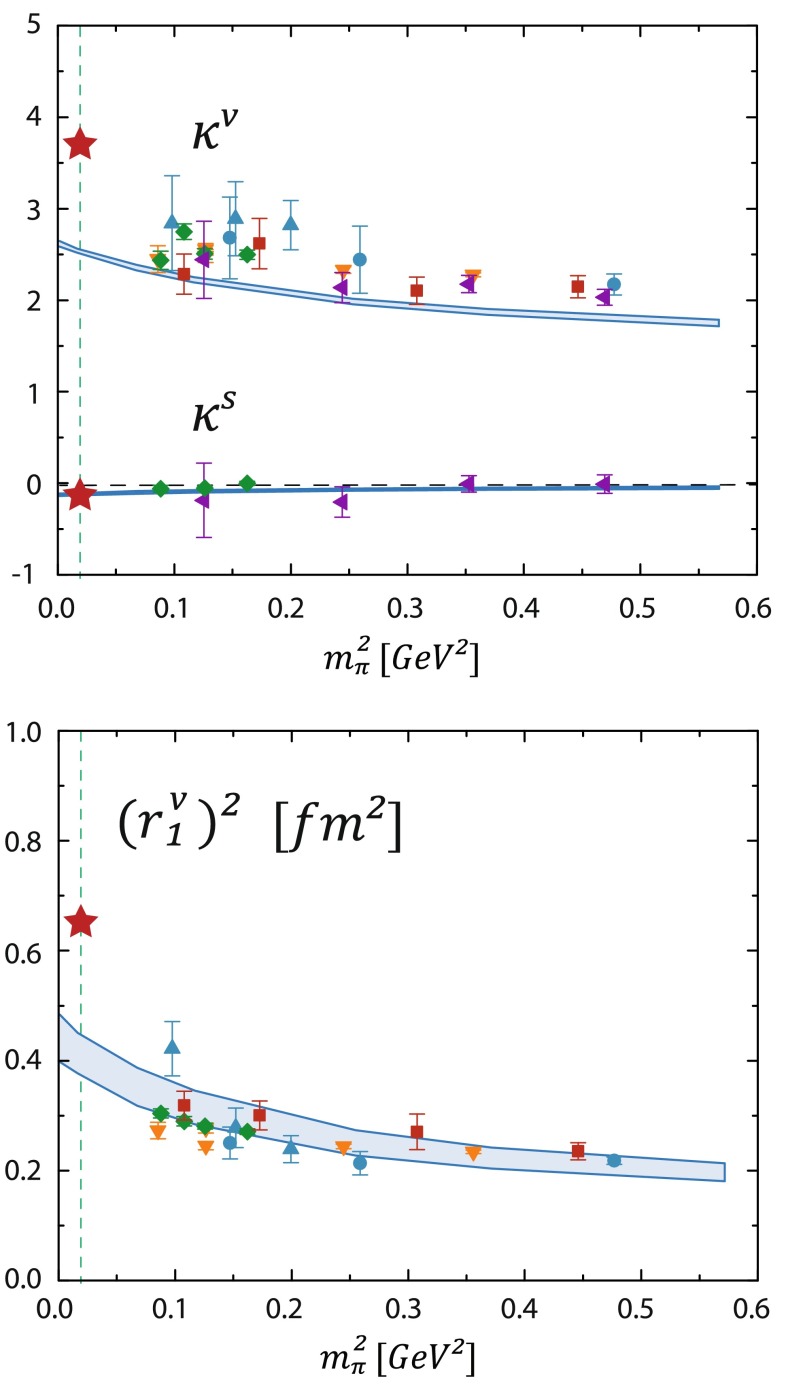
Results for the nucleon’s isoscalar and isovector anomalous magnetic moments and isovector Dirac radius in the Poincaré-covariant Faddeev approach as compared to lattice QCD results and experiment (*stars*) (adapted from [265])

In Fig. 11 the results for the electromagnetic form factors of the proton and neutron are shown. It is immediately visible that the agreement with the experimental data at large $Q^2$ is good. In addition, there is also good agreement with lattice data at large quark masses. These two observations lead to the expectation that the difference of the calculated results with respect to the observed data is due to missing pion-cloud contributions in the region of small explicit chiral symmetry breaking. This is corroborated by the observation that the pion-loop corrections of ChPT are compatible with the discrepancies appearing in Fig. 11. This can be deduced in a qualitative way from Fig. 12. The results of the Faddeev approach are, like the lattice results, only weakly dependent on the current quark mass (viz., the pion mass squared). Whereas lattice results are not (yet) available at small masses, the Faddeev calculation can be performed also in the chiral limit. However, pion loop (or pion cloud) effects are not (yet) contained in this type of calculations. Correspondingly there are deviations at the physical pion mass. To this end it is important to note that in the isoscalar combination of the anomalous magnetic moment leading-order pion effects are vanishing. As a matter of fact, the Faddeev approach gives the correct answer within the error margin of the calculation. Details can be found in [265].

**Fig. 13:**
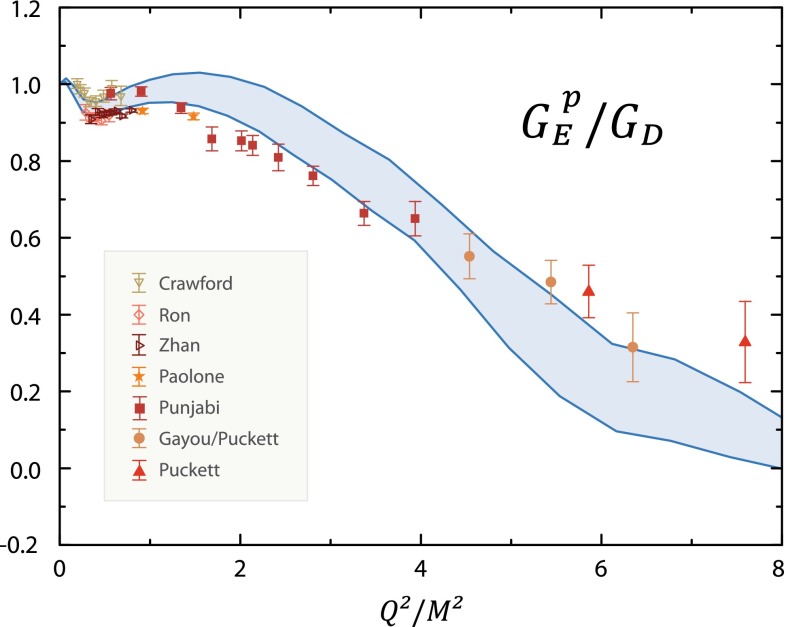
$Q^2$-evolution of the ratio of the proton’s electric form factor to a dipole form factor in the Poincaré-covariant Faddeev approach as compared to experimental data (adapted from [265])

Last but not least, the $Q^2$-evolution of the proton’s electric form factor in the multi-GeV region is a topic which has attracted a lot of interest in the last decade. Contrary to some expectations (raised by experimental data relying on the Rosenbluth separation) data from polarization experiments have shown a very strong decrease of the ratio of the proton’s electric to magnetic form factor. Even the possibility that the proton’s electric form factor possesses a zero at $Q^2 \approx 9$ GeV$^2$ is in agreement with the data. However, more details will be known only after the 12 GeV upgrade of JLab is fully operational. In this respect it is interesting to note that the quite complex Dirac–Lorentz structure of the proton’s Faddeev amplitude quite naturally leads to a strong decrease for $Q^2>2~$GeV$^2$ as shown in Fig. 13. Several authors attribute the difference between the data relying on Rosenbluth separation and polarized-target data to two-photon processes, see, e.g.,  [279]. This has initiated a study of two-photon processes in the Faddeev approach, and an extension to study Compton scattering has made first but important progress [280].

In [266] the axial and pseudoscalar form factors of the nucleon have been calculated in this approach. It is reassuring that the Goldberger–Treiman relation is fulfilled for the results of these calculations for all values of the current quark mass. On the other hand, the result for the axial charge is underestimated by approximately 20 %, yielding $g_A\approx 1$ in the chiral limit, which is again attributed to missing pion effects. This is corroborated by the finding that the axial and pseudoscalar form factors agree with phenomenological and lattice results in the range $Q^2>1\ldots 2$ GeV$^2$. In any case, the weak current-quark mass dependence of $g_A$ in the Faddeev approach deserves further investigation.

Decuplet, i.e., spin-3/2, baryons possess four electromagnetic form factors. These have been calculated in the Poincaré-covariant Faddeev approach for the $\Delta$ and the $\Omega$ [281], and the comments made above for the electric monopole and magnetic dipole form factors for the nucleon also apply here. The electric quadrupole (E2) form factor is in good agreement with the lattice QCD data and provides further evidence for the deformation of the electric charge contribution from sphericity. The magnetic octupole form factor measures the deviation from sphericity of the magnetic dipole distribution, and the Faddeev approach predicts nonvanishing but small values for this quantity.

Summarizing, the current status of results within the Poincaré-covariant Faddeev approach is quite promising. The main missing contributions beyond rainbow-ladder seem to be pionic effects, and it will be interesting to see whether future calculations employing only input from first-principle calculations will verify a picture of a quark core (whose rich structure is mostly determined by Poincaré and parity covariance) plus a pion cloud.

#### The proton radius puzzle

**Fig. 14:**
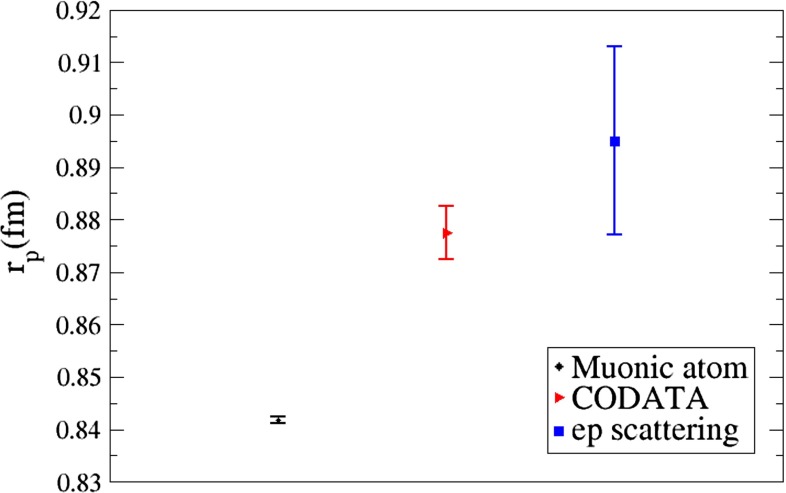
Proton radius determinations from (i) the muonic-hydrogen Lamb shift (*left*), (ii) electron–proton scattering (*right*), and (iii) the CODATA-2010 combination of the latter with ordinary hydrogen spectroscopy (*center*). Data taken from [290]

The so-called proton radius puzzle began as a disagreement at the 5$\sigma$ level between its extraction from a precise measurement of the Lamb shift in muonic hydrogen [282] and its CODATA value [283], compiled from proton-radius determinations from measurements of the Lamb shift in ordinary hydrogen and of electron–proton scattering. A recent refinement of the muonic hydrogen Lamb shift measurement has sharpened the discrepancy with respect to the CODATA-2010 [284] value to more than 7$\sigma$ [285]. The CODATA values are driven by the Lamb-shift measurements in ordinary hydrogen, and a snapshot of the situation is shown in Fig. 14, revealing that tensions exist between all the determinations at varying levels of significance.

The measured Lamb shift in muonic hydrogen is $202.3706 \pm 0.0023$ meV [285], and theory [286–289] yields a value of $206.0336 \pm 0.0015 - (5.2275 \pm 0.0010)r_\mathrm{E}^2 + \Delta E_\mathrm{TPE}$ in meV [290], where $r_\mathrm{E}$ is the proton charge radius and $\Delta E_\mathrm{TPE}$ reflects the possibility of two-photon exchange between the electron and proton. The first number is the prediction from QED theory and experiment. The proton-radius disagreement amounts to about a 300 $\upmu$eV change in the prediction of the Lamb shift. Considered broadly, the topic shows explicitly how a precise, low-energy experiment interplays with highly accurate theory (QED) to reveal potentially new phenomena. We now turn to a discussion of possible resolutions, noting the review of [291].

Since the QED calculations are believed to be well understood and indeed would have to be grossly wrong to explain the discrepancy [289] (and a recently suggested non-perturbative QED effect does not exist [292, 293]), a lot of attention has focused on the hadronic contribution arising from the proton’s structure, to which the muonic atom, given its smaller Bohr radius $a_0(\mu )\simeq (m_e/m_{\mu }) a_0 (e)$, is much more sensitive. If the disagreement is assigned to an error in the proton-radius determination, then, as we have noted, the disagreement between the muonic-atom determination [285] ($r_\mathrm{E}^{(\mu )}$) and the CODATA-2010 [284] value (based on hydrogen spectroscopy as well as elastic electron–proton scattering data) ($r_\mathrm{E}^{(e)}$) is very large, namely,

3.13 $$\begin{aligned} r_\mathrm{E}^{(\mu )}&= 0.84087 \pm 0.00039 \,\mathrm{fm} ,\nonumber \\ r_\mathrm{E}^{(e)}&= 0.8775 \pm 0.0051 \, \mathrm{fm}. \end{aligned}$$

It has been argued [294] that atomic physicists measure the rest-frame proton radius, but electron-scattering data, parametrized in terms of the Rosenbluth form factors, yields the Breit-frame proton radius, and these do not coincide. A resolution by definition might be convenient, but it is not true: precisely the same definition, namely, that of (3.12), is used in both contexts [288, 292]. The value of $r_\mathrm{E}^{(e)}$ from hydrogen spectroscopy does rely, though, on the value of the Rydberg constant $R_\infty$ [295], and new experiments plan to improve the determination of this important quantity [285].

The precision of the experimental extraction of the vector form factor from $ep$ scattering, from which the proton radius is extracted as per (3.12) [296], has also been questioned [297, 298]. In particular, it has been noted that the low-energy Coulomb correction from $ep$ final-state interactions is sizeable, and this ameliorates the discrepancy between the charge radii determined from hydrogen spectroscopy and its determination in $ep$ scattering [299].

Higher-order hadronic corrections involving two-photon processes have also been considered as a way of resolving the puzzle [300, 301]. Revised, precise dispersive reevaluations of the proton’s two-photon kernel [302] based on experimental input (photo- and electro-production of resonances off the nucleon and high-energy pomeron-dominated cross-section) yield a contribution of $40\pm 5\ \upmu$eV to the muonic hydrogen Lamb shift. The small uncertainty which remains is controlled with the “$J=0$” fixed pole of Compton scattering, i.e., the local coupling of two photons to the proton, and which is phenomenologically known only for real photons. This result is in tension with the value $\Delta E_\mathrm{TPE}=33.2 \pm 2.0\,\upmu \mathrm{eV}$ used in [285], but it remains an order of magnitude too small to explain the discrepancy in the Lamb shift. The appearance of different energy scales in the analysis of muonic hydrogen makes it a natural candidate for the application of effective field theory techniques [301, 303]. Limitations in the ability to assess the low-energy constants would seem to make such analyses inconclusive. Nevertheless, a systematic treatment under the combined use of heavy-baryon effective theory and (potential) non-relativistic QED [303, 304] has recently been employed to determine a proton radius of $0.8433\pm 0.0017\,\mathrm{fm}$ from the muonic hydrogen data [285], assuming that the underlying power counting determines the numerical size of the neglected terms. This result remains $6.4\sigma$ away from the CODATA-2010 result.

To summarize, hopes that hadronic contributions to the two-photon exchange between the muon and the proton would resolve the issue quickly are starting to fade away because the correction needed to explain the discrepancy is unnaturally large [305]. Therefore, it might be useful to test ideas of physics beyond the Standard Model, i.e., a different interaction of muons and electrons, in the context of the proton radius puzzle, see Sect. 5.6 for a corresponding discussion.

#### The pion and other pseudoscalar mesons

The lightest hadron, the pion, is one of the most important strongly interacting particles and serves as a “laboratory” to test various methods within QCD, both on the perturbative and the non-perturbative side. The electromagnetic form factor at spacelike momenta has been treated by many authors over the last decades using various techniques based on collinear factorization [306–308] with calculations up to the NLO order of perturbation theory, see, e.g., [309, 310]. A novel method was recently presented in [311] which uses the Dyson–Schwinger equation framework in QCD (see [312] for a review). This analysis shows the prevalence of the leading-twist perturbative QCD result (i.e., the hard contribution) for $Q^2F_{\pi }(Q^2)$ beyond $Q^2 \gtrsim 8$ GeV$^2$ in agreement with the earlier results of [310]. Furthermore, it reflects via the dressed quark propagator the scale of dynamical chiral symmetry breaking (D$\chi$SB) which is of paramount importance and still on the wish list of hadron physics, because a detailed microscopic understanding of this mechanism is still lacking. Moreover, our current understanding of the pion’s electromagnetic form factor in the timelike region is still marginal [313].

Nevertheless, the dual nature of the pion—being on the one hand the would-be Goldstone boson of D$\chi$SB and on the other hand a superposition of highly relativistic bound states of quark–antiquark pairs in quantum field theory—is basically understood and generally accepted. Furthermore, as discussed in Sect. 3.2.1, its valence parton distribution function has been recently determined with a higher precision using threshold resummation techniques [59]. Finally, the quark distribution amplitude for the pion, which universally describes its strong interactions in exclusive reactions, has been reconstructed from the world data on the pion–photon transition form factor as we will see below and is found to be wider than the asymptotic one [314].

*a. Form factors of pseudoscalar mesons* The two-photon processes $\gamma ^{*}(q_{1}^2)\gamma (q_{2}^{2}) \rightarrow P$ with $q_{1}^{2}=-Q^2$ and $q_{2}^{2}=-q^2\sim 0$ of pseudoscalar mesons $P=\pi ^0, \eta , \eta '$ in the high-$Q^2$ region have been studied extensively within QCD (see [312, 315, 316] for analysis and references). This theoretical interest stems from the fact that in leading order such processes are purely electromagnetic with all strong-interaction (binding) effects factorized out into the distribution amplitude of the pseudoscalar meson in question by virtue of collinear factorization. This implies that for $Q^2$ sufficiently large, the transition form factor for such a process can be formulated as the convolution of a hard-scattering amplitude $T(Q^2, q^2\rightarrow 0, x) = Q^{-2}(1/x + \mathcal {O}(\alpha _\mathrm{s}))$, describing the elementary process $\gamma ^*\gamma \longrightarrow q\bar{q}$, with the twist-two meson distribution amplitude [317]. Therefore, this process constitutes a valuable tool to test models of the distribution amplitudes of these mesons.

Several experimental collaborations have measured the cross section for $Q^{2}F^{\gamma ^*\gamma \pi ^0}(Q^2,q^2\rightarrow 0)$ and $Q^2F^{\gamma ^*\gamma \eta (\eta ')} (Q^2,q^2\rightarrow 0)$ in the two-photon processes $e^+e^{-} \rightarrow e^+e^{-} \gamma ^*\gamma \rightarrow e^+e^{-} X$, where $X=\pi ^{0}$ [318–320], $\eta$ and $\eta '$ [319, 321], through the so-called single-tag mode in which one of the final electrons is detected. From the measurement of the cross section the meson–photon transition form factor is extracted as a function of $Q^2$. The spacelike $Q^2$ range probed varies from $0.7$–$2.2$ GeV$^2$ [318] (CELLO), to $1.5$–$9.0$ GeV$^2$ [319] (CLEO), to $4$–$40$ GeV$^2$ [320] (BaBar) and [322] (Belle). A statistical analysis and classification of all available experimental data versus various theoretical approaches can be found in [314]. The BaBar Collaboration extended substantially the range of the spacelike $Q^2$, which had been studied before by CELLO [318] and CLEO [319] below 9 GeV$^2$ to $Q^2 < 40$ GeV$^2$. While at low momentum transfers the results of BaBar agree with those of CLEO and have significantly higher accuracy, above 9 GeV$^2$ the form factor shows rapid growth and from $\sim 10$ GeV$^2$ it exceeds the asymptotic limit predicted by perturbative QCD [306]. The most recent results reported by Belle [322] for the wide kinematical region $4 \lesssim Q^2 \lesssim 40$ GeV$^2$ have provided important evidence in favor of the collinear factorization scheme of QCD. The rise of the measured form factor $Q^{2}F^{\gamma ^*\gamma \pi ^0}$, observed earlier by the BaBar Collaboration [320] in the high-$Q^2$ region, has not been confirmed. This continued rise of the form factor would indicate that the asymptotic value of the form factor predicted by QCD would be approached from above and at much higher $Q^2$ than currently accessible, casting serious doubts on the validity of the QCD factorization approach and fueling intensive theoretical investigations in order to explain it (see, for example, [323]). The results of the Belle measurement are closer to the standard theoretical expectations [306] and do not hint to a flat-like pion distribution amplitude as proposed in [323]. Further support for this comes from the data reported by the BaBar Collaboration [321] for the $\eta (\eta ')$-photon transition form factor that also complies with the QCD theoretical expectations of form-factor scaling at higher $Q^2$. A new experiment by KLOE-2 at Frascati will provide information on the $\pi$–$\gamma$ transition form factor in the low-$Q^2$ domain, while the BES-III experiment at Beijing will measure this form factor below $5$ GeV$^2$ with high statistics.

*b. Neutral pion lifetime* In the low-energy regime, the two-photon process $\pi ^0\rightarrow \gamma \gamma$ is also important because one can test at once the Goldstone boson nature of the $\pi ^0$ and the chiral Adler–Bell–Jackiw anomaly [324, 325]. While the level of accuracy achieved long ago makes existing tests satisfactory, deviations due to the nonvanishing quark masses should become observable at some point. The key quark-mass effect is due to the isospin-breaking-induced mixing: $\pi ^0$–$\eta$ and $\pi ^0$–$\eta '$, with the mixing being driven by $m_d-m_u$. The full ChPT correction has been evaluated by several authors and an enhancement of the decay width of about $4.5\pm 1.0$ % has been found [326–328], leading to the prediction $\Gamma _{\pi ^0\rightarrow 2 \gamma }= 8.10$ eV.

The most recent measurement was carried out by the PRIMEX collaboration at JLab with an experiment based on the Primakoff effect [329], providing the result $\Gamma (\pi ^0\rightarrow \gamma \gamma )=7.82$ eV with a global uncertainty of 2.8 %, which is by far the most precise result to date. Taking into account the uncertainties, it is marginally compatible with the ChPT predictions. With the aim of reducing the error down to 2 %, a second PRIMEX experiment has been completed and results of the analysis should appear soon. A measurement of $\Gamma _{\pi ^0\rightarrow 2\gamma }$ at the per cent level is also planned in the study of two-photon collisions with the KLOE-2 detector [330]. A recent review of the subject can be found in [331].

**Fig. 15:**
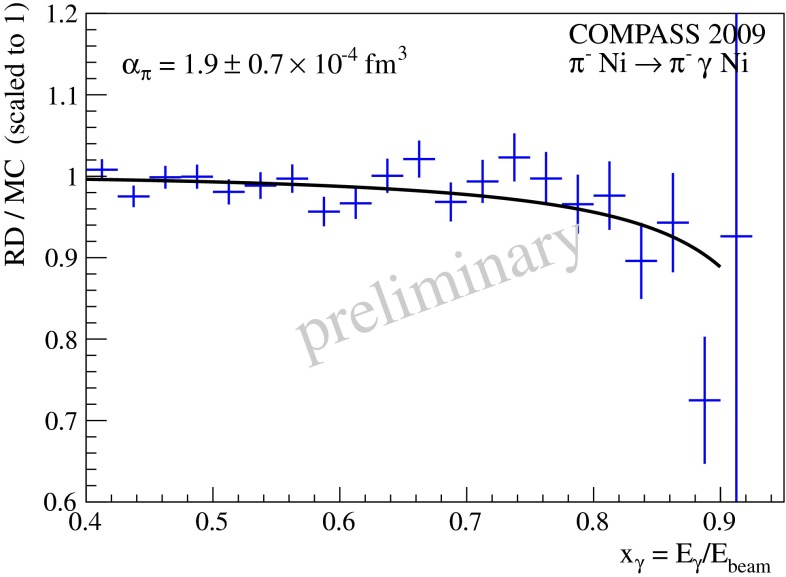
Determination of the pion polarizability at COMPASS through the process $\pi ^{-} \mathrm {Ni}\rightarrow \pi ^{-}\gamma \mathrm {Ni}$ [337]

*c. Pion polarizabilities* Further fundamental low-energy properties of the pion are its electric and magnetic polarizabilities $\alpha _\pi$ and $\beta _\pi$. While firm theoretical predictions exist based on ChPT [332, 333], the experimental determination of these quantities from pion–photon interactions using the Primakoff effect [334], radiative pion photoproduction [335], and $\gamma \gamma \rightarrow \pi \pi$ [336], resulted in largely scattered and inconsistent results. The COMPASS experiment at CERN has performed a first measurement of the pion polarizability in pion-Compton scattering with $190\,~{\mathrm {GeV}}/c$ pions off a Ni target via the Primakoff effect. The preliminary result, extracted from a fit to the ratio of measured cross section and the one expected for a point-like boson shown in Fig. 15, is $\alpha _\pi =(1.9 \pm 0.7_\mathrm {stat}\pm 0.8_\mathrm {sys})\cdot 10^{-4}\,\mathrm {fm}^3$, where the relation between electric and magnetic polarizability $\alpha _\pi =-\beta _\pi$ has been assumed [337]. This result is in tension with previous experimental results, but is in good agreement with the expectation from ChPT [332]. New data taken with the COMPASS spectrometer in 2012 are expected to decrease the statistical and systematic error, determined at COMPASS by a control measurement with muons in the same kinematic region, by a factor of about three. The data will for the first time allow an independent determination of $\alpha _\pi$ and $\beta _\pi$, as well as a first glimpse on the polarizability of the kaon. Studies of the charged pion polarizability have been proposed and approved at JLab, where the photon beam delivered to Hall D will be used for the Primakoff production of $\pi ^+\pi ^{-}$ of a nuclear target. A similar study of the $\pi ^0$ polarizability will also be possible.

### Hadron spectroscopy

In contrast to physical systems bound by electromagnetic interactions, the masses of light hadrons are not dominated by the masses of their elementary building blocks but are to a very large extent generated dynamically by the strong force. The coupling of the light quarks to the Higgs field is only responsible for ${\sim } 1~\%$ of the visible mass of our present-day universe, the rest is a consequence of the interactions between quarks and gluons. While at high energies the interactions between partons become asymptotically free, allowing systematic calculations in QCD using perturbation theory, the average energies and momenta of partons inside hadrons are below the scale where perturbative methods are justified. As a consequence, the fundamental degrees of freedom of the underlying theory of QCD do not directly manifest themselves in the physical spectrum of hadrons, which, rather, are complex, colorless, many-body systems. One of the main goals of the physics of strong interactions for many years has been the determination and the understanding of the excitation spectrum of these strongly bound states. In the past, phenomenological models have been developed, which quite successfully describe certain aspects of the properties of hadrons in terms of effective degrees of freedom, e.g., the quark model [338, 339], the bag model [340, 341], the flux-tube model [342], or QCD sum rules [343]. A full understanding of the hadron spectrum from the underlying theory of QCD, however, is still missing. Nowadays, QCD solved numerically on a discrete spacetime lattice [344] is one of the most promising routes towards this goal.

On the experimental side, significant advances in the light-quark sector have been made in the last few years. Data with unprecedented statistical accuracy have become available from experiments at both electron and hadron machines, often coupled with new observables related to polarization or precise determination of the initial and final-state properties. In the light-meson sector, the unambiguous identification and systematic study of bound states beyond the constituent quark degrees of freedom, e.g., multiquark states or states with gluonic degrees of freedom (hybrids, glueballs), allowed by QCD due to its non-Abelian structure, is within reach of present and future generations of experiments. For a recent review, see e.g. [345]. For the light baryons, photoproduction experiments shed new light on the long-standing puzzle of missing resonances. Here, the recent progress is summarized in [346].

On the theoretical side, hadron spectroscopy has received a huge boost from lattice QCD. Simulations with dynamical up, down, and strange quarks are now routinely performed, and in many cases the need for chiral extrapolations is becoming obsolete thanks to the ability to simulate at or near the physical values of the up and down quark masses [347, 348]. This concerns, in particular, lattice calculations of the masses of the lightest mesons and baryons [349–351], which show excellent agreement with experiment. Lattice-QCD calculations for the masses of higher-lying mesons, baryons, as well as possible glueball and hybrid states can provide guidance for experiments to establish a complete understanding of the hadron spectrum. Other theoretical tools, such as dispersion relations, provide a way to extract physically relevant quantities such as pole positions and residues of amplitudes.

#### Lattice QCD

The long-sought objective of studying hadron resonances with lattice QCD is finally becoming a reality. The discrete energy spectrum of hadrons can be determined by computing correlation functions between creation and annihilation of an interpolating operator $\mathcal {O}$ at Euclidean times $0$ and $t$, respectively,

3.14 $$\begin{aligned} C(t) = \left\langle 0 \right| \mathcal {O}(t)\mathcal {O}^\dagger (0)\left| 0 \right\rangle \!. \end{aligned}$$

Inserting a complete set of eigenfunctions $\left| n \right\rangle$ of the Hamiltonian $\hat{H}$ which satisfy $\hat{H}\left| n \right\rangle =E_k\left| n \right\rangle$, the correlation function can be written as a sum of contributions from all states in the spectrum with the same quantum numbers,

3.15 $$\begin{aligned} C(t) = \sum _n{\left| \left\langle 0 \right| \mathcal {O}\left| n \right\rangle \right| ^2 e^{-E_n t}}\!. \end{aligned}$$

For large times, the ground state dominates, while the excited states are subleading contributions. To measure the energies of excited states, it is thus important to construct operators which have a large overlap with a given state. The technique of smearing the quark-field creation operators is well established to improve operator overlap [245, 352–354]. A breakthrough for the study of excited states was the introduction of the distillation technique [355], where the smearing function is replaced by a cost-effective low-rank approximation. The interpolating operators are usually constructed from a sum of basis operators $\mathcal {O}_i$ for a given channel,

3.16 $$\begin{aligned} \mathcal {O}=\sum _i{v_i}\mathcal {O}_i, \end{aligned}$$

and a variational method [356] is then employed to extract the best linear combination of operators within a finite basis for each state which maximizes $C(t)/C(t_0)$. This requires the determination of all elements of the correlation matrix

3.17 $$\begin{aligned} C_{ij}(t) = \left\langle 0 \right| \mathcal {O}_i(t)\mathcal {O}_j^\dagger (0)\left| 0 \right\rangle \!, \end{aligned}$$

and the solution of the generalized eigenvalue problem [357, 358]

3.18 $$\begin{aligned} C(t)v_n = \lambda _n C (t_0)v_n. \end{aligned}$$

The procedure requires a good basis set of operators that resembles the states of interest.

**Fig. 16:**
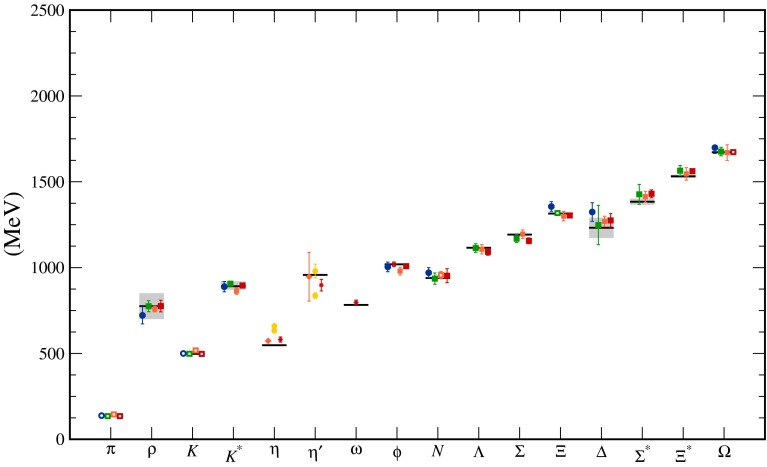
Hadron spectrum from lattice QCD. Wide-ranging results are from MILC [361, 362], PACS-CS [349], BMW [350], and QCDSF [363]. Results for $\eta$ and $\eta '$ are from RBC & UKQCD [364], Hadron Spectrum [365] (also the only $\omega$ mass), and UKQCD [366]. *Symbol shape* denotes the formulation used for sea quarks. *Asterisks* represent anisotropic lattices. *Open symbols* denote the masses used to fix parameters. *Filled symbols* (and *asterisks*) denote results. *Red*, *orange*, *yellow*, *green*, and *blue* stand for increasing numbers of ensembles (i.e., lattice spacing and sea quark mass). *Horizontal bars* (*gray boxes*) denote experimentally measured masses (*widths*). Adapted from [360]

Thanks to algorithmic and computational advances in recent years, lattice-QCD calculations of the lowest-lying mesons and baryons with given quantum numbers and quark content have been performed with full control of the systematics due to lattice artifacts (see the review in [359]). Figure 16 shows a 2012 compilation of lattice-QCD calculations of the light-hadron spectrum [360]. The pion and kaon masses have been used to fix the masses of light and strange quarks, and (in each case) another observable is used to set the overall mass scale. The experimentally observed spectrum of the baryon octet and decuplet states, as well as the masses of some light vector mesons, are well reproduced within a few percent of accuracy. Except for the isosinglet mesons, the calculations shown use several lattice spacings and a wide range of pion masses. They also all incorporate $2+1$ flavors into the sea, but the chosen discretization of the QCD action differs. The consistency across all calculations suggests that the systematics, which are different for different calculations, are well controlled. This body of work is a major achievement for lattice QCD, and the precision will improve while the methods are applied to more challenging problems.

Also for simulations of excited mesons and baryons huge progress has been made, although the control of the systematics is still much less advanced than in the case of the ground states [276, 365, 367–378]. These calculations are typically performed for relatively few fairly coarse lattice spacings, and no continuum extrapolation is attempted. A systematic study of finite-volume effects, as well as the extrapolation to physical quark masses, have not yet been performed. The goal of these calculations is to establish a general excitation pattern rather than to perform precision calculations. The focus at the moment is therefore on identifying a good operator basis, on disentangling various excitations in a given channel, and on separating resonances from multihadron states.

*a. Light mesons* As an example of recent progress, the work of the Hadron Spectrum Collaboration [365, 368, 371] is highlighted here, which recently performed a fully dynamical (unquenched) lattice-QCD calculation of the complete light-quark spectrum of mesons and baryons. The simulations are carried out on anisotropic lattices with lattice spacings $a_\mathrm {s}\sim 0.12\,\mathrm {fm}$ and $a_t^{-1}\sim 5.6\,\mathrm {GeV}$ in the spatial and temporal directions, respectively, and with spatial volumes of $L^3 \sim (2.0\,\mathrm {fm})^3$ and $(2.5\,\mathrm {fm})^3$. They are performed with three flavors of order-$a$ improved Wilson quarks, i.e., a mass-degenerate light-quark doublet, corresponding to a pion mass down to $396\,~\mathrm {MeV}$, and a heavier quark whose mass is tuned to that of the strange quark. A large basis of smeared operators for single mesons was built using fermion bilinears projected onto zero meson momentum, including up to three gauge-covariant derivatives. No operators corresponding to multiparticle states, however, were used. The distillation method was used to optimize coupling to low-lying excited states. The correlators are analyzed by a variational method, which gives the best estimate for masses and overlaps. The spins of states are determined by projection of angular momentum eigenstates onto the irreducible representations of the hypercubic group.

**Fig. 17:**
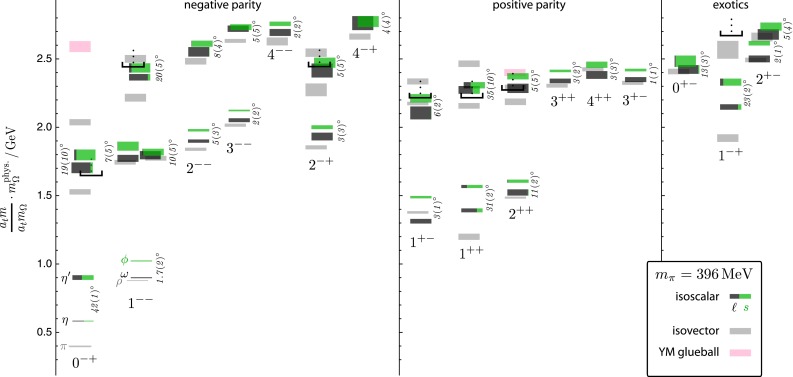
Light-quark meson spectrum resulting from lattice QCD [365], sorted by the quantum numbers $J^{\mathrm{PC}}$. Note that these results have been obtained with an unphysical pion mass, $m_\pi =396\,~\mathrm {MeV}$

The resulting isoscalar and isovector meson spectrum is shown in Fig. 17 [365]. Quantum numbers and the quark–gluon structure of a meson state $n$ with a given mass $m_{n}$ are extracted by studying matrix elements $\langle n|\mathcal {O}_i|0\rangle$, which encode the extent to which operator $\mathcal {O}_i$ overlaps with state $n$. States with high spins, up to $4$, are resolved. The resulting spectrum, as well as the strange–nonstrange mixing of isoscalar mesons, compares well with the currently known states [1]. The calculated masses come out about $15~\%$ too high, probably owing to the unphysical pion mass, $m_\pi =396\,~\mathrm {MeV}$. The lattice-QCD simulations also predict a number of extra states, that are not yet well established experimentally. These include a series of exotic states with quantum numbers which cannot be produced by pairing a quark and an antiquark, like $J^{\mathrm{PC}}=0^{+-},1^{-+},2^{+-},\ldots$, which have been previously postulated to exist also in various models. For some states, a significant overlap with operators containing the gluon field strength tensor has been found, making them candidates for hybrids. It is interesting to note that the quantum numbers and the degeneracy pattern predicted by lattice QCD for hybrid mesons are quite different from those of most models. Lattice QCD predicts four low-mass hybrid multiplets at masses around $2\,~{\mathrm {GeV}}$ with quantum numbers $1^{-+},0^{-+},1^{-\,\!-},2^{-+}$, in agreement with the bag model [379, 380], but at variance with the flux-tube model [342, 381], which predicts eight nearly degenerate hybrid multiplets. At masses larger than $2.4\,~{\mathrm {GeV}}$, lattice QCD predicts a group of ten hybrid multiplets, in disagreement with bag and flux-tube model predictions. The pattern emerging from lattice QCD, i.e., of four low-mass and ten higher-mass multiplets, can be reproduced by a $q\overline{q}'$ pair in an $S$- or $P$-wave coupled to a $1^{+-}$ chromomagnetic gluonic excitation, which can be modeled by a quasi-gluon in a $P$-wave with respect to the $q\overline{q}'$ pair [382].

The spectrum of glueballs has first been calculated on a lattice in pure SU(3) Yang–Mills theory, i.e. in the quenched approximation to QCD [383–385] at a lattice spacing of $a\sim 0.1$–0.2 fm. A full spectrum of states is predicted with the lightest one having scalar quantum numbers, $0^{++}$, and a mass between $1.5\,~{\mathrm {GeV}}$ and $1.7\,~{\mathrm {GeV}}$. Also the next-higher glueball states have non-exotic quantum numbers, $2^{+\,\!+}$ (mass $2.3$–$2.4\,~{\mathrm {GeV}}$) and $0^{-+}$ (mass $2.3$–$2.6\,~{\mathrm {GeV}}$), and hence will be difficult to identify experimentally. In a simple constituent gluon picture, these three states correspond to two-gluon systems in relative $S$ wave, with different combinations of helicities. Table 1 summarizes the quenched lattice results for the masses of the lightest glueballs.

**Table 1:** Continuum-limit glueball masses (in MeV) from quenched lattice QCD. The first parentheses contain the statistical errors, while the second, where present, include the scale uncertainty

$J^{PC}$	Bali [383]	Morningstar [384]	Chen [385]
$0^{+\,\!+}$	$1,550(50)$	$1,730(50)(80)$	$1,710(50)(80)$
$2^{+\,\!+}$	$2,270(100)$	$2,400(25)(120)$	$2,390(30)(120)$
$0^{-\,\!+}$	$2,330(270)$	$2,590(40)(130)$	$2,560(35)(120)$

**Fig. 18:**
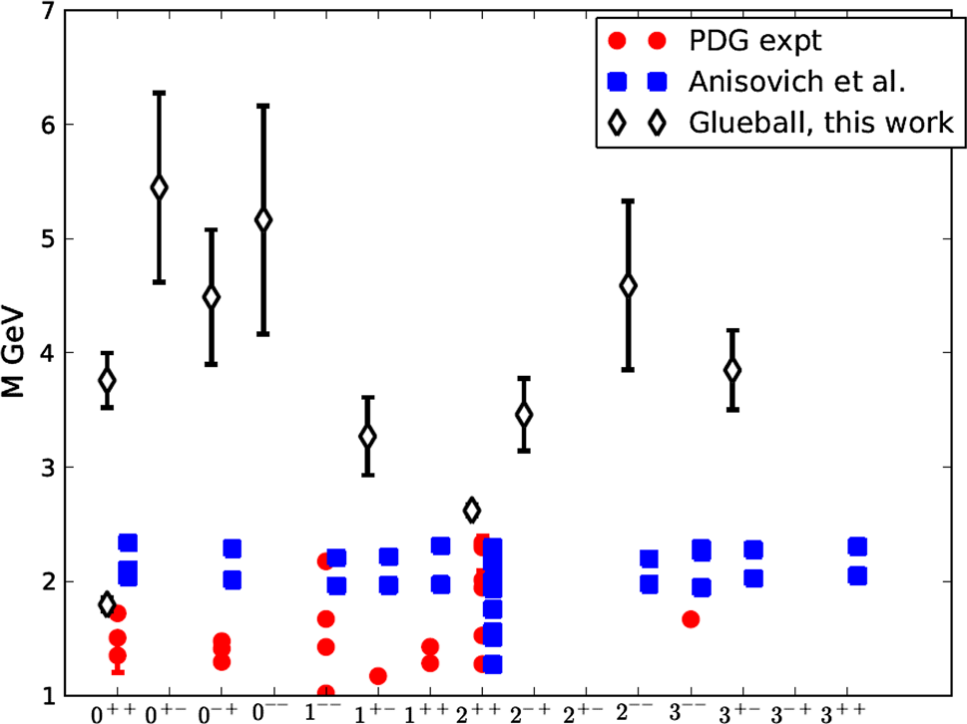
Glueball masses resulting from unquenched lattice QCD [388], compared with experimental meson masses [1, 389]. From [388]

While the glueball spectrum in pure SU(3) Yang–Mills theory is theoretically well defined, because the glueball operators do not mix with fermionic operators, unquenched lattice calculations are more difficult. The dynamical sea quarks will cause the glueball and flavor singlet fermionic $0^{+\,\!+}$ interpolating operators to couple to the same physical states. In addition, decays of the $0^{+\,\!+}$ states into two mesons are allowed for sufficiently light quark masses, and may thus play an important role and dynamically modify the properties of the glueball state. Hence, lattice-QCD calculations of the glueball spectrum with dynamical $q\overline{q}$ contributions are still at a relatively early stage [386–388]. One particular problem is the unfavorable signal-to-noise ratio of the relevant correlation functions, which requires large statistics. The authors of [388], using 2+1 flavors of ASQTAD improved staggered fermions and a variational technique which includes glueball scattering states, found no evidence for large effects from including dynamical sea quarks. Their mass for the $0^{+\,\!+}$ glueball, $1795(60)\,~\mathrm {MeV}$, is only slightly higher compared to the quenched result of [385]. Figure 18 shows the glueball masses calculated in [388], compared to some experimental meson masses. No extrapolation to the continuum, however, was performed, and no fermionic scattering states were included. Much higher statistics will be needed for precise unquenched calculations of flavor singlet sector on the lattice, with a $200\,~\mathrm {MeV}$ resolution needed, e.g., to distinguish the three isoscalar mesons in the $1.5\,~{\mathrm {GeV}}$ mass range. A technique designed to overcome the problem of an exponentially increasing noise-to-signal ratio in glueball calculations has been proposed and tested in the quenched approximation [390]. However, it is not known whether it can be generalized to full QCD.

*b. Light baryons* In addition to the meson spectrum, also the spectrum of baryons containing the light quarks $u, d$, and $s$ has been calculated recently by different groups [276, 367, 369–372, 374–377, 391]. While the focus lies mostly on establishing the spectral pattern of baryon resonances, the possibility of the existence of hybrid baryons has also been addressed. For instance, the Hadron Spectrum Collaboration has obtained spectra for $N$ and $\Delta$ baryons with $J\le \frac{7}{2}$ and masses up to ${\sim } 1.9M_\Omega$ [371]. The well-known pattern of organizing the states in multiplets of $\mathrm{SU}(6)\times O(3)$, where the first is the spin-flavor group, clearly emerges when checking the overlap of the states with the different source/sink operators. The multiplicity of states observed is similar to that of the non-relativistic quark model. The first excited positive-parity state, however, is found to have significantly higher mass than its negative spin-parity partner, in contrast to the experimental ordering of the $N(1440)\frac{1}{2}^+$ and the $N(1535)\frac{1}{2}^{-}$. The chiral behavior of the observed level structure in these calculations has been analyzed in detail in [276, 370, 375, 377]. Furthermore, no obvious pattern of degenerate levels with opposite parity (parity doubling) emerges from the simulations for higher masses, in contrast to indications from experiments [392]. Lattice-QCD calculations have been extended to include excited hyperons [372, 376, 377]. In particular, the nature of the $\Lambda (1405)$ has been the subject of the study in [372]. Moreover, lattice QCD presents the possibility of testing for the presence of excited glue in baryons (hybrids) for the first time, and it has been carried out in [374]. In contrast to the meson sector, however, all possible $J^P$ values for baryons can be built up from states consisting of three quarks with non-vanishing orbital angular momentum between them, so that there is no spin-exotic signature of hybrid baryons. It is found that their multiplet structure is compatible with a color-octet chromomagnetic excitation with quantum numbers $J^P=1^{+-}$, coupling to three quarks in a color-octet state and forming a color-neutral object, as in the case of hybrid mesons. Also the mass splitting between the $qqq$ states and the hybrid states is the same as observed for the meson sector, indicating a common bound-state structure for hybrid mesons and baryons.

*c. Future directions* These very exciting developments still lack two aspects: First, there is the issue of controlling systematic effects such as lattice artifacts, finite-volume effects, and long chiral extrapolations owing to the use of unphysical quark masses. Second, the fact that hadron resonances have a non-zero width is largely ignored in the calculations described above, i.e., resonances are treated as stable particles. While the first issue will be dealt with once gauge ensembles with finer lattice spacing and smaller pion masses are used for spectrum calculations, the second problem requires a different conceptual approach. The position and width of a resonance are usually determined from the scattering amplitude. However, as noted in [393], the latter cannot be determined directly from correlation functions computed in Euclidean space-time. Lüscher pointed out in his seminal work [394–397] that the phase shift of the scattering amplitude in the elastic region can be determined from the discrete spectrum of multi-particle states in a finite volume. When plotted as a function of ${m_\pi }L$, resonances can be identified via the typical avoided level crossing.

The Lüscher formalism, which was originally derived for the center-of-mass frame of mass-degenerate hadrons, has since been generalized to different kinematical situations [398–404]. Numerical applications of the method are computationally quite demanding, since they require precise calculations for a wide range of spatial volumes, as well as the inclusion of multi-hadron interpolating operators. Most studies have therefore focused on the simplest case, i.e., the $\rho$-meson [405–411]. As reviewed in [412], other mesonic channels such as $K\pi$, $D\pi$, and $D^{*}\pi$, as well as the $N\pi$ system (i.e., the $\Delta$-resonance) have also been considered. While the feasibility of extracting scattering phase shifts via the Lüscher method has been demonstrated, lattice calculations of resonance properties are still at an early stage. In spite of the technical challenges involved in its implementation, the Lüscher method has been extended to the phenomenologically more interesting cases of multi-channel scattering [413–415] and three-particle intermediate states [416, 417].

#### Continuum methods

Although lattice calculations will provide answers to many questions in strong QCD, the development of reliable analytical continuum methods is a necessity to develop an intuitive understanding of QCD from first principles, to construct advanced phenomenological models, and to address computationally challenging tasks like the extrapolation to physical quark masses or large hadronic systems. The tools at our disposal include effective theories such as ChPT, Dyson–Schwinger methods, fixed gauge Hamiltonian QCD approaches, and QCD sum rule methods.

A study of baryon resonances with various models has been carried out since time immemorial. In recent times, dynamical models based on meson-baryon degrees of freedom have received much attention [418–421], in particular in the case of $S$ wave resonances, such as the $\Lambda (1405)$. These models use effective Lagrangians to couple light mesons to the ground-state baryons, and in this way generate resonances dynamically. Since baryons couple strongly to the continuum, it is known that meson-baryon dynamics plays an important role; one would like to eventually understand how to better quantify that role by using improved models. One can speculate that this can also be an interesting topic of exploration in the framework of lattice QCD, where the possibility of varying the quark masses can illuminate how excited baryon properties change with the pion mass. Models of baryons based on the Schwinger–Dyson equations have also been studied [422, 423], and are being developed into important tools to study excited baryons with a framework anchored in the principles of QCD.

In the spirit of effective theories, one approach based on the $1/N_\mathrm{c}$ expansion has been developed [424–427]. In the limit of large $N_\mathrm{c}$, a spin-flavor dynamical symmetry emerges in the baryon sector, which is broken at subleading order in $1/N_\mathrm{c}$ and thus provides a starting point for the description of baryon observables in a power series in $1/N_\mathrm{c}$. As in every effective theory, it is necessary to give inputs, namely baryon observables determined phenomenologically, and the $1/N_\mathrm{c}$ expansion serves to organize and relate them at each order in the expansion. The framework is presented as an expansion in composite operators, where quantities or observables are expanded on a basis of operators at a given order in $1/N_\mathrm{c}$, and the coefficients of the expansion, which encode the QCD dynamics, are determined by fitting to the observables. It has been applied to baryon masses [428–434], partial decay widths [435–437], and photocouplings [438]. Through those analyses it is observed that the different effects, which are classified by their $\mathrm{SU}(2 N_\mathrm{f})\times O(3)$ structure ($N_\mathrm{f}$ is the number of light flavors) and by their power in $1/N_\mathrm{c}$, seem to follow the natural order of the $1/N_\mathrm{c}$ expansion, that is, they have natural magnitude. An interesting challenge is the implementation of the $1/N_\mathrm{c}$ expansion constraints in models, in order to have a more detailed understanding of the dynamics. One such nice and illustrative example has been given in [439].

#### Experiments

The fundamental difficulty in studying the light-hadron spectrum is that in most cases resonances do not appear as isolated, narrow peaks. Instead, states have rather large widths of several hundred $~\mathrm {MeV}$ and consequently overlap. Peaks observed in a spectrum may be related to thresholds opening up or interference effects rather than to genuine resonances, not to speak of kinematic reflections or experimental acceptance effects. In addition, nonresonant contributions and final-state effects may also affect the measured cross section. Partial wave or amplitude analysis (PWA) techniques are the state-of-the-art way to disentangle contributions from individual, and even small, resonances and to determine their quantum numbers. Multiparticle decays are usually modeled using the phenomenological approach of the isobar model, which describes multiparticle final states by sequential two-body decays into intermediate resonances (isobars), that eventually decay into the final state observed in the experiment. Event-based fits allow one to take into account the full correlation between final-state particles. Coupled-channel analyses are needed to reliably extract resonance parameters from different reactions or final states.

One notoriously difficult problem is the parameterization of the dynamical properties of resonances. Very often, masses and widths of resonances are determined from Breit–Wigner parameterizations, although this approach is strictly only valid for isolated, narrow states with a single decay channel. For two-body processes, e.g., the K-matrix formalism provides a way to ensure that the amplitudes fulfill the unitarity condition also in the case of overlapping resonances. The rigorous definition of a resonance is by means of a pole in the second (unphysical) Riemann sheet of the complex energy plane. For poles deep in the complex plane, however, none of the above approaches yield reliable results, although they might describe the data well. The correct analytical properties of the amplitude are essential for an extrapolation from the experimental data (real axis) into the complex plane in order to determine the pole positions. Dispersion relations provide a rigorous way to do this by relating the amplitude at any point in the complex plane to an integral over the (imaginary part of the) amplitude evaluated on the real axis (i.e., the data) making use of Cauchy’s theorem.

*a. Scalar mesons and glueballs* The identification and classification of scalar mesons with masses below $2.5\,~{\mathrm {GeV}}$ is a long-standing puzzle. Some of them have large decay widths and couple strongly to the two-pseudoscalar continuum. The opening of nearby thresholds such as $K\overline{K}$ and $\eta \eta$ strongly distort the resonance shapes. In addition, non-$q\overline{q}'$ scalar objects like glueballs and multi-quark states are expected in the mass range below $2\,~{\mathrm {GeV}}$, which will mix with the states composed of $q\overline{q}'$. The Particle Data Group (PDG) currently lists the following light scalars [1], sorted according to their isospin: ($I=0$) $f_0(500)$, $f_0(980)$, $f_0(1370)$, $f_0(1500)$, $f_0(1710)$, ($I=1/2$) $K_0^*(800)$ (listed as still requiring confirmation), $K_0^*(1430)$, ($I=1$) $a_0(980)$, $a_0(1450)$. One possible interpretation is that the scalars with masses below $1\,~{\mathrm {GeV}}$ form a new nonet with an inverted mass hierarchy, with the wide, isoscalar $f_0(500)$ as the lightest member, the $K_0^*(800)$ (neutral and charged), and the isospin-triplet $a_0(980)$, which does not have any $s$-quark content in the quark model, and its isospin-singlet counterpart $f_0(980)$ as the heaviest members. The high masses of the $a_0(980)$ and the $f_0(980)$ and their large coupling to $K\bar{K}$ could be explained by interpreting them as tightly bound tetraquark states [440] or $K\bar{K}$ molecule-like objects [441]. The scalar mesons above $1\,~{\mathrm {GeV}}$ would form another nonet, with one supernumerary isoscalar state, indicating the presence of a glueball in the $1.5\,~{\mathrm {GeV}}$ mass region mixing with the $q\bar{q}$ states [442]. Other interpretations favor an ordinary $q\bar{q}$ nonet consisting of $f_0(980)$, $a_0(980)$, $K_0^*(1430)$, and $f_0(1500)$ [443, 444]. The $f_0(1370)$ is interpreted as an interference effect. The $K_0^*(800)$ is not required in this model, and the supernumerary broad $f_0(500)$ would then have a large admixture of a light glueball. In view of these different interpretations it is important to clarify the properties of scalar mesons. An updated review on the topic can be found, e.g., in the PDG’s “Note on Scalar Mesons below $2\,~{\mathrm {GeV}}$” [1].

When it comes to the lightest scalar mesons, the $f_0(500)$, huge progress has been made in recent years towards a confirmation of its resonant nature and the determination of its pole position. Although omitted from the PDG’s compilation for many years, its existence has been verified in several phenomenological analyses of $\pi$–$\pi$ scattering data. As for other scalar particles, the $f_0(500)$, also known as $\sigma$, is produced in, e.g., $\pi$–$N$-scattering or $\bar{p}p$-annihilation, and data is, in particular, obtained from $\pi$–$\pi$, $K$–$\bar{K}$, $\eta$–$\eta$, and 4$\pi$ systems in the $S$-wave channel. The analyses of several processes require four poles, the $f_0(500)$ and three other scalars, in the region from the $\pi$–$\pi$ threshold to $1600\,~\mathrm {MeV}$. Hereby the missing distinct resonance structure below $900\,~\mathrm {MeV}$ in $\bar{p} p$-annihilation was somehow controversial. However, by now it is accepted that also these data are described well with the standard solution requiring the existence of the broad $f_0(500)$.

The pole position, i.e., the pole mass and related width, is also accurately determined. The combined analysis with ChPT and dispersion theory of $$\pi $$–$$\pi $$ scattering [445] has led to a particularly accurate determination of those parameters. The PDG quotes a pole position of $$M-i\Gamma /2\simeq \sqrt{s_\sigma } = (400$$–$$550)-i(200$$–$$350)\,~\mathrm {MeV}$$, whereas averaging over the most advanced dispersive analyses gives a much more restricted value of $$\sqrt{s_\sigma } = (446\pm 6)-i(276\pm 5)\,~\mathrm {MeV}$$. Especially relevant for the precise determination of the $$f_0(500)$$ pole were recent data from the NA48/2 experiment at CERN’s Super Proton Synchrotron (SPS) on $$K^\pm \rightarrow \pi ^+\pi ^{-} e^\pm \nu $$ (K$$_{e4}$$) decays [446], which have a much smaller systematic uncertainty than the older data from $$\pi N\rightarrow \pi \pi N$$ scattering due to the absence of other hadrons in the final state. NA48/2 has collected $$1.13$$ million K$$_{e4}$$ events using simultaneous $$K^+$$ and $$K^{-}$$ beams with a momentum of $$60\,~{\mathrm {GeV}}$$.

As mentioned above, however, there exist many, partly mutually excluding interpretations of the $f_0(500)$: a quark–antiquark bound state, $\pi$–$\pi$ molecule, tetraquark, QCD dilaton, to name the most prominent ones. In addition, it will certainly also mix with the lightest glueball. From the phenomenological side it is evident that the large $\pi$–$\pi$ decay width is the largest obstacle in gaining more accurate information. However, it is exactly the pattern of D$\chi$SB which makes this width quite naturally so large. An $f_0(500)$ with a small width could only occur if there is substantial explicit breaking of chiral symmetry (because, e.g., a large current mass would lead to $m_\sigma < 2 m_\pi$) or if by some other mechanism the scalar mass would be reduced.

Here a look to the electroweak sector of the Standard Model is quite enlightening. The scalar particle claimed last year by CMS and ATLAS with mass $125$–$126\,~{\mathrm {GeV}}$ is consistent with the Standard Model (SM) Higgs boson, cf. Sect. 5.2.3. It appears to be very narrow as its width-to-mass ratio is small. Though the mass is a free parameter of the SM, one natural explanation of its lightness relative to its “natural” mass of about $300$–$400\,~{\mathrm {GeV}}$ is fermion-loop mass renormalization, strongest by the top quark loop. This is one clear contribution that makes the Higgs light.^5^ In any case, the accident $m_\mathrm{H} < 2 m_W$ prevents the decay $h\rightarrow WW$. Since the longitudinal $W$ components are the Goldstone bosons of electroweak symmetry breaking, the analogy to $\sigma \pi \pi$ in QCD is evident. If the top quark were much lighter, or if it would be less strongly coupled (such as the nucleon to the sigma), the Higgs mass could naturally be higher by some hundreds of GeV, the decay channel to $WW$ would open, and the Higgs would have a width comparable in magnitude to its mass. This comparison makes it plain that the $f_0(500)$, for which no fermion that strongly couples to it is similar in mass, is naturally so broad because of the existence of pions as light would-be Goldstone bosons and its strong coupling to the two-pion channel. Unfortunately, this also implies that the nature of the $f_0(500)$ can be only revealed by yet unknown non-perturbative methods. It has to be emphasized that the lack of understanding of the ground state in the scalar meson channel is an unresolved but important question of hadron physics.

In recent dispersive analyses [447, 448] of $\pi \pi$ scattering data and the very recent $K_{\ell 4}$ experimental results [446], the pole positions of the $f_0(500)$ and $f_0(980)$ were determined simultaneously, and the results, summarized in Table 2, are in excellent agreement with each other.

**Table 2:** Positions of the complex poles of the $f_0(500)$ and $f_0(980)$, determined in dispersive analyses [447, 448] of $\pi \pi$ scattering data and $K_{\ell 4}$ decays

Ref.	$\sqrt{s_0}$ ($~\mathrm {MeV}$)	
	$f_0(500)$	$f_0(980)$
[447]	$\left( 457^{+14}_{-13}\right) -i\left( 279^{+11}_{-7}\right)$	$\left( 996\pm 7\right) -i\left( 25^{+10}_{-6}\right)$
[448]	$\left( 442^{+5}_{-8}\right) -i\left( 274^{+6}_{-5}\right)$	$\left( 996^{+4}_{-14}\right) -i\left( 24^{+11}_{-3}\right)$

The situation with the lightest strange scalar, $K_0^*(800)$ or $\kappa$, is more complicated. A dispersive analysis of $\pi K\rightarrow \pi K$ scattering data gives a pole position of the $K_0^*(800)$ of $\left( 658\pm 13\right) -i/2\left( 557 \pm 24\right) \,~\mathrm {MeV}$ [449], while recent measurements by BESII in $J/\psi \rightarrow K_\mathrm{S} K_\mathrm{S} \pi ^+\pi ^{-}$ decays [450] give a slightly higher value for the pole position of $\left( 764\pm 63^{+71}_{-54}\right) -i\left( 306\pm 149^{+143}_{-85}\right) \,~\mathrm {MeV}$. Similar results from dispersive analyses are expected for the $a_0(980)$. A broad scalar with mass close to that above is also needed for the interpretation of the $K\pi$ invariant mass spectrum observed by Belle in $\tau ^{-} \rightarrow K^0_\mathrm{S}\pi ^{-}\nu _\tau$ decay [451]. Numerous measurements of invariant mass spectra in hadronic decays of $D$ and $B$ mesons are hardly conclusive because of the large number of interfering resonances involved in parameterizations and different models used in the analyses.

**Fig. 19:**
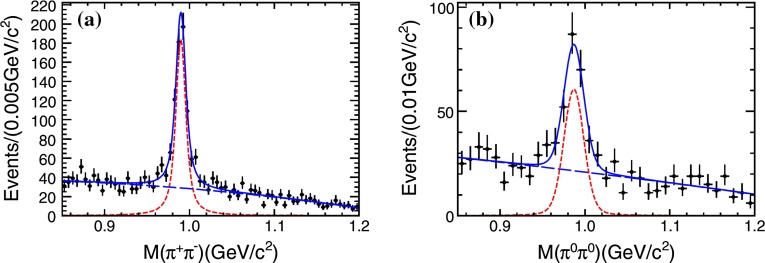
Invariant mass of $\pi ^+\pi ^{-}$ and $\pi ^0\pi ^0$ with the $\pi ^+\pi ^{-}\pi ^0$ ($3\pi ^0$) mass in the $\eta (1405)$ mass region, measured at BES III [453]

New data are being collected by BES III at the recently upgraded BEPCII $e^+e^{-}$ collider in Beijing in the $\tau$-charm mass region at a luminosity of $10^{33}\,\mathrm {cm}^{-2}\,\mathrm {s}^{-1}$ (at a center-of-mass (CM) energy of $2\times 1.89\,~{\mathrm {GeV}}$), with a maximum CM energy of $4.6\,~{\mathrm {GeV}}$ [452]. In the last 3 years, the experiment has collected the world’s largest data samples of $J/\psi$, $\psi (2S)$, and $\psi (3770)$ decays. These data are also being used to make a variety of studies in light-hadron spectroscopy, especially in the scalar meson sector. Recently, BES III reported the first observation of the isospin-violating decay $\eta (1405) \rightarrow \pi ^0 f_0(980)$ in $J/\psi \rightarrow \gamma 3\pi$ [453], together with an anomalous lineshape of the $f_0(980)$ in the $2\pi$ invariant mass spectra, as shown in Fig. 19.

The $f_0$ mass, deduced from a Breit–Wigner fit to the mass spectra, is slightly shifted compared to its nominal value, with a width of $<11.8\,~\mathrm {MeV}$ ($90~\%$ C.L.), much smaller than its nominal value. The observed isospin violation is $(17.9\pm 4.2)~\%$, too large to be explained by $f_0(980)$–$a_0(980)$ mixing, also observed recently by BES III at the $3.4\sigma$ level [454]. Wu et al. [455] suggest that a $K$ triangle anomaly could be large enough to account for the data.

**Fig. 20:**
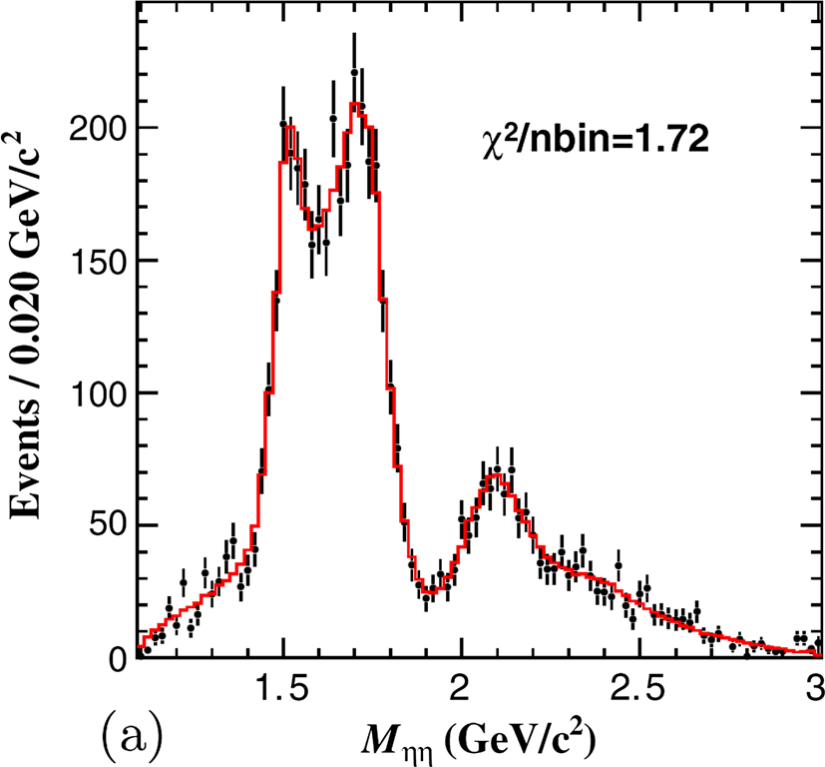
Invariant mass distribution of $\eta \eta$ from $J/\psi \rightarrow \gamma \eta \eta$, and the projection of the PWA fit from BES III [456]

BES III has recently performed a full PWA of $5460$ radiative $J/\psi$ decays to two pseudoscalar mesons, $J/\psi \rightarrow \gamma \eta \eta$, commonly regarded as an ideal system to look for scalar and tensor glueballs. In its baseline solution, the fit contains six scalar and tensor resonances [456], $f_0(1500)$, $f_0(1710)$, $f_0(2100)$, $f_2'(1525)$, $f_2(1810)$, and $f_2(2340)$, as well as $0^{+\,\!+}$ phase space and $J/\psi \rightarrow \phi \eta$. The scalars $f_0(1710)$, $f_0(2100)$, and $f_0(1500)$ are found to be the dominant contributions, with the production rate for the latter being about one order of magnitude smaller than for the first two. No evident contributions from $f_0(1370)$ or other scalar mesons are seen. The well-known tensor resonance $f_2'(1525)$ is clearly observed, but several $2^{+\,\!+}$ tensor components are also needed in the mass range between $1.8$ and $2.5\,~{\mathrm {GeV}}$. The statistical precision of the data, however, is not yet sufficient to distinguish the contributions. Figure 20 shows the resulting PWA fit result of the $\eta \eta$ invariant mass spectrum.

In conclusion, the situation in the scalar meson sector is still unresolved. The lightest glueball is predicted to have scalar quantum numbers and is therefore expected to mix with nearby isoscalar scalar $q\bar{q}$ $P$-wave states. For recent reviews on glueballs, see [444, 457, 458]. On the experimental side, further new results from BES III, from Belle on two-photon production of meson pairs [459, 460], and from COMPASS on central production [461] may help to resolve some of the questions in the scalar sector in the future.

*b. Hybrid mesons* Experimental evidence for the existence of hybrid mesons can come from two sources. The observation of an overpopulation of states with $q\overline{q}'$ quantum numbers may indicate the existence of states beyond the quark model, i.e., hybrids, glueballs, or multi-quark states. The densely populated spectrum of light mesons in the mass region between $1$ and $2\,~{\mathrm {GeV}}/c^2$, and the broad nature of the states involved, however, makes this approach difficult. It requires the unambiguous identification of all quark-model states of a given $J^{\mathrm{PC}}$ nonet, a task which has been achieved only for the ground-state nonets so far. The identification of a resonant state with exotic, i.e., non-$q\overline{q}'$ states, however, is considered a “smoking gun” for the existence of such states. Table 3 lists experimental candidates for hybrid mesons and their main properties.^6^

Models, as well as lattice QCD, consistently predict a light hybrid multiplet with spin-exotic quantum numbers $J^{\mathrm{PC}}=1^{-+}$. Currently, there are three experimental candidates for a light $1^{-+}$ hybrid [1] (for recent reviews, see [487, 488]): the $\pi _1(1400)$ and the $\pi _1(1600)$, observed in diffractive reactions and $\overline{p}N$ annihilation, and the $\pi _1(2015)$, seen only in diffraction. The $\pi _1(1400)$ has only been observed in the $\pi \eta$ final state, and is generally considered too light to be a hybrid meson. In addition, a hybrid should not decay into a $P$-wave $\eta \pi$ system from SU(3) symmetry arguments [489]. There are a number of studies that suggest it is a nonresonant effect, possibly related to cusp effects due to two-meson thresholds. The $\pi _1(1600)$ has been seen decaying into $\rho \pi$, $\eta '\pi$, $f_1(1285)\pi$, and $b_1(1235)\pi$. New data on the $1^{-+}$ wave have recently been provided by COMPASS, CLEO-c, and CLAS and will be reviewed in the following.

**Fig. 21:**
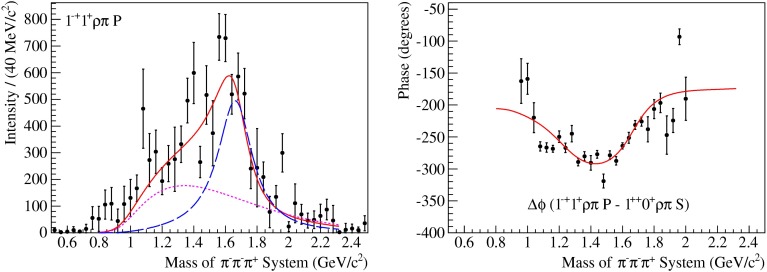
Exotic $1^{-+}1^+\,\rho \pi \,P$ wave observed at the COMPASS experiment [467] for 4-momentum transfer between $0.1$ and $1.0\,~{\mathrm {GeV}}^2$ on a Pb target and $\pi ^{-}\pi ^{-}\pi ^+$ final state. *Left* intensity, *right* phase difference from the $1^{+\,\!+}0^+\,\rho \pi \,S$ wave as a function of the $3\pi$ invariant mass. The *data points* represent the result of the fit in mass bins, the *lines* are the result of the mass-dependent fit

The COMPASS experiment [490] at CERN’s Super Proton Synchrotron (SPS) is investigating diffractive and Coulomb production reactions of hadronic beam particles into final states containing charged and neutral particles. In a first analysis of the $\pi ^{-}\pi ^{-}\pi ^+$ final state from scattering of $190\,~{\mathrm {GeV}}$ $\pi ^{-}$ on a Pb target, a clear signal in intensity and phase motion in the $1^{-+}1^+\,\rho \pi \,P$ partial wave has been observed [467], as shown in Fig. 21.

**Table 3:** Experimental properties of low-mass hybrid candidate states with quantum numbers $J^{PC}=1^{-\,\!+}$, $0^{-\,\!+}$, $2^{-\,\!+}$, $1^{-\,\!-}$

State	$J^{PC}$	Final state	Decay mode(s)	Mass $(~\mathrm {MeV})$	Width $(~\mathrm {MeV})$	Events	Reference
$\pi _1(1400)$	$1^{-\,\!+}$	$\pi ^{+}\pi ^{-}\pi ^{0} \pi ^{0}$	$\eta \pi ^0$	$1257\pm 20\pm 25$	$354\pm 64\pm 60$	$24$k	E852 [462]
		$2\pi ^{+} 2\pi ^{-}$	$\rho \pi$	$1384\pm 20\pm 35$	$378\pm 58$	$90$k	OBELIX [463]
		$\pi ^{0} \pi ^{0}\eta (2\gamma )$	$\eta \pi ^0$	$1360\pm 25$	$220\pm 90$	$270$k	CB [464]
		$\pi ^{-}\pi ^0\eta (2\gamma )$	$\eta \pi$	$1400\pm 20\pm 20$	$310\pm 50^{+50}_{-30}$	$53$k	CB [465]
		$\pi ^{-}\eta (2\gamma )$	$\eta \pi ^{-}$	$1370\pm 16^{+50}_{-30}$	$385\pm 40^{+65}_{-105}$	$47$k	E852 [466]
$\pi _1(1600)$	$1^{-\,\!+}$	$\pi ^{+}\pi ^{-}\pi ^{-}$	$\rho \pi ^{-}$	$1660\pm 10^{+0}_{-64}$	$269\pm 21^{+42}_{-64}$	$420$k	COMPASS [467]
		$\pi ^{-}\pi ^0\omega (\pi ^{+}\pi ^{-}\pi ^0)$	$b_1(1235)\pi ^{-}$	$1664\pm 8\pm 10$	$185\pm 25\pm 28$	$145$k	E852 [468]
		$\pi ^{-}\pi ^{-}\pi ^{+}\eta (\gamma \gamma )$	$f_1(1285)\pi ^{-}$	$1709\pm 24\pm 41$	$403\pm 80\pm 115$	$69$k	E852 [469]
		$\pi ^{-}\pi ^{-}\pi ^{+}\eta (\gamma \gamma )$	$\eta ^{\prime }\pi ^{-}$	$1597\pm 10^{+45}_{-10}$	$340\pm 40\pm 50$	$6$k	E852 [470]
$\pi _1(2015)$	$1^{-\,\!+}$	$\pi ^{-}\pi ^0\omega (\pi ^{+}\pi ^{-}\pi ^0)$	$b_1(1235)\pi ^{-}$	$2014\pm 20\pm 16$	$230\pm 32\pm 73$	$145$k	E852 [468]
		$\pi ^{-}\pi ^{-}\pi ^{+}\eta (\gamma \gamma )$	$f_1(1285)\pi ^{-}$	$2001\pm 30\pm 92$	$333\pm 52\pm 49$	$69$k	E852 [469]
$\pi (1800)$	$0^{-\,\!+}$	$3\pi ^{-}2\pi ^{+}$	$f_0(1500)\pi ^{-}$	$1781\pm 5^{+1}_{-6}$	$168\pm 9^{+5}_{-14}$	$200$k	COMPASS [471]
		$\pi ^{+}\pi ^{-}\pi ^{-}$	$f_0(980)\pi ^{-}$	$1785\pm 9^{+12}_{-6}$	$208\pm 22^{+21}_{-37}$	$420$k	COMPASS [467]
		$\eta (\gamma \gamma )\eta (\pi ^{+}\pi ^{-}\pi ^0)\pi ^{-}$	$a_0(980)\eta$, $f_0(1500)\pi ^{-}$	$1876\pm 18\pm 16$	$221\pm 26\pm 38$	$4$k	E852 [472]
		$\pi ^{+}\pi ^{-}\pi ^{-}$	$f_0(980)\pi ^{-}$	$1774\pm 18\pm 20$	$223\pm 48\pm 50$	$250$k	E852 [473]
		$\pi ^{+}\pi ^{-}\pi ^{-}$	$(\pi \pi )_S\pi ^{-}$	$1863\pm 9\pm 10$	$191\pm 21\pm 20$	$250$k	E852 [473]
		$\eta (\pi ^{+}\pi ^{-}\pi ^0)\eta (\gamma \gamma )\pi ^{-}$	$a_0(980)\eta$	$1840\pm 10\pm 10$	$210\pm 30\pm 30$	$1$k	VES [474]
		$\pi ^{+}\pi ^{-}\pi ^{-}$	$f_0(980)\pi ^{-}$, $(\pi \pi )_S\pi ^{-}$	$1775\pm 7\pm 10$	$190\pm 15\pm 15$	$2000$k	VES [475]
		$K^{+}K^{-}\pi ^{-}$	$f_0(980)\pi ^{-}$, $K_0^*(800) K^{-}$	$1790\pm 14$	$210\pm 70$	$145$k	VES [476]
		$\eta ^{\prime }(\pi ^{+}\pi ^{-}\eta (\gamma \gamma ),\rho ^0\gamma )\eta (\gamma \gamma )\pi ^{-}$	$\eta ^{\prime }\eta \pi ^{-}$	$1873\pm 33\pm 20$	$225\pm 35\pm 20$	$1.9$k	VES [477]
		$\eta (\pi ^{+}\pi ^{-}\pi ^0)\eta (\gamma \gamma )\pi ^{-}$	$\eta \eta \pi ^{-}$	$1814\pm 10\pm 23$	$205\pm 18\pm 32$	$0.4$k	VES [478]
		$\pi ^{+}\pi ^{-}\pi ^{-}$	$(\pi \pi )_S\pi ^{-}$	$1770\pm 30$	$310\pm 50$	$120$k	SERP [479]
$\pi _2(1880)$	$2^{-\,\!+}$	$3\pi ^{-}2\pi ^{+}$	$f_2(1270)\pi ^{-}$, $a_1(1260)\rho$,				
			$a_2(1320)\rho$	$1854\pm 6^{+6}_{-4}$	$259\pm 13^{+7}_{-17}$	$200$k	COMPASS [471]
		$\eta (\gamma \gamma )\eta (\pi ^{+}\pi ^{-}\pi ^0)\pi ^{-}$	$a_2(1320)\eta$	$1929\pm 24\pm 18$	$323\pm 87\pm 43$	$4$k	E852 [472]
		$\pi ^{-}\pi ^0\omega (\pi ^{+}\pi ^{-}\pi ^0)$	$\omega \rho ^{-}$	$1876\pm 11\pm 67$	$146\pm 17\pm 62$	$145$k	E852 [468]
		$\pi ^{-}\pi ^{-}\pi ^{+}\eta (\gamma \gamma )$	$f_1(1285)\pi ^{-}$, $a_2(1320)\eta$	$2003\pm 88\pm 148$	$306\pm 132\pm 121$	$69$k	E852 [469]
		$\pi ^{0} \pi ^{0}\eta (\gamma \gamma )\eta (\gamma \gamma )$	$a_2(1320)\eta$	$1880\pm 20$	$255\pm 45$	$15$k	CB [480]
$\eta _2(1870)$	$2^{-\,\!+}$	$\eta (\gamma \gamma ,\pi ^{+}\pi ^{-}\pi ^0)\pi ^{+}\pi ^{-}$	$a_2(1320)\pi$, $a_0(980)\pi$	$1835\pm 12$	$235\pm 23$		WA102 [481]
		$2\pi ^{+}2\pi ^{-}$, $\pi ^{+}\pi ^{-}\pi ^{0} \pi ^{0}$	$a_2(1320)\pi$	$1844\pm 13$	$228\pm 23$	$1500$k	WA102 [482]
		$2\pi ^{+}2\pi ^{-}$	$a_2(1320)\pi$	$1840\pm 25$	$200\pm 40$	$1200$k	WA102 [483]
		$\eta (\gamma \gamma )3\pi ^0$	$f_2(1270)\eta$	$1875\pm 20\pm 35$	$200\pm 25\pm 45$	$5$k	CB [484]
		$\eta (\gamma \gamma )\pi ^{0} \pi ^{0}$	$a_2(1320)\pi$, $a_0(980)\pi$	$1881\pm 32\pm 40$	$221\pm 92\pm 44$	$1.2$k	CBall [485]
$\rho (1450)$	$1^{-\,\!-}$	$\pi \pi$, $4\pi$, $e^{+}e^{-}$		$1465\pm 25$	$400\pm 60$		PDG est. [1]
$\rho (1570)$	$1^{-\,\!-}$	$K^{+}K^{-}\pi ^0$	$\phi \pi ^0$	$1570\pm 36\pm 62$	$144\pm 75 \pm 43$	$54$	BABAR [486]

A much bigger data set was taken by the same experiment with a liquid hydrogen target, surpassing the existing world data set by about one order of magnitude [491, 492]. For both the Pb and the H targets a large broad nonresonant contribution at lower masses is needed to describe the mass dependence of the spin-density matrix. First studies suggest that the background can be reasonably well described by Deck-like processes [493] which proceed through 1-pion exchange. A more refined analysis in bins of $3\pi$ mass and $t$ is being performed on the larger data set and is expected to shed more light on the relative contribution of resonant and nonresonant processes in this and other waves.

**Fig. 22:**
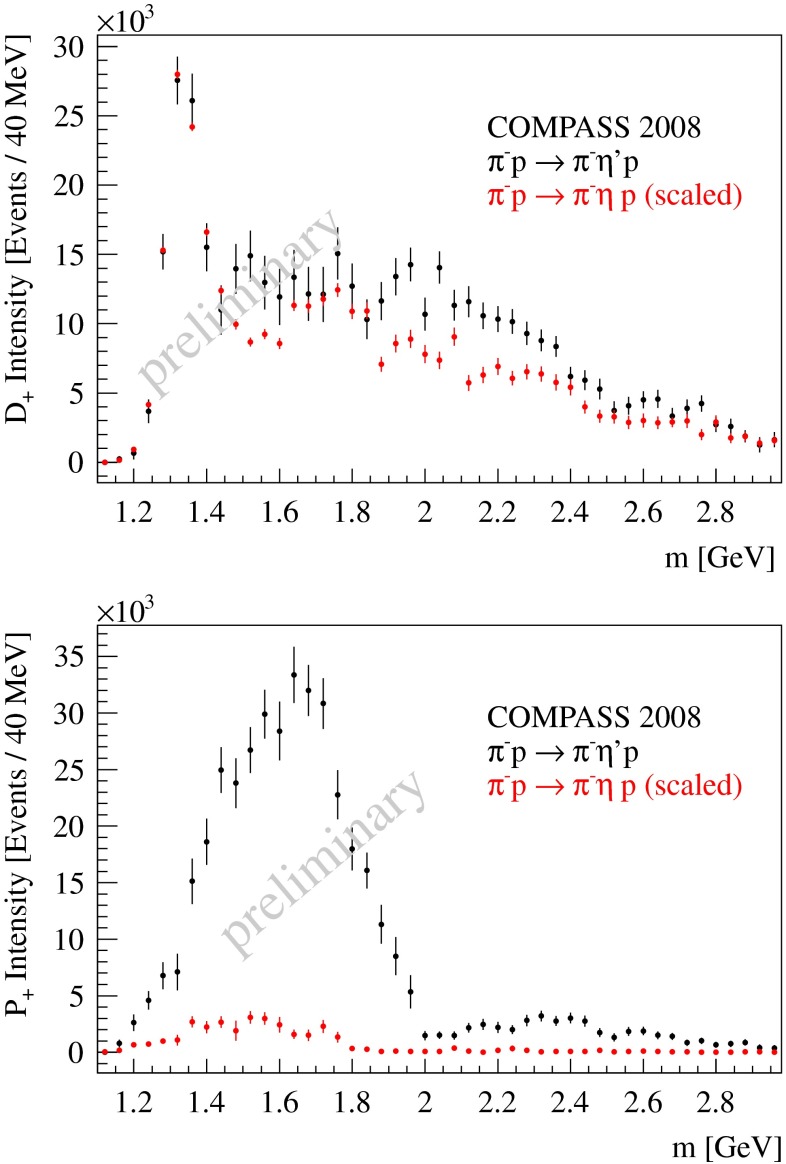
Comparison of waves for $\eta \pi$ (*red data points*) and $\eta '\pi$ (*black data points*) final states. *Top* Intensity of the $J^\mathrm{PC}=2^{+\,\!+}$ $D$ wave, *bottom* intensity of the spin-exotic $1^{-+}$ $P$ wave from COMPASS [494]

COMPASS has also presented data for $\eta \pi$ ($\eta \rightarrow \pi ^+\pi ^{-}\pi ^0$) and $\eta '\pi$ ($\eta '\rightarrow \pi ^+\pi ^{-}\eta$, $\eta \rightarrow \gamma \gamma$) final states from diffractive scattering of $\pi ^{-}$ off the H target [494], which exceed the statistics of previous experiments by more than a factor of $5$. Figure 22 shows the intensities in the (top panel) $2^{+\,\!+}1^+$ and (bottom panel) $1^{-+}1^+$ waves for the $\eta '\pi$ (black data points) and the $\eta \pi$ final state (red data points), respectively, where the data points for the latter final state have been scaled by a phase-space factor. While the intensities in the $D$ wave are remarkably similar in intensity and shape in both final states after normalization, the $P$ wave intensities appear to be very different. For $\eta \pi$, the $P$ wave is strongly suppressed, while for $\eta '\pi$ it is the dominant wave. The phase differences between the $2^{+\,\!+}1^+$ and the $1^{-+}1^+$ waves agree for the two final states for masses below $1.4\,~{\mathrm {GeV}}$, showing a rising behavior due to the resonating $D$ wave, while they evolve quite differently at masses larger than $1.4\,~{\mathrm {GeV}}$, suggesting a different resonant contribution in the two final states. As for the $3\pi$ final states, resonant, as well as nonresonant, contributions to the exotic wave have to be included in a fit to the spin-density matrix in order to describe both intensities and phase shifts [494]. Regardless of this, the spin-exotic contribution to the total intensity is found to be much larger for the $\eta '\pi$ final state than for the $\eta \pi$ final state, as expected for a hybrid candidate.

**Fig. 23:**
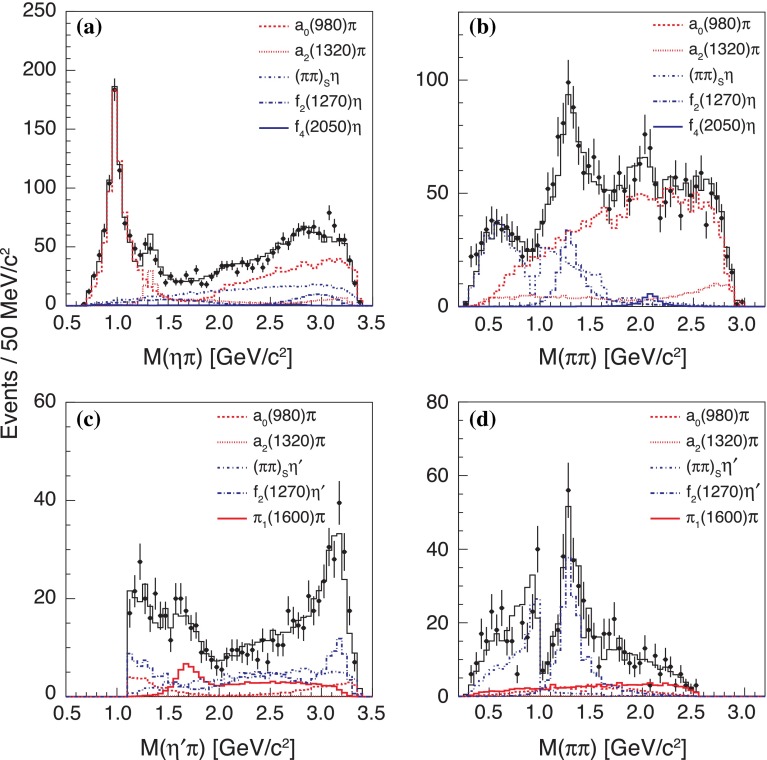
Invariant mass projections from the analyses of **a**, **b** $\chi _{c1}\rightarrow \eta \pi ^+\pi ^{-}$, and **c**, **d** $\chi _{c1}\rightarrow \eta '\pi ^+\pi ^{-}$ measured by CLEO-c [496]. The contributions of the individual fitted decay modes are indicated by *lines*, the data points with full points

The CLEO-c detector [495] at the Cornell Electron Storage Ring studied charmed mesons at high luminosities until 2008. The advantage of using charmonium states as a source for light-quark states is a clearly defined initial state, which allows one to limit the available decay modes and to select the quantum numbers through which the final state is reached. Using the full CLEO-c data sample of $25.9\times 10^{6}$ $\psi (2S)$ decays, an amplitude analysis of the decay chains $\psi (2S)\rightarrow \gamma \chi _{c1}$, with $\chi _{c1}\rightarrow \eta \pi ^+\pi ^{-}$ or $\chi _{c1}\rightarrow \eta '\pi ^+\pi ^{-}$ has been performed [496]. For these final states, the only allowed $S$-wave decay of the $\chi _{c1}$ goes through the spin-exotic $1^{-+}$ wave, which then decays to $\eta (')\pi$. There was no need to include a spin-exotic wave for the $\eta \pi ^+\pi ^{-}$ final state, for which 2498 events had been observed. In the $\eta '\pi ^+\pi ^{-}$ channel with 698 events, a significant contribution of an exotic $\pi _1$ state decaying to $\eta '\pi$ is required in order to describe the data, as can be seen from Fig. 23. This is consistent with the COMPASS observation of a strong exotic $1^{-+}$ wave in the same final state in diffractive production, and is the first evidence of a light-quark meson with exotic quantum numbers in charmonium decays.

**Fig. 24:**
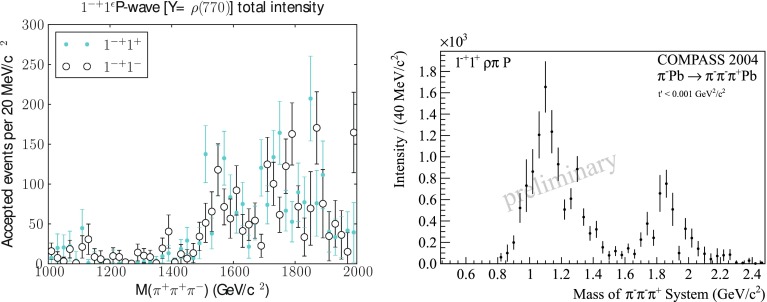
Intensity of the $1^{-+}1^\pm \,\rho \pi \,P$ waves from photoproduction at (*left*) CLAS [499] and (*right*) COMPASS [488] as a function of $3\pi$ invariant mass

The CEBAF Large Acceptance Spectrometer (CLAS) [497] at Hall B of JLab is studying photo- and electro-induced hadronic reactions by detecting final states containing charged and neutral particles. Since the coverage for photon detection is limited in CLAS, undetected neutral particles are inferred mostly via energy-momentum conservation from the precisely measured 4-momenta of the charged particles. CLAS investigated the reaction $\gamma p\rightarrow \Delta ^{+\,\!+}\eta \pi ^{-}$ in order to search for an exotic $\pi _1$ meson decaying to the $\eta \pi$ final state [498]. They found the $J^{\mathrm{PC}}=2^{+\,\!+}$ wave to be dominant, with Breit–Wigner parameters consistent with the $a_2(1320)$. No structure or clear phase motion was observed for the $1^{-+}$ wave. Two CLAS experimental campaigns in 2001 and 2008 were dedicated to a search for exotic mesons photoproduced in the charge exchange reaction $\gamma p\rightarrow \pi ^+\pi ^+\pi ^{-} (n)$. The intensity of the exotic $1^{-+}1^\pm \,\rho \pi \,P$ wave, shown in Fig. 24 (left) as a function of the $3\pi$ invariant mass, does not exhibit any evidence for structures around $1.7\,~{\mathrm {GeV}}$. Also its phase difference relative to the $2^{-+}1^\pm \,f_2\pi \,S$ wave does not suggest any resonant behavior of the $1^{-+}$ wave in this mass region.

The conclusion from the CLAS experiments is that there is no evidence for an exotic $1^{-+}$ wave in photoproduction. This is in contradiction to some models [500–502], according to which photoproduction of mesons with exotic quantum numbers was expected to occur with a strength comparable to $a_2(1320)$ production.

The COMPASS experiment studied pion-induced reactions on a Pb target at very low values of 4-momentum transfer, which proceed via the exchange of quasi-real photons from the Coulomb field of the heavy nucleus. A partial wave analysis of this data set does not show any sign of a resonance in the exotic $1^{-+}1^\pm \,\rho \pi \,P$ wave at a mass of $1.7\,~{\mathrm {GeV}}$ (see Fig. 24), consistent with the CLAS observation.

While there is some evidence for an isovector member of a light $1^{-+}$ exotic nonet, as detailed in the previous paragraphs, members of non-exotic hybrid multiplets will be more difficult to identify. Most of the light meson resonances observed until now are in fact compatible with a $q\overline{q}'$ interpretation. Taking the lattice-QCD predictions as guidance, the lowest isovector hybrids with ordinary quantum numbers should have $J^\mathrm{PC}=0^{-+}$, $1^{-\,\!-}$, and $2^{-+}$ (see Sect. 3.3.1a). In the following paragraphs, recent experimental results for states with these quantum numbers are summarized.

There is clear experimental evidence for the $\pi (1800)$ [1]. The latest measurements of this state come from the COMPASS experiment which observes it in the $3\pi$ and $5\pi$ final states, using a $190\,~{\mathrm {GeV}}$ $\pi ^{-}$ beam impinging on a Pb target. Table 3 includes the masses and widths obtained by fitting Breit–Wigner functions to the spin density matrix. More statistics and advanced coupled-channel analyses are certainly needed to clarify the decay pattern and thus the hybrid or $3S$ $q\overline{q}'$ interpretation of this state.

There is growing experimental evidence for the existence of the $\pi _2(1880)$. The latest high-statistics measurements of this state again come from COMPASS. For both Pb and H targets a clear peak is observed in the intensity of the $2^{-+}0^+\,f_2\pi \,D$ wave of the $3\pi$ final state [491], which is shifted in mass with respect to the $\pi _2(1670)$, and also exhibits a phase motion relative to the latter in the $f_2\pi \,S$ wave. This observation, however, was also explained differently, including, e.g., the interference of the $f_2\pi \,S$ wave with a Deck-like amplitude, which shifts the true $\pi _2$ peak to lower masses [503]. For $5\pi$ final states [471], a total of three resonances are needed to describe the $2^{-+}$ sector, the $\pi _2(1670)$, the $\pi _2(1880)$, and a high-mass $\pi _2(2200)$. The resulting mass and width deduced from this fit for the $\pi _2(1880)$ are also included in Table 3. A possible isoscalar partner of the $\pi _2(1880)$, the $\eta _2(1870)$ has also been reported [1], but needs confirmation.

The PDG lists two $\rho$-like excited states, the $\rho (1450)$ and the $\rho (1700)$, observed in $e^+e^{-}$ annihilation, photoproduction, antiproton annihilation and $\tau$ decays [1]. Their masses are consistent with the $2^3S_1$ and $1^3D_1$ $q\overline{q}'$ states, respectively, but their decay patterns do not follow the $^3P_0$ rule [504]. The existence of a light vector hybrid state, mixing with the $q\overline{q}'$ states, was proposed to solve these discrepancies [505]. Recently, BaBar has reported the observation of a $1^{-\,\!-}$ state decaying to $\phi \pi ^0$ [486], the $\rho (1570)$, which might be identical to an earlier observation in Serpukhov [506]. Interpretations of this signal include a new state, a threshold effect, and an OZI-suppressed decay of the $\rho (1700)$. A very broad vector state with pole position $M=(1576^{+49+98}_{-55-91}+\frac{i}{2} 818^{+22+64}_{-23-133})\,~\mathrm {MeV}$ has been reported by BES [507] and is listed as $X(1575)$ by the PDG [1]. It has been interpreted to be due to interference effects in final-state interactions, and in tetraquark scenarios. In conclusion, there is no clear evidence for a hybrid state with vector quantum numbers. A clarification of the nature of the $\rho$-like states, especially above $1.6\,~{\mathrm {GeV}}$, requires more data than those obtained in previous ISR measurements at BaBar and Belle, which will hopefully be reached in current $e^+e^{-}$ experiments (CMD-3 and SND at the VEPP-2000 collider, BES III at BEPCII) as well as with ISR at the future Belle II detector.

The final test for the hybrid hypothesis of these candidate states will, of course, be the identification of the isoscalar and strange members of a multiplet. Identification of some reasonable subset of these states is needed to experimentally confirm what we now expect from lattice QCD. New experiments with higher statistical significance and better acceptance, allowing for more elaborate analysis techniques, are needed in order to shed new light on these questions.

*c. Light baryons* Light baryon resonances represent one of the key areas for studying the strong QCD dynamics. Despite large efforts, the fundamental degrees of freedom underlying the baryon spectrum are not yet fully understood. The determinations of baryon resonance parameters, namely quantum numbers, masses and partial widths and their structure such as electromagnetic (EM) helicity amplitudes are currently among the most active areas in hadron physics, with a convergence of experimental programs, and analysis and theoretical activities. An appraisal of the present status of the field can be found in [346]. Many important questions and open problems motivate those concerted efforts. Most important among them is the problem of missing resonances: in quark models based on approximate flavor SU(3) symmetry it is expected that resonances form multiplets; many excited states are predicted which have not been observed (for a review see [508]), with certain configurations seemingly not realized in nature at all [509]. More recently lattice-QCD calculations (at relatively large quark masses) [371] also predict a similar proliferation of states. Do (some of) those predicted states exist, and if so, is it possible to identify them in the experimental data? In addition to $N$ and $\varDelta$ baryons made of $u$ and $d$ quarks, the search for hyperon resonances remains an important challenge. Efforts in that direction are ongoing at current facilities, in particular at JLab (CLAS), where studies of $S=-1$ excited hyperons, e.g., in photoproduction of $\Lambda (1405)$ [510, 511], have been completed. A program to study hyperons with $S=-1$, $-2$, and even $-3$ is part of the CLAS12 upgrade.

Another important task is quantifying and understanding the structure of resonances, which still is in its early stages. Experimentally, one important access to structure is provided by measurements at resonance electro-production, as exemplified by recent work [512, 513] where the EM helicity amplitudes $A_{1/2}(Q^2)$ (electro-couplings) of the Roper and $N$(1520) resonances have been determined from measurements at CLAS, an effort that will continue with the CLAS12 program. An additional tool is provided by meson transition couplings which can be obtained from single meson EM production. Both experimental and theoretical studies of resonance structure are key to further progress.

Since most of the information on light-quark baryon resonances listed in [1] comes from partial wave analyses of $\pi N$ scattering, one possible reason why many predicted resonances were not observed may be due to small couplings to $\pi N$. Additional information may come from the observation of other final states like $\eta N$, $\eta ' N$, $KY$, $\omega N$, or $2\pi N$. A significant number of the current and future experimental efforts are in electro- and photoproduction experiments, namely JLab (CLAS and CLAS12), Mainz (MAMI-C), Bonn (ELSA) and Osaka (SPring8-LEPS). Experiments with proton beams are being carried out at CERN (COMPASS), J-PARC (Japan; also K beam), COSY and GSI (Germany), and at the proton synchrotron at ITEP (Russia). Resonance production in charmonium decays (BES III and CLEO-c) is also an important source of new excited baryon data.

**Fig. 25:**
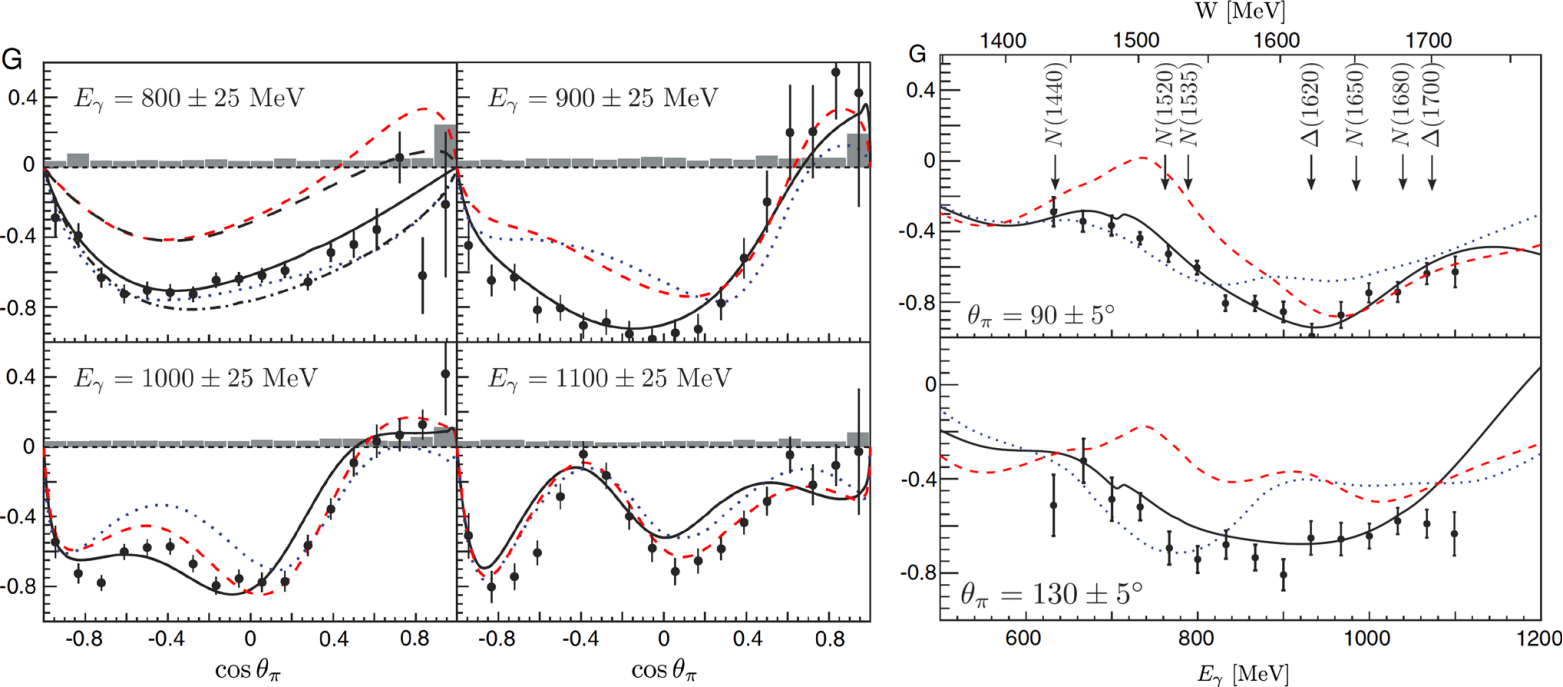
Double-polarization observable $G$ measured at CBELSA [515], (*left*) as a function of $\cos {\theta _\pi }$ for four different photon energies, (*right*) as a function of photon energy for two different pion polar angles $\theta _\pi$, compared to predictions by different PWA formalisms, (*blue*) SAID, (*red*) BnGa, (*black*) MAID

As in the light-meson sector, the broad and overlapping nature of baryon resonances in the mass region below $2.5\,~{\mathrm {GeV}}$ requires the application of sophisticated amplitude or partial-wave analyses in order to disentangle the properties of the contributing states. Partial wave analysis is currently a very active area, with several important groups employing different methods and models. Among the groups are SAID (George Washington Univ.), MAID (Mainz), EBAC (Jefferson Lab), Bonn-Gatchina (BnGa), Bonn-Jülich, Valencia, Gießen, and others. While at present the analyses are based to the largest extent on $\pi N$ and $K N$ data, the large data sets already accumulated and to be acquired in the near future in photo- and electroproduction are expected to have a big impact in future analyses.

The extraction of amplitudes from the measured differential cross sections suffers from ambiguities, as the latter are bilinear products of amplitudes. These ambiguities can be resolved or at least minimized by imposing physical constraints on the amplitudes, or by measuring a well-chosen set of single and double polarization observables which further constrain the problem. For photoproduction experiments, a “complete experiment” to extract the full scattering amplitude unambiguously [514] requires a combination of linearly and circularly polarized photon beams, longitudinally and transversely polarized targets (protons and neutrons), or the polarization of the recoil nucleon, measured for each energy. These amplitudes are then expanded in terms of partial waves, which are usually truncated at some values of angular momentum. Such measurements are one of the main objectives for the near future, which will give unprecedented detailed access to established baryon resonances, means to confirm or reject less established ones and also possibly lead to the discovery of new resonances.

Even for the simplest photoproduction reaction, $\gamma p\rightarrow \pi ^0 p$, recently investigated in a double-polarization experiment at CBELSA/TAPS (Bonn) using linearly polarized photons hitting longitudinally polarized protons [515], discrepancies between the latest PWA predictions and the data were found at rather low energies in the region of the four-star resonances $N(1440)$, $N(1535)$, and $N(1520)$. Figure 25 (left) shows the observable $G$ as a function of $\cos {\theta _\pi }$ for four different photon energies, where $\theta _\pi$ is the polar angle of the outgoing pion, compared to predictions by several PWA formalisms. $G$ is the amplitude of a $\sin {2\phi _\pi }$ modulation of the cross section in a double-polarization experiment, where $\phi _\pi$ is the azimuthal angle of the produced pion.Figure 25 (right) shows $G$ as a function of the photon energy $E_\gamma$ for two selected bins in pion polar angle $\theta _\pi$. The differences in the theory predictions arise from different descriptions of two multipoles, $E_{0^+}$ and $E_{2^{-}}$, in the three analyses, which are related to the properties of the $N(1520)$ $J^P=\frac{3}{2}^{-}$ and $N(1535)\frac{1}{2}^{-}$ resonances, respectively.

**Fig. 26:**
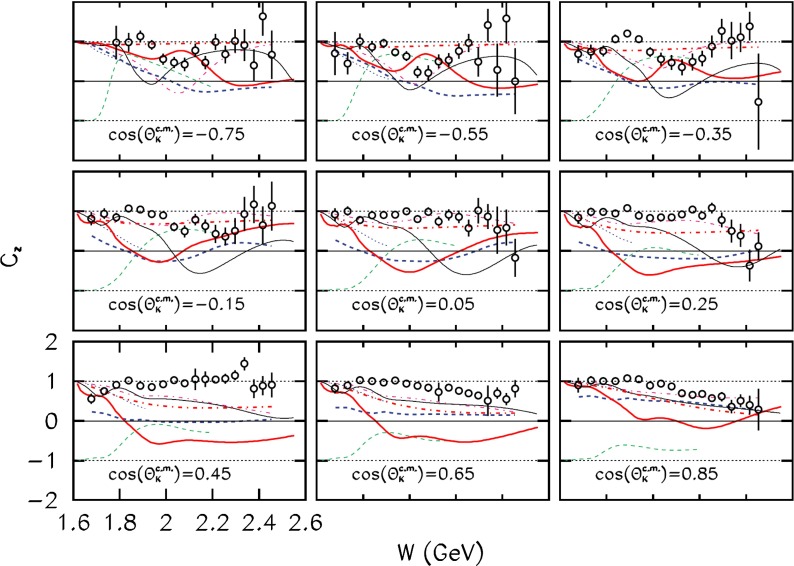
Beam-recoil observable $C_z$ for circularly polarized photons in the reaction $\gamma p\rightarrow K^+\Lambda$ as a function of $\gamma$–$p$ CM energy for different kaon polar angles $\theta _K^{\mathrm {CM}}$ measured by CLAS [516]. The data points are compared to different models (see [516] for details)

Photoproduction of strangeness, where a hyperon is produced in association with a strange meson, e.g., $\gamma p\rightarrow K Y$ ($Y=\Lambda ,\Sigma$), provides complementary access to nonstrange baryon resonances that may couple only weakly to single-pion final states. In addition, the self-analyzing weak decay of hyperons offers a convenient way to access double polarization observables, as has been recently exploited at CLAS and GRAAL. Using a beam of circularly polarized photons, the polarization transfer to the recoiling hyperon along orthogonal axes in the production plane is characterized by $C_x$ and $C_z$. The CLAS collaboration [516] reported that for the case of $\Lambda$ photoproduction the polarization transfer along the photon momentum axis $C_z\sim +1$ over a wide kinematic range (see Fig. 26), and the corresponding transverse polarization transfer $C_x\sim C_z-1$. The magnitude of the total $\Lambda$ polarization vector $\sqrt{P^2+C_x^2+C_z^2}$, including the induced polarization $P$, is consistent with unity at all measured energies and angles for a fully polarized photon beam, an observation which still lacks a proper understanding. Consistent results were obtained by GRAAL [517] for the double polarization observables $O_{x,z}$ using linearly polarized photons.

Decays to vector mesons provide additional polarization information by a measurement of the spin-density matrix, which constrains the PWA of the reaction. Additionally, the photoproduction of $\omega$ mesons, like that of $\eta$, serves as an isospin filter for $N^*$ resonances. A PWA based on a recent high-statistics CLAS measurement of the unpolarized cross section of the reaction $\gamma p\rightarrow \omega p$ at CM energies up to $2.4\,~{\mathrm {GeV}}$ [518] required contributions from at least two $\frac{5}{2}^+$ resonances, identified as the $N(1680)\frac{5}{2}^+$ and $N(2000)\frac{5}{2}^+$, and a heavier $N(2190)\frac{7}{2}^{-}$ resonance. The latter had previously only been observed in $\pi N$ scattering, and was confirmed more recently by CBELSA/TAPS in $\pi ^0$ photoproduction [519].

As a consequence of the recent high-statistics data sets from photoproduction, in particular for the reaction $\gamma p\rightarrow K^+\Lambda$, several baryon resonances, some of which had previously been only weakly observed in $\pi N$ scattering, have been newly proposed in a recent multichannel analysis of the Bonn-Gatchina PWA group [520] and are now listed in the 2012 PDG review [1]. Table 4 shows the new states in bold letters.

**Table 4:** Summary of new light-quark baryon resonances (in bold) proposed in [520] and listed in the 2012 review of particle physics [1]

$J^P$	Resonance region			
$1/2^{+}$	$N(1440)$****	$N(1710)$***	$\varvec{N(1880)}$ **	$\mathbf {N(2100)}$ *
$1/2^{-}$	$N(1535)$****	$N(1650)$****	$\varvec{N(1895)}$ **	
$3/2^{+}$		$N(1720)$****	$N(1900)$***	$\varvec{N(2040)}$ *
$3/2^{-}$	$N(1520)$****	$N(1700)$***	$\varvec{N(1875)}$ ***	$\varvec{N(2120)}$ **
$5/2^{+}$		$N(1680)$****	$\varvec{N(1860)}$ **	$N(2000)$**
$5/2^{-}$		$N(1675)$****		$\varvec{N(2060)}$ **
$3/2^{-}$		$\Delta (1700)$***	$\Delta (1940)$**	

Some solutions of the partial wave analyses of the world data seem to indicate the existence of parity doublets at higher masses [392, 509], i.e., two approximately degenerate states with the same spin but opposite parity (see also Table 4). This is consistent with predictions based on the effective restoration of chiral symmetry at high baryon masses [521, 522]. Similar patterns, however, are also predicted in models which do not make explicit reference to chiral symmetry [523, 524]. In contrast, the most recent lattice-QCD calculations of excited, higher-spin baryon masses [371] uncover no evidence for the existence of parity doublets. Thus, the question of whether or not chiral doublets exist in the upper reaches of the baryon spectrum remains unanswered.

*d. Future directions* Spectroscopy of light hadrons will remain an active field of research in the future. In order to arrive at a full understanding of the excitation spectrum of QCD, a departure from simplistic Breit–Wigner resonance descriptions towards a full specification of the pole positions of the amplitude in the complex plane, including dynamical effects, thresholds, cusps, is required. As masses increase, multiparticle channels open up, leading to broad and overlapping resonances. Partial-wave analysis models have to be extended to fully respect unitarity, analyticity, and crossing symmetry, in order to extract fundamental, process-independent quantities. The rigorous way of determining the poles and residues of the amplitude from experiment, which has been performed at physical values of $s$ and $t$, is by means of dispersion relations, which provide the correct analytic extension of the amplitudes to the complex plane. If and how these can be incorporated into fit models for multiparticle final states remains an open question. A clear separation of resonant and nonresonant contributions, a recurring question for many of the observed signals in the light meson sector, e.g., requires coupled-channel analyses of different final states, but also studies in different reactions and kinematics in order to clarify the underlying production mechanisms.

New results from running experiments are to be expected in the near future. The extraction of polarization observables for baryon resonances in electromagnetically induced reactions will continue at ELSA and MAMI, which in turn will provide input to multichannel PWA. COMPASS, whose data set with hadron beams ($\pi$, $p$, $K$) is currently being analyzed, will continue to take data for a couple of years with muon and pion beams  [525]. New experiments are on the horizon or have already started to take data, which are expected to considerably advance our understanding of the excitation spectrum of QCD. Key features of these experiments will be large data sets requiring highest possible luminosities and sensitivity to production cross sections in the sub-nanobarn region. This can only be achieved by hermetic detectors with excellent resolution and particle identification capabilities, providing a very high acceptance for charged and neutral particles.

Although not their primary goal, $e^{+} e^{-}$ machines, operating at charmonium or bottomonium center-of-mass energies, have initiated a renaissance of hadron spectroscopy in the past few years by discovering many new and yet unexplained states containing charm and bottom quarks. In $e^{+} e^{-}$ collisions states with photon quantum numbers are directly formed. Other states including exotics can be accessed via hadronic or radiative decays of heavy mesons, or are produced recoiling against other particles. Hadronic decays of heavy-quark states may serve as a source for light-quark states, with a clearly defined initial state facilitating the partial wave analysis. BES III at the BEPCII collider in Beijing has already started to take data in the $\tau$-charm region with a luminosity of $10^{33}\,\mathrm {cm}^{-2}\,\mathrm {s}^{-1}$ at a CM energy of $2\times 1.89\,~{\mathrm {GeV}}$, and will continue to do so over the next years. The Belle II experiment at SuperKEKB [526], aiming at a 40-fold luminosity increase to values of $8\times 10^{35}\,\mathrm {cm}^{-2}\,\mathrm {s}^{-1}$, is expected to increase the sensitivity for new states in the charm and bottom sector dramatically, but will also feed the light-quark sector. Experiments at the LHC, especially LHCb with its excellent resolution, are also expected to deliver high-statistics data on the meson spectrum.

GlueX [527] is a new experiment which will study photoproduction of mesons with masses below $3\,~{\mathrm {GeV}}$ at the $12\,~{\mathrm {GeV}}$ upgrade of CEBAF at JLab. An important advantage of the experiment will be the use of polarized photons, which narrows down the possible initial states and gives direct information on the production process. Hadron spectroscopy in Hall B of JLab will be extended to a new domain of higher mass resonances and the range of higher transferred momentum using electron beams up to $11\,~{\mathrm {GeV}}$ and the upgraded CLAS12 detector [528]. In addition to studying GPDs, CLAS12 will perform hadron spectroscopy using photoproduction of high-mass baryon and meson resonances, either by electron scattering via quasi-real photons or by high-energy real photon beams. The detector will consist of a forward detector, making use of partly existing equipment with new superconducting torus coils, and a central detector with a new $5\,\mathrm {T}$ solenoid magnet and a barrel tracker, providing nearly $4\pi$ solid angle coverage for hadronic final states.

PANDA, a new experiment at the FAIR antiproton storage ring HESR, is designed for high-precision studies of the hadron spectrum in the charmonium mass range [529]. In $\overline{p}p$ annihilations, all states with non-exotic quantum numbers can be formed directly. Consequently, the mass resolution for these states is only limited by the beam momentum resolution. Spin-exotic states can be obtained in production experiments. PANDA is expected to run at center-of-mass energies between $2.3$ and $5.5\,~{\mathrm {GeV}}$ with a maximum luminosity of $2\times 10^{32}\,\mathrm {cm}^{-2}\,\mathrm {s}^{-1}$. As for the $e^+e^{-}$ machines, hadronic decays of heavy hadrons may also serve as a well-defined source for light mesons. The study of multistrange hyperons in proton–antiproton annihilation is also foreseen in the PANDA experiment.

### Chiral dynamics

The low-energy regime of light hadron physics plays a key role in tests of the non-perturbative phenomena of QCD. In particular, the approximate chiral $\mathrm{SU}_L(3)\times \mathrm{SU}_R(3)$ symmetry and its spontaneous breaking sets the stage for low-energy QCD. The rigorous description of low-energy QCD in terms of effective theories, namely Chiral Perturbation Theory (ChPT) in its various versions, the availability of fundamental experiments, and most recently the advent of lattice-QCD calculations with small quark masses, are signs of progress that continues unabated, leading to very accurate tests of QCD’s chiral dynamics.

ChPT is a low-energy effective field theory of QCD, in which the degrees of freedom are the eight Goldstone bosons of the hadronic world, corresponding to the $\pi$, $K$, and $\eta$ mesons, and resulting from the spontaneous breakdown of the chiral $\mathrm{SU}(3)_L\times \mathrm{SU}(3)_R$ symmetry in the limit of massless $u$, $d$, $s$ quarks [530, 531]. ChPT can be readily extended to include the low-energy physics of ground-state light baryons, as well as that of heavy mesons and baryons.

We review here the most salient experimental and theoretical developments that have been accomplished recently in the areas of meson–meson and meson–nucleon dynamics, along with an outlook for the future.

#### $\pi \pi$ and $\pi K$ scattering lengths

Measurements of the $S$-wave $\pi \pi$ scattering lengths represent one of the most precise tests of the $\mathrm{SU}(2)_L^{}\times \mathrm{SU}(2)_R^{}$ sector of chiral dynamics. The NA48/2 experiment at the CERN SPS [446] has analyzed, on the basis of more than one million events, the $K_{e4}$ decay $K^{\pm }\rightarrow \pi ^+\pi ^{-}e^{\pm }\nu$. The analysis of the corresponding form factors, and through them of the $\pi \pi$ final-state interactions, has led to the currently most accurate determination of the $S$-wave isospin-0 and isospin-2 scattering lengths $a_0^0$ and $a_0^2$, where $a_\ell ^I$ denotes the channel with orbital angular momentum $\ell$ and isospin $I$. In this analysis, a crucial role is played by isospin breaking effects [532]. An additional improvement has been attained by combining the latter results with those of the experiment NA48/2 on the nonleptonic decay $K^{\pm }\rightarrow \pi ^{\pm }\pi ^0\pi ^0$, with more than 60 million events, and the impact of the cusp properties at $\pi ^0\pi ^0$ threshold, due to the mass difference between charged and neutral pions. The current results are summarized by:

3.19 $$\begin{aligned}&m_{\pi }a_0^0=0.2210\pm 0.0047_{\mathrm {stat}} \pm 0.0040_{\mathrm {syst}}, \nonumber \\&m_{\pi }a_0^2=-0.0429\pm 0.0044_{\mathrm {stat}} \pm 0.0018_{\mathrm {syst}}, \nonumber \\&m_{\pi }(a_0^0-a_0^2)=0.2639\pm 0.0020_{\mathrm {stat}} \pm 0.0015_{\mathrm {syst}}, \end{aligned}$$

where $m_{\pi }$ is the charged pion mass. The agreement with the ChPT result at two-loop order [533] is striking:

3.20 $$\begin{aligned}&m_{\pi }a_0^0=0.220\pm 0.005, \nonumber \\&m_{\pi }a_0^2=-0.0444\pm 0.0010, \nonumber \\&m_{\pi }(a_0^0-a_0^2)=0.265\pm 0.004. \end{aligned}$$

The $\pi \pi$ scattering amplitude is usually analyzed with the aid of the so-called Roy equations [534], which are fixed-$t$ dispersion relations based on analyticity, crossing symmetry and unitarity. The corresponding representation has been used in [533] to check the consistency of the chiral representation and of the corresponding values of the scattering lengths and to restrict as much as possible the resulting uncertainties. Dispersion relations and Roy equations have also been used in [535], without the input of ChPT, to analyze the $\pi \pi$ scattering amplitude; using high-energy data and the $K_{e4}$ decay measurements, results in agreement with those of [533] have been found.

Recently, the NA48/2 collaboration also measured the branching ratio of $K_{e4}$ decay [536], which permits the determination of the normalization of the corresponding form factors. This in turn can be used for additional tests of ChPT predictions.

On the other hand, the measurement of the $K_{\mu 4}$ decay [537] will give access to the $R$ form factor, which is not detectable in $K_{e4}$ decay, since it contributes to the differential decay rate with a multiplicative factor proportional to the lepton mass squared. $R$ is one of the three form factors associated with the matrix element of the axial vector current; it is mostly sensitive to the matrix element of the divergence of the axial vector current and hence brings information about the chiral symmetry breaking parameters.

Distinct access to the $\pi \pi$ scattering lengths is provided through the DIRAC experiment at CERN, which measures the lifetime of the pionium atom. The atom, because of the mass difference between the charged and neutral pions, decays mainly into two $\pi ^0$’s. The decay width is proportional, at leading non-relativistic order, to $(a_0^0-a_0^2)^2$ [538]. Corrections coming from relativistic effects, photon radiative corrections, and isospin breaking must be taken into account to render the connection between the lifetime and the strong interaction scattering lengths more accurate: these amount to a 6 % effect [539] (and references therein). The DIRAC experiment, which started almost 10 years ago, reached last year the objective of measuring the pionium lifetime with an error smaller than 10 %. From a sample of $21000$ pionium atoms a 4 % measurement of the difference of the $\pi \pi$ scattering lengths has been obtained [540]:

3.21 $$\begin{aligned} m_{\pi }|a_0^0-a_0^2|=0.2533^{+0.0080}_{-0.0078}|_ {\mathrm {stat}}{}^{+0.0078}_{-0.0073}|_{\mathrm {syst}}, \end{aligned}$$

a result which is in agreement with those of (3.20) and (3.19), taking into account the relatively large uncertainty.

In the future, the DIRAC Collaboration also aims to measure the $2s-2p$ energy splitting, which would allow for the separate measurements of the two $S$-wave scattering lengths. Another project of the collaboration is the study of the properties of the $\pi K$ atom, in analogy with the pionium case, thus providing the $S$-wave $\pi K$ scattering lengths [541, 542]. Preliminary tests of the experiment at CERN have already begun [543].

A review of the status of several scattering processes which are sensitive to the spontaneous and explicit chiral symmetry breaking of QCD can be found in [544].

The analysis of the $\pi K$ scattering process is a particularly representative computation in ChPT in the presence of a strange quark. Calculations, similar to those of the $\pi \pi$ scattering amplitude, have been carried out. The elastic scattering amplitude has been evaluated in one- and two-loop order [545, 546]. One finds a slow but reasonable convergence of the results at each step of the evaluation. The $S$-wave isospin 1/2 and 3/2 scattering lengths are found at the two-loop order:

3.22 $$\begin{aligned} m_{\pi }a_0^{1/2}=+0.220,\ \ \ \ \ \ m_{\pi }a_0^{3/2}=-0.047. \end{aligned}$$

The uncertainties, not quoted explicitly, depend on the variations of the parameters that enter in the modeling of the $O(p^6)$ low energy constants.

The experimental values of the scattering lengths are obtained by using Roy–Steiner equations [534, 547], which generalize the Roy equations to the $\pi K$ system, and high-energy data for $\pi K$ scattering [548], leading to:

3.23 $$\begin{aligned} m_{\pi }a_0^{1/2}&= +0.224\pm 0.022,\nonumber \\ m_{\pi }a_0^{3/2}&= -0.0448\pm 0.0077. \end{aligned}$$

The agreement between the ChPT evaluation and the experimental output seems satisfactory, with, however, larger uncertainties than in the $\pi \pi$ case.

Efforts are also being made to extract the $\pi K$ phase shifts from the nonleptonic decays of $D$ and $B$ mesons [549–552]. The results are not yet sufficiently precise to allow for quantitative comparisons with previous work.

In recent years, the lattice-QCD determination of the $\pi \pi$ and $\pi K$ scattering lengths is providing increasingly accurate results in full QCD [553–559]. This work is still maturing, as can be seen in the wide range of both central values and error estimates (some of which are not yet complete). A comparative summary of lattice-QCD results can be found in [559]. Once all sources of uncertainty are controlled, however, one can foresee the time when lattice QCD will compete with and even supersede the experimental extraction of scattering lengths.

#### Lattice QCD calculations: quark masses and effective couplings

While the determination of scattering lengths in lattice QCD is still at an early stage, other quantities, such as quark masses or low-energy constants (LECs) of mesonic ChPT, have been obtained with high overall precision and controlled systematic uncertainties. The “Flavour Averaging Group” (FLAG) has set itself the task of collecting and compiling the available lattice results for phenomenologically relevant quantities [44, 45]. Furthermore, FLAG provides a critical assessment of individual calculations regarding control over systematic effects. Results which satisfy a set of quality criteria are then combined to form global estimates. Here we briefly summarize the results and discussions in [45], related to determinations of the light quark masses and LECs. We focus on QCD with $2+1$ dynamical quarks, which corresponds to a degenerate doublet of $u,d$ quarks, supplemented by the heavier strange quark.

The FLAG estimates for the strange quark mass, $m_\mathrm{s}$, and the average light quark mass, $\hat{m}\equiv \frac{1}{2}(m_u+m_d)$, were obtained by combining the results of [37, 39, 40], with [2] as an important cross check. In the $\mathrm \overline{MS}$ scheme at a scale 2 GeV one finds

3.24 $$\begin{aligned}&\hat{m} = 3.42 \pm 0.06_\mathrm{stat}\pm 0.07_\mathrm{sys}\,\mathrm{MeV}, \end{aligned}$$

3.25 $$\begin{aligned}&{m_\mathrm{s}} = 93.8 \pm 1.5_\mathrm{stat}\pm 1.9_\mathrm{sys}\,\mathrm{MeV}. \end{aligned}$$

The FLAG estimate for  the scheme- and scale-independent ratio $m_\mathrm{s}/\hat{m}$, in which some systematic effects cancel, reads

3.26 $$\begin{aligned} m_\mathrm{s}/\hat{m} = 27.46\pm 0.15_\mathrm{stat}\pm 0.41_\mathrm{sys}. \end{aligned}$$

In order to provide separate estimates for the masses of the up and down quarks, one has to account for isospin breaking effects, stemming from both the strong and electromagnetic interactions. Current lattice estimates of $m_u$ and $m_d$ are mostly based on additional input from phenomenology [39, 40, 560]. In some cases, electromagnetic effects (i.e., corrections to Dashen’s theorem [561]) have been determined via the inclusion of a quenched electromagnetic field [562, 563]. The FLAG results for $m_u, m_d$ are obtained by combining the global lattice estimate for $\hat{m}$ with the ChPT estimate for the ratio $m_u/m_d$ and phenomenological estimates of electromagnetic self-energies. In the $\overline{\mathrm{MS}}$ scheme at 2 GeV this yields

3.27 $$\begin{aligned}&{m_u} = 2.16 \pm 0.09_\mathrm{stat+sys}\pm 0.07_\mathrm{e.m.}\,\mathrm{MeV}, \end{aligned}$$

3.28 $$\begin{aligned}&{m_d} = 4.68 \pm 0.14_\mathrm{stat+sys}\pm 0.07_\mathrm{e.m.}\,\mathrm{MeV}, \end{aligned}$$

3.29 $$\begin{aligned}&m_u/m_d = 0.46\pm 0.02_\mathrm{stat+sys}\pm 0.02_\mathrm{e.m.}. \end{aligned}$$

For a detailed discussion we refer the reader to the FLAG report [45]. The quark mass ratio $Q$, defined by

3.30 $$\begin{aligned} Q^2=(m_\mathrm{s}^2-\hat{m}^2)/(m_\mathrm{d}^2-m_u^2), \end{aligned}$$

is a measure of isospin-breaking effects. By combining (3.24), (3.25), (3.27), and (3.28) one arrives at the lattice estimate

3.31 $$\begin{aligned} Q=22.6\pm 0.7_\mathrm{stat+sys}\pm 0.6_\mathrm{e.m.}. \end{aligned}$$

In addition to providing accurate values for the light quark masses, lattice QCD has also made significant progress in determining the effective couplings of ChPT. This concerns not only the LECs that arise at order $p^2$ in the chiral expansion, i.e., the chiral condensate $\Sigma$ and the pion decay constant in the chiral limit, $F$, but also the LECs that appear at $O(p^4)$. Moreover, lattice QCD can be used to test the convergence properties of ChPT, since the bare quark masses are freely tunable parameters, except for the technical limitation that simulations become less affordable near the physical pion mass.

The recent FLAG averages for the leading-order LECs for QCD with $2+1$ flavors read [45]

3.32 $$\begin{aligned} \Sigma =(265\pm 17)^3\,\mathrm{MeV}^3, \quad F_\pi /F=1.0620\pm 0.0034, \end{aligned}$$

where $F_\pi /F$ denotes the ratio of the physical pion decay constant over its value in the chiral limit. As discussed in detail in Sect. 5 of [45], there are many different quantities and methods which allow for the determination of the LECs of either SU(2) or SU(3) ChPT. The overall picture that emerges is quite coherent, as one observes broad consistency among the results, independent of the details of their extraction. For specific estimates of the $O(p^4)$ LECs we again refer to the FLAG report. Despite the fact that the LECs can be determined consistently using a variety of methods, some collaborations [349, 564] have reported difficulties in fitting their data to SU(3) ChPT for pion masses above 400 MeV. Whether this is due to the employed “partially quenched” setting, in which the sea and valence quark masses are allowed to differ, remains to be clarified.

#### $\mathrm{SU}(3)_L\times \mathrm{SU}(3)_R$ global fits

Due to the relatively large value of the strange quark mass with respect to the masses of the nonstrange quarks, the matter of the convergence and accuracy of $\mathrm{SU}(3)_L^{}\times \mathrm{SU}(3)_R^{}$ ChPT becomes of great importance. In the meson sector, this has been investigated over a long period of time by Bijnens and collaborators [565] at NNLO in the chiral expansion. Taking into account new experimental data, mainly on the $K_{e4}$ and $K_{\ell 3}$ form factors, a new global analysis has been carried out up to $O(p^6)$ effects [566]. The difficulty of the task comes from the fact that the number of LECs at two-loop order, $C_i^r$, is huge and no unambiguous predictions of them are possible. One is left here with educated guesses based on naive dimensional analysis or model calculations. Several methods of estimate have been used and compared with each other. It turns out that the most consistent estimate of the LECs $C_i^r$ comes from their evaluation with the resonance saturation scheme. One is then able to extract from the various experimental data the values of the $O(p^4)$ LECs $L_i^r$. $\mathrm{SU}(3)_L\times \mathrm{SU}(3)_R$ ChPT seems now to satisfy improved convergence properties concerning the expressions of the meson masses and decay couplings, a feature which was not evident in the past evaluations. Nevertheless, the new global fit still suffers from several drawbacks, mainly related to a bad verification of the expected large-$N_\mathrm{c}$ properties of some OZI-rule violating quantities. Another drawback is related to the difficulty of reproducing the curvature of one of the form factors of the $K_{e4}$ decay. Incorporation of latest lattice-QCD results is expected to improve the precision of the analysis.

The question of the convergence of $\mathrm{SU}(3)_L\times \mathrm{SU}(3)_R$ ChPT has also led some authors to adopt a different line of approach. It has been noticed that, because of the proximity of the strange quark mass value to the QCD scale parameter $\Lambda _{\mathrm{QCD}}$, vacuum fluctuations of strange quark loops may be enhanced in OZI-rule violating scalar sectors and hence may cause instabilities invalidating the conventional counting rules of ChPT [567] in that context. To cure that difficulty, it has been proposed to treat the quantities that may be impacted by such instabilities with resummation techniques. Analyses, supported by some lattice-QCD calculations [349, 564], seem to provide a consistent picture of three-flavor ChPT [568], at least for pion masses below about 400 MeV.

The problem of including strangeness in Baryon Chiral Perturbation Theory (BChPT) is, on the other hand, still a wide open question. It is particularly striking in the quark mass expansion of the baryon masses, where very large nonanalytic terms proportional to $M_K^3$ indicate a failure of the chiral expansion, and this happens in every known version of BChPT. In other observables, such as the axial couplings, magnetic moments, and meson-baryon scattering, certain versions of BChPT, namely, those including the decuplet baryons as explicit degrees of freedom, lead to important improvements in its convergence. As discussed later, these latter versions are motivated by the $1/N_\mathrm{c}$ expansion, and they provide several such improvements which lend a strong support to their use.

#### $\eta \rightarrow 3\pi$ and the nonstrange quark masses

A process of particular interest in ChPT is $\eta \rightarrow 3\pi$ decay. This process is due to the breaking of isospin symmetry and therefore should allow for measurements of the nonstrange quark-mass difference. Nevertheless, attempts to evaluate the decay through the Dalitz plot analysis, at one-loop order [569], as well as at two-loop order [570], do not seem successful. One of the difficulties is related to the fit to the neutral-channel Dalitz plot slope parameter $\alpha$, whose experimental value is negative, while ChPT calculations yield a positive value. To remedy difficulties inherent to higher-order effects, a dispersive approach has been suggested, in which $\pi \pi$ rescattering effects are taken into account in a more systematic way [571].

Including new experimental data accumulated during recent years (Crystal Barrel [572], Crystal Ball [573–575], WASA [576, 577], KLOE [578]), several groups have reanalyzed the $\eta \rightarrow 3\pi$ problem. Reference [579], using a modified non-relativistic effective field theory approach, shows that the failure to reproduce $\alpha$ in ChPT can be traced back to the neglect of $\pi \pi$ rescattering effects. References [580] and [581] tackle this problem using the dispersive method, which takes into account higher-order rescattering effects. The two groups use similar methods of approach and the same data, but differ in the imposed normalization conditions. The sign of the parameter $\alpha$ is found to be negative in both works, but it is slightly greater in magnitude than the experimental value. The parameter that measures the isospin-breaking effect is $Q$, defined in terms of quark masses; see (3.30). The value found for $Q$ in [580] is $Q=23.1\pm 0.7$, to be compared with the lattice-QCD evaluation $Q=22.6\pm 0.9$ [45]. (Results of [581] are still preliminary and will not be quoted.)

It is possible to obtain the values of the nonstrange quark masses $m_u$ and $m_d$ from the value of $Q$, provided one has additional information about the strange quark mass $m_\mathrm{s}$ and about $\hat{m}$. Using the lattice-QCD results $m_\mathrm{s}=(93.8\pm 2.4)$ MeV and $\hat{m}=(3.42\pm 0.09)$ MeV [45], calculated in the $\overline{\mathrm{MS}}$ scheme at the running scale $\mu =2$ GeV, one finds [580]

3.33 $$\begin{aligned} m_u=(2.23\pm 0.14)\, \mathrm {MeV},\, \, \, \, m_d=(4.63\pm 0.14)\, \mathrm {MeV}, \end{aligned}$$

which are in good agreement with the lattice-QCD results [45] quoted in (3.27) and (3.28).

Some qualitative differences exist between [580] and [581]. The key point concerns the Adler zeros [582] for the $\eta \rightarrow 3\pi$ decay amplitude, whose existence is derived here as a consequence of a $\mathrm{SU}(2)_L^{}\times \mathrm{SU}(2)_R^{}$ low-energy theorem [583], therefore not using the expansion in terms of the strange quark mass. While the two solutions are close to each other in the physical region, they differ in the unphysical region where the Adler zeros exist. The solution obtained in [580] does not seem to display, for small non-zero values of the nonstrange quark masses, any nearby Adler zeros. However, the authors of [580] point out that the quadratic slopes of the amplitude are not protected by the above mentioned symmetry and might find larger corrections than expected.

If the difference between the results of the above two approaches persists in the future, it might be an indication that the detailed properties of the $\eta \rightarrow 3\pi$ decays are not yet fully under control. A continuous effort seems still to be needed to reach a final satisfactory answer. For the most recent appraisal of the theoretical status, see [584].

On the experimental side, the $\eta \rightarrow 3\pi$ width is determined through the branching ratio from the measurement of the $\eta \rightarrow \gamma \gamma$ width. For a long time, measurements of the latter using the reaction $e^+e^{-}\rightarrow e^+e^{-}\eta$ consistently gave a significantly higher value [1] than that of the old determination via the Primakoff effect [585]. However, a reanalysis of this result based on a new calculation of the inelastic background, due to incoherent $\eta$ photoproduction, brought the Primakoff measurement in line with those at $e^+e^{-}$ colliders [586]. A new Primakoff measurement has been proposed by the PRIMEX Collaboration at JLab, using the 11 GeV tagged photon beam to be delivered to Hall D, with the aim of a width measurement with an error of 3 % or less. Also, the large data base collected by Hall B at JLab contains a large sample of $\eta \rightarrow \pi ^+\pi ^{-}\pi ^0$, of the order of $2\times 10^6$ events, which will significantly improve the knowledge of its Dalitz distribution. A recent precise measurement of $\Gamma _{\eta \rightarrow \gamma \gamma }$ by KLOE [587] shows a high promise of the new measurement planned with KLOE-2.

Isospin-breaking effects are also being investigated with lattice-QCD calculations, as recently reviewed in [588]. The effects of the quark-mass difference $m_d-m_u$ on kaon masses, as well as on nucleon masses, have recently been studied in [560], in which earlier references can also be found. In addition, QED effects have also been included [589–592]. These concern mainly the evaluation of the corrections to Dashen’s theorem [561], which establishes, in the $\mathrm{SU}(3)_L\times \mathrm{SU}(3)_R$ chiral limit, relationships between the electromagnetic mass differences of hadrons belonging to the same $\mathrm{SU}(3)_V$ multiplet. A summary of results regarding the latter subject, as well as about the ratio of the nonstrange quark masses, can be found in [593]. The issue of the ChPT LECs in the presence of electromagnetism and isospin breaking through lattice-QCD calculations is also considered in [594].

#### Other tests with electromagnetic probes

One of the classic low-energy processes is $\pi ^0\rightarrow \gamma \gamma$ decay, which tests the Goldstone boson nature of the $\pi ^0$ and the chiral Adler–Bell–Jackiw anomaly [324, 325]. This subject is considered at the end of Sect. 3.2.7b to which the reader is referred.

One important test remaining to be improved is that of the process $\gamma \pi \rightarrow 2\pi$, whose amplitude $F_{3\pi }$ is fixed in the chiral and low-energy limit by the chiral box anomaly. The two existing results for $F_{3\pi }$, namely the Primakoff one [595] from Serpukhov and the recent analysis [596] of the $e^{-}\pi ^{-}\rightarrow e^{-}\pi ^{-}\pi ^0$ data [597], disagree with each other and with leading order ChPT. Currently, data from COMPASS using the Primakoff effect for measuring $\gamma \pi \rightarrow 2\pi$ are under analysis (for early results on the $2\pi$ invariant mass spectrum see the COMPASS-II proposal [598]), and this result is expected to have a significant impact for experimentally establishing this important process. Recently, and motivated by the COMPASS measurement, a new theoretical analysis of $\gamma \pi \rightarrow 2\pi$ has been carried out [599], in which the whole kinematic domain of this process is taken into account using ChPT supplemented with dispersion relations. In particular, this analysis gives also information that can be used to describe more accurately the amplitude $\pi ^0\gamma \gamma ^*$, important in the analysis of light-by-light scattering and the muon’s $g-2$.Theoretical works on the corrections to the contributions of the anomaly to $\gamma \pi \rightarrow 2\pi$ have been addressed in ChPT to higher orders in [600–602], and in the vector meson dominance model [603]. $F_{3\pi }$ has also been calculated from the pion’s Bethe–Salpeter amplitude, see [604] and references therein. In these and related studies three key constraints are met: the quark propagator and the pion amplitude respect the axial-vector Ward identity, the full quark–photon vertex fulfills an electromagnetic Ward identity, and a complete set of ladder diagrams beyond the impulse approximation are taken into account. The three conditions are necessary to reproduce the low-energy theorem for the anomalous form factor. Results at large momentum and nonvanishing pion mass agree with the limited data and exhibit the same resonance behavior as the phenomenological vector meson dominance model. The latter property signals that a dynamically calculated quark–photon vertex contains the $\rho$ meson pole, and that in the relevant kinematical regions the vector mesons are the key physical ingredient in this QCD-based calculation. It seems that the time for an accurate test of $\gamma \pi \rightarrow 2\pi$ has arrived. A recent additional test of the box anomaly contributions is the decay $\eta \rightarrow \pi ^+\pi ^{-}\gamma$, which is currently being investigated in measurements at COSY (WASA) [605].

Another test of ChPT is provided by the measurement at COMPASS of $\pi ^{-} \gamma \rightarrow \pi ^{-}\pi ^{-}\pi ^+$ at $\sqrt{s}\le 5 M_\pi$ with an uncertainty in the cross section of 20 %. The results have been published in [606], along with a discussion of the good agreement with the leading-order ChPT result [607].

#### Hard pion ChPT

ChPT also describes situations in which pions are emitted by heavy mesons ($K$, $D$, $B$, etc.). In such decays, there are regions of phase space where the pion is hard and where chiral counting rules can no longer be applied. It has been, however, argued that chiral logarithms, calculated in regions with soft pions, might still survive in hard pion regimes and therefore might enlarge, under certain conditions, the domain of validity of the ChPT analysis [608, 609]. This line of approach has been called “Hard pion ChPT” and assumes that the chiral logarithms factorize with respect to the energy dependence in the chiral limit. Such factorization properties have been carefully analyzed in [610] using dispersion relations and explicitly shown to be violated for pion form factors by the inelastic contributions, starting at three loops. The study in [610] is presently being extended to heavy-light form factors. This will help clarify to what degree of approximation and in what regions of phase space hard pion ChPT might have practical applications in the analysis of heavy meson decays.

#### Baryon chiral dynamics

Baryon chiral dynamics still represents a challenge, but very exciting progress is being made on three fronts: experimental, theoretical, and lattice QCD. Here we highlight some of them.

Combining data from pionic hydrogen and deuterium [611, 612], the $\pi N$ scattering lengths have been extracted with the so-called Deser formula [538, 539], leading to [613]: $m_{\pi }a_0^{-}=(86.1\pm 0.1)\times 10^{-3}$ and $m_{\pi }a_0^+=(7.6\pm 3.1)\times 10^{-3}$, to be compared with the leading-order predictions [614]: $m_{\pi }a_0^{-}\simeq 80\times 10^{-3}$ and $m_{\pi }a_0^+=0$. ($a_0^+$ and $a_0^{-}$ are the $S$-wave isospin even and isospin odd scattering lengths, respectively. They are related to the isospin $1/2$ and $3/2$ scattering lengths through the formulas $a_0^+=(a_0^{1/2}+2a_0^{3/2})/3$ and $a_0^{-}=(a_0^{1/2}-a_0^{3/2})/3$.) In the same spirit, kaon–nucleon scattering lengths have been extracted from the combined data coming from kaonic hydrogen X-ray emissions [615] and kaon deuterium scattering [616]. The latter analysis uses data coming from the recent SIDDHARTA experiment at the DA$\Phi$NE electron–positron collider [617].

In spite of existing huge data sets on pion–nucleon scattering, the low-energy scattering amplitudes are still not known with great precision. And yet this is the region in which low-energy theorems and ChPT predictions exist. To remedy this deficiency, a systematic construction of $\pi N$ scattering amplitudes has been undertaken in [618] using the Roy–Steiner equations, based on a partial wave decomposition, crossing symmetry, analyticity, and dispersion relations. This approach parallels the one undertaken for $\pi K$ scattering [548], although in the present case the spin degrees of freedom of the nucleon considerably increase the number of Lorentz invariant amplitudes. It is hoped that a self-consistent iterative procedure between solutions obtained in different channels will yield a precise description of the low-energy $\pi N$ scattering amplitude.

Another long-standing problem in $\pi N$ physics is the evaluation of the pion–nucleon sigma term. In general, sigma terms are defined as forward matrix elements of quark mass operators between single hadronic states and are denoted, with appropriate indices, by $\sigma$. More generally, the sigma terms are related to the scalar form factors of the hadrons, denoted by $\sigma (t)$, where $t$ is the momentum transfer squared, with $\sigma (0)$ corresponding to the conventional sigma term. The interest in the sigma terms resides in their property of being related to the mass spectrum of the hadrons and to the scattering amplitudes through Ward identities. Concerning the pion–nucleon sigma term, in spite of an existing low-energy theorem [619], its full evaluation necessitates an extrapolation of the low-energy $\pi N$ scattering amplitude to an unphysical region [620]. The result depends crucially on the way the data are analyzed. Several contradictory results have been obtained in the past, and this has given rise to much debate. Recent evaluations of the sigma term continue to raise the same questions. In [621], a relatively large value of the sigma term is found, $\sigma =(59\pm 7)$ MeV, while in [622], the relatively low value of [620] is confirmed, $\sigma =(43.1\pm 12.0)$ MeV; the two evaluations remain, however, marginally compatible. One application of the equations of [618] concerns a dispersive analysis of the scalar form factor of the nucleon [623]. This has allowed the evaluation of the correction $\Delta _{\sigma }=\sigma (2m_{\pi }^2)-\sigma (0)$ of the scalar form factor of the nucleon, needed for the extraction of the $\pi N$ sigma term from $\pi N$ scattering. Using updated phase shift inputs, the value $\Delta _{\sigma }=(15.2\pm 0.4)$ MeV has been found, confirming the earlier estimate of [624].

A complementary access to the sigma term is becoming possible thanks to lattice-QCD calculations of the nucleon mass at varying values of the quark masses [625]. The current limitations reside in the relatively large quark masses used, and also in the still significant error bars from calculations which employ the lowest possible quark masses. It is however feasible that in the near future results competitive in accuracy to the ones obtained from $\pi N$ analyses will be available from lattice QCD.

One issue that has been open for a long time is the precise value of the $g_{\pi NN}$ coupling. A new extraction by an analysis in [626] based on the Gell-Mann–Oakes–Renner (GMO) sum rule gives $g_{\pi NN}^2/(4\pi )=13.69(12)(15)$. This value agrees with those of analyses favoring smaller values of the coupling. It, in particular, supports the argument based on the naturalness of the Goldberger–Treiman discrepancy when extended to $\mathrm{SU}(3)$ [627].

A theoretical development in BChPT which has been taking place over many years is the development of effective theories with explicit spin 3/2 baryons degrees of freedom. It has been known for a long time [628] that the inclusion of the spin 3/2 decuplet improved the convergence of the chiral expansion for certain key quantities. The theoretical foundation for it is found in the $1/N_\mathrm{c}$ expansion [629, 630], the key player being the (contracted) spin-flavor symmetry of baryons in large $N_\mathrm{c}$. This has led to formulating BChPT in conjunction with the $1/N_\mathrm{c}$ expansion [631–634], a framework which has yet to be applied extensively but which has already shown its advantages. Evidence of this is provided by several works on baryon semileptonic decays [632, 633, 635], and in particular in the analysis of the nucleon’s axial coupling [634], where the cancellations between the contributions from the nucleon and $\varDelta$ to one-loop chiral corrections are crucial for describing the near independence of $g_A$ with respect to the quark masses as obtained from lattice-QCD calculations [206, 236, 237, 256, 259, 261]. We expect that many further applications of the BChPT$\otimes 1/N_\mathrm{c}$ framework will take place in the near future, and it will be interesting to see what its impact becomes in some of the most difficult problems such as baryon polarizabilities, spin-polarizabilities, $\pi N$ scattering, etc. Further afield, and addressed elsewhere in this review, are the applications to few-nucleon effective theories, of which the effective theory in the one-nucleon sector is a part. An interesting recent development in baryon lattice QCD is the calculation of masses at varying $N_\mathrm{c}$ [636]. Although at this point the calculations are limited to quenched QCD, they represent a new tool for understanding the validity of $N_\mathrm{c}$ counting arguments in the real world, which will be further improved by calculations in full QCD and at lower quark masses. For an analysis of the results in [636] in the light of BChPT$\otimes 1/N_\mathrm{c}$ framework, see [637].

A new direction worth mentioning is the application of BChPT to the study of the nucleon partonic structure at large transverse distances [638], which offers an example of the possible applications of effective theories to the soft structures accompanying hard processes in QCD.

#### Other topics

Many other subjects are in the domains of interest and expertise of ChPT and are being studied actively. We merely quote some of them: pion and eta photoproduction off protons [639–644], pion polarizabilities [332, 333] (see also Sect. 3.2.7c) and two-pion production in $\gamma \gamma$ collisions [645], the decay $\eta '\rightarrow \eta \pi \pi$ [646], the electromagnetic rare decays $\eta '\rightarrow \pi ^0\gamma \gamma$ and $\eta '\rightarrow \eta \gamma \gamma$ [647], $K$ meson rare decays [648, 649], hadronic light-by-light scattering [650], etc. The incorporation of the $\eta '$ meson into the ChPT calculations is usually done in association with the $1/N_\mathrm{c}$ expansion [651], since for finite $N_\mathrm{c}$, the $\eta '$ is not a Goldstone boson in the chiral limit.

The above processes enlarge the field of investigation of ChPT, by allowing for the determination of new LECs and tests of nontrivial predictions. Some of the amplitudes of these processes do not receive contributions at tree level and have as leading terms $O(p^4)$ or $O(p^6)$ loop contributions. Therefore they offer more sensitive tests of higher-order terms of the chiral expansion.

An important area of applications of ChPT is to weak decays, which unfortunately cannot be covered in this succinct review. Of particular current interest are the inputs to nonleptonic kaon decays, where lattice-QCD calculations have been steadily progressing and are making headway in understanding old, difficult problems such as the $|\Delta I|=1/2$ rule [652]. We refer to [44] for a review of the current status of kaon nonleptonic decays vis-à-vis lattice QCD. Many topics in baryon physics have also not been touched upon, among them the study of low-energy aspects of the EM properties of baryons such as the study of polarizabilities, in particular, the spin polarizabilities and generalized polarizabilities as studied with electron scattering [653, 654].

#### Outlook and remarks

As a low-energy effective field theory of QCD, ChPT offers a solid and reliable framework for a systematic evaluation of various dynamical contributions, where the unknown parts are encoded within a certain number of low-energy constants (LECs). Two-flavor ChPT is well established, founded on a firm ground. The main challenge now concerns the convergence properties of three-flavor ChPT, where a definite progress in our understanding of the role of the strange quark is still missing. Efforts are being continued in this domain, and it is hoped that new results coming from lattice-QCD calculations will help clarify the situation. Another specific challenge concerns the understanding of isospin breaking, including the evaluation of electromagnetic effects, in the decay $\eta \rightarrow 3\pi$. New domains of interest, such as the probe of hard-pion regions in heavy-particle decays, $\eta '$ physics, and rare kaon decays, are being explored. This, together with data provided by high-precision experimental projects, gives confidence in the progress that should be accomplished in the near future.

In baryons, the present progress in lattice QCD is leading to an important understanding of the behavior of the chiral expansion thanks to the possibility of studying the quark-mass dependence of key observables. Although issues remain, such as the problem in confronting with the empirical value of $g_A$, it is clear that lattice QCD will have a fundamental impact in our understanding of the chiral expansion in baryons. Further, the union of BChPT and the $1/N_\mathrm{c}$ expansion represents a very promising framework for further advances in the low-energy effective theory for baryons.

### Low-energy precision observables and tests of the Standard Model

#### The muon’s anomalous magnetic moment

The muon’s anomalous magnetic moment, $a_{\mu }=(g-2)_{\mu }/2$, is one of the most precisely measured quantities in particle physics, reaching a precision of 0.54 ppm. The most recent experimental measurement, BNL 821 [655], is

3.34 $$\begin{aligned} 10^{10}a_{\mu } = 11\,659\,208.9(6.3). \end{aligned}$$

This result should be compared with the theoretical calculation within the Standard Model (a topic worthy of a review in itself):3.35using the compilation in [657]. Here the leading-order (LO) hadronic vacuum polarization is taken from measurements of $R(e^+e^{-}\rightarrow \text {hadrons})$, and the electroweak (EW) corrections have been adjusted slightly to account for the (since measured) Higgs mass $M_\mathrm{H}=125~\text {GeV}$. While QED and electroweak contributions account for more than 99.9999 % of the value $a_{\mu }$, the dominant errors in (3.35) stem from the hadronic vacuum polarization (HVP) and hadronic light-by-light (HLbL) scattering—they stem from QCD.

The difference between the values in (3.34) and (3.35) is $28.5\pm 6.3_\text {expt}\pm 4.9_\text {SM}$, which is both large—larger than the EW contributions $19.5-3.9=15.6$—and significant—around $3.5\sigma$. This deviation has persisted for many years and, if corroborated, would provide a strong hint for physics beyond the Standard Model. This situation has motivated two new experiments with a target precision of 0.14 ppm, FNAL 989 [658], and J-PARC P34 [659]. The new experiments have, in turn, triggered novel theoretical efforts with the objective to obtain a substantial improvement of the theoretical values of the QCD corrections to the muon anomaly. In this section, we address HVP and HLbL in turn, discussing approaches (such as $R(e^+e^{-})$) involving other experiments, lattice QCD, and for HLbL also models of QCD.

The principal phenomenological approach to computing the HVP contribution $a_{\mu }^\mathrm{had;VP}$ is based on the optical theorem and proceeds by evaluating a dispersion integral, using the experimentally measured cross section for $e^{+}e^{-}\rightarrow \mathrm hadrons$. Evaluations of various authors use the same data sets and basically agree, differing slightly in the computational methods and final uncertainties deslightly pending on the conservatism of the authors [660–662]. Note that recent measurements of $\sigma (e^+e^{-} \rightarrow \pi ^+\pi ^{-})$, the process dominating the LO HVP contribution, performed using initial-state radiation at BaBar [663] and KLOE [664, 665] do not show complete agreement with each other and with the previous measurements based on direct scans [666, 667]. Determination of the cross section in all these experiments, in particular those using initial-state radiation, crucially depends on the rather complicated radiative corrections.

An alternative phenomenological approach is to use $\tau$ decay to hadrons to estimate the HVP. This approach is very sensitive to the way isospin-breaking corrections are evaluated. While a model-dependent method trying to take into account various effects due to $m_d \ne m_u$ still shows notable deviation from the $e^+e^{-}$ based estimate [668], the authors of [661] claim that after correcting the $\tau$ data for the missing $\rho$–$\omega$ mixing contribution, in addition to the other known isospin-symmetry-violating corrections, $e^+e^{-}$ and $\tau$-based calculations give fully compatible results.

**Fig. 27:**
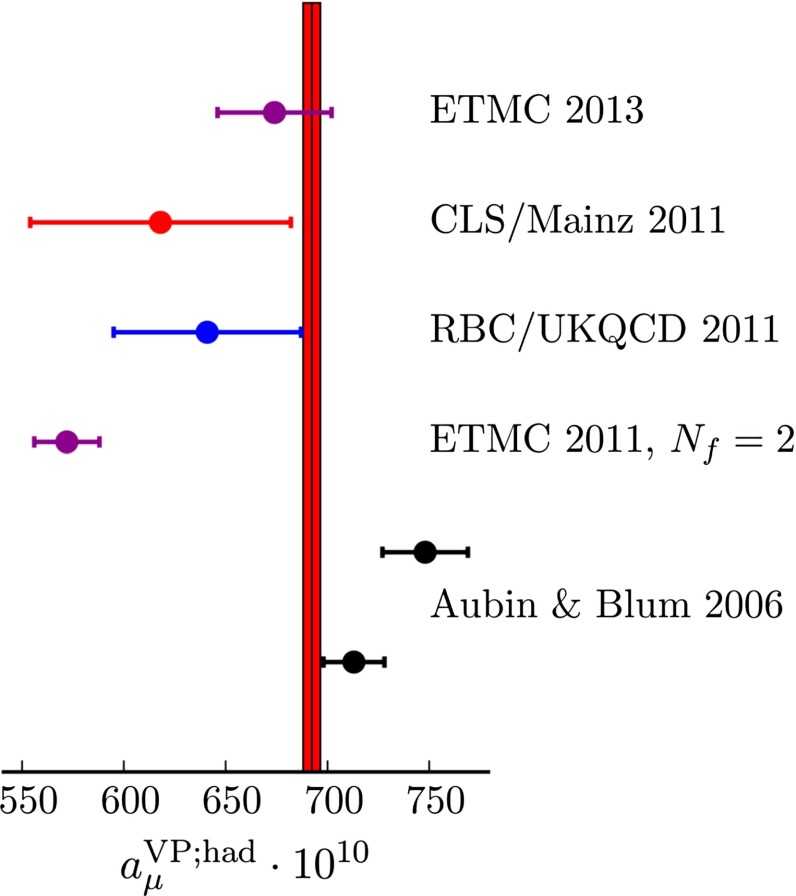
Compilation of recently published lattice QCD results for the leading hadronic vacuum polarization contribution to the muon’s anomalous magnetic moment. Displayed is $10^{10}a_{\mu }^\mathrm{VP;had}$, from ETM [672, 676], CLS/Mainz [674], RBC/UKQCD [673] and Aubin et al. [671]. The position and width of the *red vertical line* denote the phenomenological result from dispersion theory and its uncertainty, respectively

To complement the phenomenological approach, it is desirable to determine the contributions due to HVP from first principles. Lattice QCD is usually restricted to space-like momenta, and in [669, 670] it was shown that $a_{\mu }^\mathrm{had;VP}$ can be expressed in terms of a convolution integral, i.e.,

3.36 $$\begin{aligned} a_{\mu }^\mathrm{VP;had} = 4\pi ^2\left( \frac{\alpha }{\pi }\right) ^2 \int _0^\infty \mathrm{d}Q^2\, f(Q^2)\left\{ \Pi (Q^2)-\Pi (0) \right\} , \end{aligned}$$

where the vacuum polarization amplitude, $\Pi (Q^2)$, is determined by computing the correlation function of the vector current. Recent calculations based on this approach appeared in [671–677], and a compilation of published results is shown in Fig. 27.

The evaluation of the correlation function of the electromagnetic current involves quark-disconnected diagrams, which are also encountered in isoscalar form factors of the nucleon discussed earlier in this section. Given that a statistically precise evaluation is very costly, such contributions have been largely neglected so far. Another major difficulty arises from the fact that the known convolution function $f(Q^2)$ in (3.36) is peaked at momenta around the muon mass, which is a lot smaller than the typical nonzero momentum that can be achieved on current lattices. Therefore, it appears that lattice estimates of $a_{\mu }^\mathrm{VP;had}$ are afflicted with considerable systematic uncertainties related to the low-$Q^2$ region. In [674] it was therefore proposed to apply partially twisted boundary conditions [678, 679] to compute the quark-connected part of the correlator. In this way, it is possible to obtain a very high density of data points, which penetrate the region where the convolution integral receives its dominant contribution.

Recently, there have been proposals which are designed to overcome this problem. In [680, 681] the subtracted vacuum polarization amplitude, $\Pi (Q^2)-\Pi (0)$, is expressed as an integral of a partially summed vector-vector correlator, which is easily evaluated on the lattice for any given value of the $Q^2$. Furthermore, a method designed to compute the additive renormalization $\Pi (0)$ directly, i.e., without the need for an extrapolation to vanishing $Q^2$, has been proposed [682].

The compilation of recent lattice results for the leading hadronic vacuum polarization contributions and their comparison to the standard dispersive approach in Fig. 27 shows that the accuracy of current lattice estimates of $a_{\mu }^\mathrm{{VP;had}}$ is not yet competitive. In particular, statistical uncertainties will have to be considerably reduced before lattice results can challenge the accuracy of dispersion theory. One step in this direction has been taken in [683], which advocates the use of efficient noise reduction techniques, dubbed “all-mode-averaging”. Other recent activities include the study of the systematic effects related to the use of twisted boundary conditions [684] and the Ansatz used to extrapolate $\Pi (Q^2)$ to vanishing $Q^2$ [685].

For HLbL, a direct experimental determination analogous to those discussed for HVP is not directly available. HLbL enters in $\mathcal{O}(\alpha ^3)$, just as the NLO HVP does. The latter, however, is assessed in a dispersion relation framework [686], similar to that of the LO piece—the piece associated with the electromagnetic dressing of the HVP is part of the final-state radiative correction to the LO HVP term [687]. As for the HLbL term, it must be calculated; we refer to [656, 688] for reviews. The diagrammatic contributions to it can be organized in a simultaneous expansion in momentum $p$ and number of colors $N_\mathrm{c}$ [689]; the leading contribution in $N_\mathrm{c}$ is a $\pi ^0$ exchange graph. The computation of HLbL requires integration over three of the four photon momenta. Detailed analysis reveals that the bulk of the integral does not come from small, virtual momenta, making ChPT of little use. Consequently heavier meson exchanges should be included as well; this makes the uncertainties in the HLbL computation more challenging to assess. We have reported the HLbL result determined by the consensus of different groups [656]. Recently there has been discussion of the charged-pion loop graph (which enters as a subleading effect) in chiral perturbation theory, arguing that existing model calculations of HLbL are inconsistent with the low-energy structure of QCD [650]. Including the omitted low-energy constants in the usual framework does modify the HLbL prediction at the 10 % level [690]. The upshot is that the uncertainties can be better controlled through measurement of the pion polarizability (or generally of processes involving a $\pi ^+\pi ^{-} \gamma ^*\gamma ^*$ vertex such as $e^{+} e^{-} \rightarrow e^{+} e^{-} \pi ^+\pi ^0$), which is possible at JLab [691]. As long recognized, data on $\pi ^0\rightarrow \gamma \gamma ^*$, $\pi ^0\rightarrow \gamma ^*\gamma ^*$, as well as $\pi ^0 \rightarrow e^{+} e^{-} (\gamma )$, should also help in constraining the primary $\pi ^0$ exchange contribution. Recently, a dispersive framework for the analysis of the $\pi$ and $2\pi$ intermediate states (and generalizable to other mesons) to HLbL has been developed [692]; we are hopeful in regards to its future prospects.

Unfortunately, lattice-QCD calculations of HLbL are still at a very early stage. A survey of recent ideas with a status report is given in [693]. Here, we comment briefly on only two approaches: the extended Nambu–Jona–Lasinio (ENJL) model (see, e.g., [694]) and a functional approach based on calculations of Landau-gauge-QCD Green’s functions (see, e.g., [695, 696]). The latter is based on a model interaction (cf. the remarks on the Faddeev approach to nucleon observables in Sect. 3.2.5b). However, such a calculation based on input determined from first-principle calculations would be highly desirable.

In the ENJL model one has a nonrenormalizable contact interaction, and consequently a momentum-independent quark mass and no quark wave-function renormalization. The quark–photon vertex is modeled as a sum of the tree-level term and a purely transverse term containing the vector meson pole. On the other hand, the Green function approach is based on an interaction according to the ultraviolet behavior of QCD and is therefore renormalizable. The resulting quark propagator is characterized by a momentum-dependent quark mass and a momentum-dependent quark wave-function renormalization. The quark–photon vertex is consistently calculated and contains a dynamical vector meson pole. Although the different momentum dependencies cancel each other partly (which is understandable when considering the related Ward identities), remarkable differences in these calculations remain. Based on a detailed comparison the authors of [695] argue that the suppression of the quark-loop reported in the ENJL model is an artifact of the momentum-independent quark mass and the momentum restriction within the quark–photon vertex, which, in turn, are natural consequences of the contact interaction employed there. Regardless of whether one concludes from these arguments that the standard value for the hadronic light-by-light scattering contribution may be too small, one almost inevitably needs to conclude that the given comparison provides evidence that the systematic error attributed to the ENJL calculation is largely underestimated.

As it is obvious that an increased theoretical error leads to a different conclusion on the size of the discrepancy of the value for $a_{\mu }$ between the theoretical and experimental values, an increased effort on the QCD theory side is needed. One important aspect of future lattice calculations of the hadronic light-by-light contribution is to employ them in a complementary way together with other methods. For instance, an identification of the relevant kinematics of the hadronic contribution to the photon four-point function through the cross-fertilization of different approaches might already pave the way for much more accurate computations. The forthcoming direct measurement of $a_{\mu }$ at FNAL is expected to reduce the overall error by a factor of five. Therefore, a significant improvement of the theoretical uncertainty for the hadronic light-by-light scattering contribution down to the level of 10 % is required. Hereby the systematic comparison of different approaches such as effective models, functional methods, and lattice gauge theory may be needed to achieve this goal.

#### The electroweak mixing angle

The observed deviation between direct measurements and theoretical predictions of the muon anomalous magnetic moment—if corroborated in the future—may be taken as a strong hint for physics beyond the Standard Model. Another quantity which provides a stringent test of the Standard Model is the electroweak mixing angle, $\sin ^2\theta _W$. There is, however, a three-sigma difference between the most precise experimental determinations of $\sin ^2\theta _W(M_Z)_\mathrm{\overline{MS}}$ at SLD [697], measuring the left-right asymmetry in polarized $e^{+}e^{-}$ annihilation, and LEP [698], which is based on the forward-backward asymmetry in $Z\rightarrow b\bar{b}$. The origin of the tension between these two results has never been resolved. While an existing measurement at the Tevatron [699, 700] is not accurate enough to decide the issue, it will be interesting to see whether the LHC experiments can improve the situation.

The value of $\sin ^2\theta _W$ can be translated into a value of the Higgs mass, given several other SM parameters as input, including the strong coupling constant $\alpha _\mathrm{s}$, the running of the fine-structure constant $\Delta \alpha$, and the mass of the top quark, $m_t$. The two conflicting measurements at the $Z$-pole lead to very different predictions for the Higgs mass $m_\mathrm{H}$ [701], which can be confronted with the direct Higgs mass measurement at the LHC. In order to decide whether any observed discrepancy could be a signal for physics beyond the Standard Model, further experimental efforts to pin down the value of $\sin ^2\theta _W(M_Z)_\mathrm{\overline{MS}}$ are required.

**Fig. 28:**
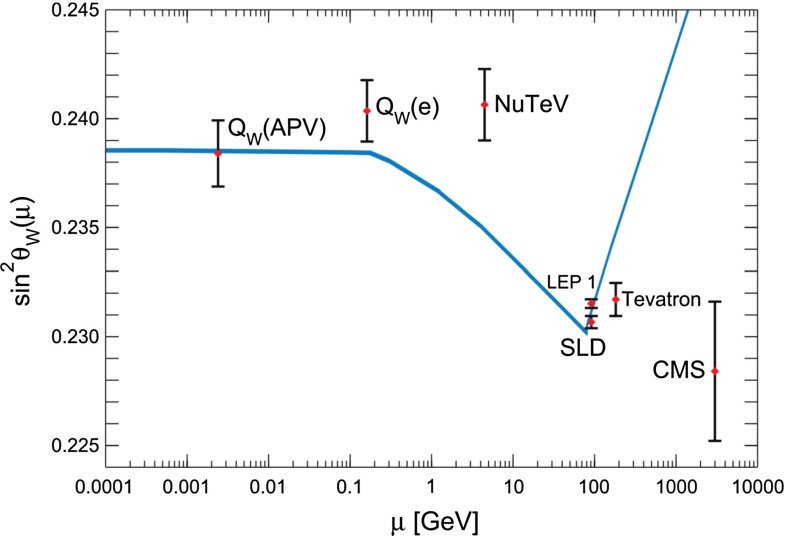
The scale dependence of the electroweak mixing angle in the $\mathrm \overline{MS}$ scheme. The *blue band* is the theoretical prediction, while its width denotes the theoretical uncertainty from strong interaction effects. From [1]

In addition to the activities at high-energy colliders, there are also new (QWEAK [702]) and planned experiments (MOLLER [703], P2@MESA), designed to measure the electroweak mixing angle with high precision at low energies, by measuring the weak charge of the proton. These efforts extend earlier measurements of the parity-violating asymmetry in Møller scattering [704] and complement other low-energy determinations, based on atomic parity violation (APV) and neutrino-DIS (NuTeV). The collection of measurements across the entire accessible energy range can be used to test whether the running of $\sin ^2\theta _W$ is correctly predicted by the SM, i.e., by checking that the different determinations can be consistently translated into a common value at the $Z$-pole. The current status is depicted in Fig. 28.

We will now discuss the particular importance of low-energy hadronic determinations of the electroweak mixing angle, and the role of new experiments (for an in-depth treatment, see [705]). These are based on measuring the weak charge of the proton, $Q_W^p$, which is accessible by measuring the helicity-dependent cross section in polarized $ep$ scattering. For a precise determination of the electroweak mixing angle, one must augment the tree-level relation $Q_W^p=1-4\sin ^2\theta _W$ by radiative corrections [706]. It then turns out that the dominant theoretical uncertainty is associated with hadronic effects, whose evaluation involves some degree of modeling [707]. Radiative corrections arising from ${\gamma }Z$ box graphs play a particularly important role, and their contributions have been evaluated in [708–712]. An important feature is that they are strongly suppressed at low energies. It is therefore advantageous to measure the weak charge in low-energy $ep$ scattering, since the dominant theoretical uncertainties in the relation between $Q_W^p$ and $\sin ^2\theta _W$ are suppressed.

#### $\alpha _\mathrm{s}$ from inclusive hadronic $\tau$ decay

As remarked several times in this review, the precise determination of $\alpha _\mathrm{s}$ at different scales, and hereby especially the impressive agreement between experimental determinations and theoretical predictions, provides an important test of asymptotic freedom and plays a significant role in establishing QCD as the correct fundamental theory of the Strong Interaction.

Hadronic $\tau$ decays allow for a determination of $\alpha _\mathrm{s}$ at quite low momentum scales [713]. The decisive experimental observable is the inclusive ratio of $\tau$ decay widths,

3.37 $$\begin{aligned} R_\tau \equiv \frac{\Gamma [\tau ^{-} \rightarrow \nu _\tau {\mathrm {hadrons} \, (\gamma )]}}{\Gamma [\tau ^{-} \rightarrow \nu _\tau e^{-}\bar{\nu }_e (\gamma )]} , \end{aligned}$$

which can be rigorously analyzed with the short-distance operator product expansion.

Since non-perturbative corrections are heavily suppressed by six powers of the $\tau$ mass, the theoretical prediction is dominated by the perturbative contribution, which is already known to $O(\alpha _\mathrm{s}^4)$ and amounts to a 20 % increase of the naive parton-model result $R_\tau = N_C = 3$. Thus, $R_\tau$ turns out to be very sensitive to the value of the strong coupling at the $\tau$ mass scale; see, e.g., [714] and references therein.

From the current $\tau$ decay data, one obtains [714]

3.38 $$\begin{aligned} \alpha _\mathrm{s}(m_\tau ^2) = 0.331\pm 0.013. \end{aligned}$$

The recent Belle measurement of the $\tau$ lifetime [715] has slightly increased the central value by $+0.002$, with respect to the previous result [716]. After evolution to the scale $M_Z$, the strong coupling decreases to

3.39 $$\begin{aligned} \alpha _\mathrm{s}(M_Z^2) = 0.1200\pm 0.0015, \end{aligned}$$

in excellent agreement with the direct measurement at the $Z$ peak, $\alpha _\mathrm{s}(M_Z^2) = 0.1197\pm 0.0028$ [1]. Owing to the QCD running, the error on $\alpha _\mathrm{s}$ decreases by one order of magnitude from $\mu = m_\tau$ to $\mu =M_Z$.

The largest source of uncertainty has a purely perturbative origin. The $R_\tau$ calculation involves a closed contour integration in the complex $s$-plane, along the circle $|s| = m_\tau ^2$. The long running of $\alpha _\mathrm{s}(-s)$ generates powers of large logarithms, $\log {(-s/m_\tau ^2)}=i \phi$, $\phi \in [-\pi ,\pi ]$, which need to be resummed using the renormalization group. One gets in this way an improved perturbative series, known as contour-improved perturbation theory (CIPT) [717], which shows quite good convergence properties and a mild dependence on the renormalization scale. A naive expansion in powers of $\alpha _\mathrm{s}(m_\tau ^2)$ (fixed-order perturbation theory, FOPT), without resumming those large logarithms, gives instead a badly-behaved series which suffers from a large renormalization-scale dependence. A careful study of the contour integral shows that, even at $O(\alpha _\mathrm{s}^4)$, FOPT overestimates the total perturbative correction by about 11 %; therefore, it leads to a smaller fitted value for $\alpha _\mathrm{s}$. Using CIPT one obtains $\alpha _\mathrm{s}(m_\tau ^2) = 0.341\pm 0.013$, while FOPT results in $\alpha _\mathrm{s}(m_\tau ^2) = 0.319\pm 0.014$ [714].

The asymptotic nature of the perturbative QCD series has been argued to play an important role even at low orders in the coupling expansion. Assuming that the fourth-order series is already governed by the lowest ultraviolet and infrared renormalons, and fitting the known expansion coefficients to ad-hoc renormalon models, one predicts a positive correction from the unknown higher orders, which results in a total perturbative contribution to $R_\tau$ close to the naive FOPT result [718]. This conclusion is however model dependent [714]. In the absence of a better understanding of higher-order corrections, the CIPT and FOPT determinations have been averaged in (3.38), but keeping the larger error.

A precise extraction of $\alpha _\mathrm{s}$ at such low scale necessitates also a thorough understanding of the small non-perturbative condensate contributions to $R_\tau$. Fortunately, the numerical size of non-perturbative effects can be determined from the measured invariant-mass distribution of the final hadrons in $\tau$ decay [719]. With good data, one could also analyze the possible role of corrections beyond the operator product expansion. The latter are called duality violations (because they signal the breakdown of quark-hadron duality underlying the operator product expansion), and there is (as yet) no first-principle theoretical description available. These effects are negligible for $R_\tau$, because the operator-product-expansion uncertainties near the real axis are kinematically suppressed in the relevant contour integral; however, they could be more relevant for other moments of the hadronic distribution.

The presently most complete and precise experimental analysis, performed with the ALEPH data, obtains a total non-perturbative correction to $R_\tau$, $\delta _{\mathrm {NP}} = -(0.59\pm 0.14)~\%$ [720], in good agreement with the theoretical expectations and previous experimental determinations by ALEPH, CLEO, and OPAL [714]. This correction has been taken into account in the $\alpha _\mathrm{s}$ determination in (3.38). A more recent fit to rescaled OPAL data (adjusted to reflect current values of exclusive hadronic $\tau$-decay branching ratios), with moments chosen to maximize duality violations, finds $\delta _{\mathrm {NP}} = -(0.3\pm 1.2)~\%$ [721], in agreement with the ALEPH result but less precise because of the much larger errors of the OPAL data.

A substantial improvement of the $\alpha _\mathrm{s}(m_\tau ^2)$ determination requires more accurate $\tau$ spectral-function data, which should be available in the near future, and a better theoretical control of higher-order perturbative contributions, i.e., an improved understanding of the asymptotic nature of the QCD perturbative series.

Experimental knowledge on $\alpha _\mathrm{s}$ at even lower scales ($s\!<\!m^2_\tau$), at the borderline of the perturbative to non-perturbative regime of QCD, could profit from lattice simulations of appropriately chosen observables. Last but not least, it should be noted that in the non-perturbative domain, i.e., at scales below 1 GeV, an unambiguous definition of the strong coupling is missing; for a corresponding discussion see, e.g., [722].

### Future directions

In a broad sense, the physics of light quarks remains a key for understanding strong QCD dynamics, from its more fundamental non-perturbative effects to the varied dynamical effects which manifest themselves in the different properties of hadrons. Recent progress in the theoretical and experimental fronts has been remarkable.

Numerous experimental results keep flowing from different facilities employing hadron (J-PARC, COSY, COMPASS, VES) or electron beams (CLAS, MAMI-C, ELSA, SPring-8, CLEO-c, BESIII, KLOE-2, and CMD-3 and SND at VEPP-2000). The experiments aim at investigating the full hadron spectrum, searching, e.g., for exotic and hybrid mesons or missing baryon resonances, as well as at determining dynamical properties of those excited states such as helicity amplitudes and form factors. New facilities are planned to come into operation in the next few years, which are expected to deliver data with extremely high statistical accuracy. The upcoming 12 GeV upgrade of JLab with the new Hall D is one of the key new additions to that line of research. Also the upgraded CLAS12 detector at JLab is expected to contribute to hadron spectroscopy. Hadronic decays of heavy-quark states produced at future $e^+e^{-}$ (Belle II) or $p\bar{p}$ machines (PANDA) will serve as abundant source of light-quark states with clearly defined initial states. In addition, the particularly clean access to light hadron states via direct production in $e^+e^{-}$ annihilation with initial-state radiation, as well as via $\gamma \gamma$ fusion, is possible at Belle II. The anticipated data from these next-generation experiments should, in principle, allow us to clarify the existence and nature of hadronic resonances beyond the quark model and to determine resonance parameters reliably for states where this has not been possible in the past because of pole positions far in the complex plane, overlapping resonances, or weak couplings to experimentally accessible channels. A model- and reaction-independent characterization of resonance parameters in terms of pole positions and residues, however, also requires advances on the analysis side to develop models which respect the theoretical constraints of unitarity and analyticity.

Experiments on the ground-state mesons and baryons will continue at the intermediate- and high-energy facilities, which can have an impact in and beyond QCD. Examples include the elucidation of the spin structure of the nucleon at the partonic level, which is one of the motivations for the work currently underway on the design of an Electron Ion Collider, precision photo-production on the nucleon and of light mesons, and experiments that impact the Standard Model, such as those necessary for improving the calculation of the hadronic contributions to the muon’s anomalous magnetic moment and the measurements of the weak charge of the nucleon, which impacts the knowledge of the EW angle at lower energies. Naturally, most of the topics discussed in this review are part of the broad experimental programs in place today and planned for the near future.

On the theoretical front, LQCD is opening new perspectives. Full QCD calculations with light quark masses nearing the physical limit are becoming standard. This is allowing for unprecedented insights into the quark mass dependencies of meson and baryon observables, which especially influence the determination of numerous LECs in EFT which are poorly known from phenomenology, and also in the knowledge of form factors and moments of structure functions. The study of excited light hadrons in LQCD is one of the most important developments in recent years, with the promise of illuminating the present rather sparse knowledge of those excited states, as well as possibly leading to the “discovery” of new states which are of difficult experimental access. It is clear that the progress in LQCD will continue, turning it into a key tool for exploration and discovery, as well as a precision tool for light quark physics.

Progress also continues with analytic methods, in particular with methods rooted in QCD, such as Schwinger-Dyson equations, ChPT, dispersion theory combined with ChPT, SCET, various approaches in perturbative QCD, $1/N_\mathrm{c}$ expansion, AdS/QCD, etc. Most analytic methods rely on experimental and/or lattice QCD information, which is currently fueling theoretical progress thanks to the abundance and quality of that information.

## Heavy quarks

7Heavy quarks have played a crucial role in the establishing and development of QCD in particular, and the Standard Model of particle physics in general. Experimentally this is related to a clean signature of many observables even in the presence of only few rare events, which allows the study of both new emergent phenomena in the realm of QCD and new physics beyond the Standard Model. Theoretically, the clean signature may be traced back to the fact that

4.1 $$\begin{aligned} m_Q \gg \Lambda _{\mathrm{QCD}}, \end{aligned}$$

which implies that processes happening at the scale of the heavy-quark mass $m_Q$ can be described by perturbative QCD and that non-perturbative effects, including the formation of background low-energy light hadrons, are suppressed by powers of $\Lambda _{\mathrm{QCD}}/m_Q$. The hierarchy (4.1) gets complicated by lower energy scales if more than one heavy quark is involved in the physical process, but the basic fact that high-energy physics at the scale $m_Q$ can be factorized from low-energy non-perturbative physics at the hadronic scale $\Lambda _{\mathrm{QCD}}$ is at the core of the dynamics of any system involving a heavy quark.

The hierarchy (4.1) is usually exploited to replace QCD with equivalent Effective Field Theories (EFTs) that make manifest at the Lagrangian level the factorization of the high-energy modes from the low-energy ones. Examples are the Heavy Quark Effective Theory (HQET) [723–726] suitable to describe systems made of one heavy quark, and EFTs like Non-relativistic QCD (NRQCD) [727, 728] or potential Non-relativistic QCD (pNRQCD) [729, 730], suitable to describe systems made of two or more heavy quarks. Non-relativistic EFTs [731] have been systematically used both in analytical and in numerical (lattice) calculations involving heavy quarks. Concerning lattice studies, nowadays the standard approach is to resort to EFTs when bottom quarks are involved, and to rely on full lattice QCD calculations when studying systems made of charm quarks.

The section aims at highlighting some of the most relevant progress made in the last few years in the heavy-quark sector of QCD both from the methodological and phenomenological point of view. There is no aim of completeness. It is organized in the following way. In Sect. 4.1 we discuss methodological novelties in the formulation of non-relativistic EFTs and in lattice QCD, whereas the following sections are devoted to more phenomenological aspects. These are divided in phenomenology of heavy-light mesons, discussed in Sect. 4.2 and in phenomenology of heavy quarkonia. In Sect. 4.3 we present recent progress in quarkonium spectroscopy with particular emphasis on the quarkonium-like states at and above the open flavor threshold. Section 4.4 provides an updated list of ${\alpha _{\mathrm{s}}}$ extractions from quarkonium observables. Section 4.5 summarizes our current understanding of quarkonium production. Finally, Sect. 4.6 outlines future directions.

### Methods

#### Non-relativistic effective field theories

The non-relativistic EFT of QCD suited to describe a heavy quark bound into a heavy-light meson is HQET [725, 732] (see [726] for an early review). Heavy-light mesons are characterized by only two energy scales: the heavy quark mass $m_Q$ and the hadronic scale $\Lambda _{\mathrm{QCD}}$. Hence the HQET Lagrangian is organized as an expansion in $1/m_Q$ and physical observables as an expansion in $\Lambda _{\mathrm{QCD}}/m_Q$ (and ${\alpha _{\mathrm{s}}}$ encoded in the Wilson coefficients). In the limit where $1/m_Q$ corrections are neglected, the HQET Lagrangian is independent of the flavor and spin of the heavy quark. This symmetry is called the heavy quark symmetry. Some of its phenomenological consequences for $B$ and $D$ decays will be discussed in Sect. 4.2.

In the case of two or more heavy quarks, the system is characterized by more energy scales. We will focus on systems made of a quark and an antiquark, i.e. quarkonia, although EFTs have been also developed for baryons made of three quarks [733–735]. For quarkonia, one has to consider at least the scale of the typical momentum transfer between the quarks, which is also proportional to the inverse of the typical distance, and the scale of the binding energy. In a non-relativistic bound state, the first goes parametrically like $m_Qv$ and the second like $m_Qv^2$, where $v$ is the velocity of the heavy quark in the center-of-mass frame. An EFT suited to describe heavy quarkonia at a scale lower than $m_Q$ but larger than $m_Qv$ and $\Lambda _{\mathrm{QCD}}$ is NRQCD [727, 728] (whose lattice version was formulated in [736, 737]). Also the NRQCD Lagrangian is organized as an expansion in $1/m_Q$ and physical observables as an expansion in $v$ (and ${\alpha _{\mathrm{s}}}$ encoded in the Wilson coefficients). In the heavy-quark bilinear sector the Lagrangian coincides with the one of HQET (see also [738]), but the Lagrangian contains also four-quark operators. These are necessary to describe heavy-quarkonium annihilation and production, which are processes happening at the scale $m_Q$. The NRQCD factorization for heavy quarkonium annihilation processes has long been rigorously proved [728], while this is not the case for heavy quarkonium production. Due to its relevance, we devote the entire Sect. 4.1.2 to the most recent progress towards a proof of factorization for heavy quarkonium production. The state of the art of our understanding of heavy quarkonium production in the framework of NRQCD is presented in Sect. 4.5.

The power counting of NRQCD is not unique because the low-energy matrix elements depend on more than one residual energy scale. These residual scales are $m_Qv$, $m_Q v^2$, $\Lambda _{\mathrm{QCD}}$ and possibly other lower energy scales. The ambiguity in the power counting is reduced and in some dynamical regimes solved by integrating out modes associated to the scale $m_Qv$ and by replacing NRQCD by pNRQCD, an EFT suited to describe quarkonium physics at the scale $m_Qv^2$ [729, 730]. The pNRQCD Lagrangian is organized as an expansion in $1/m_Q$, inherited from NRQCD, and an expansion in powers of the distance between the heavy quarks. This second expansion reflects the expansion in the scale $m_Qv^2$ relative to the scale $m_Qv$ specific to pNRQCD. Like in NRQCD, contributions to physical observables are counted in powers of $v$ (and ${\alpha _{\mathrm{s}}}$ encoded in the high-energy Wilson coefficients). The degrees of freedom of pNRQCD depend on the specific hierarchy between $m_Qv^2$ and $\Lambda _{\mathrm{QCD}}$ for the system under examination.

The charmonium ground state and the lowest bottomonium states may have a sufficiently small radius to satisfy the condition $m_Qv^2 \gtrsim \Lambda _{\mathrm{QCD}}$. If this is the case, the degrees of freedom of pNRQCD are quark–antiquark states and gluons. The system can be studied in perturbative QCD, non-perturbative contributions are small and in general one may expect precise theoretical determinations once potentially large logarithms have been resummed by solving renormalization group equations and renormalon-like singularities have been suitably subtracted. For early applications we refer to [739–744], for a dedicated review see [745]. As an example of the quality of these determinations, we mention the determination of the $\eta _b$ mass in [743]. This was precise and solid enough to challenge early experimental measurements, while being closer to the most recent ones. We will come back to this and other determinations in Sect. 4.3.

Excited bottomonium and charmonium states are likely strongly bound, which implies that $\Lambda _{\mathrm{QCD}}\gtrsim m_Qv^2$. The degrees of freedom of pNRQCD are colorless and made of color-singlet quark–antiquark and light quark states [746–750]. The potentials binding the quark and antiquark have a rigorous expression in terms of Wilson loops and can be determined by lattice QCD [751–754]. It is important to mention that lattice determinations of the potentials have been performed so far in the quenched approximation. Moreover, at order $1/m_Q^2$ not all the necessary potentials have been computed (the set is complete only for the spin-dependent potentials). This implies that the quarkonium dynamics in the strongly coupled regime is not yet exactly known beyond leading $1/m_Q$ corrections.

For states at or above the open flavor threshold, new degrees of freedom may become important (heavy-light mesons, tetraquarks, molecules, hadro-quarkonia, hybrids, glueballs,$\ldots$). These states can in principle be described in a very similar framework to the one discussed above for states below threshold [755–758]. However, a general theory does not exist so far and specific EFTs have been built to describe specific states (an example is the well-known $X(3872)$ [759–764]). This is the reason why many of our expectations for these states still rely on potential models.

In Sect. 4.3 we will discuss new results concerning the charmonium and bottomonium spectroscopy below, at and above threshold, the distinction being dictated by our different understanding of these systems. For instance, we will see that there has been noteworthy progress in describing radiative decays of quarkonium below threshold and that the theory is now in the position to provide for many of the transitions competitive and model-independent results.

Finally, on a more theoretical side, since the inception of non-relativistic EFTs there has been an ongoing investigation on how they realize Lorentz invariance. It has been shown in [738, 765] that HQET is reparameterization invariant. Reparameterization invariance constrains the form of the Wilson coefficients of the theory. In [766, 767] it was shown that the same constraints follow from imposing the Poincaré algebra on the generators of the Poincaré group in the EFT. Hence reparameterization invariance appears as the way in which Lorentz invariance, which is manifestly broken by a non-relativistic EFT, is retained order by order in $1/m_Q$ by the EFT. This understanding has recently been further substantiated in [768], where the consequences of reparameterization and Poincaré invariance have been studied to an unprecedented level of accuracy.

#### The progress on NRQCD factorization

The NRQCD factorization approach to heavy quarkonium production, introduced as a conjecture [728], is phenomenologically successful in describing existing data, although there remain challenges particularly in connection with polarization observations [757]. In the NRQCD factorization approach, the inclusive cross section for the direct production of a quarkonium state $H$ at large momentum transfer ($p_\mathrm{T}$) is written as a sum of “short-distance” coefficients times NRQCD long-distance matrix elements (LDMEs),

4.2 $$\begin{aligned} \sigma ^H(p_\mathrm{T},m_Q) = \sum _n \sigma _n(p_\mathrm{T},m_Q,\Lambda ) \langle 0| \mathcal{O}_n^H(\Lambda )|0\rangle . \end{aligned}$$

Here $\Lambda$ is the ultraviolet cut-off of the NRQCD effective theory. The *short-distance* coefficients $\sigma _n$ are essentially the process-dependent partonic cross sections to produce a $Q\bar{Q}$ pair in various color, spin, and orbital angular momentum states $n$ (convolved with the parton distributions of incoming hadrons for hadronic collisions), and perturbatively calculated in powers of $\alpha _\mathrm{s}$. The LDMEs are non-perturbative, but universal, representing the probability for a $Q\bar{Q}$ pair in a particular state $n$ to evolve into a heavy quarkonium. The sum over the $Q\bar{Q}$ states $n$ is organized in terms of powers of the pair’s relative velocity $v$, an intrinsic scale of the LDMEs. For charmonia, $v^2\approx 0.3$, and for bottomonia, $v^2\approx 0.1$. The current successful phenomenology of quarkonium production mainly uses only NRQCD LDMEs through relative order $v^4$, as summarized in Table 5. The traditional color singlet model is recovered as the $v\rightarrow 0$ limit. In case of $P$ wave quarkonia and relativistic corrections to $S$ state quarkonia [769], the color singlet model is incomplete, due to uncanceled infrared singularities.

**Table 5:** NRQCD velocity scaling of the LDMEs contributing to ${^3}S_1$ quarkonium production up to the order $O(v^4)$ relative to the leading ${^3}S_1$ color singlet contribution [728]. Upper indices $^{[1]}$ refer to color singlet states and upper indices $^{[8]}$ to color octet states. The $\langle \mathcal{O}^{J/\psi }({^3}S_1^{[1]}) \rangle$, $\langle \mathcal{P}^{J/\psi }({^3}S_1^{[1]}) \rangle$, and $\langle \mathcal{Q}^{J/\psi }({^3}S_1^{[1]}) \rangle$ LDMEs correspond to the leading order, and the $O(v^{2})$ and $O(v^4)$ relativistic correction contributions to the color singlet model. The contributions involving the $\langle \mathcal{O}^{J/\psi }({^1}S_0^{[8]}) \rangle$, $\langle \mathcal{O}^{J/\psi }({^3}S_1^{[8]}) \rangle$ and $\langle \mathcal{O}^{J/\psi }({^3}P_J^{[8]}) \rangle$ LDMEs are often referred to as *the* Color Octet states

Relative scaling	Contributing LDMEs
1	$\langle \mathcal{O}^{H}({^3}S_1^{[1]}) \rangle$
$v^{2}$	$\langle \mathcal{P}^{H}({^3}S_1^{[1]}) \rangle$
$v^3$	$\langle \mathcal{O}^{H}({^1}S_0^{[8]}) \rangle$
$v^4$	$\langle \mathcal{Q}^{H}({^3}S_1^{[1]}) \rangle$, $\langle \mathcal{O}^{H}({^3}S_1^{[8]}) \rangle$, $\langle \mathcal{O}^{H}({^3}P_J^{[8]}) \rangle$

Despite the well-documented phenomenological successes, there remain two major challenges for the NRQCD factorization approach to heavy quarkonium production. One is the validity of the factorization itself, which has not been proved, and the other is the difficulty in explaining the polarization of produced quarkonia in high-energy scattering, as will be reviewed in Sect. 4.5. These two major challenges could well be closely connected to each other, and could also be connected to the observed tension in extracting LDMEs from global analyses of all data from different scattering processes [770]. A proof of the factorization to all orders in $\alpha _\mathrm{s}$ is complicated because gluons can dress the basic factorized production process in ways that apparently violate factorization. Although there is a clear scale hierarchy for heavy quarkonium, $m_Q \gg m_Q v \gg m_Q v^2$, which is necessary for using an effective field theory approach, a full proof of NRQCD factorization would require a demonstration that all partonic diagrams at each order in $\alpha _\mathrm{s}$ can be reorganized such that (1) all soft singularities cancel or can be absorbed into NRQCD LDMEs, and (2) all collinear singularities and spectator interactions can be either canceled or absorbed into incoming hadrons’ parton distributions. So far, this has been established at all orders only for exclusive production in helicity-non-flip processes in $e^+e^{-}$ annihilation and $B$-meson decay [771–773].

For heavy quarkonium production at collider energies, there is sufficient phase space to produce more than one pair of heavy quarks, and additional observed momentum scales, such as $p_\mathrm{T}$. The NRQCD factorization in (4.2) breaks when there are co-moving heavy quarks [774, 775]. The short-distance coefficient $\sigma _n(p_\mathrm{T},m_Q,\Lambda )$ in (4.2) for a $Q\bar{Q}(n)$ state can have different power behavior in $p_\mathrm{T}$ at different orders in $\alpha _\mathrm{s}$. For example, for $Q\bar{Q}(^3S_1^{[1]})$-channel, the Leading Order (LO) coefficient in $\alpha _\mathrm{s}$ is dominated by $1/p_\mathrm{T}^8$, and the Next-to-Leading Order (NLO) dominated by $1/p_\mathrm{T}^6$, while the Next-to-Next-to-Leading Order (NNLO) coefficient has terms proportional to $1/p_\mathrm{T}^4$. When $p_\mathrm{T}$ increases, the logarithmic dependence of $\alpha _\mathrm{s}$ on the hard scale cannot compensate the power enhancement in $p_\mathrm{T}$ at higher orders, which leads to an unwanted phenomenon that the NLO correction to a given channel could be an order of magnitude larger than the LO contribution [776, 777]. Besides the power enhancement at higher orders, the perturbative coefficients at higher orders have higher powers of large $\ln (p_\mathrm{T}^2/m_Q^2)$-type logarithms, which should be systematically resummed. That is, when $p_\mathrm{T}\gg m_Q$, a new organization of the short-distance coefficients in (4.2) or a new factorization formalism is necessary. Very significant progress has been made in recent years.

Two new factorization formalisms were derived for heavy quarkonium production at large $p_\mathrm{T}$. One is based on perturbative QCD (pQCD) collinear factorization [778–784], and the other based on soft collinear effective theory (SCET) [785, 786]. Both approaches focus on quarkonium production when $p_\mathrm{T}\gg m_Q$, and explore potential connections to the NRQCD factorization.

The pQCD collinear factorization approach, also referred to as the fragmentation function approach [757], organizes the contributions to the quarkonium production cross section in an expansion in powers of $1/p_\mathrm{T}$, and then factorizes the leading power (and the next-to-leading power) contribution in terms of “short-distance” production of a single-parton of flavor $f$ (and a heavy quark pair $[Q\bar{Q}(\kappa )]$ with $\kappa$ labeling the pair’s spin and color) convolved with a universal fragmentation function for this parton (and the pair) to evolve into a heavy quarkonium,

4.3 $$\begin{aligned} \mathrm{d}\sigma _\mathrm{H}(p_\mathrm{T},m_Q)&\approx \sum _{f} d\hat{\sigma }_\mathrm{f}(p_\mathrm{T},z)\otimes D_{f\rightarrow H}(z,m_Q)\, \nonumber \\&+ \sum _{[Q\bar{Q}(\kappa )]} d\hat{\sigma }_{[Q\bar{Q}(\kappa )]}(p_\mathrm{T},z,u,v)\nonumber \\&\&\otimes \mathcal{D}_{[Q\bar{Q}(\kappa )] \rightarrow H}(z,u,v,m_Q) , \end{aligned}$$

where factorization scale dependence was suppressed, $z,u,v$ are momentum fractions, and $\otimes$ represents the convolution of these momentum fractions. Both the single parton and heavy quark pair fragmentation functions, $D_\mathrm{f}$ and $\mathcal{D}_{[Q\bar{Q}(\kappa )]}$, are universal, and we can resum large logarithms by solving the corresponding evolution equations [780, 782, 783]. The factorization formalism in (4.3) holds to all orders in $\alpha _\mathrm{s}$ in pQCD up to corrections of $\mathcal{O}(1/p_\mathrm{T}^4)$ ($\mathcal{O}(1/p_\mathrm{T}^2)$) with (without) a heavy quark pair, $[Q\bar{Q}(\kappa )]$, being produced [778, 780, 783].

Including the $1/p_\mathrm{T}$-type power correction into the factorized production cross section in (4.3) necessarily requires modifying the evolution equation of a single parton fragmentation function as [780, 783],

4.4 $$\begin{aligned}&\frac{\partial }{\partial \ln \mu ^2}D_{f\rightarrow H}(z,\mu ^2;m_Q) = \sum _{f'} \gamma _{f\rightarrow f'} \otimes D_{f'\rightarrow H} \nonumber \\&\quad +\, \frac{1}{\mu ^2} \sum _{[Q\bar{Q}(\kappa ')]} \gamma _{f\rightarrow [Q\bar{Q}(\kappa ')]} \otimes \mathcal{D}_{[Q\bar{Q}(\kappa ')]\rightarrow H}, \end{aligned}$$

where $\otimes$ represents the convolution of momentum fractions as those in (4.3), and the dependence of momentum fractions in the right-hand-side is suppressed. The first line in (4.3) is effectively equal to the well-known DGLAP evolution equation. The second term on the right of (4.3) is new, and is needed for the single-parton fragmentation functions to absorb the power collinear divergence of partonic cross sections producing a “massless” ($m_Q/p_\mathrm{T} \sim 0$) heavy quark pair to ensure that the short-distance hard part, $\hat{\sigma }_{[Q\bar{Q}(\kappa )]}(p_\mathrm{T},z,u,v)$ in (4.3), is infrared and collinear safe [784]. The modified single-parton evolution equation in (4.4), together with the evolution equation of heavy quark-pair fragmentation functions [780, 783, 785],

4.5 $$\begin{aligned}&\frac{\partial }{\partial \ln \mu ^2}\mathcal{D}_{[Q\bar{Q}(\kappa )]\rightarrow H}(z,u,v,\mu ^2;m_Q) \nonumber \\&\quad = \sum _{[Q\bar{Q}(\kappa ')]} \Gamma _{[Q\bar{Q}(\kappa )]\rightarrow [Q\bar{Q}(\kappa ')]} \otimes \mathcal{D}_{[Q\bar{Q}(\kappa ')]\rightarrow H}, \end{aligned}$$

forms a closed set of evolution equations of all fragmentation functions in (4.3). The $\mathcal{O}(\alpha _\mathrm{s}^2)$ evolution kernels for mixing the single-parton and heavy quark-pair fragmentation functions, $\gamma _{f\rightarrow [Q\bar{Q}(\kappa ')]}$ in (4.4), are available [783], and the $\mathcal{O}(\alpha _\mathrm{s})$ evolution kernels of heavy quark-pair fragmentation functions, $\Gamma _{[Q\bar{Q}(\kappa )]\rightarrow [Q\bar{Q}(\kappa ')]}$ in (4.5), were derived by two groups [783, 786].

For production of heavy quarkonium, it is necessary to produce a heavy quark pair. The combination of the QCD factorization formula in (4.3) and the evolution equation in (4.4) presents a clear picture of how QCD organizes the contributions to the production of heavy quark pairs in terms of distance scale (or time) where (or when) the pair was produced. The first (the second) term in (4.3) describes the production of the heavy quark pairs after (at) the initial hard partonic collision. The first term in (4.4) describes the evolution of a single active parton before the creation of the heavy quark pair, and the power-suppressed second term summarizes the leading contribution to the production of a heavy quark pair at any stage during the evolution. Without the power-suppressed term in (4.4), the evolved single-parton fragmentation function is restricted to the situation when the heavy quark pair is only produced *after* the time corresponding to the input scale of the evolution $\mu _0\gtrsim 2m_Q$. With perturbatively calculated short-distance hard parts [784] and evolution kernels [783, 786], the predictive power of the pQCD factorization formalism in (4.3) relies on the experimental extraction of the universal fragmentation functions at the input scale $\mu _0$, at which the $\ln (\mu _0^2/(2m_Q)^2)$-type contribution is comparable to $(2m_Q/\mu _0)^2$-type power corrections. It is these input fragmentation functions at $\mu _0$ that are responsible for the characteristics of producing different heavy quarkonium states, such as their spin and polarization, since perturbatively calculated short-distance partonic hard parts and evolution kernels of these fragmentation functions are universal for all heavy quarkonium states.

The input fragmentation functions are universal and have a clear scale hierarchy $\mu _0 \gtrsim 2m_Q \gg m_Q v$. It is natural to apply the NRQCD factorization in (4.2) to these input fragmentation functions as [781, 784]

4.6 $$\begin{aligned}&D_{f\rightarrow H}(z,m_Q,\mu _0) = \sum _n d_{f\rightarrow n}(z,m_Q,\mu _0)\langle 0| \mathcal{O}_n^H |0\rangle \nonumber \\&\mathcal{D}_{[Q\bar{Q}(\kappa )]\rightarrow H}(z,u,v,m_Q,\mu _0) \\&\quad = \sum _n d_{[Q\bar{Q}(\kappa )]\rightarrow n}(z,u,v,m_Q,\mu _0)\langle 0| \mathcal{O}_n^H | 0 \rangle \, . \nonumber \end{aligned}$$

The above NRQCD factorization for single-parton fragmentation functions was verified to NNLO [778], and was also found to be valid for heavy-quark pair fragmentation functions at NLO [787, 788]. But a proof to all orders in NRQCD is still lacking. If the factorization in (4.6) would be proved to be valid, the pQCD factorization in (4.3) is effectively a reorganization of the NRQCD factorization in (4.2) when $p_\mathrm{T}\gg m_Q$, which resums the large logarithmic contributions to make the perturbative calculations much more reliable [781, 784]. In this case, the experimental extraction of the input fragmentation functions at $\mu _0$ is reduced to the extraction of a few universal NRQCD LDMEs.

When $p_\mathrm{T}\gg m_Q$, the effective theory, NRQCD, does not contain all the relevant degrees of freedom. In addition to the soft modes absorbed into LDMEs, there are also dangerous collinear modes when $m_Q/p_\mathrm{T} \sim 0$. On the other hand, SCET [789, 790] is an effective field theory coupling soft and collinear degrees of freedom and should be more suited for studying heavy quarkonium production when $p_\mathrm{T}\gg m_Q$. The SCET approach matches QCD onto massive SCET at $\mu \sim p_\mathrm{T}$ and expands perturbatively in powers of $\alpha _\mathrm{s}(p_\mathrm{T})$ with a power counting parameter $\lambda \sim (2m_Q)/p_\mathrm{T}$. The approach derives effectively the same factorization formalism for heavy quarkonium production as that in (4.3) for the first two powers in $\lambda$. However, the derivation in SCET, due to the way the effective theory was set up, does not address the cancellation of Glauber gluon interactions between spectators, and may face further difficulties having to do with infinite hierarchies of gluon energy scales, and therefore may be not as complete as in the pQCD approach. As expected, the new fragmentation functions for a heavy quark pair to fragment into a heavy quarkonium obey the same evolution equations derived in the pQCD collinear factorization approach. Recently, it was verified that the first-order evolution kernels for heavy-quark pair fragmentation functions calculated in both pQCD and SCET approaches are indeed consistent [782, 783, 786]. However, it is not clear how to derive the evolution kernels for mixing the single-parton and heavy quark-pair fragmentation functions, like $\gamma _{f\rightarrow [Q\bar{Q}(\kappa ')]}$ in (4.4), in SCET [791].

In the SCET approach to heavy quarkonium production, the heavy-quark pair fragmentation functions defined in SCET are matched onto NRQCD after running the fragmentation scale down to the order of $2m_Q$. It was argued [785] that the matching works and NRQCD results can be recovered under the assumption that the LDMEs are universal. However, the NRQCD factorization in (4.6) has not been proved to all orders in pQCD because of the potential for the input fragmentation functions to have light-parton jet(s) of order of $m_Q$. It is encouraging that major progress has been achieved in understanding heavy quarkonium production and its factorization recently, but more work is still needed.

#### Lattice gauge theory

With ensembles at very fine lattice spacings becoming increasingly available due to the continuous growth of available computer power, simulations employing relativistic valence charm quarks are now becoming more and more common. Indeed, the first ensembles incorporating dynamical (sea) charm quarks [42, 792, 793] are beginning to become available.

**Fig. 29:**
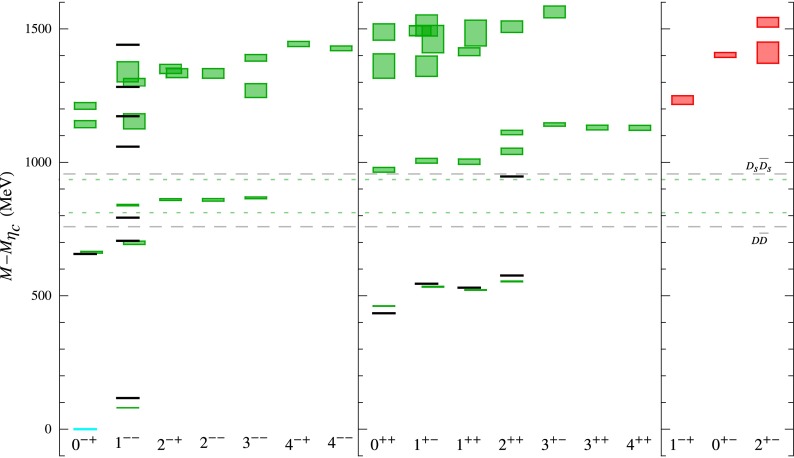
The charmonium spectrum from lattice simulations of the Hadron Spectrum Collaboration using $N_\mathrm{f}=2+1$ flavors of dynamical light quarks and a relativistic valence charm quark on anisotropic lattices. The *shaded boxes* indicate the $1\sigma$ confidence interval from the lattice for the masses relative to the simulated $\eta _\mathrm{c}$ mass, while the corresponding experimental mass differences are shown as *black lines*. The $D\overline{D}$ and $D_\mathrm{s}\overline{D}_\mathrm{s}$ thresholds from lattice simulation and experiment are shown as *green* and *grey dashed lines*, respectively. From [812]

The heavy mass of the charm quark means that (since $m_ca\!\not \ll \! 1$) discretization effects cannot be completely neglected and have to be accounted for properly. This is possible using the Symanzik effective theory formalism [794, 795]. For any given lattice action, it is possible to formulate an effective theory (the Symanzik effective theory) defined in the continuum, which has the lattice spacing $a$ as its dimensionful expansion parameter and incorporates all operators compatible with the symmetries of the lattice action (including Lorentz-violating term with hypercubic symmetry), and the short-distance coefficients of which are fixed by determining that it should reproduce the on-shell matrix elements of the lattice theory up to some given order in $a$. The use of this effective theory in lattice QCD is twofold [796]: firstly, it provides a means to parameterize the discretization artifacts as a function of the lattice spacing, thus allowing an extrapolation to the $a\rightarrow 0$ continuum limit from a fit to results obtained at a range of (sufficiently small) lattice spacings. Secondly, one can take different lattice actions discretizing the same continuum theory and consider a lattice action formed from their weighted sum with the weights chosen so as to ensure that the leading short-distance coefficients of the Symanzik effective action become zero for the resulting (improved) action. Examples of improved actions in current use are the Sheikholeslami-Wohlert (clover) action [797, 798], which removes the O($a$) artifacts of the Wilson quark action, and the asqtad ($a^2$ tadpole-improved) [799] and HISQ (Highly Improved Staggered Quark) [800] actions for staggered quarks. Likewise, it is possible to improve the lattice action for NRQCD [737, 801] so as to remove O($a^2$) artifacts. The operators used to measure correlation functions may be improved in a similar fashion; cf. e.g. [802–804] for the O($a$) improvement of the static-light axial and vector currents used in HQET. Finally, one can use HQET instead of the Symanzik theory to understand the cutoff effects with heavy quarks [805–807], which when applied to the Wilson or clover action is known as the Fermilab method [808].

Since the experimental discovery of the $X(3872)$ resonance by the Belle collaboration [809], and the subsequent emergence of more and more puzzling charmonium-like states, the spectroscopy of charmonium has gained increased interest. Lattice studies of states containing charm quarks are thus of great importance, as they provide an *a priori* approach to charm spectroscopy. The use of relativistic charm quarks eliminates systematic uncertainties arising from the use of effective theories, leaving discretization errors as the leading source of systematic errors, which can in principle be controlled using improved actions.

A variety of lattice studies with different actions are now available, with both the HISQ [800] action [810], and O($a$)-improved Wilson fermions [811] having been used for a fully relativistic treatment of the charm quark. In addition, anisotropic lattices have been employed to improve the time resolution of the correlation functions to allow for better control of excited states [812] (Fig. 29). An important ingredient in all spectroscopy studies is the use of the variational method [356–358] to resolve excited states.

As flavor singlets, charmonium states also receive contributions from quark-disconnected diagrams representing quark–antiquark annihilation and mixing with glueball and light-quark states [813]. Using improved stochastic estimators, Bali et al. [814] have studied disconnected contributions, finding no resulting energy shift within the still sizeable statistical errors. The use of the new “distillation” method [355, 815] to estimate all-to-all propagators has been found to be helpful in resolving disconnected diagrams, whose contributions have been found to be small [816].

Besides the mixing with non-$c\bar{c}$ states arising from the disconnected diagram contributions, quarkonium states above or near the open-charm threshold may also mix with molecular $D\overline{D}$ and tetraquark states. Studies incorporating these mixings [814, 817] have found evidence for a tightly bound molecular $D\overline{D}^*$ state. Recently, a study of $DD^*$ scattering on the lattice [818] using Lüscher’s method [396] found the first evidence of an $X(3872)$ candidate. It was found that the observed spectrum of states near the threshold depends strongly on the basis of operators used; in particular, the $X(3872)$ candidate was not observed if only $\bar{c}c$ operators, but no dimeson operators, were included in the basis, nor if the basis contained only dimeson, but no $\bar{c}c$ operators. This was interpreted as evidence that the $X(3872)$ might be the consequence of an accidental interference between $\bar{c}c$ and scattering states. On the other hand, it could also be seen as rendering the results of this and similar studies doubtful in so far as it cannot be easily excluded that the inclusion of further operators might not change the near-threshold spectrum again. A significant challenge in this area is thus to clarify which operators are needed to obtain reliable physical results. Recently, it has been suggested [819] based on large-$N$ arguments that for tetraquark operators the singly disconnected contraction is of leading order in $1/N$ whenever it contributes. This would appear to apply also to the tetraquark operators relevant near the open-charm threshold, making use of all-to-all methods such as distillation [355, 815] (which was used in [818]) mandatory for near-threshold studies.

The spectra of the open-charm $D$ and $D_\mathrm{s}$ mesons have been studied by Mohler and Woloshyn [820] using the Fermilab formalism for the charm quark. It was found that while the ground state $D$, $D^*$, $D_\mathrm{s}$ and $D_\mathrm{s}^*$ masses were reasonably well reproduced, the masses of the $D_J$ and $D_{sJ}$ states from their simulation strongly disagreed with experiment; possible reasons include neglected contributions from mixing with multihadron states.

As for $b$ quarks, the currently achievable lattice spacings do not allow the direct use of relativistic actions. An interesting development in this direction is the use of highly improved actions (such as HISQ [800]) to simulate at a range of quark masses around and above the physical charm quark mass, but below the physical $b$ quark mass, in order to extrapolate to the physical $b$ quark mass using Bayesian fits [821] incorporating the functional form of the expected discretization artifacts and $1/m_Q$ corrections [822, 823]. This method relies on the convergence of the Symanzik expansion up to values of $m_Qa\sim 1$, and of the heavy-quark expansion in the vicinity of the charm quark mass; neither assumption can be proven with present methods, but empirical evidence [824] suggests that at least for the heavy-quark expansion convergence is much better than might naively be expected. The removal of as many sources of discretization errors as possible, including using the $N_\mathrm{f}=2+1+1$ HISQ MILC ensembles [825] with reduced sea quark discretization effects [826] might be helpful in addressing the question of the convergence of the expansion in $a$.

Otherwise, simulations of $b$ quarks need to rely on effective field theories, specifically non-perturbatively matched HQET [827–829] for heavy–light systems, and NRQCD or m(oving)NRQCD [737, 801, 830] for heavy–heavy, as well as heavy–light, systems. An important point to note in this context is that each discretization choice (such as the use of HYP1 versus HYP2 links [831–833] in the static action of HQET, or the use of different values of the stability parameter in the lattice NRQCD action [736, 834, 835]) within either approach constitutes a separate theory with its own set of renormalization constants which must be matched to continuum QCD separately.

The non-perturbative matching of HQET to quenched QCD at order $1/m_b$ has been accomplished in [804], and subsequent applications to the spectroscopy [836] and leptonic decays [824] of the $B_\mathrm{s}$ system have showcased the power of this approach. The extension to $N_\mathrm{f}=2$ is well under way [837, 838], and future studies at $N_\mathrm{f}=2+1$ are to be expected. Beyond the standard observables such as masses and decay constants, observables featuring in effective descriptions of strong hadronic interactions, such as the $B^*B\pi$ coupling in Heavy Meson Chiral Perturbation Theory [246, 839, 840] and the $B^{*'}\rightarrow B$ matrix element [841] have been studied successfully in this approach.

In NRQCD, until recently only tree-level actions were available. In [842, 843], the one-loop corrections to the coefficients $c_1$, $c_5$ and $c_6$ of the kinetic terms in an $\mathrm {O}(v^4)$ NRQCD lattice action have been calculated, and in [835, 844], the background field method has been used to calculate also the one-loop corrections to the coefficients $c_2$ and $c_4$ of the chromomagnetic $\sigma \cdot \mathbf{B}$ and chromoelectric Darwin terms for a number of lattice NRQCD actions. Simulations incorporating these perturbative improvements [843, 845] have shown a reduced lattice-spacing dependence and improved agreement with experiment.

Matching the NRQCD action to QCD beyond tree-level has a significant beneficial effect on lattice determinations of bottomonium spectra, in particular for the case of the bottomonium $1S$ hyperfine splitting, which moves from $\Delta M_\mathrm{HF}(1S) = 61(14)$ MeV without the perturbative improvements [834] to $\Delta M_\mathrm{HF}(1S) =70(9)$ MeV with the perturbative improvements [843].

The most recent determination based on lattice NRQCD, including $O(v^6)$ corrections, radiative one-loop corrections to $c_4$, non-perturbative four-quark interactions and the effect of $u$, $d$, $s$ and $c$ sea quarks, gives $\Delta M_\mathrm{HF}(1S) = (62.8 \pm 6.7)\,~\mathrm {MeV}$ [846], which is to be compared to the PDG value of $\Delta M_\mathrm{HF}(1S) = 69.3(2.9)$ MeV [1] excluding the most recent Belle data [847], or $\Delta M_\mathrm{HF}(1S) = 64.5(3.0)$ MeV [1] when including them.

The resulting prediction for the bottomonium 2S hyperfine splitting of $\Delta M_\mathrm{HF}(2S) = 35(3)(1)$ MeV [843] is in reasonable agreement with the Belle result $\Delta M_\mathrm{HF}(2S) =24.3^{+4.0}_{-4.5}$ MeV [848], but disagrees with the CLEO result of Dobbs et al., $\Delta M_\mathrm{HF}(2S) = 48.7(2.3)(2.1)$ MeV [849]; see discussion in Sect. 4.3.

Another factor with a potentially significant influence on the bottomonium hyperfine splitting is the lack of, or the inclusion of, spin-dependent interactions at higher orders in the non-relativistic expansion. In [850], it was shown that including the $\mathrm {O}(v^6)$ spin-dependent terms in the NRQCD action leads to an increase in the 1S hyperfine splitting, moving it away from the experimental value. The results of [835] suggest that this effect will at least partially be compensated by the inclusion of perturbative corrections to the coefficients of the spin-dependent operators. The $2S$ hyperfine splitting is not similarly affected, and the prediction $\Delta M_\mathrm{HF}(2S) = 23.5(4.1)(2.1)(0.8)$ MeV of [850] is in excellent agreement with the Belle value [848].

The $B_\mathrm{c}$ system combines the challenges of both the $b$ and charm sectors, while also allowing for one of the relatively few predictions from QCD that is *not* to some extent a “postdiction” in that it precedes experiment, *viz.* the mass of the as yet undiscovered $B_\mathrm{c}^*$ meson, which has been predicted by the HPQCD collaboration to be $M_{B_\mathrm{c}^*}=6330(7)(2)(6)$ MeV [851] using NRQCD for the $b$ and the HISQ action for the charm quarks. Reproducing this prediction using another combination of lattice actions might be worthwhile. For the time being, we note that the lattice prediction compares very well with the perturbative calculation of [852], which gives $M_{B_\mathrm{c}^*}=6327(17)^{+15}_{-12}(6)$ MeV to next-to-leading logarithmic accuracy.

### Heavy semileptonic decays

Semileptonic decays of $B$ and $D$ mesons have been extensively studied in the last years. They provide information about the CKM matrix elements $|V_{cb}|$, $|V_{ub}|$, $|V_{cd}|$ and $|V_{cs}|$ through exclusive and inclusive processes driven by $b \rightarrow c(u)$ and $c \rightarrow s(d)$ decays, respectively (for recent reviews see, e.g., Refs. [853–857]).

The leptonic decays $B^{+} \rightarrow l^{+} \nu$ and $D^+_{(s)} \rightarrow l^{+} \nu$ can also be used for the determination of CKM matrix elements. The advantages of semileptonic decays are that they are not helicity suppressed and new physics is not expected to play a relevant role; so, it is generally, but not always, disregarded.

In deep inelastic neutrino (or antineutrino)–nucleon scattering, single charm particles can be produced through $dc$ and $sc$ electroweak currents. Analyses based on neutrino and antineutrino interactions give a determination of $|V_{cd}|$ with comparable, and often better, precision than the ones obtained from semileptonic charm decays. Not so for the determination of $|V_{cs}|$, which suffers from the uncertainty of the s-quark sea content [1]. On-shell $W^\pm$ decays sensitive to $|V_{cs}|$ have also been used [858], but semileptonic $D$ or leptonic $D_\mathrm{s}$ decays provide direct and more precise determinations.

#### Exclusive and inclusive $D$ decays

The hadronic matrix element for a generic semileptonic decay $H \rightarrow P l \nu$, where $H$ and $P$ denote a heavy and a light pseudoscalar meson, respectively, is usually written in terms of two form factors $f_+(q^2)$ and $f_0(q^2)$

4.7 $$\begin{aligned} \langle P(p_P)| J^\mu | H(p_\mathrm{H}) \rangle&= f_+(q^2) \left( p_\mathrm{H}^\mu +p_P^\mu \!-\! \frac{m^2_\mathrm{H}-m_P^2}{q^2} q^\mu \right) \nonumber \\&\!\! + f_0(q^2) \frac{m^2_\mathrm{H}-m_P^2}{q^2} q^\mu , \end{aligned}$$

where $$ q \equiv p_\mathrm{H} - p_P$$ is the momentum transferred to the lepton pair, and $$J^\mu $$ denotes the heavy-to-light vector current. In the case of massless leptons, the form factor $$f_0(q^2)$$ is absent and the differential decay rate depends on $$f_+(q^2) $$ only.

The main theoretical challenge is the non-perturbative evaluation of the form factors. In this section, we consider $H$ to be a $D_{(q)}$ meson. For simplicity’s sake, one can split the non-perturbative evaluation of the form factors into two steps, the evaluation of their normalization at $q^2=0$ and the determination of their $q^2$ dependence.

The form factors are expected to decrease at low values of $q^2$, that is at high values of spectator quark recoil. Indeed, in the leading spectator diagram, the probability of forming a hadron in the final state decreases as the recoil momentum of the spectator quark increases. Moreover, the form factors are expected to be analytic functions everywhere in the complex $q^2$ plane outside a cut extending along the positive $q^2$ axis from the mass of the lowest-lying $c \bar{q}$ resonance. That implies they can be described by dispersion relations, whose exact form is not known a priori, but can be reasonably assumed to be dominated, at high $q^2$, by the nearest poles to $q^2_{\mathrm {max}}= (m_{D_{(q)}}-m_P)^2$. Pole dominance implies current conservation at large $q^2$. We expect the form factors to have a singular behavior as $q^2$ approaches the lowest lying poles, without reaching them, since they are beyond the kinematic cutoff. The simplest parameterization of the $q^2$ dependence motivated by this behavior is the simple pole model, where a single pole dominance is assumed. By restricting to the form factor $f_+(q^2)$, we have

4.8 $$\begin{aligned} f_+(q^2)= \frac{f_+(0)}{1-\frac{q^2}{m_{\mathrm {pole}}^2}}. \end{aligned}$$

In $D \rightarrow \pi l \nu$ decays, the pole for $f_+(q^2)$ corresponds to the $c \bar{d}$ vector meson of lowest mass $D^\star$. In $D \rightarrow K l \nu$ and $D_\mathrm{s} \rightarrow \eta ^{(\prime )} l \nu$ decays, the poles correspond to the $c \bar{s}$ vector mesons and the lowest resonance compatible with $J^P=1^{-}$ is $D_\mathrm{s}^{*\pm }$, with mass $M_ {D_\mathrm{s}^{*}}= 2112.3 \pm 0.5$ MeV. Form factor fits have been performed for $D \rightarrow K(\pi ) l \nu$ by the CLEO [859] and BESIII Collaborations [860], where several models for the $q^2$ shape have been considered. In the simple pole model, agreement with data is only reached when the value of $m_{\mathrm {pole}}$ is not fixed at the $D^\star _{(s)}$ mass, but is a free parameter. In order to take into account higher poles, while keeping the number of free parameters low, a modified pole model has been proposed [861], where

4.9 $$\begin{aligned} f_+(q^2)= \frac{f_+(0)}{ \left( 1-\frac{q^2}{m_{\mathrm {pole}}^2}\right) \left( 1- \alpha \frac{q^2}{m_{\mathrm {pole}}^2}\right) }. \end{aligned}$$

Another parameterization, known as the series or $z$-expansion, is based on a transformation that maps the cut in the $q^2$ plane onto a unit circle in another variable, $z$, and fits the form factor as a power series (in $z$) with improved properties of convergence [862–864]. More in detail, the first step is to remove poles by the form factors, that is, for the $B \rightarrow K$ decays

4.10 $$\begin{aligned} \tilde{f}_0^{D \rightarrow K } (q^2)&= \left( 1 - \frac{q^2}{ m_{D^*_{s 0}}^2 }\right) f_0^{D \rightarrow K } (q^2) \nonumber \\ \tilde{f}_+^{D \rightarrow K } (q^2)&= \left( 1 - \frac{q^2}{ m_{D^*_{s}}^2 }\right) f_+^{D \rightarrow K } (q^2) \end{aligned}$$

The variable $z$ is defined as

4.11 $$\begin{aligned} z(q^2) = \frac{ \sqrt{t_+ - q^2}- \sqrt{t_+ - t_0} }{ \sqrt{t_+ - q^2} + \sqrt{t_+ - t_0}} \qquad t_+ = (m_D+m_K)^2\nonumber \\ \end{aligned}$$

The final step consists in fitting $\tilde{f}$ as a power series in $z$,

4.12 $$\begin{aligned} \tilde{f}_0^{D \rightarrow K } (q^2)&= \sum _{n \ge 0} c_n z^n \nonumber \\ \tilde{f}_+^{D \rightarrow K } (q^2)&= \sum _{n \ge 0} b_n z^n \qquad c_0=b_0 \end{aligned}$$

Employing this parameterization, the shapes of $f_{0,+}^{D \rightarrow K }$ form factors have been very recently estimated by the HPQCD Collaboration [865].

To evaluate the normalization of the form factors, lattice and QCD sum rules are generally employed. Lately, high statistics studies on the lattice have become available and preliminary results for $f_{0,+}^{D \rightarrow K/\pi }$ have been presented by ETMC [866, 867], HPQCD [868] and Fermilab/MILC [869].

The most recent published $|V_{cd}|$ estimates are from HPQCD [870], where the value of $|V_{cd}|$ has been evaluated using the Highly Improved Staggered Quark (HISQ) action for valence charm and light quarks on MILC $N_\mathrm{f}=2+1$ lattices with experimental inputs from CLEO [871] and BESIII [872]. The value $|V_{cd}| = 0.223 \pm 0.010_{\mathrm {exp}} \pm 0.004_{\mathrm {lat}}$ [870], with the first error coming from experiment and the second from the lattice computation, is in agreement with the value of $|V_{cd}|$ the same collaboration has recently extracted from leptonic decays. It also agrees, with a competitive error, with the value $|V_{cd}| = 0.230 \pm 0.011$ [1] from neutrino scattering.

The same HPQCD collaboration gives also the most recent $$|V_{cs}| $$ estimate by analyzing $$D \rightarrow K/\pi \, l \, \nu $$, $$D_\mathrm{s} \rightarrow \phi /\eta _\mathrm{s} \, l \, \nu $$ and using experimental inputs from CLEO [859], BaBar [873, 874], Belle [875]. and BESIII (preliminary) [860]. Their best value $$ |V_{cs}| = 0.963 \pm 0.005_{\mathrm {exp}} \pm 0.014_{\mathrm {lat}}$$ is in agreement with values from indirect fits [1]. The big increase in accuracy with respect to their older determinations, is due to the larger amount of data employed. Specifically they have used all experimental $$q^2$$ bins, rather than just the $$q^2 \rightarrow 0$$ limit or the total rate. The FLAG $$N_\mathrm{f}=2+1$$ average value from semileptonic decays gives $$|V_{cs}|= 0.9746 \pm 0.0248 \pm 0.0067$$ [876]. Experiments at BESIII, together with experiments at present and future flavor factories, all have the potential to reduce the errors on the measured decay branching fractions of $$D^+_{(s)}$$ and $$D^0$$ leptonic and semileptonic decays, in order to allow more precise comparison of these CKM matrix elements. In particular, BESIII is actively working on semileptonic charm decays; new preliminary results on the branching fractions and form factors in the parameterizations mentioned above, for the $$D \rightarrow K/\pi e \nu $$ channels, have been recently reported [877].

Lattice determinations of the decay constant $f_{D_\mathrm{s}}$ governing the leptonic decays $D_\mathrm{s}^+\rightarrow \mu ^+\nu$ and $D_\mathrm{s}^+\rightarrow \tau ^+\nu$ have for several years exhibited the “$f_{D_\mathrm{s}}$ puzzle”, an apparent $(3-4)\sigma$ discrepancy between lattice determinations of $f_{D_\mathrm{s}}$ [878–881] and the value of $f_{D_\mathrm{s}}$ inferred from experimental measurements of the branching ratios $B(D_\mathrm{s}^+\rightarrow \mu ^+\nu )$ and $B(D_\mathrm{s}^+\rightarrow \tau ^+\nu )$ [882–886]. When this discrepancy first appeared, it was immediately discussed as a signal for new physics [887]; in the meantime, however, careful investigation of all sources of systematic error, combined with increased statistics, has led to the lattice values shifting up slightly [888–890] and the experimental values shifting down noticeably [891–894], thus more or less eliminating the “puzzle” [895]. However, the most recent determinations still show some tension versus the FLAG $N_\mathrm{f}=2+1$ average value from leptonic decays $|V_{cs}|= 1.018 \pm 0.011 \pm 0.021$ [876].

It is interesting to observe that, according to lattice determinations in [868], the form factors are insensitive to the spectator quark: The $D_\mathrm{s} \rightarrow \eta _\mathrm{s} l \nu$ and $D \rightarrow K l \nu$ form factors are equal within 3 %, and the same holds for $D_\mathrm{s} \rightarrow K l \nu$ and $D \rightarrow \pi l \nu$ within 5 %. This result, which can be tested experimentally, is expected by heavy quark symmetry to hold also for $B$ meson decays so that the $B_\mathrm{s} \rightarrow D_\mathrm{s}$ and $B \rightarrow D$ form factors would be equal.

QCD light-cone sum rules have also been employed to extract $|V_{cs}|$ and $|V_{cd}|$ [896], giving substantial agreement on the averages and higher theoretical error with respect to the previously-quoted lattice results. By using the same data and a revised version of QCD sum rules, errors on $|V_{cd}|$ have been reduced, but a higher average value has been obtained: $|V_{cd}|= 0.244 \pm 0.005 \pm 0.003 \pm 0.008$. The first and second errors are of an experimental origin and the third is due to the theoretical uncertainty [897].

Form factors for semileptonic transitions to a vector or a pseudoscalar meson have also been investigated within a model which combines heavy quark symmetry and properties of the chiral Lagrangian [898–900].

Exclusive semileptonic $D$ decays play also a role in better understanding the composition of the $\eta$ and $\eta ^{\prime }$ wave functions, a long-standing problem. The transitions $D_\mathrm{s}^{+} \rightarrow \eta ^{(\prime )} l^{+} \nu$ and $D^+\rightarrow \eta ^{(\prime )} l^{+} \nu$ are driven by weak interactions at the Cabibbo-allowed and Cabibbo-suppressed levels, and provide us with complementary information since they produce the $\eta ^{(\prime )}$ via their $s \bar{s}$ and $d \bar{d}$ components, respectively. In addition, $\eta ^{(\prime )}$ could be excited via a $gg$ component. That is important since it would validate, for the first time, an independent role of gluons in hadronic spectroscopy, outside their traditional domain of mediating strong interactions. Also $B$ decays, semileptonic or hadronic, have been similarly employed (see e.g., Refs. [853, 901, 902]). Experimental evidence of glueballs is searched for in a variety of processes at several experiments, e.g., BESIII and PANDA. In 2009 the first absolute measurement of $\mathcal{{B}} ( D_\mathrm{s}^{+} \rightarrow \eta ^{(\prime )} e^{+} \nu _e)$ [903] and the first observation of the $D^{+} \rightarrow \eta \, e^{+} \nu _e$ decay [904] were reported by CLEO. Improved branching fraction measurements, together with the first observation of the decay mode $D^{+} \rightarrow \eta ^\prime e^{+} \nu _e$ and the first form factor determination for $D^{+} \rightarrow \eta \, e^{+} \nu _e$, followed in 2011 [905]. On the theoretical side, recent lattice results have become available for the values of mixing angles [364, 365], quoting values of the mixing angle $\phi$ between $40^\circ$ and $50^\circ$. The latest analysis, by ETM, leads to a value of $\phi = (44 \pm 5)^\circ$ [906], with a statistical error only. Systematic uncertainties, difficult to estimate on the lattice, are likely to affect this result. Preliminary results by the QCDSF Collaboration [907, 908] give a mixing angle $\theta \sim -(7^\circ , 8^\circ )$ in the octet-singlet basis, that is, in the quark-flavor basis, $\phi = \theta +\arctan \sqrt{2} \sim 47^\circ$. Out of chorus is the lower value favored by the recent UKQCD staggered investigation [366], $\phi = (34 \pm 3)^\circ$. All lattice analyses do not include a gluonic operator, discussing only the relative quark content. The agreement with other determinations from semileptonic decays based on different phenomenological approaches and older data is remarkable (see, e.g., Refs. [901, 909–911]). Recent experimental and theoretical progress has increased the role of semileptonic $D$ decays with respect to traditional, low-energy analyses [912].

In the vector sector, the $\phi$–$\omega$ mixing is not expected as large as in the pseudoscalar one, because there is no additional mixing induced by the axial $U(1)$ anomaly. In the absence of mixing, the state $\omega$ has no strange valence quark and corresponds to $|u \bar{u} + d \bar{d}\rangle /\sqrt{2}$. Cabibbo-favored semileptonic decays of $D_\mathrm{s}$ are expected to lead to final states that can couple to $|\bar{s} s \rangle$, in the quark flavor basis. The decay $D^+_\mathrm{s} \rightarrow \omega e^{+} \nu _e$ occurs through $\phi$–$\omega$ mixing and/or Weak Annihilation (WA) diagrams, where the lepton pair couples weakly to the $c \bar{s}$ vertex. Experimentally, only an upper limit is available on the branching fraction $\mathcal{{B}}( D^+_\mathrm{s} \rightarrow \omega e^{+} \nu _e) <0.20~\%$, at 90 % C.L. [913].

Exclusive semileptonic $D$ decays also offer the chance to explore possible exotic states. An interesting channel is the $D^+_\mathrm{s} \rightarrow f_0(980) \, l^{+} \nu$ decay. The nontrivial nature of the experimentally well-established $f_0(980)$ state has been discussed for decades and there are still different interpretations, from the conventional quark–antiquark picture, to a multiquark or molecular bound state. The channels $D_{(s)}^{+} \rightarrow f_0(980) \, l^{+} \nu$ can be used as a probe of the hadronic structure of the light scalar resonance; more recent experimental investigation has been made available by CLEO [914]. A further handle is given by the possibility to correlate observables related to the charm semileptonic branching ratios with theoretical and experimental analyses of the hadronic $B_\mathrm{s} \rightarrow J/\psi f_0$ decay [914–916].

The most recent experimental results on inclusive $D^0$ and $D_{(s)}^+$ semileptonic branching fractions have been derived using the complete CLEO-c data sets [917]. Besides being important in their own right, these measurements, due to similarities between the $D$ and $B$ sectors, can be helpful to improve understanding of $B$ semileptonic decays, with the hope to reduce the theoretical uncertainty in the determination of the still-debated weak mixing parameter $|V_{ub}|$. In [917], knowledge about exclusive semileptonic modes and form factor models is used to extrapolate the spectra below the 200 MeV momentum cutoff. The ratios of the semileptonic decay widths are determined to be $\Gamma _{D^+}^{\mathrm {SL}}/\Gamma _{D^0}^{\mathrm {SL}} = 0.985 \pm 0.015 \pm 0.024$ and $\Gamma _{D^+_\mathrm{s}}^{\mathrm {SL}}/\Gamma _{D^0_\mathrm{s}}^{\mathrm {SL}} = 0.828 \pm 0.051 \pm 0.025$. The former agrees with isospin symmetry, while the latter ratio shows an indication of difference. Significant improvements of the branching ratio measurement $\mathcal{B} (D \rightarrow X \mu ^{+} \nu _{\mu })$ can be expected at BESIII, because of advantages provided by the capabilities of the BESIII $\mu$ detection system [918]. The $D^{0,\pm }$ and $D_\mathrm{s}$ inclusive decays are differently affected by the WA diagrams, since they are Cabibbo-suppressed in the $D^\pm$ case, Cabibbo-favored in $D_\mathrm{s}$ decays, and completely absent in $D^0$ decays. The semileptonic decays of $D$ and $D_\mathrm{s}$ can be helpful in constraining the WA matrix elements that enter the $B \rightarrow X_u \, l \bar{\nu }$ decay, via heavy quark symmetry. By comparison of measured total semileptonic rates or moments in these channels, we can hope to extract information on the WA contributions. The “theoretical background” to take into account is the fact that such contributions compete with additional ones arising from $\mathrm{SU}(3)$ breaking in the matrix elements, and/or from weak annihilation. However, no relevance or clear evidence of WA effects has been found considering the semileptonic widths [919] or the widths and the lepton–energy moments [920].

#### Exclusive $B$ decays

Most theoretical approaches exploit the fact that the mass $m_b$ of the $b$ quark is large compared to the QCD scale that determines low-energy hadronic physics in order to build differential ratios. Neglecting the charged lepton and neutrino masses, we can recast the differential ratios as

4.13 $$\begin{aligned}&\frac{d\Gamma }{d \omega } (\bar{B}\rightarrow D\,l \bar{\nu }) = \frac{G_\mathrm{F}^2}{48 \pi ^3}\, K_1\, (\omega ^2-1)^{\frac{3}{2}}\, |V_{cb}|^2 \mathcal{G}^2(\omega )\nonumber \\&\quad \frac{d\Gamma }{d \omega }(\bar{B}\rightarrow D^*\,l \bar{\nu }) = \frac{G_\mathrm{F}^2}{48 \pi ^3} K_2 (\omega ^2-1)^{\frac{1}{2}} |V_{cb}|^2 \mathcal{F}^2(\omega )\nonumber \\ \end{aligned}$$

where $$K_1= (m_B+m_D)^2 m_D^3 $$, $$K_2 = (m_B-m_{D^*})^2 m_{D^*}^3 \chi (\omega ) $$ and $$\chi (\omega )$$ is a known phase space. The semileptonic decays $$ \bar{B}\rightarrow D \, l \, \bar{\nu }$$ and $$ \bar{B}\rightarrow D^*\, l \, \bar{\nu }$$ depend on the form factors $$\mathcal{G}(\omega )$$ and $$\mathcal{F}(\omega )$$, respectively, where $$\omega $$ is the product of the heavy quark velocities $$v_B= p_B/m_B$$ and $$v_{D^{(*)}}= p_{D^{(*)}}/m_{D^{(*)}}$$. The form factors, in the heavy-quark limit, are both normalized to unity at the zero recoil point $$\omega =1$$. Corrections to this limit have been calculated in the lattice unquenched approximation, giving $$ \mathcal{G}(1) = 1.074 \pm 0.024 $$ [921] and $$ \mathcal{F}(1) =0.906 \pm 0.004 \pm 0.012 $$ [922], including the enhancement factor 1.007, due to the electroweak corrections to the four-fermion operator mediating the semileptonic decay.

The lattice calculations have been compared with non-lattice ones (see, e.g., Ref. [923]). By combining the heavy-quark expansion with a “BPS” expansion [924], in which $\mu _\pi ^2=\mu ^2_G$, the following value is quoted $\mathcal{G}(1) =1.04 \pm 0.02$. Recently, the value $\mathcal{F}(1) = 0.86 \pm 0.02$ [925, 926] has been calculated, using zero recoil sum rules, including full $\alpha _\mathrm{s}$ and estimated effects up to $1/m_Q^5$.

Since the zero recoil point is not accessible experimentally, due to the kinematical suppression of the differential decay rates, the $|V_{cb}|$ estimates rely on the extrapolation from $\omega \ne 0$ to the zero recoil point. In Table 6 we list the results of the $|V_{cb}|$ determinations obtained from the comparison of the previous form factors at zero recoil with experimental data. The errors are experimental and theoretical, respectively. The first three averages are taken by HFAG [927], the fourth one by PDG [1]. The slightly smaller values for the form factors in non-lattice determinations imply slightly higher values of $|V_{cb}|$. In the last line, we quote the result due to an alternative lattice determination, currently available only in the quenched approximation, which consists of calculating the form factor normalization directly at values $\omega >1$, avoiding the large extrapolation to $\omega =1$ and thus reducing the model dependence [928]. This approach, by using 2009 BaBar data [929], gives a slightly higher value than the unquenched lattice result. The errors are statistical, systematic and due to the theoretical uncertainty in the form factor $\mathcal{G}$, respectively. Calculations of form factors at non-zero recoil have been recently completed for $B \rightarrow D$ semileptonic decays, giving the value $|V_{cb}|=(38.5 \pm 1.9_\mathrm{exp+lat} \pm 0.2_\mathrm{QED}) \times 10^{-3}$ [930].

**Table 6:** Comparison of some exclusive determinations of $|V_{cb}|$

Theory	$|V_{cb}| \times 10^{3}$
$$ \bar{B}\rightarrow D^*\, l \, \bar{\nu }$$	
HFAG (Lattice) [922, 927, 931]	$39.04 \pm 0.49_{\mathrm {exp}} \pm 0.53_{\mathrm {QCD}}$
	$$\pm 0.19_{\mathrm {QED}}$$
HFAG (SR) [925–927]	$41.6\pm 0.6_{\mathrm {exp}}\pm 1.9_{\mathrm {th}}$
$$ \bar{B}\rightarrow D \, l \, \bar{\nu }$$	
HFAG (Lattice) [921, 927]	$39.70 \pm 1.42_{\mathrm {exp}} \pm 0.89_{\mathrm {th}}$
PDG (BPS) [1, 924]	$40.7 \pm 1.5_{\mathrm {exp}} \pm 0.8_{\mathrm {th}}$
BaBar (Lattice $\omega \ne 1$) [928, 929]	$41.6 \pm 1.8 \pm 1.4 \pm 0.7_{\mathrm{FF}}$

Until a few years ago, only exclusive decays where the final lepton was an electron or a muon had been observed, since decays into a $$\tau $$ lepton are suppressed because of the large $$\tau $$ mass. Moreover, these modes are very difficult to measure because of the multiple neutrinos in the final state, the low lepton momenta, and the large associated background contamination. Results of semileptonic decays with a $$\tau $$ in the final state were limited to inclusive and semi-inclusive measurements in LEP experiments. The first observation of an exclusive semileptonic $$B$$ decay was reported by the Belle Collaboration in 2007. They measured the branching fraction $$ \mathcal{{B}} (\bar{B}^0 \rightarrow D^{*+} \tau ^{-} \bar{\nu }_\tau )$$ [932]. Recently the BaBar Collaboration has published results of their measurements of $$B \rightarrow D^{(*)} \tau \nu $$ branching fractions normalized to the corresponding $$B \rightarrow D^{(*)} l \nu $$ modes (with $$l=e , \mu $$) by using the full BaBar data sample [933]. Their results are in agreement with measurements by Belle using $$657 \times 10^6$$ $$B \bar{B}$$ events [934], and indicate an enhancement of order $$(2 \sim 3) \sigma $$ above theoretical results within the SM. It will be interesting to compare with the final Belle results on these modes using the full data sample of $$772 \times 10^6$$ $$B \bar{B}$$ pairs together with improved hadronic tagging. Indeed, a similar deviation from the SM has been previously observed also in leptonic decays $$B^{-} \rightarrow \tau ^{-} \bar{\nu }_\tau $$, but Belle finds now a much lower value, in agreement with the SM, by using the full data set of $$B \bar{B}$$ events [935]. By using Belle data and the FLAG $$N_\mathrm{f}=2+1$$ determination of $$f_B$$, one obtains the value $$|V_{ub}| = (3.35 \pm 0.65 \pm 0.07) \times 10^{-3}$$ [876]. The accuracy is not sufficient to make this channel competitive for $$ |V_{ub}|$$ extraction, but the intriguing experimental situation has led to a reconsideration of SM predictions as well as exploring the possibility of new physics contributions, traditionally not expected in processes driven by the tree level semileptonic $$b$$ decay. (For more details see, e.g., Refs. [854, 936].)

The analysis of exclusive charmless semileptonic decays, in particular the $\bar{B} \rightarrow \pi l \bar{\nu }_l$ decay, is currently employed to determine the CKM parameter $|V_{ub}|$, which plays a crucial role in the study of the unitarity constraints. Also here, information about hadronic matrix elements is required via form factors. Recent $|V_{ub}|$ determinations have been reported by the BaBar collaboration; see Table VII of Ref. [937] (see also [853]), all in agreement with each other and with the value $|V_{ub}| = (3.25 \pm 0.31) \times 10^{-3}$, determined from the simultaneous fit to the experimental data and the lattice theoretical predictions [937]. They are also in agreement with the Belle results for $|V_{ub}| = (3.43 \pm 0.33) \times 10^{-3}$ extracted from the $\bar{B} \rightarrow \pi l \bar{\nu }_l$ decay channel [938] and for $|V_{ub}|$ from the $\bar{B} \rightarrow \rho l \bar{\nu }_l$ decay channel, with precision of twice as good as the world average [939].

Finally, we just mention that exclusive $B_\mathrm{s}$ decays are attracting a lot of attention due to the avalanche of recent data and to the expectation of new data. $B_\mathrm{s}$ physics has been, and is, the domain of Tevatron and LHCb, but also present and future $e^{+} e^{-}$ colliders can give their contribution, since the $\Upsilon \mathrm {(5S)}$ decays in about 20 % of the cases to $B_\mathrm{s}^{(\star )}$ meson-antimeson pairs. The measurement of the semileptonic asymmetry and its analysis are particularly interesting, since CP violation is expected to be tiny in the SM and any significant enhancement would be evidence for NP (see also [853, 940]).

#### Inclusive $B$ decays

In most of the phase space for inclusive $B \rightarrow X_q l \nu$ decays, long and short distance dynamics are factorized by means of the heavy quark expansion. However, the phase space region includes a region of singularity, also called endpoint or threshold region, plagued by the presence of large double (Sudakov-like) perturbative logarithms at all orders in the strong coupling.^8^ For $b \rightarrow c$ semileptonic decays, the effect of the small region of singularity is not very important; in addition, corrections are not expected as singular as in the $b \rightarrow u$ case, being cut off by the charm mass.

Recently, a global fit [927] has been performed to the width and all available measurements of moments in $B \rightarrow X_\mathrm{c} l \nu$ decays, yielding, in the kinetic scheme $|V_{cb}| = (41.88 \pm 0.73) \times 10^{-3}$ and in the 1S scheme $|V_{cb}| = (41.96 \pm 0.45) \times 10^{-3}$. Each scheme has its own non-perturbative parameters that have been estimated together with the charm and bottom masses. The inclusive averages are in good agreement with the values extracted from exclusive decays in Table 6, within the errors.

In principle, the method of extraction of $$|V_{ub}|$$ from inclusive $$ \bar{B} \rightarrow X_u l \bar{\nu }_l$$ decays follows in the footsteps of the $$|V_{cb}|$$ determination from $$ \bar{B} \rightarrow X_\mathrm{c} l \bar{\nu }_l$$, but the copious background from the $$ \bar{B} \rightarrow X_\mathrm{c} l \bar{\nu }_l$$ process, which has a rate about 50 times higher, limits the experimental sensitivity to restricted regions of phase space, where the background is kinematically suppressed. The relative weight of the threshold region, where the previous approach fails, increases and new theoretical issues need to be addressed. Latest results by Belle [947] and BaBar [948] access about $$ 90$$ % of the $$ \bar{B} \rightarrow X_u l \bar{\nu }_l$$ phase space. On the theoretical side, several approaches have been devised to analyze data in the threshold region, with differences in treatment of perturbative corrections and the parameterization of non-perturbative effects.

**Table 7:** Comparison of inclusive determinations of $|V_{ub}|$ [927]

Theory	$|V_{ub}| \times 10^{3}$
BLNP	$4.40 \pm 0.15^{+0.19}_{-0.21}$
DGE	$4.45 \pm 0.15^{+ 0.15}_{- 0.16}$
ADFR	$4.03 \pm 0.13^{+ 0.18}_{- 0.12}$
GGOU	$4.39 \pm 0.15^{ + 0.12}_ { -0.20}$

The average values for $|V_{ub}|$ have been extracted by HFAG [927] from the partial branching fractions, adopting a specific theoretical framework and taking into account correlations among the various measurements and theoretical uncertainties. In Table 7 we list some determinations, specifically the QCD theoretical calculations taking into account the whole set of experimental results, or most of it, starting from 2002 CLEO data [949]. They refer to the BLNP approach by Bosch, Lange, Neubert, and Paz [950], the GGOU one by Gambino, Giordano, Ossola and Uraltsev [951], the DGE one, the dressed gluon exponentiation, by Andersen and Gardi [952, 953] and the ADFR approach, by Aglietti, Di Lodovico, Ferrara, and Ricciardi, [954–956]. The results listed in Table 7 are consistent within the errors, but the theoretical uncertainty among determinations can reach 10 %. Other theoretical approaches have also been proposed in [957–959]. Notwithstanding all the experimental and theoretical efforts, the values of $|V_{ub}|$ extracted from inclusive decays remain about two $\sigma$ above the values given by exclusive determinations.

#### Rare charm decays

The decays driven by $c \rightarrow u l^{+} l^{-}$ are forbidden at tree level in the standard model (SM) and proceed via one-loop diagrams (box and penguin) at leading order in the electroweak interactions. Virtual quarks in the loops are of the down type, and no breaking due to the large top mass occurs. The GIM mechanism works more effectively in suppressing flavor (charm) changing neutral currents than their strangeness and beauty analogues, leading to tiny decay rates, dominated by long-distance effects. On the other side, we expect possible enhancements due to new physics to stand out, once we exclude potentially large long-distance SM contributions.

In the SM, a very low branching ratio has been estimated for inclusive decays, largely dominated by long-distance contributions ${ \mathcal B} (D \rightarrow X_u l^{+} l^{-}) = { \mathcal B}_\mathrm {LD} (D \rightarrow X_u l^{+} l^{-}) \sim O(10^{-6})$ [960]. Long-distance contributions are assumed to proceed from intermediate vector resonances such as $D \rightarrow X_u V$, $V \rightarrow l^+l^{-}$, where $V = \phi$, $\rho$ or $\omega$, which set the scale with branching fractions of order $10^{-6}$. Short-distance contributions lay far behind [960–962]; the latest estimate gives ${ \mathcal B}_\mathrm {SD} (D \rightarrow X_u e^{+} e^{-}) \sim 4 \,\times \, 10^{-9}$ [962]. Handling long-distance dynamics in these processes becomes equivalent to handling several intermediate charmless resonances, in a larger number than in the case of $B$ meson analogs. Their effect can be separated from short-distance contributions by applying selection criteria on the invariant mass of the leptonic pair.

To consider exclusive decays, let us start from $D_{(s)}^\pm \rightarrow h^\pm l^{+} l^{-}$, with $h \in (\pi , \rho , K, K^\star )$ and $l \in (e, \mu )$, none of which has been observed up to now. The best experimental limits on branching fractions are $O(10^{-6})$ or higher, at 90 % confidence level (CL), coming all from BaBar [963, 964], with a few exceptions: very old limits on $D^{+} \rightarrow \rho ^{+} \mu ^{+} \mu ^{-}$ and $D_\mathrm{s}^{+} \rightarrow K^{*+}(892) \mu ^{+} \mu ^{-}$ decays, given by E653 [965], and the recent limits on $D^{+}_{(s)} \rightarrow \pi ^{\pm } \mu ^\mp \mu ^+$ decays, given by LHCb with an integrated luminosity of 1.0 ${\mathrm {fb}}^{-1}$ [966]. The BESIII collaboration will be able to reach a sensitivity of $O(10^{-7})$ for $D^{+} \rightarrow K^+/\pi ^{+} \, l^{+} l^{-}$ at 90 % CL with a 20 fb$^{-1}$ data sample taken at the $\psi (3770)$ peak [918]. The LHCb collaboration can also search for $D_{(s)}^\pm \rightarrow h^\pm l^{+} l^{-}$ decays. The very recent update on the $D_{(s)}^{+} \rightarrow \pi ^{+} \mu ^{+} \mu ^{-}$ channel with a 3 ${\mathrm {fb}}^{-1}$ full data sample is still orders of magnitudes above the SM prediction; new searches for the $D_{(s)}^{+} \rightarrow K^{+} \mu ^{+} \mu ^{-}$ decays are ongoing [967]. Also decays $D^0 \rightarrow h^0 l^{+} l^{-}$ have not been observed yet; the best experimental limits at 90 % CL are of order $O(10^{-5})$ or higher, and are given by older analyses of CLEO [968], E653 [965] and E791 [969]. Future Super B factories are expected to reach a sensitivity of $O(10^{-8})$ on a 90 % CL on various rare decays, including $D^{+} \rightarrow \pi ^{+} l^{+} l^{-}$ and $D^0 \rightarrow \pi ^0 l^{+} l^{-}$ [970].

A way to disentangle possible new physics is to choose appropriate observables containing mainly short distance contributions. Last year, hints of possible new physics (NP) have been advocated in the charm sector to explain the nonvanishing direct CP asymmetry in $D^0 \rightarrow K^{+} K^{-}$ and $D^0 \rightarrow \pi ^{+} \pi ^{-}$, measured by LHCb [971], confirmed by CDF [972] and supported by recent data from Belle [973]. Encouraged by these results, effects of the same kind of possible NP have been looked for in other processes, including rare charm decays. CP asymmetries can be generated by imaginary parts of Wilson coefficients in the effective Hamiltonian for $c \rightarrow u l^{+} l^{-}$ driven decays. They have been investigated in $D^{+} \rightarrow \pi ^{+} \mu ^{+} \mu ^{-}$ and $D_\mathrm{s}^{+} \rightarrow K^{+} \mu ^{+} \mu ^{-}$ decays, around the $\phi$ resonance peak in the spectrum of dilepton invariant mass, concluding that in favorable conditions their value can be as high as 10 % [974]. Older studies report investigations of semileptonic decays in the framework of other NP models, such as R-parity violating supersymmetric models, extra heavy up vector-like quark models [975], Little Higgs [962], or leptoquark models [976]. The parameter space discussed in older analyses cannot take into account the constraints given by recent LHC data, most notably the discovery of the 125 GeV resonance. In several cases, a reassessment in the updated framework could be used advantageously.

### Spectroscopy

The year 2013 marks the 10th anniversary of the observation of the $X(3872)$ charmonium-like state [809] that put an end to the era when heavy quarkonium was considered as a relatively well established bound system of a heavy quark and antiquark. Since 2003 every year has been bringing discoveries of new particles with unexpected properties, not fitting a simple $q\bar{q}$ classification scheme. The wealth of new results is mainly from B- and c-factories, Belle, BaBar and BES III, where data samples with unprecedented statistics became available.

In this section we first describe experiments that contribute to the subject, discuss recent developments for low-lying states, then we move to the open flavor thresholds and beyond. We consider the charmonium- and bottomonium-(like) states in parallel to stress similarities between the observed phenomena in the two quarkonium sectors.

#### Experimental tools

Over the last decade the main suppliers of new information about quarkonium states have been the $B$-factories, the experiments working at asymmetric-energy $e^{+}e^{-}$ colliders operated at center-of-mass energies in the $\Upsilon$-resonance region. Both Belle and BaBar detectors are general-purpose 4$\pi$ spectrometers with excellent momentum resolution, vertex positioning and particle identification for charged tracks, as well as with high-resolution electromagnetic calorimeters. Although the main purpose of the $B$-factories is to study CP asymmetries in $B$-decays, these experiments allow for many other searches apart from the major goal. Charmonium states at $B$-factories are copiously produced in $B$-decays, two-photon fusion, charm quark fragmentation in $e^{+}e^{-}\rightarrow c\bar{c}$ annihilation (mostly via double $c\bar{c}$ production) and via initial-state radiation, when the energy of $e^{+}e^{-}$ annihilation is dumped by emission of photons in the initial state. Both $B$-factories intensively studied also bottomonium states, taking data at different $\Upsilon$ states that allow to access lower mass bottomonia via hadronic and radiative transitions. Although both $B$-factories completed their data taking already long ago (BaBar in 2008 and Belle in 2010), the analysis of the collected data is still ongoing, and many interesting results have been obtained recently. The data samples of the two experiments are summarized in Table 8.

**Table 8:** Integrated luminosities (in fb$^{-1}$) collected by the BaBar and Belle experiments at different $e^{+}e^{-}$ energies

	BaBar	Belle
$\Upsilon ({1}{S})$	–	5.7
$\Upsilon ({2}{S})$	14	24.1
$\Upsilon ({3}{S})$	30	3.0
$\Upsilon ({4}{S})$	433	711
Off-resonance	54	87
$\Upsilon ({5}{S})$	–	121
$\Upsilon ({5}{S})$- $\Upsilon ({6}{S})$ scan	5	27

Another class of experiments where charmonium states are extensively studied are the charm-$\tau$ factories. For the last decade BES II, CLEOc, and finally BES III have successively covered measurements of $e^{+}e^{-}$ annihilation around the charmonium region. The BES III experiment started data taking in 2009 after a major upgrade of the BEPC $e^{+}e^{-}$ collider and the BES II spectrometer. The BEPC II accelerator operates in the c.m. energy range of $\sqrt{s} = (2 - 4.6)~{\mathrm {GeV}}$ and has already reached a peak luminosity close to the designed one. Starting late 2012 BES III has collected data at high energies to study $Y(4260)$ and other highly excited charmonium-like states.

**Table 9:** Quarkonium states below the corresponding open flavor thresholds

State	$M,\,~\mathrm {MeV}$	$\Gamma ,\,~\mathrm {MeV}$	$J^{PC}$	Process (mode)	Experiment (#$\sigma$)	Year	Status
$\psi _2(1D)$	$3823.1\pm 1.9$	$<24$	$2^{-\,\!-}$	$B\rightarrow K(\gamma \,\chi _{c1})$	Belle [977] (3.8)	2013	NC!
$\eta _b(1S)$	$9398.0\pm 3.2$	$11^{+6}_{-4}$	$0^{-\,\!+}$	$\Upsilon (3S)\rightarrow \gamma \,(...)$	BaBar [978] (10), CLEO [979] (4.0)	2008	OK
				$\Upsilon (2S)\rightarrow \gamma \,(...)$	BaBar [980] (3.0)	2009	NC!
				$h_b(1P,2P)\rightarrow \gamma \,(...)$	Belle [848] (14)	2012	NC!
$h_b(1P)$	$9899.3\pm 1.0$	?	$1^{+\,\!-}$	$\Upsilon (10860)\rightarrow \pi ^{+}\pi ^{-}\,(...)$	Belle [848, 981] (5.5)	2011	NC!
				$\Upsilon (3S)\rightarrow \pi ^0\,(...)$	BaBar [982] (3.0)	2011	NC!
$\eta _b(2S)$	$9999\pm 4$	$<24$	$0^{-\,\!+}$	$h_b(2P)\rightarrow \gamma \,(...)$	Belle [848] (4.2)	2012	NC!
$\Upsilon (1D)$	$10163.7\pm 1.4$	?	$2^{-\,\!-}$	$\Upsilon (3S)\rightarrow \gamma \gamma \,(\gamma \gamma \,\Upsilon (1S))$	CLEO [983] (10.2)	2004	NC!
				$\Upsilon (3S)\rightarrow \gamma \gamma \,(\pi ^{+}\pi ^{-}\Upsilon (1S))$	BaBar [984] (5.8)	2010	NC!
				$\Upsilon (10860)\rightarrow \pi ^{+}\pi ^{-}(\gamma \gamma \,\Upsilon (1S))$	Belle [985] (9)	2012	NC!
$h_b(2P)$	$10259.8\pm 1.2$	?	$1^{+\,\!-}$	$\Upsilon (10860)\rightarrow \pi ^{+}\pi ^{-}\,(...)$	Belle [848, 981] (11.2)	2011	NC!
$\chi _{bJ}(3P)$	$10534\pm 9$	?	$(1,2)^{+\,\!+}$	$pp,p\bar{p}\rightarrow (\gamma \Upsilon (1S,2S))\,...$	ATLAS [986] ($>$6), D0 [987] (5.6)	2011	OK

Experiments at hadron machines (Tevatron and LHC) can investigate quarkonium produced promptly in high-energy hadronic collisions in addition to charmonium produced in $B$-decays. The Tevatron experiments CDF and D0 completed their experimental program in 2010, after CERN started operating the LHC. Four LHC experiments are complementary in tasks and design. While LHCb has been optimized for mainly heavy flavor physics, ATLAS and CMS are contributing to the field by investigating certain signatures in the central rapidity range with high statistics. The LHC accelerator performance has fulfilled and even exceeded expectations. The integrated luminosity delivered to the general-purpose experiments (ATLAS and CMS) in 2011 was about 6 fb$^{-1}$, and more than 20 fb$^{-1}$ in 2012. The instantaneous luminosity delivered to LHCb is leveled to a constant rate due to limitations in the LHCb trigger and readout, and to collect data under relatively clean conditions. The integrated luminosity delivered to LHCb was 1 fb$^{-1}$ and 2 fb$^{-1}$ in 2011 and 2012, respectively.

The new $B$ factory at KEK, SuperKEKB, will be commissioned in 2015 according to the current planning schedule. It is expected that the target integrated luminosity, 50 ab$^{-1}$ , will be collected by 2022.

#### Heavy quarkonia below open flavor thresholds

Recently, significant progress has been achieved in the studies of the spin-singlet bottomonium states. In addition, last year two more states have been found below their corresponding open flavor thresholds, the $\psi _2(1D)$ charmonium and the $\chi _b(3P)$ bottomonium (in the latter case the levels with different $J$ are not resolved), see Table 9. All these new data provide important tests of the theory, which, due to lattice and effective field theories, is rather solid and predictive below the open flavor threshold. The theory verification in this particular region becomes even more important given the difficulties of the theory for states near or above the open flavor threshold.

Spin-singlet bottomonium states do not have production or decay channels convenient for experimental studies. Therefore their discovery became possible only with the high statistics of the $B$-factories. An unexpected source of the spin-singlet states turned out to be the di-pion transitions from the $\Upsilon ({5}{S})$. The states are reconstructed inclusively using the missing mass of the accompanying particles. Belle observed the $h_{b}(1P)$ and $h_{b}(2P)$ states in the transitions $\Upsilon ({5}{S})\rightarrow {{\pi ^{+}\pi ^{-}}}h_{b}(nP)$ [981]. The hyperfine splittings were measured to be $(+0.8\pm 1.1)\,~\mathrm {MeV}$ for $n=1$ and $(+0.5\pm 1.2)\,~\mathrm {MeV}$ for $n=2$ [848]. The results are consistent with perturbative QCD expectations [988–991]. This shows in particular that the spin–spin potential does not have a sizeable long-range contribution [992], an observation supported by direct lattice computations [752]. For comparison, in the charmonium sector the measured $1P$ hyperfine splitting of $(-0.11\pm 0.17)\,~\mathrm {MeV}$ [1] is also consistent with zero with even higher accuracy.

The $\eta _b(1S)$ is found in M1 radiative transitions from $\Upsilon ({3}{S})$ [978, 979] and $\Upsilon ({2}{S})$ [980]. The measured averaged hyperfine splitting $\Delta M_\mathrm{HF}(1S)=M_{\Upsilon ({1}{S})}-M_{\eta _{b}(1S)}= (69.3\pm 2.8)~\mathrm {MeV}$ [1] was larger than perturbative pNRQCD $(41 \pm 14)\,~\mathrm {MeV}$ [743] and lattice $(60 \pm 8)\,~\mathrm {MeV}$ [850] estimates. In 2012, using a large sample of $h_{b}(mP)$ from $\Upsilon ({5}{S})$ Belle observed the $h_{b}(1P)\rightarrow {\eta _{b}(1S)}\gamma$ and $h_{b}(2P)\rightarrow {\eta _{b}(1S)}\gamma$ transitions [848]. The Belle ${\eta _{b}(1S)}$ mass measurement is more precise than the PDG2012 average and is $(11.4 \pm 3.6)\,~\mathrm {MeV}$ above the central value, which is in better agreement with the perturbative pNRQCD determination. The residual difference of about $17\,~\mathrm {MeV}$ is consistent with the uncertainty of the theoretical determination. Also lattice determinations have improved their analyses (see Sect. 4.1.3). The latest determination based on lattice NRQCD, which includes spin-dependent relativistic corrections through $O(v^6)$, radiative corrections to the leading spin-magnetic coupling, non-perturbative four-quark interactions and the effect of $u$, $d$, $s$ and $c$ quark vacuum polarization, gives $\Delta M_\mathrm{HF}(1S) = (62.8 \pm 6.7)\,~\mathrm {MeV}$ [846]. Belle measured for the first time also the ${\eta _{b}(1S)}$ width, $\Gamma _{{\eta _{b}(1S)}} = (10.8\,^{+4.0}_{-3.7}\,^{+4.5}_{-2.0})\,~\mathrm {MeV}$, which is consistent with expectations.

Belle found the first strong evidence for the ${\eta _{b}(2S)}$ with a significance of $4.4\,\sigma$ using the $h_{b}(2P)\rightarrow \gamma {\eta _{b}(2S)}$ transition. The hyperfine splitting was measured to be $\Delta M_\mathrm{HF}(2S)=(24.3^{+4.0}_{-4.5})\,~\mathrm {MeV}$. The ratio $\Delta M_\mathrm{HF}(2S)/ \Delta M_\mathrm{HF}(1S)=0.420^{+0.071}_{-0.079}$ is in agreement with NRQCD lattice calculations [843, 846, 850], the most recent of which gives $\Delta M_\mathrm{HF}(2S)/\Delta M_\mathrm{HF}(1S)=0.425\pm 0.025$ [846] (see also Sect. 4.1.3). The measured branching fractions $\mathcal {B}(h_{b}(1P)\rightarrow \gamma {\eta _{b}(1S)})=(49.2\pm 5.7\,^{+5.6}_{-3.3})~\%$, $\mathcal {B}(h_{b}(2P)\rightarrow \gamma {\eta _{b}(1S)})=(22.3\pm 3.8\,^{+3.1}_{-3.3})~\%$, and $\mathcal {B}(h_{b}(2P)\rightarrow \gamma {\eta _{b}(2S)})=(47.5\pm 10.5\,^{+6.8}_{-7.7})~\%$ are somewhat higher than the model predictions [993].

There is another claim of the ${\eta _{b}(2S)}$ signal by the group of K. Seth from Northwestern University, that used CLEO data [849]. The $\Upsilon ({2}{S})\rightarrow {\eta _{b}(2S)}\gamma$ production channel is considered and the ${\eta _{b}(2S)}$ is reconstructed in 26 exclusive channels with up to 10 charged tracks in the final state. The measured hyperfine splitting $\Delta M_\mathrm{HF}(2S)=(48.7\pm 3.1)\,~\mathrm {MeV}$ is $5\,\sigma$ away from the Belle value and is in strong disagreement with theoretical expectations [994]. In [849] the contribution of final-state radiation is not considered, therefore the background model is incomplete and the claimed significance of $4.6\,\sigma$ is overestimated. Belle repeated the same analysis with 17 times higher statistics and found no signal [995]. The Belle upper limit is an order of magnitude lower than the central value in [849]. We conclude that the evidence for the ${\eta _{b}(2S)}$ with the anomalous mass reported in [849] is refuted.

The $n=3$ radial excitation of the $\chi _{bJ}$ system was recently observed by ATLAS [986] and confirmed by D0 [987]. The $\chi _{bJ}(3P)$ states are produced inclusively in the $pp$ and $p\overline{p}$ collisions and are reconstructed in the $\gamma \Upsilon (1S,2S)$ channels with $\Upsilon \rightarrow \mu ^+\mu ^{-}$. Converted photons and photons reconstructed from energy deposits in the electromagnetic calorimeter are used. The mass resolution does not allow to discern individual $\chi _{bJ}(3P)$ states with $J=0$, 1 and 2. A measured barycenter of the triplet $10534\pm 9\,~\mathrm {MeV}$ is close to the quark model expectations of typically $10525\,~\mathrm {MeV}$ [996, 997].

Potential models predict that $D$-wave charmonium levels are situated between the $D \bar{D}$ and $D \bar{D}^{*}$ thresholds [998]. Among them the states $\eta _{c2}$ ($J^{\mathrm{PC}}=2^{-+}$) and $\psi _2$ ($J^{\mathrm{PC}}=2^{-\,\!-}$) cannot decay to $D \bar{D}$ because of unnatural spin–parity, and they are the only undiscovered charmonium levels that are expected to be narrow. Recently Belle reported the first evidence for the $\psi _2(1D)$ using the $B^+\rightarrow K^+\psi _2(1D)[\rightarrow \gamma \chi _{c1}]$ decays [977], with a mass of $M=(3823.1\pm 1.9)\,~\mathrm {MeV}$ and width consistent with zero, $\Gamma <24\,~\mathrm {MeV}$. The full width is likely to be very small, since the state is observed in the radiative decay and the typical charmonium radiative decay widths are at the $O(100)\,~{\mathrm {keV}}$ level. The odd $C$-parity (fixed by decay products) discriminates between the $\eta _{c2}$ and $\psi _2$ hypotheses. No signal is found in the $\gamma \chi _{c2}$ channel, in agreement with expectations for the $\psi _2$ [998]. Belle measured $\mathcal {B}(B^+\rightarrow K^+\psi _2)\times \mathcal {B}(\psi _2\rightarrow \gamma \chi _{c1})= (9.7{^{+2.8}_{-2.5}}{^{+1.1}_{-1.0}})\times 10^{-6}$. Given that one expects $\mathcal {B}(\psi _2\rightarrow \gamma \chi _{c1})\sim 2/3$ [998], $\mathcal {B}(B^+\rightarrow K^+\psi _2)$ is a factor of 50 smaller than the corresponding branching fractions for the $J/\psi$, $\psi (2S)$ and $\chi _{c1}$ due to the factorization suppression [999, 1000].

Many of the above studies and, in particular, many discovery channels involve radiative decays. For states below threshold, theory has made in the last few years remarkable progress in the study of these decay channels. From the EFT side, pNRQCD provides now an (almost) complete description of E1 and M1 transitions [1001, 1002], which means that we have expressions for all these decay channels up to and including corrections of relative order $v^2$. The only exception are M1 transitions for strongly bound quarkonia that depend at order $v^2$ on a not-yet-calculated Wilson coefficient. The kind of insight in the QCD dynamics of quarkonia that one may get from having analytical expressions for these decay rates can be understood by looking at the transition $J/\psi \rightarrow \eta _\mathrm{c}(1S)\gamma$. The PDG average for the width $\Gamma (J/\psi \rightarrow \eta _\mathrm{c}(1S)\gamma )$ is $(1.58 \pm 0.37) \,~{\mathrm {keV}}$, which is clearly lower than the leading order estimate $2.83\,~{\mathrm {keV}}$. Corrections of relative order $v^2$ are positive in the case of a confining potential, whereas they are negative in the case of a Coulomb potential [1001]. Therefore the current PDG average favors an interpretation of the $J/\psi$ as a Coulombic bound state. This interpretation may be challenged by the most recent KEDR analysis that finds $\Gamma (J/\psi \rightarrow \eta _\mathrm{c}(1S)\gamma ) = (2.98 \pm 0.18^{+0.15}_{-0.33})\,\mathrm{keV}$ [1003]. The KEDR result has a better accuracy than the current world average and is $3.0\,\sigma$ above its central value.

In [1004], a determination of $\Gamma (J/\psi \rightarrow \eta _\mathrm{c}(1S)\gamma )$ based on lattice QCD in the continuum limit with two dynamical quarks, the authors find $\Gamma (J/\psi \rightarrow \eta _\mathrm{c}(1S)\gamma ) = (2.64\pm 0.11\pm 0.03)\,~{\mathrm {keV}}$. Earlier lattice determinations of the charmonium radiative transitions in quenched lattice QCD can be found in [1005, 1006]. In [1007], a determination of $\Gamma (J/\psi \rightarrow \eta _\mathrm{c}(1S)\gamma )$ in perturbative pNRQCD, the authors find $\Gamma (J/\psi \rightarrow \eta _\mathrm{c}(1S)\gamma ) = (2.12\pm 0.40) \,\mathrm{keV}$. Both theoretical determinations are consistent with each other and fall in between the PDG average and the latest KEDR determination with the lattice determination favoring a somewhat larger value and the perturbative QCD determination a somewhat smaller value of the transition width. Part of the tension between data, and between data and theoretical determinations may be due to the fact that the extraction of the $J/\psi \rightarrow \eta _\mathrm{c}(1S)\gamma$ branching fraction from the photon energy line shape in $J/\psi \rightarrow X\gamma$ is not free from uncontrolled uncertainties [1008].

**Table 10:** Quarkonium-like states at the open flavor thresholds. For charged states, the $C$-parity is given for the neutral members of the corresponding isotriplets

State	$M,\,~\mathrm {MeV}$	$\Gamma ,\,~\mathrm {MeV}$	$J^{PC}$	Process (mode)	Experiment (#$\sigma$)	Year	Status
$X(3872)$	$3871.68\pm 0.17$	$<\!1.2$	$1^{+\,\!+}$	$B\rightarrow K(\pi ^{+}\pi ^{-}J/\psi )$	Belle [809, 1029] ($>$10), BaBar [1030] (8.6)	2003	OK
				$p\bar{p}\rightarrow (\pi ^{+}\pi ^{-}J/\psi )\,...$	CDF [1031, 1032] (11.6), D0 [1033] (5.2)	2003	OK
				$pp\rightarrow (\pi ^{+}\pi ^{-}J/\psi )\,...$	LHCb [1034, 1035] (np)	2012	OK
				$B\rightarrow K(\pi ^{+}\pi ^{-}\pi ^0J/\psi )$	Belle [1036] (4.3), BaBar [1037] (4.0)	2005	OK
				$B\rightarrow K(\gamma \, J/\psi )$	Belle [1038] (5.5), BaBar [1039] (3.5)	2005	OK
					LHCb [1040] ($>10$)		
				$B\rightarrow K(\gamma \, \psi (2S))$	BaBar [1039] (3.6), Belle [1038] (0.2)	2008	NC!
					LHCb [1040] (4.4)		
				$B\rightarrow K(D\bar{D}^{*})$	Belle [1041] (6.4), BaBar [1042] (4.9)	2006	OK
$Z_{c}(3885)^{+}$	$3883.9\pm 4.5$	$25\pm 12$	$1^{+\,\!-}$	$Y(4260)\rightarrow \pi ^{-}(D\bar{D}^{*})^{+}$	BES III [1043] (np)	2013	NC!
$Z_{c}(3900)^{+}$	$3891.2\pm 3.3$	$40\pm 8$	$?^{?-}$	$Y(4260)\rightarrow \pi ^{-}(\pi ^{+}J/\psi )$	BES III [1044] (8), Belle [1045] (5.2)	2013	OK
					T. Xiao et al. [CLEO data] [1046] ($>$5)		
$Z_{c}(4020)^{+}$	$4022.9\pm 2.8$	$7.9\pm 3.7$	$?^{?-}$	$Y(4260,4360)\rightarrow \pi ^{-}(\pi ^{+}h_{c})$	BES III [1047] (8.9)	2013	NC!
$Z_{c}(4025)^{+}$	$4026.3\pm 4.5$	$24.8\pm 9.5$	$?^{?-}$	$Y(4260)\rightarrow \pi ^{-}(D^{*}\bar{D}^{*})^{+}$	BES III [1048] (10)	2013	NC!
$Z_b(10610)^{+}$	$10607.2\pm 2.0$	$18.4\pm 2.4$	$1^{+\,\!-}$	$\Upsilon (10860)\rightarrow \pi (\pi \Upsilon (1S,2S,3S))$	Belle [1049–1051] ($>$10)	2011	OK
				$\Upsilon (10860)\rightarrow \pi ^{-}(\pi ^{+}h_b(1P,2P))$	Belle [1050] (16)	2011	OK
				$\Upsilon (10860)\rightarrow \pi ^{-}(B\bar{B}^{*})^{+}$	Belle [1052] (8)	2012	NC!
$Z_b(10650)^{+}$	$10652.2\pm 1.5$	$11.5\pm 2.2$	$1^{+\,\!-}$	$\Upsilon (10860)\rightarrow \pi ^{-}(\pi ^{+}\Upsilon (1S,2S,3S))$	Belle [1049, 1050] ($>$10)	2011	OK
				$\Upsilon (10860)\rightarrow \pi ^{-}(\pi ^{+}h_b(1P,2P))$	Belle [1050] (16)	2011	OK
				$\Upsilon (10860)\rightarrow \pi ^{-}(B^{*}\bar{B}^{*})^{+}$	Belle [1052] (6.8)	2012	NC!

Bottomonium M1 transitions have been studied in perturbative pNRQCD in [1001] and [1007]. In particular, in [1007] a class of large perturbative contributions associated with the static potential has been resummed providing an improved determination of several M1 transitions: $\Gamma ({\Upsilon (1S) \rightarrow \eta _b(1S)\gamma }) = (15.18 \pm 0.51) \,\mathrm{eV}$, $\Gamma (h_b(1P) \rightarrow \chi _{b0}(1P)\gamma ) = (0.962\pm 0.035) \,\mathrm{eV}$, $\Gamma ({h_b(1P) \rightarrow \chi _{b1}(1P)\gamma }) = (8.99\pm 0.55) \times 10^{-3}\,\mathrm{eV}$, $\Gamma ({\chi _{b2}(1P) \rightarrow h_b(1P)\gamma }) = (0.118\pm 0.006) \,\mathrm{eV}$ and $\Gamma ({\Upsilon (2S) \rightarrow \eta _b(1S)\gamma }) =6^{+26}_{-6} \, \mathrm{eV}$. The improved determination of $\Gamma ({\Upsilon (2S) \rightarrow \eta _b(1S)\gamma })$ is particularly noteworthy because it is consistent with the most recent data, $(12.5\pm 4.9)\,\mathrm{eV}$ from BaBar [980], while the leading-order determination is off by at least one order of magnitude. Bottomonium transitions in lattice NRQCD with $2+1$ dynamical quarks have been computed in [1009, 1010].

E1 transitions are more difficult to study both on the lattice and with analytical methods. The reason is that even at leading order they involve a non-perturbative matrix element. A complete theoretical formulation in the framework of pNRQCD can be found in [1002] with a preliminary but promising phenomenological analysis in [1011].

The theoretical status of quarkonium hadronic transitions, inclusive and exclusive hadronic and electromagnetic decays has been summarized in [757, 1012, 1013]. There has been a limited use of the pNRQCD factorization for these processes and only restricted to inclusive hadronic and electromagnetic decays [744, 745, 748, 749, 1014, 1015], while most of the recent work has concentrated on improving the expansion in the NRQCD factorization framework to higher orders in $v$ and $\alpha _\mathrm{s}$ [1016–1028].

#### Quarkonium-like states at open flavor thresholds

There are several states in both the charmonium and bottomonium sectors lying very close to the threshold of their decay to a pair of open flavor mesons; see Table 10. This proximity suggests a molecular structure for these states.

The $X(3872)$ is a state very close to the $D^{*0} \bar{ D^{0}}$threshold, $\delta m_{X(3872)}=m_{X(3872)}-m_{D^{*0}}-m_{D^0}=-0.11\pm 0.22\,~\mathrm {MeV}$ [1, 1053, 1054]. The decays $X(3872)\rightarrow \rho J/\psi$ and $X(3872)\rightarrow \omega J/\psi$ have similar branching fractions, $\mathcal {B}_{\omega }/ \mathcal {B}_{\rho } =0.8\pm 0.3$ [1036, 1037]; this corresponds to a strong isospin violation. The favorite $X(3872)$ interpretation is a mixture of a charmonium state $\chi _{c1}(2P)$ and an $S$-wave $D^{*0} \bar{ D^{0}}$molecule [759–764, 1055–1074], with the molecular component responsible for the isospin violation and the charmonium component accounting for the production in $B$ decays and at hadron collisions. For alternative interpretations we refer to [757] and references therein. The molecular hypothesis is valid only for the spin–parity assignment $J^{\mathrm{PC}}=1^{+\,\!+}$. Experimentally $1^{+\,\!+}$ was favored, but $2^{-+}$ was not excluded for a long time [1029, 1031, 1075]. This intrigue has been settled recently by LHCb with a clear preference of $1^{+\,\!+}$ and exclusion of $2^{-+}$ hypothesis [1035]. Progress towards a lattice understanding of the $X(3872)$ has been discussed in Sect. 4.1.3.

The contributions to the $\delta m_{X(3872)}$ uncertainty are $0.17\,~\mathrm {MeV}$ from the $X(3872)$ mass, $0.13\,~\mathrm {MeV}$ from the $D^0$ mass and $0.07\,~\mathrm {MeV}$ from the $D^{*0}-D^0$ mass difference. LHCb can improve the accuracy in $m_{X(3872)}$ and $m_{D^0}$, BES III and KEDR can contribute to the $m_{D^0}$ measurement. The $D^{*0}-D^0$ mass difference was measured 20 years ago by ARGUS and CLEO and also can be improved.

The radiative $X(3872)\rightarrow \gamma J/\psi$ decay is established, while there is an experimental controversy regarding $X(3872) \rightarrow \gamma \psi (2S)$ [1036, 1038, 1039], with recent LHCb evidence pointing towards existence of this channel [1040]. The dominant decay mode of the $X(3872)$ is $D^{*0} \bar{ D^{0}}$[1041, 1042, 1076], as expected for the molecule, however, the absolute branching fraction is not yet determined. These questions will have to wait for Belle II data.

Charged bottomonium-like states $Z_{b}(10610)$ and $Z_{b}(10650)$ are observed by Belle as intermediate $\Upsilon ({n}{S})\pi ^{\pm }$ and $h_b(mP)\pi ^{\pm }$ resonances in the $\Upsilon ({5}{S})\rightarrow \pi ^+\pi ^{-}\Upsilon ({n}{S})$ and $\Upsilon ({5}{S})\rightarrow \pi ^+\pi ^{-} h_b(mP)$ decays [1050]. The nonresonant contribution is sizable for the $\Upsilon ({5}{S})\rightarrow \pi ^+\pi ^{-}\Upsilon ({n}{S})$ decays and is consistent with zero for the $\Upsilon ({5}{S})\rightarrow \pi ^+\pi ^{-}h_b(mP)$ decays. The mass and width of the $Z_{b}$ states were measured from the amplitude analysis, assuming a Breit–Wigner form of the $Z_{b}$ amplitude. The parameters are in agreement among all five decay channels, with the average values $M_1=(10607.4\pm 2.0)\,~\mathrm {MeV}$, $\Gamma _1=(18.4\pm 2.4)\,~\mathrm {MeV}$ and $M_2=(10652.2\pm 1.5)\,~\mathrm {MeV}$, $\Gamma _2=(11.5\pm 2.2)\,~\mathrm {MeV}$. The measured $Z_{b}(10610)$ and $Z_{b}(10650)$ masses coincide within uncertainties with the $B\bar{B}^*$ and $B^*\bar{B}^*$ thresholds, respectively.

Belle observed the $Z_{b}(10610)\rightarrow B\bar{B}^*$ and $Z_{b}(10650)\rightarrow B^*\bar{B}^*$ decays with the significances of $8\,\sigma$ and $6.8\,\sigma$, respectively, using the partially reconstructed $\Upsilon ({5}{S})\rightarrow (B^{(*)}\bar{B}^*)^{\pm }\pi ^{\mp }$ transitions [1052]. Despite much larger phase space, no significant signal of the $Z_{b}(10650)\rightarrow B\bar{B}^*$ decay was found. Assuming that the decays observed so far saturate the $Z_b$ decay table, Belle calculated the relative branching fractions of $Z_{b}(10610)$ and $Z_{b}(10650)$ (Table 11).

**Table 11:** Branching fractions ($\mathcal {B}$) of $Z_{b}$s in per cent

Channel	$\mathcal {B}$ of $Z_{b}(10610)$	$\mathcal {B}$ of $Z_{b}(10650)$
$\pi ^{+}\Upsilon ({1}{S})$	$0.32\pm 0.09$	$0.24\pm 0.07$
$\pi ^{+}\Upsilon ({2}{S})$	$4.38\pm 1.21$	$2.40\pm 0.63$
$\pi ^{+}\Upsilon ({3}{S})$	$2.15\pm 0.56$	$1.64\pm 0.40$
$\pi ^{+}h_{b}(1P)$	$2.81\pm 1.10$	$7.43\pm 2.70$
$\pi ^{+}h_{b}(2P)$	$4.34\pm 2.07$	$14.8\pm 6.22$
$B^{+}\bar{B}^{*0}+\bar{B}^0B^{*+}$	$86.0\pm 3.6$	–
$B^{*+}\bar{B}^{*0}$	–	$73.4\pm 7.0$

The $Z_{b}(10610)\rightarrow B\bar{B}^*$ and $Z_{b}(10650)\rightarrow B^*\bar{B}^*$ decays are dominant with a branching fraction of about 80 %. If the $Z_{b}(10650)\rightarrow B\bar{B}^*$ channel is included in the decay table, its branching fraction is $\mathcal {B}(Z_{b}(10650)\rightarrow B\bar{B}^*)=(25\pm 10)\,~\%$ and all other $Z_{b}(10650)$ branching fractions are reduced by a factor of 1.33.

Belle observed the neutral member of the $Z_b(10610)$ isotriplet by performing a Dalitz analysis of the $\Upsilon ({5}{S})\rightarrow \pi ^0\pi ^0\Upsilon ({n}{S})$ ($n=1,2,3$) decays [1051]. The $Z_b(10610)^0$ significance combined over the $\pi ^0\Upsilon ({2}{S})$ and $\pi ^0\Upsilon ({3}{S})$ channels is $6.5\,\sigma$. The measured mass value $M_{Z_b(10610)^0}=(10609\pm 6)\,~\mathrm {MeV}$ is in agreement with the mass of the charged $Z_{b}(10610)^{\pm }$. No significant signal of the $Z_b(10650)^0$ is found; the data are consistent with the existence of the $Z_b(10650)^0$ state, but the available statistics are insufficient to observe it.

To determine the spin and parity of the $Z_b$ states, Belle performed a full six-dimensional amplitude analysis of the $\Upsilon (5S)\rightarrow \pi ^+\pi ^{-}\Upsilon (nS)$ $(n=1,2,3)$ decays [1077]. The $Z_b(10610)$ and $Z_b(10650)$ are found to have the same spin–parity $J^P=1^+$, while all other hypotheses with $J\le 2$ are rejected at more than $10\,\sigma$ level. The highest discriminating power is provided by the interference pattern between the $Z_b$ signals and the nonresonant contribution.

The proximity to the $B\bar{B}^*$ and $B^*\bar{B}^*$ thresholds suggests that the $Z_{b}$ states have molecular structure, i.e., their wave function at large distances is the same as that of an S-wave meson pair in the $I^G(J^P)=1^+(1^+)$ state [1078].

The assumption of the molecular structure can naturally explain all the properties of the $Z_{b}$ states without specifying their dynamical model [1078]. The decays into constituents [i.e. $Z_{b}(10610)\rightarrow B\bar{B}^*$ and $Z_{b}(10650)\rightarrow B^*\bar{B}^*$], if kinematically allowed, should dominate. The molecular spin function, once decomposed into $b\bar{b}$ spin eigenstates, appears to be a mixture of the ortho- and para-bottomonium components. The weights of the components are equal, therefore the decays into $\pi \Upsilon$ and $\pi h_b$ have comparable widths. The $B\bar{B}^*$ and $B^*\bar{B}^*$ states differ by a sign between the ortho- and para-bottomonium components. This sign difference is observed in the interference pattern between the $Z_{b}(10610)$ and $Z_{b}(10650)$ signals in the $\pi \Upsilon$ and $\pi h_b$ final states [1050].

The question of the dynamical model of the molecules remains open. Among different possibilities are nonresonant rescattering [1079], multiple rescatterings that result in a pole in the amplitude, known as a coupled channel resonance [1080], and deuteron-like molecule bound by meson exchanges [1081]. All these mechanisms are closely related and a successful phenomenological model should probably account for all of them. Predictions for the $Z_b$ lineshapes that can be used to fit data would be useful to discriminate between different mechanisms.

Alternatively, the $Z_{b}$ states are proposed to have diquark–antidiquark structure [1082]. In this model the $B^{(*)}\bar{B}^*$ channels are not dominant and the lighter (heavier) state couples predominantly to $B^*\bar{B}^*$ ($B\bar{B}^*$). The data on the decay pattern of the $Z_{b}$ states strongly disfavor the diquark–antidiquark hypothesis.

Observation of the charged $Z_b$ states motivated a search for their partners in the charm sector. Since late 2012 BES III has been collecting data at different energies above $4\,~{\mathrm {GeV}}$ to study charmonium-like states.

In the course of 2013 the states $Z_\mathrm{c}(3885)^{\pm }\rightarrow (D\bar{D}^*)^{\pm }$, $Z_\mathrm{c}(3900)^{\pm }\rightarrow \pi ^{\pm }J/\psi$, $Z_\mathrm{c}(4020)\rightarrow \pi ^{\pm }h_\mathrm{c}$, $Z_\mathrm{c}(4025)\rightarrow (D^*\bar{D}^*)^{\pm }$ were observed (see Table 10). The masses and widths of the $Z_\mathrm{c}(3885)$/$Z_\mathrm{c}(3900)$ and $Z_\mathrm{c}(4020)$/$Z_\mathrm{c}(4025)$ pairs agree at about $2\,\sigma$ level. All current measurements disregard the interference between the $Z_\mathrm{c}$ signal and the nonresonant contribution, which is found to be significant in all channels (including $\pi h_\mathrm{c}$, in contrast to the $\pi h_b$ case). Interference effects could shift the peak position by as much as half the resonance width. A more accurate measurement of masses and widths as well as spins and parities using the amplitude analyses will help to clarify whether the above $Z_\mathrm{c}$ pairs could be merged.

The $Z_\mathrm{c}(3885)$ and $Z_\mathrm{c}(3900)$ [$Z_\mathrm{c}(4020)$ and $Z_\mathrm{c}(4025)$] states are close to the $D\bar{D}^*$ [$D^*\bar{D}^*$] threshold. In fact, all the measured masses are about $10\,~\mathrm {MeV}$ *above* the thresholds. This is a challenge for a molecular model, but could be an experimental artifact caused by neglecting the interference.

If the $Z_\mathrm{c}(3885)$ and $Z_\mathrm{c}(3900)$ states are merged, the properties of the resulting state agree with the expectations for the $D\bar{D}^*$ molecular structure. The $D\bar{D}^*$ channel is dominant [1043],

4.14 $$\begin{aligned} \frac{\Gamma [Z_\mathrm{c}(3885)^{\pm }\rightarrow (D\bar{D}^*)^{\pm }]}{\Gamma [Z_\mathrm{c}(3900)^{\pm }\rightarrow \pi ^{\pm }J/\psi ]}=6.2\pm 2.9. \end{aligned}$$

A $2.1\,\sigma$ hint for the $Z_\mathrm{c}(3900)\rightarrow \pi ^{\pm }h_\mathrm{c}$ transition [1047] implies that the state couples to both ortho- and para-charmonium, with a weaker $\pi h_\mathrm{c}$ signal due to phase-space suppression. Finally, the spin–parity measured for the $Z_\mathrm{c}(3885)$ $J^P=1^+$ corresponds to $D\bar{D}^*$ in S-wave.

Identification of the $Z_\mathrm{c}(4020)$ or $Z_\mathrm{c}(4025)$ as a $D^*\bar{D}^*$ molecule is difficult. If the $D\bar{D}^*$ molecule decays to $\pi ^{\pm }J/\psi$, then according to heavy-quark spin symmetry the $D^*\bar{D}^*$ molecule should also decay to $\pi ^{\pm }J/\psi$. However, no hint of $Z_\mathrm{c}(4020)$ or $Z_\mathrm{c}(4025)$ is seen in the $\pi ^{\pm }J/\psi$ final state.

It could be that the $D^*\bar{D}^*$ molecule is not produced in the $Y(4260)$ decays, as would be the case if the $Y(4260)$ is a $D_1(2420)\bar{D}$ molecule (see next section).

**Table 12:** Quarkonium-like states above the corresponding open flavor thresholds. For charged states, the $C$-parity is given for the neutral members of the corresponding isotriplets

State	$M,\,~\mathrm {MeV}$	$\Gamma ,\,~\mathrm {MeV}$	$J^{PC}$	Process (mode)	Experiment (#$\sigma$)	Year	Status
$Y(3915)$	$3918.4\pm 1.9$	$20\pm 5$	$0/2^{?+}$	$B\rightarrow K(\omega J/\psi )$	Belle [1087] (8), BaBar [1037, 1088] (19)	2004	OK
				$e^{+}e^{-}\rightarrow e^{+}e^{-}(\omega J/\psi )$	Belle [1089] (7.7), BaBar [1090] (7.6)	2009	OK
$\chi _{c2}(2P)$	$3927.2\pm 2.6$	$24\pm 6$	$2^{+\,\!+}$	$e^{+}e^{-}\rightarrow e^{+}e^{-}(D\bar{D})$	Belle [1091] (5.3), BaBar [1092] (5.8)	2005	OK
$X(3940)$	$3942^{+9}_{-8}$	$37^{+27}_{-17}$	$?^{?+}$	$e^{+}e^{-}\rightarrow J/\psi \,(D\bar{D}^{*})$	Belle [1085, 1086] (6)	2005	NC!
$Y(4008)$	$3891\pm 42$	$255\pm 42$	$1^{-\,\!-}$	$e^{+}e^{-}\rightarrow (\pi ^{+}\pi ^{-}J/\psi )$	Belle [1045, 1093] (7.4)	2007	NC!
$\psi (4040)$	$4039\pm 1$	$80\pm 10$	$1^{-\,\!-}$	$e^{+}e^{-}\rightarrow (D^{(*)}\bar{D}^{(*)}(\pi ))$	PDG [1]	1978	OK
				$e^{+}e^{-}\rightarrow (\eta J/\psi )$	Belle [1094] (6.0)	2013	NC!
$Z(4050)^{+}$	$4051^{+24}_{-43}$	$82^{+51}_{-55}$	$?^{?+}$	$\bar{B}^0\rightarrow K^{-}(\pi ^{+}\chi _{c1})$	Belle [1095] (5.0), BaBar [1096] (1.1)	2008	NC!
$Y(4140)$	$4145.8\pm 2.6$	$18\pm 8$	$?^{?+}$	$B^{+}\rightarrow K^{+}(\phi J/\psi )$	CDF [1097] (5.0), Belle [1098] (1.9),	2009	NC!
					LHCb [1099] (1.4), CMS [1100] ($>$5)		
					D0 [1101] (3.1)		
$\psi (4160)$	$4153\pm 3$	$103\pm 8$	$1^{-\,\!-}$	$e^{+}e^{-}\rightarrow (D^{(*)}\bar{D}^{(*)})$	PDG [1]	1978	OK
				$e^{+}e^{-}\rightarrow (\eta J/\psi )$	Belle [1094] (6.5)	2013	NC!
$X(4160)$	$4156^{+29}_{-25}$	$139^{+113}_{-65}$	$?^{?+}$	$e^{+}e^{-}\rightarrow J/\psi \,(D^{*}\bar{D}^{*})$	Belle [1086] (5.5)	2007	NC!
$Z(4200)^{+}$	$4196^{+35}_{-30}$	$370^{+99}_{-110}$	$1^{+\,\!-}$	$\bar{B}^0\rightarrow K^{-}(\pi ^{+}J/\psi )$	Belle [1102] (7.2)	2014	NC!
$Z(4250)^{+}$	$4248^{+185}_{-45}$	$177^{+321}_{-72}$	$?^{?+}$	$\bar{B}^0\rightarrow K^{-}(\pi ^{+}\chi _{c1})$	Belle [1095] (5.0), BaBar [1096] (2.0)	2008	NC!
$Y(4260)$	$4250\pm 9$	$108\pm 12$	$1^{-\,\!-}$	$e^{+}e^{-}\rightarrow (\pi \pi J/\psi )$	BaBar [1103, 1104] (8), CLEO [1105, 1106] (11)	2005	OK
					Belle [1045, 1093] (15), BES III [1044] (np)		
				$e^{+}e^{-}\rightarrow (f_0(980)J/\psi )$	BaBar [1104] (np), Belle [1045] (np)	2012	OK
				$e^{+}e^{-}\rightarrow (\pi ^{-}Z_{c}(3900)^{+})$	BES III [1044] (8), Belle [1045] (5.2)	2013	OK
				$e^{+}e^{-}\rightarrow (\gamma \,X(3872))$	BES III [1107] (5.3)	2013	NC!
$Y(4274)$	$4293\pm 20$	$35\pm 16$	$?^{?+}$	$B^{+}\rightarrow K^{+}(\phi J/\psi )$	CDF [1097] (3.1), LHCb [1099] (1.0),	2011	NC!
					CMS [1100] ($>$3), D0 [1101] (np)		
$X(4350)$	$4350.6^{+4.6}_{-5.1}$	$13^{+18}_{-10}$	$0/2^{?+}$	$e^{+}e^{-}\rightarrow e^{+}e^{-}(\phi J/\psi )$	Belle [1108] (3.2)	2009	NC!
$Y(4360)$	$4354\pm 11$	$78\pm 16$	$1^{-\,\!-}$	$e^{+}e^{-}\rightarrow (\pi ^{+}\pi ^{-}\psi (2S))$	Belle [1109] (8), BaBar [1110] (np)	2007	OK
$Z(4430)^{+}$	$4458\pm 15$	$166^{+37}_{-32}$	$1^{+\,\!-}$	$\bar{B}^0\rightarrow K^{-}(\pi ^{+}\psi (2S))$	Belle [1111, 1112] (6.4), BaBar [1113] (2.4)	2007	OK
					LHCb [1114] (13.9)		
				$\bar{B}^0\rightarrow K^{-}(\pi ^{+}J/\psi )$	Belle [1102] (4.0)	2014	NC!
$X(4630)$	$4634^{+9}_{-11}$	$92^{+41}_{-32}$	$1^{-\,\!-}$	$e^{+}e^{-}\rightarrow (\Lambda _{c}^{+}\bar{\Lambda }_{c}^{-})$	Belle [1115] (8.2)	2007	NC!
$Y(4660)$	$4665\pm 10$	$53\pm 14$	$1^{-\,\!-}$	$e^{+}e^{-}\rightarrow (\pi ^{+}\pi ^{-}\psi (2S))$	Belle [1109] (5.8), BaBar [1110] (5)	2007	OK
$\Upsilon (10860)$	$10876\pm 11$	$55\pm 28$	$1^{-\,\!-}$	$e^{+}e^{-}\rightarrow (B_{(s)}^{(*)}\bar{B}_{(s)}^{(*)}(\pi ))$	PDG [1]	1985	OK
				$e^{+}e^{-}\rightarrow (\pi \pi \Upsilon (1S,2S,3S))$	Belle [1050, 1051, 1116] ($>$10)	2007	OK
				$e^{+}e^{-}\rightarrow (f_0(980)\Upsilon (1S))$	Belle [1050, 1051] ($>$5)	2011	OK
				$e^{+}e^{-}\rightarrow (\pi Z_b(10610,10650))$	Belle [1050, 1051] ($>$10)	2011	OK
				$e^{+}e^{-}\rightarrow (\eta \Upsilon (1S,2S))$	Belle [985] (10)	2012	OK
				$e^{+}e^{-}\rightarrow (\pi ^{+}\pi ^{-}\Upsilon (1D))$	Belle [985] (9)	2012	OK
$Y_b(10888)$	$10888.4\pm 3.0$	$30.7^{+8.9}_{-7.7}$	$1^{-\,\!-}$	$e^{+}e^{-}\rightarrow (\pi ^{+}\pi ^{-}\Upsilon (nS))$	Belle [1117] (2.3)	2008	NC!

The $Z_\mathrm{c}(4020)$ could be a candidate for hadrocharmonium, a color-neutral quarkonium state in a cloud of light meson(s) [1083]. The decay into constituent charmonium and light meson should dominate, while the decay to another charmonium is suppressed. The available experimental information on $Z_\mathrm{c}(4020)$ agrees with this picture. The $Z_\mathrm{c}(4025)$ is not a suitable hadrocharmonium candidate since the $D^*\bar{D}^*$ channel dominates. Hadrocharmonium was proposed to explain the affinity of many charmonium-like states [$Y(4260)$, $Y(4360)$, $Z(4050$, $Z(4250)$, $Z(4430)$,...] to some particular channels with charmonium and light hadrons, as discussed below [1084].

Another configuration proposed for the $Z_\mathrm{c}$ states is a Born–Oppenheimer tetraquark [758]. In such a state a colored $c\bar{c}$ pair is moving in the adiabatic potential created by the light degrees of freedom. This approach aims at providing a general framework for the description of all $XYZ$ states.

To summarize, the properties of the $Z_b(10610)$ and $Z_b(10650)$ states are in good agreement with the assumption that they have molecular structure. The $Z_\mathrm{c}(3885/3900)$ state is a candidate for the $D\bar{D}^*$ molecule, while the absence of the $Z_\mathrm{c}(4020,4025)\rightarrow \pi J/\psi$ signal disfavors the interpretation of $Z_\mathrm{c}(4020)$ or $Z_\mathrm{c}(4025)$ as a $D^*\bar{D}^*$ molecule. The peak positions of the $Z_\mathrm{c}$ signals are about $10\,~\mathrm {MeV}$ above the $D\bar{D}^*$ or $D^*\bar{D}^*$ thresholds. Unless future amplitude analyses find values that are closer to the thresholds, this could be a challenge for the molecular model. Upcoming BES III results on the $Z_\mathrm{c}$ masses, widths, branching fractions and spin-parities from the amplitude analyses, and on the search for other decay channels ($\pi \psi (2S)$ and $\rho \eta _\mathrm{c}$), are crucial for interpreting the $Z_\mathrm{c}$ states.

The $Z_\mathrm{c}$ and $Z_b$ states provide a very rich testing ground for phenomenological models and, given intensive experimental and theoretical efforts, one can expect progress in understanding of the hadronic systems near the open flavor thresholds.

#### Quarkonium and quarkonium-like states above open flavor thresholds 

More than 10 new charmonium and charmonium-like states well above the $D \bar{D}$ threshold have been observed in the last decade by the $B$-factories and other experiments; see Table 12. We discuss first the states that can be assigned to vacant charmonium levels. In 2008 Belle observed the $\chi _{c2}(2P)$ state in $\gamma \gamma$ collisions, later confirmed by BaBar. Almost all of the $\chi _{c2}(2P)$ properties (diphoton width, full width, decay mode) are in nice agreement with the theory expectations, only the mass of the state is ${\sim } 50~\mathrm {MeV}$ below potential model predictions. Another two charmonium candidates (for the third and fourth radial excitations of ${\eta _{c}(1S)}$) are observed by Belle [1085, 1086] in the double charmonium production process $e^{+}e^{-}\rightarrow J/\psi X(3940/4160)$, that decay to $D \bar{D}^{*}$ and $D^{*} \bar{D}^{*}$ channels, respectively. BaBar has not reported any studies of these processes yet. While production processes and decay modes are typical of conventional charmonium, the masses of these states are significantly lower than potential model expectations (e.g., $\eta _\mathrm{c}(4S)$ is expected to be ${\sim } 300~\mathrm {MeV}$ heavier than the observed $X(4160)$). The assignment can be tested by studying the angular distribution of the final state at Belle II.

For the majority of the other new particles, the assignments to the ordinary charmonium states are not well recognized. Contrary to expectations, most of the new states above the open charm threshold, the so-called “$XYZ$ states”, decay into final states containing charmonium, but do not decay into open charm pairs with a detectable rate. This is the main reason why they are discussed as candidates for exotic states. An extended discussion on the different interpretations of these states can be found in [757] and references therein. In the following we discuss recent results on the states above open heavy flavor thresholds.

BaBar confirmed the observation of the process $\gamma \gamma \rightarrow Y(3915)\rightarrow \omega J/\psi$ that was observed by Belle in 2009 [1090]. From angular analyses BaBar determined the $Y(3915)$ spin–parity to be $J^P=0^+$ [1090]. In this analysis it is assumed that in the alternative hypothesis of $J=2$ it is produced in the helicity-2 state, analogous to the production of $\chi _{c2}(1P)$. Given the unknown nature of $Y(3915)$, this assumption could be unjustified. The $J^P=0^+$ state can decay to $D\bar{D}$ in S-wave. Since $Y(3915)$ is $200\,~\mathrm {MeV}$ above the $D\bar{D}$ threshold, its width of $20\,~\mathrm {MeV}$ looks extremely narrow and points to its exotic nature. In addition, the mass difference relative to $\chi _{c2}(2P)$ of $9\,~\mathrm {MeV}$ is too small [1118] to interpret the $Y(3915)$ as $\chi _{c0}(2P)$.

CMS and D0 studied the $B^+\rightarrow K^+\phi J/\psi$ decays [1100, 1101] and confirmed the $Y(4140)$ state near the $\phi J/\psi$ threshold that was observed in 2008 by CDF [1097]. The experiments also see a second structure, the $Y(4274)$, though the mass measurements agree only at about $3\,\sigma$ level. The background under the $Y(4274)$ can be distorted by reflections from the $K^{*+}\rightarrow \phi K^+$ decays, which makes an estimate of the $Y(4274)$ significance difficult [1100]. The $Y(4140)$ and $Y(4274)$ states were not seen in $B$ decays by Belle [1098] and LHCb [1099] and in $\gamma \gamma$ collisions by Belle [1108]. Amplitude analyses with increased statistics at the LHC could settle the controversy.

BaBar updated the $e^+e^{-}\rightarrow \pi ^+\pi ^{-}\psi (2S)$ study using ISR photons and confirmed the $Y(4660)$ that was earlier observed by Belle [1110]. Both BaBar and Belle updated the $e^+e^{-}\rightarrow \pi ^+\pi ^{-} J/\psi$ analyses [1045, 1104]. Belle confirms the $Y(4008)$ using an increased data sample. However, the mass becomes smaller, $M=3891\pm 42\,~\mathrm {MeV}$. BaBar sees events in the same mass region, but they are attributed to a contribution with an exponential shape. BES III data taken in this region will help to clarify the existence of the $Y(4008)$ resonance.

BES III measured the $e^+e^{-}\rightarrow \pi ^+\pi ^{-}h_\mathrm{c}$ cross section at several energies above $4\,~{\mathrm {GeV}}$ [1047]. Unlike the $e^+e^{-}\rightarrow \pi ^+\pi ^{-}h_b$ reaction, the final three-body state is mainly nonresonant. The shape of the cross section looks different from that of the $\pi ^+\pi ^{-}J/\psi$ final state and possibly exhibits structures distinct from known $Y$ states [1119]. Since hybrids contain a $c\bar{c}$ pair in the spin-singlet state, such structures could be candidates for hybrids. A more detailed scan by BES III is underway.

Belle performed the full amplitude analysis of the $B^0\rightarrow K^+\pi ^{-}\psi (2S)$ decays to determine the spin–parity of the $Z(4430)^{\pm }$ [1112], which is the first charged quarkonium-like state observed by Belle in 2007 [1095, 1120]. The $J^P=1^+$ hypothesis is favored over the $0^{-}$, $1^{-}$ and $2^{-}$ and $2^+$ hypotheses at the levels of $3.4\,\sigma$, $3.7\,\sigma$, $4.7\,\sigma$ and $5.1\,\sigma$, respectively. The width of the $Z(4430)^{\pm }$ became broader, $\Gamma =200^{+49}_{-58}\,~\mathrm {MeV}$. This state and two more states, $Z(4050)^{\pm }$ and $Z(4250)^{\pm }$, in the $\pi ^{\pm }\chi _{c1}$ channel are not confirmed by BaBar [1096, 1113]. The long-standing question of the $Z(4430)^\pm$’s existence has finally been settled by the LHCb experiment, which confirmed both the state itself and its spin–parity assignment of $1^+$ [1114]. For the first time, LHCb has demonstrated resonant behavior of the $Z(4430)^\pm$ amplitude. Belle has performed a full amplitude analysis of the $\bar{B}^0\rightarrow K^{-}\pi ^+J/\psi$ decays and observed a new charged charmonium-like state $Z(4200)^+$ and evidence for the $Z(4430)^+$ decay to $\pi ^+J/\psi$ [1102]. This decay is within the reach of LHCb. Further studies of $Z(4050)^{\pm }$ and $Z(4250)^{\pm }$ could be more difficult at LHCb because of soft photons in the final state and might have to wait for Belle II to run.

Given that the signals of $Y(4260)$, $Y(4360)$ and $Y(4660)$ are not seen in the $e^+e^{-}\rightarrow \mathrm {hadrons}$ cross section ($R_\mathrm{c}$ scan), one can set the limit $\Gamma [Y\rightarrow \pi ^+\pi ^{-}\psi ]\gtrsim 1\,~\mathrm {MeV}$ [1121]. This is at least one order of magnitude higher than that of $\psi (2S)$ and $\psi (3770)$. Recently Belle found that $\psi$ states seen as prominent peaks in the $R_\mathrm{c}$ scan, can also have anomalous hadronic transitions to lower charmonia. Belle observed $\psi (4040)$ and $\psi (4160)$ signals in the $e^{+}e^{-}\rightarrow \eta J/\psi$ cross section measured using ISR [1122]. The partial widths are measured to be $\Gamma [\psi (4040,4160)\rightarrow \eta J/\psi ]\sim 1\,~\mathrm {MeV}$. Thus it seems to be a general feature of all charmonium(-like) states above the open charm thresholds to have intense hadronic transitions to lower charmonia. A similar phenomenon is found in the bottomonium sector: In 2008 Belle observed anomalously large rates of the $\Upsilon ({5}{S})\rightarrow {{\pi ^{+}\pi ^{-}}}\Upsilon ({n}{S})$ ($n=1,~2,~3$) transitions with partial widths of $300-400\,~{\mathrm {keV}}$ [1116]. Recently Belle reported preliminary results on the observation of $\Upsilon ({5}{S})\rightarrow \eta \Upsilon (1S,2S)$ and $\Upsilon ({5}{S}) \rightarrow {{\pi ^{+}\pi ^{-}}}\Upsilon ({1}{D})$ with anomalously large rates [985]. It is proposed that these anomalies are due to rescatterings [1123, 1124]. The large branching fraction of the $\Upsilon ({4}{S})\rightarrow \Upsilon ({1}{S})\eta$ decay observed in 2010 by BaBar could have a similar origin [1125].

The mechanism can be considered either as a rescattering of the $D \bar{D}$ or $B\bar{B}$ mesons, or as a contribution of the molecular component to the quarkonium wave function.

The model in which $Y(4260)$ is a $D_1(2420)\bar{D}$ molecule naturally explains the high probability of the intermediate molecular resonance in the $Y(4260)\rightarrow \pi ^+\pi ^{-}J/\psi$ transitions [1126, 1127] and predicts the $Y(4260)\rightarrow \gamma X(3872)$ transitions with high rates [1128]. Such transitions have recently been observed by BES III, with [1107]

4.15 $$\begin{aligned} \frac{\sigma [e^+e^{-}\rightarrow \gamma X(3872)]}{\sigma [e^+e^{-}\rightarrow \pi ^+\pi ^{-}J/\psi ]}\sim 11~\%. \end{aligned}$$

Despite striking similarities between the observations in the charmonium and bottomonium sectors, there are also clear differences. In the charmonium sector, each of the $Y(3915)$, $\psi (4040)$, $\psi (4160)$, $Y(4260)$, $Y(4360)$ and $Y(4660)$ decays to only one particular final state with charmonium [$\omega J/\psi$, $\eta J/\psi$, ${{\pi ^{+}\pi ^{-}}}J/\psi$ or ${{\pi ^{+}\pi ^{-}}}\psi (2S)$]. In the bottomonium sector, there is one state with anomalous properties, the $\Upsilon ({5}{S})$, and it decays to different channels with similar rates [${{\pi ^{+}\pi ^{-}}}\Upsilon ({n}{S})$, ${{\pi ^{+}\pi ^{-}}}h_{b}(mP)$, ${{\pi ^{+}\pi ^{-}}}\Upsilon ({1}{D})$, $\eta \Upsilon ({n}{S})$]. There is no general model describing these peculiarities. To explain the affinity of the charmonium-like states to some particular channels, the notion of “hadrocharmonium” was proposed in [1084]. It is a heavy quarkonium embedded into a cloud of light hadron(s), thus the fall-apart decay is dominant. Hadrocharmonium could also provide an explanation for the charged charmonium-like states $Z(4430)^+$, $Z(4050)^+$ and $Z(4250)^+$.

#### Summary

Quarkonium spectroscopy enjoys an intensive flood of new results. The number of spin-singlet bottomonium states has increased from one to four over the last 2 years, including a more precise measurement of the ${\eta _{b}(1S)}$ mass, $11\,~\mathrm {MeV}$ away from the PDG2012 average. There is evidence for one of the two still missing narrow charmonium states expected in the region between the $D \bar{D}$ and $D \bar{D}^{*}$ thresholds. Observations and detailed studies of the *charged* bottomonium-like states $Z_{b}(10610)$ and $Z_{b}(10650)$ and first results on the charged charmonium-like states $Z_\mathrm{c}$ open a rich phenomenological field to study exotic states near open flavor thresholds. There is also significant progress and a more clear experimental situation for the highly excited heavy quarkonium states above open flavor thresholds. Recent highlights include confirmation of the $Y(4140)$ state by CMS and D0, observation of the decays $\psi (4040,4160)\rightarrow \eta J/\psi$ by Belle, measurement of the energy dependence of the $e^+e^{-}\rightarrow \pi ^+\pi ^{-}h_\mathrm{c}$ cross section by BES III, observation of the $Y(4260)\rightarrow \gamma X(3872)$ by BES III and determination of the $Z(4430)$ spin–parity from full amplitude analysis by Belle. A general feature of highly excited states is their large decay rate to lower quarkonia with the emission of light hadrons. Rescattering is important for understanding their properties, however, there is no general model explaining their decay patterns. The remaining experimental open questions or controversies are within the reach of the LHC or will have to wait for the next generation $B$-factory.

From the theoretical point of view, low quarkonium excitations are in agreement with lattice QCD and effective field theories calculations, which are quite accurate and able to challenge the accuracy of the data. Higher quarkonium excitations show some unexpected properties. Specific effective field theories have been developed for some of these excitations. Lattice studies provide a qualitative guide, but in most cases theoretical expectations still rely on models and a quantitative general theory is still missing.

### Strong coupling $\alpha _\mathrm{s}$

There are several heavy-quark systems that are suitable for a precise determination of ${\alpha _{\mathrm{s}}}$, mainly involving quarkonium, or quarkonium-like, configurations, which are basically governed by the strong interactions. One can typically take advantage of non-relativistic effective theories, high-order perturbative calculations that are available for these systems, and of progress in lattice computations.

Using moments of heavy-quark correlators calculated on the lattice, and the continuum perturbation theory results for them [1129], the HPQCD collaboration has obtained ${\alpha _{\mathrm{s}}}(M_Z)=0.1183\pm 0.0007$ [2]. This result is very close, both in the central value and error, to the one obtained from measuring several quantities related to short-distance Wilson loops by the same collaboration [2]. The energy between two static sources in the fundamental representation, as a function of its separation, is also suitable for a precise ${\alpha _{\mathrm{s}}}$ extraction. The perturbative computation has now reached a three-loop level [1130–1135], and lattice-QCD results with ${N}_\mathrm{f}=2+1$ sea quarks are available [1136]. A comparison of the two gives ${\alpha _{\mathrm{s}}}(M_Z)=0.1156^{+0.0021}_{-0.0022}$ [1137]. New lattice data for the static energy, including points at shorter distances, will be available in the near future, and an update of the result for ${\alpha _{\mathrm{s}}}$ can be expected, in principle with reduced errors.

Quarkonium decays, or more precisely ratios of their widths (used to reduce the sensitivity to long-distance effects), were readily identified as a good place for ${\alpha _{\mathrm{s}}}$ extractions. One complication is the dependence on color-octet configurations. The best ratio for ${\alpha _{\mathrm{s}}}$ extractions, in the sense that the sensitivity to color-octet matrix elements and relativistic effects is most reduced, turns out to be $R_{\gamma }:=\Gamma (\Upsilon \rightarrow \gamma X)/\Gamma (\Upsilon \rightarrow X)$, from which one obtains ${\alpha _{\mathrm{s}}}(M_Z)=0.119^{+0.006}_{-0.005}$ [1138]. The main uncertainty in this result comes from the systematic errors of the experimental measurement of $R_{\gamma }$ [1139]. Belle could be able to produce an improved measurement of $R_{\gamma }$, which may translate into a better ${\alpha _{\mathrm{s}}}$ determination.

Very recently the CMS collaboration has presented a determination of ${\alpha _{\mathrm{s}}}$ from the measurement of the inclusive cross section for $t\bar{t}$ production, by comparing it with the NNLO QCD prediction. The analysis is performed with different NNLO PDF sets, and the result from the NNPDF set is used as the main result. Employing $m_t=173.2\pm 1.4$ GeV, ${\alpha _{\mathrm{s}}}(M_Z)=0.1151^{+0.0033}_{-0.0032}$ is obtained [1140], the first ${\alpha _{\mathrm{s}}}$ determination from top-quark production.

### Heavy quarkonium production

Forty years after the discovery of the $J/\psi$, the mechanism underlying quarkonium production has still not been clarified. Until the mid-90s mostly the traditional color singlet model was used in perturbative cross section calculations. The dramatic failure to describe $J/\psi$ production at the Tevatron led, however, to a search for alternative explanations. The NRQCD factorization conjecture has by now received most acceptance, although not yet being fully established.

#### Summary of recent experimental progress

The past couple of years have seen incredible progress in measurements of quarkonium production observables, which was mainly, but not solely, due to the operation of the different LHC experiments. Here we will give an overview of the most remarkable results of the past years.

The production rates of a heavy quarkonium $H$ are split into direct, prompt, and nonprompt contributions. Direct production refers to the production of $H$ directly at the interaction point of the initial particles, while prompt production also includes production via radiative decays of higher quarkonium states, called *feed-down* contributions. Nonprompt production refers to all other production mechanisms, mainly the production of charmonia from decaying $B$ mesons, which can be identified by a second decay vertex displaced from the interaction point.

*a.* $J/\psi$ *production in* $pp$ *collisions* The 2004 CDF transverse momentum $p_\mathrm{T}$ distribution measurement of the $J/\psi$ production cross section [1141] is still among the most precise heavy quarkonium production measurements. But since theory errors in all models for heavy quarkonium production are still much larger than today’s experimental errors, it is in general not higher precision which is needed from the theory side, but rather new and more diverse production observables. And this is where the LHC experiments have provided very important input. As for the $J/\psi$ hadroproduction cross section, they have extended the CDF measurement [1141] into new kinematic regions: Obviously, the measurements have been performed at much higher center-of-mass energies than before, namely at $\sqrt{s}=2.76$, 7, and 8 TeV. But more important for testing quarkonium production models is the fact that there are measurements which exceed the previously measured $p_\mathrm{T}$ range both at high $p_\mathrm{T}$, as by ATLAS [1142] and CMS [1143], and at low $p_\mathrm{T}$, as in the earlier CMS measurement [1144], but also in the recent measurement by the PHENIX collaboration at RHIC [1145]. We note that this list is not complete, and that there have been many more $J/\psi$ hadroproduction measurements recently than those cited here.

*b.* $\psi (2S)$ *and* $\chi _\mathrm{c}$ *production in* $pp$ *collisions* $J/\psi$ is the quarkonium which is easiest to be measured due to the large branching ratio of its leptonic decay modes, but in recent years, high precision measurements have been also performed for the $\psi (2S)$, namely by the CDF [1146], the CMS [1143], and the LHCb [1147] collaborations. Also the $\chi _\mathrm{c}$ production cross sections were measured via their decays into $J/\psi$ by LHCb [1148], the first time since the CDF measurement [1149] in 2001. The $\chi _{c2}$ to $\chi _{c1}$ production ratio was measured at LHCb [1150], CMS [1151] and previously by CDF [1152]. These measurements are of great importance for the theory side since they allow fits of NRQCD LDMEs for these charmonia and determine direct $J/\psi$ production data, which can in turn be compared to direct production theory predictions.

*c.* $\Upsilon$ *production in* $pp$ *collisions* $\Upsilon (1S)$, $\Upsilon (2S)$, and $\Upsilon (3S)$ production cross sections were measured at the LHC by ATLAS [1153] and LHCb [1154, 1155]. Previously, $\Upsilon$ was produced only at the Tevatron [1156, 1157].

**Fig. 30:**
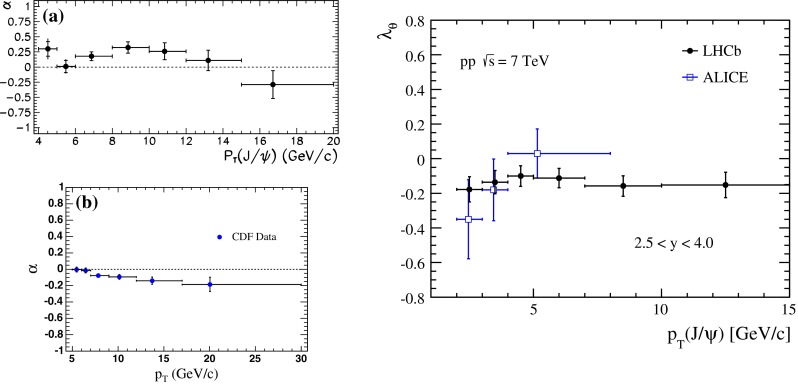
The $J/\psi$ polarization parameter $\alpha \equiv \lambda _\theta$ in the helicity frame as measured by CDF in Tevatron run I [1158] (**a**), run II [1159] (**b**), and by ALICE [1160] and LHCb [1161] at the LHC (*right*). Adapted from [1158, 1159, 1161], respectively

**Fig. 31:**
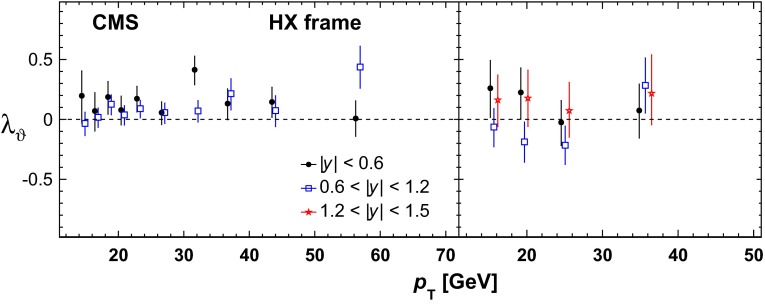
The polarization parameter $\lambda _\theta$ in the helicity frame for $J/\psi$ (*left*) and $\psi (2S)$ (*right*) production as measured by CMS [1162]. Adapted from [1162]

**Fig. 32:**
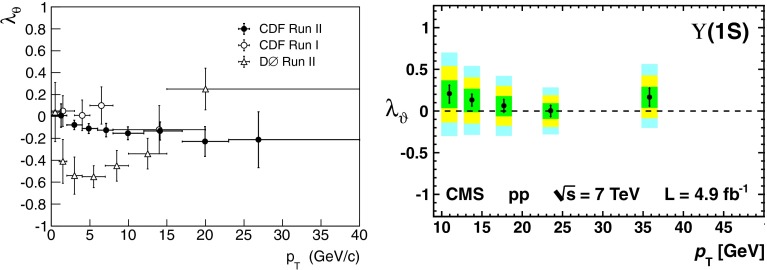
The $\Upsilon (1S)$ polarization parameter $\lambda _\theta$ in the helicity frame as measured by CDF [1156, 1163], D0 [1164] and CMS [1165]. Adapted from [1163, 1165], respectively

*d.* *Polarization measurements in* $pp$ *collisions* The measurements of the angular distributions of the quarkonium decay leptons are among the most challenging experimental tasks in quarkonium physics, because much more statistics and a much better understanding of the detector acceptances than in cross section measurements is needed. These angular distributions $W(\theta ,\phi )$ are directly described by the polarization parameters $\lambda _\theta$, $\lambda _\phi$, and $\lambda _{\theta \phi }$ via

4.16 $$\begin{aligned} W(\theta ,\phi )&\propto 1+\lambda _\theta \cos ^2\theta +\lambda _\phi \sin ^2\theta \cos (2\phi ) \nonumber \\&{}+\lambda _{\theta \phi }\sin (2\theta )\cos \phi , \end{aligned}$$

where $\theta$ and $\phi$ are, respectively, the polar and azimuthal angles of the positively charged decay lepton in the quarkonium rest frame. These polarization measurements pose highly nontrivial tests for quarkonium production models, and have therefore probably been the most anticipated LHC results on quarkonium. Previous Tevatron measurements tended to give ambiguous results: The CDF measurements of $J/\psi$ polarization in Tevatron run I [1158] and II [1159] have been in partial disagreement; see Fig. 30, similar to the $\Upsilon (1S)$ polarization measured by D0 [1164] and by CDF in Tevatron run I [1156] and II [1163]; see Fig. 32. At RHIC, $J/\psi$ polarization has been measured by PHENIX [1166] and STAR [1167]. At the LHC, $J/\psi$ polarization has so far been measured by ALICE [1160], LHCb [1161], and CMS [1162]; see Figs. 30 and 31. Furthermore, CMS has measured the $\psi (2S)$ [1162] and $\Upsilon$ [1165] polarization; see Figs. 31 and 32. None of the CDF Tevatron run II and the LHC measurements have found a strong and significant transverse or longitudinal polarization for any quarkonium. CDF at Tevatron run II and LHCb do, however, seem to prefer slight longitudinal polarizations in their helicity frame quarkonium polarization measurements, whereas in the CMS measurements there seems to be a tendency for slight transverse polarizations in the helicity frame, see Figs. 30, 31, and 32.

*e. Recent* $ep$ *collision results* For testing theory predictions, in particular the universality of NRQCD long distance matrix elements, we need to consider experimental data from a variety of different production mechanisms. Very important charmonium production data have thereby in the past come from inelastic photoproduction at the $ep$ collider HERA, which came in distributions in the transverse charmonium momentum $p_\mathrm{T}$, the photon-proton invariant mass $W$ and the inelasticity variable $z$. The latest update on inclusive $J/\psi$ production cross sections was in 2012 by the ZEUS collaboration [1168]. This publication also presented values for $\sigma (\psi (2S))/\sigma (J/\psi )$ with error bars reduced by about two thirds relative to the previous ZEUS measurement [1169] at HERA 1. The $J/\psi$ polarization measurements by the ZEUS [1170] and H1 [1171] collaborations were, however, still associated with such large errors that no unambiguous picture of the $J/\psi$ polarization in photoproduction emerged. Furthermore, no $\Upsilon$ photoproduction could be observed at HERA. Therefore, from the theory side, a new $ep$ collider at much higher energies and luminosities than HERA, like possibly an LHeC, would be highly desired. On the other hand, there is still no NLO calculation for $J/\psi$ production in deep inelastic scattering available, as, for example, measured most recently by H1 [1171].

*f. Further production observables* The LHCb experiment with its especially rich quarkonium program has also measured completely new observables which still need to be exploited fully in theory tests: For the first time in $pp$ collisions the double $J/\psi$ production cross section was measured [1172], as well as the production of $J/\psi$ in association with charmed mesons [1173]. Like double charmonium production, $J/\psi +c\overline{c}$ was previously only measured at the $B$ factories, latest in the Belle analysis [1174], which was crucial for testing $J/\psi$ production mechanisms in $e^+e^{-}$ production. $J/\psi$ production in association with $W$ bosons has for the first time been measured by the ATLAS collaboration [1175]. Exclusive charmonium hadroproduction has been observed recently by CDF [1176] and LHCb [1177, 1178]. Exclusive production had previously been a domain of $ep$ experiments; see [1179] for a recent update by the H1 collaboration. Another observable for which theory predictions exist is the $J/\psi$ production rate in $\gamma \gamma$ scattering. This observable has previously been measured at LEP by DELPHI [1180] with very large uncertainties and could possibly be remeasured at an ILC.

#### NLO tests of NRQCD LDME universality

**Table 13:** Overview of different NLO fits of the CO LDMEs. Analysis [770] is a global fit to inclusive $J/\psi$ yield data from 10 different $pp$, $\gamma p$, $ee$, and $\gamma \gamma$ experiments. In [1181], fits to $pp$ yields from CDF [1141, 1146] and LHCb [1147, 1148, 1182] were made. In [1183], three values for their combined fit to CDF $J/\psi$ yield and polarization [1158, 1159] data are given: A default set, and two alternative sets. Analysis [1184] is a fit to the $\chi _{c2}/\chi _{c1}$ production ratio measured by CDF [1152]. The analyses [770] and [1183] refer only to direct $J/\psi$ production, and in the analyses [1181] and [1183] $p_T<7$ GeV data was not considered. The color singlet LDMEs for the ${^3}S_1^{[1]}$ and ${^3}P_0^{[1]}$ states were not fitted. The values of the LDMEs given in the second through sixth column (referring to [770, 1181], and [1183]) were used for the plots of Fig. 33

	Butenschoen, Kniehl [770]:	Gong, Wan, Wang, Zhang [1181]:	Chao, Ma, Shao, Wang, Zhang [1183]:			Ma, Wang, Chao [1184]:
			(default set)	(set 2)	(set 3)	
$\langle \mathcal{O}^{J/\psi }({^3}S_1^{[1]}) \rangle /\text{ GeV }^3$	$1.32$	$1.16$	$1.16$	$1.16$	$1.16$	
$\langle \mathcal{O}^{J/\psi }({^1}S_0^{[8]}) \rangle /\text{ GeV }^3$	$0.0497\pm 0.0044$	$0.097\pm 0.009$	$0.089\pm 0.0098$	$0$	$0.11$	
$\langle \mathcal{O}^{J/\psi }({^3}S_1^{[8]}) \rangle /\text{ GeV }^3$	$0.0022\pm 0.0006$	$-0.0046\pm 0.0013$	$0.0030\pm 0.012$	$0.014$	$0$	
$\langle \mathcal{O}^{J/\psi }({^3}P_0^{[8]}) \rangle /\text{ GeV }^5$	$-0.0161\pm 0.0020$	$-0.0214\pm 0.0056$	$0.0126\pm 0.0047$	$0.054$	$0$	
$\langle \mathcal{O}^{\psi (2S)}({^3}S_1^{[1]}) \rangle /\text{ GeV }^3$		$0.758$				
$\langle \mathcal{O}^{\psi (2S)}({^1}S_0^{[8]}) \rangle /\text{ GeV }^3$		$-0.0001\pm 0.0087$				
$\langle \mathcal{O}^{\psi (2S)}({^3}S_1^{[8]}) \rangle /\text{ GeV }^3$		$0.0034\pm 0.0012$				
$\langle \mathcal{O}^{\psi (2S)}({^3}P_0^{[8]}) \rangle /\text{ GeV }^5$		$0.0095\pm 0.0054$				
$\langle \mathcal{O}^{\chi _0}({^3}P_0^{[1]}) \rangle /\text{ GeV }^5$		$0.107$				$0.107$
$\langle \mathcal{O}^{\chi _0}({^3}S_1^{[8]}) \rangle /\text{ GeV }^3$		$0.0022\pm 0.0005$				$0.0021\pm 0.0005$

**Fig. 33:**
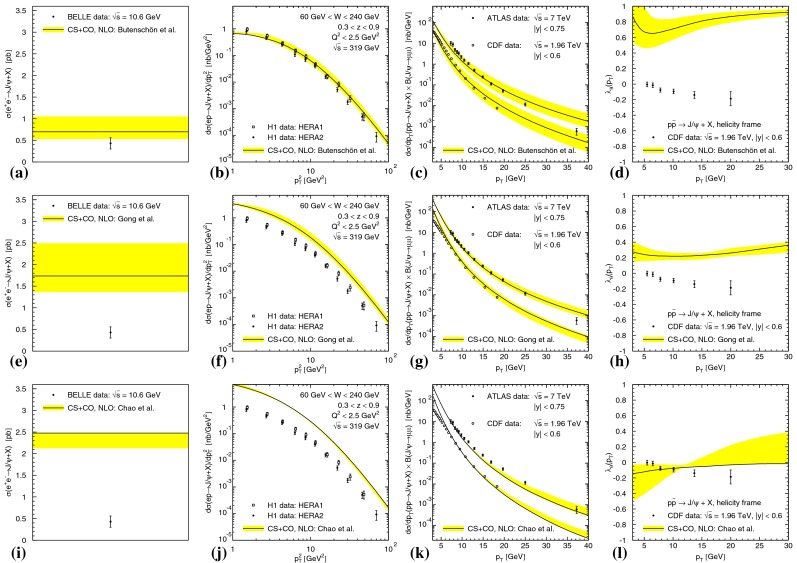
The predictions of the $J/\psi$ total $e^+e^{-}$ cross section measured by Belle [1174], the transverse momentum distributions in photoproduction measured by H1 at HERA [1171, 1185], and in hadroproduction measured by CDF [1141] and ATLAS [1142], and the polarization parameter $\lambda _\theta$ measured by CDF in Tevatron run II [1159]. The predictions are plotted using the values of the CO LDMEs given in [770], [1181] and [1183] and listed in Table 13. The *error bars* of graphs **a**–**g** refer to scale variations, of graph **d** also fit errors, errors of graph **h** according to [1181]. As for graphs **i**–**l**, the central lines are evaluated with the default set, and the *error bars* evaluated with the alternative sets of the CO LDMEs used in [1183] and listed in Table 13. From [1186]

The phenomenological relevance of the NRQCD factorization conjecture is closely tied to the question of whether or not the LDMEs can be shown to be universal. In this section recent works will be reviewed which aim at examining this universality at Next-to-Leading Order (NLO) in $\alpha _\mathrm{s}$. In the case of $\chi _{cJ}$, these tests include just the leading order of the NRQCD $v$ expansion, formed by the $n={^3}P_J^{[1]}$ and $n={^3S}_1^{[8]}$ states. In the case of ${^3}S_1$ quarkonia, these tests include the terms up to relative order $O(v^4)$ in the $v$ expansion, namely the $n={^3}S_1^{[1]}$ color singlet state, as well as the $n={^1}S_0^{[8]}$, ${^3}S_1^{[8]}$, and ${^3}P_J^{[8]}$ Color Octet (CO) states; see Table 5. The relativistic corrections involving the $\langle \mathcal{P}^{H}({^3}S_1^{[1]}) \rangle$ and $\langle \mathcal{Q}^{H}({^3}S_1^{[1]}) \rangle$ LDMEs are, however, not part of these analyses, although they are of order $O(v^2)$ and $O(v^4)$ in the $v$ expansion. There are two reasons for that: First, the corresponding NLO calculations are far beyond the reach of current techniques, and secondly, they are expected to give significant contributions to hadroproduction only at $p_\mathrm{T}\ll m_\mathrm{c}$ and for photoproduction only at $z\approx 1$. This behavior is inferred from the behavior at LO in $\alpha _\mathrm{s}$ [1187, 1188] and can be understood by noting that new topologies of Feynman diagrams open up when doing the transition from the ${^3}S_1^{[1]}$ state to the CO states, but not when calculating relativistic corrections: For example, at leading order in $\alpha _\mathrm{s}$ the slope of the transverse momentum distribution in hadroproduction is $\mathrm{d}\sigma /dp_\mathrm{T} \approx p_\mathrm{T}^{-8}$ for the ${^3}S_1^{[1]}$ state, compared to $\mathrm{d}\sigma /dp_\mathrm{T} \approx p_\mathrm{T}^{-6}$ for the ${^1}S_0^{[8]}$ and ${^3}P_J^{[8]}$ states and $\mathrm{d}\sigma /dp_\mathrm{T} \approx p_\mathrm{T}^{-4}$ for the ${^3}S_1^{[8]}$ state.

The $O(\alpha _\mathrm{s})$ corrections to the necessary unpolarized short-distance cross sections of the $n={^1}S_0^{[8]}$, ${^3}S_1^{[1/8]}$, and ${^3}P_J^{[1/8]}$ intermediate states have been calculated for most of the phenomenologically relevant inclusive quarkonium production processes: For two-photon scattering [770, 1189], $e^+e^{-}$ scattering [1190], photoproduction [770, 1191] and hadroproduction [1184, 1192–1195]. The polarized cross sections have been calculated for photoproduction [1196] and hadroproduction [1181, 1183, 1197, 1198].

In [770], a global fit of the $J/\psi$ CO LDMEs to 26 sets of inclusive $J/\psi$ production yield data from 10 different $pp$, $\gamma$p, $\gamma \gamma$, and $e^+e^{-}$ experiments was done; see the second column of Table 13 for the fit results. This fit describes all data, except perhaps the two-photon scattering at LEP [1180], reasonably well. This fit is overconstrained, and practically independent of possible low-$p_\mathrm{T}$ cuts (unless such high $p_\mathrm{T}$ cuts are chosen that all data except hadroproduction drop out of the fit [1199]). Furthermore, the resulting LDMEs are in accordance with the velocity scaling rules predicted by NRQCD; see Table 5. Thus the fit is in itself already a nontrivial test of the NRQCD factorization conjecture, especially since the high-$z$ photoproduction region can now also be well described, which had been plagued by divergent behavior in the earlier Born analyses [1200, 1201]. However, in [1197] it was shown that these CO LDME values lead to predictions of a strong transverse $J/\psi$ polarization in the hadroproduction helicity frame, which is in contrast to the precise CDF Tevatron run II measurement [1159]; see Fig. 33d. On the other hand, in [1183] it was shown that both the measured $J/\psi$ hadroproduction cross sections and the CDF run II polarization measurement [1159] can, even at the highest measured $p_\mathrm{T}$ values, be well described when choosing one of the three CO LDME sets listed in columns four through six of Table 13. These LDMEs, however, result in predictions for $e^+e^{-}$ annihilation and photoproduction which are factors four to six above the data; see Fig. 13e–f. Third, the calculation [1181] is the first NLO polarization analysis to include feed-down contributions. To this end, the CO LDMEs of $J/\psi$, $\psi (2S)$ and $\chi _{cJ}$ were fitted to CDF [1141, 1146] and LHCb [1147, 1148, 1182] unpolarized production data with $p_\mathrm{T}>7$ GeV; see column three of Table 13. These fit results were then used for the predictions of Fig. 33e–h, taking the $\psi (2S)$ and $\chi _{cJ}$ feed-down contributions consistently into account. A similar analysis has recently also been performed for $\Upsilon (1S,2S,3S)$ production [1198].

The shape of high-$p_\mathrm{T}$ $J/\psi$ hadroproduction yield can be nicely described by the ${^1}S_0^{[8]}$ component alone, which automatically yields unpolarized hadroproduction. Since at $p_\mathrm{T}>10$ GeV this is already all data available, there is no tension between NRQCD predictions and current data if the validity of the NRQCD factorization conjecture is restricted to high enough $p_\mathrm{T}$ values and the ${^3}S_1^{[1/8]}$ and ${^3}P_J^{[1/8]}$ LDMEs are very small or even put to zero, as for example in the sixth column of Fig. 33 (set 3). This is also the spirit of [1202], and of the analysis [1203], in which the NLO short distance cross sections used in [1183] are combined with $c\overline{c}$ production via single parton fragmentation using fragmentation functions at order $\alpha _\mathrm{s}^2$ including a leading log resummation.

To summarize, none of the proposed CO LDME sets is able to describe all of the studied $J/\psi$ production data sets, which poses a challenge to the LDME universality. Possible resolutions include the following:

1. The perturbative $v$ expansion might converge too slowly.
2. NRQCD factorization might hold for exclusive, but not inclusive, production.
3. NRQCD factorization might hold only in the region $p_\mathrm{T}\gg M_\mathrm{onium }$. Currently, photoproduction cross sections are measured only up to $p_\mathrm{T}=10$ GeV.
4. NRQCD factorization might not hold for polarized production.

#### Recent calculations of relativistic corrections

**Table 14:** Color singlet model predictions for $\sigma (e^{+}e^{-}\rightarrow J/\psi +\eta _{c})$ compared to $B$-factory data [1204–1206]. As for the theoretical predictions for the leading-order cross section as well as the corrections of order $O(\alpha _{s})$, $O(v^{2})$, and $O(\alpha _{s} v^{2})$, we compare the results obtained in [1207–1209]. These calculations mainly differ by different methods of color singlet LDME determinations. As for the values of [1208], the leading-order results include pure QED contributions, the $O(\alpha _{s})$ results include interference terms with the QED contributions, and the $O(v^{2})$ results include in part a resummation of relativistic corrections, the $O(\alpha _{s} v^{2})$ results do, however, include the interference terms of the $O(\alpha _{s})$ and $O(v^{2})$ amplitudes only. The short-distance coefficients of the $O(\alpha _{s})$ contribution used in [1207] and [1208] were taken over from [1210]. The experimental cross sections refer to data samples in which at least 2, respectively 4, charged tracks were identified

	He, Fan,	Bodwin,	Li, Wang [1209]	
	Chao [1207]	Lee,Yu [1208]		
	$\alpha _{s}(2m_{c})$	$\alpha _{s}(\sqrt{s}/2)$	$\alpha _{s}(\sqrt{s}/2)$	$\alpha _{s}(2m_{c})$
$\sigma _{LO}$	9.0 fb	6.4 fb	4.381 fb	7.0145 fb
$\sigma (\alpha _{s})$	8.8 fb	6.9 fb	5.196 fb	7.367 fb
$\sigma (v^{2})$	2.2 fb	2.9 fb	1.714 fb	2.745 fb
$\sigma (\alpha _{s} v^{2})$		1.4 fb	0.731 fb	0.245 fb
sum	20.0 fb	$17.6^{+8.1}_{-6.7}$ fb	12.022 fb	17.372 fb
Belle [1204]		$33^{+7}_{-6}\pm 9$ fb ($\ge$4 charged tracks)		
Belle [1205]		$25.6\pm 2.8 \pm 3.4$ fb ($\ge$2 charged tracks)		
BaBar [1206]		$17.6\pm 2.8^{+1.5}_{-2.1}$ fb ($\ge$2 charged tracks)		

As explained in the last section, the relativistic corrections of order $O(v^2)$ in the NRQCD $v$ expansion have at leading order in $\alpha _\mathrm{s}$ in inclusive hadro- [1188] and photoproduction [1187] been shown to be less significant than the CO contributions of order $O(v^4)$ in the NRQCD $v$ expansion. Similarly, the $O(v^2)$ [1211] and the technically challenging $O(v^4)$ [769] relativistic corrections to gluon fragmentation into ${^3}S_1$ quarkonia have turned out to be small. The relativistic $O(v^2)$ corrections to the process $e^+e^{-}\rightarrow J/\psi +gg$ have, however, turned out to be between 20 % and 30 % [1212, 1213] relative to the leading order CS cross section, an enhancement comparable in size to the $O(\alpha _\mathrm{s})$ CS correction [1214, 1215]. These corrections helped bring the color singlet model prediction for inclusive $J/\psi$ production in $e^+e^{-}$ collisions in rough agreement with experimental data [1174].

Similarly, in the exclusive process $e^+e^{-}\rightarrow J/\psi +\eta _\mathrm{c}$, $O(\alpha _\mathrm{s})$ corrections as well as relativistic corrections of $O(v^2)$ were necessary to bring the color singlet model prediction in agreement with data; see Table 14. Recently, even $O(\alpha _\mathrm{s} v^2)$ corrections to this process have been calculated [1209, 1216]. For a review of the history of the measurements and calculations of this process, as well as for a description of different methods to determine the LDMEs of relative order $O(v^2)$, we refer to section 4.5.1 of [757].

As a final point of this section, we mention the interesting work [1217] in which relativistic corrections to the process $gg\rightarrow J/\psi +g$ via color octet states formally of order $O(v^6)$ were estimated. According to this analysis, at leading order in $\alpha _\mathrm{s}$, they might reduce the $O(v^4)$ CO contributions by up to 20–40 % in size.

#### Calculations using $k_\mathrm{T}$ factorization

Color singlet model predictions for $J/\psi$ production face many phenomenological problems: Except for $e^+e^{-}$ annihilation, NLO color singlet model predictions are shown to lie significantly below inclusive $J/\psi$ production data, 1–2 orders of magnitude for hadroproduction and $\gamma \gamma$ scattering, and a factor 3–5 for photoproduction at HERA; see, for example, [770]. As in photoproduction [1218, 1219], $J/\psi$ polarization in hadroproduction [1220] is at NLO predicted to be highly longitudinal in the helicity frame, in contrast to the CDF measurement at Tevatron run II [1159].

According to [1221, 1222], these shortcomings can be overcome when the transverse momenta $k_\mathrm{T}$ of the initial gluons are retained. The off-shell matrix elements are then folded with unintegrated, $k_\mathrm{T}$ dependent, Parton Distribution Functions (uPDFs). The weakest point of this approach is certainly the derivation of the uPDFs from the usual gluon PDFs using varying prescriptions. The latest analyses [1221, 1222] show very good agreement with $J/\psi$ photoproduction data at HERA [1169–1171, 1185] and hadroproduction at the LHC [1142, 1144, 1182]. On top of that, the $J/\psi$ is predicted to be largely unpolarized, in line with all recent polarization measurements; see paragraph $d$ in Sect. 4.5.1. As for hadroproduction, the conclusions are however contrary to the author’s earlier findings [1223], which show longitudinal $J/\psi$ polarization and cross sections an order of magnitude below the CDF production data. They also disagree with the recent work [1224], where $J/\psi$ hadroproduction at the LHC was studied in the same way, comparing to the same data [1142, 1144, 1182], even when the same uPDFs [1225, 1226] were used. Here, the color singlet predictions lie again clearly below the data, and the difference was even used to fit the CO LDMEs of NRQCD in a $k_\mathrm{T}$ factorization approach.

We note that calculations in the $k_\mathrm{T}$ factorization scheme can be performed for any intermediate Fock state of the NRQCD $v$ expansion. On the other hand, even a fully worked out framework of $k_\mathrm{T}$ factorization at NLO in $\alpha _\mathrm{s}$ could not cure the problem of uncanceled infrared singularities in color singlet model calculations for $P$ wave quarkonia.

#### Current trends in theory

The most prominent candidate theory for heavy quarkonium production is NRQCD, and lots of effort is going on to prove its factorization theorem on the one hand, and to show the universality of the LDMEs by comparison to data on the other. Since at the moment there are hints that at least to the orders currently considered in perturbation theory, not all data might be simultaneously described by single LDME sets, more effort will be going on to refine NRQCD calculations for specific observables or specific kinematic regimes, such as the low and high $p_\mathrm{T}$ limits of the hadroproduction cross sections. For low $p_\mathrm{T}$ resummation of large logarithms, the recent work [1227] followed the idea of [1228] to apply the Collins–Soper–Sterman impact parameter resummation formalism [90]. For high $p_\mathrm{T}$ resummation, the factorization theorem of [781, 783] in terms of single and double parton fragmentation functions, and the soft-collinear effective theory approach [785, 786] can be applied. Other paths may be to apply transverse momentum-dependent PDFs in quarkonium production calculations, but the uncertainties inherent to these calculations will still need to be thoroughly investigated, as can be seen from contradicting $k_\mathrm{T}$ factorization results. But also in more phenomenologically based models, like the color evaporation model, new predictions are still calculated [1229].

### Future directions

Our understanding of heavy quark hadronic systems improves with the progress made on experimental measurements of masses, production and decay rates, the development of suitable effective field theories, perturbative calculations within these frameworks, and the progress on lattice gauge theory calculations.

Lattice simulations are obtaining a more and more prominent role in heavy quark physics. They may compute low-energy matrix elements, factorized by effective field theories, appearing in the study of quarkonia below threshold, improving our understanding of the dynamics of these systems and providing, among others, precision determinations of the strong coupling constant at low energies and the heavy quark masses. For states at and above threshold, they may eventually be able to determine the nature of the $XYZ$ exotic states, including in particular the role that mixing between tetraquark and multihadron states plays. A possible way to address these problems that relies on lattice simulations has been very recently proposed in [1230, 1231]. Lattice simulations are also required for determining non-perturbative form factors needed in extracting the CKM matrix elements $|V_{cb}|$, $|V_{ub}|$, $|V_{cs}|$ and $|V_{cd}|$ from $B \rightarrow D^\star /\pi l \nu$ and $D \rightarrow K/\pi l \nu$ decays, respectively. Current gaps between lattice determinations and experimental fits of these form factors are expected to be removed by further progress in lattice simulations. The emergence of ensembles incorporating the effects of dynamical charm quarks in lattice calculations will help to establish whether charm sea contributions to charmonium spectra and to flavor observables are relevant. At the same time, the trend to finer lattice spacings (even if currently somewhat displaced by a trend to perform simulations at the physical pion mass) is likely to continue in the long run and will eventually enable the use of fully relativistic b-quarks, which will provide an important cross-check on effective field theories, and eventually for some observables replace them.

Rapid progress on the side of effective field theories is currently happening for any system involving heavy quarks. Many quantities, like spectra, decays, transitions and production cross sections, are computed in this framework with unprecedented precision in the velocity and $\alpha _\mathrm{s}$ expansions. Noteworthy progress is happening, in particular, in the field of quarkonium production. Here, the recent Snowmass White Paper on “Quarkonium at the Frontiers of High Energy Physics” [1013] provides an excellent summary. Future work will be likely centered around the effort to search for a rigorous theoretical framework (factorization with a rigorous proof) for inclusive as well as exclusive production of quarkonia at various momentum scales. While a proof of NRQCD factorization is still lacking, performing global analyses of all available data in terms of the NRQCD factorization formalism is equally important, so that the universality of the NRQCD LDMEs can be systematically tested, which is a necessary condition for the factorization conjecture. To better test the conjecture, a resummation of various large logarithms in perturbative calculations in different production environments are critically needed.

The currently running experiments, in particular BESIII and the LHC experiments, will at this stage primarily help refine previous measurements. The LHC will in particular continue to provide measurements on heavy quarkonium production rates at unprecedented values of transverse momentum, provide better measurements on quarkonium polarization, but might also provide more diverse observables, such as associated production of a heavy quarkonium with gauge bosons, jets or other particles. The LHC will also continue to contribute to the studies of $XYZ$ states, and determine the $XYZ$ quantum numbers from amplitude analyses. Studies of $Z_\mathrm{c}$ states at BESIII will continue and provide precise measurements of spin-parities and resonance parameters from multiple decay channels and amplitude analyses.

In the farther future, however, Belle II is expected to produce more and better data that will be particularly useful to reduce the uncertainties on the CKM matrix elements $|V_{cb}|$ and $|V_{ub}|$. Data from a larger phase space can provide more precise information to solve the long-standing discrepancy between the inclusive and exclusive measurements of $|V_{ub}|$. Having about 100 fb$^{-1}$ integrated luminosity from the first Belle II run at the $\Upsilon (6S)$ resonance or at a nearby energy will be very exciting for bottomonium studies. $\Upsilon (6S)$ deserves further studies, in particular, to clarify if $Z_b$ states are also produced in its decays, to search for $\Upsilon (6S) \rightarrow h_b \pi ^+\pi ^{-}$ transitions, and to measure the $e^+e^{-} \rightarrow h_b \pi ^+\pi ^{-}$ cross section as a function of energy, which should provide important information that is needed to answer whether $\Upsilon (6S)$ is more similar to $\Upsilon (5S)$ or to $Y(4260)$ in its properties. With a possible upgrade of the injection system to increase its energy from current $11.2$ GeV, Belle II could access also more molecular states close to $B^{(*)} \overline{B}^{(*)}$, predicted from heavy quark spin symmetry. Belle II and the LHC upgrade, as well as future higher energy/luminosity $ep$ (electron-ion) and $e^+e^{-}$ (Higgs factory) colliders, will provide precision measurements of heavy quarkonium production with more diverse observables in various environments, and might thereby challenge our understanding of how heavy quarkonia emerge from high-energy collisions.

## Searching for new physics with precision measurements and computations

### Introduction

9The scope of the current chapter extends beyond that of QCD. Therefore, we begin with a brief overview of the standard model (SM) in order to provide a context for the new physics searches we describe throughout.

The current SM of particle physics is a renormalizable quantum field theory based on an exact SU(3)$_{c}\times$SU(2)$_{L} \times$U(1) gauge symmetry. As a result of these features and its specific particle content, it contains additional, accidental global symmetries, of which the combination B–L is anomaly free. It also preserves the discrete spacetime symmetry CPT, but C and P and T are not separately guaranteed—and indeed P and C are violated by its explicit construction. It describes all the observed interactions of known matter, save for those involving gravity, with a minimum of 25 parameters. These parameters can be taken as the six quark masses, the six lepton masses, the four parameters each (three mixing angles and a CP-violating phase) in the CKM and Pontecorvo–Maki–Nakagawa–Sakata (PMNS) matrices which describe the mixing of quarks and neutrinos,^10^ respectively, under the weak interactions, and the five parameters describing the gauge and Higgs sectors. The SM encodes CP violation in the quark sector not only through a phase in the CKM matrix but also through a “would-be” parameter $\bar{\theta }$, which the nonobservation of a permanent electric dipole moment of the neutron [1232] limits to $\bar{\theta }< 10^{-10}$ if no other sources of CP violation operate.

The SM, successful though it is, is incomplete in that it leaves many questions unanswered. Setting aside the question of gravity, which is excluded from the onset, the SM cannot explain, e.g., why the $W$ and $Z$ bosons have the masses that they do, the observed pattern of masses and mixings of the fermions, nor why there are three generations. It cannot explain why $\bar{\theta }$ is so small, nor why the baryon asymmetry of the Universe has its observed value. It does not address the nature or even the existence of dark matter and dark energy. It has long been thought that the answers to some of these questions could be linked and, moreover, would find their resolution in new physics at the weak scale. The LHC is engaged in just such a search for those distinct and new phenomena that cannot be described within the SM framework. In Sect. 5.2 we review current collider efforts and how QCD studies advance and support them.

Direct searches for new particles and interactions at colliders certainly involve precision measurements and computations, but discoveries of new physics can also be made at low energies through such efforts. There are two paths: one can discover new physics through (i) the observed failure of the symmetries of the SM, or (ii) the failure of a precision computation to confront a precision measurement. Examples from the first path include searches for permanent electric dipole moments (EDMs) and for charged-lepton flavor violation, at current levels of sensitivity, as well as searches for neutrinoless double beta decay and $n$–$\bar{n}$ oscillations. Prominent examples from the second path are the determination of the lepton anomalous magnetic moments, the $g-2$ of the muon and of the electron. Taken more broadly, the second path is also realized by overconstraining the SM parameters with multiple experiments and trying to find an inconsistency among them. Updated elsewhere in this review are determinations of the weak mixing angle $\theta _W$ (Sect. 3.5) and the strong coupling constant $\alpha _\mathrm{s}$ (Sect. 3.5.3), which are under intense scrutiny by the QCD community. We refer to Sect. 3.5 for a discussion of the muon $g-2$. In this chapter we review such results from quark flavor physics.

QCD plays various roles in these efforts. In the first case, the discovery of whether a SM symmetry is actually broken is essentially an experimental question, though QCD effects play a key role not only in assessing the relative sensitivity of different experiments but also in the interpretation of an experimental result in terms of the parameters of an underlying new physics model. In the second case, the importance of QCD and confinement physics is clear. QCD effects are naturally dominant in all experiments searching for new physics that involve hadrons. We emphasize that experiments in the lepton sector are not immune to such issues, because hadronic effects are simply suppressed by power(s) of the fine-structure constant $\alpha$—they enter virtually through loop corrections. Their ultimate importance is predicated by the precision required to discover new physics in a particular process. Generally, for fixed experimental precision, a lack of commensurate control over QCD corrections, be it in experiments at high-energy colliders or at low energies, can jeopardize our search for physics beyond the SM.

In this document, we consider the broad ramifications of the physics of confinement, with a particular focus on our ability to assess its impact in the context of QCD. This interest drives the selection of the topics which follow. We begin with a brief overview of the role of QCD in collider physics. This part particularly concerns factorization theorems and resummation, which is illustrated with a few select examples. Our discussion, however, is not comprehensive, so that we do not review here the recent and impressive progress on next-to-leading-order (NLO) predictions for multi-parton production processes; see Ref. [1233] for a recent example, or the associated development of on-shell methods, which are reviewed in [1234, 1235]. We refer to Sect. 9.2 of this document for a terse summary of these developments. Next, we move to the primary focus of the chapter, which is the role of QCD in the search for new physics in low-energy processes. There is a large array of possible observables to consider; we refer the reader to a brief, recent overview [1236], as well as to a dedicated suite of reviews [1237–1244]. In this chapter we describe the theoretical framework in which such experiments can be analyzed before delving more deeply into examples which illustrate the themes we have described. We consider, particularly, searches for permanent electric dipole moments of the neutron and proton and precision determinations of $\beta$-decay correlation coefficients. We refer the reader to Sect. 3.5 for a detailed discussion of the magnetic moment of the muon. We proceed to consider the need for and the computation of particular nucleon matrix elements rather broadly before turning to a summary of recent results in flavor-changing processes and an assessment of future directions.

### QCD for collider-based BSM searches

#### Theoretical overview: factorization

A general cross section for a collider process involving hadrons is not directly calculable in perturbative QCD. Any such process will involve, at least, the energy scale of the collision and scales associated with masses of the hadrons, apart from other possible scales related to the definition of the jets involved in the process or to necessary experimental cuts. There is therefore an unavoidable dependence on long-distance, non-perturbative scales, and one cannot invoke asymptotic freedom to cope with it. Factorization theorems in QCD allow us to separate, in a systematic way, short-distance and, thus, perturbatively calculable effects from long-distance non-perturbative physics, which are encoded in process-independent objects, such as the parton distribution functions (PDFs). We refer to Sect. 3.2.1 for the theoretical definition of a PDF in the Wilson line formalism and a discussion of its empirical extraction. (A summary of pertinent lattice-QCD results, notably of the lowest moment of the isovector PDF, can be found in Sect. 3.2.5a.) Factorization theorems are, therefore, essential to QCD calculations of hadronic hard-scattering processes. The simpler structure of emissions in the soft and collinear limits, which can generate low-virtuality states, are at the basis of factorization proofs. Factorized forms for the cross sections (see the next section and Sect.  3.2.1 for some examples) can be obtained via diagrammatic methods in perturbative QCD [51] or, alternatively, by employing effective field theories (EFTs) to deal with the different scales present in the process. Soft collinear effective theory (SCET) [790, 1245–1247] is the effective theory that implements the structure of soft and collinear interactions at the Lagrangian level, and it has been extensively used in the last years for many different processes, along with traditional diagrammatic approaches. Establishing a factorized form for the cross section is also the first necessary step for performing resummations of logarithmically enhanced terms, which are key for numerical accuracy in certain portions of phase space. In the following, we discuss a few illustrative examples, which allow us not only to glimpse state-of-the-art techniques but also to gain an impression of the current challenges.

#### Outcomes for a few sample processes

We begin with single vector-boson ($W/Z/\gamma$) production in hadron-hadron collisions in order to illustrate an application of the procedure known as threshold resummation. The transverse momentum, $p_\mathrm{T}$, spectrum for these processes is known at NLO [1248–1250], and there is ongoing work to obtain the NNLO corrections. This is an extremely challenging calculation, but even without it one can improve the fixed-order results by including the resummation of higher-order terms that are enhanced in certain limits. In some cases, such resummations of the fixed-order results are necessary in order to obtain a reasonable cross section. In particular, we focus now on the large-$p_\mathrm{T}$ region of the spectrum, where enhancements related to partonic thresholds can appear. By a partonic threshold we mean configurations in which the colliding partons have just enough energy to produce the desired final state. In these cases, the invariant mass of the jet recoiling against the vector boson is small, and the perturbative corrections are enhanced by logarithms of the jet mass over $p_\mathrm{T}$. The idea is that one can expand around the threshold limit and resum such terms. For single-particle production this was first achieved at next-to-leading-logarithmic (NLL) accuracy in [1251]. In general the cross section also receives contributions from regions away from the partonic threshold, but due to the rapid fall-off of the PDFs at large $x$ the threshold region often gives the bulk of the perturbative correction. SCET offers a convenient, well-developed framework in which to perform such resummations, and allows one to push them to higher orders. A typical factorized form for the partonic cross section $d\hat{\sigma }$, for example in the $q\bar{q}\rightarrow gZ$ channel, looks schematically as follows

5.1 $$\begin{aligned} d\hat{\sigma } \propto H \, \int \! \mathrm {d} k \, J_g(m_X^2-(2E_J)k) S_{q\bar{q}}(k), \end{aligned}$$

with $m_X$ and $E_J$ the invariant mass and energy of the radiation recoiling against the vector boson, respectively. The jet function $J_g$ describes collinear radiation initiated (in this case) by the gluon $g$ present at Born level, the soft function $S_{q\bar{q}}$ encodes soft radiation, and $H$ is the hard function which contains short-distance virtual corrections. The argument of $J_g$ in (5.1) can be understood by recalling that the recoiling radiation $p_X^{\mu }$ is almost massless, i.e., it consists of collinear radiation $p_J^{\mu }$ and additional soft radiation $p_\mathrm{S}^{\mu }$. We can then write $m^2_X=p_X^2=(p_J^{\mu }+p_\mathrm{S}^{\mu })^2=p_J^2+2p_J\cdot p_\mathrm{S}$, up to terms of order $p_\mathrm{S}^2\ll p_J^2$; the collinear radiation can be approximated at leading order as $p_J^{\mu }\sim E_Jn_J^{\mu }$, with $n_J^{\mu }$ a light-like vector, and we obtain $p_J^2=m_X^2-(2E_J)k$, where $k\equiv n_J\cdot p_\mathrm{S}$ is the only component of the soft radiation that is relevant in the threshold limit. The hadronic cross section $\mathrm{d}\sigma$ is given by a further convolution with the PDFs $f_a$ as

5.2 $$\begin{aligned} \mathrm{d}\sigma \propto \sum _{ab=q,\bar{q},g}\int \mathrm{d}x_1\mathrm{d}x_2f_a(x_1)f_b(x_2)\left[ d\hat{\sigma }_{ab}\right] , \end{aligned}$$

where we include a sum over all allowed partonic channels $ab$. Resummation has now been achieved at NNLL accuracy in Refs. [1252–1254] using SCET techniques, which are based on the renormalization group (RG) evolution of the hard, jet, and soft functions. Some NNLL results obtained using diagrammatic methods have also been presented in [1255]. All ingredients required to achieve N$^3$LL accuracy within the SCET framework are essentially known [1256–1263]. A phenomenological analysis at that unprecedented level of accuracy, combined with the inclusion of electroweak corrections which are enhanced by logarithms of the $Z$ or $W$ mass over $p_\mathrm{T}$ [1264], can be expected to appear in the near future. These predictions can then be used, for instance, to constrain the $u/d$ ratio of PDFs at large $x$ (to which we return again in Sect. 5.2.4 from a lower-energy point of view), and to help estimate the $Z(\rightarrow \nu \bar{\nu })+$jets background to new heavy-particle searches [1265] at the LHC.

The same vector-boson production process but in the opposite limit, i.e., at low $p_\mathrm{T}$, is a classic example in which resummation is essential to obtain reasonable predictions, since the perturbative fixed-order calculation diverges. An all-orders resummation formula for this cross section at small $p_\mathrm{T}$ was first obtained in [90]. All ingredients necessary for NNLL accuracy have been computed. Predictions for the cross section at this level of accuracy are discussed in Refs. [105, 106, 1266]. The factorized formulas for this process are more involved than the corresponding ones for threshold resummation in the previous paragraph. In the SCET language, they involve what is sometimes called a “collinear factorization anomaly.” This means that the treatment of singularities present in SCET diagrams requires the introduction of additional regulators, in addition to the usual dimensional regularization, such as an analytic phase-space regularization [112], or, alternatively, one can also use the so-called “rapidity renormalization group” formalism [111], which is based on the regularization of Wilson lines. In any case, this generates some additional dependence (the aforementioned collinear anomaly) on the large scale of the process, $Q$, with respect to what one might otherwise expect. There are, by now, well-understood consistency conditions [105, 1267] that restrict the form of this $Q$ dependence to all orders, and the factorization formula remains predictive and useful. This nuance is directly related to the definition and regularization of the TMD PDFs, which appear in the factorization formula; see Sect. 3.2.1 for further discussion of TMD PDFs. Similar issues also appear when studying the evolution of double parton distribution functions in double-parton scattering (DPS) processes [1268]; further discussion on DPS is given in Sect. 5.2.4.

As we discuss in the next section, much of the current effort is, of course, devoted to the study of the Higgs and its properties. Let us just highlight here one example where good control over QCD effects is necessary, and for which recent progress has been significant.

To optimize the sensitivity of the analyses, Higgs-search data are often separated into bins with a specific number of jets in the final state. In particular, for the Higgs coupling measurements and spin studies, the $H\rightarrow W^+W^{-}$ decay channel is quite relevant; but in this channel there is a large background coming from $t\bar{t}$ production, which after the tops decay can produce a $W^+W^{-}$ pair together with two $b$-quark jets. To reduce this background, events containing jets with transverse momentum above a certain threshold are rejected, i.e., one focuses on the 0-jet bin, which is also known as the jet-veto cross section. This restriction on the cross section enhances the higher-order QCD corrections to the process, by terms that contain logarithms of the transverse-momentum veto scale (typically around 25–30 GeV) over the Higgs mass. One should be careful when estimating the perturbative uncertainty of fixed-order predictions for the jet-veto cross section, since the cancellation of different effects can lead to artificially small estimations. A reliable procedure to estimate it was presented in [1269], and the outcome is that the perturbative uncertainty for the jet-veto cross section is around $20~\%$, which is comparable to the current statistical experimental uncertainty and larger than the systematic one. It is therefore desirable to improve these theoretical predictions. There has been a lot of progress, starting with [1270], which showed that the resummation could be performed at NLL accuracy, and its authors also computed the NNLL terms associated with the jet radius dependence. Subsequently, resummation of these logarithms was performed at NNLL precision [1271–1275]. An all-orders factorization formula was also put forward in Refs. [1272, 1275] within the SCET framework; its adequacy, though, has been questioned in Refs. [1271, 1274]. In any case, the accuracy for this jet-veto cross section has significantly improved, and there is room to continue improving the understanding of jet-veto cross sections and their uncertainty.

Related to the discussion of the previous paragraph, one would also like to have resummed predictions for $N$-jet processes, by which we mean any process with $N$ hard jets. Although there has been important recent progress [1276, 1277] regarding the structure of infrared singularities in gauge theories, connecting them to $N$-jet operators and its evolution in SCET, many multi-jet processes involve so-called nonglobal logarithms [1278]. These are logarithms that arise in observables that are sensitive to radiation in only a part of the phase space. In general they appear at the NLL level, and although several explicit computations of these kinds of terms have been performed, it is not known how to resum them in general. Their presence, therefore, hinders the way to resummation for general $N$-jet cross sections. One might be forced to switch to simpler observables; see, e.g., Ref. [1279], to be able to produce predictions at higher-logarithmic accuracy.

Giving their present significance, jet studies command a great part of the current focus of attention. In particular, driven in part by the new possibilities that the LHC offers, the study of jet substructure, and jet properties in general, is a growing field. Jet substructure analysis can allow one, for instance, to distinguish QCD jets from jets coming from hadronic decays of boosted heavy objects; see, e.g., Refs. [1280–1282]. Many other new results have appeared recently, and one can certainly expect more progress regarding jet studies in the near future. This will hopefully allow for improved identification techniques in searches for new heavy particles.

#### LHC results: Higgs and top physics

The announcement in mid-2012 of the discovery of a boson of mass near 125 GeV while searching for the SM Higgs electrified the world and represents a landmark achievement in experimental particle physics. It is decidedly a new physics result and one which we hope will open a new world to us. The discovery raises several key questions: What is its spin? Its parity? Is it pointlike or composite? One particle or the beginning of a multiplet? Does it couple like the SM Higgs to quarks, leptons, and gauge bosons? No other significant deviations from SM expectations have as yet been observed, falsifying many new-physics models. Nevertheless, plenty of room remains for new possibilities, both within and beyond the Higgs sector, and we anticipate that resolving whether the new particle is “just” the SM Higgs will require years of effort, possibly extending beyond the LHC. The ability to control QCD uncertainties will be essential to the success of the effort, as we can already illustrate.

**Fig. 34:**
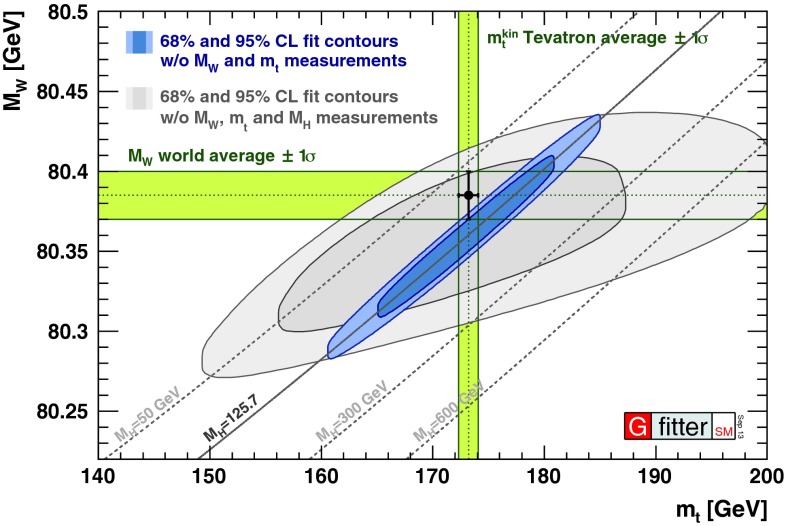
Direct and indirect determinations of the $W$-boson and $t$-quark masses within the SM from measurements at LEP [698] and the Tevatron [1288], and from Higgs mass $M_\mathrm{H}$ measurements at the LHC [1283, 1284]. The *nearly elliptical contours* indicate constraints from global fits to electroweak data, note http://cern.ch/gfitter [1290], exclusive of direct measurements of $M_W$ and $m_t$ from LEP and the Tevatron [1289, 1291]. The smaller (larger) *contours* include (exclude) the Higgs mass determinations from the LHC. We show a September, 2013 update from a similar figure in [1289] and refer to it for all details

*a. Higgs production and decay* The observation of the Higgs candidate by the ATLAS (http://atlas.ch [1283]) and CMS (http://cern.ch/cms [1284]) collaborations was based on the study of the $H\rightarrow \gamma \gamma$ and $H\rightarrow ZZ \rightarrow 4{\ell }$, with ${\ell } \in e,\mu$, channels, due to the excellent mass resolution possible in these final states [1285, 1286]. The finding was supported by reasonably good statistics, exceeding 4$\sigma$ significance, in the four-lepton channel, for which the background is small, whereas the background in $H\rightarrow \gamma \gamma$ is rather larger. Further work has led to an observed significance of $6.7\sigma$ (CMS) in the $H\rightarrow ZZ$ channel alone, and to studies of the $H\rightarrow WW$, $H\rightarrow bb$, $H\rightarrow \tau \tau$ decay modes as well [1286]. It is worth noting that the Bose symmetry of the observed two-photon final state precludes a $J=1$ spin assignment to the new particle; this conclusion is also supported by further study of $H\rightarrow ZZ\rightarrow 4 {\ell }$ [1287]. Moreover, the finding is compatible with indirect evidence for the existence of a light Higgs boson [1288]. Figure 34 shows a comparison of recent direct and indirect determinations of the $t$ quark and $W$ gauge boson masses; this tests the consistency of the SM. The horizontal and vertical bands result from using the observed $W$ (LEP+Tevatron) and $t$ (Tevatron) masses at 68 % C.L., and global fits to precision electroweak data, once the $t$ and $W$ direct measurements are excluded, are shown as well [1289]. The smaller set of ellipses include determinations of the Higgs mass determinations from the LHC.

**Fig. 35:**
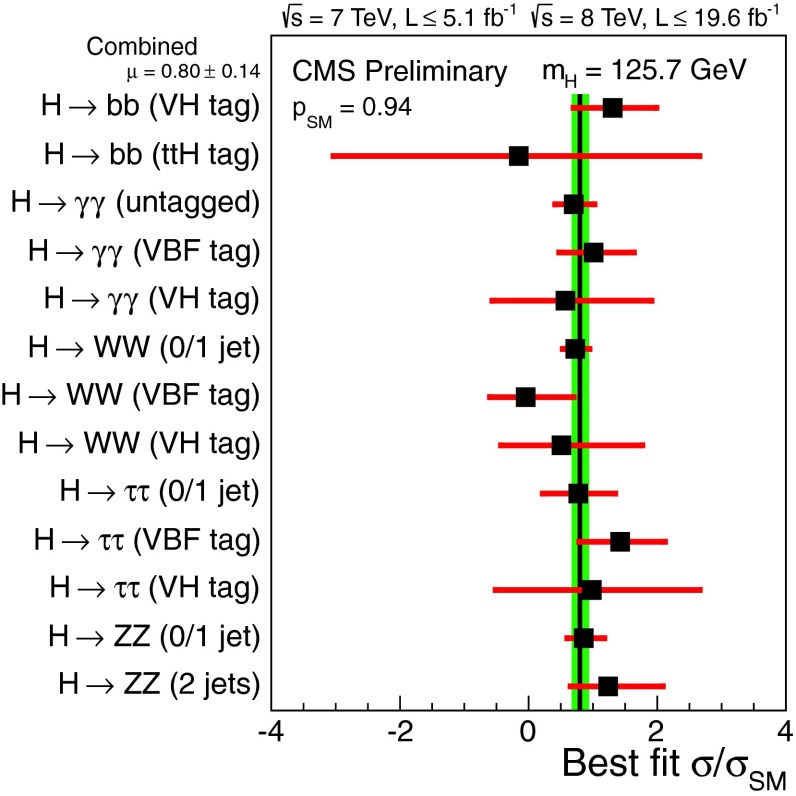
Values of $\sigma /\sigma _\mathrm{SM}$ for particular decay modes, or of subcombinations therein which target particular production mechanisms. The *horizontal bars* indicate $\pm 1\,\sigma$ errors including both statistical and systematic uncertainties; the *vertical band* shows the overall uncertainty. The quantity $\sigma /\sigma _\mathrm{SM}$ (denoted $\mu (x,y)$ in text) is the production cross section times the branching fraction, relative to the SM expectation [1286]. (Figure reproduced from [1286], courtesy of the CMS collaboration.)

We now summarize ongoing studies of the Higgs couplings, as well as of its spin and parity, highlighting the essential role of QCD in these efforts. It is evident that the Higgs discovery opens a new experimental approach to the search for new physics, through the determination of its properties and couplings that are poorly constrained beyond the SM [1292]. The theoretical control over the requisite SM cross sections and backgrounds needed to expose new physics becomes more stringent as the constraints sharpen without observation of departures from the SM. Figure 35 shows the value of $\sigma /\sigma _\mathrm{SM}$, namely, of the production cross section times the branching fraction, relative to the SM expectation [1286], with decay mode and targeted production mechanism, where the latter includes $gg$, VBF, VH (WH and ZH), and $t{\bar{t}}H$ processes. This quantity is usually called $\mu$, and we can define, for production mode $X$ and decay channel $Y$,

5.3 $$\begin{aligned} \mu (X,Y) \equiv \frac{\sigma (X)\mathcal{B}(H\rightarrow Y)}{\sigma _\mathrm{SM}(X)\mathcal{B}_\mathrm{SM}(H\rightarrow Y)} , \end{aligned}$$

noting a global average of $\mu =0.80\pm 0.14$ for a Higgs boson mass of 125.7 GeV [1286]. See Ref. [1293] for further results and discussion and Ref. [1294] for a succinct review. We note that $pp\rightarrow H$ via gluon–gluon fusion is computed to NNLO $+$ NNLL precision in QCD, with an estimated uncertainty of about $\pm 10~\%$ by varying the renormalization and factorization scales [1294, 1295]. In contrast, the error in the computed partial width of $H\rightarrow b\bar{b}$ is about 6 % [1296]. The Higgs partial widths are typically accessed through channels in which the Higgs appears in an intermediate state, as in (5.3). Consequently, the ratio of the Higgs coupling to a final state $Y$ with respect to its SM value, defined as $\kappa _Y^2 = \Gamma (H\rightarrow Y{\bar{Y}})/\Gamma _\mathrm{SM}(H\rightarrow Y{\bar{Y}})$, is determined through a multi-channel fit. The ability of the LHC to probe $\kappa _Y$ has been forecast to be some 10–30 % [1294, 1297, 1298]. Estimates instigated by the U.S.-based Community Planning Study (Snowmass 2013) support these assessments [1292], comparing the sensitivity of the current stage of the LHC (data samples at 7–8 TeV with an integrated luminosity of $20\,\mathrm{fb}^{-1}$) to staged improvements at the LHC and to possible new accelerators, such as differing realizations of a linear $e^+e^{-}$ collider. New backgrounds can appear at the LHC which were not known at LEP; e.g., a previously unappreciated background to the Higgs signal in $H\rightarrow ZZ$ and $H\rightarrow WW$, arising from asymmetric internal Dalitz conversion to a lepton pair, has been discovered [1299]. Nevertheless, even with conservative assessments of the eventual (albeit known) systematic errors, tests of the Higgs coupling to $W$’s or $b$-quarks of sub-10 % precision are within reach of the LHC’s high luminosity upgrade, with tests of sub-1 % precision possible at an $e^+e^{-}$ collider [1292]. These prospects demand further refinements of the existing SM predictions, with concomitant improvements in the theoretical inputs such as $\alpha _\mathrm{s}$, $m_b$, and $m_\mathrm{c}$ [1292].

Current constraints on the quantum numbers of the new boson support a $0^+$ assignment but operate under the assumption that it is *exclusively* of a particular spin and parity. Of course admixtures are possible, and they can reflect the existence of CP-violating couplings; such possibilities are more challenging to constrain. Near-degenerate states are also possible and are potentially discoverable [1300]. ATLAS has studied various, possible spin and parity assignments, namely of $J^P = 0^{-},1^+, 1^{-}, 2^+$, as alternative hypotheses to the $0^+$ assignment associated with a SM Higgs, and excludes these at a C.L. in excess of 97.8 % [1287]. In the case of the $2^+$, however, a specific graviton-inspired model is chosen to reduce the possible couplings to SM particles. It is worth noting that QCD effects play a role in these studies as well. In the particular example of the $H\rightarrow \gamma \gamma$ mode, the $J^P$ assignments of $0^+$ and $2^+$ are compared vis-a-vis the angular distribution of the photons with respect to the $z$-axis in the Collins-Soper frame [1287]. The expected angular distribution of the signal yields in the $0^+$ case is corrected for interference effects with the nonresonant diphoton background $gg\rightarrow \gamma \gamma$ mediated through quark loops [1301].

EFT methods familiar from the study of processes at lower energies also play an important role, and can work to disparate ends. They can be used, e.g., to describe a generalized Higgs sector [1302], providing not only a theoretical framework for the simultaneous possibility of various SM extensions therein [1303, 1304] but also a description of its CP-violating aspects [1305]. In addition, such methods can be used to capture the effect of higher-loop computations within the Standard Model. For example, the effective vertex ($v$ is the Higgs vacuum expectation value) [1306]

5.4 $$\begin{aligned} \mathcal {L}_\mathrm{eff}= \alpha _\mathrm{s} \frac{C_1}{4v} H F^a_{\mu \nu } F^{a\ \mu \nu } \end{aligned}$$

couples the Higgs to the two gluons in a SU(3)$_\mathrm{c}$-gauge-invariant manner. It can capture this coupling in a very efficient way, yielding a difference of less than 1 % between the exact and approximate NLO cross sections for a Higgs mass of less than 200 GeV [1294]. This speeds up Monte Carlo programs, for example. All short-distance information (at the scales of $M_\mathrm{H}$, $m_t$, or new physics) is encoded in the Wilson coefficient $C_1$, which is separately computed in perturbation theory.

**Fig. 36:**
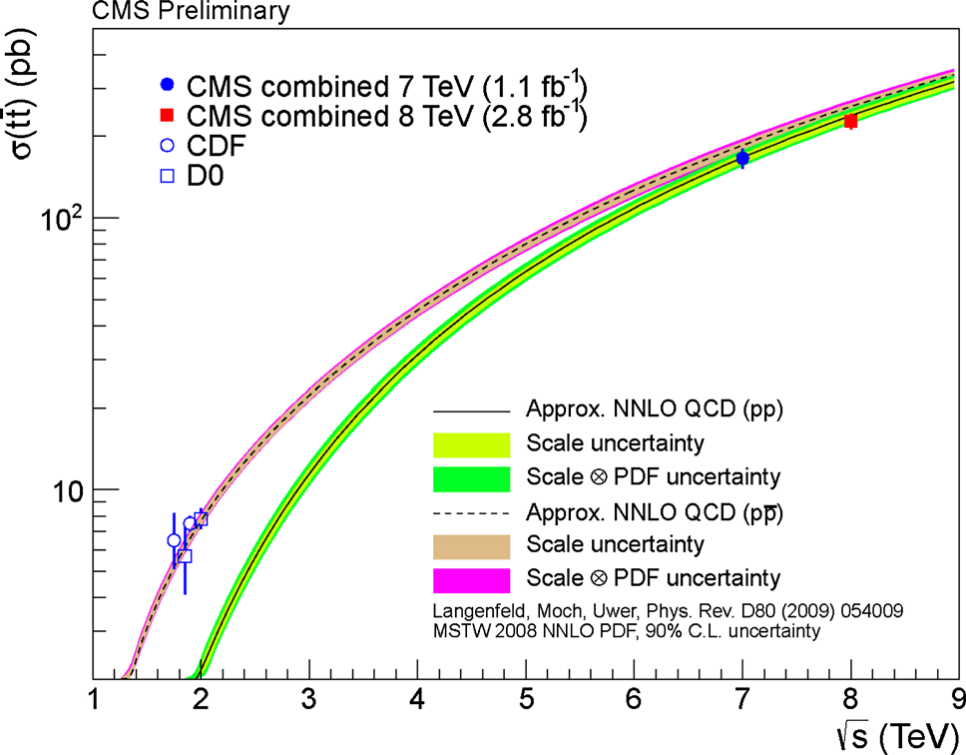
Inclusive cross section for top pair production with center-of-mass energy in $pp$ and $p\bar{p}$ collisions [1307], compared with experimental cross sections from CDF, D0, ATLAS, and CMS [1314]. (Figure reproduced from [1314], courtesy of the CMS collaboration.)

*b. Top quark studies* From the Tevatron to the LHC, the cross section for top-quark pair production $\sigma (t\bar{t})$, in Fig. 36, grows by a factor of roughly $30$ due to the larger phase space; from 7 pb at the 1.96 TeV center-of-mass (CM) energy of the Tevatron to some 160 pb at 7 TeV and to some 220 pb at 8 TeV. We refer to [1307] cross-section predictions at 14 TeV and to [1308] for recent cross-section results from CMS and ATLAS.

A good part of $t\bar{t}$ production is near threshold, with a small relative velocity between the two heavy quarks. A non-relativistic, fixed-order organization of the perturbative series is appropriate. Supplementing such a NNLO calculation with a resummation of soft and Coulomb corrections at NNLL accuracy, a computation of $\sigma (t\bar{t})$ at the LHC (7 TeV) of $10$ pb precision has been reported [1309–1311]. More generally, the predictions show a residual theoretical uncertainty of some $3$–$4~\%$, with an additional $4$–$4.5~\%$ uncertainty from the PDFs and the determination of $\alpha _\mathrm{s}$ [1310, 1311]. Measurements of the $t\bar{t}$ inclusive cross section can thus be used to extract the top-quark mass, yielding a result of $m_t=171.4 \mathop {{}_{-5.7}}\limits ^{+5.4}\,\mathrm{GeV}$ [1310], in good agreement with the direct mass determination from the Tevatron, $m_t=173.18 \pm 0.56\,(\mathrm{stat.})\,\pm 0.75\,(\mathrm{syst.})\, \mathrm{GeV}$ [1288], but less precise. The measurement of near-threshold $t\bar{t}$ production at an $e^+e^{-}$ collider, in contrast, can reduce the precision with which $m_t$ is known by a factor of a few, spurring further theoretical refinements [1312, 1313]. Moreover, in this case, the connection to a particular top mass definition is also crisp.

*c. Collider searches for new particles* ATLAS and CMS continue to search for the new physics effects expected in various extensions of the SM. All searches, thus far, yield results compatible with the SM. Certain efforts concern searches for high mass $t\bar{t}$ resonances, such as could be generated through a high mass (leptophobic) $Z^\prime$ or Kaluza-Klein gluon, or searches for top $+$ jet resonances, such as could be generated through a high mass $W^\prime$ [1315–1319]. Experimental collaborations face a new problem in collecting large top samples at the higher LHC energies: often the $t$ and $\bar{t}$ fly away together in a boosted frame, so that the SM decay with visible particles

5.5 $$\begin{aligned} t\bar{t}\rightarrow Wb W\bar{b}\ \ (\rightarrow 6\ \mathrm{jets\ or \ \rightarrow 2 \ jets} + 2 \mathrm{leptons} ) \end{aligned}$$

contains several jets that may overlap yielding “fat jets,” for which new algorithms are being developed [1320].

The constraints are sharpest for $t\bar{t}$ resonances, which decay into lepton pairs, with exclusion limits of 2.79 TeV at 95 % C.L. for a $Z^\prime$ (with SM-like couplings) decaying into $e^+e^{-}$. In contrast, the 95 % C.L. exclusion limit on a leptophobic $Z^\prime$ decaying into $t\bar{t}$ is greater than 1.5 TeV [1319]. The parity programs at JLab (note, e.g., HAPPEX, http://hallaweb.jlab.org/experiment/HAPPEX and Q-weak, http://www.jlab.org/qweak) and MESA (http://www.prisma.uni-mainz.de/mesa.php) at Mainz are geared towards searches for similar objects, in complementary regions of parameter space, through the precision measurement of parity-violating asymmetries at low momentum transfers [705, 1321]. Moreover, a unique window on the possibility of a leptophobic $Z^\prime$ can come from the study of parity-violating deep inelastic scattering of polarized electrons from deuterium [1322].

Significant indirect constraints exist on the possibility of an extra chiral generation of quarks from the observation of $H\rightarrow \gamma \gamma$ [1323], as well as through the apparent production of the Higgs through $gg$ fusion. Direct searches are mounted, however, for certain “exotic” variants of the extra generation hypothesis, be they vector-like quarks, or quarks with unusual electric charge assignments [1316, 1318]. All searches thus far are null, and $(5/3)e$-charged up quarks, e.g., are excluded for masses below 700 GeV at 95 % C.L. [1319].

Because no new particle (beyond the Higgs-like particle) has yet appeared in the mass region below 1 TeV, direct searches for a new resonance $R$ will likely extend to higher mass scales. This will push the QCD inputs needed for PDF fits to the limits of currently available phase space, and it is worth exploring the prospects for better control of such quantities. Precision determinations of the particle properties and couplings of the particles we know also drive a desire to understand the PDFs as accurately as possible. We also refer to Sect. 3.2.1 for a discussion of PDFs and their uncertainties.

#### Uncertainties from nucleon structure and PDFs

In order to produce a previously unknown particle $R$, the colliding partons in the initial state, as in for instance $g(x_1) g(x_2)\rightarrow R +X$, must each carry a significant fraction of the proton’s momentum. This makes constraining parton distribution functions at large Bjorken $x$, particularly for $x>0.5$, ever more important as the mass of $R$ increases. As we have seen, the PDF and scale uncertainties are the largest uncertainties in the predicted inclusive $t\bar{t}$ cross section. Such uncertainties are also important to the interpretation of ultra-high–energy neutrino events observed at Ice Cube [1324], whose rate may exceed that of the SM. There is currently an effort [135, 1325, 1326] to investigate this issue by combining the traditional CTEQ fits in the large-$x$ ($x\rightarrow 1$) region with JLab data at lower energies. These efforts will likely wax with importance in time because, the 12 GeV upgrade at JLab will allow greatly expanded access to the large-$x$ region [1327]. Various complications emerge as $x\rightarrow 1$, and it is challenging to separate the additional contributions that arise. In particular, large logarithms, the so-called Sudakov double logarithms, appear in the $x\rightarrow 1$ region, and they need to be resummed in order to get an accurate assessment of the cross section. To this end the $x\rightarrow 1$ region has been subject to extensive theoretical investigation, both in traditional approaches based on factorization theorems [1328, 1329] and in effective field theory [1330–1334]. Moreover, studies of deep inelastic scattering in nuclei require the assessment of Fermi-motion effects as well. The former issue is skirted in traditional global fits, based on structure functions in leading-twist, collinear factorization, by making the cut on the hadronic invariant mass $W$ large, such as in [131] for which $W^2 \ge 15\,\mathrm{GeV}^2$. Here, $W^2 =M^2 + Q^2 (1-x)/x$. The global-fit approach in [135, 1325, 1326] includes both large-$x$ and nuclear corrections and allows the $W$ cut to be relaxed to $W\sim 1.7\,\mathrm{GeV}$ [1335].

To obtain the $d$ quark distribution, for example, one uses the data on the unpolarized structure function $F_2$, e.g., from deep inelastic scattering on the proton and neutron, to find

5.6 $$\begin{aligned} \frac{d(x)}{u(x)} = \frac{4 F_{2n}(x) - F_{2p}(x)}{4F_{2p}(x) -F_{2n}(x)} , \end{aligned}$$

where, for brevity, we suppress the $Q^2$ dependence. Since there are no free neutron targets, the experiments are performed with few-body nuclei, either the deuteron or $^3$He. For $x$ above $x\simeq 0.5$, the nuclear corrections become large. The CTEQ-JLab fits employ the collinear factorization formula

5.7 $$\begin{aligned} F_{2d}(x,Q^2)&= \sum _{N=p,n} \int dy S_{N/A}(y,\gamma ) F_2(x/y,Q^2) \nonumber \\&+ \Delta ^\mathrm{off}(x,Q^2) , \end{aligned}$$

where the deuteron structure function is computed from the parametrized nucleon $F_2$, the modeled off-shell correction $\Delta ^\mathrm{off}$, and the nuclear smearing function $S_{N/A}$, computed from traditional nuclear potential theory based on the Paris, Argonne, or CD-Bonn interactions. There is clearly room for QCD-based progress in these computations. The notion of [135, 1336] is that data on the $W^\pm$ charge asymmetry from the Tevatron [1337, 1338] can be used to fix the $d(x)/u(x)$ ratio at large $x$, and then precision nuclear experiments can be used to fix the nuclear corrections. Future JLab experiments, which are less sensitive to nuclear effects, can then be used to test the procedure [1336].

Of course, the higher energy run of the LHC at 14 TeV, scheduled for 2015, should also lower the $x$ needed for a given energy reach. Taking 2 TeV as the reference CM energy for a gluon–gluon collision, doubling the LHC energy from 7 to 14 TeV increases the parton luminosity by a factor of 50 [1339], making the new physics reach at $\mathcal{O}(1\,\text {TeV})$ less sensitive to the large $x$ behavior of the PDFs. At 14 TeV the parton luminosity (taking this as a crude proxy for $x$) of the 2 TeV gluon–gluon subprocess in the 7 TeV collision is found at a CM energy of 3.3 TeV [1339]. Sorting out the PDFs in the large-$x$ region may prove essential to establishing new physics.

Another issue for new physics searches and Higgs physics is double-parton scattering [1268, 1340]. Two hard partons collide if they coincide within a transverse area of size $1/Q^2$ out of the total $1/\Lambda _\mathrm{QCD}^2$. The flux factor being $1/\Lambda _\mathrm{QCD}^2$, the probability of one hard collision scales as $\hat{\sigma }_1 \propto ({1}/{\Lambda _\mathrm{QCD}^2}) ( {\Lambda _\mathrm{QCD}^2}/{Q^2})$. The probability of a double collision in the same $pp$ event (this is not the same as pile up, which is the aftermath of multiple, nearly simultaneous $pp$ events) is thus power-suppressed, $\hat{\sigma }_2 \propto ({1}/{\Lambda _\mathrm{QCD}^2}) ( {\Lambda _\mathrm{QCD}^2}/{Q^2})^2$. The rate is small but still leads to a background about three times the signal in Higgs processes such as $pp\rightarrow WH\rightarrow l\bar{\nu }b\bar{b}$ [1341]. It also entails power corrections to double Drell–Yan processes, an important background to four-lepton Higgs decays. Like-sign $W^+W^+$ production has long been recognized as a viable way to identify double-parton scattering [1342, 1343] because this final state is not possible in single-parton scattering unless two additional jets are emitted (due to charge and quark-number conservation). It comes to be dominated by double scattering when the particle pairs come out almost back-to-back (typically $|{\mathbf {p}}_{1\mathrm{T}} + {\mathbf {p}}_{2\mathrm{T}}| \sim \Lambda _\mathrm{QCD}$).

One might suppose the differential cross section for double-parton scattering could be described as [1344]

5.8 $$\begin{aligned}&\frac{\mathrm{d}\sigma ^{DPS}}{\mathrm{d}x_1\mathrm{d}x_2\mathrm{d}x_3\mathrm{d}x_4} \propto \nonumber \\&\quad \times \int \mathrm{d}^2z_\perp F_{ij}(x_1,x_2,z_\perp ) F_{kl}(x_3,x_4,z_\perp ) \hat{\sigma }_{ik} \hat{\sigma }_{jl} , \end{aligned}$$

employing a distribution-like function $F$ to describe the probability of finding the two partons in the proton at $z_\perp$ from each other in the plane perpendicular to the momentum, with given momentum fractions $x_i$. Quantum interference is intrinsic to this process, however, so that some knowledge of the proton at the wave function or amplitude level is needed, as a purely probabilistic description is insufficient. We refer to [1268] for a detailed analysis.

#### Complementarity with low-energy probes

Searches for unambiguous signs of new physics at high-energy colliders have so far proved null; it may be that new physics appears at yet higher energy scales or that it is more weakly coupled than has been usually assumed. In the former case, a common theoretical framework, which is model-independent and contains few assumptions, can be used to connect the constraints from collider observables to those from low-energy precision measurements; we provide an overview thereof in the next section. In the latter case, an explicit BSM model is required to connect experimental studies at high and low energy scales, and the minimal supersymmetric standard model (MSSM) is a particularly popular example. The impact of permanent electric dipole moment (EDM) searches at low energies, for example, on the appearance of CP-violating terms in the softly broken supersymmetric sector of the MSSM and its broader implications have been studied for decades [1345–1350]. Computations of the various QCD matrix elements which appear are important to assessing the loci of points in parameter space which survive these constraints; we discuss the state of the art, albeit in simpler cases, in Sect. 5.4.4.

In the event that new physics is beyond the reach of current colliders, the connection between experimental probes at the highest and lowest energies mentioned is particularly transparent and certainly two-way. Although collider experiments limit new-physics possibilities at low energies, it is also the case that low-energy experiments limit the scope of new-physics at colliders. Before closing this section, we consider an example of how a model-independent approach employing effective Lagrangian techniques can be used in the top-quark sector as well [1351]. Usually such techniques are employed assuming the accessible energy to be no larger than the $W$ mass [1352, 1353]. In particular, we consider the possibility that the top quark itself could have a permanent (chromo)electric or (chromo)magnetic dipole moment. This is particularly natural if the top quark is a composite particle [1354], and the large top-quark mass suggests that the effects could well be large [1355]. Although such effects could potentially be probed directly through spin observables [1356], constraints from the neutron EDM also operate [1357, 1358], to yield a severe constraint on the chromoelectric top-quark operator through its effect on the coefficient $w$ of the Weinberg three-gluon operator

5.9 $$\begin{aligned} {\mathcal L}_{W3g}= -\frac{w}{6} f^{abc} \varepsilon ^{\mu \nu \lambda \rho } (F^a)_{\mu \sigma } (F^b)^\sigma _\nu (F^\mathrm{c})_{\lambda \rho } \end{aligned}$$

at low energies [1357], where $f^{abc}$ are SU(3) structure constants. Turning to the specific numerical details, the QCD matrix element of the Weinberg operator in the neutron is needed, and the QCD sum rule calculation of [1359] has been employed to obtain the limits noted [1357]. (See Sects. 5.4.3 and 5.4.4 for further discussion of matrix elements for EDMs.) Stronger limits on the color-blind dipole moments, however, come from $b\rightarrow s \gamma$ and $b\rightarrow s {\ell }^+{\ell }^{-}$ decays [1357, 1360]. In the face of such constraints, the space of new-physics models to be explored at the LHC is significantly reduced [1357, 1358], and presumably can be sharpened further, even in the absence of additional experimental data, if the non-perturbative matrix element can be more accurately calculated. In the sections to follow we will find further examples of low-to-high-energy complementarity.

### Low-energy framework for the analysis of BSM effects

The SM leaves many questions unanswered, and the best-motivated models of new physics are those which are able to address them. Commonly, this is realized so that the more fundamental theory has the SM as its low-energy limit. It is thus natural to analyze the possibility of physics beyond the SM within an effective field theory framework. To do this we need only assume that we work at some energy $E$ below the scale $\Lambda$ at which new particles appear. Consequently for $E< \Lambda$ any new degrees of freedom are “integrated out,” and the SM is amended by higher-dimension operators written in terms of fields associated with SM particles [1361]. Specifically,

5.10 $$\begin{aligned} \mathcal{L}_\mathrm{SM} \rightarrow \mathcal{L}_\mathrm{SM} + \sum _i \frac{c_i}{\Lambda ^{D-4}} {\mathcal{O}^D_i} , \end{aligned}$$

where the new operators $\mathcal{O}_i^D$ have dimension $D>4$. We emphasize that $\mathcal{L}_\mathrm{SM}$ contains a dimension-four operator, controlled by $\bar{\theta }$, that can also engender CP-violating effects, though they have not yet been observed. The experimental limit on the neutron EDM implies $\bar{\theta }<10^{-10}$ [1232], though the underlying reason for its small value is unclear. This limitation is known as the “strong CP problem”. If its resolution is in a new continuous symmetry [1362] that is spontaneously and mechanically broken at low energy, then there is a new particle, the axion [1363, 1364], which we may yet discover [1365, 1366]. The higher-dimension operators include terms which manifestly break SM symmetries and others which do not.

Since flavor-physics observables constrain the appearance of operators that are not SM invariant to energies far beyond the weak scale [1367–1369], it is more efficient to organize the higher-dimension terms so that only those invariant under SM electroweak gauge symmetry are included. Under these conditions, and setting aside B- and L-violating operators, the leading-order (dimension-six) terms in our SM extension can be found in [1352, 1353]. Nevertheless, this description does not capture all the possibilities usually considered in dimension six because of the existence of neutrino mass. The latter has been established beyond all doubt [1], though the need for the inclusion of dynamics beyond that in the SM to explain it has, as yet, not been established. To be specific, we can use the Higgs mechanism to generate their mass.^11^ Since the neutrinos are all light in mass, to explore the consequences of this possibility, we must include three right-handed neutrinos explicitly in our description at low energies [1371]. Finally, if we evolve our description (valid for $E<\Lambda$) to the low energies ($E \ll M_W, \Lambda$) appropriate to the study of the weak decays of neutrons and nuclei, we recover precisely 10 independent terms, just as argued long ago by Lee and Yang starting from the assumption of Lorentz invariance and the possibility of parity nonconservation [1372]. The latter continues to be the framework in which new physics searches in $\beta$-decay are analyzed, as discussed, e.g., in [1240, 1373–1375].

In order to employ the low-energy quark and gluon operator framework we have discussed in a chiral effective theory in nucleon degrees of freedom, nucleon, rather than meson, matrix elements need to be computed. Nucleon matrix elements are generally more computationally demanding than meson matrix elements in lattice QCD, since the statistical noise grows with Euclidean time $t$ as $\exp [(M_\mathrm{N}-3 M_\pi /2)t]$ for each nucleon in the system. Thus, results with high precision in the nucleon sector lag those in the meson sector. Furthermore, extrapolating to the physical light-quark masses is more challenging for baryons, since chiral perturbation theory converges more slowly. The latter issue is likely to be brought under control in the near future, as ensembles of lattices begin to be generated with physical $u$ and $d$ (and $s$ and $c$) quark masses. This should greatly reduce the systematic uncertainties. Other systematics, such as finite-volume effects, renormalization, and excited-state contamination can be systematically reduced by improved algorithms and by increasing the computational resources devoted to the calculations. We refer to Sect. 3.2.5a for additional discussion.

One interesting idea from experimental physics is to perform “blind” analyses, so that the true result is hidden while the analysis is performed. Concretely what this means is that the result should only be revealed after all the systematics have been estimated. This technique has begun to be employed in lattice-QCD calculations, notably in the computation of the exclusive semileptonic decay matrix elements needed to determine the CKM matrix elements $|V_{cb}|$ and $|V_{ub}|$ [931, 1376]. It would be advantageous to implement this approach in lattice-QCD calculations of nucleon matrix elements as well, so that an analysis of systematic effects could be concluded on grounds independent of the specific result found. Blind analysis would help in ensuring an extremely careful analysis of systematics, and we hope the lattice community will choose to follow this approach in the next few years.

We now turn to the analysis of particular low-energy experiments to the end of discovering physics BSM and the manner in which theoretical control over confinement physics can support or limit them.

### Permanent EDMs

#### Overview

The (permanent) EDM of the neutron is a measure of the distribution of positive and negative charge inside the neutron; it is nonzero if a slight offset in the arrangement of the positive and negative charges exists. This is possible if interactions are present which break the discrete symmetries of parity P and time reversal T. In the context of the CPT theorem, it also reflects the existence of CP violation, i.e., of the product of charge conjugation C and parity P, as well. Consequently, permanent EDM searches probe the possibility of new sources of CP violation at the Lagrangian level. The EDM $\mathbf {d}$ of a nondegenerate system is proportional to its spin $\mathbf {S}$, and it is nonzero if the energy of the system shifts in an external electric field $\mathbf {E}$, with an interaction energy proportional to $\mathbf {S}\cdot \mathbf {E}$.

As we have already noted, the SM nominally possesses two sources of CP violation, the single phase $\delta$ in the Cabibbo–Kobayashi–Maskawa (CKM) matrix and the coefficient $\bar{\theta }$ which controls the T-odd, P-odd product of the gluon field-strength tensor and its dual, namely $\bar{\theta } ({\alpha _\mathrm{s}}/{8\pi }) F^a \tilde{F}^a$. Experimental studies of CP violation in the B system have shown that $\delta \sim \mathcal{O}(1)$ [1368, 1369], whereas neutron EDM limits have shown that the second source of CP violation does not appear to operate. Even if a physical mechanism exists to remove the appearance of $\bar{\theta }$, higher-dimension operators from physics BSM may still induce it, so that we use experiment to constrain this second source, as well as CP-violating effects arising from other BSM operators.

The CKM mechanism of CP violation does give rise to nonzero permanent EDMs; however, the first nontrivial contributions to the quark and charged lepton EDMs come in three- and four-loop order (for massless neutrinos), respectively, so that for the down quark $|d_d| \sim 10^{-34}\, e\hbox {-}{\mathrm {cm}}$ [1377, 1378]. Nevertheless, there exists a well-known, long-distance chiral enhancement of the neutron EDM (arising from a pion loop and controlled by $\log (m_\pi /M_\mathrm{N})$), and estimates yield $|d_n| \sim 10^{-31}$–$10^{-33}\, e\hbox {-}{\mathrm {cm}}$ [1379–1381], making it relatively larger but still several orders of magnitude below the current experimental sensitivity. It is worth noting that the nucleon’s intrinsic flavor content can also modify an EDM estimate [1382–1384]. Finally, if neutrinos are massive Majorana particles, then the electron EDM induced by the CKM matrix can be greatly enhanced, though not sufficiently to make it experimentally observable [1385]. (Neutrino mixing and Majorana-mass dynamics can also augment the muon EDM in the MSSM in a manner which evades $e$–$\mu$ universality, motivating a dedicated search for $d_{\mu }$ [1386].) A compilation of the results from various systems is shown in Table 15.

**Table 15:** Upper limits on EDMs ($|d|$) from different experiments. For the “Nucleus” category, the EDM values are of the $^{199}$Hg atom that contains the nucleus. No *direct* limit yet exists on the proton EDM, though such could be realized through a storage-ring experiment. Here we report the best inferred limit in brackets, which is determined by asserting that the $^{199}$Hg limit is saturated by $d_p$ exclusively. Table adapted from [1387]

Category	EDM Limit ($e-{\mathrm {cm}}$)	SM Value ($e-{\mathrm {cm}}$)
Electron	$1.0\times 10^{-27}\,(90\%\, \mathrm{C.L.})$ [1388]	10$^{-38}$
Muon	$1.9\times 10^{-19}\,(95\%\, \mathrm{C.L.})$ [1389]	10$^{-35}$
Neutron	$2.9\times 10^{-26}\,(90\%\, \mathrm{C.L.})$ [1232]	10$^{-31}$
Proton	$[7.9\times 10^{-25}]\quad \quad \quad \quad$ [1390]	10$^{-31}$
Nucleus	$3.1\times 10^{-29}\,(95\%\, \mathrm{C.L.})$ [1390]	10$^{-33}$

#### Experiments, and their interpretation and implications

The last few years have seen an explosion of interest in experimental approaches to searches for electric dipole moments of particles composed of light quarks and leptons. This increased scientific interest has developed for many reasons. First, the power of the existing and achievable constraints from EDM searches on sources of CP violation BSM has become more and more widely recognized. Moreover, other sensitive experimental tests of “T” invariance come from particle decays and reactions in which the observables are only motion-reversal odd and thus do not reflect true tests of time-reversal invariance [1391]. Such can be mimicked by various forms of final-state effects which eventually limit their sensitivity. In contrast, the matrix element associated with an intrinsic particle EDM has definite transformation properties under time reversal because the initial state and the final state are the same. The consequence is that an EDM search is one of the few true null tests for time-reversal invariance. Consequently an upper bound on an EDM constitutes a crisp, non-negotiable limit, and a positive observation of an EDM at foreseeable levels of sensitivity would constitute incontrovertible evidence for T violation. Moreover, since the SM prediction is inaccessibly small, as shown in Table 15, it would also speak directly to the existence of new physics. Popular models of new physics at the weak scale generate EDMs greatly in excess of SM expectations, and the parameter space of these models is already strongly constrained by current limits. Consequently, even null results from the next generation of EDM experiments would be interesting, for these would give hints as to the energy scale at which new physics could be.

Such null results could also damage beyond repair certain theoretical explanations for generating the baryon asymmetry of the universe through the physics of the electroweak phase transition. Two of the famous Sakharov conditions for the generation of the baryon asymmetry (namely, B violation and a departure from thermal equilibrium) are already present in the SM, in principle. For a Higgs mass of some 125 GeV, however, SU(2) lattice gauge–Higgs theory simulations, as in [1392], e.g., reveal that the electroweak phase transition is not of first order. The lack of a sufficiently robust first-order phase transition can also be problematic in BSM models. Nevertheless, new mechanisms, or sources, of CP (or T) and C violation in the quark sector could make baryon production much more effective. Existing EDM constraints curtail possible electroweak baryogenesis scenarios in the MSSM severely [1393–1395], and an improvement in the experimental bound on $d_n$ by a factor of $\sim$100 could rule out the MSSM as a model of electroweak baryogenesis [1350, 1396, 1397]. This outcome would thereby favor supersymmetric models beyond the MSSM, such as in [1398–1408], or possibly mechanisms based on the two-Higgs doublet model (2HDM) [1409], or mechanisms which are not tied to the weak scale, such as leptogenesis, or dark-matter mediated scenarios. Consequently, people have come to recognize that a measurement of an EDM in any system, regardless of its complexity, is of fundamental interest. Since there are many different possibilities for generating an EDM at a microscopic level, many experiments are likely to be needed to localize the fundamental source of any EDM once observed. New ideas for EDM measurements abound and have come from scientific communities in atomic, molecular, nuclear, particle, and condensed-matter physics.

Compact overviews of this field can be found in [1387, 1410], whereas a recent theoretical review can be found in [1238]. The most stringent limits on particle EDMs come from atomic physics measurements in $^{199}$Hg [1390]. However, it is known that, in the pointlike, non-relativistic limit, the electron cloud of an atom shields any EDM which might be present in the nucleus—making the atomic EDM zero even if the nuclear EDM were not. This “no-go” result is known as Schiff’s theorem [1411]. As a consequence, the fantastic upper bound on the EDM in this atom places a much weaker constraint on the EDM of its nucleons.

Atomic and molecular physicists have long sought systems in which the EDM could be amplified rather than shielded by electron effects; such an amplification can indeed occur in certain polar molecules [1388, 1412]. Gross enhancements also exist in certain heavy atoms whose relativistic motion evades Schiff’s theorem, yielding an EDM which scales as $Z^3\alpha ^2$ [1413, 1414]. More recently it has been recognized that atoms whose nuclei possess octupole deformations [1415] can have particularly enhanced atomic EDMs, by orders of magnitude over $^{199}$Hg [1416], in part through the resulting mixing of certain nearly-degenerate atomic energy levels. Even such enhancements do not defeat Schiff’s theorem completely, though they can come close. To be suitable for an EDM experiment, it is also necessary to be able to polarize sufficiently large ensembles of nuclei in order to perform the delicate NMR frequency-difference measurements typically needed to detect EDMs. Such needs, in concert with the desired enhancements, lead one to consider certain heavy radioactive nuclei such as radon and radium. Recently, the first direct evidence of octupole deformation in $^{224}$Ra has been established through measurements of Coulomb excitation of 2.85 MeV/amu rare-isotope beams at REX-ISOLDE (CERN) [1417], strengthening the confidence in the size of the Schiff moment in like systems, whose computation is dominated by many-body calculations in nuclear and atomic physics. Generally, in the presence of rigid octupole deformation, as observed in $^{224}$Ra, the computation of the Schiff moment is expected to be more robust [1238]. This underscores the discovery potential of an EDM measurement in such systems. Progress towards an EDM measurement in $^{225}$Ra, e.g., is ongoing [1418], and the sensitivity of an eventual EDM limit could be greatly increased through the enhanced isotope production capability of a megawatt-class 1 GeV proton linac [1387].

EDM searches on simpler objects such as the neutron, proton, or deuteron, e.g., are of course more directly interpretable in terms of the fundamental sources of CP violation at the quark level. The theoretical interpretation of these systems in chiral effective theory has been under intense development [1419–1423]. Many experiments to search for a neutron EDM are in progress [1424–1427], of which the nEDM-SNS experiment under development at ORNL is the most ambitious [1424]. Its ultimate goal is to improve the sensitivity by more than two orders of magnitude beyond the present 90 % CL bound of some $3\times 10^{-26}\, e\hbox {-}{\mathrm {cm}}$ [1232]. This limit already constrains, e.g., the CP-violating phases in minimal supersymmetric models to assume unnaturally small values, or to make the masses of the supersymmetric partner particles larger than previously anticipated, or to make the spectrum of partner particles possess unexpected degeneracies. These experiments are broadly similar in experimental strategy to atomic physics approaches.

Over the last few years a qualitatively new approach to the measurement of particle EDMs using charged particles in storage rings [1428], exploiting the large electric fields present in such environments, has come under active development. Such an experiment would have the advantage of enlarging the spectrum of available species to include charged particles, and the ability to allow a coherent effect to accumulate over many revolutions around a ring. A variety of operators can generate an EDM, so that stringent EDM measurements on the proton, neutron, and other light nuclei are complementary and can help unravel the underlying CP-violating mechanism if a signal is seen. The theoretical insights to be gained have been studied carefully [1421, 1422, 1429]. An experimental difficulty of this approach is that one loses the clean electric-field flip used in previous experiments on electrically neutral objects to reduce systematic errors. Instead one must typically measure a rotation of the plane of polarization of a transversely polarized particle in the ring and to develop other methods to deal with systematic errors, as discussed in [1387]. Measurements in existing storage rings to quantify these instrumental issues are in progress.

Finally one can consider constraints on the EDMs of leptons. The muon EDM can be limited in part as a byproduct of the muon $g-2$ measurements [1389], and the heavier mass of the muon amplifies its sensitivity to certain new-physics possibilities. Nevertheless, at anticipated levels of sensitivity, such experiments constrain CP-violating sources which do not simply scale with the mass of the muon. Such flavor-blind CP-violating contributions to the muon EDM are already severely constrained by electron EDM limits; rather, direct limits probe the possibility of lepton-flavor violation here as well [1386, 1430, 1431]. The electron EDM possesses stringent limits from atomic and molecular physics measurements, and in addition there are many promising approaches under development, which could achieve even higher levels of sensitivity. These range from solid-state systems at low temperature [1432, 1433] to new experiments with cold molecules [1434]. Indeed, the ACME collaboration, using ThO, has just announced a limit on $d_e$ an order of magnitude smaller than any ever achieved before [1435]. These constraints are important in themselves and are also needed to interpret the source of an EDM if observed in an atomic physics experiment.

#### EFTs for EDMs: the neutron case

We now consider how sources of CP violation beyond the SM can generate a permanent EDM at low energies. Noting [1348], we organize the expected contributions in terms of the mass dimension of the possible CP-violating operators, in quark and gluon degrees of freedom, appearing in an effective field theory with a cutoff of ${\sim }1$ GeV:

5.11 $$\begin{aligned} \mathcal{L}&= \frac{\alpha _\mathrm{s} \bar{\theta }}{8\pi } \epsilon ^{\alpha \beta \mu \nu }F_{\alpha \beta }^a F_{\mu \nu }^a \nonumber \\&- \frac{i}{2} \sum _{i\in u,d,s}\!\left( d_i \bar{\psi }_i F_{\mu \nu }\sigma ^{\mu \nu } \gamma _5 \psi _i + {\tilde{d}}_i \bar{\psi }_i F_{\mu \nu }^a T^a\sigma ^{\mu \nu }\gamma _5 \psi _i \right) \nonumber \\&+ \frac{w}{3} f^{abc} \epsilon ^{\nu \beta \rho \delta } (F^a)_{\mu \nu } (F^b)_{\rho \delta } (F^\mathrm{c})_\beta ^{\mu } \nonumber \\&+ \sum _{i,j} C_{ij} (\bar{\psi }_i \psi _i)(\bar{\psi }_j i\gamma _5\psi _j) + \cdots \end{aligned}$$

with $i,j\in u,d,s$ unless otherwise noted—all heavier degrees of freedom have been integrated out. The leading term is the dimension-four strong CP term already discussed, proportional to the parameter $\bar{\theta }$, though it can also be induced by higher-dimension operators even in the presence of axion dynamics [1348, 1436] so that we retain it explicitly. The terms in the second line of (5.11) appear to be of dimension five, but their chirality-changing nature implies that a Higgs insertion, of form $H/v$, say, is needed to make the operator invariant under SU(2)$_{L}\times$U(1) symmetry. (See (3.1) in [1437] for an explicit expression.) Therefore these operators, which determine the fermion EDMs $d_i$ and quark chromo-EDMs (CEDM) $\tilde{d}_i$, are suppressed by an additional factor containing a large mass scale and should be regarded as dimension-six operators in numerical effect. The remaining terms in (5.11) are the dimension-six Weinberg three-gluon operator from (5.9) with coefficient $w$, and CP-violating four-fermion operators, characterized by coefficients $C_{ij}$. Turning to [1353], we note that after electroweak symmetry breaking there are also chirality-changing four-fermion operators which, analogously, are of dimension eight numerically once SU(2)$_{L}\times$U(1) symmetry is imposed. Various extensions of the SM can generate the low-energy constants which appear, so that, in turn, EDM limits thereby constrain the new sources of CP violation which appear in such models. In connecting the Wilson coefficients of these operators and hence models of new physics to the low-energy constants of a chiral effective theory in meson and nucleon degrees of freedom requires the evaluation of non-perturbative hadron matrix elements. Parametrically, we have [1348]

5.12 $$\begin{aligned} d_n&= d_n({\bar{\theta }},d_i, {\tilde{d}}_i,w,C_{ij}) \nonumber \\ {\bar{g}}_{\pi NN}^{(i)} ,&= {\bar{g}}_{\pi NN}^{(i)}({\bar{\theta }},d_i, {\tilde{d}}_i,w,C_{ij}). \end{aligned}$$

Several computational aspects must be considered in connecting a model of new physics at the TeV scale to the low-energy constants of (5.11). After matching to an effective theory in SM degrees of freedom, there are QCD evolution and operator-mixing effects, as well as flavor thresholds, involved in realizing the Wilson coefficients at a scale of ${\sim }$1 GeV. Beyond this, the hadronic matrix elements must be computed. A detailed review of all these issues can be found in [1238]. Typically QCD sum rule methods, or a SU(6) quark model, have been employed in the computation of the matrix elements [1348]; for the neutron, we refer to [1438] for a comparative review of different methods. Lattice gauge theory can also be used to compute the needed proton and neutron matrix elements, and the current status and prospects for lattice-QCD calculations are presented in the next section. We note in passing that $d_n$ and $d_p$ have also been analyzed in chiral perturbation theory [1439–1441], as well as in light-cone QCD [1442]. We refer to Sect. 3.4.7 for a general discussion of chiral perturbation theory in the baryon sector.

We turn now to the evaluation of the requisite hadron matrix elements with lattice QCD.

#### Lattice-QCD matrix elements

**Fig. 37:**
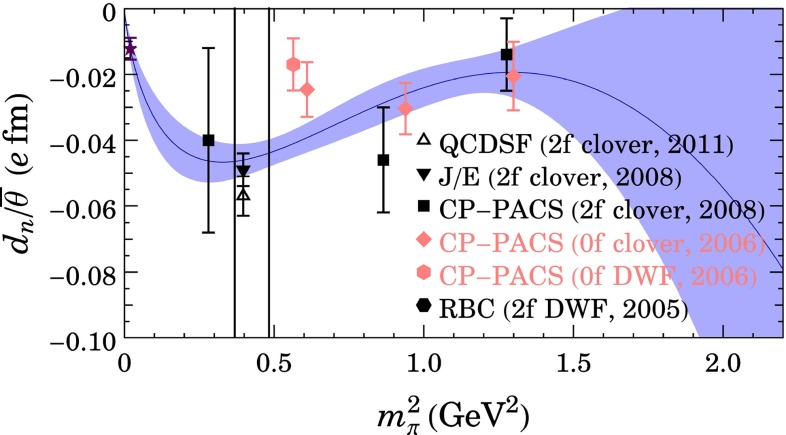
Summary of the latest dynamical calculations of the neutron EDM [1443–1448] $d_n$ as a function of $m_\pi ^2$ from a nonzero $\bar{\theta }$ term in QCD. The band is a global extrapolation at 68 % CL combining all the lattice points (except for [1448]) each weighted by its *error bar*. The *leftmost star* indicates the value at the physical pion mass. Figure taken from [1449]

To generate a nonzero neutron EDM, one needs interactions that violate CP symmetry, and the CP-odd $\bar{\theta }$-term in the SM is one possible example. The most common type of lattice-QCD EDM calculation is that of the neutron matrix element of the operator associated with the leading $\bar{\theta }$ term. A recent combined analysis gives $O(30~\%)$ in the statistical error alone, noting Fig. 37 for a summary, so that the precision of lattice-QCD calculations needs to be greatly improved. All-mode averaging (AMA) has been proposed to improve the current statistics even at near-physical pion mass [683].

There are currently three main approaches to computing these matrix elements using lattice QCD. One is a direct computation, studying the electromagnetic form factor $F_3$ under the QCD Lagrangian including the CP-odd $\theta$ term (as adopted by RBC, J/E, and CP-PACS (2005) [1443, 1445–1448])5.13where $J^\mathrm{EM}_{\mu }$ is the electromagnetic current, $\bar{u}$ and $u$ are appropriate spinors for the neutron, and $q$ is the transferred momentum. This requires an extrapolation of the form factors to $q^2=0$, which can introduce systematic error and exacerbate the statistical error. Another method is introducing an external static and uniform electric field and looking at the energy difference induced between the two spin states of the nucleon at zero momentum (by CP-PACS [1445]), one can infer $d_n$. Or, finally, one can compute the product of the anomalous magnetic moment of neutron $\kappa _\mathrm{N}$ and $\tan (2\alpha )$ (by QCDSF [1448]), where $\alpha$ is the $\gamma _5$ rotation of the nucleon spin induced by the CP-odd source. A summary of $d_n$ calculations from dynamical lattice QCD is shown in Fig. 37, where the results are given as a function of the pion mass used in the calculation. Combining all data and extrapolating to the physical pion mass yields $d_n^\mathrm{lat}=(0.015 \pm 0.005) \bar{\theta }$  $e\hbox {-}{\mathrm {fm}}$ [1449], which is the starred point in the figure. Further and more precise calculations from various groups are currently in progress, using improved techniques to reduce the statistical error, such as the aforementioned AMA [683].

It should also be possible to compute the nucleon matrix elements of higher-dimension operators, such as the quark electric dipole moment (qEDM) and the chromoelectric dipole moment (CEDM). This will require us to extend lattice-QCD calculations to such cases [1437], and we now discuss the prospects.

**Fig. 38:**
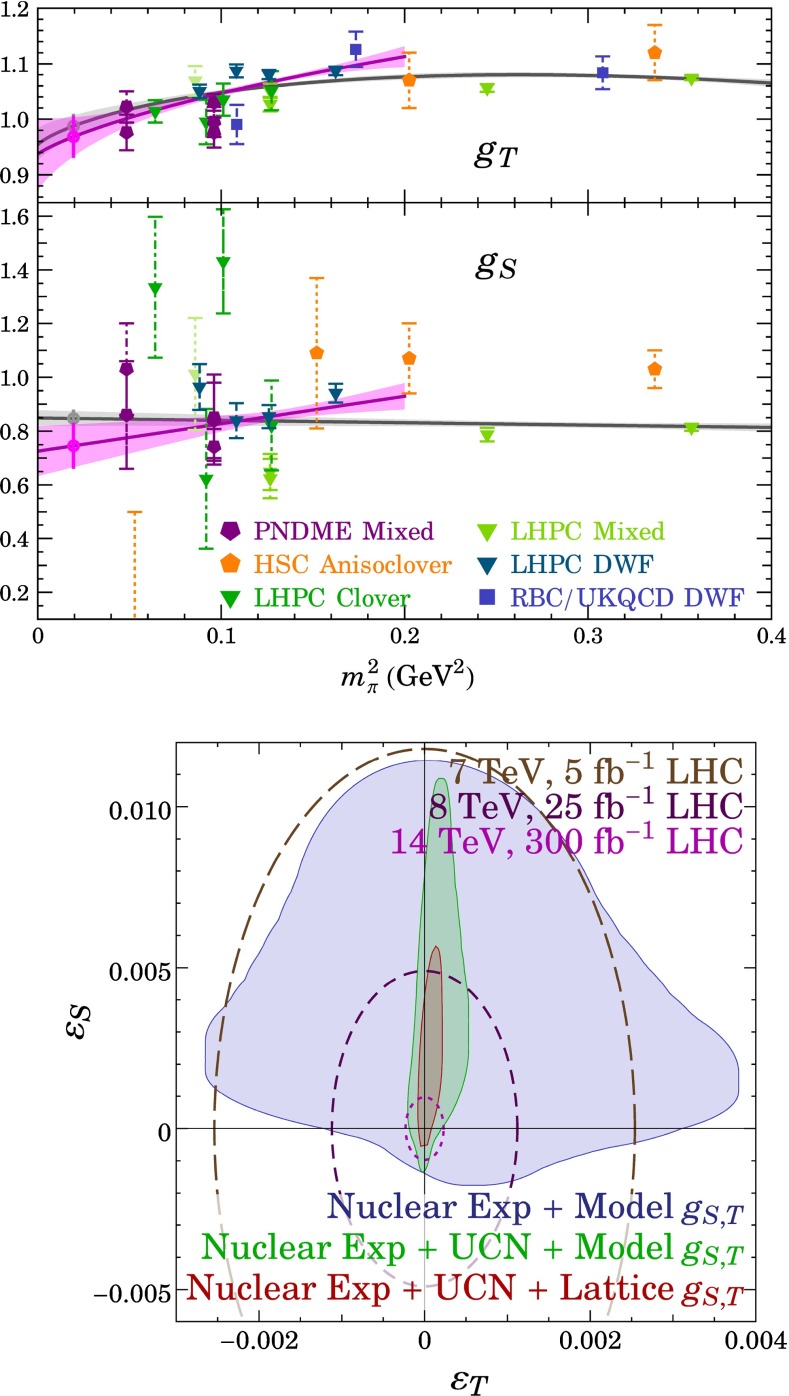
Figures adapted from [203]. (*Upper figure*) Global analysis of all $N_\mathrm{f}=2+1(+1)$ lattice calculations of $g_\mathrm{T}$ (*upper*) and $g_\mathrm{S}$ (*lower*) [206, 234, 251, 261, 1458] with $m_\pi L >4$ and $m_\pi T > 7$ cuts to avoid systematics due to small spatial or temporal extent. The *leftmost points* are the extrapolated values at the physical pion mass. The two bands show extrapolations with different upper pion-mass cuts: $m_\pi ^2 <0.4$ and $m_\pi ^2 <0.2$. The $m_\pi L<4$ data points are marked faded within each calculation; the lattice spacings for each point are denoted by a *solid line* for $a\le 0.06$ fm, *dashed* $0.06 < a \le 0.09$ fm, *dot–dashed* $0.09 < a \le 0.12$ fm, and *dotted* $a > 0.12$ fm. (*Lower figure*) The allowed $\epsilon _{S}$–$\epsilon _{T}$ parameter region using different experimental and theoretical inputs. The outermost (*green*), middle (*purple*), and innermost (*magenta*) *dashed lines* are the constraints from the first LHC run [1463], along with near-term expectations, running to a scale of 2 GeV to compare with the low-energy experiments. The inputs for the low-energy experiments assume that limits (at 68 % CL) of $|b|< 10^{-3}$ and $|B_\mathrm{BSM} -b| < 10^{-3}$ from neutron $\beta$ decay and a limit of $g_\mathrm{T} \epsilon _\mathrm{T} < 2\times 10^{-4}$ from $^6$He $\beta$ decay [1464], which is a purely Gamow–Teller transition. These low-energy experiments probe $S,T$ interactions through possible interference terms and yield constraints on $\mathrm{Re}(\epsilon _\mathrm{T})$ and $\mathrm{Re}(\epsilon _\mathrm{S})$ only

*a. Quark Electric Dipole Moment* In this case, the neutron EDM is induced by nonzero quark electric dipole moments, which are related to the following matrix elements of the hadronic part of the first of the effectively dimension-six operators in (5.11):5.14The nucleon matrix elements can be accessed through direct lattice-QCD calculations with isoscalar and isovector tensor charges. There are several existing lattice-QCD calculations of the isovector tensor charge; see Fig. 38.

*b. Chromoelectric Dipole Moment* In this case a direct calculation of the chromoelectric dipole moments would be more challenging on the lattice, since it requires the calculation of a four-point Green function. Only a few such calculations have previously been attempted. One way to avoid this problem would be to apply the Feynman–Hellmann theorem by introducing an external electric field $E$ to extract the matrix elements:5.15where $A_{\mu }(E)$ refers to the corresponding vector potential and $G^{\nu \kappa }$ is shorthand for $(F^a)^{\nu \kappa } T^a$. Similar techniques have been widely implemented in lattice QCD to determine the strangeness contribution to the nucleon mass; one only needs to combine the idea with a nucleon matrix element calculation. Although as of the time of this review, no lattice calculation of the chromoelectric dipole moment has been attempted, we are optimistic that it will be explored within the next few years.

Currently, lattice-QCD calculations on $d_n$ due to the leading $\theta$ term have statistical errors at the level of 30 % after a chiral extrapolation combining all existing dynamical data. More updates and precise calculations from various groups are currently in progress, including improved numerical techniques that will significantly reduce the errors. Within the next 5 years, lattice QCD should be able to make predictions of better than 10 % precision, and one can hope that percent-level computations will be available on a ten-year timescale.

Outside the leading-order $\theta$ term, there are plans for calculating the dimension-six operator matrix elements by the PNDME (http://www.phys.washington.edu/users/hwlin/pndme/index.xhtml) collaboration. The matrix elements relevant to the quark electric dipole moments are rather straightforward, involving isovector and isoscalar nucleon tensor matrix elements. The latter one requires disconnected diagrams with extra explicit quark loops. They will require techniques similar to those already used to determine the strangeness contribution to the nucleon mass and the strange spin contribution to nucleon. However, the chromoelectric dipole moment is more difficult still, since it requires a four-point Green function. One alternative method we have considered would be to take a numerical derivative with the magnitude of the external electric field [1437]. We should see some preliminary results soon.

### Probing non-$(V-A)$ interactions in beta decay

The measurement of non-SM contributions to precision neutron (nuclear) beta-decay measurements would hint to the existence of BSM particles at the TeV scale; if new particles exist, their fundamental high-scale interactions would appear at low energy in the neutron beta-decay Hamiltonian as new terms, where we recall (5.10) and the opening discussion of Sect. 5.3. In this case the new terms are most readily revealed by their symmetry; they can violate the so-called $V-A$ law of the weak interactions. Specifically, in dimension six, the effective Hamiltonian takes the form

5.16 $$\begin{aligned} H_\mathrm{eff} = G_\mathrm{F} \Bigg ( J_{V-A}^\mathrm{lept} \times J_{V-A}^\mathrm{quark} + \sum _i \epsilon _i \hat{O}_i^\mathrm{lept} \times \hat{O}_i^\mathrm{quark} \Bigg ), \nonumber \\ \end{aligned}$$

where $G_\mathrm{F}$ is the Fermi constant, $J_{V-A}$ is the left-handed current of the indicated particle, and the sum includes operators of non-$(V-A)$ form which represent physics BSM. As we have noted, the new operators will enter with coefficients controlled by the mass scale of new physics; this is similar to how the dimensionful Fermi constant gave hints to the masses of the $W$ and $Z$ bosons of the electroweak theory prior to their discovery. Matching this to an effective theory at the nucleon level, the ten terms of the effective Hamiltonian are independent, linear combinations of the coefficients of the Lee–Yang Hamiltonian [1371, 1372]. Since scalar and tensor structures (controlled by $\epsilon _\mathrm{S}$ and $\epsilon _\mathrm{T}$ in $\beta$ decay) do not appear in the SM Lagrangian, signals in these channels at current levels of sensitivity would be clear signs of BSM physics. In neutron decay, the new operators of (5.16) yield, in particular, the following low-energy coupling constants $g_\mathrm{T}$ and $g_\mathrm{S}$ (here, multiplied by proton and neutron spin wave functions):

5.17 $$\begin{aligned} g_\mathrm{T} \bar{u}_n \sigma _{\mu \nu } u_p&= \langle n | \overline{u}\sigma _{\mu \nu } d | p \rangle \end{aligned}$$

5.18 $$\begin{aligned} g_\mathrm{S} \bar{u}_n u_p&= \langle n | \overline{u} d | p \rangle . \end{aligned}$$

Lattice QCD is a perfect theoretical tool to determine these constants precisely.

The search for BSM physics proceeds experimentally by either measuring the Fierz interference term $b$ (i.e., $b m_e/E_e$) or the neutrino asymmetry parameter $B$ (i.e., $B(E_e){\mathbf {S}}_n \cdot {\mathbf {p}}_\nu$) of the neutron differential decay rate [1240]. The Fierz term can either be measured directly or indirectly, the latter through either its impact on the electron-neutrino correlation $a {\mathbf {p}}_e\cdot {\mathbf {p}}_\nu$ or on the electron-momentum correlation with neutron-spin, $A{\mathbf {S}}_n\cdot {\mathbf {p}}_e$. Here, ${\mathbf {S}}_n$ and ${\mathbf {p}}_{\ell }$ denote the neutron spin and a lepton momentum (${\ell } \in (e,\nu )$), respectively. We note, neglecting Coulomb corrections, $b=(2/(1+3\lambda ^2))(g_\mathrm{S} \mathrm{Re(\epsilon _\mathrm{S})} - 12 \lambda g_\mathrm{T} \mathrm{Re}(\epsilon _\mathrm{T}))$ [1450], where $\lambda =g_A/g_V\approx 1.27$. Assessing $b$ through $a$ or $A$ employs an asymmetry measurement, reducing the impact of possible systematic errors. The Fierz term is nonzero only if scalar or tensor currents appear, whereas the latter contribute to the magnitude of $B$.

There are several upcoming and planned experiments worldwide to measure the correlation coefficients in neutron decay, with plans to probe $b$ and $B_\mathrm{BSM}$ up to the ${\sim } 10^{-3}$ level or better, and they include PERC [1451] at the FRM-II, PERKEOIII at the ILL [1452], UCNB [1453] and UCNb [1454] at LANL, Nab at ORNL [1455, 1456], and ACORN [1457] at NIST—indeed the PERC experiment [1451] has proposed attaining $10^{-4}$ precision. Models of QCD give rather loose bounds on $g_\mathrm{S}$ and $g_\mathrm{T}$; for example, $g_\mathrm{S}$ is estimated to range between 0.25 and 1 [1458]. Consequently, lattice-QCD calculations of these quantities need not be terribly precise to have a dramatic positive impact. Indeed, determining $g_{S,T}$ to 10–20 % (after summing all systematic uncertainties) [1458] is a useful and feasible goal. The obvious improvement in the ability to limit the coefficients of the underlying non-$(V-A)$ interactions speaks to its importance.

The PNDME collaboration reported the first lattice-QCD results for both $g_\mathrm{S}$ and $g_\mathrm{T}$ [1458] and gave the first estimate of the allowed region of $\epsilon _{S}$–$\epsilon _{T}$ parameter-space when combined with the expected experimental precision; we will return to this point in a moment. The latest review, from [203], contains a summary of these charges; to avoid the unknown systematics coming from finite-size artifacts, data with $M_\pi L \le 4$ and $M_\pi T \le 8$ are omitted, as shown on the lower part of Fig. 38. This figure includes updated calculations of $g_{S,T}$ after [1458] from the PNDME and LHP collaborations. Reference [203] uses the chiral formulation given in [1459] and [1460] for the tensor and scalar charges, respectively, to extrapolate to the physical pion mass. We see that the PNDME points greatly constrain the uncertainty due to chiral extrapolation in both cases and obtain $g_\mathrm{T}^\mathrm{lat}= 0.978 \pm 0.035$ and $g_\mathrm{S}^\mathrm{lat}= 0.796 \pm 0.079$, where only statistical errors have been reported.

More recently, the PNDME collaboration has computed the axial, scalar, and tensor charges on two HISQ ensembles with 2+1+1 dynamical flavors at a lattice spacing of 0.12 fm and with light-quark masses corresponding to pions with masses of 310 and 220 MeV [248]. These ensembles have been generated by the MILC Collaboration [825]. Including systematic errors, the continuum and chiral extrapolation yields the estimates $g_S = 0.72 \pm 0.32$ and $g_T = 1.047 \pm 0.061$. In comparison, the recent LHPC results are $g_S =1.08 \pm 0.28 \pm 0.16$ and $g_T = 1.038 \pm 0.011 \pm 0.012$, with $M_{\pi } \approx 150$ MeV at a single lattice spacing of $a \approx 0.116$ fm [1460]. A different, promising path to $g_S$ has been realized in [1461], exploiting lattice-QCD calculations of the neutron–proton mass difference in pure QCD to yield a value of $g_S$.

As previously mentioned, the tensor and scalar charges can be combined with experimental data to determine the allowed region of parameter space for scalar and tensor BSM couplings. Using the $g_{S,T}$ from the model estimations and combining with the existing nuclear experimental data,^12^ we get the constraints shown as the outermost band of the lower part of Fig. 38. Combining anticipated (in the shorter term) results from $\beta$-decay and existing measurements, and again, using the model inputs of $g_{S,T}$, we see the uncertainties in $\epsilon _{S,T}$ are significantly improved. (A limit on $g_\mathrm{T} \epsilon _\mathrm{T}$ also comes from radiative pion decay, but it can be evaded by cancellation and has been omitted [1240, 1462].) Finally, using our present lattice-QCD values of the scalar and tensor charges, combined with the anticipated precision of the experimental bounds on the deviation of low-energy decay parameters from their SM values, we find the constraints on $\epsilon _{S,T}$ are further improved, shown as the innermost region. These upper bounds on the effective couplings $\epsilon _{S,T}$ would correspond to lower bounds for the scales $\Lambda _{S,T}$ at 5.6 and 10 TeV, respectively, determined using naive dimensional analysis ($\epsilon _i \sim (v/\Lambda _i)^2$ with $v \sim 174~\mathrm{GeV}$), for new physics in these channels.

**Fig. 39:**
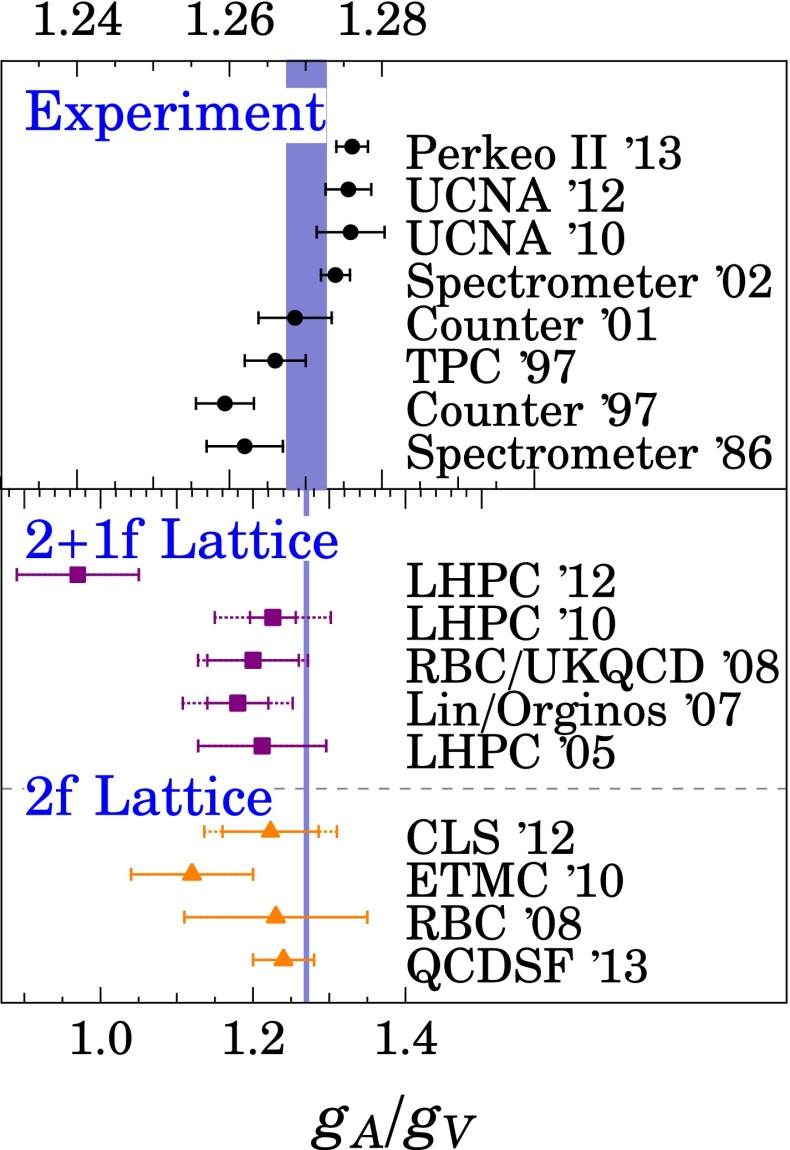
Compilation of $g_A$ determined from experiment (*top*) and lattice QCD (*bottom*) adapted from Ref. [1437]. The *lower panel* shows $g_A$ values after extrapolating to the physical pion mass collected from dynamical 2+1-flavor and 2-flavor lattice calculations using $O(a)$-improved fermions [209, 236, 240, 242, 247, 249–251, 255, 256, 259–261, 1460, 1469–1472]. A small discrepancy persists: while calculations continue to tend towards values around 1.22 with a sizeable error, the experimental values are converging towards $1.275 \pm 0.005$. A significant lattice effort will be necessary to reduce the systematics and achieve total error at the percent level

There is a complication, however, that should be noted. The analysis of neutron $\beta$ decay requires a value of the neutron axial vector coupling $g_A$ as well (similar considerations operate for Gamow–Teller nuclear transitions); presently, this important quantity is taken from experiment because theory cannot determine it well enough, as illustrated in Fig. 39. This topic is also addressed in Sect. 3.2.5a; here we revisit possible resolutions. A crucial direction for lattice QCD is to reexamine the systematics in the nucleon matrix elements, a task that was somewhat neglected in the past when we struggled to get enough computing power to address merely statistical errors. Resources have improved, and many groups have investigated the excited-state contamination, and this seems to be under control in recent years. However, the results remain inconsistent with experiment, and more extensive studies of finite-volume corrections with high statistics will be carried out in the future. In addition, the uncertainty associated with extrapolating to a physical pion mass should be greatly improved within the next year or two. Overall, we believe $g_A$ will be calculated to the percent-level or better (systematically and statistically) in the next few years. It is worth noting that a blind analysis should be easy to carry out for $g_A$ since it is an overall constant in the lattice three-point correlators. Nevertheless, it is currently the case that poorly understood systematics can affect the lattice-QCD computations of the nucleon matrix elements, and those of $g_A$ serve as an explicit example. However, those uncertainties are not so large that they undermine the usefulness of the $g_\mathrm{S}$ and $g_\mathrm{T}$ results. As we have shown, the lattice computations of these quantities need not be very precise to be useful.

There are also other $\beta$-decay nucleon matrix elements induced by strong-interaction effects which enter as recoil corrections at $\mathcal{O}(E/M)$, where $E$ is the electron energy scale and $M$ is the nucleon mass. The weak magnetic coupling $f_2$ can be determined using the conserved-vector-current (CVC) hypothesis (though a fact in the SM) and the isovector nucleon magnetic moment, though this prediction, as well as that of the other matrix elements to this order, is modified by isospin-breaking effects. This makes it useful to include errors in the assessment of such matrix elements, when optimizing the parameters to be determined from experiment. Such a scheme has been developed, after that in [1369], in [1465], and the impact of such theory errors on the ability to resolve non-$(V-A)$ interactions has been studied, suggesting that it is important to study the induced tensor term $g_2$ and other recoil-order matrix elements using lattice QCD as well. The study of [1465] shows that it is also crucial to measure the neutron lifetime extremely well, ideally to $\mathcal{O}(0.1\,\mathrm{sec})$ precision, in order to falsify the $V-A$ law and establish the existence of physics BSM in these processes. We refer the reader to Sect. 5.5.1 for a perspective on the neutron lifetime and its measurement.

Second-class currents gain in importance in neutron decay precisely because it is a mixed transition—and because BSM effects are already known to be so small. No direct lattice-QCD study of these isospin-breaking couplings has yet been done, but a few previous works have tried to estimate their size in hyperon decay [1466–1468]. Perhaps particularly interesting is the analysis of the process $\Xi ^0 \rightarrow \Sigma ^{+} {\ell }\bar{\nu }$, in which the second-class current terms emerge as SU(3)$_\mathrm{f}$ breaking effects. In this case, [1467] $|f_3(0)/f_1(0)| = 0.14 \pm 0.09$ and $|g_2(0)/f_1(0)| = 0.68 \pm 0.18$; this exploratory calculation is made in the quenched approximation with a relatively heavy pion mass of 539–656 MeV. Nevertheless, this decay is a strict analog of the neutron decay process, with the $d$ valence quark replaced by $s$, so that one can *estimate* the size of $g_2/g_A$ in neutron decay by scaling the earlier results by a factor of $m_d/m_\mathrm{s} \sim 1/20$ [1465]. Ultimately, one can foresee results with reduced uncertainties from direct calculations on physical pion mass ensembles, using the variation of the up and down quark masses to resolve the second-class contributions in neutron decay.

High-energy colliders can constrain $\epsilon _\mathrm{S}$ and $\epsilon _\mathrm{T}$ in the manner shown in Fig. 38. Unfortunately, as shown in [203, 1458], the CDF and D0 results do not provide useful constraints in this context. The limits shown follow from estimating the $\epsilon _{S,T}$ constraints from LHC current bounds and near-term expectations through an effective Lagrangian

5.19 $$\begin{aligned} \mathcal{L} = -\frac{\eta _\mathrm{S}}{\Lambda _\mathrm{S}^2}V_{ud}(\overline{u}d)(\overline{e}P_L\nu _e) -\frac{\eta _\mathrm{T}}{\Lambda _\mathrm{T}^2}V_{ud}(\overline{u}\sigma ^{\mu \nu }d) (\overline{e}\sigma _{\mu \nu }P_L\nu _e), \end{aligned}$$

where $\eta _{S,T}=\pm 1$ to account for the possible sign of the couplings at low-energy. The high-energy bounds are scaled down to a scale of 2 GeV to compare with low-energy predictions. By looking at events with high transverse mass from the LHC in the $e\nu +X$ channel and comparing with the SM $W$ background, the authors of [203, 1458] estimated 90 %-C.L. constraints on $\epsilon _{S,T}$ based on existing data [1463], $\sqrt{s}=7$ TeV $L=10 \text{ fb }^{-1}$ (the outermost (green) line) and the anticipated (null result) data sets at $\sqrt{s}=8$ TeV $L=25 \text{ fb }^{-1}$ (the middle (purple) line) and $\sqrt{s}=14$ TeV $L=300 \text{ fb }^{-1}$ (the innermost (magenta) line) of the lower panel in Fig. 38. The low-energy experiments can potentially yield much sharper constraints.

There is plenty of room for further improvements of lattice-QCD calculations of $g_{S,T}$. Currently, there are fewer direct lattice calculations of $g_\mathrm{T}$ and $g_\mathrm{S}$, and the errors are roughly 10 % and 30 %, respectively. Ongoing calculations are improving control over the systematics due to chiral extrapolation and finite-volume effects. In addition, we expect more collaborations will compute these quantities, and near-future work will substantially reduce the errors. In particular, there is presently no chiral perturbation theory formula for the extrapolation to a physical pion mass, and operator matching is done either at tree- or one-loop level. Work is under way to reduce these errors, and we expect results with 5 % errors (including all systematics) on a five-year timescale.

#### The role of the neutron lifetime

The neutron lifetime value provides important input to test weak-interaction theory in the charged-current sector [1473]. It is also important for Big-Bang nucleosynthesis (BBN), which is becoming more and more important for constraining many BSM physics scenarios which produce new contributions to the relativistic particle energy density [1243]. BBN predicts the primordial abundances of the light elements (H, He, D, Li) in terms of the baryon-to-photon ratio $\eta$, together with nuclear physics input that includes 11 key nuclear reaction cross sections, along with the neutron lifetime [1474]. As primordial neutrons are protected against $\beta$-decay by fusing with protons into deuterons and then into $^4$He, a shorter neutron lifetime results in a smaller $^4$He abundance ($Y_{p}$). The dependence of the helium abundance on changes in the neutron lifetime, the “effective” number of light neutrinos $N_\mathrm{eff}$, and the baryon-to-photon ratio are: $\delta Y_{p}/Y_{p}=+0.72 \delta \tau _{n}/\tau _{n}$, $\delta Y_{p}/Y_{p}=+0.17 \delta N_\mathrm{eff}/N_\mathrm{eff}$, and $\delta Y_{p}/Y_{p}=+0.039 \delta \eta / \eta$  [1475, 1476]. With the precise determination of $\eta$ from WMAP [1477] and now PLANCK [1478], the 0.2–0.3 % error on the BBN prediction for Y$_p$ is now dominated by the uncertainty in the neutron lifetime. At the same time astrophysical measurements of the helium abundance ($Y_{p}=0.252 \pm 0.003$ [1476, 1479]) from direct observations of the H and He emission lines from low-metallicity regions are poised for significant improvement. Astronomers are now in a position to re-observe many of the lowest abundance objects used for nebular $^{4}$He abundance determinations over the next 3–5 years and will continue to find additional ultralow abundance objects [1480]. Sharpening this test of BBN will constrain many aspects of nonstandard physics scenarios.

Measurements of the neutron lifetime had been thought to be approaching the 0.1 % level of precision (corresponding to a $\sim$ 1 s uncertainty) by 2005, with the Particle Data Group [1481] reporting $885.70\pm 0.85$ s. However, several neutron lifetime results since 2005 using ultracold neutron measurements in traps [1482–1485] reported significantly different results from the earlier PDG average: the latest PDG value ($880.1 \pm 1.1$ s) [1] includes all these measurements, with the uncertainty scaled up by a factor of $2.7$. The cause of this many-sigma shift has not yet been resolved. The large discrepancies between the latest lifetime measurements using ultracold neutrons in material bottles make it clear that systematic errors in at least some previous measurements have been seriously underestimated, and precision measurements using alternative techniques are badly needed [1486]. The latest update [1487] from a Penning trap neutron lifetime experiment in a cold neutron beam [1488, 1489] gives $\tau _{n}= 887.7 \pm 1.2\,(\mathrm{stat.})\,\pm 1.9\,(\mathrm{sys})\,\mathrm{s}$. In addition to continued measurements using the Penning trap technique, neutron lifetime measurements with ultracold neutrons now concentrate on trapping the neutrons using magnetic field gradients in an attempt to avoid what people suspect to be uncontrolled systematic errors from surface effects in material traps. A recent experiment at Los Alamos using a magneto-gravitational trap that employs an asymmetric Halbach permanent magnet array [1490] has observed encouraging results [1491].

### Broader applications of QCD

Nucleon matrix elements and lattice-QCD methods are key to a broad sweep of low-energy observables which probe how precisely we understand the nature of things. We now consider a range of examples, to illustrate the breadth of the possibilities.

#### Determination of the proton radius

The charge radius of the proton $r_p$ has not yet been precisely calculated in lattice QCD because the computation of disconnected diagrams with explicit quark loops is required. (In the case of the isovector charge radius ($r_p - r_n$) the disconnected diagrams cancel, so that this quantity could be more precisely calculated than $r_p$.) Rather, it is currently determined from the theoretical analysis of experimental results. There has been great interest in $r_p$ because the determination of this quantity from the study of the Lamb shift in muonic hydrogen [282, 285], yielding [285]

5.20 $$\begin{aligned} r_p^{(\mu H)} = 0.84087 \pm 0.00039 \,\mathrm{fm} , \end{aligned}$$

is some $7\sigma$ different from the value in the CODATA-2010 compilation [284], determined from measurements of hydrogen spectroscopy ($r_p^{(e H)}$) and electron–proton ($r_p^{(e p)}$) scattering. The incompatibility of the various extractions offers a challenge to both theory and experiment.

We note that $r_p^{(ep)}$ is by no means a directly determined quantity, because two-photon exchange effects do play a numerical role as well. Such corrections also appear in the context of the muonic-hydrogen analysis, though the effects turn out to be too small to explain the discrepancies. For example, a dispersive re-evaluation [302] of such hadronic effects based on experimental input (photo- and electroproduction of resonances off the nucleon and high-energy pomeron-dominated cross section) yields a contribution of $40\pm 5$ $\upmu$eV to the muonic hydrogen Lamb shift. Even if the error were underestimated for some unknown reason, its order of magnitude is insufficient to resolve the 300 $\upmu$eV discrepancy between direct measurement of the muonic Lamb shift [282, 285] and its expectation determined from QED theory and conventional spectroscopy. Such difficulties have prompted much discussion [289], and we refer to Sect. 3.2.6 for further details. It is still too speculative to state that we are confronting a violation of universality in the couplings of the electron and the muon, but hope that hadron contributions to the two-photon exchange between the muon and the proton would resolve the issue seems misplaced. Nevertheless, a viable BSM model does exist which would permit the discrepancy to stand [1492]. It predicts the existence of new parity-violating muonic forces which potentially can be probed through experiments using low-energy muon beams, notably through the measurement of a parity-violating asymmetry in elastic scattering from a nuclear target. Unfortunately, this picture cannot easily explain the existing muon $g-2$ discrepancy [1492]. Disagreement between theory and experiment lurks there also, but the precision of the discrepancy is two orders of magnitude smaller than in the muonic Lamb shift case. Indeed the muon $g-2$ result constrains new, muon-specific forces [1493]. Planned studies of $\mu p$ and $e p$ scattering at PSI should offer a useful direct test on the universality of lepton–proton interactions [1494].

#### Dark-matter searches

Various threads of astronomical evidence reveal that we live in a Universe dominated by dark matter and dark energy [1]. It is commonly thought that dark matter could be comprised of an as yet unidentified weakly interacting massive particle (WIMP). Such particles in the local solar neighborhood of our own Milky Way galaxy can be constrained or discovered through low-background experiments which search for anomalous recoil events involving the scattering of WIMPs from nuclei [1495, 1496]. Supersymmetric models offer a suitable candidate particle, the neutralino, which can be made compatible with all known astrophysical constraints [1497, 1498]. The neutralino is made stable by introducing a discrete symmetry, $R$ parity, that forbids its decay. An analogous discrete symmetry can be introduced in other, nonsupersymmetric new-physics contexts, such as in “little Higgs” models [1499], to yield an identical effect—generally, one can introduce a dark-matter parity that renders the dark-matter candidate stable.

WIMP–nuclear interactions mediated by $Z^0$ exchange were long-ago ruled out [1498, 1500], so that the WIMP of supersymmetric models is commonly regarded as a neutralino. Current experiments probe the possibility of mediation by Higgs exchange. Consequently, the spin-independent neutralino–nucleon cross section is particularly sensitive to the strange scalar density, namely, the value of $y=2 \langle N | \bar{s} s | N \rangle / \langle N | \bar{u} u + \bar{d} d | N \rangle$, noting [1501] and references therein, because the Higgs coupling increases with quark mass. The value of this quantity impacts the mapping of the loci of supersymmetric parameter space to the exclusion plot of WIMP mass versus the WIMP–nucleon cross section. Earlier studies relate $y$ to the $\pi N$ sigma term $\Sigma _{\pi N}$ via $y=1- \sigma _0/\Sigma _{\pi N}$ for fixed $\sigma _0 \equiv m_l \langle N | \bar{u} u + \bar{d} d - 2 \bar{s} s | N \rangle$ [1501], where $m_l\equiv (m_u+m_d)/2$, suggesting that the predicted neutralino–nucleon cross section depends strongly on the value of this phenomenological quantity [1502]. Its impact can be remediated, however, without recourse to assumptions in regards to SU(3)-flavor breaking; e.g., as shown in [1503], the couplings to the $u$- and $d$-quarks can be analyzed directly within the framework of $\mathrm{SU}(2)$ chiral perturbation theory (ChPT), permitting, in addition, control over isospin-breaking effects.

The matrix elements $m_\mathrm{s} \langle N | \bar{s} s | N \rangle$ and $\Sigma _{\pi N} \equiv m_l\langle N | \bar{u} u + \bar{d} d | N \rangle$ can also be computed directly in lattice-QCD, via different techniques, and the sensitivity to $\Sigma _{\pi N}$ is lessened [1502]. Several lattice-QCD groups have addressed this problem, and new results continue to emerge [1504–1506]. The spin-independent WIMP–nucleon cross section can be predicted to much better precision than previously thought, though the cross section tends to be smaller than that previously assumed [1502], diminishing the new physics reach of a particular WIMP direct detection experiment. Heavier quark flavors can also play a significant role in mediating the gluon coupling to the Higgs, and hence to the neutralino, and the leading contribution in the heavy-quark limit is well-known [1498, 1507]—and this treatment should describe elastic scattering sufficiently well. Nevertheless, the non-perturbative scalar charm matrix element should also be considered, and it has also been recently evaluated [1508]. We note, moreover, in the case of heavy WIMP–nucleon scattering, that the renormalization-group evolution from the weak to typical hadronic scales also plays a numerically important role [1509].

The effects of the nuclear medium in mediating effects beyond the impulse approximation (for scalar-mediated interactions) have also been argued to be important [1510]. This possibility has been recently investigated on the lattice, and the effects actually appear rather modest [1511]. Nevertheless, two-body exchange currents, which appear in chiral effective theory, can be important in regions of WIMP parameter space for which the usual WIMP–nucleon interaction is suppressed [1512]. For a study in spin-dependent WIMP–nuclear scattering see [1513].

#### Neutrino physics

The physics of QCD also plays a crucial role in the analysis of neutrino experiments, particularly through the axial-vector form factor of the nucleon (and of nuclei). The value of the axial coupling of the nucleon $g_A$, which is precisely measured in neutron $\beta$-decay, is key to the crisp interpretation of low-energy neutrino experiments such as SNO [1514]. In higher-energy experiments, however, the $q^2$ dependence of the axial form factor becomes important. In particular, elucidating the axial mass $M_A$, which reflects the rate at which the form factor changes with $q^2$, is crucial to the interpretation of neutrino oscillation experiments at $\mathcal{O}(1~\mathrm{GeV})$, an energy scale typical of accelerator-based studies. Commonly the value of $M_A$ is assessed experimentally by assuming the form factor can be described by a dipole approximation,

5.21 $$\begin{aligned} G_A^\mathrm{dipole}(q^2) = \frac{g_A}{\left[ 1- q^2/M_A^2\right] ^2} , \end{aligned}$$

and the nuclear effects, at least for neutrino quasi-elastic scattering, are assessed within a relativistic Fermi gas model, though final-state interactions of the produced hadrons in the nucleus can also be included. A consistent description of the neutrino–nuclear cross sections with beam energy and nuclear target is essential for future investigations of the neutrino mass hierarchy and CP violation in long-baseline experiments (LBNE, T2K, NO$\nu$A, CNGS). Within this framework, tension exists in the empirically determined values of $M_A$ [1462]. Moreover, recent studies at MiniBoone (http://www-boone.fnal.gov) have illustrated that the framework itself-appears to be wanting [1515, 1516]. Current and future studies at MINER$\nu$A (http://minerva.fnal.gov) can address these deficiencies by measuring the neutrino (and antineutrino) reaction cross sections with various nuclei [1517, 1518]. Model-independent analyses of experimental data have also been developed [1462] and have explored ways in which to relax the usual dipole parameterization of the axial form factor of the nucleon, as it is only an approximation. Nevertheless, a computation of the $q^2$ dependence of the nucleon axial form factor within QCD is greatly desired.

The value of $M_A$ can be estimated from the nucleon isovector axial form factor by a fit of its $q^2$ dependence to a dipole form. Alternatively, $r_A$, the axial radius, is calculated by taking the derivative of the form factors near $Q^2=0$, and they are linked through $r_A^2={12}/{M_A^2}$ (in the dipole approximation). The quantity $r_A$ is ultimately of greater interest as it is not tied to a dipole form. Lattice-QCD calculations of axial form factors, as well as of vector form factors, tend to yield smaller slopes and, thus, prefer a larger value of $M_A$ [206, 236, 1519, 1520]. This tendency may stem from a heavy pion mass or finite volume effects.

#### Cold nuclear medium effects

Many precision searches for new physics are undertaken within nuclear environments, be they dark-matter searches or studies of neutrino properties, and so far there is no universal understanding nor theoretical control over nuclear corrections. A common assumption is that the WIMP, or neutrino, interactions in the nucleus are determined by the sum of the individual interactions with the nucleons in the nucleus, as, e.g., in [1521, 1522]. This impulse-approximation picture treats nuclear-structure effects independently of the particle-physics interaction with a single nucleon. Nevertheless, single-particle properties can be modified in the nuclear medium, and evidence for such effects range from low-to-high energy scales. For example, at the lowest energy scales, the possibility of quenching of the Gamow–Teller strength in nuclei (with respect to its free-nucleon value) has been discussed for some time [1523–1526], though its source is unclear. It may be an artifact of the limitations of nuclear shell-model calculations^13^ [1528] or a genuine effect, possibly arising from meson-exchange currents in nuclei [1529]. At larger energies, in deep-inelastic lepton scattering from nuclei, medium effects are long established, most famously through the so-called EMC effect noted in $F_2$ structure function data [1530]. At $\mathcal{O}(1\,\mathrm{GeV})$ energy scales important for accelerator-based, long-baseline neutrino experiments, medium effects have also been observed in the studies of $3.5~\mathrm{GeV}$ neutrino–nuclear interactions in the MINER$\nu$A experiment [1517, 1518]. The inclusion of two-nucleon knock-out in addition to quasi-elastic scattering appears to be needed to explain the observed neutrino–nuclear cross sections at these energies [1531]. This is a challenging energy regime from a QCD viewpoint; the interactions of $\mathcal{O}(1\,\mathrm{GeV})$ nucleons are not suitable for treatment in chiral effective theory or perturbative QCD.

In-medium effects may also help explain older puzzles. For example, the NuTeV (http://www-e815.fnal.gov) experiment [1532] yields a value of $\sin ^2\theta _W$ in neutrino-nucleus scattering ${\sim } 3\sigma$ away from the SM expectation. This anomalous result can be explained, at least in part, by QCD effects, through corrections arising from modifications of the nuclear environment [1533].

Theoretical insight into these problems may prove essential to the discovery of new physics. Unfortunately, multibaryon systems are complicated to calculate in lattice QCD due to a rapid increase in statistical noise. An analogous, albeit simpler, system using many pions has been the subject of an exploratory study. This first lattice-QCD attempt to measure many-hadron modifications of the hadronic structure in a pion ($\pi ^+$) medium uses pion masses ranging 290–490 MeV at 2 lattice spacings [1534]. The preliminary result indicates strong medium corrections to the first moment of the pion quark-momentum fraction. With recent improvements to the efficiency of making quark contractions, which was one of the bottlenecks preventing lattice QCD from accessing even just 12-quark systems, we expect to see development toward structure calculations for light nuclei albeit at heavier pion masses within the next few years.

#### Gluonic structure

In the current global fit of the unpolarized parton distribution functions (PDFs) the gluonic contribution plays an enormously important role—roughly half of the nucleon’s momentum is carried by glue. However, gluonic structure has been notoriously difficult to calculate with reasonable statistical signals in lattice QCD, even for just the first moment. Despite these difficulties, gluonic structure has been re-examined recently, with new work providing approaches and successful demonstrations that give some hope that the problem can be addressed. Both $\chi$QCD [226, 1535] and QCDSF [1536] (note also [1537]) have made breakthroughs with updated studies of gluonic moments in quenched ensembles with lightest pion masses of 480 MeV. The two groups attack the problem using different techniques and show promising results, with around 15 % uncertainty when extrapolated to the physical pion mass. These methods are now being applied to gauge ensembles with dynamical sea quarks, and we expect to see updated results within a few years. Similar methods are also now used to probe the role of glue in the angular momentum of the proton [226, 1535].

Let us conclude this section more broadly and note that, in addition to these known effects, lattice-QCD matrix elements are also important to experiments which have not yet observed any events, such as $n$–$\bar{n}$ oscillations [1538] or proton decay [1539]. Lattice-QCD calculations can provide low-energy constants to constrain the experimental search ranges. The potential to search for new physics using these precision nucleon matrix elements during the LHC era and in anticipation of future experiments at Fermilab make lattice-QCD calculations of nucleon structure particularly timely and important.

### Quark flavor physics

The majority of the SM parameters have their origin in the flavor sector. The quark and lepton masses vary widely, which is an enduring puzzle. In this section we review studies of flavor and CP violation in the quark sector, usually probed through the weak decays of hadrons. In the SM the pattern of observed quark flavor and CP violation is captured by the CKM matrix, and the pattern is sufficiently distinctive that by overconstraining its parameters with multiple experiments and by employing accurate calculations, there is hope that an inconsistency between them (and therefore new physics) will ultimately emerge. At a minimum, this effort would allow the extraction of the CKM parameters with ever increasing precision. Extensive reviews of this issue already exist; we note the massive efforts of the Heavy Flavor Averaging Group [927] and the PDG [1] for experimental matters, as well as similar reviews of lattice-QCD results [44, 45, 1540]. Thus, we concentrate here on a few highlights suggested by very recent progress or promise of principle. We turn first, however, to two topics in non-CKM flavor physics which link to searches for BSM physics at low energies.

#### Quark masses and charges

*a. Light quark masses* The pattern of fermion masses has no explanation in the SM, but if the lightest quark mass were to vanish, the strong CP problem would disappear. Thus in light of our discussion of permanent EDMs and the new sources of CP violation that those experimental studies may reveal, it is pertinent to summarize the latest lattice-QCD results for the light quark masses. Current computations work in the isospin limit ($m_u=m_d$), treating electromagnetism perturbatively. Turning to the compilation of the second phase of the Flavour Lattice Averaging Group (FLAG2) [45], we note with $N_\mathrm{f}=2+1$ flavors (implying that a strange sea quark has been included) in the ${\overline{\mathrm{MS}}}$ scheme at a renormalization scale of $\mu =2$ GeV that

5.22 $$\begin{aligned} {m}_\mathrm{s}&= (93.8\pm 1.5 \pm 1.9)~\mathrm{MeV} ,\, \nonumber \\ {m}_{ud}&= (3.42\pm 0.06\pm 0.07)~\mathrm{MeV} , \end{aligned}$$

with $m_{ud} \equiv (m_{u} + m_{d})/2$, where the first error comes from averaging the lattice results and the second comes from the neglect of charm (and more massive) sea quarks. The $m_\mathrm{s}$ average value employs the results of [37, 39, 40, 348], whereas the $m_{ud}$ average value employs the results of [37, 39, 348, 1541]. To determine $m_u$ and $m_d$ individually additional input is needed. A study of isospin-breaking effects in chiral perturbation theory yields an estimate of $m_u/m_d$; this with the lattice results of (5.22) yields [45]

5.23 $$\begin{aligned} {m}_u&= (2.16\pm 0.09 \pm 0.07)~\mathrm{MeV} ,\, \nonumber \\ {m}_{d}&= (4.68\pm 0.14 \pm 0.07)~\mathrm{MeV} , \end{aligned}$$

where the first error represents the lattice statistical and systematic errors, taken in quadrature, and the second comes from uncertainties in the electromagnetic corrections.^14^ The electromagnetic effects could well deserve closer scrutiny.

Nevertheless, it is apparent the determined up quark mass is definitely nonzero. This conclusion is not a new one, even if the computations themselves reflect the latest technical advances, and it is worthwhile to remark on the ($m_u=0$) proposal’s enduring appeal. Ambiguities in the determination of $m_u$ have long been noted [1542, 1543]; particularly, Banks et al. [1543] have argued that both the real and imaginary parts of $m_u$ could be set to zero if there were an accidental U(1) symmetry predicated by some new, spontaneously broken, horizontal symmetry. This would allow $\delta$ of the CKM matrix to remain large at the TeV scale, while making $\bar{\theta }$ small. In this picture, a nonzero $m_u$ still exists at low scales, but it is driven by non-perturbative QCD effects (and the strong CP problem can still be solved if its impact on the EDM is sufficiently small). That is, in this picture $m_u$ is zero at high scales but is made nonzero at low scales through additive renormalization [1543]. Namely, its evolution from its low-scale value $\mu _u$ to a high-scale value $m_u$ ($m_u=0$) would be determined by

5.24 $$\begin{aligned} \mu _u = \beta _1 m_u + \beta _2 \frac{m_d m_\mathrm{s}}{\Lambda _\mathrm{QCD}} +\cdots , \end{aligned}$$

where $\beta _1$ and $\beta _2$ are dimensionless, scheme-dependent constants. This solution has been argued to be untenable because $m_d$ and $m_\mathrm{s}$ are guaranteed not to vanish (independently of detailed dynamical calculations) by simple spectroscopy and no symmetry distinguishes the $m_u=0$ point [1544, 1545]. This makes the notion of a zero up-quark mass ill-posed [1545] within strict QCD, though this does not contradict the proposal in [1543], precisely because their analysis takes the second term of (5.24) into account.

In [1543], $\mu _u$ on the left-hand side of (5.24) was argued to hold for the mass parameter of the chiral Lagrangian. The pertinent question is whether it applies to the masses of the QCD Lagrangian, obtained from lattice gauge theory. Because $\beta _2$ is scheme dependent, the answer depends on details of the lattice determination. Still, there is no evidence that the additive renormalization term is large enough to make the $m_u=0$ proposal phenomenologically viable [1546]. The proposal of [1543] could be independently falsified if the residual $\mathrm{Im}(m_u)$ effects at low energies could be shown at odds with the existing neutron EDM bounds.

*b. Quark charges* In the SM with a single generation, electric-charge quantization (i.e., unique $U(1)_Y$ quantum number assignments) is predicated by the requirement that the gauge anomalies cancel, and ensures that both the neutron and atomic hydrogen carry identically zero electric charge. There has been much discussion of the fate of electric charge quantization upon the inclusion of new physics degrees of freedom, prompted by the experimental discovery that neutrinos have mass—we refer to [1547] for a review. For example, enlarging the SM with a gauge-singlet fermion, or right-handed neutrino, breaks the uniqueness of the hypercharge assignments, so that electric charge is no longer quantized unless the new neutrino is a Majorana particle [1548, 1549]. This outcome can be understood in terms a hidden $B-L$ symmetry which is broken if the added particle is Majorana [1548–1551]. More generally, electric charge quantization is not guaranteed in theories for which the Lagrangian contains anomaly-free global symmetries which are independent of the SM hypercharge Y [1547].^15^ If neutrinoless double $\beta$ decay or neutron–antineutron oscillations are observed to occur, then the puzzle of electric-charge quantization is solved. Alternatively, if the charge neutrality of the neutron or atomic hydrogen were found to be experimentally violated, then it would suggest neither neutrinos nor neutrons are Majorana particles. Another pathway to charge quantization could lead from making the SM the low-energy limit of a grand unified theory, though this is not guaranteed even if such a larger theory occurs in nature. Ultimately, then, searches for the violation of charge neutrality, both of the neutron and of atoms with equal numbers of protons and electrons, probe for the presence of new physics at very high scales [1552]. Such a violation could also connect to the existence of new sources of CP violation [1553]. Novel, highly sensitive, experiments [1552, 1554] are under development, and plan to better existing limits by orders of magnitude.

**Fig. 40:**
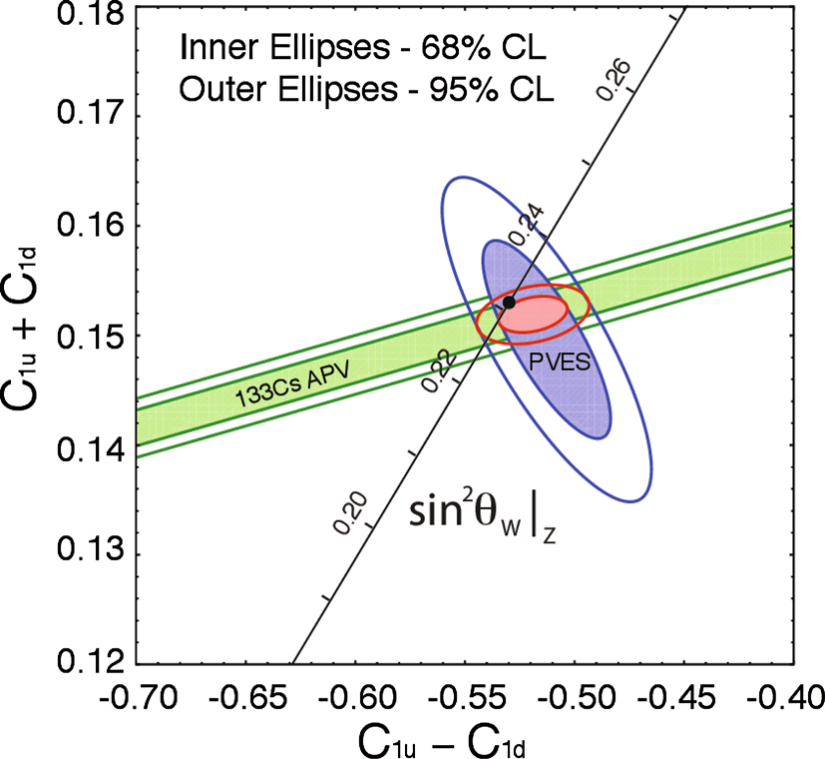
Constraints on the (neutral-current) weak couplings of the $u$ and $d$ quarks plotted in the $C_{1u}$–$C_{1d}$ plane. The *band* refers to the limits from APV, whereas the more *vertical ellipse* represents a global fit to the existing PVES data with $Q^2< 0.63~(\mathrm{GeV})^2$. The small, more *horizontal ellipse* refers to the constraint determined from combining the APV and PVES data. The SM prediction as a function of $\sin ^2\theta _W$ in the ${\overline{\mathrm{MS}}}$ scheme appears as a diagonal line; the SM best fit value is $\sin ^2\theta _W=0.23116$ [1]. Figure taken from [1321], and we refer to it for all details

Experimental measurements of parity-violating electron scattering (PVES) observables yield significant constraints on the weak charges of the quarks and leptons, probed through the neutral current. Recently, the weak charge of the proton $Q_W^p$ has been measured in polarized $\varvec{e}$–$p$ elastic scattering at $Q^2=0.025~(\mathrm{GeV})^2$ [1321]. This result, when combined with measurements of atomic parity violation (APV), yields the weak charge of the neutron $Q_W^n$ as well. The associated limits on the weak couplings of the quarks are shown in Fig. 40. For reference, we note that $Q_W^p=-2(2C_{1u} + C_{1d})$, where $C_{1i} = 2 g_A^e g_V^i$ and $g_A^e$ and $g_V^i$ denote the axial electron and vector quark couplings, respectively. The plot depicts an alternate way of illustrating the constraints on the $Q^2$ evolution of $\sin ^2\theta _W$ in the ${\overline{\mathrm{MS}}}$ scheme discussed in Sect. 3.5.

Non-perturbative QCD effects enter in this context as well, and we pause briefly to consider the extent to which they could limit the sensitivity of BSM tests in PVES. One notable effect is the energy-dependent radiative correction which arises from the $\gamma$–$Z$ box diagram. Dispersion techniques can be used to evaluate it [708–712, 1555, 1556], and the correction is demonstrably large, contributing to some 8 % of $Q_W^p$ in the SM [1]. Currently the dispersion in its assessed error is greater than that in the predicted central value, though the expected error can be refined through the use of additional PDF data [1556]. Charge-symmetry-breaking effects in the nucleon form factors may eventually prove significant as well but are presently negligible as they should represent a $\lesssim 1~\%$ correction [1557–1561]. The implications of such theoretical errors, which appear manageable at current levels of sensitivity, could eventually be assessed in a framework analogous to that recently developed for neutron decay observables [1465].

#### Testing the CKM paradigm

We begin by presenting the moduli of the elements $V_{ij}$ of the CKM matrix determined in particular charged-current processes, using the compilation of [1]:

5.25 $$\begin{aligned}&\begin{array}{|ccc|} |V_{ud}| &{} |V_{us}| &{} |V_{ub}| \\ |V_{cd}| &{} |V_{cs}| &{} |V_{cb}| \\ |V_{td}| &{} |V_{ts}| &{} |V_{tb}| \end{array} \propto \quad \quad \quad \quad \quad \quad \\ \nonumber&\begin{array}{|ccc|} 0.97425\!\pm \! 0.00022 &{} 0.2252\! \pm \! 0.0009 &{} 0.00415\! \pm \! 0.00049 \\ 0.230 \!\pm \! 0.011 &{} 1.006\! \pm \! 0.023 &{} 0.0409 \!\pm \! 0.0011 \\ 0.0084 \!\pm \! 0.0006 &{} 0.0429 \!\pm \! 0.0026 &{} 0.89 \!\pm \! 0.07 \end{array} .\quad \end{aligned}$$

Quark intergenerational mixing is characterized by the parameter $\lambda \approx |V_{us}|$, with mixing of the first (second) and third generations scaling as $\mathcal{O}(\lambda ^3)$ ($\mathcal{O}(\lambda ^2)$) [1562]. The CKM matrix $V_\mathrm{CKM}$ can be written in terms of the parameters $\lambda , A, {\bar{\rho }}, {\bar{\eta }}$; thus parametrized it is unitary to all orders in $\lambda$ [1369, 1563]. We test the SM of CP violation by determining whether all CP-violating phenomena are compatible with a universal value of $({\bar{\rho }}, {\bar{\eta }})$ (note [1] for the explicit connection to $\delta$).

**Fig. 41:**
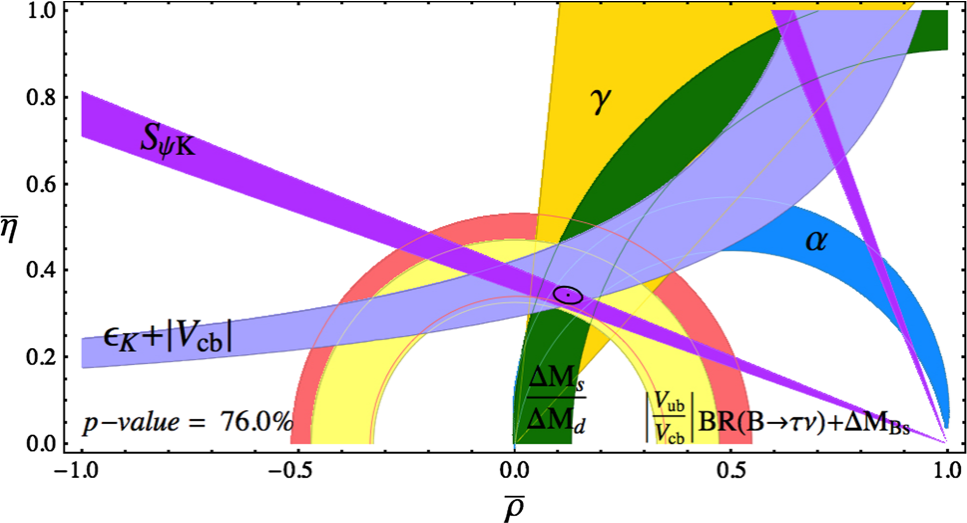
Precision test of the SM mechanism of CP violation in charged-current processes realized through the comparison of the parameters $\bar{\rho }$ and $\bar{\eta }$ determined through various experimental observables and theory inputs from lattice QCD. The experimental inputs are as of September 2013, and the lattice inputs are derived from published results through April 30, 2013; the figure is an update of those in Ref. [1540]

Current constraints are illustrated in Fig. 41. The so-called unitarity triangle in the ${\bar{\rho }}$–${\bar{\eta }}$ plane has vertices located at $(0,0)$, $(1,0)$, and $(\bar{\rho }_\mathrm{SM},\bar{\eta }_\mathrm{SM})$. The associated interior angles at each vertex are $\gamma (\phi _3)$, $\beta (\phi _1)$, and $\alpha (\phi _2)$, respectively. The CP asymmetry $S_{\psi K}$ is realized through the interference of $B^0$–$\bar{B}^0$ mixing and direct decay into $\psi K$ and related modes. It is $\sin 2\beta$ in the SM up to hadronic uncertainties which appear in $\mathcal{O}(\lambda ^2)$. The other observables require hadronic input of some kind to determine the parameters of interest; lattice-QCD calculations are essential to realize the precision of the tests shown in Fig. 41.

The constraints thus far are consistent with the SM of CP violation; the upper $S_{\psi K}$ band in the $\bar{\rho }-\bar{\eta }$ plane arising from a discrete ambiguity has been ruled out by the determination that $\cos 2\beta > 0$ at 95 % C.L. [1564]. Experimental studies of CP violation in the $B$ system continue, and we note an improved constraint on $\gamma$ of $\gamma =67^\circ \pm 12^\circ$ from LHCb [1565], which is consistent with the SM and with earlier B-factory determinations [1566]. Certain, early anomalies in B-physics observables can be explained by a possible fourth SM-like generation [1567, 1568], and it remains an intriguing idea. Its existence, however, is becoming less and less consistent with experimental data. Direct searches have yielded nothing so far [1569], and a fourth SM-like generation is disfavored by the observation of the Higgs, and most notably of $H\rightarrow \gamma \gamma$, as well [1323, 1570]. Flavor and CP violation are well-described by the CKM matrix [1367], so that it has become popular to build BSM models of the electroweak scale which embed this feature. That is, flavor symmetry is broken only by the standard Yukawa couplings of the SM; this paradigm is called Minimal Flavor Violation (MFV) [1571–1573].

The structure of the CKM matrix can also be tested by determining whether the empirically determined elements are compatible with unitarity. Figure 41 illustrates that unitarity is maintained if probed through the angles determined from CP-violating observables, that is, e.g., $\alpha +\beta +\gamma =(178^{+11}_{-12})^\circ$ [1]. The most precise unitarity test comes from the first row [1], namely, of whether $\Delta _u\equiv |V_{ud}|^2 + |V_{us}|^2 + |V_{ub}|^2 -1$ is nonzero. The contribution of $|V_{ub}|^2$ is negligibly small at current levels of sensitivity, and for the last, several years the uncertainty has been dominated by that in $|V_{us}|$ [1]. This situation changed, however, in 2013 with new, precise calculations of kaon decay parameters in lattice QCD becoming available [1574–1576]. The quantity $|V_{ij}|^2 (\delta |V_{ij}|)^2$ determines the impact of a CKM matrix element on the unitarity test, and by this measure that of $|V_{us}|$ and $|V_{ud}|$ [1577] are now comparable [1575, 1576]. Consequently, the earlier result [1]

5.26 $$\begin{aligned} \Delta _u = -0.0001\pm 0.0006 , \end{aligned}$$

becomes, using the average value of $f_{K^\pm }/f_{\pi ^\pi }$ in QCD with broken isospin from [45], $\Delta _u =0 \pm 0.0006$. By averaging over computations in $N_\mathrm{f}=2+1+1$, $N_\mathrm{f}=2+1$, and $N_\mathrm{f}=2$ ensembles the improvements associated with the included (published at that time) $N_\mathrm{f}=2+1+1$ computation [1574] is muted, begging the question of whether it is appropriate to average calculations which differ in their quenching of heavier sea quarks.^16^ The use of the most precise kaon results yields a tension with CKM unitarity [1575, 1576]. The value of $|V_{us}|$ can also be determined from $\tau$ decay, and the situation there is quite different. The inclusive $\tau$ decay data yield a value of $|V_{us}|$ which is less precisely determined but still different from the one assuming 3-flavor CKM unitarity by three sigma [45, 927]; more theoretical [718, 1578–1580] and experimental work will likely be needed to determine the origin of the discrepancy. We refer to Sect. 3.5.3 for a discussion of the determination $\alpha _\mathrm{s}$ in hadronic $\tau$ decays, needed for a determination of $|V_{us}|$.

The most precise determination of $|V_{ud}|$, $|V_{ud}|=0.97425 \pm 0.00022$ [1577], comes from the study of superallowed ($0^+\rightarrow 0^+$) transitions in nuclei. Its error is dominated by theoretical uncertainties, particularly from Coulomb corrections in the nuclear matrix elements and other nuclear-structure-dependent effects [1577] and from the evaluation of the $\gamma$–$W$ box diagram [1581–1583]. The assessment of nuclear Coulomb corrections [1577] has been criticized as incomplete [1584, 1585], though it has been experimentally validated in a superallowed decay for which the corrections are particularly large [1586]. Another unitarity test comes from the second row; this can either be accessed directly through determinations of the $V_{ij}$ or indirectly though the leptonic width of the $W$, for which the hadronic uncertainties are trivially small. The former procedure yields $\Delta _\mathrm{c} \equiv |V_{cd}|^2 + |V_{cs}|^2 + |V_{cb}|^2 -1 = 0.04 \pm 0.06$ [45], whereas the latter yields $\Delta _\mathrm{c} = 0.002 \pm 0.027$ [1], making the indirect method more precise.

Theory plays a key and indeed expanding role in making all these tests more precise, so that increasingly the comparison between theory and experiment becomes a test field for QCD. We now consider some of the theory inputs in greater detail.

*a. Theory inputs for* $V_{us}$ Until very recently, the error in $V_{us}$ dominated that of the first-row CKM unitarity test. Here, we consider different pathways to $V_{us}$ through meson decays; as we have noted, such efforts parallel the extraction of $V_{us}$ from $\tau$ decays [714, 1580, 1587, 1588].

Typically, $V_{us}$ has been determined through $K\rightarrow \pi {\ell } \nu$ ($K_{{\ell }3}$) decays and for which the following formula for the decay width applies [1589]:

5.27 $$\begin{aligned} \Gamma (K_{{\ell } 3})&= \frac{G_\mathrm{F}^2 m_K^5}{128\pi ^3} C_K^2 S_{\mathrm{EW}} (1+\delta _\mathrm{SU(2)}^{K\pi } + \delta _\mathrm{EM}^{K\ell }) \nonumber \\&\times |V_{us}|^2 |f_+^{K^0\pi ^{-}}(0)|^2 I_{K\ell } , \end{aligned}$$

which includes various electroweak, electromagnetic, and isospin-breaking corrections, in addition to the phase space integral $I_{K\ell }$ and other known factors. We have separated in the second line two of the most interesting ones. The first is the wanted CKM matrix element, and the second is a hadronic form factor to be evaluated at zero-momentum transfer. The form factors $f_{\pm }^{K\pi }(t)$ are determined by the QCD matrix elements

5.28 $$\begin{aligned}&\langle \pi (p_\pi )|{\bar{s}}\gamma _{\mu } u| K(p_K)\rangle \nonumber \\&\quad = (p_\pi + p_K)_{\mu } f_+^{K\pi }(t) + (p_\pi - p_K)_{\mu } f_-^{K\pi }(t), \end{aligned}$$

where $t=(p_K- p_\pi )^2$, and we note

5.29 $$\begin{aligned} \delta _\mathrm{SU(2)}^{K \pi } = (f_+^{K\pi }(0)/f_+^{K^0\pi ^{-}}(0))^2 -1 . \end{aligned}$$

There are, in principle, five different widths to be determined, in $K^\pm _{e3}, K^\pm _{\mu 3}, K_{Le3}, K_{L\mu 3}$, and $K_{S\mu 3}$ decay, and the corrections in each can differ. Moreover, real-photon radiation also distinguishes the various processes, and it must be treated carefully to determine the experimental decay widths [1589]. Great strides have been made in the analysis of the various corrections [1589–1591], which are effected in the context of chiral perturbation theory, and it is reasonable to make a global average of the determinations of $V_{us} f_+(0)$ in the various modes [1]. Progress also continues to be made on the experimental front, there being new measurements of $K^\pm \rightarrow \pi ^0 l^\pm \nu$ by the NA48/2 experiment at CERN [1592]. The updated five-channel average is $f_+(0)|V_{us}|=0.2163 \pm 0.0005$ [1593]. The $t$ dependence of the form factor is embedded in the evaluation of $I_K^{\ell }$ in (5.27). NA48/2 has selected events with one charged lepton and two photons that reconstruct the $\pi ^0$ meson and extract form factors that they fit with either a quadratic polynomial in $t$, or a simple pole ansatz (be it scalar or vector),

5.30 $$\begin{aligned} f_{+,0}(t) = \frac{m_{v,s}^2}{m_{v,s}^2-t}, \end{aligned}$$

where $f_0(t)=f_+(t) + (t/(m_K^2 - m_\pi ^2))f_-(t)$. A good fit is obtained with $m_v= 877 \pm 6$ MeV and $m_\mathrm{s}=1176 \pm 31$ MeV; these quantities do not precisely correspond to known particles but are of a reasonable magnitude. We detour, briefly, to note that the systematic error in the precise choice of fitting form can be mitigated through considerations of analyticity and crossing symmetry [1594, 1595]; the latter permits the use of experimental data in $\tau \rightarrow K \pi \nu$ decays [1595] to constrain the fitting function. Finally we turn to the determination of $f_+(0)$, for which increasingly sophisticated lattice-QCD calculations have become available. Noting [45], we report $[N_\mathrm{f} =2]$ [1596] and $[N_\mathrm{f} =2 +1]$ [1597] results:

5.31 $$\begin{aligned}&f_+(0) = 0.9560 \pm 0.0057 \pm 0.0062 \quad [N_\mathrm{f} =2] \nonumber \\&f_+(0) = 0.9667 \pm 0.0023 \pm 0.0033 \quad [N_\mathrm{f} =2 +1] \end{aligned}$$

as well as [1576]

5.32 $$\begin{aligned} f_+(0) = 0.9704 \pm 0.0024 \pm 0.0022 \quad [N_\mathrm{f} =2 +1 +1]. \end{aligned}$$

Using the last value for $f_+(0)$, which attains a physical value of the pion mass, and those of $|V_{us}| f_+(0)$ and $V_{ud}$ we have reported, yields $\Delta _u=-0.00115\pm 0.00040 \pm 0.0043$, where the first (second) error is associated with $V_{us}$ ($V_{ud}$), and roughly a $2\sigma$ tension with unitarity [1576].

As a final topic we consider the possibility of determining $V_{us}/V_{ud}$ from the ratio of $K_{\ell 2 (\gamma )}$ and $\pi _{\ell 2 (\gamma )}$ decay widths with the use of the decay constant ratio $f_K/f_\pi$ computed in lattice QCD [1598]. This method competes with the $K_{\ell 3}$ decays in precision. In a recent development, the isospin-breaking effects which enter can now be computed using lattice-QCD methods as well; the method is based on the expansion of the Euclidean functional integral in the terms of the up-down mass difference [560, 590]. Generally, the separation of isospin-breaking effects in terms of up-down quark mass and electromagnetic contributions is one of convention, because the quark masses themselves accrue electromagnetic corrections which diverge in the ultraviolet [560, 1599]. Technically, however, the pseudoscalar meson decay constants are only defined within pure QCD, so that

5.33 $$\begin{aligned} \frac{f_{K^+}}{f_{\pi ^+}} = \frac{f_{K}}{f_{\pi }} \left( 1 + \delta _\mathrm{SU(2)}\right) , \end{aligned}$$

where $f_K/f_\pi$ are evaluated in the isospin-symmetric ($m_u=m_d$) limit. Thus we can crisply compare the ChPT determination of $\delta _\mathrm{SU(2)}$ [1600] with a completely different non-perturbative method. Namely, noting [1599], we have $\delta _\mathrm{SU(2)}^\mathrm{ChPT}=-0.0021\pm 0.0006$ [1600], whereas $\delta _\mathrm{SU(2)}^\mathrm{lattice}=-0.0040\pm 0.0003 \pm 0.0002$ [590] and $\delta _\mathrm{SU(2)}^\mathrm{lattice}=-0.0027\pm 0.006$ [1575]. Thus tension exists in the various assessments of SU(2)-breaking effects, and it will be interesting to follow future developments.

*b.* $B$ *and* $D$ *form factors* Lattice-QCD methods can also be used for the computation of the $B$ and $D$ meson form factors in exclusive semileptonic decays, yielding ultimately additional CKM matrix elements once the appropriate partial widths are experimentally determined. Generally, CKM information can be gleaned from both exclusive and inclusive (to a final state characterized by a quark flavor $q$, as in $B\rightarrow X_q {\ell } \nu$ decay) $B$ meson decays, and different theoretical methods figure in each. In the inclusive case, the factorization of soft and hard degrees of freedom is realized using heavy quark effective theory, and the needed non-perturbative ingredients are determined through fits to data. As we have noted, lattice-QCD methods can be employed in the exclusive channels, and the leptonic process $B\rightarrow \tau \nu$, along with a lattice-QCD computation of the decay constant $f_B$, also yields $|V_{ub}|$, though this pathway is not yet competitive with other methods. We refer to Sect. 4 for a detailed discussion, though we note that tension continues to exist between the various determinations of $|V_{ub}|$ and $|V_{cb}|$. In particular, an exclusive extraction from $B\rightarrow \pi l \nu$ decay has been made using form factors computed with $[N_\mathrm{f} = 2+1]$ dynamical quark flavors by HPQCD [1601] and FNAL/MILC [1376]. A simultaneous fit of the lattice and experimental form factors to determine their relative normalization $|V_{ub}|$ yields [45] $|V_{ub}|=0.00337 \pm 0.00021$ (BaBar [937]) and $|V_{ub}|=0.00347 \pm 0.00022$ (Belle [938]). These values remains below the inclusive determination of $0.00440\pm 0.00025$ [927]. The two determinations remain to be reconciled, perhaps by better measurements separating the background charm decays of the $B$, since the theoretical determination in terms of

5.34 $$\begin{aligned} \frac{d\Gamma (\bar{B}^0\rightarrow \pi ^+l\bar{\nu })}{dq^2} = \frac{G_\mathrm{F}^2 | {\mathbf {p}}_\pi |^3}{24\pi ^3} | V_{ub}|^2 | f_+(q^2)|^2 \end{aligned}$$

seems crisp, though it could be aided perhaps by better resolution of the $q^2$ dependence of the form factor(s).

A similar situation is found in comparing the exclusive and inclusive determinations of $|V_{cb}|$ where there remains roughly a $2\sigma$ tension between the results. This parameter is important in many instances, for example in tightening constrained-MFV models [1602]. The exclusive extraction requires determining the form factors of ${d\Gamma (B\rightarrow (D/D^*)+ l\nu )}/{d({\mathbf {v}}_b \cdot {\mathbf {v}}_\mathrm{c})}$ at the zero recoil point. Only a single calculation, of the $B\rightarrow D^*$ form factor, currently satisfies the FLAG criteria [45]. This result, the 2010 FNAL/MILC calculation [1603], employing $N_\mathrm{f}=2+1$ dynamical quark flavors, yields $|V_{cb}|_\mathrm{exc}=0.003955 \pm 0.000072 \pm 0.000050$ [45], where the errors denote lattice and non-lattice (experiment and non-lattice theory) uncertainties, respectively. This is to be compared with $|V_{cb}|_\mathrm{inc}=0.004242 \pm 0.000086$ [1604]; the two results are discrepant at about $2.3\sigma$. New lattice calculations of the $B\rightarrow D^{(*)}$ form factors are in progress; presumably this will improve the situation considerably. Alternatively, the possibility of higher-order effects in the heavy-quark expansion for inclusive B decays, particularly those due to “intrinsic charm,” have been discussed [1605–1607], though their magnitude has not yet been established.

In charm decays, the determinations of $|V_{cd}|$ and $|V_{cs}|$ via leptonic and semileptonic modes are in reasonably good agreement. The $|V_{cd}|$ determinations are all consistent within errors, whereas the $|V_{cs}|$ in leptonic and semileptonic modes disagree at $1.2 \sigma$ [45]. Using the results, e.g., for $f_+^{D\pi }(0)|V_{cd}|$ and $f_+^{DK}(0)|V_{cs}|$ from [927], and the form factor calculation of the only $N_\mathrm{f}=2+1$ lattice calculation to satisfy FLAG criteria in each case ([1608] and [1609], respectively), yields $|V_{cd}|=0.2192 \pm 0.0095 \pm 0.0045$ and $|V_{cs}|=0.9746 \pm 0.0248 \pm 0.0067$ [45]. For reference, from neutrino scattering one has $|V_{cd}|=0.230\pm 0.011$ [1].

*c. Tests of lepton-flavor universality in heavy-light decays* Heavy-light semileptonic processes can also be used to challenge the SM with minimum theory input, through tests of lepton-flavor universality [1610–1612]. In particular, we recall from Sect. 4 that BaBar has measured the ratio

5.35 $$\begin{aligned} R(D) \equiv \frac{\mathcal{B}(B\rightarrow D \tau \nu )}{\mathcal{B}(B\rightarrow D{\ell }\nu )} = 0.440\pm 0.058 \pm 0.042 , \end{aligned}$$

with ${\ell }\in e, \mu$, and substituting the $D$ for a $D^*$ yields $R(D^*)=0.332\pm 0.024 \pm 0.018$ [933]. These ratios are in excess of SM predictions, at $2.0\sigma$ and $2.7\sigma$, respectively, and the apparent, observed violation of lepton-flavor universality can be mediated by a new charged Higgs boson [933]. The measured ratio of ratios, however, appears to be odds with the Type II two-Higgs-doublet model [933], though there are many other BSM possibilities which can generate an effect [1613–1615]. The ratio $R(D)$ has been revisited by the FNAL/MILC collaboration to find $R(D)=0.316\pm 0.014$ [1616]), a value of some $1.7\sigma$ smaller than the BaBar result if the errors are combined in quadrature. Nevertheless, their study illustrates the importance of the computation of the scalar form factor to the prediction of $R(D)$, and we look forward to future results in regard to $R(D^*)$. We note that combining the $R(D)$ and $R(D^*)$ experimental results currently yields a disagreement of $3.4\sigma$ with the SM [933]. Lattice-QCD methods will also no doubt be important to evaluating the success of a particular BSM model in confronting the experimental values of $R(D)$ and $R(D^*)$.

**Fig. 42:**
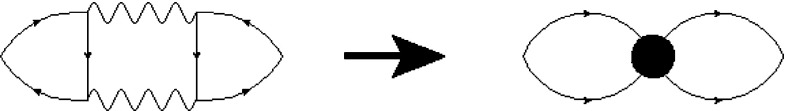
The box diagram for neutral meson mixing with double $W$ exchange (as in the SM) reduces at low energy to the matrix element of a contact four-quark operator that yields the “bag parameter”

*d. Neutral meson mixing and bag parameters* The non-perturbative matrix element associated with neutral-meson mixing, depicted schematically in Fig. 42, is termed the bag parameter $B_\mathrm{mes}$; it captures the deviation of the operator matrix element from its vacuum insertion value for which $B_\mathrm{mes}=1$. In the kaon system, it is essential to an understanding of $|\epsilon _K|$ [1], the parameter which characterizes CP violation in $K^0$–$\bar{K}^0$ mixing, and whose interpretation in terms of CKM parameters has languished for decades. In the $K^0$–$\bar{K}^0$ system $B_K$ is given by

5.36 $$\begin{aligned} B_K = \frac{\langle \bar{K}^0\arrowvert {\mathcal {O}}_{LL}^{\Delta S=2} \arrowvert K^0\rangle }{\frac{8}{3}f_K^2 m_K^2}\, , \end{aligned}$$

at some scale $\mu$, where ${\mathcal {O}}_{LL}^{\Delta S=2} = (\bar{s} \gamma ^\mu (1-\gamma _5) d)(\bar{s} \gamma _{\mu } (1-\gamma _5) d)$, and from which the renormalization-group-invariant (RGI) quantity $\hat{B}_K$ can be determined [1617]. Several $N_\mathrm{f}=2+1$ calculations now exist, and their average (specifically of [37, 38, 1618–1620]) yields $\hat{B}_K=0.766 \pm 0.0010$ [45]. Since $\hat{B}_K$ is now known to some 1.3 %, the ability to interpret $\epsilon$ has changed dramatically for the better. This improvement is captured in the width of the $|\epsilon _K|$ band in Fig. 41, and the dominant residual uncertainties in its interpretation come from that in $|V_{cb}|$, which enters as $|V_{cb}|^4$, and in the perturbative contribution from $c\bar{c}$ quarks [1621]. Concerning new physics searches, the computation of a complete set of $|\Delta S|=2$ hadron operators for $K^0$–$\bar{K}^0$ mixing is under way by several collaborations, including the ETMC [1622] and RBC/UKQCD [1623]. This should help constrain the couplings to and masses of additional particles beyond the SM.

The parameter which characterizes direct CP violation in the kaon system, $\mathrm{Re}(\epsilon '/\epsilon )$, is definitely nonzero, $\mathrm{Re}(\epsilon '/\epsilon )=(1.67\pm 0.23)\times 10^{-3}$ [1], and probes $\mathrm{Im}(V_{td}V^*_{ts})$, though large theoretical hadronic uncertainties beset its interpretation. This arises due to the approximate cancellation of the gluonic and electroweak penguin contributions, exacerbating the role of isospin-violating effects [1624, 1625], so that effects beyond $\pi ^0-\eta ,\eta ^\prime$ mixing can also play an important role [1626–1628]. The recent strides in lattice-QCD computations has spurned progress in this system as well [1629, 1630], though an analysis of its isospin-breaking effects would seem beyond the scope of current ambitions.

Mixing in the $B^0_{s}\bar{B}^0_{s}$ system has now also been established [1631, 1632], and the comparison of its measured mixing parameters with those of the $B^0_{d}\bar{B}^0_{d}$ system yields a precision test of the SM. The mass difference between the weak-interaction eigenstates in the SM is given by

5.37 $$\begin{aligned} \Delta M_{q}|_\mathrm{SM} \propto |V^*_{t(q)}V_{tb}|^2 M_{B_{q}} f_{B_{q}}^2 \hat{B}_{B_{q}} , \end{aligned}$$

with $q\in (d,s)$ and where we define $B_{B_q}$ after (5.36), noting $\hat{B}_{B_{q}}$ is the RGI quantity. The constant of proportionality is common to the two systems, so that the ratio $(\Delta M_\mathrm{s}/\Delta M_d)_\mathrm{SM}$ is determined by $\xi ^2 M_{B_\mathrm{s}}/M_{B_d}$, where the non-perturbative parameter

5.38 $$\begin{aligned} \xi =\frac{f_{B_{s}} \sqrt{B_{B_{s}}}}{f_{B_{d}} \sqrt{B_{B_{d}}}}\, \end{aligned}$$

can be computed in lattice QCD. Its deviation from unity measures the size of SU(3)$_\mathrm{f}$ breaking. There have been several $N_\mathrm{f}=2+1$ lattice-QCD calculations of this quantity, but only one passes all the FLAG criteria [45], so that we report $\xi =1.268 \pm 0.063$ [1633]. Confronting $\Delta M_q$ directly is a much more challenging task, though this, as well as the matrix elements of all five (leading-dimension) operators that can generate $B^0_{q}$–$\bar{B}^0_{q}$ mixing system are under analysis [1633]. Such efforts are crucial to determining to what extent BSM efforts operate in the $B^0_{q}\bar{B}^0_{q}$ system (and in the $K^0\bar{K}^0$ system by comparison) and to constrain the models which could generate them [1634].

#### New windows on CP and T violation

We now review some recent results in the study of CP and T violation. Several new results concern searches for direct CP violation in systems for which such observables are parametrically small in the SM. The key issue is how large the latter can possibly be; can the observation of a larger-than-expected CP asymmetry be an imprimatur of new physics? Direct CP violation, such as a rate asymmetry in $B\rightarrow f$ and $\bar{B} \rightarrow \bar{f}$ decays, follows from the quantum interference of two amplitudes, typically termed “tree” and “penguin” as per their quark-flow diagrams, that differ in both their strong and weak phase. Unfortunately the tree-penguin interference effects which give rise to the CP asymmetries are notoriously challenging to calculate and can be subject to non-perturbative enhancement. It is probably better to be cautious in considering a larger-than-expected CP asymmetry as evidence of new physics. To give a context to this assessment we offer a terse overview of the theory of nonleptonic $B$-meson decays, though the essential ideas apply to the analysis of nonleptonic $D$ and $K$ decays as well.

Early exploratory studies employed “vacuum saturation” [1635] or “generalized factorization” [1636, 1637] to evaluate the matrix elements of the dimension-six operators which appear in $B$ decays, though such work had conceptual and computational limitations [1638]. Alternatively, the large energy release of $B$ decays to light hadrons motivates the use of flavor-symmetry-based (of the $u$, $d$, $s$ valence quarks) strategies, relating experimental data in various final states to determine the ill-known amplitudes, as in, e.g., [1639]. Such strategies are approximate and may fail as constraints on new physics become more severe.

The effective Hamiltonian for $|\Delta B|=1$ processes at the $b$-quark mass scale has been known to NLO precision for some time [1640, 1641]. The construction of a “QCD factorization,” based on the combined use of the heavy quark and strong-coupling-constant expansions, was a major step forward —the scale dependence of the decay amplitude (combining the pieces from the Wilson coefficients with those from the evaluation of the matrix elements of the associated local operators) was shown, for the first time, to vanish in NLO precision and at leading power in the heavy-quark expansion [1642, 1643]. The approach has been applied to a sweep of two-body $B$-meson decays and works fairly well [1644, 1645], though there are some systemic problems. The theory has difficulties confronting empirical branching ratios in modes for which the tree amplitude is suppressed, and it has trouble confronting CP-asymmetries. Systematic study suggests that the power corrections (in the heavy-quark mass) are phenomenologically important [1646, 1647], though explicit studies of NNLO corrections in $\alpha _\mathrm{s}$ [1648–1650] have also eased tension with the data in the explicit modes studied [1651]. Other approaches include the use of SCET [1652–1654] and of $k_\mathrm{T}$ factorization [1655–1658]; the latter does not take the heavy-quark limit. Possible troubles with power corrections in the heavy-quark mass do not bode well for the analysis of hadronic $D$ decays, though the $k_\mathrm{T}$ factorization approach can be and has been used [1659].

*a. Three-body decays and Dalitz plot analyses* Two-body decays have been much studied in the analysis of CP-violating observables in the $B$ system, but the large phase space available in the decay of $B$ mesons to light hadrons makes three-body final states far more copious. Such final states are theoretically more difficult to handle because factorization theorems in QCD of exclusive heavy-light decays exist for two-body final states [1642, 1643, 1652]. However, flavor-based analyses, such as an isospin analysis of $B\rightarrow \rho \pi$ [1660], can nevertheless be successful in extracting CKM phase information, even in the presence of SM isospin-breaking effects such as $\pi ^0-\eta ,\eta ^\prime$ mixing [1661]. A great deal of information is encoded in the Dalitz plot of the final state and can yield new pathways to the identification of direct CP violation [1662–1667], both in the $B$ and $D$ system. Interpreting the Dalitz plot requires good control of the manner in which the various resonances can appear, making the use of simple Breit–Wigner forms insufficient. Rather, constraints from low-energy chiral dynamics, including those of unitarity and analyticity, such as realized in Omnès-based approaches, should be brought to bear [1661]. The latter are gaining ground [1668–1670].

Recently LHCb has reported an enhanced signal of CP violation in $B^\pm \rightarrow K^\pm \pi ^+\pi ^{-}$ and $B^\pm \rightarrow K^\pm K^{+} K^{-}$ final states [1671, 1672], and theoretical work has concerned, e.g., whether the effects are consistent with $U$-spin symmetry [1673], as well as the particular Dalitz-plot interference mechanisms needed to explain them [1674].

*b. CP violation in the* $B_\mathrm{s}$ *system* Recently, the LHCb collaboration has made a series of measurements probing the decays of $B_\mathrm{s}^0 (\bar{B}_\mathrm{s}^0)$ mesons to different CP-eigenstates, specifically $J/\psi \phi$ [1675], $J/\psi f_0$ [1676], $J/\psi \pi ^+\pi ^{-}$ [1677, 1678], and $J/\psi K^+K^{-}$ [1678]. We note that ATLAS has studied $B_\mathrm{s}\rightarrow J/\psi \phi$ as well [1679]. The CP-violating phase $\phi _\mathrm{s}$, which is determined by the argument of the ratio of the off-diagonal real and dispersive pieces in $B_q^0$–$\bar{B}_q^0$ mixing, and other mixing parameters are also determined, which include the average $B_\mathrm{s}^0$ decay width $\Gamma _\mathrm{s}$ and the width difference $\Delta \Gamma _\mathrm{s}$. The measurement of $B_\mathrm{s} \rightarrow J/\psi \phi$ alone leaves a two-fold ambiguity in $(\phi _\mathrm{s},\Delta \Gamma _\mathrm{s})$ but this can be resolved by the study of the $J/\psi K^+K^{-}$ final state with the invariant mass of the $K^+K^{-}$ pair. The latest LHCb results are [1678]:

5.39 $$\begin{aligned} \phi _\mathrm{s}&= 0.07 \pm 0.09 \pm 0.01 \,\mathrm{rad} \quad [J/\psi K^+K^{-}], \end{aligned}$$

5.40 $$\begin{aligned} \phi _\mathrm{s}&= 0.01 \pm 0.07 \pm 0.01 \,\mathrm{rad} \quad [\mathrm{combined}], \end{aligned}$$

where “combined” refers to a combined fit of $J/\psi K^+K^{-}$ and $J/\psi \pi ^+\pi ^{-}$ events. The enumerated errors are statistical and systematic, respectively. In the SM, ignoring subleading penguin contributions, $\phi _\mathrm{s} = -2\beta _\mathrm{s}$, where $\beta _\mathrm{s}= \mathrm{arg}\left( - {V_{ts} V_{tb}^*}/{V_{cs} V_{cb}^*}\right)$, and indirect global fits assuming the SM yield $2\beta _\mathrm{s}=0.0364\pm 0.0016\,\mathrm{rad}$ [1680]. The $\phi _\mathrm{s}$ result, as well as those for $\Gamma _\mathrm{s}$ and $\Delta \Gamma _\mathrm{s}$ are compatible with SM expectations [1680, 1681]. CP violation has been observed in the $B_\mathrm{s}^0$ system, however, in $B_\mathrm{s}^0\rightarrow K^{-}\pi ^+$ decay [1682].

*c. CP violation in the* $D$ *system* There has been much interest in probing CP violation in the $D$ system since the common lore is that a CP asymmetry in excess of $10^{-3}$ in magnitude would be a signal of new physics [1683]. The $D$ meson is produced copiously by $e^+e^{-}$ machines at the $\psi (3770)$ resonance, as well as at higher-mass resonances such as the $\psi (4040)$ or $\psi (4160)$ that can be used at BES-III. It is also a common end-product of the fragmentation of a c-quark at the LHC.

Much discussion has been sparked by a claim of evidence for direct CP violation in $D$ decays by the LHCb collaboration [971], and there has been ongoing discussion as to how large SM CP-violating effects can really be, given theoretical uncertainties in the long-distance physics which can enter [1684, 1685]. We can construct a CP asymmetry in the usual way:

5.41 $$\begin{aligned} A_\mathrm{CP}= \frac{\Gamma (D^0\rightarrow h^+h^{-})-\Gamma (\bar{D}^0\rightarrow h^+h^{-})}{\Gamma (D^0\rightarrow h^+h^{-})+\Gamma (\bar{D}^0\rightarrow h^+h^{-})} , \end{aligned}$$

from which the direct and indirect (via $D^0\leftrightarrow \bar{D}^0$ mixing) contributions can be separated, since both $\pi \pi$ and $KK$ channels are available. Thus we form the direct CP asymmetry $\Delta A_\mathrm{CP} = A_\mathrm{CP}(K^{+} K^{-}) - A_\mathrm{CP}(\pi ^{+} \pi ^{-})$, for which LHCb [971] reports $(-0.82\pm 0.21 \pm 0.11)~\%$, and the CDF result using the full Run II data set is comparable in size: $(-0.62\pm 0.21 \pm 0.10)~\%$ [1686]. An update of the earlier LHCb analysis using a much larger data set yields $\Delta A_\mathrm{CP} = (-0.34 \pm 0.15 \pm 0.10)~\%$ [1687], which is much smaller. Moreover, an independent LHCb measurement based on $D^0$’s from semileptonic $b$-hadron decays yields $(0.49 \pm 0.30 \pm 0.14)~\%$ [1688]. Thus the early evidence remains unconfirmed. The possibility of direct CP violation in the charm sector is of enduring interest [1689, 1690], however, and the search goes on.

*d. Observation of T violation in the* $B$ *system* In a separate development, an observation of direct T-violation has been claimed [1691]. Its presence is expected because the CPT theorem of local, Lorentz-invariant quantum field theory implies the existence of T violation in the presence of CP violation. Direct measurement of a fundamental T-violating effect in hadronic processes is a bit tricky, however, because it requires being able to compare an S-matrix element $S_{f,i}$ to its reciprocal $S_{i_\mathrm{T},f_\mathrm{T}}$ in which $i_\mathrm{T}$ and $f_\mathrm{T}$ are the time-reversed states of $i$ and $f$. It is challenging to prepare the requisite states, so that robust “detailed balance” tests of T are rare. A nonzero permanent EDM, of course, would display a fundamental violation of T-invariance in a stationary state, and experimental limits are becoming more stringent. The CPLEAR collaboration [1692] observed a difference in the rate of $K^0 \rightarrow \bar{K}^0$ and $\bar{K}^0 \rightarrow K^0$, where the initial $K^0$ ($\bar{K}^0$) is identified by its associated production with a $K^+$ ($K^{-}$) in $p \bar{p}$ collisions and the final-state $\bar{K}^0$ ($K^0$) is identified through the sign of the lepton charge in semileptonic decay. This has been questioned as a direct test of time-reversal violation [1693, 1694] because (i) the constructed asymmetry is independent of time and (ii) unitarity considerations reveal that if more $K^0$ goes to $\bar{K}^0$ than $\bar{K}^0$ goes to $K^0$ this can only occur if more $\bar{K}^0$ decays to $\pi \pi$ than $K^0$, making the appearance of particle decay (which is irreversible) essential to the effect. In regards to a detailed balance study of T in the B system, a theory proposal [1695] has been recently implemented by the BaBar collaboration [1691]; this is a much richer system than that studied by CPLEAR. The initial and final states are pairs of neutral $B$ mesons, be they in the flavor-eigenstate basis $B^0$, $\bar{B}^0$, or in the CP eigenstate basis $B^{\mathrm{CP}+}$, $B^{\mathrm{CP}-}$. The two reactions whose rates are compared are the neutral meson oscillations between states in the two different bases, e.g.,

5.42 $$\begin{aligned} \bar{B}^0\rightarrow B^{\mathrm{CP}-}\quad \quad \quad ; \quad \quad B^{\mathrm{CP}-}\rightarrow \bar{B}^{0}. \end{aligned}$$

**Fig. 43:**
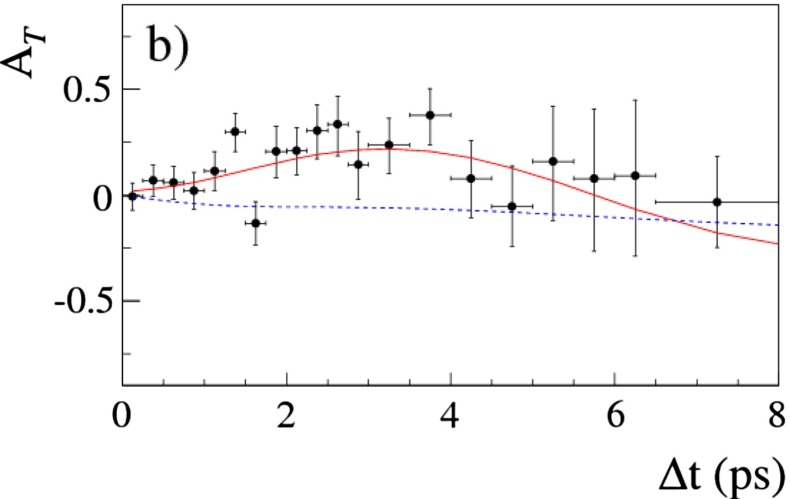
One of four T-violating asymmetries reported by the BaBar collaboration [1691]

To prepare the initial state, BaBar makes use of quantum entanglement in the reaction $e^{-}e^+\rightarrow \Upsilon (4S) \rightarrow B\bar{B}$. Because the intermediate vector $\Upsilon (4S)$ state has definite b-flavor (0) and CP ($+$), one chooses to make a measurement of either the CP or flavor of one of the two $B$-mesons, and this leaves its entangled $B$ partner in a CP or a flavor eigenstate. The partner of the tagged B is left to propagate and then the opposite measurement, of either flavor or CP, is made on the second $B$ meson. This second $B$ must have undergone the transition in (5.42) since it was produced as an eigenstate of either CP or flavor, but it is detected as an eigenstate of the other variable. The two reactions can at last be compared. It remains to be said what measurements reveal the CP content or the flavor content of the neutral $B$. The flavor of a $B$ meson can be tagged by the sign of the lepton charge in semileptonic decay, whereas its CP can be tagged by using $B\rightarrow J/\psi K_\mathrm{S}$ (CP$=-$) or $B\rightarrow J/\psi K_L$ (CP$=+$) decays, noting that direct CP violation in these decays is both $\mathcal{O}(\lambda ^2)$ and $\alpha _\mathrm{s}$ suppressed, note [1696] for an explicit estimate. An example outcome of the experiment is reproduced in Fig. 43. Splendidly, the use of entanglement allows both of the reservations [1693, 1694] levied against the CPLEAR experiment to be set to rest: $A_\mathrm{T}$ changes sign with that of $\Delta t$, and unitarity does not require particle decay to make $A_\mathrm{T}$ nonzero. Moreover, in these observables the T and CP transformations are distinct [1691]. Consequently, we conclude that BaBar has indeed observed direct T-violation in these reactions. The CP-violating asymmetry is of the same magnitude as the T-violating one, so that the outcome is compatible with CPT symmetry. This expectation can be broken by direct CP violation in the CP tag, i.e., through that in the $K$ decay [1697, 1698], and the cross check is compatible with the known smallness of such effects.

#### Rare decays

Rare decays of heavy mesons offer another class of useful “null” tests for BSM searches. The SM predictions tend to be exceeding small, and improving the experimental limits on their decay rates sharpens the constraints on models of physics BSM. We refer to Sect. 4 for a discussion of rare charm decays. In this section we focus on $B_q\rightarrow \mu ^+\mu ^{-}$ decay, with $q\in (d,s)$, because these decays are expected to occur at enhanced rates in the MSSM at large $\tan \beta$ [1699]. The decay $B_\mathrm{s} \rightarrow \mu ^{+} \mu ^{-}$ has recently been observed for the first time [1700, 1701], at a rate compatible with SM expectations. As reviewed in [1702], there are different ways to compute the $B_q\rightarrow \mu ^+\mu ^{-}$ decay rates within the SM, using distinct non-perturbative parameters computed in lattice QCD. For example, modern computations of the bag parameters $\hat{B}_\mathrm{s}=1.33 \pm 0.06$ and $\hat{B}_d=1.26 \pm 0.11$ [1703] serve to update the SM prediction for the $B_q \rightarrow \mu ^+\mu ^{-}$ branching ratios; specifically, $\mathcal{B}(B_q\rightarrow \mu ^+\mu ^{-}) = [\mathrm{known}\, \mathrm{factors}]/\hat{B}_q$. Using empirical values of the lifetimes and $\Delta \Gamma _\mathrm{s}=0.116\pm 0.019\,\mathrm{ps}^{-1}$, one gets [1702]

5.43 $$\begin{aligned} \mathcal{B}(B_\mathrm{s}\rightarrow \mu ^+\mu ^{-})&= (3.65\pm 0.20)\times 10^{-9} \,; \end{aligned}$$

5.44 $$\begin{aligned} \mathcal{B}(B_d\rightarrow \mu ^+\mu ^{-})&= (1.04\pm 0.09)\times 10^{-10} . \end{aligned}$$

We note that the alternate pathway uses the lattice-QCD meson decay constants $f_{B_q}$ and gives branching ratios which are in excellent agreement [1702]. The experimental values are [1700, 1701]

5.45 $$\begin{aligned} \mathcal{B}(B_d\rightarrow \mu ^+\mu ^{-})&< 0.81 \times 10^{-9}\,[90~\%\,\mathrm{C.L.}] \, ; \end{aligned}$$

5.46 $$\begin{aligned} \mathcal{B}(B_\mathrm{s}\rightarrow \mu ^+\mu ^{-})&= 3.2^{+1.5}_{-1.2} \times 10^{-9} , \end{aligned}$$

and the comparison with the SM expectations seems to leave little room for new physics. In particular, the MSSM at large $\tan \beta$ is quite constrained [1704]. Belle-II will hopefully be able to improve their experimental sensitivity to the extent that they can probe down to the SM level in both channels. Interestingly, the ratio

5.47 $$\begin{aligned} \frac{\mathcal{B}(B_\mathrm{s}\rightarrow \mu ^+\mu ^{-})\Delta m_d \tau _d \hat{B}_\mathrm{s}}{\mathcal{B}(B_d\rightarrow \mu ^+\mu ^{-})\Delta m_\mathrm{s} \tau _\mathrm{s} \hat{B}_d} \end{aligned}$$

still leaves room for significant new physics effects. The ATLAS collaboration [1705] is addressing this, employing as a benchmark the well-known $B\rightarrow J/\psi K$ decay as a reference in order to compute the branching fractions.

To conclude our discussion of flavor physics, we observe that all the quark flavor and CP violation currently observed in nature appears to be controlled by the CKM matrix [1367]. We have considered a broad sweep of low-energy observables, many of which are only statistics limited in their sensitivity, and for which theoretical uncertainties are under sufficient control to permit the discovery of departures from the CKM paradigm and indeed of physics BSM.

### Future directions

Popular models of physics BSM are becoming increasingly constrained through null results from direct searches at collider energies as well as from indirect searches realized from precision measurements at lower energies. This sweep of negative results nevertheless allows us to come to at least one positive conclusion, for we have established beyond doubt that the dominant mechanism of flavor and CP violation within the quark sector is due to the CKM matrix. CP violation may well exist in the neutrino sector as well, and with effort we should have the knowledge we need in regards to the interactions of neutrinos with matter in order to discover whether it does. We may also have discovered the mechanism by which elementary particles accrue mass, though it may take decades to establish whether the couplings of the Higgs are as predicted in the SM or not. Irrespective of this, and in contrast, continuing null (or contradictory) results in regards to particle dark matter yields no positive conclusion, for dark matter, and dark energy for that matter, have no explanation within the SM. Nevertheless, the astrophysical observations which led to their articulation are both robust and concrete. There is undoubtedly new physics to explain, and possibly an expansion of the SM that we can empirically establish to explain it.

It is entirely possible that the physics BSM for which we search will fit within the context of a model that we know. This means that the sweep of experiments we have considered are the right ones and that we need only be able to interpret experiments of enhanced sensitivity. We have offered a suite of experimental observables for which that is the case. In that class, there are, most transparently, various null tests, such as searches for permanent EDMs, or for neutrinoless double-beta decay. In the case of EDMs we have considered how robust non-perturbative methods in QCD, be their origin in lattice QCD or in effective field theory, can be used to interpret the experimental results in various systems if discoveries are ultimately made. In the case of neutron EDM matrix elements in lattice-QCD of nonleading dimension operators, the detailed methods are still under development. This theoretical control also extends to measurements of nonzero quantities to higher precision, such as that of the anomalous magnetic moment of the muon, or of the parameters of meson mixing, or of the neutral weak couplings of the quarks in PVES.

It is also possible that the explanations we seek will surprise us, that the BSM models of ultimate use have as yet to be invented. Since little in regards to dark matter is established, this is quite possible and supports broader thinking in regards to possible experiments [1366, 1706–1710]. Nevertheless, the non-perturbative tools we have discussed for the control of QCD will undoubtedly continue to play an important role.

Although we have illustrated through many examples in a sweep of contexts that lattice-QCD can play and has played a key role in the search for physics BSM, its utility has nevertheless been limited to particular classes of problems. That is, it has been restricted to systems for which the non-perturbative dynamics can be captured by the matrix elements of local operators (and typically of low operator dimension) and for which disconnected insertions, or quark loops, play a minimal role in the dynamics. Concretely, then, we have used lattice-QCD methods to greatest effect in the analysis of flavor-changing weak decays to leptonic and semileptonic final states. Let us then conclude with a perspective on the possibility of extending lattice-QCD methods to particle decays with nonleptonic final states [1711]. It is worth emphasizing that such a generalization would be key to the study of systems with enhanced, long-distance effects, such as $D{\bar{D}}$ mixing [1712], or the study of rescattering effects in hadronic $B$ (or $D$ or $K$) decays [1713, 1714]. Ultimately the limitations of lattice-QCD in this regard stem not from the use of discrete spacetime per se, but rather from a famous “no-go” theorem [393]: it is generally not possible to analytically continue a 3-point Green function computed in Euclidean space back to Minkowski space. A possible resolution to this puzzle relies on the structure of the S-matrix; e.g., the S-matrix and the energy-levels of two-particle systems at finite volume are closely tied [395]. An early application of these ideas was to systems with nearly elastic interactions in the final state [1715]. Systems with inelastic interactions are more interesting, however, and recently progress has been made to understand inelastic scattering in a finite volume [403, 1716, 1717]. Such are the first steps towards the complete analysis of nonleptonic decays (or of $D{\bar{D}}$ mixing) in QCD, and we relish such prospects.

## Deconfinement

17A robust prediction of Quantum Chromodynamics (QCD) is that at a certain value of temperature (or energy density), hadronic matter undergoes a transition to a deconfined state of quarks and gluons, known as the Quark–Gluon Plasma (QGP). By now, numerical simulations of lattice QCD have convincingly shown that this transition is in fact not a true phase transition but instead a rapid crossover that takes place at temperatures around 160 MeV. In the same temperature region, chiral symmetry is additionally restored up to a small explicit breaking due to nonzero quark masses. The physics of these two conceptually distinct but almost concurrent transitions has been the subject of intense activity in the theory community. The study of the transition region has subsequently been extended to nonzero baryon chemical potential $\mu _B$, corresponding to a nonzero average value of the net baryon density in the system. Increasing the chemical potential from zero, the transition may strengthen and eventually become a first-order phase transition, signaling the presence of a so-called critical point on the QCD phase diagram. An alternative scenario, potentially without a critical point, is that the crossover from hadronic to QGP matter becomes broader with $\mu _B$. The existence of a critical point would establish a remarkable universality link between QCD matter and condensed matter physics. Indeed, a prediction of universality is that many properties of quark matter near the critical point would be the same same as in a large class of condensed matter systems near their respective critical points.

Experimentally, heavy-ion collisions make it possible to study strongly interacting matter under extreme conditions in the laboratory. Several facilities contribute to understanding the details of the QCD phase transition, mapping out different regions of temperature and baryon chemical potential in the QCD phase diagram. At the top RHIC and LHC collider energies, the produced matter is characterized by very small net baryon densities and high temperatures, while future facilities at FAIR and NICA are planned to explore the phase diagram at high baryon chemical potential and lower temperature.

After the first experimental efforts in the 1970s at LBNL and JINR and intense theoretical and experimental research at different facilities and energies from GSI SIS to BNL AGS and CERN SPS, an assessment of the SPS program was presented in 2000 [1718, 1719]. The essence of the assessment, based on the results of half a dozen experiments at the SPS [1720–1722], was that a new state of matter was produced in the SPS energy regime, featuring some of the most important predicted characteristics of a QGP (thermalization, chiral symmetry restoration, deconfinement). The continuation of the heavy-ion program at RHIC at BNL [1723–1726] and at the CERN SPS [1727] confirmed and further refined the first SPS results. A comprehensive analysis of the first years’ data from all RHIC experiments (BRAHMS [1728], PHENIX [1729], PHOBOS [1730] and STAR [1731]) led to an assessment in 2005 [1723–1726, 1732] establishing the existence of the sQGP (where s stands for “strongly interacting”). The produced matter was found to behave like an extremely strongly interacting, almost perfect liquid with minimal shear viscosity, absorbing much of the energy of fast partons traversing it [1733, 1734]. After the discovery phase for the QGP and its qualitative characterization was well under way, the LHC [1735] took over with a primary objective of continuing and expanding the quantitative precision measurements begun at RHIC, taking advantage of the much increased energy and luminosity. First results [1736] came quickly, confirming the RHIC observations and exploring the properties of this new state of matter in the higher-energy regime. While ALICE [1737] was designed as a dedicated experiment to study typical heavy-ion observables [1738, 1739], all other LHC experiments, ATLAS [1740], CMS [1741] and LHCb [1742] also participate in the heavy-ion program, contributing to the detailed characterization of the produced matter (with LHCb taking part in the $p$–nucleus part of the program).

Detailed studies of the QGP produced in nuclear collisions at LHC and RHIC have already shown that this new state of matter has unique properties and presents challenging questions to theory [1743]. While theory has no complete answers yet, great advances have been made toward developing frameworks in which such questions can be addressed. Thus, experimental data can be used to clarify those properties of hot QCD matter that cannot yet be reliably predicted by QCD. A particular problem hindering the theoretical interpretation of the experimental results is the extremely rapid and complex dynamical evolution of the produced system. Typically, instead of a microscopic theory, effective descriptions are employed, ranging from relativistic hydrodynamics to Monte-Carlo transport simulations and simplified models.

Despite these challenges, the field is currently advancing towards a “standard model of heavy-ion collisions”. The initial collision of the two nuclei is thought to result in the formation of a dense, nonequilibrium QCD plasma which rapidly thermalizes. The expansion and cooling of the near-thermal QGP is described by hydrodynamics until thermal freeze-out produces a hadronic resonance gas. At this point, although the chemical composition of the produced particles is approximately fixed (chemical freeze-out), the spectral distributions still evolve until kinetic freeze-out. As a way to test the emerging qualitative picture, a number of experimental observables have been employed to probe the properties of the produced medium as well as the space-time evolution of the system. A non-exhaustive list of experimental observables, related to the properties of the QGP that we expect to determine from these studies, can be summarized as follows [1743]: (i) The equation of state of the produced matter is reflected in the spectra of the emitted particles and lattice QCD can reliably compute these quantities. (ii) Microscopic properties, such as the QGP transport coefficients, are related to the final-state flow pattern and the energy loss of high-$p_\mathrm{T}$ partons. Those include the shear viscosity, the coefficient ($\hat{q}$) governing the transverse momentum diffusion of a fast parton, the coefficient of linear energy loss, and the diffusion coefficient of a heavy quark in matter. Currently, lattice gauge theory cannot reliably calculate these dynamical quantities. (iii) The dissolution of bound states of heavy quarks in the QGP is governed by static color screening, which can be reliably calculated on the lattice. (iv) The electromagnetic response function of the QGP is reflected in the emission of thermal photons and lepton pairs. While it is difficult to calculate this dynamical quantity on the lattice, some progress has been made recently.

This interplay of theory and experiment, as well as the complementarity between different approaches, particularly essential for advances in the heavy-ion field, is reflected in this chapter. We review recent progress in the study of the deconfined phase of QCD, on both the theoretical [1743] and experimental sides [1744], pointing out current challenges and open questions. Thus we mostly present recent advances from the LHC era, not attempting a review of the field. The review of first results at LHC [1736], followed as a basis, is also a source of primary literature. The material was updated following the fast progress reported at major conferences, from QM2012 [1745] to more recent ones [1746]. In anticipation of new interesting results presented at QM2014 [1747], the reader is referred to the upcoming presentations and publications.

In this chapter, we concentrate on finite temperatures, leaving the case of cold and dense (nuclear) matter to Sect. 7. We begin by reviewing what is known about the equilibrium properties of the theory, in particular the part of the phase diagram explored by lattice QCD calculations in Sect. 6.1. In connection with the phase diagram, we describe the status of the Beam Energy Scan (BES) at RHIC and briefly touch upon “event-by-event” studies which employ fluctuations and correlations to search for critical behavior. From low transverse momentum particles, we can infer the bulk properties of the created matter and the dynamical evolution of the system. The main aspects of the hydrodynamic description of a near-thermal QGP are reviewed in Sect. 6.2 together with experimental results on the bulk properties and collective behavior of the system. Our current theoretical understanding of the different stages of the collision prior to the formation of the QGP is discussed in Sect. 6.3.1. Experimental results and our current theoretical understanding of the particle multiplicity and entropy production are discussed in Sect. 6.3.2. The high energies and luminosities of modern colliders, in particular the LHC, allow detailed studies of “hard probes”. These are produced by hard scatterings at early times during the initial stage of the collision ($t\sim 1/Q$) and can therefore be regarded as external probes of the nature and properties of the QGP. The current status of the theoretical and experimental efforts concerning these probes is reviewed in Sect. 6.4. We begin with an introduction to the theory of hard probes, starting with nuclear matter effects which provide the baseline for understanding the modification of these probes in hot matter. We provide brief theoretical overviews of energy loss in hot matter and of quarkonium suppression. We then turn to recent experimental results on high-$p_{T}$ particle and jet production as well as heavy-flavor production. Recent results on $p$A  collisions, studied in order to disentangle initial- from final-state effects, are discussed in Sect. 6.5. In addition to lattice QCD, which is best suited for the regime of low baryon density and static observables, theoretical frameworks have been developed to address the dynamical properties of the QGP. As an alternative to weak-coupling methods, strong-coupling calculations involving gauge/gravity duality have provided a different paradigm for the QGP studies at the temperatures explored in heavy-ion collisions. More generally, a number of effective field theories (EFTs) have been developed in the last decades to address different physical regimes and observables: Hard Thermal Loop (HTL) EFT, Electrostatic QCD (EQCD), Magnetostatic QCD (MQCD), Hard Thermal Loop NRQCD and Hard Thermal Loop (pNRQCD). They establish the link between perturbative calculations and strong-coupling calculations and allow precise definition and systematic calculation of quantities of great physical impact (such as the heavy quark–antiquark potential at finite temperature). In Sect. 6.6, we compare and contrast several results for bulk thermodynamics and transport quantities computed within these frameworks. In Sect. 6.7, we discuss recent progress in thermal field theory calculations in the context of hot matter in the early universe—a closely related area where progress is often directly tied to advances in heavy-ion physics. Finally, in Sect. 6.8 we present experimental results on the chiral magnetic effect, while a theoretical review of this phenomenon is given in Sect. 6.8. We end with a discussion of open questions and future directions for the field in Sect. 6.9.

### Mapping the QCD phase diagram

The QCD phase diagram as a function of temperature $T$ and baryon chemical potential $\mu _{B}$ is expected to have a rich structure. In this section, we discuss the bulk properties of quark matter in the region of small to moderate baryon chemical potential, $0\le \mu _B\lesssim 1\,\mathrm{GeV}$, which can be explored experimentally in heavy-ion collisions. In particular, lattice QCD allows for first principles calculations of equilibrium quantities at $\mu _B=0$. To extend these studies to moderate values of the baryon chemical potential, $\mu _B \lesssim 3T$, various methods have been recently used  [1748]. For the phase structure at higher baryon densities see Sects. 6.1.2 and 7.

The equation of state is an important input in the hydrodynamic calculations that have been successful in describing the evolution of the expanding matter created in relativistic heavy-ion collisions (see Sect. 6.2). Quantifying the equation of state and the associated quark number susceptibilities [1749, 1750] below the transition temperature is important for testing the freeze-out mechanism and the Hadron Resonance Gas [1751] description of hadronic matter up to the crossover temperature. The cumulants of the quark number distributions also provide information about the presence of a critical point in the QCD phase diagram if a sufficient number of them are known [1749, 1750]; see Sect. 6.1.2.

While educated guesses as to the qualitative behavior of the equation of state have been around for a long time, it has been determined with precision on the lattice [1752] only in the last 5 years. At low temperatures, the matter can be described in terms of a dilute hadron gas. The passage from a bulk hadronic state at low temperatures to a quark–gluon plasma phase at high temperatures was found to be an analytic crossover in lattice QCD calculations [1753]. A rapid rise of the entropy density, $s$, occurs around a temperature of ${\sim } 160$ MeV [1752]. This can be interpreted as a transition to partonic degrees of freedom. Above 400 MeV, $s/T^3$ has weak temperature dependence and is expected to reach the Stefan–Boltzmann limit at asymptotically high temperatures. The fact that lattice data are still below the ideal gas limit is an indication that interactions are still important at high $p_\mathrm{T}$. Agreement with the perturbative equation of state has been established at high temperatures in the (numerically less demanding) pure gluon plasma [1754]. Some recent results on the equation of state and the quark number susceptibilities are discussed below in Sect. 6.1.1. We refer the reader to [1755] for a more complete introduction to finite-temperature lattice calculations.

At vanishing baryon chemical potential, the integrand in the standard path integral expression for the QCD partition function is real and positive once the quark fields are integrated out analytically. This integrand can therefore be interpreted as a probability distribution for the gluon fields. The high-dimensional integral can then be estimated by importance-sampling Monte-Carlo methods: the gluon fields are sampled in such a way that the probability of occurrence of a field configuration is proportional to the value of the integrand evaluated on that configuration. When nonzero baryon chemical potential is introduced on the lattice, the integrand becomes complex. In this case, Monte-Carlo methods based on the importance sampling of field configurations no longer apply. The phase of the integrand can be absorbed into the observables, but its fluctuations from configuration to configuration lead to uncontrollably large cancellations. This numerical challenge is known as the “sign” problem. It is only recently that ways of overcoming this difficulty have been developed, including overlap-improving multi-parameter reweighting [1756–1758], Taylor expansion [1759] and analytic continuation from imaginary to real chemical potential [1760]. While the transition initially exhibits little sensitivity to the baryon chemical potential, some of these calculations suggest that the phase transition is no longer a crossover beyond a certain critical value of $\mu _B$, but instead becomes a first-order transition (Sect. 6.1.2). There is strong experimental interest in discovering this critical point. Recent studies are described in Sect. 6.1.3.

#### Precision lattice QCD calculations at finite temperature

In precision lattice QCD calculations, two aspects are particularly important. First of all, physical quark masses should be used. While it is relatively easy to reach the physical value of the strange quark mass, $m_\mathrm{s}$, in present day lattice simulations, it is much more difficult to work with physical up and down quark masses $m_{u,d}$, because they are much smaller: $m_\mathrm{s}/m_{u,d} \approx 28$ (Sect. 3). In calculations with $m_\mathrm{s}/m_{u,d} < 28$, the strange quark mass is usually tuned to its approximate physical value while the average up and down quark masses are larger than their physical values. Second, the characteristics of the thermal transition are known to suffer from discretization errors [1752, 1761]. The only way to eliminate these errors is to take smaller and smaller lattice spacings and systematically extrapolate to vanishing lattice spacing (and thus to the continuum limit). It is computationally very demanding to fulfill both conditions. There are only a few cases for which this has been achieved. Within the staggered formalism of lattice QCD (see for instance [1755] for a description of different lattice fermion actions), there are full results on quantities such as the nature of the transition [1753], the transition temperature for vanishing and small chemical potential [1136, 1762–1764], the equation of state [1752] and fluctuations [1749, 1750].

**Fig. 44:**
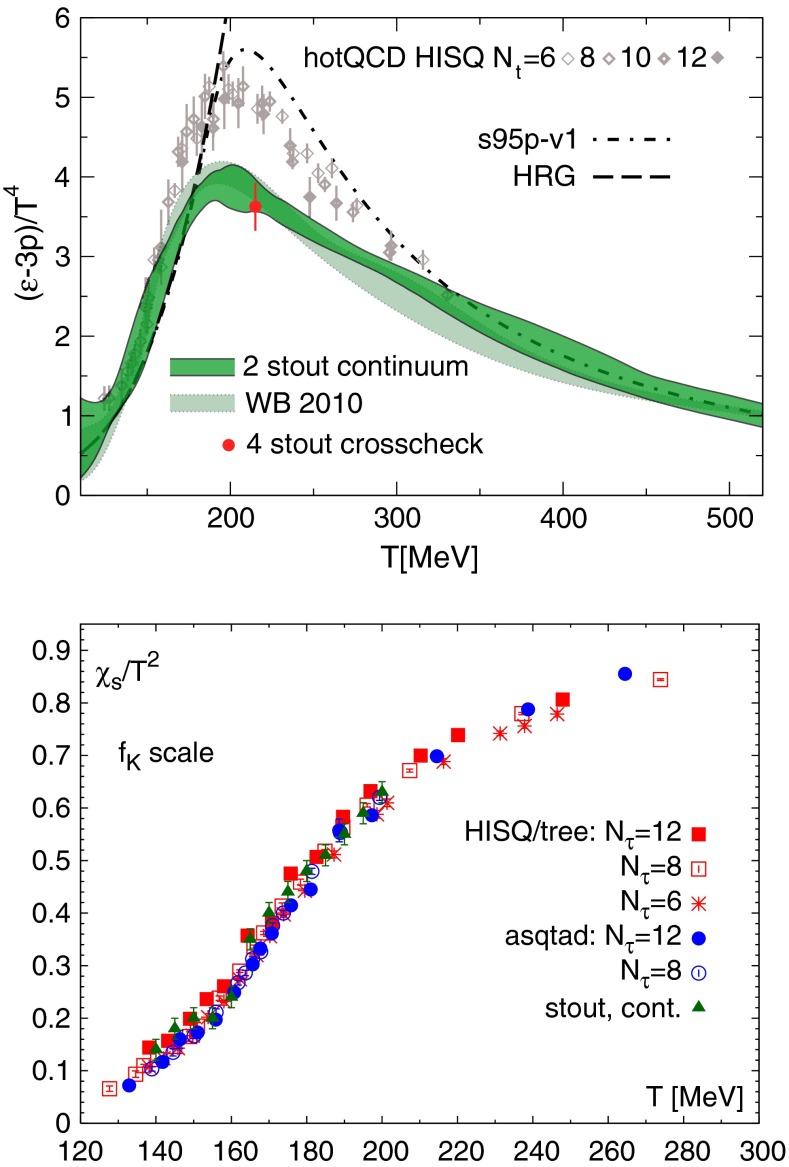
*Top* Comparison of the equations of state obtained by the Wuppertal-Budapest group (*shaded region*) and the HotQCD Collaboration (*points*). There is still a sizable discrepancy between the results. From [1768]. *Bottom* The strange quark susceptibilities calculated by the two groups. In the continuum limit, the results agree. From [1136]

*a. Status of the equation of state* The first step in obtaining any trustworthy result in QCD thermodynamics is to determine the temperature of the QCD transition. Its value was under debate for some years, but it is a great success for the field of lattice QCD that the results from two independent groups using different lattice discretizations now completely agree [1136, 1762, 1763]. Since the transition is a crossover, the precise value of the transition temperature depends on the chosen definition, but a typical value based on the chiral condensate and the associated susceptibility is 155 MeV with a (combined statistical and systematic) uncertainty of ${\sim } 3 ~\%$. The next important step is the determination of the equation of state. There are various calculations with different fermion formulations, see Ref. [1765] for a calculation using Wilson fermions. The current most precise results have been obtained with staggered quarks. In these calculations, the light and strange quark masses take their (approximate) physical values. There is still a discrepancy in the equation of state in the literature. The Wuppertal-Budapest group obtained [1766] a peak value of the trace anomaly at $(\epsilon -3p)/T^4 \sim 4$, confirmed later in  [1752]. The HotQCD Collaboration typically finds higher values for the peak value of the trace anomaly, see Ref. [1767]. The top panel of Fig. 44 compares the results from the two groups. Still more work is needed to clarify the source of the difference.

*b. Susceptibilities from lattice QCD* Fluctuations and correlations of conserved charges are important probes of various aspects of deconfinement. This is because fluctuations of conserved charges are sensitive to the underlying degrees of freedom which could be hadronic (in the low-temperature phase) or partonic (in the high-temperature phase). Fluctuations of conserved charges have primarily been studied using different staggered actions. The two most complete calculations have been carried out by the Wuppertal-Budapest group and by the HotQCD Collaboration [1749, 1750, 1769, 1770]. The bottom panel of Fig. 44 compares results on the strange quark number susceptibility.

The fluctuations are small at low temperatures because strangeness is carried by massive strange hadrons (primarily by kaons). This region is described by the Hadron Resonance Gas model [1751]. Strangeness fluctuations rise sharply through the transition region, as the strange quarks are no longer bound. At the highest temperatures shown, the susceptibility approaches unity.

The strange quark susceptibility has been determined to high precision. Other quantities and, in particular, higher cumulants are under investigation by many lattice groups. High quality results are expected in the near future.

**Fig. 45:**
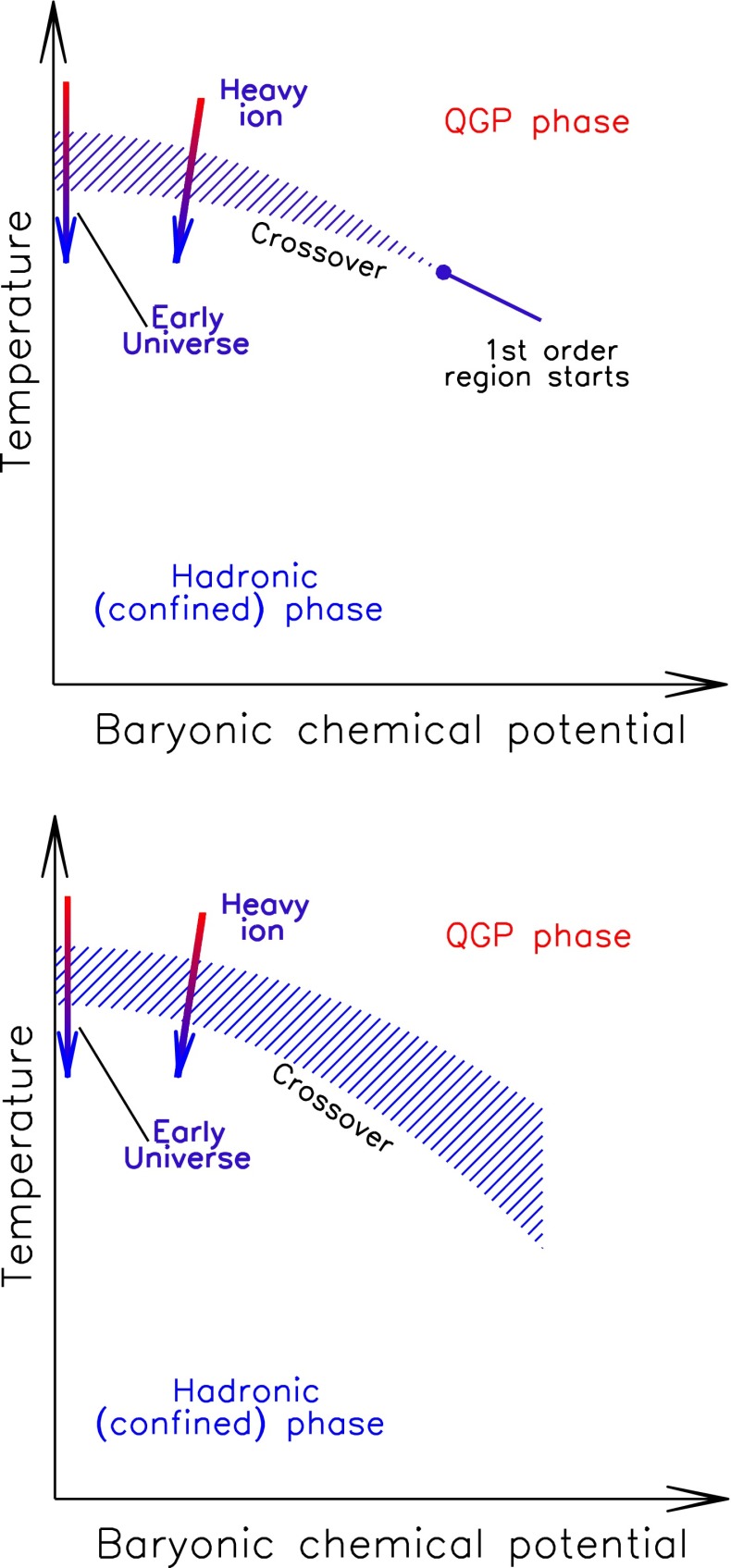
Two scenarios for the phase diagram of QCD for small to moderate baryon chemical potential $\mu _B$. In the *upper panel*, the phase diagram contains a critical point in this region, while in the *bottom* it does not. From [1764]

#### A critical point in the QCD phase diagram?

A number of interesting properties of QCD matter follow from the assumption that a critical point exists in the phase diagram. Two scenarios for the phase diagram of QCD for small to moderate baryon chemical potential $\mu _B$ are presented in Fig. 45. In the first, the phase diagram contains a critical point in this region, while in the second it does not. The critical point is the end of a line of a first-order transition and, as such, is similar to the critical point in the water-vapor system. The universality class is the three-dimensional Ising model class, Z(2). The dynamic universality class is that of model ‘H’ of the classification [1771], corresponding to the liquid-gas phase transition [1772].

Does the critical point exist? There is no firm answer yet from the theory side. Chiral models remain inconclusive; for a recent discussion, see [1773] and references therein. Two kinds of lattice results speak in favor of it. One is the reweighting result [1774], obtained on a coarse lattice. The other is the Taylor expansion of the pressure [1775, 1776]. When all Taylor coefficients have the same sign, a radius of convergence can be estimated which gives the location of the critical point. However a large number of terms are needed to convincingly establish the existence of the critical point [1777, 1778].

What speaks against a critical point relatively close to the $\mu _B=0$ axis is the study of the width of the transition region as a function of $\mu _B$ using a Taylor series around $\mu _B=0$ [1764]. It shows that the width is initially practically independent of $\mu _B$. This result goes in the same direction as the study of de Forcrand and Philipsen [1779, 1780], who tracked the chiral critical surface in the parameter space of light and strange quark masses and the chemical potential, $(m_l,m_\mathrm{s},\mu _B)$. A point on the surface corresponds to a set of parameters for which the thermal phase transition is second order. In the plane $\mu _B=0$, a critical line separates the origin (where the transition is first order) from the point of physical quark masses (where the transition is a crossover). At small $\mu _B$ they showed that the critical surface recedes away from the point $(m_l^\mathrm{phys},m_\mathrm{s}^\mathrm{phys},\mu _B)$ indicating that at physical quark masses the transition becomes weaker upon switching on a small chemical potential. It is not excluded however that the chiral critical surface $(m_l^\mathrm{crit}(\mu _B),m_\mathrm{s}^\mathrm{crit}(\mu _B),\mu _B)$ bends over again. The critical point would be given by the conditions $m_{l,s}^\mathrm{crit}(\mu _B^\mathrm{crit}) = m_{l,s}^\mathrm{phys}$. These results both suggest that, if a critical point exists, it lies beyond about $\mu _B\simeq 500\,\mathrm{MeV}$ [1748, 1764].

#### Experimental exploration of the QCD phase diagram

By varying $\sqrt{s_{NN}}$ in heavy-ion reactions, experiments can scan a large region of the phase diagram. The systems created at different values of $\sqrt{s_{NN}}$ have different trajectories in the $T-\mu _{B}$ plane and may pass through the critical point. There have been two experimental programs so far to search for the critical point and signatures of a phase transition. Both programs employ an energy scan over a region of relatively low center of mass energies.

The first such systematic study was performed within the CERN SPS beam energy scan program between 1998 and 2002. This scan, covering five values of $E_\mathrm{beam}$, was primarily undertaken by the NA49 [1781] experiment with participation from NA45 and NA57 [1782]. This program is currently being extended by NA49’s successor, NA61. After finishing the $pp$ and Be+Be measurements, data will be taken with the larger systems Ar+Ca and Xe+La. The second program, currently active, is the beam energy scan program at RHIC. The STAR collaboration [1731] is, as described below, taking data over a similar $\sqrt{s_{NN}}$ range as that of the SPS. As a collider experiment, STAR has the advantage that its acceptance around midrapidity does not depend on $\sqrt{s_{NN}}$. The PHENIX collaboration has placed its emphasis on higher energies, $\sqrt{s_{NN}} \ge 39$ GeV [1729].

The NA49 experiment at the SPS carried out, in fixed-target mode, the first beam energy scan at energies ranging from $\sqrt{s_{NN}} = 17.2$ GeV down to $6.2$ GeV [1783–1788]. The NA49 collaboration has published various inclusive measurements which they have interpreted as hinting at the onset of deconfinement near $\sqrt{s_{NN}} = 7.7$ GeV. These measurements include, among others, the “horn” effect, which is a local peak in the $K/\pi$ ratio as a function of $E_\mathrm{beam}$, and the “dale” phenomenon, which is a minimum in the width of the pion rapidity density, compared to a reference expectation, as a function of $E_\mathrm{beam}$ [1783–1788]. This SPS-based program will be taken over by NA61 experiment.

Establishing whether or not a critical point exists is a top priority. The divergence of susceptibilities of conserved quantities such as baryon number, charge, and strangeness at the critical point translate into critical fluctuations in the multiplicity distributions and can be studied experimentally [1789, 1790]. Generally speaking, one is looking for a qualitative change in these observables as a function of baryon chemical potential $\mu _B$. Therefore, experimental studies focus on the behavior of multiplicity fluctuation-related observables in small steps of beam energy. At first, experimental investigations were limited to the second moments of multiplicity distributions, which are proportional to the square of the correlation length $\xi$ [1789]. In heavy-ion collisions, the latter is estimated to be small, $\sim$ 2–3 fm [1791], in the vicinity of a critical point. Therefore, the higher moments of event-by-event multiplicity distributions are preferred; the higher the order of the moment, the more sensitive it is to the correlation length of the system, e.g., the third moment (skewness) $S\sim \xi {^{4.5}}$ and the fourth moment (kurtosis) $\kappa {^2}\sim \xi {^7}$ [1789]. Measurements of higher moments of event-by-event identified-particle multiplicity distributions, and their variation with centrality and beam energy, provide a direct link between experimental observables and lattice QCD calculations.

The exploratory phase, Phase I, of the Beam Energy Scan (BES) program at RHIC was completed in 2011, with data taken at $\sqrt{s_{NN}} = 39$, 27, 19.6, 11.5 and 7.7 GeV. All data taken by the STAR detector below the RHIC injection energy ${\sim } 20$ GeV are affected by large statistical errors, increasing steeply with decreasing energy. Together with larger data sets at 62, 130 and 200 GeV, these measurements provided an initial look into the uncharted territory of the QCD phase diagram.

The BES program goals [1792] are focused on three areas. The first, and least complicated, is a scan of the phase diagram at different $\sqrt{s_{NN}}$ to vary the values of $\mu _B$ and $T$ to determine at which energy (if any) the key QGP signatures reported at the highest RHIC energies [1725, 1726] are no longer observed. The disappearance of a single QGP signature as the energy is decreased would not be convincing evidence that the border between confinement and deconfinement has been reached at that energy since other phenomena, unrelated to deconfinement, could result in similar effects, or else the sensitivity to the particular signature could be reduced at lower energies. However, the modification or disappearance of several signatures simultaneously would constitute a more compelling case.

A second goal is the search for critical fluctuations, e.g., measured in net-proton multiplicity distributions, associated with a strong increase in various susceptibilities, expected in the vicinity of a critical endpoint. However, finite size effects tend to wash out this critical behavior, making it difficult to predict the signatures of the critical fluctuations quantitatively.

A third proposed goal is to find evidence of the softening of the equation of state as the system enters a mixed phase (such as a speed of sound in medium well below the ideal $1/\sqrt{3}$). Promising observables in this search include the directed flow $v_1$ and elliptic flow $v_2$ (i.e., the first and second Fourier coefficients for the azimuthal anisotropy relative to the reaction plane; see Sect. 6.2.2 for a more complete discussion), and these flow measurements are for charged particles as well as identified protons, net protons, and pions. Other relevant measurements are azimuthally sensitive particle correlations.

The STAR BES Phase I results discussed below [1793–1796] have made it possible to close in on some of the goals outlined above. It is very encouraging that the performance of both the collider and the experiments was excellent throughout the entire energy range explored to date. Phase I Energy Scan data allowed STAR to extend the $\mu _B$ range of RHIC from a few tens of MeV up to ${\sim } 400$ MeV. The critical region in $\mu _B$ has been predicted to span an interval of 50 to 100 MeV [1775, 1797–1802].

As to the first goal, the violation of constituent quark number scaling and the disappearance of high $p_\mathrm{T}$ hadron suppression [1793–1796] suggest that hadronic interactions dominate over partonic interactions when the collision energy is decreased below the measured energy point at $\sqrt{s_{NN}} = 11.5$ GeV.

**Fig. 46:**
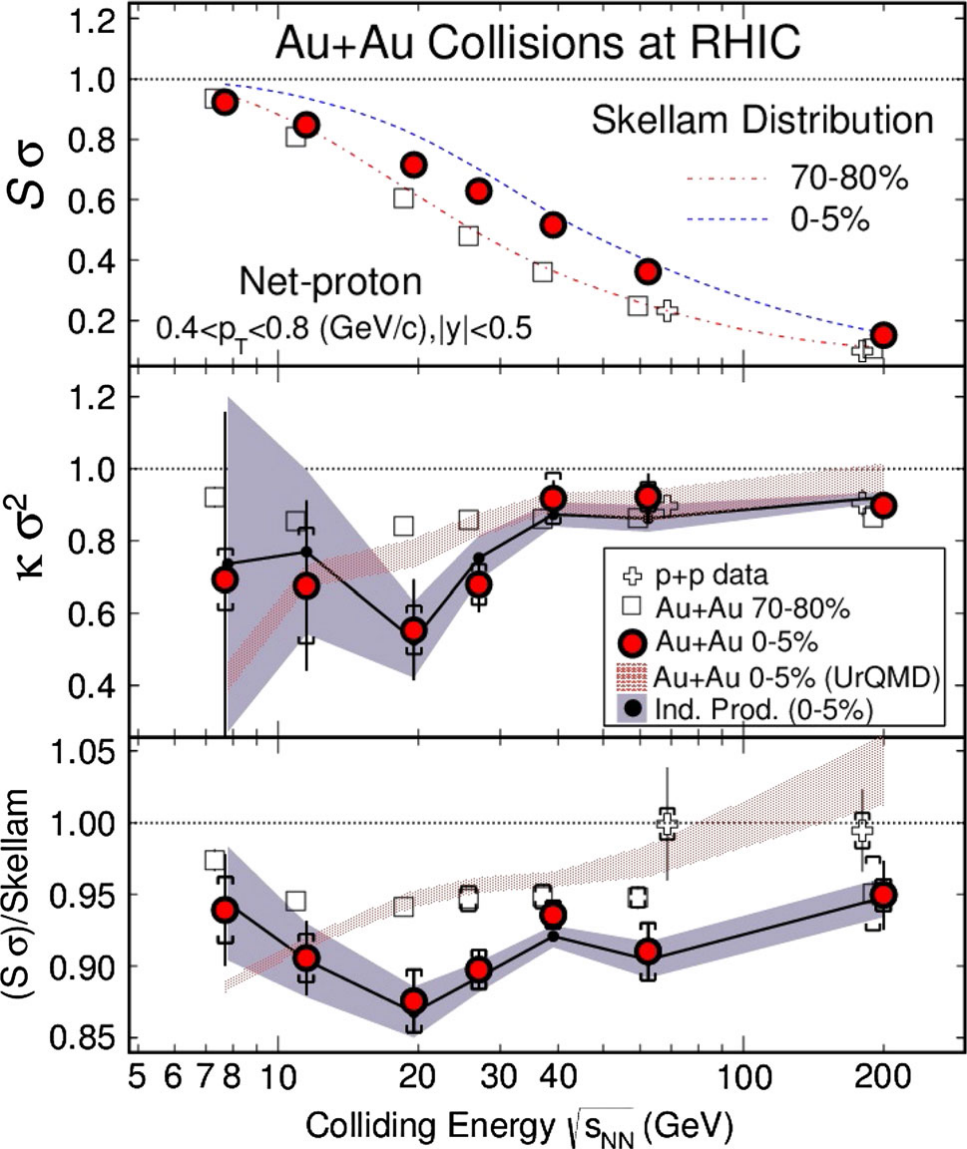
STAR’s measurements of $\kappa \sigma ^2$, $S\sigma$ and $S\sigma /$Skellam as a function of beam energy, at two different centralities. A Skellam distribution is the difference between two independent Poisson distributions [1803]. Results from $p$+$p$ collisions are also shown. One *shaded band* is an expectation based on assuming independent proton and antiproton production, and the other *shaded band* is based on the UrQMD model. From [1803]

In order to address the second goal, higher moments of the net-proton distribution (a proxy for net-baryon number) are considered to be the best suited observables in the search for a critical point. Figure 46 demonstrates that the measurements from BES Phase I do not deviate from expectations based on assuming independent production of protons and antiprotons [1803]. However, there is a considerable gap in $\mu _B$, of the order of 110 MeV, between the beam energy points at 11.5 GeV and 19.6 GeV. Based on common estimates of the extent of the critical region in $\mu _B$, which could well be of the same order, it is a valid concern that BES Phase I could have missed it. Therefore, at the beginning of 2014, the STAR collaboration started to run Au+Au collisions at 14.6 GeV.

In terms of the third goal, the first signals of possible softening of the equation of state were also observed. In particular the directed flow of protons and net protons within $7.7 < \sqrt{s_{NN}} < 200$ GeV [1793–1796] bears a striking similarity to hydrodynamic simulations with a first-order phase transition [1804]. The implications of these measurements for understanding the QCD phase structure are however not yet resolved.

The statistics collected during Phase I of BES are insufficient for final conclusions on the program goals. Therefore, STAR proposed precision measurements in Phase II to map out the QCD phase diagram with an order of magnitude increase in statistics, planned around 2018 and 2019.

There is also a plan to run STAR in fixed-target mode concurrently with collider mode during BES Phase II. With a fixed-target program in STAR, the range of accessible values of baryon chemical potential could be extended from $\mu _{B} \sim 400$ MeV up to ${\sim } 800$ MeV at $\sqrt{s_{NN}} \sim 2.5$ GeV.

This wide-ranging experimental effort must be accompanied by advances in theory. The detailed evolution of the matter produced at RHIC, and its transformation from hadronic to partonic degrees of freedom and back again, are not understood. Simulations employing models with and without a phase transition as well as with and without a critical point over the BES range are important to guide the experimental program and interpret the results. For example, it is necessary to know whether or not STAR net-proton directed flow measurements at BES energies can be explained by hadron physics only. While there is no qualitatively viable hadronic explanation based on current models, tighter scrutiny is needed to convincingly exclude such a description. Therefore, more predictions of measurable observables related to the location of the critical point and/or phase boundaries should be made. In particular, the behavior of observables in simulations that incorporate a first-order phase transition needs further study. For example, a mean-field potential can be constructed to implement a first-order phase transition in transport models. Overall, significant progress has been made up to this point, but the additional detailed data expected from BES Phase II will be essential for completing the program goals, while parallel theoretical progress will be equally vital.

### Near-equilibrium properties of the QGP

#### Global event characterization

In ultrarelativistic heavy-ion collisions, the majority of the produced particles are emitted with transverse momenta below a few GeV/$c$. Precision studies of their production characterizes the dynamic evolution of the bulk matter created in the collision. Measurements of the multiplicity distribution are related to the initial energy density. Identified particle yields and spectra reflect the conditions at and shortly after hadronization. The space-time evolution of the particle-emitting source and its transport properties are accessible experimentally through particle correlations. In this section, we briefly describe some of the relevant observables and recent results. The experimental overview presented in this section is largely based on the review by Müller, Schukraft and Wyslouch [1736], which summarized the first results of Pb+Pb data taking at the LHC and extends it with the latest findings based on increased statistics and more refined analyses.

**Fig. 47:**
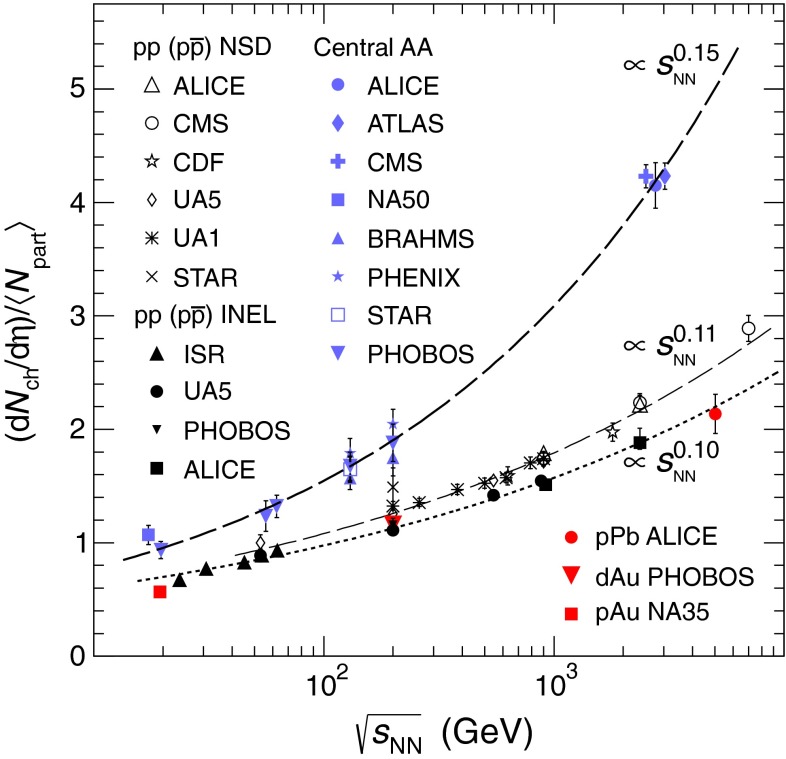
Charged particle pseudorapidity density at midrapidity, $dN_\mathrm{ch}/d\eta$, per participant as a function of $\sqrt{s_{NN}}$ for $pp$, $pA$ and $AA$. From [1805]

*a. Multiplicity* Particle production at low $p_\mathrm{T}$ cannot be calculated from first principles with currently available theoretical tools. Despite the availability of RHIC data before the LHC startup, the predictions for particle multiplicities varied widely. Figure 47 presents a summary of the charged particle pseudorapidity density per participant measured in $pp$, $pA$ and $AA$ collisions [1805, 1806]. While the energy dependence of $dN_{\mathrm{ch}}/d\eta$ in non-single diffractive (NSD) $pp$ and $pA$ collisions follows a power law, $s_{NN}^\alpha$ with $\alpha = 0.1$, the $AA$ data show a much steeper dependence that can be best described with $\alpha = 0.15$.

This behavior underlines the fundamental differences of bulk particle production in $AA$ with respect to $pp$ and $pA$ collisions and provides an essential constraint for models, see Sect. 6.5. A comparison between data and theoretical models can be found in [1736, 1807]. In addition, the multiplicity distribution in $AA$ collisions has also been studied employing holographic approaches, as discussed in Sect. 6.3.2.

*b. Energy density* The measured $dN_\mathrm{ch}/d\eta$ can be related to the initial energy density of the system using the Bjorken hydrodynamic model [1808], based on a longitudinal, isentropic expansion. The energy density reached in the initial stage ($\tau _{0}=1$ fm/$c$) of a central Pb+Pb collision at the LHC of about $\epsilon = 15$ GeV/fm$^3$ [1809] is almost three times higher than the one reported at RHIC [1723–1726, 1810] and well above the critical energy density required for the predicted phase transition to a deconfined state of quarks and gluons of about 0.7 GeV/fm$^3$ [1811].

**Fig. 48:**
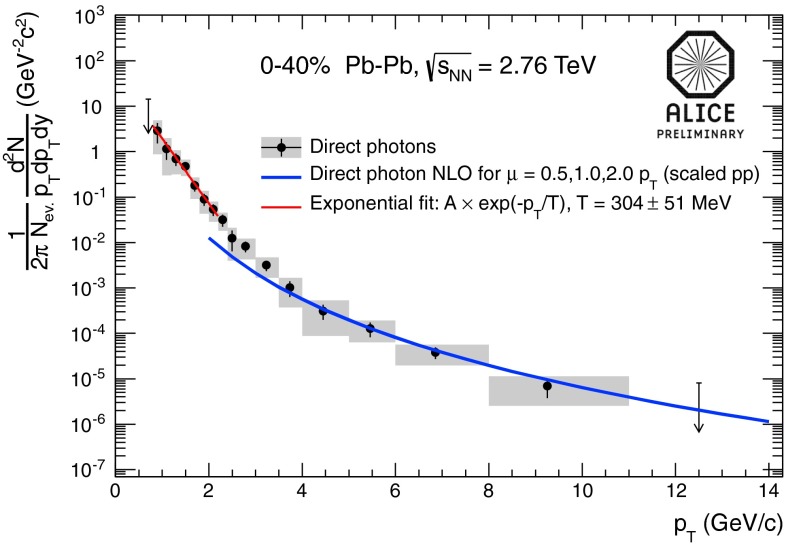
The direct photon $p_{T}$ spectrum with the NLO prediction at high $p_{T}$ and an exponential fit at low $p_{T}$. From [1812]

*c. Initial temperature* This relative increase of energy density from RHIC to the LHC implies a corresponding initial temperature at the LHC of $\approx 300$ MeV for central Pb+Pb collisions. An experimental access to this temperature is given by the measurement of thermal photons, emitted in the initial stage of the collision. The $p_{T}$ spectrum of direct photons, measured by the ALICE Collaboration using $\gamma$ conversions in the 40$~\%$ most central Pb+Pb collisions [1812], is shown in Fig. 48. The spectrum is reproduced by the NLO pQCD prediction for $pp$ collisions, scaled by the number of binary collisions at $p_{T}$ $>4$ GeV/$c$. Below 2 GeV/$c$ there is an excess attributed to thermal photons. An exponential fit in the range $0.8<p_\mathrm{T}<2.2$ GeV/$c$ yields an inverse slope parameter $T=(304 \pm 51$) MeV. The quoted uncertainties include both statistical and systematic uncertainties. The LHC value of this effective temperature is about 40$~\%$ higher than that measured in a similar analysis by PHENIX [1813] and is clearly above the expected phase transition temperature of about 160 MeV. Before firm conclusions can be drawn from these measurements, two important considerations have to be taken into account. First, the measurement of the thermal photon spectrum is experimentally very demanding [1814]. Despite the impressive precision already achieved [1815], further refined analyses are expected in the future. In particular, a more precise estimation of the detector material budget, needed for the determination of the photon conversion probability, are expected to further reduce the experimental uncertainties. Furthermore, the thermal photon spectrum is obtained by subtracting the decay photon spectrum which is obtained by a complicated cocktail calculation. While the contribution from the $\pi ^{0} \rightarrow \gamma \gamma$ decay can be based on the measured spectrum, the contribution from unmeasured meson yields in Pb+Pb collisions (such as $\eta$, $\eta '$, $~\omega$, $~\rho ^{0}$) have to be interpolated from $m_{T}$-scaling. Second, more rigorous theoretical analyses of the ALICE data [1816] are ongoing, which also include Doppler blue-shift corrections of the temperature due to the radially expanding medium [1817].

*d. System size and lifetime* The space-time evolution of the expanding system is studied using identical pion interferometry techniques known as Hanbury-Brown Twiss (HBT) correlations [1818]. At LHC energies, the measurement in the 5$~\%$ most central Pb+Pb collisions shows that the homogeneity volume at freezeout (when strong interactions cease) is 5000 fm$^3$, twice as large as the volume measured at RHIC. The total lifetime of the system (the time between the initial nucleon–nucleon collisions and freezeout) is approximately 10 fm/$c$, 30$~\%$ larger than at RHIC [1819]. The extracted volume increases linearly as a function of charged particle multiplicity. Extrapolation to $dN_\mathrm{ch}/d\eta$ shows that, in this limit, the system size coincides with the initial volume of a Pb nucleus and its lifetime vanishes [1736]. Hydrodynamic models correctly describe the evolution with center-of-mass energy from RHIC to LHC as well as the dependence of the individual radius parameters on the pair momentum, which is sensitive to radial flow [1820–1822]. Measurements with kaons and protons are being carried out to test whether the collective motion includes heavier mesons and baryons. In addition, baryon correlations are sensitive to mutual strong interactions, which are poorly known, especially for baryon-antibaryon pairs. The parameters of this interaction can be deduced from two-particle baryon correlations [1823, 1824], which constitute a powerful way to obtain such information.

As a reference the same measurement was carried out in smaller systems ($pp$, $pA$). Particular attention was given to high multiplicity events where collectivity was predicted to arise in some models [1825, 1826]. The extraction of femtoscopic radii in such systems is complicated due to the presence of other correlation sources, i.e., mini-jets and energy and momentum conservation. Monte-Carlo models have to be used to account for these effects [1827]. Other methods, such as three-pion correlations, are by construction less sensitive to such background, due to the usage of higher-order cumulants [1828]. The analysis shows that the radii in small systems depend on multiplicity and pair momentum but not on collision energy. The overall magnitude is smaller than in collisions of heavy ions at comparable multiplicity. Although decrease of radii with pair momentum is observed it is of different nature as compared to heavy-ion collisions. Therefore qualitatively new features are observed in HBT of small systems, that still require theoretical investigation.

*e. Particle spectra in different* $p_{T}$ *ranges* Transverse momentum spectra are sensitive to different underlying physics processes in different $p_{T}$ domains. In a crude classification, three separate regions can be identified: low, intermediate and high $p_{T}$. At $p_{T} < 2$ GeV/$c$, the bulk matter dynamics can be described by relativistic hydrodynamic models. Even at LHC energies, more than 95$~\%$ of all particles are produced within this $p_{T}$ range. While the spectral shape reflects the conditions at kinetic freezeout (where particle momenta are fixed), the integrated particle yields reflect the conditions at chemical freeze-out (where particle abundances are fixed). At $p_{T} > 8$ GeV/$c$, partons from hard scatterings interacting with the medium dominate the spectrum. At intermediate $p_{T}$, the data reflect an interplay of soft and hard processes. The energies available at the LHC open up the possibility for detailed measurements over an extended $p_{T}$ range, up to hundreds of GeV$/c$ in some cases. Understanding the interplay of soft and hard processes and the onset of hard processes remains a theoretical challenge. We discuss some low and intermediate $p_{T}$ results in the remainder of this section. Hard processes are discussed in Sect. 6.4.

*f. Low* $p_{T}$ The spectra of identified charged hadrons ($\pi$, $K$ and $p$), measured in the 5$~\%$ most central Pb+Pb collisions at the LHC [1829] for $0.1 <$ $p_{T}$ $< 4.5$ GeV/$c$ and at midrapidity, $|y| < 0.5$, are harder than the ones measured in central Au+Au collisions at $\sqrt{s_{NN}} = 200$ GeV at RHIC [1830, 1831], reflecting the stronger radial flow at the LHC. A blast-wave fit of the spectra [1832] yields a kinetic freeze-out temperature $T_{kin} = 96 \pm 10$ MeV, similar to the one at RHIC, and a collective radial flow velocity, $\langle \beta _{T} \rangle = 0.65 \pm 0.02$, 10$~\%$ higher than the one at RHIC. When compared to hydrodynamic calculations [1822, 1833–1836], the data are in better agreement with calculations including rescattering during the hadronic phase. Similar behavior is observed in other centrality classes [1837].

The conditions at chemical freeze-out, where particle abundances are fixed, are characterized by the chemical freeze-out temperature ($T_{\mathrm{ch}}$) and baryochemical potential ($\mu _{B}$) and are determined from measured particle yields in thermal model calculations. Recent comparisons of the ALICE measurements in central Pb+Pb collisions with thermal models [1838, 1839] show the best agreement between data and theory calculations at vanishing baryochemical potential, $\mu _{B}\approx 1$ MeV, and at a chemical freeze-out temperature of $T_{\mathrm{ch}} \approx 156$ MeV, lower than the value $T_{\mathrm{ch}} \approx 164$ MeV predicted before the LHC startup. This difference was caused by an overestimate of the proton yield in the model for higher chemical freeze-out temperatures. The remaining tension between the fit and the proton yield at $T_{\mathrm{ch}} = 156$ MeV is 23 % (2.9$\sigma$). This might be further reduced by construction of a more complete hadron spectrum within the thermal model [1838]. Additional data analyses will clarify the experimental significance of the observed effect. Several possible explanations for these deviations have been suggested. In particular large baryon-antibaryon annihilation rates in the late hadronic phase could be the source of some lower baryon yields [1840]. Such annihilation processes are reflected also in ALICE femtoscopic measurements of $p \bar{p}$ and $\Lambda \bar{\Lambda }$ correlations [1823, 1841], however the yield modification cannot be directly obtained from such considerations.

The influence of these effects on the thermal parameters extracted from the data has been quantified based on UrQMD [1842]. At lower center of mass energies, this approach improves the agreement between the experimentally reconstructed hadrochemical equilibrium points in the (T, $\mu _{B}$) plane and the parton-hadron phase boundary recently predicted by lattice QCD [1764, 1843]. Other possible explanations are based on nonequilibrium thermal models [1844] or a flavor-dependent freeze-out temperature, as indicated by recent lattice QCD calculations [1845].

*g. Particle composition at intermediate* $p_{T}$ To probe how the interplay of soft and hard processes affects the particle composition at intermediate $p_{T}$, baryon-to-meson ratios such as $\Lambda /K_{s}^{0}$ and $p/\pi$ are studied [1846–1848]. An enhancement of $\Lambda /K_{s}^{0}$, relative to the measured ratio in $pp$ collisions, was first observed at RHIC (the so-called baryon anomaly) [1849, 1850]. The ALICE $\Lambda /K_{s}^{0}$ data, measured up to $p_{T} \approx 6$ GeV/$c$, confirm that the effect persists at the LHC, is slightly stronger than at RHIC and extends to higher $p_{T}$. Comparisons of the $\Lambda /K_{s}^{0}$ ratio with models shows that the strong rise of the ratio at low $p_{T}$ can be described by relativistic hydrodynamic models. The EPOS model describes the effect over the entire $p_{T}$ range and for all studied centrality classes [1851]. In contrast to other models, it connects soft and hard processes by a mechanism in which jet-hadrons, produced inside the fluid, pick up quarks and antiquarks from the thermal matter rather than creating $q \bar{q}$ pairs by the Schwinger mechanism.

**Fig. 49:**
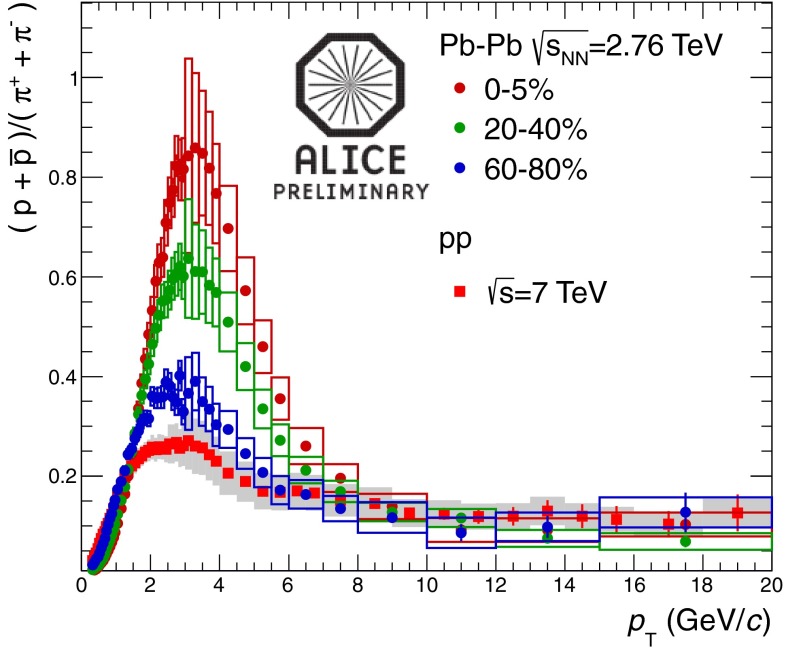
The $p/\pi$ ratio measured for several centrality classes in Pb+Pb collisions relative to $pp$ results at $\sqrt{s_{NN}} = 2.76$ TeV. From [1852]

Figure 49 shows the $p/\pi$ ratio as a function of $p_{T}$ measured up to $p_{T} \approx 20$ GeV/$c$ in several centrality bins in Pb+Pb collisions compared to $pp$ results. At $p_{T} \approx 3$ GeV/$c$, the $p/\pi$ ratio in the 5$~\%$ most central Pb+Pb collisions is a factor 3 larger than the $pp$ ratio. At higher $p_{T}$, the enhancement is reduced and, above 10 GeV/$c$, the Pb+Pb ratio becomes compatible with the $pp$ value. In the most peripheral bin, 60–80 %, the $p/\pi$ ratios in Pb+Pb and $pp$ collisions are comparable over most of the measured $p_{T}$ range.

As is the case for the $\Lambda /K_{s}^{0}$ ratio, the observed anomalous baryon to meson enhancement can be attributed to the effect of radial flow that pushes heavier particles to higher $p_{T}$. However, it seems to extend beyond the region where radial flow is applicable. This enhancement was also interpreted as possibly caused by the recombination of quarks into hadrons [1853]. Further studies involving different other observables are expected to disentangle the different effects.

#### Azimuthal anisotropies

Measurements of azimuthal particle anisotropies probe collective phenomena that are characteristic of a bulk system such as the one expected to be created in heavy-ion collisions [1854]. In non-central collisions, anisotropic pressure gradients, developed in the overlap region of the two colliding nuclei, transform the initial spatial anisotropy into an observed momentum anisotropy, through interactions between the produced particles, leading to an anisotropic particle distribution $dN$/$d\varphi$. This anisotropy is usually quantified via a Fourier expansion of the azimuthal distribution [1855]. The Fourier (or flow) coefficients, $v_{n}$, dependent on $p_{T}$ and pseudorapidity, are given by

6.1 $$\begin{aligned} v_n = \left\langle \cos [n(\varphi - \Psi _{n}) ] \right\rangle , \end{aligned}$$

where $n$ is the order of the flow harmonic, $\varphi$ the azimuthal angle of the particle and $\Psi _n$ the azimuthal angle of the initial state spatial plane of symmetry for harmonic $n$. The isotropic (or angle averaged) component is known as radial flow ($v_0$) while the $v_{1}$ coefficient is referred to as directed flow. The second Fourier coefficient, $v_2$, is the elliptic flow. In this case $\Psi _{2} \approx \Psi _{RP}$ where $\Psi _{RP}$ is the angle of the reaction plane, defined by the beam direction and the impact parameter plane. Elliptic flow has been extensively studied as a measure of collective phenomena in bulk matter in contrast to a superposition of independent $NN$ collisions, where particle momenta would be uncorrelated relative to the reaction plane.

Higher-order odd harmonics, $n \ge 3$, had previously been neglected because they were expected to be zero due to symmetry. However, the statistical nature of individual nucleon–nucleon collisions can lead to highly irregular shapes of the reaction region and thus the corresponding initial energy and pressure distributions [1856, 1857], resulting in event-by-event fluctuations in the elliptic flow direction and magnitude, as well as in all other harmonics. Different experimental methods are used to measure the symmetry plane angles and the $v_{n}$ coefficients, via two- and higher particle correlations [1858–1860]. Each coefficient is sensitive to different effects, allowing a comprehensive study of fluctuations and non-flow contributions.

The first measurements at the LHC [1861] confirmed hydrodynamic predictions and indicated that the system created in Pb+Pb collisions at $\sqrt{s_{NN}}=2.76$ TeV still behaves like a strongly interacting, almost perfect, liquid with minimal shear viscosity to entropy ratio, $\eta /s$, similar to the one at RHIC [1723–1726, 1734].

Further differential studies of the anisotropic flow coefficients involve the quantitative extraction of the transport coefficients of the medium. A precise determination is currently hampered by poor knowledge of the initial state of the collision, along with a significant number of other, smaller, theoretical uncertainties [1862]. One of the key uncertainties is the description of the initial-state geometry. The studies of higher-order flow components, in particular the triangular flow $v_3$ [1857], have provided new input to reduce these uncertainties. A complementary approach was provided by CMS studies of ultra-central collisions, 0–0.2 % [1863], where the initial-state eccentricities are defined by fluctuations of the participant geometry. Additional constraints on $\eta /s$ were obtained by studying $v_{2}$ as a function of centrality and $p_{T}$ for different particle species. Comparison with models typically yields $\eta /s \approx (1-2.5)/4\pi$ [1854], close to the lower bound conjectured by AdS/CFT for a good relativistic quantum fluid [1864]. Recent results [1854] show that “IP-Glasma” initial conditions [1865] and average values of $\eta /s \approx 0.2$ for Pb+Pb collisions at the LHC and 0.12 for Au+Au collisions at RHIC, provide a good description of the majority of the data [165].

*a.* $v_{n}$ *measurements from RHIC to LHC* Compared to RHIC, the LHC has significantly extended the azimuthal anisotropy measurements both in pseudorapidity and $p_{T}$. The ALICE and ATLAS results up to $p_{T} \approx 20$ GeV/$c$ show the same trends as the CMS data, which extends the $v_2$ measurement up to $p_{T} \approx 60$ GeV/$c$ [1812, 1863, 1866].

In general, the integrated $v_{2}$ increases by 20–30$~\%$ at midrapidity and $\approx 30$ % at forward rapidity relative to RHIC, in agreement with hydrodynamic calculations [1867]. The $n=3$ coefficient, $v_{3}$, shows a weak centrality dependence with a similar magnitude in central and peripheral collisions. In central collisions, the magnitude of $v_{2}$ is similar to that of $v_{3}$. These measurements confirm that $v_{2}$ is driven by geometry while $v_{3}$ is dominated by initial-state fluctuations. The latter also generates the finite $v_{2}$ in the most central collisions which approximates the ideal case of zero impact parameter.

The fourth-order harmonic was measured with respect to the second- and fourth-order event planes, $v_{4}(\Psi _2)$ and $v_{4}(\Psi _4)$ [1812, 1866]. The difference between the results for the two event planes is entirely due to fluctuations in the fourth-order harmonic flow and, as such, provides important constraints on the physics and origin of the flow fluctuations.

At LHC energies, the large particle multiplicities produced in each event also allow a determination of the flow coefficients in individual events. The ATLAS collaboration has measured $v_n$ for $2<n<4$ event-by-event [1868]. Comparisons with a Glauber-based geometric model [1869] and a model that includes corrections to the initial geometry due to gluon saturation effects [1870] fail to describe the experimental data consistently over most of the measured centrality range.

In addition to the integrated value of $v_{2}$, valuable information can also be determined from the dependence of $v_2$ on transverse momentum and particle mass. The shape of the $p_{T}$-differential anisotropic flow is determined by different underlying physics processes in the various $p_{T}$ regions. The behavior of the bulk matter for $p_{T} < 1$–$2~\text {GeV}/c$ is mostly determined by hydrodynamic flow which exhibits a typical “mass splitting” [1871] induced by the collective radial expansion of the system. While the effect is cumulative over the lifetime of the system, it has a significant contribution from the partonic phase. However, hadronic rescattering in the late stages might mask the information from the early stage.

The measured value of $v_{2}$ reaches a maximum around $p_{T} \approx 2$ GeV/$c$ and slowly decreases until it approaches zero for $p_{T} \approx$ 40–60  GeV/$c$ as measured by CMS. At $p_{T} > 10$ GeV/$c$ the elliptic flow results are well described by extrapolation of the WHDG model [1872] to LHC energies [1873], which takes into account collisional and radiative energy loss in an expanding medium. In this model, the anisotropy is controlled by the energy loss mechanism. Similar to $R_{AA}$, only a minor dependence on particle type is expected in this region.

**Fig. 50:**
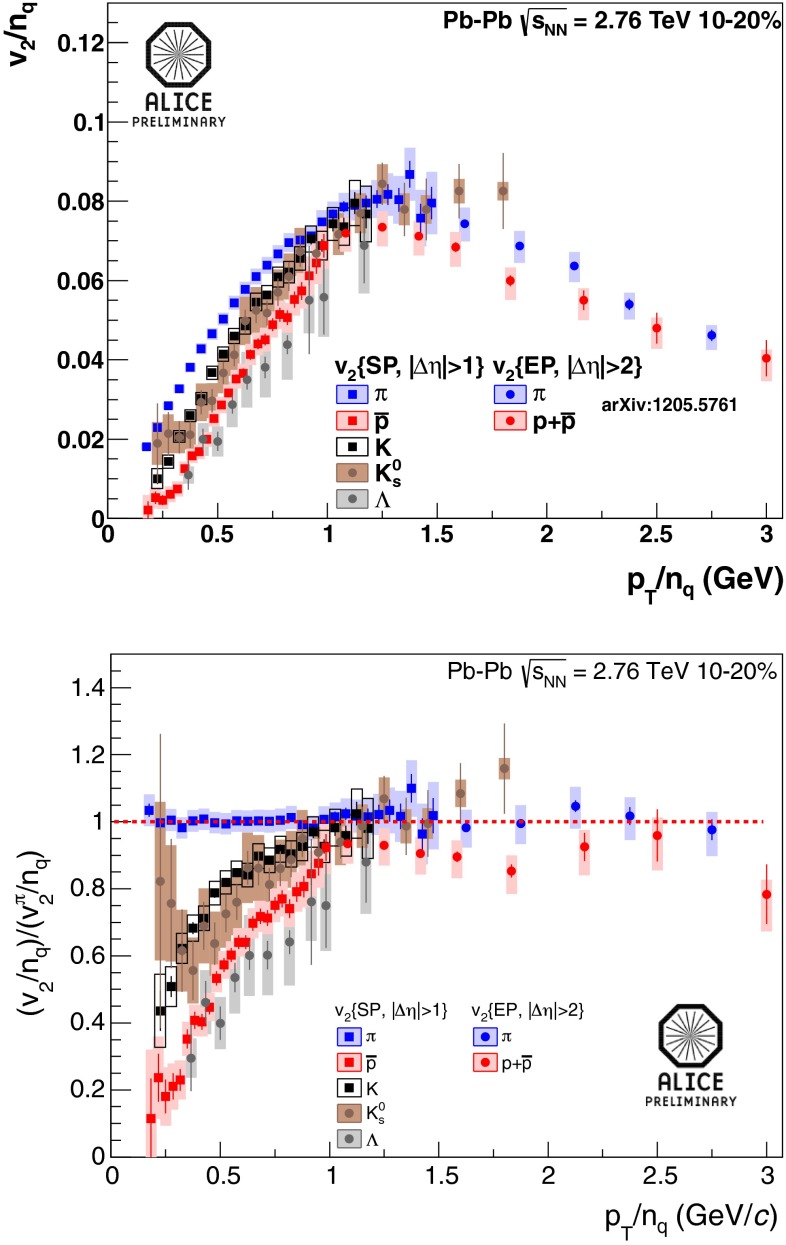
*Top* Elliptic flow coefficient $v_{2}$ as a function of the transverse momenta scaled by the number of constituent quarks in Pb+Pb collisions at $\sqrt{s_{NN}}=2.76$ TeV. *Bottom* The same data are shown normalized to the polynomial fit to the pion elliptic flow. From [1874]

*b. Quark number scaling* At RHIC, it was observed that all baryons exhibited a similar anisotropic flow pattern; with the ratio of baryon to meson $v_2$ being 3:2, see Ref. [1875] and references therein. These findings suggested that hadron formation at intermediate $p_{T}$ is dominated by quark coalescence at the end of the partonic evolution [1876]. PHENIX has observed that the scaling is broken when plotted as a function of the transverse kinetic energy at $KE_\mathrm{T}/n > 1$ GeV in all but the most central collisions [1877]. ALICE has subsequently studied quark number scaling for a number of identified particles in different centrality ranges. Also at the LHC, quark scaling appears to be broken for transverse momenta per number of constituent quarks, $p_{T}$ $/n_q$, below 1 GeV/$c^2$. At higher $p_{T}$, the scaling appears to hold at the 20 % level as shown in Fig. 50. The significance of this scaling and the size of the violations needs further study. These theoretical and experimental investigations are of particular importance as a picture of anisotropic quark flow and subsequent hadronization via coalescence has been related to deconfinement by some authors [1878–1882].

#### Transport coefficients and spectral functions: theory

A comparison of RHIC and LHC heavy-ion data with the results of viscous hydrodynamic simulations for quantities such as the elliptic flow seems to imply a remarkably small value of the shear viscosity of the QGP (see Sect. 6.2.2). While the quantitative value depends somewhat on the details of the simulation, in particular on the initial conditions, it is widely accepted that the shear viscosity to entropy ratio $\eta /s$ is rather close to the value $1/(4\pi )$ [1864] found in strongly coupled gauge theories with gravity duals. The existing (full leading order) weak-coupling prediction for this ratio is considerably larger for reasonable values of $\alpha _\mathrm{s}$ [1883].

Thus, it is very important to develop non-perturbative first-principles tools to compute the shear viscosity in QCD. More generally, transport coefficients such as the shear viscosity can be regarded as the low-energy constants of hydrodynamics, which describes slow, long-wavelength departures from equilibrium of a thermal system. The values of the transport coefficients, however, must be computed in the underlying microscopic theory—QCD in the case of the quark–gluon plasma. In strongly coupled gauge theories, important progress has been made employing the gauge/gravity correspondence; see Sect. 6.6 as well as Refs. [1884–1886]. This correspondence provides a paradigm diametrically opposite to the quasi-particle picture that underlies weak-coupling calculations. The relative ease with which real-time physics can be extracted from the gauge/gravity correspondence at strong coupling, such as with the methods of Ref. [1884], is particularly impressive. Although non-supersymmetric and conformally broken quantum field theories have been investigated using the gauge/gravity correspondence (see for instance [1887, 1888]), no exact QCD dual has been constructed to date, hence the phenomenological predictions obtained from gauge/gravity techniques must be regarded as semi-quantitative at best.

On the other hand, the lattice QCD framework is ideally suited to reliably determine the equilibrium characteristics of the QGP, such as the equation of state, see also Sect. 6.1. Because numerical lattice gauge theory employs the Euclidean formalism of thermal field theory, dynamical properties are normally only accessible through analytic continuation, posing a considerable numerical challenge; see Ref. [1889] for a recent review.

Spectral functions encode important dynamical properties of the medium. For instance, the photon and dilepton production rates in the QGP are proportional to the spectral function of the conserved vector current. Hydrodynamic modes and quarkonium states show up as peaks whose widths are proportional to the rate at which these excitations dissipate. In lattice QCD, the spectral function is obtained by solving the integral equation

6.2 $$\begin{aligned} G(\tau ,\varvec{k},T) \!=\! \int _0^\infty \! d\omega \; \rho (\omega ,\varvec{k},T) \; \frac{\cosh [\omega (\frac{1}{2T}-\tau )]}{\sinh [\frac{\omega }{2T}]}, \end{aligned}$$

given the Euclidean correlator $G$ at a discrete set of points $\tau$ with a finite statistical accuracy. When Euclidean correlation functions are known numerically instead of analytically, the determination of the spectral function involves the solution of a numerically ill-posed inverse problem. Compared to nonrelativistic systems such as cold Fermi gases, such as in Ref. [1890], QCD has the added difficulty that correlation functions are strongly divergent at short distances. In spite of these difficulties, with good numerical data and the help of prior analytic information, including effective field theory, sum rules and the operator product expansion, the gross features of the spectral function $\rho$ can be determined. In practice, however, the temperature scale imposes a limit on the frequency resolution. Then the identification of bound states or transport peaks, substantially narrower than the temperature, cannot be formulated in a model independent way. An accurate and reliable calculation of the Euclidean correlators nevertheless remains an important goal for lattice QCD, not least because they can be used to test various analytic methods; see Sect. 6.6.

At zero temperature, an one-to-one correspondence exists between the spectral function below inelastic thresholds and stationary observables, thus making the spectral function directly accessible to lattice QCD [1891]. A typical example is the possibility to calculate the $\rho$-channel spectral function [397, 1891] in the elastic regime without an explicit analytic continuation. Whether a similar correspondence can be constructed for the nonequilibrium properties of the QGP along these lines has yet to be determined. In particular, the finite volume used in the lattice simulations plays a crucial role in relating stationary observables to dynamical quantities at $T=0$ and the volume effects on the thermal spectral function should be investigated.

In the following, we briefly discuss several channels of interest. Consider first the spectral function of the conserved vector current. For a generic frequency, NLO perturbative calculations are available, including quark mass corrections [1892]. For the light quark flavors, the vector channel is related to the production of real photons and lepton pairs in the thermal medium. A recent NLO calculation of the thermal photon production rate showed that the convergence rate is reasonably good [1893]. The dilepton rate for an invariant mass on the order of the typical thermal momentum has been computed at NLO even for a non-vanishing spatial momentum [1894]. Extensive phenomenological studies have been carried out in order to compare different spectral function calculations to heavy-ion data [1895]. The low-energy part of the experimental dilepton spectrum was found to be dominated by the contribution from the confined phase. Lattice results have been reported in the continuum limit of the quenched approximation [1896], as well as with dynamical quarks at a single lattice spacing [1897, 1898]. In the thermodynamic limit, the thermal part of the spectral function is constrained by a sum rule [1899]. In the chirally restored phase of QCD with two massless quarks, the isovector-vector and axial-vector correlators are exactly degenerate so that the thermal generalization of the Weinberg sum rule [1900] is trivially satisfied.

In the shear [1901, 1902] and bulk [1903, 1904] channels, lattice QCD data are so far only available for pure Yang–Mills theory. This is due to the need for very high statistics in the flavor singlet channels which can only be reached in the computationally faster Yang–Mills case. In the bulk channel, the operator product expansion and a sum rule have also been used to further constrain the spectral function [1903]. In the shear channel, the corresponding sum rule remains incompletely known due to the complicated structure of contact terms (the correlator has a stronger short distance singularity here than in the bulk or vector channels). A more systematic derivation of sum rules and the operator product expansion predictions of the asymptotic behavior of the spectral functions is thus required [1905, 1906]. There has recently been substantial progress in perturbative calculations of the shear [1907] and bulk [1908] channel spectral functions. The convergence of the perturbative results for the Euclidean correlators is good, particularly in the shear channel. These calculations provide very useful information that can eventually be combined with numerical lattice data.

We briefly consider the idealized problem of heavy-quark diffusion in the QGP in the static limit, $m_{q}\rightarrow \infty$. An NLO perturbative calculation [1909] is available; unfortunately the convergence rate turns out to be poor. The main quantity of interest, the momentum diffusion coefficient, $\kappa$, can be extracted with Heavy Quark Effective Theory [1910] as well as with lattice QCD [1911–1913]. Since the physical observable is essentially reduced to a pure gluonic one, it is expected to be accurately computed in pure Yang–Mills theory. An important advantage of this channel over those discussed above is that no sharp features are expected in the spectral function [1910], even at weak coupling, which makes the inverse problem better defined. The most important next steps will be to determine the normalization of the chromoelectric field operator non-perturbatively and to take the continuum limit of the Euclidean correlator before attacking the inverse problem. Whether the operator product expansion and a possible sum rule can also be useful here is not yet clear and deserves further investigation.

### Approach to equilibrium

A major challenge for the theoretical description of heavy-ion collisions is to follow the evolution of the system from its initial state to a near-equilibrium plasma, the behavior of which can be approximated by hydrodynamics. To describe this equilibration process, it is necessary to solve a strongly time-dependent system away from both asymptotically weak and strong coupling. In this section, we describe recent developments in this direction, covering early perturbative work as well as holographic results.

#### Thermalization at weak and strong coupling

Conceptually, relativistic heavy-ion collisions evolve in steps. The initial nuclear collision liberates partons, which become a nonequilibrium quark–gluon plasma (or liquid), which in turn equilibrates to form a quark–gluon plasma in approximate local equilibrium. Near-equilibrium hydrodynamics then describes the evolution of the plasma from deconfinement until the time that the system begins to hadronize [1914]. The stage of the system from the initial collision through the nonequilibrium plasma has been called the “glasma”. This term arises from the description of the initial nuclei in terms of the color glass condensate, a state characterized by the presence of strong color fields and the over-occupation of soft gluon modes. The transition between the nonequilibrium and equilibrium plasma may in turn be investigated using methods generalized from traditional plasma physics to non-Abelian gauge theories.

Important recent progress has been made in understanding the processes by which a pre-equilibrium QGP approaches equilibrium at high energies or weak coupling. The situation is complicated by the fact that even in the limit where the gauge coupling is treated as arbitrarily small, the initial color fields are strong enough to make the dynamics of the system non-perturbative. The same is true even at later times for filamentary instabilities which result in the growth of chromomagnetic fields large enough to compensate for the small coupling. Thus, quantifying how the equilibration time of the system depends on the coupling appears to require a combination of analytic weak-coupling techniques and classical real-time lattice simulations. The latter, at weak coupling, correctly treat the nonlinear dynamics of the classical fields representing large soft gluon occupation numbers.

Competing analytic scenarios for the equilibration process in non-Abelian gauge theories include the bottom-up picture of Ref. [1915], as well as the newer proposals of Refs. [1916, 1917], emphasizing the role of plasma instabilities, and Ref. [1918] involving formation of a gluonic Bose-Einstein condensate. Very recently, classical simulations of SU(2) lattice gauge theory were carried out in a longitudinally-expanding system [1919]. It was found that, independent of the initial conditions, the system always appears to approach an attractor solution with scaling exponents consistent with the bottom-up solution [1915]. However, closely related work [162] challenges this outcome and instead suggests fast isotropization of the system.

Once the weak coupling thermalization mechanism has been qualitatively understood, this insight needs to be translated into quantitative predictions. A calculation of the transition of the system from pre-equilibrium to equilibrium could then be coupled to weak-coupling glasma calculations of the creation of the initial pre-equilibrium plasma to provide a complete picture of the dynamics. Recent progress in calculating the seeding and development of instabilities in the glasma [1920] is an encouraging development in this direction.

Equilibration of the QGP can also be studied in an altogether different and highly complementary limit, i.e., in strongly coupled QCD-like plasmas that have a dual gravity description. A clear distinction between the equilibration, isotropization, and hydrodynamization processes of the plasma has been achieved in this limit [1921–1924]. Formally, the success of hydrodynamics only depends on the isotropization of the stress tensor (i.e., the pressure) in the local fluid frame and not necessarily on thermal equilibration, while viscous hydrodynamics accounts for small deviations from an isotropic pressure. The observation that hydrodynamics may be a very good approximation even in situations where the anisotropy is not small [1922, 1923] was a surprise, and is not yet completely understood. This may be of quite some phenomenological relevance, as viscous hydrodynamic simulations of heavy-ion collisions reveal significant pressure anisotropy at some stages of the collision.

In the future, it is necessary to understand why hydrodynamics seems to provide an accurate description at earlier times and in a wider range of systems than naively expected. In the case of strong coupling, some of the approximations inherent in the holographic calculations listed above, such as the conformal invariance of the field theory and the limits of infinite ’t Hooft coupling and $N_\mathrm{c}$, should be relaxed. To this end, the equilibration of an ${\mathcal N}=4$ SYM plasma was studied at large but finite coupling [1925–1927], showing a clear weakening of the usual top-down pattern of holographic thermalization.

#### Multiplicities and entropy production

The particle multiplicities in heavy-ion collisions can be estimated in several ways. Event generators determine multiplicities from their models of soft particle production followed by fragmentation and hadronization [1851, 1928–1937]. A more first-principles QCD approach comes from color glass condensate (CGC), a saturation-based description of the initial state in which nuclei in a high-energy nuclear collision appear to be sheets of high-density gluon matter. In this approach, gluon production can be described by $k_{T}$-factorization which assumes an ordering in intrinsic transverse momentum rather than momentum fraction $x$, as in collinear factorization. The unintegrated gluon density associated with $k_\mathrm{T}$ factorization is related to the color dipole forward scattering amplitude which satisfies the JIMWLK evolution equations [155, 158, 1938]. In the large-$N_\mathrm{c}$ limit, the coupled JIMWLK equations simplify to the Balitsky-Kovchegov (BK) equation [150, 153, 154, 1939], a closed-form result for the rapidity evolution of the dipole amplitude. The running coupling corrections to the leading log BK equation, rcBK, have been phenomenologically successful in describing the rapidity/energy evolution of the dipole [161]. The initial condition still needs to be modeled, typically by a form motivated by the McLerran–Venugopalan model [1940–1942] with parameters constrained by data  [1943]. The impact parameter-dependent dipole saturation model (IP-Sat) [1944–1946] is a refinement of the dipole saturation model that reproduces the correct limit when the dipole radius $r_\mathrm{T} \rightarrow 0$. It includes power corrections to the collinear DGLAP evolution and should be valid where logs in $Q^2$ dominate logs of $x$. It should be noted that all of the above approaches involve some parameter tuning at some energy to predict results for other energies; for details and further model references see Ref. [1947].

**Fig. 51:**
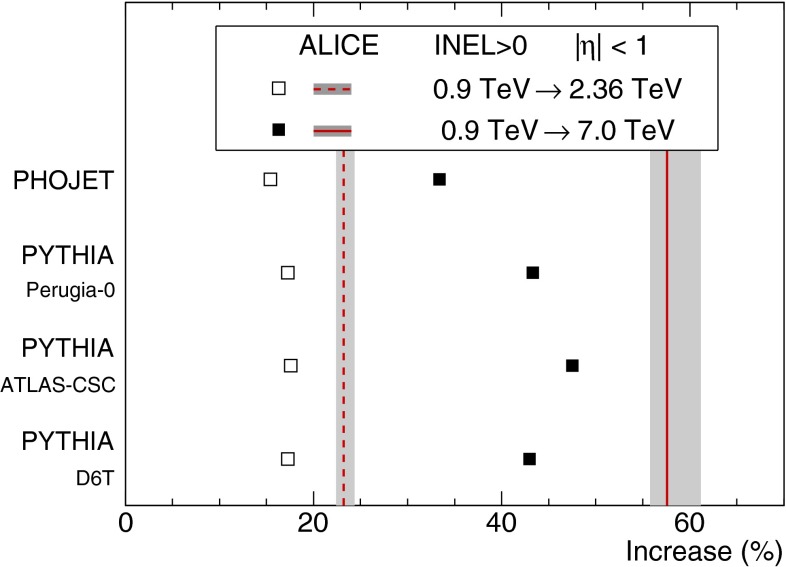
The relative increase of the charged particle pseudorapidity density for inelastic collisions having at least one charged particle in $|\eta | < 1$, between $\sqrt{s}=0.9$ and 2.36 TeV (*open squares*) and between $\sqrt{s}= 0.9$ and 7 TeV (*full squares*), is shown for various models. The corresponding ALICE measurements are shown by the *vertical dashed* and *solid lines*. The width of the *shaded bands* correspond to the statistical and systematic uncertainties added in quadrature [1948]

**Fig. 52:**
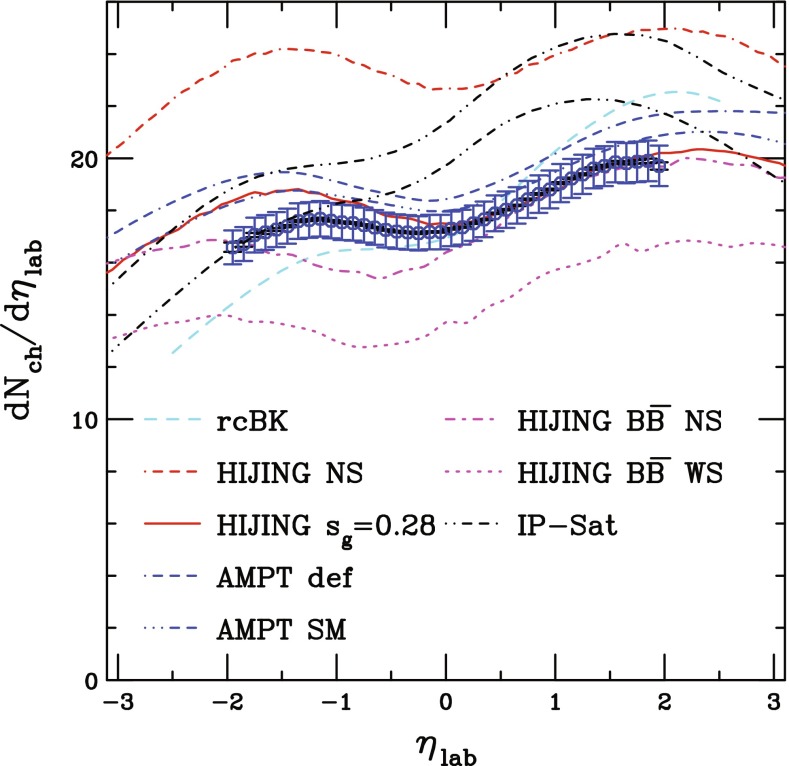
Charged particle pseudorapidity distributions for $p$+Pb collisions at $\sqrt{s_{NN}}= 5.02$ TeV in the laboratory frame. A forward-backward asymmetry between the proton and lead hemispheres is clearly visible with the $\mathrm{Pb}$ remnant going into the direction of positive pseudorapidity. The rcBK (*dashed cyan*) result is from Ref. [1943]. The IP-Sat result is shown as the *dot-dot-dash-dashed black curves*. The HIJING2.1 result without (NS, *dot-dash-dash-dashed red*) and with shadowing ($s_g = 0.28$, *solid red*) and the HIJINGB$\overline{\mathrm{B}}$ result without (*dot-dashed magenta*) and with shadowing (*dotted magenta*) are also shown. Finally, the AMPT-def (*dot-dash-dash-dashed blue*) and AMPT-SM (*dot-dot-dot-dash-dash-dashed blue*) are given. The ALICE results from Ref. [1950] are given. The systematic uncertainties are shown, the statistical uncertainties are too small to be visible on the scale of the plot. From [1947]

**Fig. 53:**
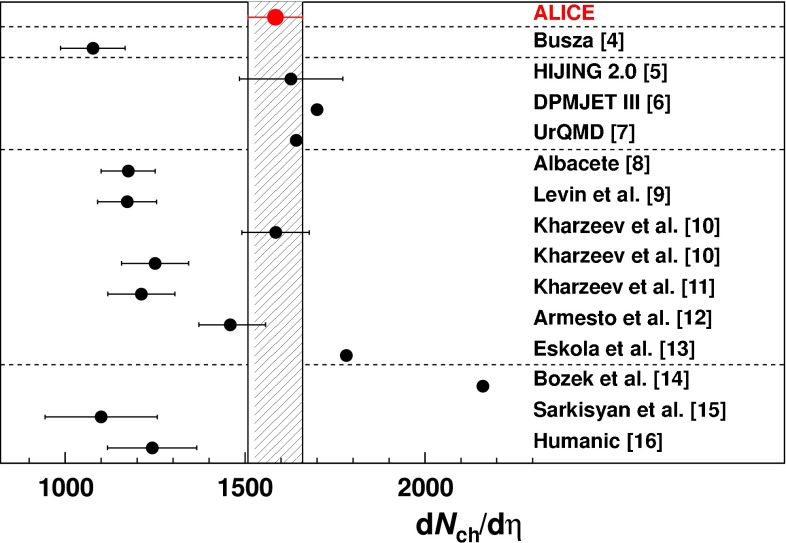
The charged particle pseudorapidity distributions in Pb+Pb collisions at $\sqrt{s_{NN}} = 2.76$ TeV [1951] are compared to model predictions. The horizontal dashed lines group similar theoretical approaches. For the model references see [1951]

Figures 51, 52 and 53 show model predictions of the charged particle multiplicity densities in $pp$, $p+$Pb, and Pb+Pb collisions compared to data.

Figure 51 shows a comparison of charged particle pseudorapidity density in $pp$ collisions at $\sqrt{s} = 0.9$, 2.36 and 7 TeV, measured by the ALICE Collaboration [1948]. The results for the relative increase of the $dN_{\mathrm{ch}}/d\eta$ in $|\eta | < 1$ between 0.9 and 2.36 TeV and between 0.9 and 7 TeV were compared to models. Three different PYTHIA tunes were compared, along with PHOJET results. The Perugia-0 tune and PHOJET were chosen because they exhibited the largest difference in multiplicity distributions at very low multiplicities. All the models underpredicted the observed relative increase.

The shapes and magnitudes of the pseudorapidity distributions predicted by models are compared to the $p+$Pb test beam data in Fig. 52. While several of the calculations are in relatively good agreement with the value of $dN_{\mathrm{ch}}/d\eta$ at $\eta _{\mathrm{lab}} = 0$, the shapes are generally not compatible with that of the data. The rcBK result was calculated assuming the same rapidity to pseudorapidity transformation in $pp$ as in $p+$Pb collisions. Another choice, based on the number of participants in the Pb nucleus would lead to a flatter distribution, more compatible with the data [1949]. Most of the event generator results disagree with both the shape and magnitude of the data except for AMPT and HIJING2.0 with shadowing ($s_g = 0.28$ in Fig. 52).

Finally, Fig. 53 compares several classes of model predictions to the Pb+Pb data at midrapidity, $|\eta |< 0.5$ [1951]. The result, $dN_{\mathrm{ch}}/d\eta = 1584 \pm 4 (\mathrm{stat.}) \pm 76 (\mathrm{syst.})$, is a factor of 2.2 larger than the 200 GeV Au+Au result at RHIC. All model calculations shown in Fig. 53 describe the RHIC results. However, most of them underpredict the Pb+Pb data by ${\sim } 25$ %, including empirical extrapolations from lower-energy data (labeled Busza); many saturation-based models (only one of the estimates from Kharzeev et al. agrees with the data); an extrapolation based on Landau hydrodynamics (maximum compression) (labeled Sarkisyan et al.); and hadronic rescattering (labeled Humanic). The event generator results in the upper part of Fig. 53 are generally in relatively good agreement with the data. Calculations based on hydrodynamics generally overpredict the data: a hybrid hydrodynamics and phase-space saturation calculation (labeled Eskola et al.) overpredicts the multiplicity by 7 % while a hydrodynamic model with a multiplicity scaled from $pp$ collisions (labeled Bozek et al.) overestimates the result by 40 %. This comparison illustrates that, even if model calculations are tuned to results at one energy, agreement with higher-energy data is not guaranteed. As Fig. 52 showed, predicting the average multiplicity at one rapidity also does not guarantee that the full pseudorapidity dependence can be reproduced.

**Fig. 54:**
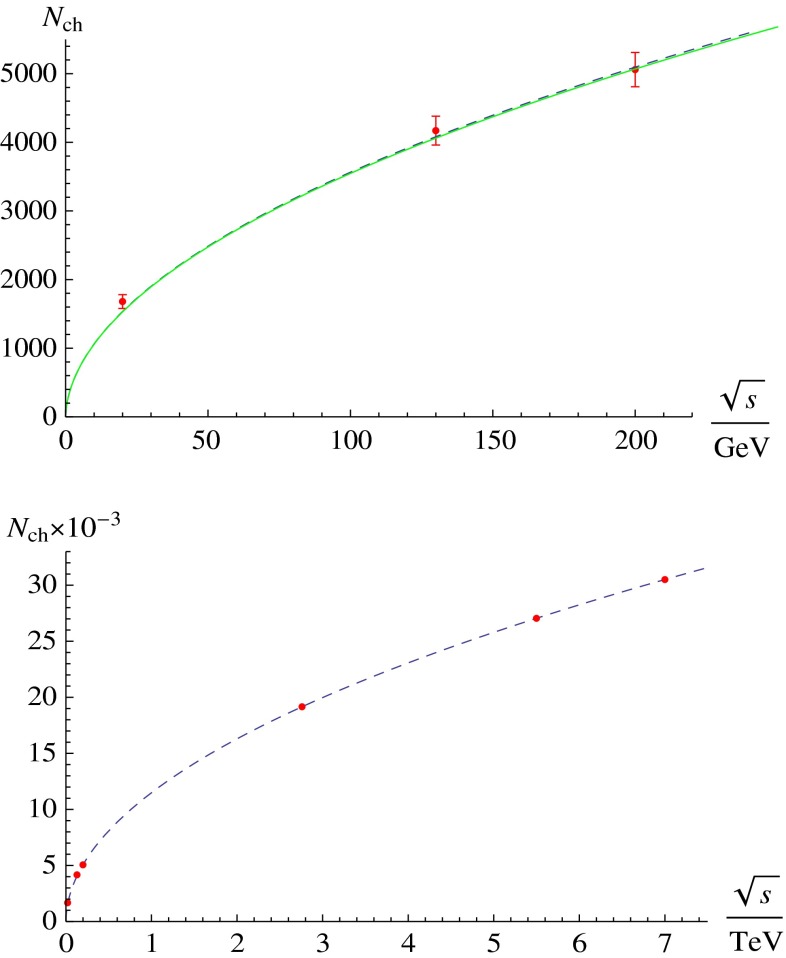
The total multiplicity as a function of center-of-mass energy measured in Au+Au collisions at RHIC (*top*) and Pb+Pb collisions at the LHC (*bottom*). The *points* on the top figure correspond to RHIC data while the *dashed curve* shows a prediction from the IHQCD scenario [1961]. In the lower figure, the *dashed line* is a prediction of the same holographic calculation, extended to higher energies. The *red point* at $2.76$ TeV corresponds to the ALICE measurement [1965], while the other red points are predictions for future LHC runs

Finally, multiplicities can be estimated with holographic methods. In the dual gravity description, local thermalization involves the formation of a horizon. The area of this horizon controls the final-state multiplicities [1952]. Gravitational techniques, pioneered by Penrose [1953], have provided useful tools for estimating the formation of horizons and given bounds for the related multiplicities using the concept of “trapped surfaces”. Several calculations in the dual of ${\mathcal N}=4$ SYM theory have analyzed the formation of trapped surfaces in collisions of planar shock waves [1954–1961]. In [1962], it was shown that the entropy released during the collision is 60 % larger that the bound obtained from trapped surface calculations, a result that is independent of the collision energy due to the conformality of the system.

In the simplest models involving planar shock waves in an AdS$_5$ space-time, the entropy and the total multiplicities scale as $N_{\mathrm{tot}} \sim s^{1/3}$. If the running coupling is simulated with an ultraviolet (UV) cutoff for the trapped surface, the energy dependence of $N_{\mathrm{tot}}$ changes to $N_{\mathrm{tot}}\sim s^{1/6}$, indicating that violation of conformal invariance may affect the dynamics of heavy-ion collisions. This issue was studied in two different approaches [1961]: “AdS-$Q_\mathrm{s}$”, where an explicit UV cutoff is introduced at $r=1/Q_\mathrm{s}$ (see [1961] for details), and Improved Holographic QCD (IHQCD), where conformal invariance is broken due to a dynamical dilaton field [1888, 1963, 1964]. The RHIC data point was fit to determine the constant parameter that scales the calculated multiplicity. Thus, the energy dependence of the multiplicity is fixed. The upper part of Fig. 54 indicates that other RHIC multiplicities are successfully reproduced. A subsequent extension to LHC energies is shown in the lower part of Fig. 54. The red points are predictions for 2.76, 5.5 and 7 TeV Pb+Pb collisions. The 2.76 TeV result is in agreement with the ALICE result [1965]. As seen in Fig. 54, the agreement of the IHQCD calculation with data is good.

### Hard processes and medium-induced effects

#### Introduction

The high energies reached in heavy-ion collision experiments at RHIC and the LHC allow precision studies of hard processes involving high momentum or mass scales. Such probes originate from partonic scatterings in the very initial stage of the collision and thus are sensitive to the state of the system at early times.

A crucial issue in the study of heavy-ion collisions is employing an appropriate reference system which would disentangle medium effects from vacuum expectations. Proton-proton collisions provide the vacuum reference, as it was verified at lower energies and then at the LHC. However, these hard probes are also subject to the state of the nuclear matter systems, when no hot matter is produced. To this end, hard probes have been studied in $\mathrm{d}+$Au collisions at RHIC and, most recently, in $p$+Pb collisions at the LHC to separate initial-state from final-state matter effects. A discussion of the theory of the initial state effects in nuclear collisions can be found in Sect. 6.4.2, while experimental results on $p$+Pb collisions at the LHC are presented in Sect. 6.5.

A detailed analysis of phenomena such as parton energy loss via collisions and medium-induced gluon radiation offers new insight into the most fundamental properties of hot QCD matter and constitutes an important subfield of heavy-ion physics. Perturbative calculations of radiative energy loss [1966] generally predict that the energy loss of a parton should be proportional to the Casimir eigenvalue of its color charge [1967]. This implies that gluons should lose approximately twice as much energy as quarks. In addition, the energy lost by heavy quarks should be reduced by the so-called dead-cone effect, i.e., the suppression of gluon radiation at small angles [1967]. However, strongly coupled gauge theories applying the AdS/CFT conjecture often predict that energy loss has a stronger dependence on the path length of the probe through the medium. These different scenarios, as well as other issues related to the theoretical description of parton energy loss, are discussed in Sect. 6.4.2.

Some of the best known hard probes are the quarkonium states. Bound states of charm and bottom quarks are predicted to be suppressed in heavy-ion collisions as a consequence of “melting” due to color screening in a QGP. Suppression of the $J/\psi$ was first predicted by Matsui and Satz [1968] in 1986. This idea was later developed into a sequential pattern of suppression for all heavy quarkonium states since the magnitude of suppression should depend on their binding energy with the most strongly bound $\Upsilon$(1S) state showing only little modification. However, other cold and hot matter effects may also contribute, see Sects. 6.4.2 for further discussion.

Experimentally, the focus of hard probes has mainly been on high $p_{T}$ hadrons, heavy flavors and quarkonium states. Manifestations of parton energy loss were first observed as strong suppression of back-to-back-emission of high $p_{T}$ hadrons at RHIC. The higher energies of the LHC allow these studies to be expanded to much higher $p_{T}$ as well as fully reconstructed jets, as discussed in Sect. 6.4.4. The mass dependence of parton energy loss, as well as other open heavy flavor observables are also described in Sect. 6.4.4, along with results on quarkonium production and suppression. Early intriguing results emerging from the LHC $p$+Pb program are also presented.

#### Theory of hard probes

*Nuclear matter effects in* $p$A *collisions*   As discussed in the introduction to this section, a reliable reference for heavy-ion results is critical for understanding the strength of plasma effects relative to non-plasma effects, referred to here as cold nuclear matter effects. These effects are in addition to the vacuum reference obtained in proton–proton collisions. They have been studied already in fixed-target interactions in addition to higher-energy measurements in $\mathrm{d}+$Au and $p$+Pb collisions at the RHIC and LHC colliders. In this section, effects important for the cold nuclear matter baseline are introduced and discussed. We do not discuss results of the highest multiplicity $pp$ and $p$+Pb collisions, for those, see Sect. 6.5.

There are several important cold nuclear matter effects that need to be taken into account when determining the strength of deconfinement effects on a particular final state. The most general, affecting all production processes, is the modification of the parton distributions in nuclei, often referred to as shadowing. This effect is well known, starting from the EMC effect at relatively large Bjorken $x$ [1969] and studied further at lower $x$ in nuclear deep-inelastic scattering (nDIS) experiments at SLAC [1970], CERN [1971–1973], HERA [1974, 1975], and Fermilab [1976]. Given the fixed-target nature of these experiments, only moderately low values of $x$ ($x \ge 0.01$) are reached at perturbative momentum transfers ($Q^2 > 1$ GeV$^2$). These data are augmented by Drell–Yan hadroproduction data at higher $Q^2$ and moderate $x$ [1977, 1978].

Since the nDIS experiments probe only charged parton densities, the nature and magnitude of the effect on the nuclear gluon density was known only from the $Q^2$ evolution of the structure function [1979] and the momentum sum rule, see e.g. Ref. [139]. While data from the RHIC collider have extended the range in $x$ and $Q^2$, in particular through $\pi ^0$ production [1980], they have not directly probed the gluon density. One possible experimental means of probing the nuclear gluon density is through ultraperipheral collisions at the LHC [1981]. In these collisions, the nuclei do not touch and only interact electromagnetically so that $J/\psi$ photoproduction involves the low $x$ gluon density in a single nucleus. The ALICE collaboration has already published such data and shows that this method can eliminate certain shadowing parameterizations [1982, 1983].

The effects of shadowing in nuclei are parameterized by various groups using global fitting methods similar to those used to evaluate the parton densities in the proton, see Sect. 3. The first such parameterizations were rather crude, involving only a single leading-order (LO) modification for quarks, antiquarks and gluons as a function of $A$ and $x$ but independent of $Q^2$ [1984]. Greater levels of sophistication have been introduced until, currently, LO and NLO sets are available with up to 31 error sets, evolving quarks, antiquarks and gluons separately with $Q^2$. Some recent sets are EPS09 [139], DSSZ [138], HKN07 [140] and FGS10 [1985]. Regardless of the level of sophistication and general agreement between different sets on the valence and sea quark densities in nuclei, the uncertainty on the gluon density in the nucleus remains large without general agreement on the best fit shape.

Quark-dominated production processes in nuclear collisions also exhibit a dependence on the relative neutron–proton content of the nucleus (isospin). For some final states, the change in production rates with nuclei related to isospin is as strong or stronger than that due to shadowing [1986, 1987]. The high energies of the LHC allow studies of these effects at higher $Q^2$ than ever before with low to moderate values of $x$, such as for vector boson production [1986–1988]. Such data are available already for $W$ and $Z^0$ production in Pb+Pb collisions at $\sqrt{s_{NN}} = 2.76$ TeV from the ATLAS [1989] and CMS [1990, 1991] collaborations.

Another significant unknown relating to nuclear shadowing is its dependence on impact parameter or collision centrality. Fixed-target data were presented as a function of $A$ and did not try to distinguish between nuclear interaction points. One exception was an experiment studying gray tracks in emulsion which did see hints of an impact parameter dependence [1992]. The impact parameter dependence was neglected in most previous parameterizations, the exception being the FGS parameterizations based on diffractive data [1985]. Instead, assumptions based on either a linear dependence on path length through the nucleus or the nuclear density were introduced [1993]. Only recently have data emerged to challenge the assumption of a linear dependence. The PHENIX $\mathrm{d}+$Au $J/\psi$ data suggested a stronger than linear dependence [1994]. These results prove challenging for the recent EPS09s spatially dependent modifications which retain up to quartic powers in the expansion of the centrality dependence as a function of path length for $A$-independent coefficients [1995]. Instead these data suggest that shadowing is concentrated in the core of the nucleus with radius of $R\sim 2.4$ fm with a relatively sharp surface, a width of $d\sim 0.12$ fm [1996]. These studies need to be backed up with more data over more final states.

A second cold matter effect is energy loss in medium. This has been treated as both an initial-state effect, related to soft scatterings of the projectile parton in the nucleus before the hard scattering to produce the final-state particle, and a final-state effect where the produced parton scatters in the medium. Initial-state energy loss has been studied in Drell–Yan production [1997]. The effect has generally been found to be small, too small to be effectively applied to $J/\psi$ production at large Feynman $x$ ($x_\mathrm{F}$) [1998]. In addition, there is an inherent ambiguity when applying initial-state energy loss to Drell–Yan production since most groups parameterizing the nuclear parton densities include these same Drell–Yan data to extract the strength of shadowing on the antiquark densities [139]. Also, by forcing the loss to be large enough to explain the high $x_\mathrm{F}$ behavior of $J/\psi$ production in fixed-target interactions [1999] violates the upper bound on energy loss established by small angle forward gluon emission [2000]. More recently, it has been proposed that rather than an initial-state effect, cold matter energy loss should be treated as a final-state effect, with scattering of the produced final-state with gluons in the medium [2001]. This would eliminate the ambiguity of shadowing relative to initial-state energy loss in Drell–Yan production and, indeed, eliminate the need to introduce energy loss effects on Drell–Yan production completely. The final-state energy loss in $pA$ collisions is currently implemented for quarkonium production as a probability distribution dependent on the energy loss parameter. The effect modifies the $x_\mathrm{F}$ and $p_\mathrm{T}$ distributions in a rather crude fashion since the quarkonium distribution in $pp$ collisions is parameterized as a convolution of factorized power laws, $\propto (1-x)^n (p_0^2/(p_0^2 + p_\mathrm{T}^2))^m$, rather than using a quarkonium production model [2001, 2002]. It has yet to be implemented for other processes.

As previously mentioned, initial-state energy loss in the medium can be connected to transverse momentum kicks that broaden the $p_\mathrm{T}$ distributions in nuclei relative to those in $pp$ collisions. This can be related to the Cronin effect [2003] and was first seen for hard processes in fixed-target jet production [2004].

Nuclear absorption, which affects only quarkonium states, involves break-up of the nascent quarkonium state in cold nuclear matter [2005]. Thus it is a final-state effect. The matter that causes the state to break up is typically assumed to be nucleons only. However, $J/\psi$ suppression in nuclear collisions was also attributed to break-up with produced particles called comovers.

Absorption is the only effect we have discussed that is related to the size and production mechanism of the interacting state and can be described by a survival probability, $S_A^{\mathrm{abs}} = \exp \{ -\int _z^\infty dz' \rho _A(b,z') \sigma ^C_{\mathrm{abs}}(z-z')\}$ where $z'$ is the production point and $z$ is the dissociation point; $\rho _A(b,z')$ is the nuclear matter density; and $\sigma ^C_{\mathrm{abs}}$ is the effective absorption cross section for quarkonium state $C$ [2006]. Because the quarkonium states have different radii, $\sigma ^C_{\mathrm{abs}}$ is e.g. dependent upon the final-state size so that $\sigma ^{\psi '}_{\mathrm{abs}} \approx 4\sigma ^{J/\psi }_{\mathrm{abs}}$ [2007]. Color singlet quarkonium states are assumed to grow from their production point until they reach their asymptotic size, typically outside the nucleus [2008, 2009]. In this case, the survival probability is less than unity for rapidities where the state can hadronize in the interior of the nucleus but equal to unity for all rapidities where the state only reaches its final-state size outside the target. Color octet quarkonium states can interact strongly inside the target but, if they convert to the final color-singlet quarkonium state inside the target before interacting and dissociating, they will interact as singlets, giving a different suppression pattern. The color octet to singlet conversion depends on the proper time after production [2010, 2011] and is most important for rapidities which the quarkonium state can interact in the interior of the nucleus and, again, is inactive when the state hadronizes outside the nucleus.

Previous studies have shown the absorption cross section to depend on rapidity (or $x_\mathrm{F}$) as well as the nucleon–nucleon center of mass energy, $\sqrt{s_{NN}},$ with stronger absorption at lower energies [2012]. Increased effective absorption at backward rapidity may be due to interaction or conversion inside the target while increased effective absorption at forward rapidity may be due to energy loss. However, some finite value of $\sigma ^C_{\mathrm{abs}}$ is assumed for all rapidities. The $J/\psi$ has been most studied. Larger effects, at least at midrapidity, have been seen for the $\psi '$ [1999]. Such effects on $\Upsilon$ production may also be expected with stronger nuclear effects on the $\Upsilon (2S)$ and $\Upsilon (3S)$ relative to the $\Upsilon (1S)$ [2013].

Interactions with comovers, while first thought to be an important effect in $AA$ collisions [2014–2016], were later assumed to be small and, indeed, negligible [2017]. More recent data on $\psi '$ production as a function of the number of binary nucleon–nucleon collisions, $N_{\mathrm{coll}}$, in $\mathrm{d}+$Au collisions at $\sqrt{s_{NN}} = 200$ GeV shows a very strong dependence on $N_{\mathrm{coll}}$ for the $\psi '$ compared to almost no effect on the $J/\psi$ [2018]. Since the $\psi '$ mass is only ${\sim } 50$ MeV/$c^2$ below the $D \overline{D}$ threshold, interactions with comoving hadrons and/or partons could easily break up the $\psi '$ but not the $J/\psi$. Unfortunately the charmonium production rates have not been measured in conjunction with charged hadron multiplicity at RHIC. However, such data exist for $pp$ collisions at the LHC and show that the $J/\psi$ multiplicity increases with the charged particle multiplicity at both mid- and forward rapidity [2019]. If the $\psi '$ exhibits similar behavior, then one might further expect stronger $\psi '$ suppression in higher multiplicity (larger $N_{\mathrm{coll}}$) collisions.

*Energy loss theory* The theory of parton energy loss in hot matter has come a long way from the “jet quenching” predictions by Bjorken and others [2020] describing radiative energy loss by a fast parton. As discussed later in Sect. 6.4.4, experimentally the field has gone from studies of leading particle suppression at RHIC to true jet suppression at the LHC. Ongoing experimental studies address the influence of color charge and quark mass on the magnitude of the effect; the relative contributions of radiative and collisional (elastic) loss; the dependence on the thickness of the medium; and, in the case of jets, where the lost energy goes (related to the dependence on the jet cone radius). Here we describe some of the pQCD approaches to parton energy loss, some remaining open questions, and new approaches in the context of gravity dual theories.

The pQCD approaches have been summarized in detail in Ref. [2021]. They are known by a number of acronyms including AMY [2022, 2023], ASW [2024–2026], BDMPS [2027–2031], DGLV [2032–2034], HT [2035, 2036] and WHDG [1872]. They differ with respect to modeling the medium, the kinetic approximations taken into account, and the treatment of multiple gluon emission. We will briefly mention the differences; for full details, see Ref. [2021].

There are several ways of modeling the medium that the fast parton passes through. The simplest is to treat the medium as a collection of scattering centers with the parton undergoing multiple soft scatterings. A particular approach in this treatment is the opacity expansion which depends on the density of scattering centers (or, equivalently, the parton mean-free path) and the Debye screening mass. This expansion includes the power-law tail of the QCD scattering cross section, resulting in shorter formation times for the radiation compared to multiple soft scatterings alone. The medium has also been characterized by matrix elements of gauge field operators, in particular in the higher-twist approach. These higher-twist matrix elements are factorized into the nuclear parton densities and matrix elements describing the interaction of the partons with the medium in terms of expectation values of field correlation functions. Finally, the medium has been formulated as a weakly coupled system in thermal equilibrium. In this case, all the properties are specified by the temperature and baryon chemical potential. This approach is really valid only in the high temperature regime, $T \gg T_\mathrm{c}$.

All the approaches, however, make similar assumptions about the kinematics of the medium. They assume that the initial parton and the radiated gluon follow eikonal trajectories with both the parton energy, $E$, and the emitted gluon energy, $\omega$, much greater than the transverse momentum exchanged with the medium, $q_\mathrm{T}$: $E \gg q_\mathrm{T}$, $\omega \gg q_\mathrm{T}$. They also assume that the gluon energy is much larger than its transverse momentum, $k_{T}$: $\omega \gg k_{T}$. In the case of massive quarks, this constraint leads to the “dead-cone” effect where gluon radiation is suppressed for angles where $k_\mathrm{T}/\omega < M/E$ [1967]. Finally, they all assume some sort of localized momentum transfer with a mean-free path much larger than the screening length: $\lambda \gg 1/\mu _D$.

Multiple gluon emission is treated differently in the models. Some assume a Poisson probability distribution for the number of emitted gluons with an energy distribution following a single gluon emission kernel. This procedure can lead to a distribution of energy loss that does not conserve energy if the degradation of the parent parton momentum is not dynamically updated. Interference between medium-induced and vacuum radiation is included but the parton fragments in vacuum. Other approaches take a coupled evolution procedure with rate equations or medium-modified DGLAP evolution. The emission probability changes as the jet energy degrades, decreasing the path length through the medium.

In most approaches, the energy loss is characterized by the transport coefficient $\hat{q}$, the mean of the squared transverse momentum exchanged with the medium per unit path length. The pQCD approaches described above were compared and contrasted for the simplified “brick” problem in Ref. [2021]. This problem involves a uniform, finite block of quark–gluon plasma surrounded by vacuum. The goal was to study the energy lost by a high-energy parton produced inside the brick which travels a distance $L \sim 2$ fm through it before exiting into the vacuum. This setup provides a useful test bed for model comparison because it separates the conceptual differences from other complications inherent in heavy-ion collisions, such as modeling the hydrodynamic flow. The aim was to develop a “master” formalism which could reproduce all other representations in limiting cases. Thus each group’s results could be reproduced by turning approximations on and off, making it possible to examine the physical processes occurring as well as quantitatively assess which approximations are the most robust. This goal has not quite been achieved, though some progress has been made.

There are several technical issues not mentioned previously that need to be taken into account when comparing models. The first is the approximation that bremsstrahlung radiation (and/or pair production) is nearly collinear to the initial high-energy parton. This may, however, not always be the case in relevant situations [2021] which may be sensitive to soft and non-collinear gluon bremsstrahlung. Some more recent formulations have incorporated non-collinear radiation and have at least roughly accounted for the accompanying kinematic constraints. However a universal treatment is still lacking.

Next, systematic organization of corrections to energy loss calculations has not yet been achieved. An illustration of this is the Landau–Pomeranchuk-Migdal (LPM) effect, which accounts for the difference between the gluon formation time and the time between scatterings in the medium. In a dense medium, a high-energy parton undergoes multiple scatterings before a bremsstrahlung gluon forms. While the treatment of the LPM effect in the collinear approximation is understood (it diagrammatically corresponds to the resummation of an infinite class of diagrams), the systematization of corrections to these calculations order-by-order in perturbation theory is unknown. There have been some recent attempts to organize these corrections by employing Soft Collinear Effective Theory [2037–2039], but it has not yet been accomplished. In the same framework as [2040] a gauge invariant definition of the jet quenching parameter has been obtained, making it possible to relate it to the quark–antiquark static potential [2040, 2041]. Recently a first step towards calculating jet quenching via lattice simulation has been undertaken [2042].

We now turn to a somewhat more fundamental issue concerning these calculations. Most derivations of jet energy loss assume that the coupling between the initial high-energy parton and the two subsequent daughter partons is weak during high-energy bremsstrahlung or pair production: $\alpha _{s}(Q_\mathrm{T}) \ll 1$. The relevant scale in the coupling, $Q_\mathrm{T}$, is the transverse momentum between the two daughter partons. In thick media $Q_\mathrm{T}$ scales only weakly with the initial parton energy, $Q_\mathrm{T} \sim (\hat{q} E)^{1/4}$. The squared transverse momentum gained per unit length as the parton traverses the medium, $\hat{q}$, is, however, a characteristic of the medium. For realistic jet energies, $\alpha _{s}$ might indeed be relatively small but not very small. It is thus important to understand the size and nature of the corrections to the weak-coupling limit.

A QCD-like toy model in which the question of scales can be (and, indeed, has been) investigated is the large-$N_{c}$ $\mathcal{N} = 4$ SYM theory. As a warm-up for more complicated problems in jet energy loss, the stopping distance of a high-momentum excitation in the plasma can be calculated. For $\mathcal{N} = 4$ SYM and QCD, the answer is (up to logs) that the maximum stopping distance scales with energy as $E^{1/2}$, see Ref. [2043] for explicit QCD results. For $\mathcal{N}{=}4$ SYM, the calculation may, however, also be carried out at strong coupling. In this case, application of the AdS/CFT duality leads to an energy dependence of $E^{1/3}$. It is unknown how the $E^{1/2}$ dependence for weak coupling transforms to $E^{1/3}$ at strong coupling. Understanding this transition may also help understand how to treat the problem of small but not very small $\alpha _{s}(Q_\mathrm{T})$ of real QCD. It may well be that a key element in the resolution of this open puzzle will be an efficient use of effective field theory techniques.

In holographic investigations of energy loss, another particularly straightforward problem is the determination of the drag force felt by a heavy quark traversing a strongly coupled ${\mathcal N}=4$ SYM plasma [2044–2046]. In the simplest formulation of the problem [2044], the quark is represented by an open string hanging from the boundary, where the string endpoint, attached to a D-brane, is being pulled along a given spatial direction with constant velocity $v$. The equations of motion of the string are solved and the radial profile of the trailing string found as it moves through a black hole background representing the deconfined heat bath. The energy absorbed by the string is calculated and the drag force is found to scale with the square root of the ’t Hooft coupling.

Since the appearance of the original works on heavy quark energy loss at strong coupling [2044, 2047, 2048], the picture has been improved and expanded. An important development has been the study of the stochastic nature of the system analogous to the dynamics of heavy particles in a heat bath, giving rise to Brownian motion. This diffusive process was first considered in a holographic setting [2047], employing the Schwinger–Keldysh formalism. Subsequently, a study of the (quantum) fluctuations of the trailing string has provided information about heavy quark momentum broadening as it moves through the plasma [2049, 2050]. The stochastic motion has also been formulated as a Langevin process associated with the correlators of string fluctuations [2051, 2052]. These developments are closely related to the determination of transport coefficients in the holographic picture, see Sect. 6.2.3.

In most experiments, heavy quarks move at relativistic velocities. Therefore, it is necessary to also study the relativistic Langevin evolution of a trailing string in the $\mathcal{N}=4$ case [2053]. A similar study in non-conformal theories, in particular IHQCD, was performed in [2054, 2055].

Finally, a salient feature of the above picture involves the presence of a string world-sheet horizon with a Hawking temperature $T_\mathrm{s}$, distinct from that of the strongly coupled plasma. In the conformal case, $T_\mathrm{s} = T(1-v^2)^{1/4}\le T$ where $v$ is the velocity of the heavy quark. This temperature controls the world-sheet ensemble of the trailing string, which is not in thermal equilibrium with the surrounding plasma.

#### Quarkonium interaction at finite temperature and quarkonium suppression

Since the pioneering paper of Matsui and Satz [1968], the suppression of quarkonium in a hot medium has been considered one of the cleanest probes of deconfined matter, detected as a suppressed yield in the easily accessible dilepton decay channel, see e.g. Refs. [757, 1012]. However, quarkonium suppression as a diagnostic tool of hot media has turned out to be quite challenging for several reasons. On one hand, the effect has to be carefully disentangled from nuclear matter effects, discussed in the first subsection of Sect. 6.4.2, and from recombination effects (relevant at least for charmonium suppression in colliders, particularly at LHC, see the discussion in Sect. 6.4.4, in the quarkonium subsection). On the other hand, the level of quarkonium suppression measured in heavy-ion collisions has to be defined with respect to a clean baseline (at colliders, suppression has been investigated employing the nuclear modification factor $R_{AA}$, defined as the quarkonium yield in nucleus-nucleus collisions divided by the corresponding yield in $pp$, scaled by the number of binary collisions, see Sect. 6.4.4 for a discussion) and the contribution of decays from the excited states to lower-lying states has to be disentangled from the measured yield to extract the direct yield. Additionally, it is critical to understand the way that heavy quarks interact in the hot medium and what this brings to quarkonium suppression.

Originally, Matsui and Satz argued that, in a deconfined medium, the interaction between the heavy quark and the heavy antiquark would be screened, leading to the dissolution of the quarkonium state at a sufficiently high temperature. The naive expectation was that the static $Q\overline{Q}$ potential would be screened by $\exp \{- m_D (T) r\}$ where $m_D$ is the Debye mass, the temperature-dependent inverse of the screening length of the chromoelectric interaction and $r$ is the distance between the quark and antiquark. Thus quarkonia states would function as an effective thermometer for the medium, dissociating at different temperatures, depending on their radii. In particular, for temperatures above the transition temperature, $T_\mathrm{c}$, the range of the heavy quark interaction would become comparable to the bound state radius. Based on this general observation, one would expect that the charmonium states, as well as the excited bottomonium states, do not remain bound at temperatures above the deconfinement transition. This effect is referred to as quarkonium *dissociation* or quarkonium *melting*.

However until recently no proper tool for defining and calculating the quarkonium potential at finite $T$ had been developed. Most prior investigations were performed with phenomenological potentials inspired by lattice calculations of the $Q \overline{Q}$ free energy. The free energy was chosen because, in the zero-temperature limit, it coincides (up to small corrections) with the zero-temperature potential, while it flattens at finite $T$ and long distance, consistent with screening [2056–2058].

On the lattice, the free energy is extracted from the calculation of quark–antiquark Polyakov loop correlators. There are singlet and octet channels that are gauge dependent. An average gauge-independent free energy can also be defined. The three above-mentioned lattice free energies do not exhibit the same dependence on the $Q\overline{Q}$ separation distance and thus lead to different binding energies when used as phenomenological potentials in the Schrödinger equation [2059]. There are many papers in the literature either employing the singlet free energy or the corresponding internal energy as phenomenological potentials to calculate quarkonium binding energies at finite $T$ (see e.g. Refs. [2060, 2061]) or reconstructing the lattice meson correlation functions from the Schrödinger wave functions [2062] to understand which approach is better.

Lacking a comprehensive theoretical framework, other effects have often been included in addition to screening of the potential, such as the break up of the bound state by inelastic gluon collisions (gluodissociation) [2017, 2063–2065] or by light partons in the medium (quasi-free dissociation) [2060, 2066, 2067].

Information about the behavior of the quarkonium bound state at finite $T$ can also be obtained directly from the spectral function. On the lattice this quantity is accessible via calculations of the corresponding Green function employing the maximum entropy method (MEM) [2068, 2069] The challenges of this approach have been discussed in Sect. 6.2.3.

It is therefore very important to find a QCD-based theoretical framework that can provide a precise definition of the finite temperature $Q\overline{Q}$ potential and thus an unambiguous calculational tool. Such a definition has been obtained recently for weak-coupling through construction of appropriate effective field theories (EFT).

First [2070, 2071], the static potential was calculated in the regime $T \gg 1/r \gtrsim m_D$ by performing an analytical continuation of the Euclidean Wilson loop to real time. The calculation was done in weak-coupling resummed perturbation theory. The imaginary part of the gluon self-energy gives an imaginary part to the static $Q \overline{Q}$ potential and hence a thermal width to the quark–antiquark bound state (see also [2072]). Subsequently, an EFT framework for finite-temperature quarkonium in real time was developed [2073] (see [2074, 2075] for results in QED) working at small coupling $g$, $g T \ll T$, and for the velocity $v$ of the quark in the bound state of order $v \sim {\alpha _{\mathrm{s}}}$ (expected to be valid for tightly bound states: $\Upsilon (1S)$, $J/\psi$, $\ldots$).

The EFT description starts from the observation that quarkonium in a medium is characterized by different energy and momentum scales. As previously explained in Sect. 4.1.1, beyond the scales typical of nonrelativistic bound states ($m_Q$, the heavy quark mass; $m_Qv$, the scale of the typical inverse distance between the heavy quark and antiquark; $m_Qv^2$, the scale of the typical binding energy or potential energy and $\Lambda _\mathrm{QCD}$) there are thermodynamical scales ($T$, the temperature; $m_D$, the Debye mass, ${\sim } gT$ in the perturbative regime) and lower scales such as the magnetic scale that we neglect in the following.

If these scales are hierarchically ordered, physical observables can be systematically expanded in the ratio of such scales. At the level of the Lagrangian, this amounts to substituting QCD with a hierarchy of EFTs which are equivalent to QCD order-by-order in the expansion parameters. At zero temperature in Sect. 4.1.1, the two nonrelativistic EFTs that follow from QCD by integrating out the scales $m_Q$ (NRQCD) and $m_Qv$ (pNRQCD) have been discussed.

**Fig. 55:**
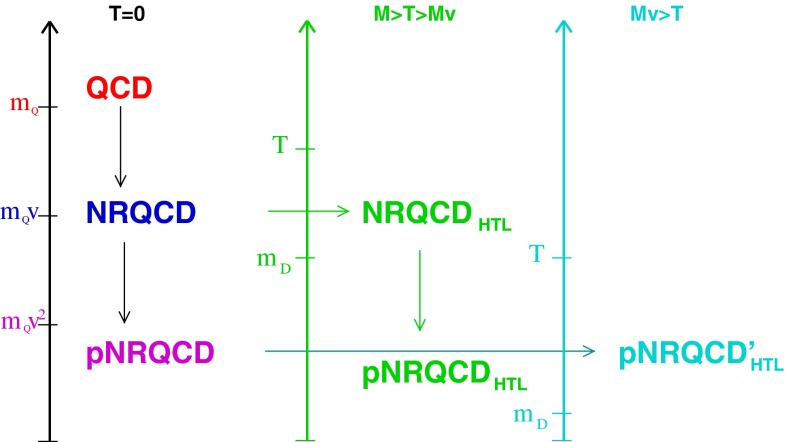
Hierarchies of EFTs for quarkonium at zero temperature (see Sect. 4.1.1 and Ref. [731]) and at finite temperature [2073–2077]. If $T$ is the next relevant scale after $m_Q$, then integrating out $T$ from NRQCD leads to an EFT called NRQCD$_\mathrm{HTL}$, because it contains the hard thermal loop (HTL) Lagrangian. Subsequently, integrating out the scale $m_Qv$ from NRQCD$_\mathrm{HTL}$ leads to a thermal version of pNRQCD called pNRQCD$_\mathrm{HTL}$. If the next relevant scale after $m_Q$ is $m_Qv$, then integrating $m_Qv$ out from NRQCD leads to pNRQCD. If the temperature is larger than $m_Qv^2$, then $T$ may be integrated out from pNRQCD, leading to a new version of pNRQCD$_\mathrm{HTL}$ [2076]. From [2078]

At finite $T$ different possibilities for the scale hierarchies arise. The corresponding EFTs are shown in Fig. 55.

In the EFT, the interaction potential $V$ is clearly defined and a structured power counting to calculate the quarkonium energy and width is provided. The potential follows from integrating out all contributions from modes with energy and momentum larger than the binding energy. For temperatures smaller than the binding energy the potential is simply the Coulomb potential. Thermal corrections affect the energy and induce a thermal width to the quarkonium state which may be relevant for describing the quarkonium in-medium modifications at relatively low temperatures. For temperatures larger than the binding energy, the potential acquires both real and imaginary thermal contributions.

This QCD-based description has resulted in a *paradigm shift* in our understanding of quarkonium properties in a weakly coupled plasma. The following pattern is observed [2070, 2071, 2073–2077]:

- The thermal part of the potential has a real and an imaginary part. The imaginary part of the potential smears out the bound state peaks of the quarkonium spectral function, leading to their dissolution at lower temperatures than those required for the onset of Debye screening in the real part of the potential (see, e.g. [2079]). Thus quarkonium dissociation appears to be a consequence of the appearance of a thermal decay width rather than being due to the color screening of the real part of the potential: the thermal decay width may become as large as the binding energy at a lower temperature than that at which color screening sets in.
- Two mechanisms contribute to the thermal decay width: the imaginary part of the gluon self-energy, induced by the Landau-damping phenomenon (also present in QED) [2070] and the quark–antiquark color singlet to color octet thermal break up (a new effect, specific to QCD) [2073]. These two mechanisms are related to the previously described gluodissociation [2017, 2063–2065] and quasi-free dissociation [2060, 2066, 2067], respectively. The EFT power counting establishes which dissociation mechanism dominates parametrically in which temperature regime. Landau damping dominates for temperatures where the Debye mass $m_D$ is larger than the binding energy $E_B$ while the singlet to octet break up dominates for $m_D < E_B$. The distinction between the two dissociation mechanisms holds at leading order. Both can be calculated by cutting appropriate diagrams in the relevant EFTs. See [2080, 2081] for results relating the quarkonium widths to the in-medium or vacuum cross sections that correct or complement the previously used approximations and phenomenological formulas.
- The resulting color singlet thermal potential, $V$, is neither the color-singlet quark–antiquark free energy [2082] nor the internal energy. It has an imaginary part and may contain divergences that eventually cancel in physical observables [2073].
- Temperature effects can be other than screening, typically they may appear as power law or logarithmic corrections [2073, 2074].
- The dissociation temperature behaves parametrically as $\pi T_\mathrm{melting} \sim m_Q g^{4/3}$ [2074, 2075, 2079].

In particular, in Ref. [2077] heavy quarkonium energy levels and decay widths in a quark–gluon plasma, at a temperature below the quarkonium melting temperature satisfying the hierarchy $m_Q \gg m_Q\alpha _\mathrm{s} \gg \pi T \gg m_Q\alpha _\mathrm{s}^2 \gg m_D$ have been calculated to order $m_Q \alpha _\mathrm{s}^5$. This hierarchy may be relevant for the lowest-lying bottomonium states ($\Upsilon (1S)$, $\eta _b$) at the LHC, for which it may hold: $m_b \approx 5 \hbox { GeV} > m_b\alpha _\mathrm{s} \approx 1.5~\hbox { GeV} > \pi T \approx 1 \hbox { GeV} > m_b\alpha _\mathrm{s}^2 \approx 0.5 ~ \hbox {GeV} \! \gtrsim m_D$. In this situation, the dissociation width grows linearly with temperature. Then the mechanism underlying the decay width is the color-singlet to color-octet thermal break-up, implying the tendency of quarkonium to decay into a continuum of color-octet states. This behavior [2075, 2077] is compatible with the data (($\Upsilon (1S)$ does not yet seem to be dissociated at LHC) and with finite $T$ NRQCD lattice calculations [2083–2085].

Even if the above-described theory holds only for weak coupling, it has had a more general impact on our understanding of the physics since, for the first time, it provides a coherent, systematic theoretical framework. A key feature of the potential obtained in this picture is that it contains a sizable imaginary part encoding the decoherence effects caused by interactions with the medium. The impact of such an imaginary part has been studied [2086, 2087] but a fully consistent phenomenological description of quarkonium suppression is yet to appear. Additional effects that are just beginning to be considered are the effect of an anisotropic medium [2088–2090] and the relative velocity between the quarkonium state and the medium [2091–2094].

The next step would be to generalize these results to strong coupling. Initial investigations have been made recently on the lattice [2095, 2096] but a complete EFT description is still lacking. Preliminary work includes study of the Polyakov loop and Wilson loop correlators and their relation to singlet and octet correlators in perturbation theory [2082] and in general. The non-trivial renormalization properties of the cyclic Wilson loop have been investigated [2097, 2098], making it possible to determine which combinations of correlators are suitable for lattice calculations.

It may be possible to calculate the behavior of the potential at strong-coupling using holographic correspondence. However, the imaginary part of the potential, responsible for the thermal decay width, was not predicted in AdS/CFT-inspired calculations. After this effect was identified in perturbative calculations [2099–2101], it was also obtained using holographic methods.

Some of the outstanding questions in quarkonium theory include whether quarkonium and heavy quarks are indeed external probes of the medium; the connection of the magnitude of their flow and the diffusion coefficients in EFTs; and quantification of the importance of recombination effects. The experimental state of the art regarding these questions is discussed in the quarkonium subsection of Sect. 6.4.4.

#### Experimental results on hard probes

The details of the production and propagation of high $p_{T}$ and high mass probes can explore the mechanisms of parton energy loss and deconfinement in the medium and shed light on the relevant physical mechanisms and the microscopic properties of the medium. In addition, the underlying event, even if considered as a background contribution to the hard probes, is an important element of the hadronic environment consisting of complex contributions, spanning over non-perturbative and perturbative QCD and including sensitivities to multiscale and low $x$ physics.

Experimentally, several methods are used to address such questions, generally through comparison of the relative production of single particles or fully reconstructed jets in nuclear collisions to expectations from a superposition of independent nucleon–nucleon collisions.

In particular, jet production is decoupled from the formation of the medium and can be considered an external probe traversing the hot medium. Due to their early production, jets are well calibrated probes: the production rates can be calculated using pQCD in the vacuum because their large energy scale minimizes cold nuclear matter effects.

At the LHC, high-$p_{T}$ hadron production is dominated by gluon fragmentation. The gluons have a larger color-coupling than light quarks, thus gluon energy loss is expected to be larger. Moreover, heavy quarks with $p_{T}$ lower than or equivalent to the quark mass should have less gluon radiation and thus a smaller suppression than light quarks. This is discussed further in the subsection dedicated to heavy flavors.

To quantify suppression effects, the nuclear modification factor, $R_{AA}$, is widely used. It is defined as the ratio of yields in $AA$ collisions to those in $pp$, scaled by the number of binary collisions,

6.3 $$\begin{aligned} R_{AA} = \frac{(1/N_{\mathrm{evt.}}^{AA})\mathrm{d}^2N_{\mathrm{ch}}^{AA}/dp_{{T}}d\eta }{\langle N_{\mathrm{coll}} \rangle (1/N_{\mathrm{evt.}}^{pp}) \mathrm{d}^2N_{\mathrm{ch}}^{pp}/dp_{T}d\eta } \, \, , \end{aligned}$$

where the average number of binary nucleon–nucleon collisions, $\langle N_{\mathrm{coll}} \rangle$, is given by the product of the nuclear overlap function, $T_{AA}$, calculated in the Glauber model [1869], and the inelastic $NN$ cross section, $\sigma _{\mathrm{in}}^{NN}$. The collision centrality is often quantified in terms of the number of nucleon participants, $N_{\mathrm{part}}$, also calculated in the same Glauber framework. In the absence of nuclear effects, $R_{AA}$ is unity by construction. In addition to $R_{AA}$, the quenching effects can be quantified using the central-to-peripheral ratio, $R_{CP}$, defined as the ratio of the per-event yield in a given centrality bin normalized by the number of $NN$ collisions in the same centrality bin to the same quantity in a more peripheral bin, typically 60–80 %.

Differential measurements include: $\gamma$+jet, hadron+jet, and dijet spectra; angular correlations; azimuthal anisotropies; jet shapes and fragmentation functions. Measurements of the azimuthal anisotropy, $v_2$, can probe thermalization at low $p_{T}$, while at high $p_{T}$ the path length dependence of energy loss can be studied. The measurement of the reaction plane allows more differential measurements such as the study of $R_{AA}$ “in-” and “out-of-plane” (i.e., along the short and long axes of the almond-shaped overlap region of the two nuclei in semi-central collisions). Azimuthal spectra of dijet events in different centrality bins as well as separation of leading and sub-leading jets all allow further insight into the path length dependence of energy loss and the redistribution of the quenched jet energy.

**Fig. 56:**
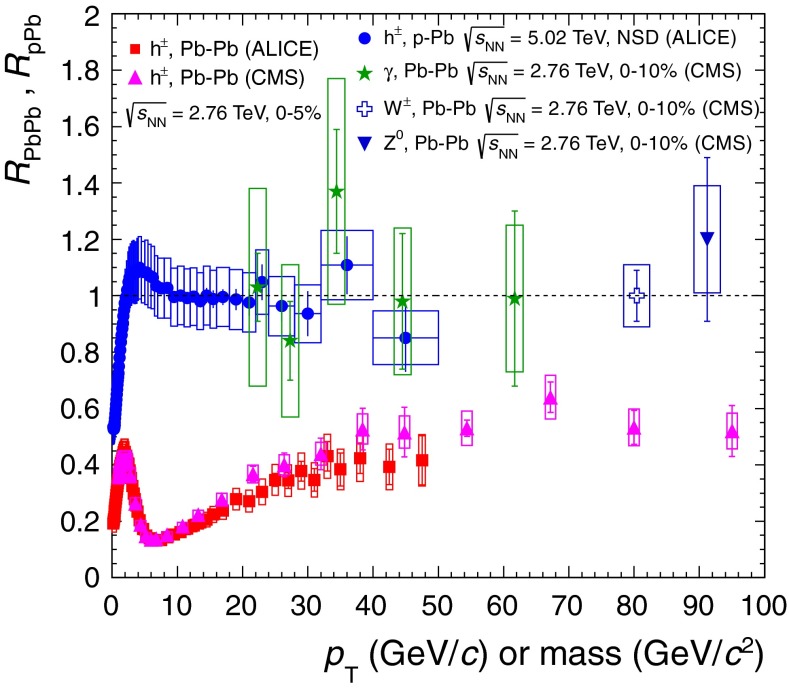
The $R_{AA}$ for charged particles in the 5 % most central Pb+Pb collisions at $\sqrt{s_{NN}} = 2.76$ TeV is compared for ALICE and CMS [2104]. The results are also compared to those for $W$and $Z$ bosons as well as isolated photons measured by CMS [1990, 1991, 2107]. The $R_{p\mathrm{Pb}}$ for $p$+Pb collisions at $\sqrt{s_{NN}} = 5.02$ TeV measured by ALICE is also shown [2102, 2103]

*High* $p_{T}$ *observables* *a. Charged hadrons and bosons* Inclusive measurements can give the first indication of the existence of a hot and dense medium. One of the most complete pictures of interactions of hadrons and electroweak bosons with the medium is shown in Fig. 56 for the charged particle $R_{AA}$ in central Pb+Pb collisions at $\sqrt{s_{NN}} = 2.76$ TeV [2102–2104] compared to the $R_{AA}$ of $W$, $Z$ [1990, 1991, 2105, 2106] and isolated photons [2107] at the same energy. The charged particle $R_{p\mathrm{Pb}}$ from $p$+Pb collisions at $\sqrt{s_{NN}} = 5.02$ TeV is also shown [2108]. Understanding the detailed structure of these ratios is the subject of intense discussions among theorists and experimentalists [2109].

The peak in $R_{\mathrm{PbPb}}^{\mathrm{ch}}$ at $p_{{T}} \approx 2$ GeV/$c$ can be interpreted as a manifestation of radial collective flow [1736]. Energy loss causes a pile up at low $p_{T}$ which is enhanced by flow. This is also supported by the results obtained looking at identified hadrons and their mass ordering effects, as discussed later. At $p_{T} \approx$ 5–7 GeV/$c$, $R_\mathrm{PbPb}^{\mathrm{ch}}$ falls to a minimum of $\approx 0.13$, lower than the minimum at RHIC [2110] of $R_{\mathrm{AA}}^{\mathrm{ch}} \approx 0.2$, indicating a slightly larger suppression at the LHC. Above 7 GeV/$c$, $R_{AA}$ increases to $\approx 0.4$ at $p_{T} > 30$ GeV/$c$ and remains relatively constant thereafter, showing that the medium can quench even very high $p_{T}$ particles. One possible explanation is that a constant energy loss shifts the entire $p_{T}$ spectrum to lower $p_{T}$. In general, the low $p_{T}$ region reflects an interplay of soft physics effects (shadowing, saturation, Cronin, flow, etc.) which are still under investigation [1947].

The LHC measurements confirm and extend the experimental signatures of partonic energy loss first observed in the 5 % most central Au+Au collisions at $\sqrt{s_{NN}} = 200$ GeV at RHIC [1723, 2111] where the measured signals include suppression of single hadrons and modification of dihadron angular correlations [2112]. At the LHC, hadron production cross sections are several orders of magnitude higher that those at RHIC, allowing measurements over a wider $p_{T}$ range and giving access to multi-dimensional studies of cross-correlated observables.

The $R_{AA}$ distributions have also been compared to model calculations employing the RHIC data to calibrate the medium density. They implement several different energy loss mechanisms [1873, 2021, 2025, 2113–2116]. Some of them can qualitatively reproduce the increase of $R_{AA}$ with $p_{T}$. This rise can be understood as a decrease of the fractional energy loss of the parton with increasing $p_{T}$, reflecting the weak dependence of pQCD radiative energy loss on parton energy. The observed trend is semi-quantitatively described by several models of QCD energy loss. The differences between the results presented in Refs. [1873, 2021, 2025, 2113–2116] and elsewhere are under systematic investigation. They may arise from poorly controlled aspects of leading-order collinear gluon radiation. A complete picture of energy loss at next-to-leading order is under study but difficult to achieve.

Further details and open questions related to the theory of energy loss are discussed in Sect. 6.4.2.

The measurements in Fig. 56 also show that isolated photons and $W$ and $Z$ bosons, which do not carry color charge, are not suppressed. This is consistent with the hypothesis that the observed charged hadron suppression is due to final-state interactions with the hot and dense medium. Further input comes from the $p$+Pb data which were expected to distinguish initial- from final-state effects, as discussed in Sect. 6.4.2. First results of $R_{p\mathrm{Pb}}$ data from the $p$+Pb pilot run at $\sqrt{s_{NN}} = 5.02$ TeV [2102, 2103] are compared to the Pb+Pb results in Fig. 56. The $p$+Pb measurement was performed for non single diffractive collisions in the pseudorapidity range $|\eta _{\mathrm{cms}}|<0.3$. In this minimum-bias sample, with no further constraints on multiplicity, the data show no strong deviation from scaling with the number of binary nucleon–nucleon collisions. This is in agreement with the hypothesis that the strong suppression of hadron production at high $p_{T}$ observed in central Pb+Pb collisions is not due to initial-state effects, supporting the production of hot quark–gluon matter in Pb+Pb collisions [2102, 2103].

The observed trends qualitatively resemble those of $R_\mathrm{dAu}$ at RHIC. At low $p_{T}$, suppression may be related to parton shadowing or saturation while the rise at $p_{T} \approx 4$ GeV/$c$ may be a manifestation of the Cronin effect which originates from multiple scattering during the initial phase of the collision.

However, further extensive analysis of the LHC data reveal different aspects. In ATLAS, per-event inclusive hadron yields were measured in different centrality and rapidity regions, demonstrating strong dependence of the Cronin peak not only on centrality, but also on rapidity [2117]. Measurements with fully reconstructed jets reveals a strong reduction of the jet yield in the proton-going direction in more central collisions relative to peripheral collisions [2118]. The reduction becomes more pronounced with increasing jet $p_{T}$ and at more forward proton-going rapidities. When the jet $R_{CP}$ is measured as function of the full jet momentum, $p_{T} \cosh y$, the rapidity variation factors out, reducing the $R_{AA}$ measured in all rapidity intervals to a single curve.

Results from CMS [1746, 2119] extend the charged particle $R_{p\mathrm{Pb}}$ up to $p_{T} \approx 130$ GeV/$c$. The value of $R_{p\mathrm{Pb}}$ rises above unity for $p_{T} > 30$ GeV/$c$, near the onset of the gluon antishadowing region but significantly larger than predicted.

Furthermore, the data reveal different trends when the measurements are performed in the low- or high-multiplicity samples. Additional intriguing features, observed for high-multiplicity events, are discussed in Sect. 6.5. In particular, indications of collective behavior are seen for several distributions in the high-multiplicity sample. Currently this is a puzzle that is actively being pursued both by experimentalists and theorists.

*b. Identified hadrons* In order to set additional constraints on energy loss, the nuclear suppression factor has been studied for identified particles. At the LHC, measurements of identified particle $R_{AA}$ include light and strange hadrons, isolated photons, $Z$, $W$, $D$, $J/\psi$ and $\Upsilon$. The suppression of individually reconstructed prompt and nonprompt $J/\psi$ (from $B$ decays, identified by displaced vertex techniques) is discussed further in the subsection dedicated to heavy flavor.

**Fig. 57:**
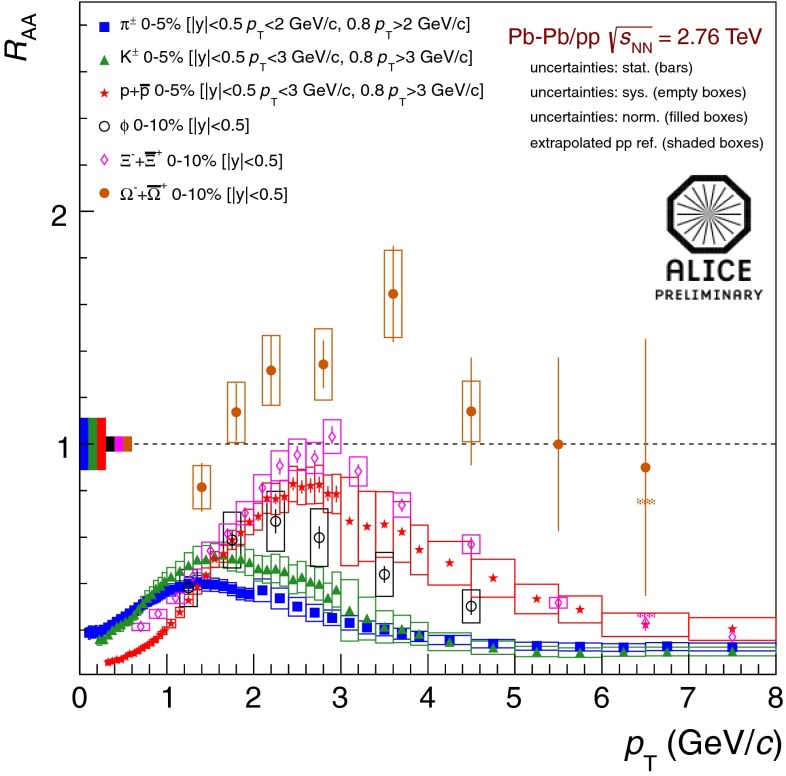
Nuclear modification factors $R_{AA}$  at midrapidity versus $p_{T}$ and centrality, for light and strange particles: pion, kaon, proton, $\phi$, $\Xi$ and $\Omega$. The measurement of pions, kaons and protons at $p_T > 3$ GeV/$c$ is in the rapidity window $|y| < 0.8$. From [2120]

The ALICE $R_{AA}$ for identified pions, kaons and protons up to $p_{T} \approx 20$ GeV/$c$, confirms the observations at RHIC and shows that a hierarchy of suppression is observed at low $p_{T}$. In order to better understand the influence of rescattering effects, $R_{AA}$s for resonances and stable hadrons have also been measured, Fig. 57. Of particular interest is the $\phi (s \overline{s})$ measurement since the $\phi$ meson, with a mass similar to that of the proton, can discriminate effects due to mass and quark content. The $\phi$ $R_{AA}$ appears to follow the strange baryon $R_{AA}$ of $\Xi$ and $\Omega$ for $p_T \le 2.5$ GeV/$c$, and lies between the $R_{AA}$ of light mesons ($\pi$ and $K$) and $R_{AA}$ of baryons ($p$ and $\Xi$) at high $p_T$ [2120]. More generally, the meson results cluster around a lower value of $R_{AA}$ than the protons, reflecting strong radial flow [1736]. For $p_{T} \ge$ 8–10 GeV/$c$, the suppression seems to be the same for different particle species, indicating that the medium effects are similar for all light hadrons.

A detailed systematic study of charged hadron spectra and $R_{AA}$ as a function of centrality was also carried out for Au+Au and d$+$Au collisions at $\sqrt{s_{NN}} = 200$  GeV [2110]. Baryon enhancement is present in both systems. In d$+$Au collisions, the Cronin enhancement has long been known to be stronger for baryons than for mesons. However, for the first time the results present clear evidence for a strong centrality dependence of this effect. In Au+Au collisions, the baryon enhancement has been attributed to parton recombination at hadronization. When combined with the mass dependence of $v_2$ measured at the LHC, there is a strong indication that the mass effect observed in $p$+Pb collisions has a collective final-state origin. A similar but weaker effect was also observed by PHENIX [1736, 1746]. In general, the measurements of identified hadrons over a wide $p_{T}$ range, have also the potential to address modifications of the jet fragmentation functions.

**Fig. 58:**
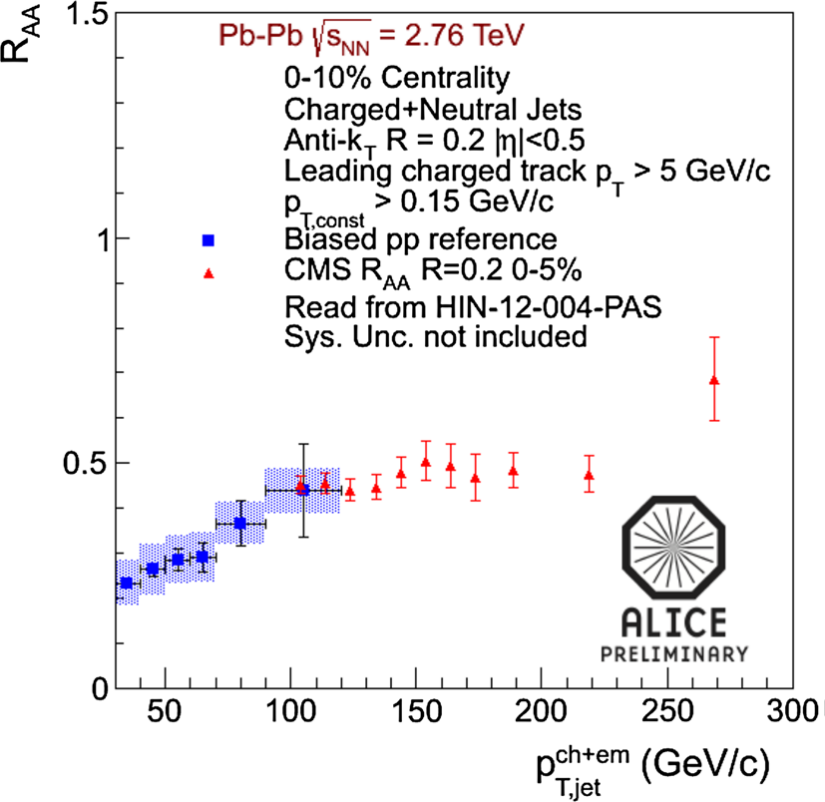
The jet $R_{AA}$ over a wide $p_{T}$ range measured by ALICE and CMS in central Pb+Pb collisions at $\sqrt{s_{NN}} = 2.76$ TeV. Data from ALICE [2124] and CMS [2122]; plot from [2125]

*c. Reconstructed jets* Fully reconstructed jets available over a wide $p_{T}$ range at $\sqrt{s_{{NN}}}=2.76$ TeV at the LHC confirm and extend the suppression pattern observed for charged particles. Figure 58 presents the ALICE $R_{AA}$ results covering low $p_{T}$, down to $\approx$ 30–40 GeV/$c$ [2121], and the CMS measurements [2122] up to $p_{T} \approx 270$ GeV/$c$. Good agreement is observed in the overlapping $p_{T}$ region. Similar results have been obtained by the ATLAS Collaboration [2123].

The complementarity of these results, together with combined systematic studies over the widest available $p_{T}$ range and employment of particle identification at low $p_{T}$ explore different aspects of energy loss. Note that, although the original parton energy is better reconstructed in a jet than by tagging only a fast hadron, the single inclusive jet suppression is similar to that of single hadrons. This can be understood if parton energy loss is predominantly through radiation outside the jet cone radius used in the jet reconstruction algorithm.

Jet reconstruction in heavy-ion collisions is challenging due to the high-multiplicity environment. However, dedicated algorithms and background subtraction techniques have been optimized to reconstruct all the particles resulting from the hadronization of the parton along its trajectory within a fixed jet-cone radius [2126, 2127].

In Pb+Pb collisions, the strongest jet suppression is observed for the most central events. A clear centrality dependence is observed in successively peripheral events with decreased suppression (larger $R_{AA}$) in peripheral collisions. In particular, imposing a minimum fragmentation bias on single tracks of 0.150 GeV/$c$, ALICE explored the low $p_{T}$ region (30–110 GeV/$c$) [2121] finding $R_{AA} \sim 0.4$ for a jet cone radius $R = 0.3$. At higher $p_{T}$, ${\sim } 200$ GeV/$c$ for ATLAS [2123] and ${\sim } 300$ GeV/$c$ for CMS, $R_{{AA}} =0.5$, almost independent of jet $p_{T}$. These results imply that the full jet energy cannot be captured for $R<0.3$ in heavy-ion collisions.

**Fig. 59:**
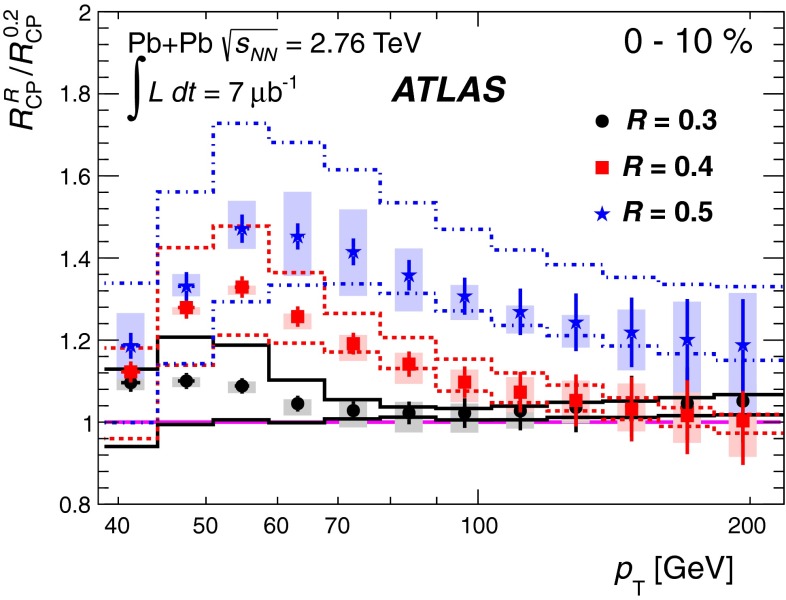
The ratios $R_{CP}^R/R_{CP}^{R=0.2}$ for $R=0.3$, $0.4$ and $0.5$ as a function of jet $p_{T}$ in the 0–10 % centrality bin. The *bars* show the statistical uncertainties, the *lines* indicate fully correlated uncertainties and the *shaded boxes* represent partially correlated uncertainties between different $p_{T}$ values. From [2123]

The same conclusion can be reached by studying the jet $R_{CP}$, as shown in Fig. 59. For $p_{T} < 100$ GeV/$c$, the ratio $R_{{CP}}^R/R_{{CP}}^{{R=0.2}}$, for $R = 0.4$ and 0.5, differs from unity beyond the statistical and systematic uncertainties, indicating a clear jet broadening. However, for $R \le 0.4$ at $p_{T} > 100$ GeV/$c$, the ratio is consistent with jet production in vacuum over all centralities. This may be interpreted as an indication that the jet core remains intact with no significant jet broadening observed within the jet cone resolution [2123].

**Fig. 60:**
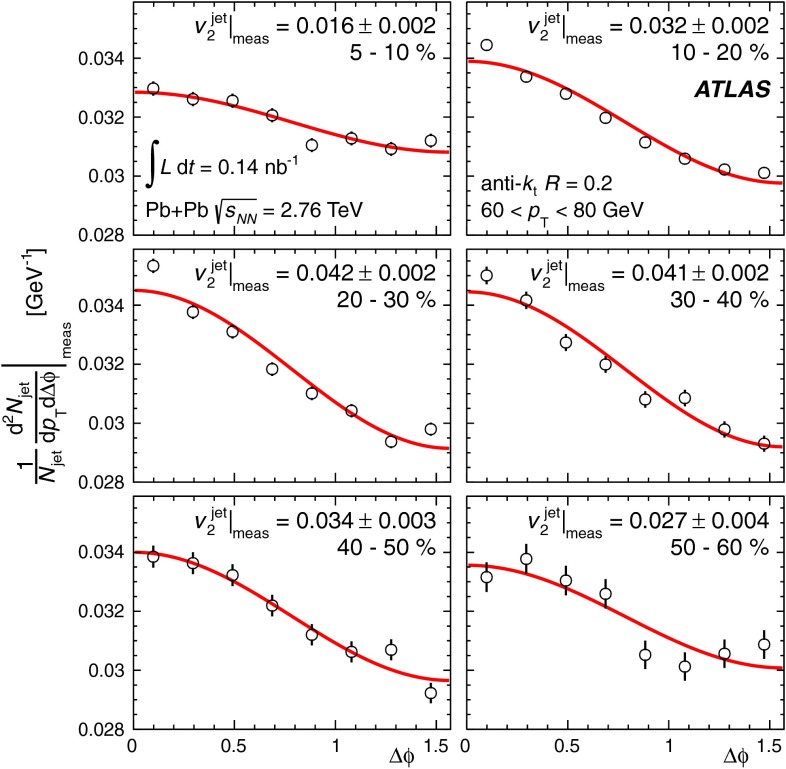
The $\Delta \phi$ dependence of measured jet yield in the 60 $<$ $p_{T}$ $<$ 80 GeV/c interval for six ranges of collision centrality. The yields are normalized by the total number of jets in the $p_{T}$ interval. The *solid curves* are a fit to the data. From [2128]

**Fig. 61:**
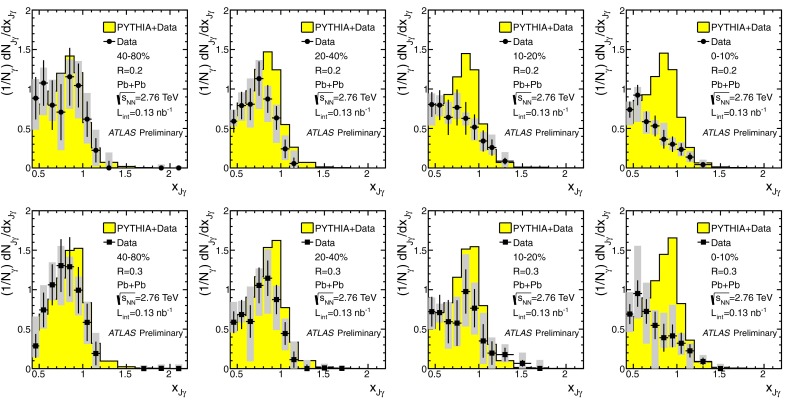
The distribution of the mean fractional energy carried by a jet opposite an isolated photon, $x_\mathrm{{J\gamma }}$, in Pb+Pb collisions (*closed symbols*) compared with PYTHIA “true jet”/“true photon” distributions (*yellow histogram*) embedded into simulated background heavy-ion events. The rows represent jet cone radii $R=0.2$ (*top*) and $R=0.3$ (*bottom*). The *columns* represent different centralities with increasing centrality from left to right. The *error bars* represent statistical errors while the *gray bands* indicate the systematic uncertainties. From [2130]

*d. Path length dependence of the energy loss* Measurements of inclusive jet suppression as a function of azimuthal separation with respect to the event plane, $\Delta \phi$, makes possible an estimate of the path-length dependence of energy loss for the first time. A measurement of the variation of the jet yield as a function of the distance traversed through the matter can provide a direct constraint on the relative theoretical models. Figure 60 shows the variations in the jet yield as a function of $\Delta \phi$ at different centralities for $60 < p_{T} < 80$ GeV/$c$ for fully reconstructed jets measured by ATLAS  [2128]. The observed azimuthal variation amounts to a reduction of 10–20 % in the jet yields between in-plane and out-of-plane directions establishing a clear relationship between jet suppression and the initial nuclear geometry and confirming that jet suppression is stronger in the direction where the parton traverses the greatest amount of hot medium.

The azimuthal anisotropy of charged particles with respect to the event plane has been studied by CMS over the widest $p_{T}$ range, up to ${\sim } 60$ GeV/$c$. The results [2105] show a rapid rise of the anisotropy to a maximum at $p_{T} \sim 3$ GeV/$c$ with a subsequent decrease in all centrality and $\eta$ ranges. A common trend in the centrality dependence is observed over a wide $p_{T}$ range, suggesting a potential connection to the initial-state geometry.

*e. Correlations* Inclusive jet measurements provide only limited information because the initial jet energy is unknown. The magnitude of the energy lost by jets can be measured by studying boson-jet correlations, assuming that the boson momentum represents the initial jet momentum. As already shown in Fig. 56, the electroweak gauge bosons, which do not carry color charge, are unaffected by the medium and therefore retain the kinematics of the initial hard scattering [1990, 1991, 2107]. This suggests that identifying the correlations between isolated photons and jets is one of the key methods of determining the energy of the parton which generated the jet [2129, 2130]. Measurements of the photon [1991, 2107], $Z$ [2108] and $W$ [2131] production rates are shown to scale with the nuclear overlap function, $T_{AA}$, in Fig. 56. In addition the shapes of the $p_{T}$ and rapidity distributions are unmodified in Pb+Pb collisions. Updated $R_{AA}$ measurements for bosons at higher statistics and in various decay channels were presented in [1746]. As an example, Fig. 61 shows the mean fractional energy distribution carried by the jet opposite a photon, $x_\mathrm{{J\gamma }}$, in Pb+Pb collisions [2130] compared to PYTHIA simulations (yellow histogram) embedded into simulated background heavy-ion events. As the centrality increases, the distribution shifts toward smaller $x_\mathrm{{J\gamma }}$, suggesting that more and more of the jet momentum distribution falls below a minimum $x_\mathrm{{J\gamma }}$. In contrast, the PYTHIA ratio of the “true jet” to “true photon” distribution exhibits no centrality dependence. Similar results are obtained from CMS with photon+jet events [2129] and from ATLAS for $Z$+jet [2132] and confirmed in [1746] with higher statistics.

**Fig. 62:**
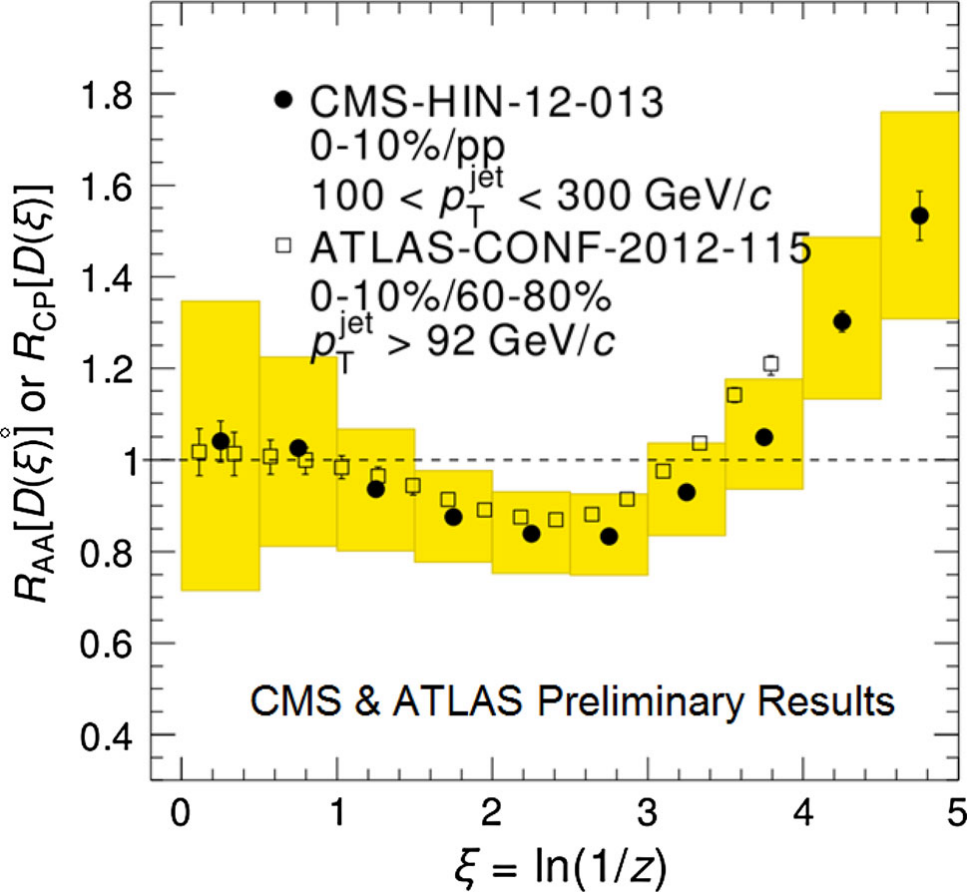
The ratio of jet fragmentation functions measured in Pb+Pb and $pp$ collisions in two centrality bins as a function of the scaling variable $\xi =\ln (1/z)$, with $z = (p^\mathrm{track}_{\parallel }/p^\mathrm{jet})$ where $p^{track}_{\parallel }$ is the momentum component of the track along the jet axis, and $p^{jet}$ is the magnitude of the jet momentum. Data from CMS [2134] and ATLAS [2135]; plot from [2136]

*f. Jet fragmentation* Jet structure in the medium has been studied through the fragmentation functions and dijet transverse momentum imbalance by means of hard momentum cuts on charged particles at $p_{T}$ $>$ 4 GeV/$c$ and jet cone radii $R<0.3$. Figure 62 shows the ratios of the fragmentation functions measured in Pb+Pb and $pp$ collisions at $\sqrt{s_{NN}} = 2.76$ TeV. The fragmentation is measured with respect to the final-state jet momentum (after energy loss). The results show that the longitudinal structure of the jet does not change in the high $p_{T}$ $>$ 100 GeV/$c$ region where the measurement has been performed [2133–2135]. However, the trend suggests a softening of the fragmentation function in the most central collisions if softer particles ($p_{T}$ $>$ 1 GeV/$c$) are included [2134].

In addition to the longitudinal structure of the jet, its transverse structure can also be studied. In central Pb+Pb events a significant shift of the transverse momentum imbalance of the leading jet and its recoil partner is observed for $\Delta \phi _{1,2}> 2\pi /3$ with respect to $pp$ collisions. The shift, which changes monotonically with centrality, does not show a significant dependence on the leading jet $p_{T}$ [2137]. The implication for the absolute magnitude of energy loss should be extracted employing realistic models [2137].

**Fig. 63:**
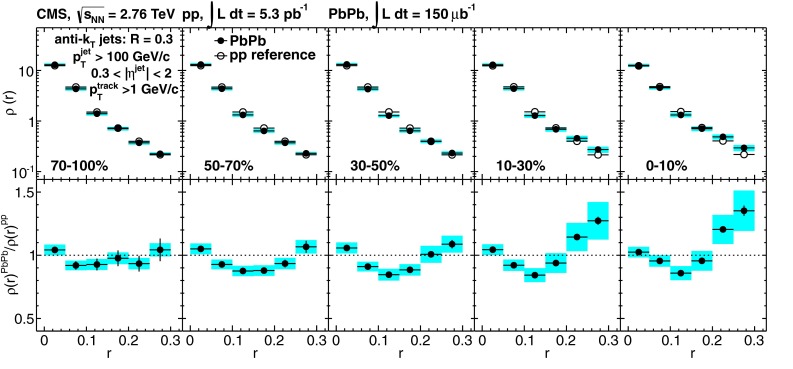
The differential jet shapes, $\rho (r)$, in Pb+Pb and $pp$ collisions determined by CMS shown as a function of the annular regions in the jet cone $r$, in steps of $\Delta R$ = 0.05. The Pb+Pb data are shown as the filled points while the open circles show the $pp$ reference. In the *bottom panel*, the ratio of the Pb+Pb to $pp$ jet shapes is shown for annular regions in the jet cone, from the center to the edge of the jet cone radius $R$. The *band* represents the total systematic uncertainty. From [2138]

*g. Jet structure* The QGP is expected to modify the jet shape both because of parton interactions with the medium and because soft particle production in the underlying event adds more particles to the jet. Thus the energy flow inside a jet, sensitive to the characteristics of the medium traversed by the jet, can be studied through jet shape analysis which should then widen due to quenching effects. CMS has measured the average fraction of the jet transverse momentum within annular regions of $\Delta R = 0.05$ from the inner part of the jet to the edge of the jet cone. Correcting for the underlying event and all instrumental effects in central collisions, moderate jet broadening in the medium is observed for $R = 0.3$ [2138]. The effect increases for more central collisions. This is consistent with the concept that energy lost by jets is redistributed at large distances from the jet axis, outside the jet cone, see Fig. 63. For an update on theoretical developments at high $p_{T}$, see Ref. [2109].

As discussed previously, the structure of high energy jets at the LHC is unmodified: the radiated energy is carried by low $p_{T}$ particles a large distance away from the jet axis [2139]. Models suggest different behaviors within the jet core and outer regions of the cone due to the different couplings to the longitudinally-flowing medium or to turbulent color field, leading to eccentric jet structure. ALICE extended the study of the centrality dependence of shape evolution in the near-side correlation peak to the low and intermediate $p_{T}$ regions by measuring the width of the peak in the $\varDelta \eta$ (longitudinal) and $\Delta \phi$ (azimuthal) directions [2140]. The width in $\Delta \eta$ shows a strong centrality dependence, increasing by a factor $\approx 1.6$ from peripheral to central Pb+Pb events, while the width in $\Delta \phi$ is almost independent of centrality. The AMPT model calculations [1937, 2141], which take into account collective phenomena, exhibit similar behavior, indicating that the observed effects reflect collectivity. Such behavior is expected in models taking into account interaction of the fragmenting jet with the longitudinally flowing medium which distorts a jet produced with an initial conical profile [2142].

To further study the interplay of soft (flow) and hard processes (jets) and how they affect hadrochemistry, the particle composition in jet-like structures was investigated by ALICE. They studied the $p/\pi$ ratio in $\Delta \eta$–$\Delta \phi$ space relative to a trigger particle. It is found that in the “near-side” peak region, the ratio is consistent with expectations from $pp$ collisions, estimated using PYTHIA. In the “bulk” region, the ratio is compatible with that obtained for non-triggered events, a factor of 3–4 increase compared to $pp$. The hadrochemistry result suggests that there is no significant medium-induced modification of particle ratios within jets and the enhancement of the inclusive $p/\pi$ ratio observed in minimum bias Pb+Pb collisions is a result of bulk processes and not jet fragmentation [2143].

*Heavy flavors* Because heavy quarks are produced in the very early stage of the collision, they probe the properties of the QCD medium while traversing it. Open heavy-flavor measurements are used to investigate details of the energy loss, thermalization, and the hadronization mechanism. Quarkonium, hidden heavy flavor bound states, are sensitive to the temperature of the system and the deconfinement mechanism.

In this section we first focus on $D$ and $B$ meson production ($B$ mesons are identified through their decay to $J/\psi$ after they have passed through the medium).

The role of these directly-reconstructed mesons as probes, rather than relying on their semileptonic decays, has come into maturity at the LHC. Single lepton measurements, while useful, do not generally allow a clean flavor separation. Thus we concentrate on $D$ and $B$ measurements at the LHC, with reference to RHIC results where appropriate. We first discuss the measurement of the nuclear modification factor $R_{AA}$ and azimuthal anisotropy $v_2$ of heavy flavor in the bulk medium. We then discuss correlation studies as well as some of the first $p$+Pb results on open heavy flavor. The rest of the section is devoted to a discussion of quarkonium results.

*h. Mass hierarchy of* $R_{AA}$   The nuclear modification factor $R_{AA}$ of heavy-flavored particles has been measured up to rather high $p_{T}$ and can thus provide information about parton energy loss in the medium. Based on QCD predictions of parton energy loss, see Sects. 6.4.2 and 6.4.4, pions, primarily originating from gluons and light quarks, should be more suppressed than charm particles which are, in turn, expected to be more suppressed than particles containing bottom quarks. Thus a hierarchy of suppression is expected with $R_{AA}^B > R_{AA}^D> R_{AA}^{\mathrm{ch}}$.

**Fig. 64:**
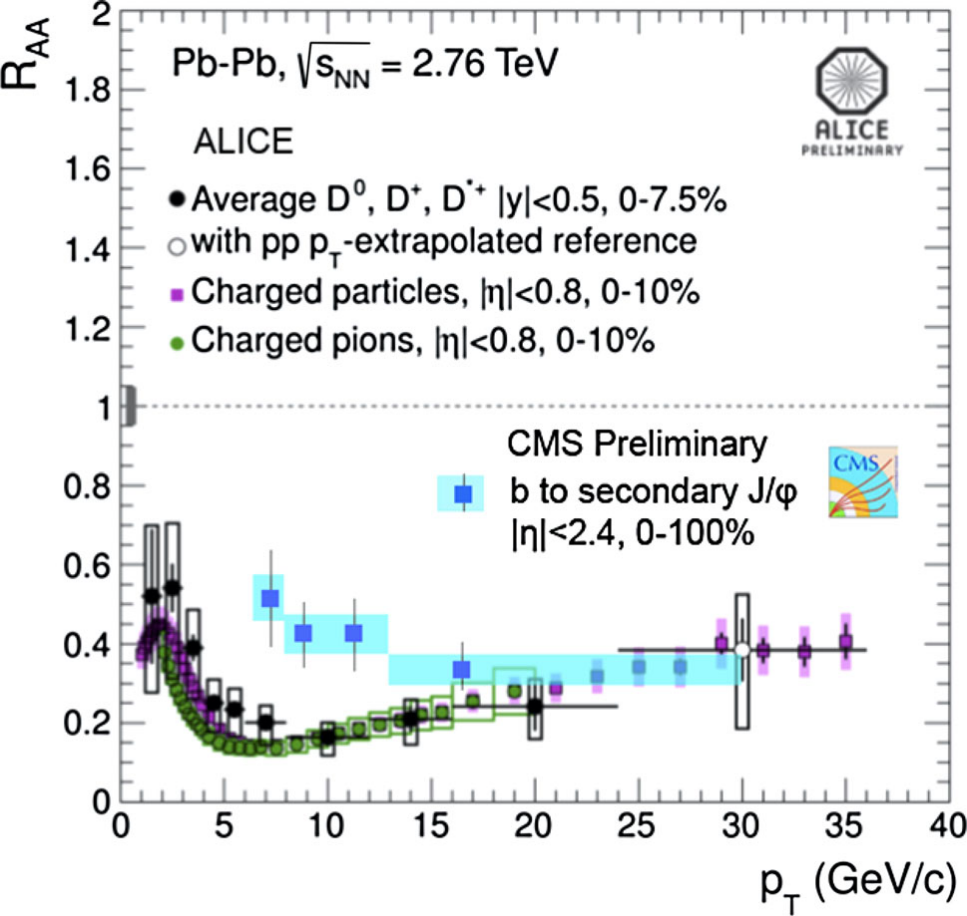
Transverse momentum dependence of the nuclear modification factor $$R_{AA}$$ for prompt $$D$$ mesons measured by ALICE as the average of the relevant factors for $$ D^{0}$$, $$ D^{+} $$, and $$ D^{*+} $$ at midrapidity in central Pb+Pb collisions at $$\sqrt{s_{NN}} = 2.76$$ TeV, compared to the $$R_{AA}$$ of charged hadrons and pions [2144]. The $$b$$-quark energy loss, via nonprompt $$J/\psi $$ from $$B$$-hadron decays by CMS is also shown [2145]. Data from ALICE [2144] and CMS [2145]; plot from [2136]

Figure 64 presents the $$R_{AA}$$ of charged hadrons, charged pions, prompt $$D$$, and prompt $$B$$ decays measured via nonprompt $$J/\psi $$. The $$R_{AA}$$ for prompt $$D$$ mesons is calculated as the average of the relevant contributions from $$ D^{0}$$, $$ D^{+} $$, and $$ D^{*+} $$ [2146] for the 7.5$$~\%$$ most central Pb+Pb collisions at the LHC [2144, 2147]. A suppression factor of 4–5 is observed, corresponding to a minimum $$R_{AA}$$ of $$\approx 0.2$$ at $$p_{T}$$ = 10  GeV/$$c$$. An increase of the $$R_{AA}$$ with $$p_{T}$$ may be expected for a power-law spectrum with an energy loss equivalent to a constant fraction of the parton momentum if the exponent in the power law increases with $$p_{T}$$. To test the predicted hierarchy of suppression, the results are compared to the charged hadron $$R_{AA}$$ and found to be very similar. At $$p_{T}$$ $$<8$$ GeV/$$c$$ the average $$R_{AA}$$ for prompt $$D$$ mesons is slightly higher than the charged particle $$R_{AA}$$ (although still within the systematic uncertainties), showing a weak indication that $$R_{AA}^D> R_{AA}^{\mathrm{ch}}$$. At higher $$p_{T}$$, the $$D$$ meson $$R_{AA}$$ is similar to that of charged hadrons [2144, 2147]. The $$b$$-quark energy loss has been measured by CMS [2145] through $$B$$-hadron decays to non-prompt $$J/\psi $$ showing a steady and smooth increase of the suppression as $$p_{T}$$ increases and remaining always above the $$D$$ mesons. Similar measurements have been published also by ATLAS, in particular open heavy-flavor production via semileptonic decays to muons as a function of centrality [2148]. However, more data are still needed to draw final conclusions about the light hadron and charm meson hierarchy of energy loss.

**Fig. 65:**
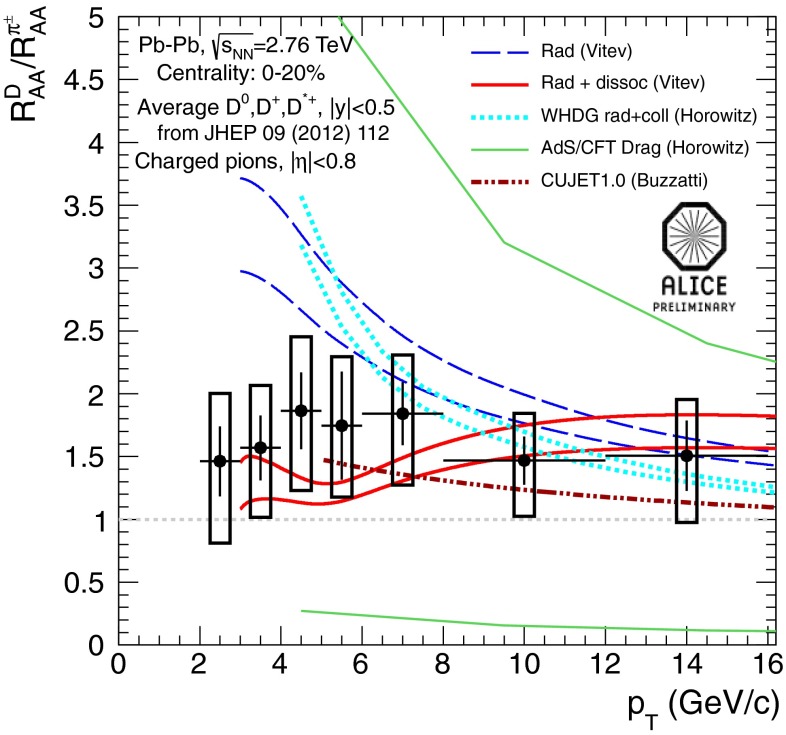
Transverse momentum dependence of the ratio of $R_{AA}$ for $D$ mesons to pions [2144]. The data are compared to the following model predictions: Rad (Vitev) and Rad+dissoc (Vitev) [2149, 2150], WHDG [2151], AdS/CFT Drag [2152], CUJET [2153]. From [2154]

To better quantify the difference between the $R_{AA}$ of $D$ mesons and charged pions, Fig. 65 shows the ratio $R_{AA}^D/R_{AA}^{\pi ^\pm }$. The ratio is larger than unity so that $R_{AA}^D> R_{AA}^{\pi ^\pm }$. The model comparisons, also presented, show that a consistent description of energy loss for light and heavy quarks is a challenge to theory. As seen in Fig. 65, partonic energy loss models achieve a good description at high $p_{T}$ while the low $p_{T}$ region is generally not well described. The similarity between light and charm hadron $R_{AA}$ at high $p_{T}$ is perhaps not so surprising because, in the region where $p_{T} \gg m_Q$, the heavy quark is effectively light as well. However, at low to intermediate values of $p_{T}$, mass effects become important and it becomes more challenging to model these results. In general, more data and quantitative comparison with models are required to understand how the relative small difference between the $R_{AA}$ for light hadrons and heavy flavor can be accommodated by theory in the region where $p_{T} \gg m$ does not hold. This behavior could be due to large elastic energy loss in the strongly coupled quark–gluon plasma [2155, 2156] or the persistence of heavy resonances within the medium [2157]. Recent studies have shown that calculations involving strong coupling [2155, 2156], fixed at RHIC energies, do not extrapolate well to LHC, neither for light nor heavy flavors. Enhanced elastic scattering with resonances in a partly confined medium [2158] seems a promising scenario.

**Fig. 66:**
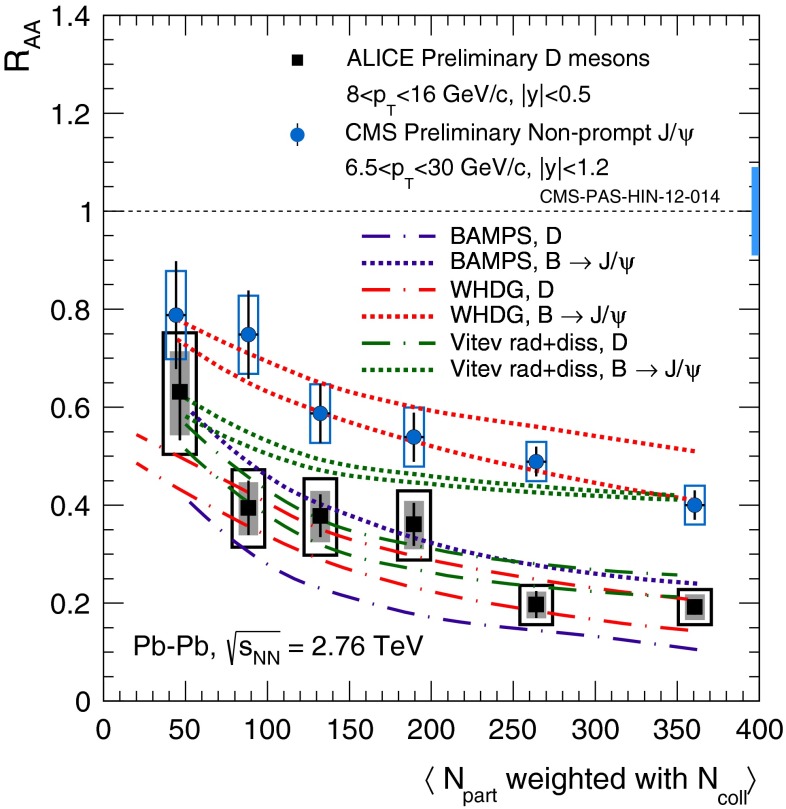
Centrality dependence of the charm and bottom hadron $R_{AA}$ [2159, 2160]. The data are compared to BAMPS [2161], WHDG [2151] and Vitev et al. [2149] model calculations. From [2144]

Further indications of the flavor dependence of $R_{AA}$ are shown in Fig. 66, which presents the centrality dependence of the charm and bottom hadron $R_{AA}$ at intermediate $p_{T}$, where $R_{AA}$ exhibits a shallow minimum.

The ALICE data on prompt charmed hadrons are compared to CMS measurements of $J/\psi$ from $B$-hadron decays to $J/\psi$. These nonprompt $J/\psi$ results were the first to directly show $B$-meson energy loss. A compatible $p_{T}$ range for $D$ ($\langle p_{{T}}^{D}\rangle \approx 10$ GeV/$c$) and $B$-mesons ($\langle p_{{T}}^{B}\rangle \approx 11$ GeV/$c$) has been chosen for more direct comparison. These results provide the first clear indication of the mass dependence of $R_{AA}$.

**Fig. 67:**
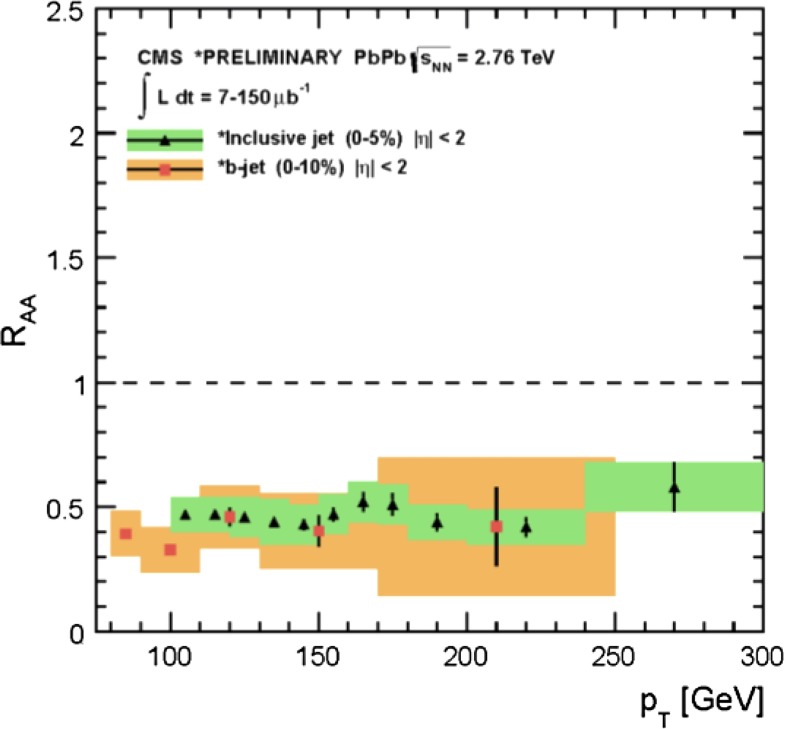
The inclusive [2122] and $b$-jet [2145] $R_{AA}$ as a function of $p_{T}$ in the most central Pb+Pb collisions

Similar to inclusive hadrons, jet modification in high-energy heavy-ion collisions is expected to depend on the flavor of the fragmenting parton. To disentangle this flavor dependence, heavy-quark jets have been studied. CMS measured $b$-quark jet production relative to inclusive jets in $pp$ and Pb+Pb collisions at $\sqrt{s_{NN}} = 2.76$ TeV [2145] (Fig. 67). The measurement is in the range $80 < p_{T} < 200$ GeV/$c$. The measured values are comparable to those predicted by PYTHIA vacuum simulations. The Pb+Pb $b$-jet fraction is also compatible with the $pp$ $b$-jet fraction, within sizeable uncertainties. The measurement is sufficiently precise to demonstrate that $b$-jets are subject to jet quenching, although a precise comparison of light- and heavy-quark jet quenching would require a reduction of the statistical and systematic uncertainties.

These results from $b$-jet studies, together with those from $B$ decays cover a wide $p_{T}$ range and provide a consistent picture. In general the mass effect seems to be as predicted: at intermediate $p_{T}$, bottom is less suppressed than charm, whereas at very high $p_{T}$ ($E/m\gg 1$) $b$-jets and inclusive jets are similarly modified [1744].

The data are compared to several model calculations. Out of the calculations shown, only the WHDG result [2151] is compatible with $R_{AA}$for both $D$ and $B$ hadrons. WHDG also achieve results in agreement with the $D$ to $\pi ^\pm$ $R_{AA}$ shown in Fig. 65 for $p_{T} > 6$ GeV/$c$. The calculations by Vitev *et al.* [2149] Rad (Vitev) and Rad+dissoc (Vitev) [2149, 2150], especially those labeled “Rad+dissoc” in Fig. 65, agree well with $R_{AA}^D/R_{AA}^{\pi ^\pm }$. However, they overpredict the $B$ meson suppression as seen in Fig. 66. The limitation of some calculations to describe the ratio of heavy-to-light $R_{AA}$  shown in Fig. 65 for $p_{T} < 8$ GeV/$c$, may be expected because, in this range, charm mass effects may still play a role.

The measurements of $$D$$ mesons with $$u$$ and $$d$$ quarks have recently been complemented with the first measurement of charm-strange, $$D$$ $$_{s}$$, mesons in Pb+Pb collisions by the ALICE collaboration [2162]. Since the $$D$$ $$_\mathrm{s}$$ contains both charm and strange quarks, neither of which exist in the initial state, these mesons can probe the details of the hadronization mechanism [2163, 2164]. For example, if in-medium hadronization is predominantly responsible for hadronization at low momentum, the relative production of strange to nonstrange charm hadrons should be enhanced. The measurement shows that $$R_{AA}$$ for $$D$$ $$_{s}$$ at $$8 < p_{{T}} < 12$$ GeV/$$c$$ is compatible with that for $$D$$ mesons, with a suppression factor of 4–5 for $$p_{{T}} > 8$$ GeV/$$c$$. In the lower $$p_{T}$$ bin, the $$D$$ $$_{s}$$ $$R_{AA}$$ seems to show an intriguing increase relative to that of $$ D^{0}$$ but the current experimental uncertainties need to be improved before any conclusive comparison can be made.

*i. Heavy-flavor azimuthal anisotropy* Further insight into the properties of the medium can be obtained by investigating the azimuthal anisotropies of heavy-flavor hadrons. If heavy quarks re-interact strongly with the medium, heavy-flavor hadrons should inherit the azimuthal anisotropy of the medium, similar to light hadrons. Measurements of the second Fourier coefficient $v_2$ at low $p_{T}$ can provide information on the degree of thermalization, while at high $p_{T}$ can give insight into the energy loss mechanism.

**Fig. 68:**
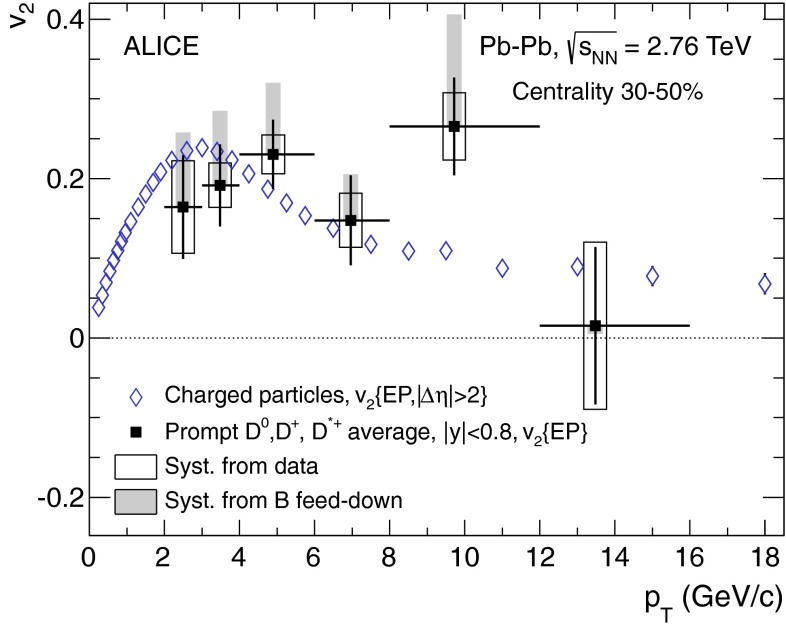
The transverse momentum dependence of $v_2$ for $D$ mesons in the 30–50 % centrality bin relative to that of inclusive charged hadrons. From [2165]

**Fig. 69:**
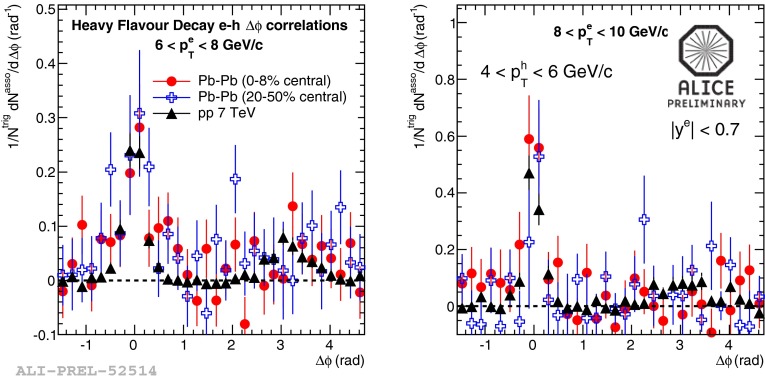
Azimuthal angular correlations $\Delta \phi (\mathrm{HFE},h)$ for $4<p_{T}^{h}<6$ GeV$/c$ in 0–8 % (*red*) and $20\hbox {--}50~\%$ (*blue*) central Pb–Pb collisions at $\sqrt{s_{NN}} = 2.76$ TeV compared to $pp$ collisions at $\sqrt{s} = 7$ TeV (*black*). From [2167]

Recent measurements of the prompt $D$ meson $v_2$ as a function of $p_{T}$ in the 30–50 % centrality bin are shown in Fig. 68. A finite $v_2$ value with a significance of 3$\sigma$ is observed for $2 < p_{T} < 8$ GeV/$c$, compatible with that of light hadrons within the uncertainties, showing that $D$ mesons interact strongly with the medium. However, higher statistics measurements covering lower $p_{T}$ are needed to draw firm conclusions about charm quark thermalization in the hot medium created at the LHC.

Further differential measurements include the study of the $$ D^{0}$$ $$R_{AA}$$ in the in- and out-of-plane azimuthal regions [2166]. The results indicate larger suppression in the out-of-plane azimuthal region, as expected, due to the longer path length traversed through the medium in this case.

Current model comparisons which include both radiative and elastic (collisional) energy loss can explain the high-$p_{T}$ $R_{AA}$ data in the region where $p_{T} \gg m_Q$. However, energy loss alone is insufficient for describing the low-$p_{T}$ $R_{AA}$ and $v_2$ results. Models which incorporate recombination or in-medium resonance formation can better describe this region where mass effects could be important.

*j. Flavor correlations* To a great extent, correlations between heavy quarks survive the fragmentation process in proton–proton interactions. On the other hand, in heavy-ion collisions, the medium alters the fragmentation process so that observables are sensitive to the properties of the medium. It has been shown that the fragmentation function, which describes how the parton momentum is distributed among the final-state hadrons, is most suited for these detailed studies. Flavor conservation implies that heavy quarks are always produced in pairs. Momentum conservation requires that these pairs are correlated in relative azimuth ($\Delta \phi$) in the plane perpendicular to the colliding beams. Since heavy flavors are produced in $2 \rightarrow 2$ ($gg \rightarrow Q \overline{Q}$) and $2 \rightarrow 3$ (e.g., $gg \rightarrow Q \overline{Q} g$) processes, the azimuthal correlation is not strictly back-to-back.

One method of exploiting this pair production characteristic is to measure the correlation of electrons from semileptonic decays of heavy-flavor hadrons (HFE) with charged hadrons. Figure 69 shows the $\Delta \phi (\mathrm{HFE, hadron})$ distribution measured by the ALICE Collaboration [2168]. A distinct near-side correlation is observed.

**Fig. 70:**
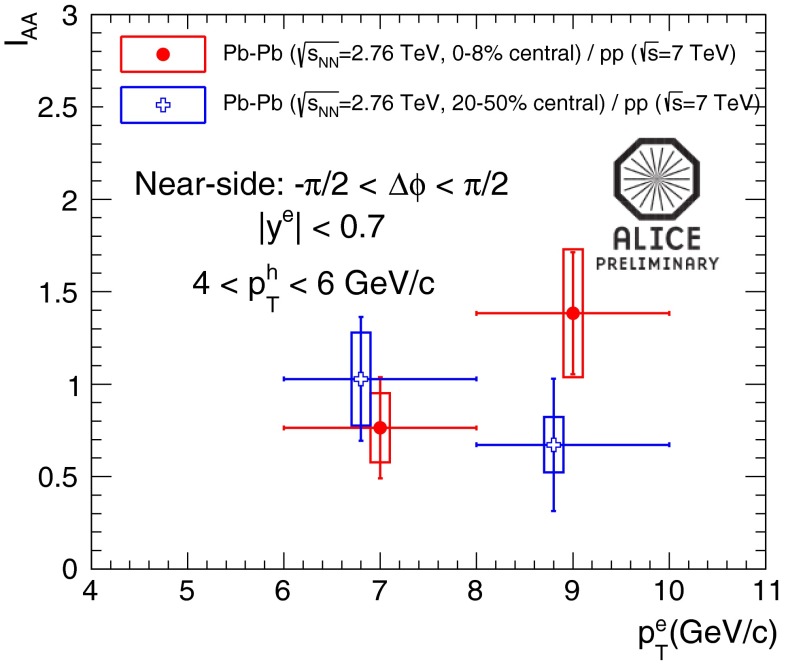
The $I_{AA}$ of the near-side $\Delta \phi (\mathrm{HFE, hadron})$ correlation in the 0–8 % and 20–50 % most central Pb+Pb collisions [2168]

The ratio of the measured Pb+Pb correlation relative to the $pp$ correlation, $I_{AA}$,

6.4 $$\begin{aligned} I_{AA} = \frac{\int _{\phi _1}^{\phi _2} d \Delta \phi (dN_{AA}/d \Delta \phi )}{\int _{\phi _1}^{\phi _2} d \Delta \phi (dN_{pp}/d \Delta \phi )}\!, \end{aligned}$$

on the near side ($-\pi /2 < \Delta \phi < \pi /2$) as a function of the electron trigger $p_{T}$ is shown in Fig. 70. An excess, $I_{AA} > 1$, may be expected at high electron $p_{T}$ in central collisions due to the depletion and broadening of the correlation signal in the medium. These results agree with previous measurements at RHIC [2169]. However, so far they are statistics limited and more precision data are needed, both at RHIC and the LHC, to draw final conclusions. Simulation studies suggest that the 5.5 TeV Pb+Pb data, expected after 2015, should be sufficient for these studies.

*k. Heavy flavor in* $p$+Pb *collisions* To quantitatively understand $AA$ results in terms of energy loss, it is important to disentangle hot nuclear matter effects from initial-state effects due to cold nuclear matter, such as the modification of the parton distribution functions in the nucleus [139], discussed in Sect. 6.4.2, and saturation effects in the heavy-flavor sector [2170]. Initial-state effects can be investigated by measuring $D$ production in $p$+Pb collisions.

**Fig. 71:**
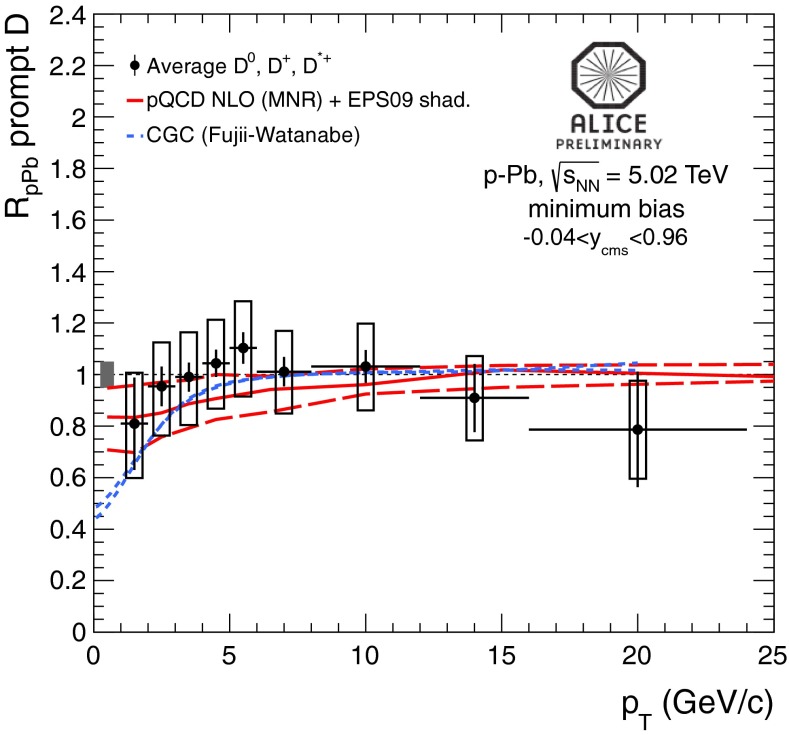
Average $$ D^{0}$$, $$ D^{+} $$, and $$ D^{*} $$ $$R_{p\mathrm{Pb}}$$ [2173] compared with NLO pQCD [139, 2171] and CGC calculations [2174]. From [2173]

The nuclear modification factor of the averaged prompt $$ D^{0}$$, $$ D^{+} $$ and $$ D^{*} $$ mesons in minimum bias $$p$$+Pb collisions at $$\sqrt{s_{NN}} = 5.02$$ TeV is compatible with unity within systematic uncertainties over the full $$p_{T}$$ range, see Fig. 71. The data are compared with pQCD calculations based on the exclusive NLO heavy-flavor calculation [2171] employing the EPS09 modifications of the parton distribution functions [139] and also with a color glass condensate-based calculation [2172]. Both models describe the data within the uncertainties indicating that the strong suppression observed in central Pb+Pb interactions is a final-state effect.

*l. Quarkonium results* We now turn to recent results on quarkonium, bound states of “hidden” charm ($J/\psi$ and $\psi '$) and bottom ($\Upsilon (1S)$, $\Upsilon (2S)$ and $\Upsilon (3S)$). As discussed in Sect. 6.4.3 the dissociation of the quarkonium states due to color screening in the QGP is one of the classic signatures of deconfinement [1968]. The sequential suppression of the quarkonium states results from their different typical radii providing a so-called “QCD thermometer” [2175]. In this scenario excited states such as the $\Upsilon (2S)$, are more suppressed than the $J/\psi$ while the $\Upsilon (1S)$, the most tightly bound quarkonium $S$ state, is the least suppressed, as shown by the CMS Collaboration [2176].

The nuclear modification factor $R_{AA}$ has been measured at mid- and forward rapidity in Pb+Pb collisions at $\sqrt{s_{NN}} = 2.76$ TeV. The $R_{AA}$ can be quantified either in terms of collision centrality, generally presented as a function of the number of nucleon participants, $N_{\mathrm{part}}$, or as a function of the quarkonium $p_{T}$ in a given centrality bin. While we present the quarkonium $R_{AA}$ here, we note that a comparison to $pp$ may not be the most relevant baseline for quarkonium. Instead, quarkonium suppression should be normalized relative to open heavy flavor results in the same acceptance [2177] because a similar suppression pattern for $J/\psi$ and $D$ mesons may not be indicative of Debye screening but of another mechanism, such as parton energy loss, particularly at high $p_{T}$.

**Fig. 72:**
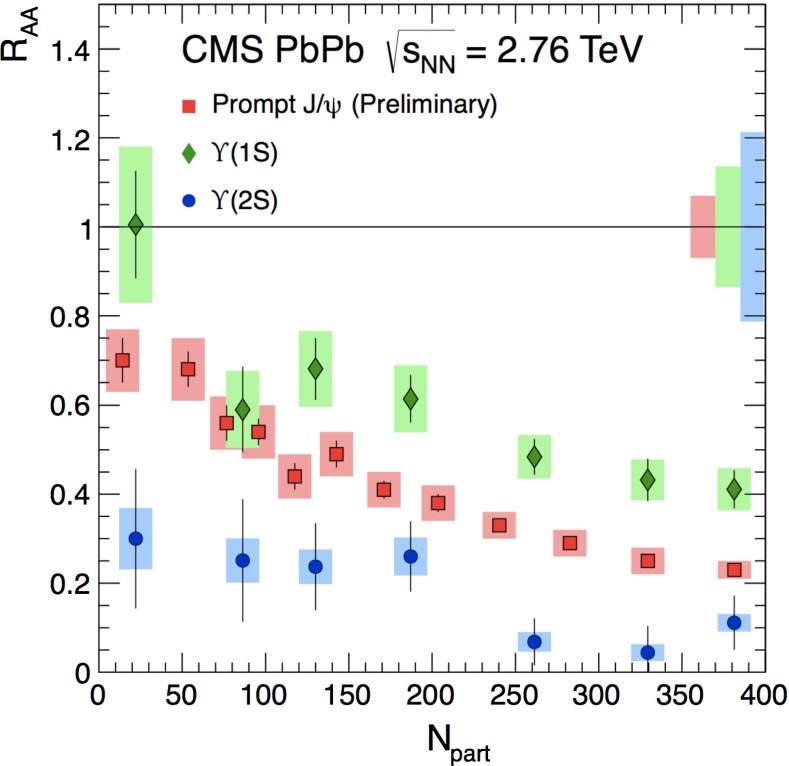
The nuclear modification factor $R_{AA}$ for prompt $J/\psi$, $\Upsilon (1S)$ and $\Upsilon (2S)$ at midrapidity as a function of the number of participants in Pb+Pb collisions at $\sqrt{s_{NN}} = 2.76$ TeV measured in the dimuon channel by the CMS Collaboration. From [2178]

The CMS $\Upsilon$ and $J/\psi$ results, shown as a function of $N_{\mathrm{part}}$ in Fig. 72, indicate that the sequential melting scenario appears to hold. The $\Upsilon (1S)$ is least suppressed while the $\Upsilon (2S)$ is almost completely suppressed in the most central collisions. The prompt $J/\psi$ result, with the $J/\psi$s from $B$ decays removed, is intermediate to the two. Note, however, that the prompt $J/\psi$ measurement is at higher $p_{T}$, $p_{T} > 6$ GeV/$c$, than those of the $\Upsilon$ states, available for $p_{T} > 0$.

The sequential suppression pattern described above may be affected by regeneration of the quarkonium states due to the large $Q \overline{Q}$ multiplicity at LHC energies, either in the QGP or at chemical freeze-out [2180–2184]. Such regeneration might lead to enhancement of the quarkonium yields in some regions of phase space, as we now discuss.

**Fig. 73:**
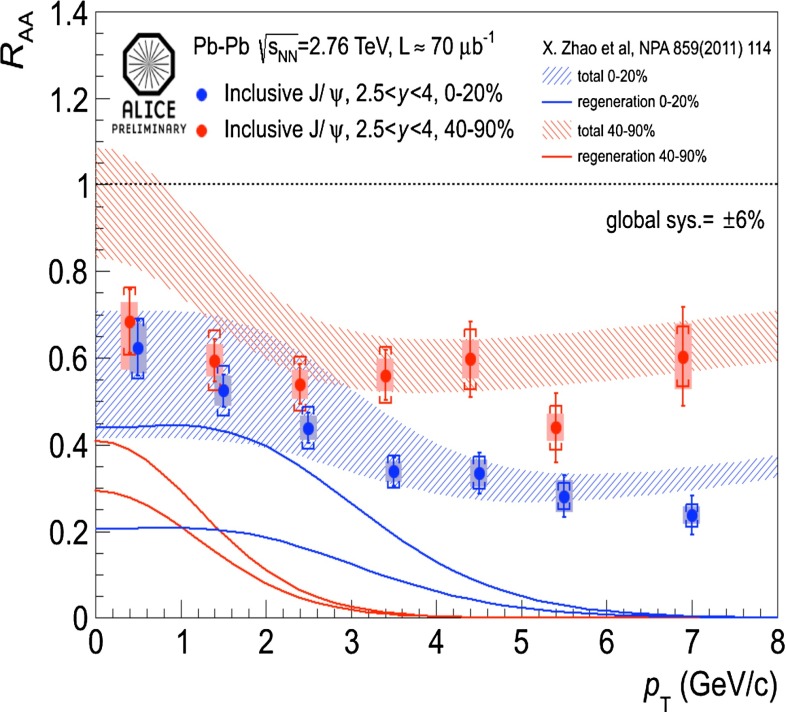
Inclusive $J/\psi$ $R_{AA}$ in the dimuon channel at forward rapidity in two different centrality bins measured by ALICE [2179]. The *curves* show transport model calculations [2180]

The ALICE Collaboration has measured $J/\psi$ suppression at midrapidity with electrons and at forward rapidity in the dimuon channel. The suppression has been studied as a function of centrality and $p_{T}$. The results indicate that inclusive $J/\psi$ production is less suppressed at low $p_{T}$, even at forward rapidity, see Fig. 73, which was not observed at the lower RHIC energy. In general, the ALICE measurements show that for collisions with $N_{\mathrm{part}} > 100$ (the 40–90 % centrality bin), $R_{AA}$ is almost constant as a function of $p_{T}$ while the overall suppression is less than that observed in the most central RHIC collisions. In the 20 % most central collisions, $R_{AA}$ decreases with $p_{T}$, as also shown in Fig. 73, similar to the RHIC measurements [2185–2188]. A smaller suppression is observed at $p_{T} < 2$ GeV/$c$ than at higher $p_{T}$ ($5 < p_{{T}} < 8$ GeV/$c$), especially in more central collisions, as also seen in Fig. 73 [2189]. The ALICE results also suggest that the midrapidity measurements (not shown) exhibit less suppression in central collisions than those at forward rapidity [2189].

The results shown in Fig. 73 are qualitatively in agreement with quarkonium regeneration, where the effects are expected to be important in central collisions, particularly at low $p_{T}$ and midrapidity. Remarkably, these results suggest that regeneration may still be important at forward rapidity. While this needs to be thoroughly checked before firm conclusions are drawn, comparison with transport and statistical model calculations suggest that a sizable regeneration component is needed to describe the low-$p_{T}$ data. Further details on the measurements and model comparisons can be found in Ref. [2154].

**Fig. 74:**
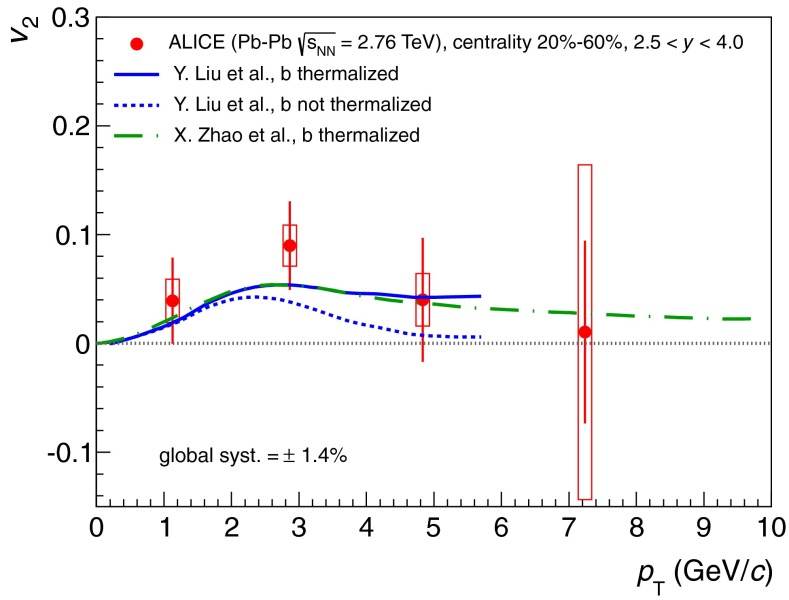
Second Fourier coefficient $v_2$ for $J/\psi$ in the 20–60 % centrality range as a function of $p_{T}$. The ALICE data [2190] are compared with transport model predictions [2191, 2192]. From [2190]

*m.* $J/\psi$ *azimuthal anisotropy* The ALICE Collaboration has studied the $J/\psi$ azimuthal anisotropy at forward rapidity. The results are shown in Fig. 74 for the 20–60$~\%$ centrality bin. This first measurement of inclusive $J/\psi$ $v_2$ at the LHC shows a hint of a nonzero value in a somewhat narrower $p_{T}$ range than that of the $D$ mesons. This measurement suggests that the $J/\psi$ may also follow the collective behavior of the bulk QGP at low $p_{T}$. These results are in agreement with expectations from kinetic and statistical hadronization models which require thermalization of the charm quarks in the QGP. The calculations differ as to whether or not the $b$ quarks responsible for nonprompt $J/\psi$ production thermalize in the medium. For more details, see Ref. [2190].

**Fig. 75:**
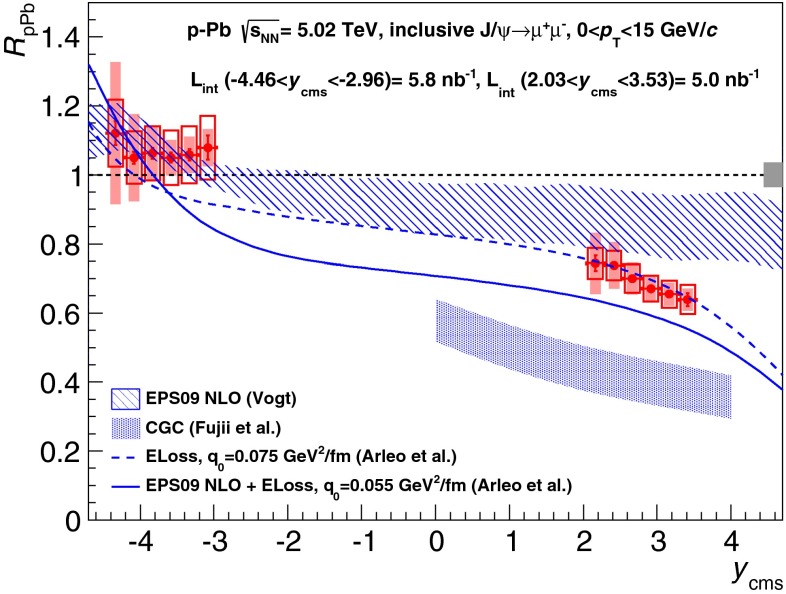
The nuclear modification factors for inclusive $J/\psi$ production at $\sqrt{s_{NN}} = 5.02$ TeV measured by the ALICE Collaboration [2197]. Calculations from several models [1947, 2001, 2198] are also shown. From [2199]

*n.* $J/\psi$ $R_{AA}$ *in* $p$+Pb *collisions* We now discuss the $J/\psi$ results in $p$+Pb collisions at the LHC. The rapidity dependence of the nuclear modification factor $R_{p\mathrm{Pb}}$ measured by ALICE is shown in Fig. 75 [2193]. The LHCb result [2194], in a narrower rapidity window, agrees well with the ALICE measurement. While there is a suppression relative to $pp$ at forward rapidity, no suppression is observed in the backward region. There is good agreement with predictions based on nuclear shadowing with the EPS09 parameterization alone [1947, 2195], as well as with models including a contribution from coherent partonic energy loss [2196]. Whether shadowing only or shadowing with energy loss is the correct description requires more data and smaller uncertainties. The largest experimental uncertainty is due to the $pp$ interpolation. The CGC prediction clearly overestimates the suppression. These results suggest that no significant final-state absorption effects on the $J/\psi$ are required to explain the data, providing an important baseline for the interpretation of heavy-ion collision results.

### Reference for heavy-ion collisions

One of the most powerful tools in heavy-ion physics is the comparison of $AA$ data with $pp$ or $pA$ reference data in order to disentangle initial- from final-state effects. The $R_{p\mathrm{Pb}}$ measurements shown in Sect. 6.4.4 for charged hadrons and heavy flavor are typical examples of this approach. It is, however, based on the assumption that final-state effects are absent in the elementary collision systems. In the LHC and RHIC energy regime, this assumption is non-trivial due to the relatively large number of produced particles and is currently under experimental investigation.

Similar to the measurement in Pb+Pb collisions, the $p_{T}$-integrated charged particle density distribution measured as a function of $\eta$ in $p$+Pb provides essential constraints [1805]: models that include shadowing [2200] or saturation [1946, 2201] predict the total measured multiplicity to within 20 % (see also Figs. 47 and 52). A closer look at the $\eta$-dependence reveals that saturation models tend to overpredict the difference in multiplicity in the Pb direction relative to the multiplicity in the proton direction, see Sect. 6.3.2. Other models, such as [1934] which consider the effects of strong longitudinal color fields and predict too much suppression when shadowing is included and too little when it is not. By tuning the gluon shadowing in d$+$Au collisions at RHIC, DPMJET [2202] and HIJING 2.1 [2200], obtain multiplicities that are close to the data. Recent ATLAS preliminary results [2203] on the centrality dependence of the charged particle multiplicity production in $p$+Pb can provide further constraints on model predictions. In particular, the data seem to be correctly described by the prediction of Ref. [2204].

**Fig. 76:**
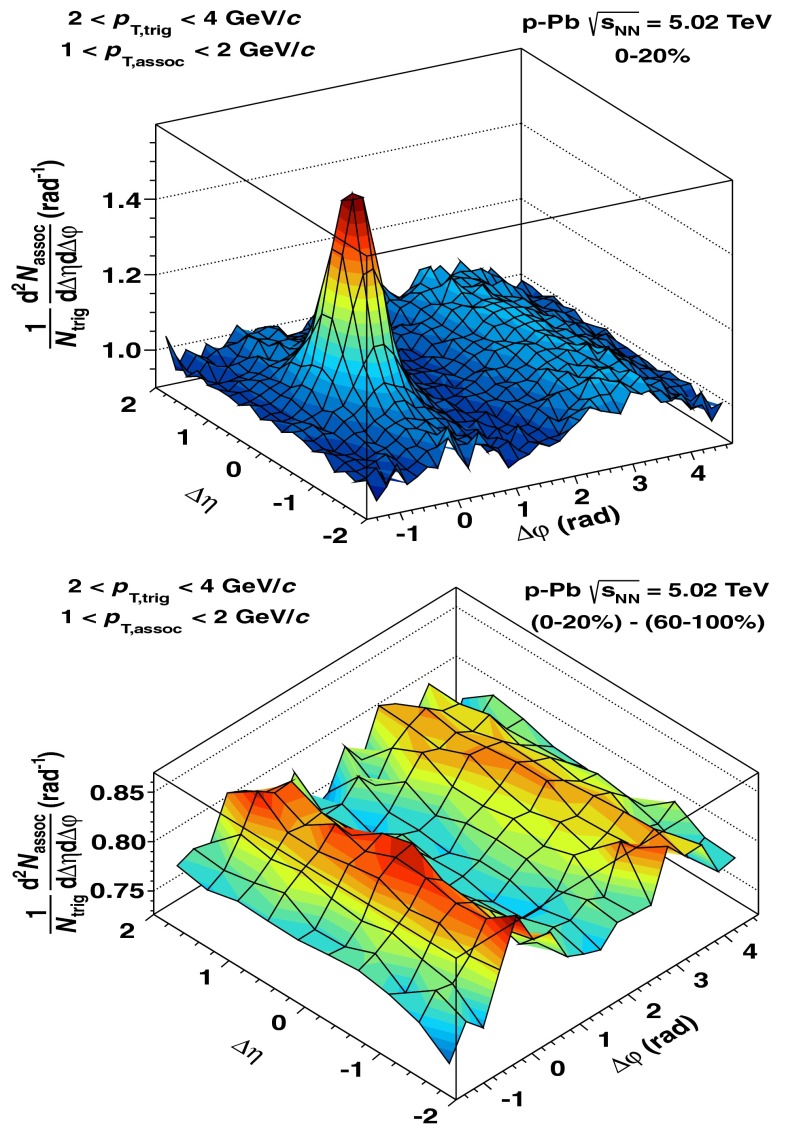
*Top* The associated yield per trigger particle in $\Delta \varphi$ and $\Delta \eta$ for pairs of charged particles with $2<$ $p_{T}$ $<$ 4 GeV/$c$ for the trigger particle and $1<$ $p_{T}$ $<$ 2 GeV/$c$ for the associated particle in $p$+Pb collisions at 5.02 TeV for the 0–20 % event multiplicity class. *Bottom* The same quantity after subtraction of the associated yield obtained in the 60–100 % event class. From [2205]

In addition to the studies of the minimum bias data samples, typical observables used to characterize heavy-ion collisions can be studied as a function of multiplicity in $pp$ and $p$+Pb collisions. In particular, at the high LHC energies the particle multiplicity in the high-multiplicity classes of elementary collisions are comparable to e.g., Cu+Cu collisions at RHIC energies.

Some of the most surprising results in elementary collision systems at the LHC have been obtained by measuring two-particle correlations in high-multiplicity events. In particular, $\Delta \eta$–$\Delta \phi$ distributions exhibit several structures that arise from different physics mechanisms; here, $\eta$ and $\phi$ denote pseudorapidity and azimuthal angle, while $\Delta$ denotes the difference between the trigger particle and the associated particle. In $pp$ [2206] as well as $p$+Pb collisions [2207–2210], a novel ridge-like correlation structure, elongated in rapidity, has been observed for particles emitted within an azimuthal angle close to that of the trigger particle. This region in phase space with $\Delta \phi \approx 0$ is often referred to as the “near side”. In $p$+Pb collisions, effects originating from the interplay of multiple $NN$ collisions are separated from those arising from a superposition of individual $NN$ collisions by subtracting the distributions of low-multiplicity events, with $N_\mathrm{part} \sim 2$, from the ones of high-multiplicity. As shown in Fig. 76, this procedure removes the jet peak close to $\Delta \eta \approx 0$ on the near-side and reveals the presence of the same ridge structure on the “away side” ($\Delta \phi \approx \pi$) with similar magnitude [2208]. In heavy-ion reactions, the double-ridge structure has been interpreted as originating from collective phenomena such as elliptic flow. Several theoretical explanations of these observations have been put forward, including those based on saturation models [1746] and hydrodynamic flow [2211]. However, the application of hydrodynamic models to small systems such as $p$+Pb is complicated because uncertainties related to initial-state geometrical fluctuations and viscous corrections may be too large for hydrodynamics to be a reliable framework [164].

To clarify the situation, the mass dependence of the ridge effect has been investigated [2208]. Indeed, an ordering of pions, kaons, and protons was found, which is reminiscent of similar observables in Pb+Pb collisions (see also Fig. 50). This behavior was successfully predicted by the EPOS event generator [2212]. The model is founded on the parton-based Gribov-Regge theory, in which the initial hard and soft scatterings create flux tubes that either escape the medium and hadronize as jets or contribute to the bulk matter, described in terms of hydrodynamics.

Significant insights into the origin of the azimuthal correlations in small collision systems has been provided by CMS by studying two- and four-particle azimuthal correlations, particularly in the context of hydrodynamic and color glass condensate models. A direct comparison of the correlation data between $p$+Pb and Pb+Pb collisions has been measured as a function of particle multiplicity and transverse momentum. The observed correlations were quantified in terms of the integrated near-side associated yields and azimuthal anisotropy Fourier harmonics ($v_n$). Multiparticle correlations were also directly investigated over a wide range of pseudorapidity as well as in full azimuth [2207].

Exploiting the excellent particle identification capabilities at low momentum, ALICE is measuring untriggered $\Delta \eta$–$\Delta \phi$ correlations of pions, kaons and protons. Qualitatively new features, relative to correlations of unidentified correlations, are observed for kaons and protons. In particular the influence of the local conservation of strangeness and baryon quantum numbers was found to be large [2213, 2214]. The effects are not well reproduced by Monte-Carlo models [2213–2215]. This measurement can shed new light on the process of fragmentation and hadronization in elementary collisions. Such studies were initiated in the 1970s by several authors, including Richard Feynman [2216].

A careful analysis of the mean transverse momenta of charged particles [2217] and of the spectral shapes of $\pi$, $K$, $p$ and $\Lambda$ production [2218, 2219] as a function of event multiplicity have been pursued in order to investigate the presence of radial flow. In both cases, hydrodynamics-based models, like EPOS, yield a reasonable description of the data. The same holds true for the blast-wave picture [1832], in which the simultaneous description of the identified particle spectra shows similar trends as in Pb+Pb collisions. The observed baryon-to-meson ratios show an enhancement at intermediate transverse momenta which is even more pronounced in high multiplicity collisions. This behavior is phenomenologically reminiscent of the evolution of the same observable with centrality in Pb+Pb collisions (see also Fig. 49).

At the same time, detailed comparisons with PYTHIA8 [2220] show that other final-state mechanisms, such as color reconnection [2221], can mimic the effect of collectivity. In particular, the evolution of $p_{T}$ distributions in PYTHIA8 from those generated in $pp$ collisions follows a trend similar to the blast-wave picture for $p$+Pb or Pb+Pb collisions, even though no hydrodynamic component is present in the model. It will therefore be challenging to differentiate between these two scenarios. Systematic comparisons of identical observable in $pp$, $p$+Pb  and Pb+Pb collisions will help clarify the situation.

However, in order to be able to perform quantitative comparisons between the different collision systems, the centrality determination in $p$+Pb collisions needs to be carefully addressed. In general, centrality classes are defined as percentiles of the multiplicity distributions observed in different sub-detectors covering disjunct pseudorapidity ranges. In contrast to $AA$ collisions, the correlation between the centrality estimator and the number of binary collisions $N_\mathrm{coll}$ is not very pronounced: the same value of $N_\mathrm{coll}$ contributes to several adjacent centrality classes. In particular at the LHC, several technical, conceptually different methods are being developed and investigated in order to reduce the influence of these fluctuations and to provide a reliable estimate of $N_\mathrm{coll}$ [1950, 2203]. For the time being, systematic comparisons between experiments and collision systems can rely on multiplicity classes similar to $pp$ collisions.

Based on latest results, as for example the ones presented at [1746], it is clear that $p$+Pb collisions can serve not only as a reference to the more complex $AA$ systems, but also provide new insights on these issues and, more generally, on QCD itself.

### Lattice QCD, AdS/CFT and perturbative QCD

One of the major questions in quark–gluon plasma physics is whether a weak-coupling based description works at temperatures of a few hundred MeV, relevant for heavy-ion collisions, or whether the system should be described using strong-coupling techniques. In the strong-coupling limit, the gauge/gravity correspondence provides a computational scheme radically different from traditional field theory tools, applicable to large-$N_\mathrm{c}$ $\mathcal{N}=4$ SYM theory and various deformations thereof. Numerous calculations have demonstrated that non-Abelian plasmas behave very differently at weak and strong coupling. In particular, while the weakly-coupled system can be described by a quasi-particle picture, at strong coupling the poles of retarded Green’s functions give rise to quasi-normal-mode spectra where the widths of the excitations grow linearly with energy. The different couplings lead to strikingly different predictions for many collective properties of the plasma. Perhaps the best known example is the extremely low shear viscosity of the system [1864].

While the weakly and strongly coupled pictures of a non-Abelian plasma are clearly contradictory, it is a priori uncertain which observables and physical processes in a real QGP fall into which realm. One clean way to address this question for equilibrium quantities is to compare advanced perturbative and gauge/gravity predictions to non-perturbative lattice QCD simulations of the same quantities. In this section, we compare several quantities (often from pure Yang–Mills theory), for which lattice, perturbative and holographic predictions exist. On the holographic side, we begin our discussion from the ${\mathcal N}=4$ SYM theory, moving later to bottom-up gravity duals for non-supersymmetric large-$N_\mathrm{c}$ Yang–Mills theory.

**Fig. 77:**
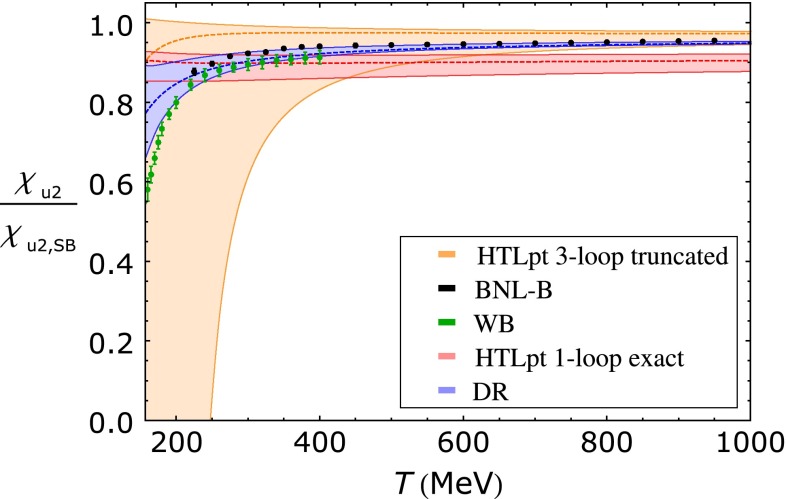
The second-order light quark number susceptibility evaluated in two different schemes of resummed perturbation theory (“dimensional reduction-inspired resummation” and HTLpt compared with recent lattice results from the BNL-Bielefeld (BNL-B) and Wuppertal-Budapest (WB) collaborations. From  [2224]

#### Weakly and strongly coupled (Super) Yang–Mills theories

We begin with the equation of state. The current best-controlled lattice-QCD calculation in the high-temperature regime is found in Ref. [1754]. By $T\approx 2-4 T_\mathrm{c}$, the pressure ($p$), entropy density ($s$) and energy density ($e$) are all in agreement with perturbative predictions. The trace anomaly $e-3p$ has on the other hand traditionally been a more problematic quantity, with perturbative calculations missing the famous peak structure at low temperatures, but a recent calculation employing Hard-Thermal Loop perturbation theory (HTLpt) finds agreement for it already at around $T\approx 2T_\mathrm{c}$ [2222]. On the holographic side, the pressure of an $\mathcal{N}=4$ SYM plasma at infinitely strong coupling is known to be equal to 3/4 the value in the noninteracting limit, similar to that of the equation of state at $T\approx 2 T_\mathrm{c}$ found in lattice QCD. It is therefore not surprising that in bottom-up holographic models of Yang–Mills theory, good quantitative agreement is found for nearly all thermodynamic observables close to the transition temperature, see Sect. 6.6.2.

To probe the region of nonzero quark density, technically very demanding for lattice QCD, one typically studies quark number susceptibilities, i.e., derivatives of the pressure with respect to the quark chemical potentials, evaluated at $\mu _q=0$. Continuum-extrapolated lattice data are currently available only up to $T\approx 400$ MeV [1749, 1750], but even below this temperature impressive agreement with resummed perturbation theory has been observed, see Refs. [2223–2225] as well as Fig. 77. This can be understood from the fermionic nature of the observable, and similar conclusions have indeed been drawn for the full density-dependent part of the pressure [2226, 2227]. Very few gauge/gravity results exist for these quantities due to the supersymmetry of the SYM theory; one exception is, however, the study of off-diagonal susceptibilities found in Ref. [2228].

Spatial correlation functions, which reflect the finite correlation lengths of the non-Abelian plasma, are another set of interesting observables. Although both perturbative [2229] and holographic [2230] predictions for these quantities exist, a systematic precision lattice-QCD study of this screening spectrum is still missing, even in pure Yang–Mills theory. At distances much shorter than $1/T$, the correlations of local operators effectively reduce to the corresponding vacuum correlators. This contribution can, however, be subtracted non-perturbatively [2231], allowing a prediction of the short distance behavior of the correlation function in the operator product expansion. Such a lattice calculation was carried out [2232] for the components of the energy-momentum tensor. Strikingly, the morphology of the vacuum-subtracted correlator of the scalar operator, $G^a_{\mu \nu }G^{\mu \nu a}$, was found to be closer to strongly coupled $\mathcal{N}=4$ SYM theory than to weakly coupled Yang–Mills theory. This prompted a higher-order calculation of the relevant Wilson coefficients in Yang–Mills theory [2233] that, while displaying less than optimal convergence properties, drove the analytic prediction towards the lattice data. It was also pointed out that considering static ($\omega =0$) rather than equal-time correlators is technically more favorable for perturbative computations [2234], suggesting that new lattice calculations should be performed to aid the comparison.

A closely related quantity, also directly accessible by lattice methods, is the Euclidean imaginary-time correlation function. These correlators play an important role in constraining the corresponding spectral functions, needed to calculate transport coefficients, but can also be subjected to a much more straightforward (and less model-dependent) test: direct comparison with the corresponding perturbative predictions [1892, 1907, 1908]. Extensive continuum-extrapolated calculations are needed to make precise comparisons, achieved in only a few cases so far. For example, when the continuum limit of the isovector-vector channel is taken in the quenched approximation [1896], an 8–9 % deviation from the massless tree-level prediction was found at $\tau =1/(2T)$. These calculations are, however, quite time intensive; some estimated computational times as a function of lattice spacing can be found in Ref. [1889].

In weak coupling, there is typically no major difference in the complexity of determining finite-temperature Green’s functions in the Minkowski-space and Euclidean formulations. Indeed, the thermal spectral function is a particularly versatile quantity since it allows direct determination of a number of other correlators. At the moment, results have been determined up to NLO in several channels. Some relevant operators include the electromagnetic current generated by massless [2235–2237] and massive quarks [2238]; the color electric field [2239]; the scalar and pseudoscalar densities [1908]; and the shear component of the energy-momentum tensor [1907]. The last two results have so far been obtained only for pure Yang–Mills theory. In Ref. [1908] the calculation of NLO spectral functions in the bulk channel was significantly refined and systematized. In particular, it was shown how these quantities can be reduced to sums of analytically calculable vacuum components and rapidly converging finite $T$ pieces. Very recently, this work was further generalized to account for nonzero external three-momenta [2240], extending the applicability of the results to particle production rates in various cosmological scenarios, see Sect. 6.7.

In the absence of lattice data on the Minkowski-space spectral functions, the perturbative results can be tested in three different ways: deriving imaginary time Green’s functions and comparing them to lattice results, as discussed above; verifying and refining non-perturbative sum rules [1906, 2241]; and direct comparison to gauge/gravity calculations. The latter path was taken in Ref. [2242], where the bulk and shear spectral functions of bottom-up Improved Holographic QCD (IHQCD), described later, were seen to accurately reproduce the short-distance (UV) behavior of the NLO perturbative Yang–Mills results [1907, 1908]. The imaginary time correlators obtained from the holographic spectral functions were also seen to be in rather good accord with current lattice data.

Finally, we note that meson spectral functions can be calculated rather straightforwardly holographically, even at finite density. Gauge/gravity duality predicts that meson bound states survive above the deconfinement temperature and that their decay is related to a first-order transition within the deconfined phase [2243–2245]. For ground state mesons, the new transition temperature is proportional to the meson mass so that heavy quarkonia survives at higher temperatures. These results are interesting to compare to those of other approaches, see Ref. [2177] and references therein.

#### Holographic breaking of scale invariance and IHQCD 

While ${\mathcal N}=4$ SYM theory provides an interesting toy model for strong interactions, to approach QCD, breaking of scale invariance must be incorporated into the dual-gravity description. There are two classes of successful string-inspired models that, beyond modifying the metric, also introduce a dynamical dilaton field $\phi$, dual to the Yang–Mills scalar operator Tr$[F^2]$ [1888, 1963, 1964, 2246]. They both can be expressed as a five-dimensional action,

6.5 $$\begin{aligned} S=M_p^2 N_\mathrm{c}^2\int \mathrm{d}^5 x\sqrt{g}\left[ R-{4\over 3}(\partial \phi )^2+V(\phi )\right] , \end{aligned}$$

where the potential $V(\phi )$ is responsible for the running of the ’t Hooft coupling, dual to $\lambda = e^{\phi }$. IHQCD [1888, 1963, 1964] is constructed so that the theory is dual to pure Yang–Mills theory at both zero and finite temperature while the formulation of Ref. [2246] only reproduces the gluon dynamics at finite temperature while it is gapless at $T=0$. In the remainder of this section, we focus on IHQCD and its salient features.

**Fig. 78:**
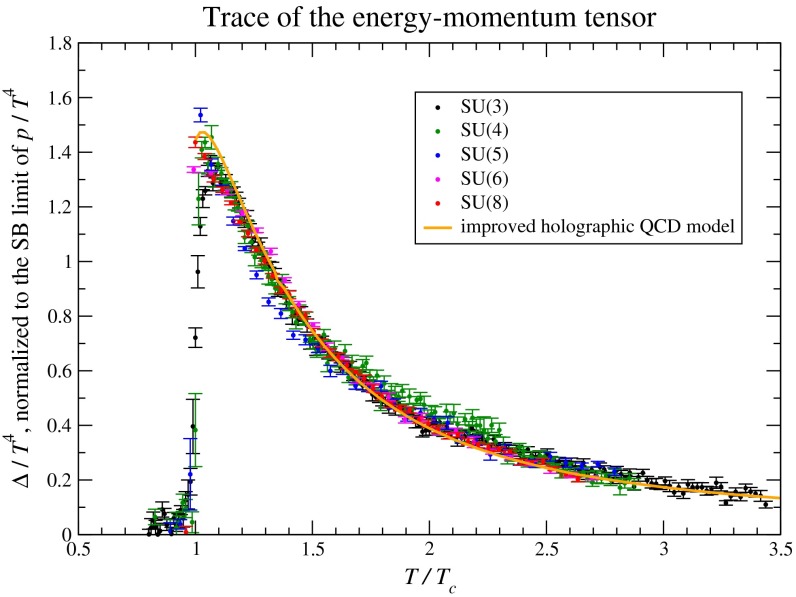
The conformal trace anomaly, $e-3p$, of SU($N_\mathrm{c}$) Yang–Mills theory, normalized by $N_\mathrm{c}^2T^4$. The points with uncertainties are from lattice calculations [2248], while the *yellow line* corresponds to the IHQCD prediction [2247]. From [2248]

For a holographic model to properly account for the UV asymptotic behavior of SU($N_\mathrm{c}$) Yang–Mills theory, the potential must have a regular expansion in the limit $\lambda \rightarrow 0$,

6.6 $$\begin{aligned} V(\lambda )\simeq {12\over \ell ^2}\left[ 1+V_1\lambda +V_2\lambda ^2+\mathcal{O}(\lambda ^3)\right] , \end{aligned}$$

where the coefficients $V_i$ are in one-to-one correspondence with the perturbative $\beta$-function of the theory, $\beta (\lambda )$. The long distance (IR) asymptotic behavior, $V(\lambda )\sim \lambda ^{4\over 3}\sqrt{\log \lambda }$, is responsible for the presence of a mass gap and a linear glueball spectrum as $\lambda \rightarrow \infty$ [1964]. Fitting $V_1$ and $V_2$, it is possible to accurately reproduce both the $T=0$ glueball spectrum and the thermodynamic behavior [2247]. This is demonstrated in Fig. 78, where the trace anomaly in IHQCD is compared to high-precision lattice results, evaluated for several values of $N_\mathrm{c}$ [2248, 2249].

In addition to bulk thermodynamic quantities, several transport coefficients have been determined in the deconfined phase of IHQCD. While the shear viscosity to entropy ratio is found to be the same as in ${\mathcal N}=4$ SYM theory, the bulk viscosity, $\zeta$, is also finite in IHQCD [2250]. Recently, these calculations have been extended to cover the full frequency dependence of the corresponding spectral densities [2242, 2251, 2252], revealing good agreement with lattice data. The Chern–Simons diffusion rate has also been determined within IHQCD and shown to be about 30 times larger than previous estimates based on ${\mathcal N}=4$ SYM [2253].

Finally, (unquenched) flavor dynamics have recently been added to IHQCD in the Veneziano limit [2254] and the conformal phase transition identified as a Berezinsky–Kosterlitz–Thouless-type topological transition. Preliminary investigations of the corresponding spectra have indicated the presence of Miransky scaling,^18^ the absence of a dilaton in the walking regime, and the presence of a substantial $S$ parameter^19^ [2255]. The finite-temperature phase diagrams have the expected forms with an additional surprise in the walking regime [2256].

Beyond IHQCD, the finite density landscape of QCD has been studied by extending the model of Ref. [2246] by the addition of an extra U(1) gauge field [2257, 2258]. In particular, it was found that the phase diagram exhibits a line of first-order phase transitions which terminates at a second-order critical endpoint, much as expected in $N_\mathrm{c} = 3$ QCD.

### Impact of thermal field theory calculations on cosmology

Systematic techniques developed for problems in hot QCD may find direct or indirect use in cosmology. Some particular cases are detailed in the following.

In cosmology, one compares the rate of expansion of the universe with the equilibration rate. The expansion (or Hubble) rate is determined from the equation of state of the matter that fills the universe via the Einstein equations. The equilibration rate depends on the microphysical processes experienced by a particular excitation. Cosmological “relics”, such as dark matter or baryon asymmetry, form if a particular equilibration rate falls below the expansion rate. For example, the cosmic microwave background radiation arises when photons effectively stop interacting with the rest of the matter. A cartoon equation for these dynamics is

6.7 $$\begin{aligned} \dot{n} + 3 H n = - \Gamma (n - n_{eq}) + O(n - n_{eq})^2 , \end{aligned}$$

where $n$ is the relevant number density, $n_{eq}$ is its equilibrium value, $H$ is the Hubble constant and $\Gamma$ the microscopic interaction rate.

Similar rates also play a role in heavy-ion experiments: the QCD equation of state determines the expansion rate of the system while microscopic rates determine how fast probes interact with the expanding plasma.

An apparent difference between cosmology and heavy-ion collisions is that, in the former, weak and electromagnetic interactions play a prominent role whereas, in heavy-ion collisions, strong interactions dominate. However, in a relativistic plasma even weak interactions become strong: obtaining formally consistent results requires delicate resummations and, even then, the results may suffer from slow convergence.

The development and application of resummation techniques in hot QCD or cosmology can benefit both fields. For example, techniques [2259] originally applied to computing the QCD equation of state [2260] have been employed to compute the equation of state of full Standard Model matter at very high temperatures [2261, 2262]. Techniques for computing transport coefficients [2263, 2264] have led to the determination of some friction coefficients in cosmology [2265, 2266]. Techniques developed for computing the photon/dilepton production rate from a hot QCD plasma near [2022] or far from [1905] the light cone can be applied to computation of the right-handed neutrino production rate in cosmology [2267–2270]. (In some cases, such as the rate of anomalous chirality changing processes or chemical equilibration of heavy particles, methods originating in cosmology [2271, 2272] were later applied to heavy-ion collisions [2273, 2274].) Also in these cases it may help to combine different QCD effective field theories (EFTs). In [2275] an EFT for nonrelativistic Majorana particles, which combines heavy-quark EFT and Hard Thermal Loop EFT, has been developed and applied to the case of heavy Majorana neutrino decaying in a hot and dense plasma of Standard Model particles, whose temperature is much smaller than the mass of the Majorana neutrino but still much larger than the electroweak scale. It may have applications to a variety of different models involving nonrelativistic Majorana fermions.

Apart from these methodological connections, there are also direct physics links between hot QCD and cosmology. In these cases, QCD particles do not themselves decouple from equilibrium: their collective dynamics provides a background for the evolution of other perturbations present in the medium. For instance, the QCD epoch could leave an imprint on the gravitational wave background [2276], or on the abundance of cold [2277] or warm [2278] dark matter. In the case of dark matter, not only the equation of state but also various spectral functions, estimated from lattice simulations [1897], could play a role [2279].

An example of an outstanding issue in cosmology is a first-principles “leptogenesis” computation of right-handed neutrinos in different mass and coupling regimes. It would be interesting to find hot QCD analogs for such CP-violating phenomena.

### The chiral magnetic effect

Parity (P) as well as its combination with charge conjugation (C) are symmetries known to be broken in the weak interaction. In the strong interactions, however, both P and CP are conserved except by the $\theta$ term, making the strong CP problem one of the remaining puzzles of the Standard Model. The possibility to observe parity violation in the hot and dense hadronic matter produced in relativistic heavy-ion collisions has been discussed for many years [2280].

In the vicinity of the deconfinement phase transition, the QCD vacuum could create domains that could introduce CP-violating effects [2280]. For a critique regarding the observability of those effects in heavy-ion collisions see [2281]. These effects could manifest themselves as charge separation along the direction of the angular momentum of the system or, equivalently, along the direction of the strong magnetic field, $\approx 10^{18}$ G, created in semi-central and peripheral heavy-ion collisions perpendicular to the reaction plane. This phenomenon is known as the chiral magnetic effect (CME). Due to fluctuations in the sign of the topological charge of these domains, the resulting charge separation, averaged over many events, is zero. This makes the observation of the CME possible only in P-even observables, expressed by correlations between two or more particles.

The CME has been studied both at RHIC and LHC employing the three-particle correlator $\langle \cos (\varphi _\alpha + \varphi _\beta - 2\Psi _\mathrm{RP}) \rangle$. Here $\varphi _i$ is the azimuthal emission angle of particles with charge or type $i$ and $\Psi _\mathrm{RP}$ is the orientation of the reaction plane. The correlator probes the magnitude of the expected signal while concurrently suppressing background correlations unrelated to the reaction plane.

The STAR Collaboration published the first results from Au+Au collisions at $\sqrt{s_{NN}} = 0.2$ TeV, consistent with CME predictions [2283]. ALICE has studied these same correlations at midrapidity in Pb+Pb collisions at $\sqrt{s_{NN}} = 2.76$ TeV [2282]. The ALICE analysis was performed over the full minimum bias event sample recorded in 2010 (${\sim } 13$M events).

**Fig. 79:**
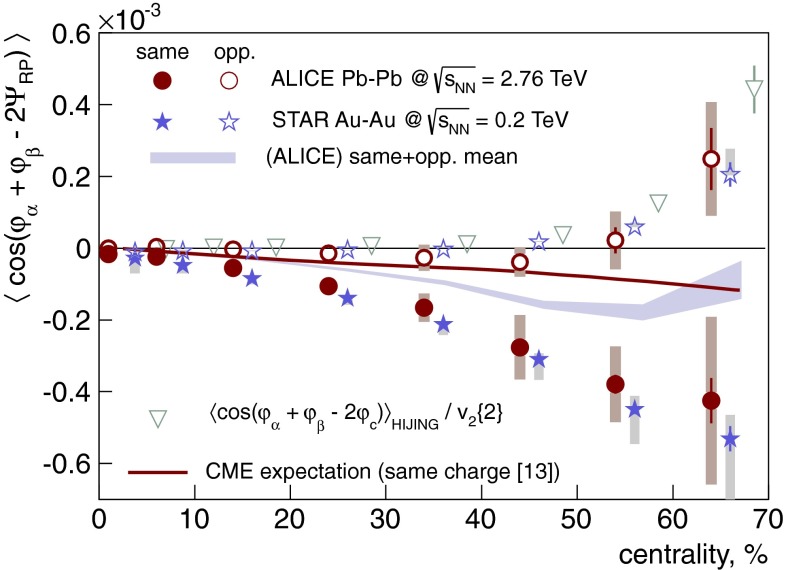
The centrality dependence of the correlator $\langle \cos (\mathsf {\varphi }_{\alpha } + \mathsf {\varphi }_{\beta } -2\Psi _\mathrm{RP}) \rangle$. From [2282]

Figure 79 presents the correlator $\langle \cos (\varphi _{\alpha } + \varphi _{\beta } - 2\Psi _\mathrm{RP}) \rangle$ measured by STAR and ALICE as a function of the collision centrality compared to model calculations. The ALICE points, filled and open circles for pairs with the same and opposite charges, respectively, indicate a significant difference not only in the magnitude but also in the sign of the correlations for different charge combinations, consistent with the qualitative expectations for the chiral magnetic effect. The effect becomes more pronounced in peripheral collisions. The earlier STAR measurement in Au+Au collisions at $\sqrt{s_{NN}} = 200$ GeV, represented by stars, is in good agreement with the LHC measurement.

The solid line in Fig. 79 shows a prediction of same-sign correlations due to the CME. The model does not predict the absolute magnitude of the effect and describes the energy dependence from the duration and time evolution of the field. It significantly underestimates the magnitude of the same-sign correlations at the LHC [2284]. Other recent models suggest that the magnitude of the CME might be independent of energy [2285, 2286].

Other effects, unrelated to the CME, may also exhibit a correlation signal. Results from the HIJING event generator, which does not include P violation, are also shown (inverted triangles), normalized by the measured value of $v_2$. Because no significant difference between same and opposite-sign pair correlations is present in the model, they are averaged in Fig. 79. The finite effect in HIJING can be attributed to jet correlations, unrelated to the reaction plane. Another possible explanation for the behavior of the correlator comes from hydrodynamics. If the correlator has an out-of-plane, charge independent, component arising from directed flow fluctuations, the baseline could be shifted [2287]. The sign and magnitude of these correlations is given by the shaded band in Fig. 79.

The measurements, including a differential analysis, will be extended to higher harmonics and identified particle correlations. These studies are expected to shed light on one of the remaining fundamental questions of the Standard Model.

The CME also occurs within AdS/CFT approaches. In AdS/CFT the CME is closely related to an anomaly [2288]. A related observable is the chiral vortical effect where the angular momentum of non-central collisions generates helicity separation between particles. This appears naturally from supergravity within AdS/CFT [2289, 2290]. The chiral vortical effect can also arise from a current generated in the presence of a gravitational vortex in a charged relativistic fluid and was found to be present even in an uncharged fluid [2291, 2292]. In the case of two U(1) charges, one axial and one vector, the CME appears formally as a first-order transport coeffcient in the vector current. In this case, there is evidence that the CME depends on $v_2$ [2293]. Finally, topological charge fluctuations can generate an axial chemical potential that splits the effective masses of vector mesons with different circular polarizations in central heavy-ion collisions, complementary to the CME in noncentral heavy-ion collisions [2294, 2295].

### Future directions

Based on the current results and open questions, as detailed in this chapter, several of the main experimental issues can be addressed in the short to medium term using the facilities currently in operation. An extended list of experimental measurements has to await long-term upgrades and planned new facilities. The driving force behind the forthcoming developments in heavy-ion physics are the existing and planned experimental heavy-ion programs. These include the collider experiments at the LHC and RHIC, as well as future programs at fixed-target facilities either under construction or in the planning stage. The physics opportunities and goals are somewhat different at each facility, offering a complementarity that can be exploited.

Indeed, after the first 2 years of ion runs at the LHC and further results from RHIC, the field has made significant progress. Detailed, multi-differential measurements have shown that the produced system can still be described by hydrodynamics in the new energy domain of the LHC. Thus its bulk macroscopic properties can be characterized. Moreover, significant progress has been made in determining the microscopic properties of the QGP (shear viscosity and plasma opacity) with increased precision. Detailed studies of identified particles and extended measurements of heavy flavors have introduced new input to the topics of thermalization and recombination.

In addition, the study of proton–proton and proton–nucleus collisions, used as reference baselines for comparison to heavy-ion results, also revealed some unexpected findings. The very first discovery at the LHC was related to the appearance of the “ridge” structure in high multiplicity $pp$ events, associated with long-range correlations. A similar but much stronger feature appeared in the proton–nucleus data, attracting great interest. It is clearly very important to identify whether the ridge phenomena is of hydrodynamic or saturation origin and how it relates to similar phenomena occurring in nuclear collisions. While saturation physics can explain the qualitative appearance of the ridge phenomena in proton–proton and proton–nucleus collisions, hydrodynamic flow could further collimate the signal, particularly in nucleus-nucleus collisions [2296].

In particular, novel, high-resolution methods to probe the early times of the evolution of $AA$ collisions, developed at RHIC and the LHC, need to be pursued with higher statistics and greater precision. The corresponding observables are parton attenuation in the early partonic medium, and higher flow moments. The latter are being extended by the analysis of single events which reflect the primordial evolution without the ensemble averages that can blur the resulting picture. Such measurements, as well as sophisticated developments from both the theoretical and experimental sides have brought some fundamental aspects of QCD within reach. First, it has become possible to probe a primordial phase founded on gluon saturation physics—the so-called Color Glass Condensate (CGC) that arises at asymptotically high gluon densities.

Next, it was recognized that the QGP is not a weakly coupled parton gas but a strongly coupled, near-ideal liquid with a very low ratio of (shear) viscosity to entropy density ($\eta /s$), close to the theoretical lower limit derived from quantum gauge field theory.

The attenuation of leading partons in the medium is characterized by the parton transport coefficient $\hat{q}$ which quantifies the medium-induced energy loss. The fundamental quantities of $\eta /s$ and $\hat{q}$ are related, as detailed in Ref. [2297]: a large value of $\hat{q}$ implies a small $\eta /s$, indicative of strong coupling. Moreover both quantities can be addressed within the so-called AdS/CFT conjecture [2298, 2299], by a dual weakly coupled string theory.

The experimental methods and avenues of theoretical research, pioneered at RHIC, could reach their full promise at the LHC with further increases in luminosity and runs at the top design energy, leading to greater precision for drawing crucial theoretical conclusions.

Indeed, within the currently approved LHC schedule, an order of magnitude higher statistics is expected to be collected, necessary for the description of statistics-limited phenomena such as the differential study of higher harmonic particle flow and high $p_{T}$ “jet quenching”.

The measurements and the conclusions reached employing jets arising from high-energy partons revealed the richness of these high $p_{T}$ probes which access not only the properties of the medium, but also properties of the strong interaction. Jets put constraints on the amount of energy loss in the medium and the dependence on the parton type which can disfavor some current models. At the LHC, jets are more clearly defined and better separated from the background, both in single and in dijet production than at RHIC, due to the larger cross section for hard processes. However, the correlation between hard jets and soft particles in the underlying event remains difficult to describe by any currently known mechanism, even if it turns out to be factorizable in QCD. In-depth studies of the energy redistribution within a jet or of the low $p_{T}$ particles emitted far away from the jet axis, together with the precise description and modeling of the modification of the jet fragmentation functions and jet shape, could unveil the properties of the QGP. These studies could also clarify why, in the case of high $p_{T}$ jet suppression, jet cone radii of up to $R=0.8$ are unable to capture all the radiated energy. A better understanding of the large average energy imbalance, also seen in the golden $\gamma +$jet channel, and of the angular correlations between jets that are, surprisingly, not strongly modified in the range $40 < p_\mathrm{T} < 300$ GeV/$c$, could be obtained by extensive studies of dijet events.

In addition, the higher luminosity will enable precision studies of quarkonium suppression, dramatically increasing our understanding of the interactions of hard particles with the thermal medium. Finally, the study of the thermalization and chiral symmetry restoration will be considerably enhanced by measurements of thermal dilepton and photon radiation, as well as the determination of vector meson spectral functions.

Furthermore, recent developments have advanced our knowledge of $AA$ collisions at comparatively modest center of mass energies, where lattice QCD predictions at finite baryon chemical potential $\mu _B$ locate the parton-hadron phase boundary. First indications of a critical point need to be clarified by further systematic studies. Such investigations are also fundamental for the characterization of the QCD phase diagram.

Complementary research is planned and is being conducted at lower center of mass energies and temperatures. Data from the beam energy scan at the RHIC collider at larger baryon densities will contribute to the search for a critical point on the QCD phase diagram. New fixed-target experiments will increase the range of energies available for the studies of hot, baryon-dense matter. The CERN SPS will remain the only fixed-target facility capable of delivering heavy-ion beams with energies greater than 30 GeV/nucleon, making studies of rare probes at these energies feasible.

At the FAIR accelerator complex under construction at GSI, Darmstadt, heavy-ion experiments are being prepared to explore the QCD phase diagram at high baryon chemical potential with unprecedented sensitivity and precision. Finally, the NICA project at JINR, Dubna, will complement these programs. In particular, these new low-energy facilities are being built to study compressed baryonic matter at high baryon density and (comparatively) low temperatures where the matter may undergo a first-order phase transition. In these systems, the produced matter is more closely related to neutron stars.

On the theory side, important progress is expected in both phenomenology and pure theory. A well-coordinated phenomenological effort is clearly needed to fully exploit the current and future precision data from the facilities mentioned above.

Indeed, the new reference data from $p$+Pb collisions at the LHC have presented some unique challenges for phenomenology. Potential new QCD phenomena could be unveiled by solving the ridge puzzle in $p$+Pb collisions. One promising proposed method is to employ multiparticle methods in order to access and measure collective phenomena which can discriminate between initial-state (CGC) and final-state (hydro) mechanisms.

The behavior of the low-$x$ gluon density in nuclei needs to be better understood, both in the shadowing and saturation pictures. In addition, the question of whether the high-multiplicity events in $pp$ and $pA$ collisions can be described in terms of cold nuclear matter or whether they should be thought of as having created a hot medium is one that will come to the fore.

In addition to phenomenology, establishing the quantitative properties of a deconfined quark–gluon plasma from first principles, both in and out of thermal equilibrium, continues to be a fundamental theory goal, requiring a combination of lattice, perturbative and effective field theory methods. In this context, the major challenges will be to extend equilibrium thermodynamic calculations on the lattice to larger quark densities; to obtain accurate non-perturbative predictions of the QGP transport properties; and to further increase understanding of the dynamics that lead to the apparent early thermalization in heavy-ion collisions.

Putting the heavy-ion program in a broader context, the LHC is the high-energy frontier facility not only of particle physics but also of nuclear physics, with an extensive, well-defined program. The active RHIC program, complementary and competitive, continues to map the phase diagram of nuclear matter at lower temperatures pursuing the search for a tricritical point.

Continuation and strengthening of the SPS fixed-target program is under discussion. Furthermore, the two new low-energy facilities (FAIR at GSI and NICA at JINR) are being built to explore the part of the phase diagram at the other extreme from the colliders. Thus, the global heavy-ion physics program can fully map the QCD phase diagram, spanning these two limits. Thus strongly interacting matter under extreme conditions, such as those prevailing in the early universe (LHC, RHIC) as well those similar to the conditions in the interior of neutron stars (FAIR, NICA) can be studied in the laboratory.

In summary, the exploration of the phases of strongly interacting matter is one of the most important topics of contemporary nuclear physics. The study of strong-interaction physics, firmly rooted in the Standard Model, has already brought surprises and discoveries as well as showcased the potential of heavy-ion research which is expected to keep on providing new and interesting results.

## Nuclear physics and dense QCD in colliders and compact stars

20In this chapter we discuss open problems and future directions in nuclear physics (addressing issues concerning dense nuclear matter as well as low-density and vacuum nuclear interactions) and high-density quark matter, both of which are relevant for the physics of compact stars. The composition of the inner core of a compact star is not known. Constraints on the Equation Of State (EOS) of the star core are imposed by the measured radii and masses, but several scenarios are possible. These scenarios vary from considering only neutrons and protons as constituting the inner core, assuming the presence of hyperons, or a kaon condensate, or the existence of a dense quark matter core. These different hypotheses are discussed from an experimental and theoretical point of view in the following subsections. The chapter is divided into three subsections. In Sect. 7.1 we focus on accelerator experiments that can shed light onto kaon–nucleon and hyperon–nucleon interactions in a dense medium and the implications for neutron stars, such as the thickness of the neutron star crust via measurements of neutron-rich nuclei. In the second part, Sect. 7.2, we discuss theoretical attempts to understand the nucleon–nucleon interaction from QCD. In particular, we address promising directions in lattice QCD, effective field theory methods, and the large-$N_\mathrm{c}$ approach. Finally, in Sect. 7.3, we mostly discuss dense quark matter, starting from asymptotic densities. We discuss several theoretical approaches and come back to compact stars to address various astrophysical observables and their relation to the microscopic physics of dense matter.

### Experimental constraints on high-density objects

The study of high density objects can be pursued among other methods by investigating hadron-hadron collisions at accelerators. On the one hand, heavy-ion collisions at moderate kinetic energies ($E_\text {KIN}=1$–8 AGeV^21^) lead to the formation of a rather dense environment with $\rho =2\hbox {--}7 \rho _0$ (with $\rho _0=\,0.172 \,\mathrm {fm^{-3}}$ being the normal nuclear density) which can be characterized in terms of its global properties and the interactions among the emitted particles. Normally, the density reached in the collisions as a function of the incoming energy is extracted from transport calculations. In these kinds of experiments, one of the goals is to determine the EOS for nuclear matter and extract constraints for the models of neutron stars. On the other hand, the understanding of the baryon–baryon and meson–baryon interaction as a function of the system density should be complemented by the study of elementary reactions that give access to the interaction in the vacuum and serve as a fundamental reference. Important references are delivered by the measurement of kaonic-atoms and hypernuclei, as described in the following paragraphs. Aside from the measurement of strange hadrons, novel measurements of the properties of neutron-rich nuclei can constrain the thickness of the external crust of neutron stars.

#### Results from heavy-ion collisions

The EOS for nuclear matter relates the pressure of the system to its internal energy, density, and temperature and is fundamental for the modelling of different astrophysical objects. Indeed, by knowing the EOS of a certain state of matter, hypotheses about the content of dense astrophysical objects can be put forward and the mass to radius relationship can be extracted starting from the EOS and exploiting the Tolman–Oppenheimer–Volkoff equations [2300]. A more detailed description of the extraction of the mass and radius of neutron stars is given in Sect. 7.3. From the experimental point of view, one of the tools used to study dense systems are heavy-ion collisions at accelerator facilities. Transport calculations [2301] indicate that in the low and intermediate energy range ($E_\mathrm {lab}$ =  0.1–2 AGeV) nuclear densities between 2 and 3 $\rho _0$ are accessible while the highest baryon densities (up to $8\rho _0$) can be reached increasing the beam kinetic energy up to $10\,\mathrm {GeV}$. The EOS of nuclear matter is normally characterized by the incompressibility parameter which is expressed as:

7.1 $$\begin{aligned} K=\left. \, 9\rho _0^2 \frac{\mathrm{d}^2E}{\mathrm{d}\rho ^2}\right| _{\rho =\,\rho _0}. \end{aligned}$$

Hence, if the system energy is parametrized as a function of the system density, the parameter $K$ represents the curvature of this function at normal nuclear density and is a measure of the evolution of the system energy as a function of the density. The boundary between a soft and a stiff EOS is set around a value of $K=\,200\,\mathrm {MeV}$, with values below and above $200\,\mathrm {MeV}$ corresponding to a soft and stiff EOS respectively, with predictions for a rather stiff EOS corresponding to $K=\,380\,\mathrm {MeV}$ [2302]. Increasing the stiffness of the EOS translates into an increased pressure of the system. The experimental variables used to characterize the EOS are linked to the system pressure.

The collective properties of the fireball formed in heavy-ion collisions for different kinetic energies are linked to the system pressure and they have been studied to derive the compressibility of nuclear matter at the achieved aforementioned densities [2302]. The compressibility of the matter formed right after the ion’s collision can be related to the variation of the mean value of the $x$-component (assuming that the $z$-component is parallel to the beam direction) of the particles momenta. A larger resulting pressure on the emitted particles correspond to a stiffer EOS and also to larger values of the sideward forward-backward deflection parameter $F$ that measures the variation of the average $p_X$ component.^22^

**Fig. 80:**
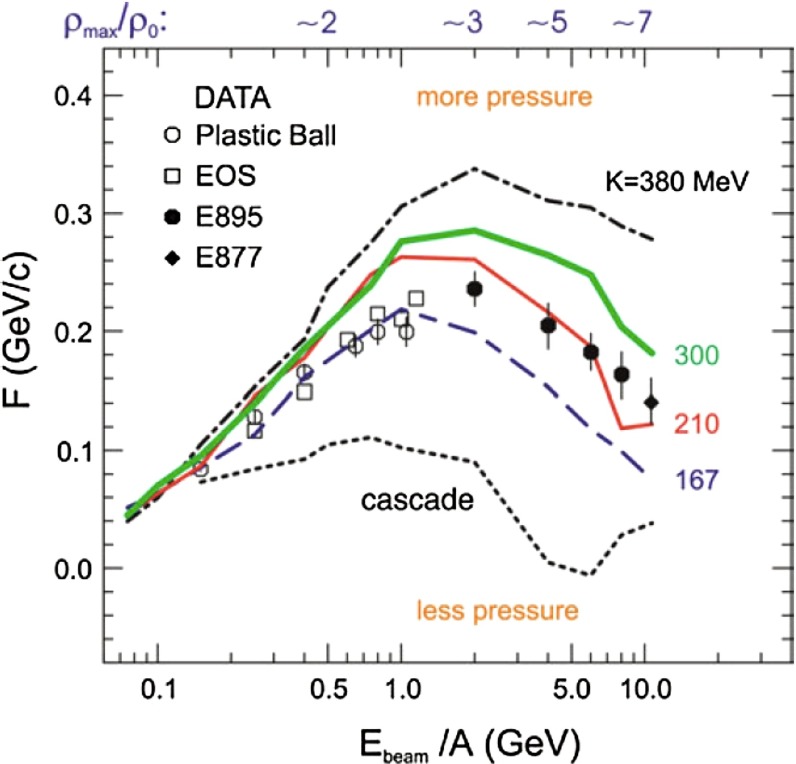
Sideward flow excitation function for Au+Au collisions. Data and transport calculations are represented by *symbols* and *lines*, respectively [2302]

Figure 80 shows the measure of the sideward forward-backward deflection $F$ for charged particles produced in Au+Au collisions for several beam energies. The maximal density reached for each setting is indicated in the upper horizontal axis. Lines represent simulations assuming different EOS and the comparison to the data points favors a compressibility parameter of $K\approx$ 170–210 MeV which translates into a rather soft EOS for normal nuclear matter. One can see that a single EOS is not sufficient to reproduce all the experimental data and that the stiffness of the system increases as a function of the density. This observation suggests that a the EOS of nuclear matter could depend on the system density and a transition from a softer to a stiffer EOS might occur.

The extraction of the EOS from the measurement of the flow of charged particles produced in heavy-ion collisions is limited by the following factors. The different transport models, that are used to compare the experimental observables measured in heavy-ion collisions at intermediate energy ($E_\text {KIN}<\,10$ AGeV), solve the Boltzmann transport equation with the inclusion of a collisional term modeling the heavy-ion collisions as the superposition of binary nucleon–nucleon interactions. Normally a solid knowledge of the elementary nucleon–nucleon, $\Delta$–nucleon and $\pi$–nucleon cross sections is needed as an input for transport models. Unfortunately, the processes involving a $\Delta$ or a neutron are either not measurable exclusively or have not been measured yet. Moreover, one has to consider that the particle equations of motion in transport models generally do not contain any dependence on the system temperature, which is certainly not negligible in heavy-ion collisions. The maximal temperature of the colliding systems can be estimated via statistical models of particle hadronization, and it already reaches $100\,\mathrm {MeV}$ for $E_\text {KIN}=\,1$–2 GeV [2303].

The properties of kaons ($K$) and antikaons ($\bar{K}$) in the nuclear medium have also been the object of numerous investigations, since the possible existence of a $\bar{K}$ condensed phase in dense nuclear matter and thus in the interior of compact neutron stars was pointed out by Kaplan and Nelson [2304].

This idea originates from the fact that various theoretical approaches, based on effective chiral models where $K$/$\bar{K}$ and nucleons are used as degrees of freedom, agree qualitatively in predicting density-dependent modifications in mass and coupling constants for $K$ and $\bar{K}$. This results in the growth (drop) of the effective mass of $K$ ($\bar{K}$) with increasing nuclear matter density [2305–2307]. Indeed, the scalar part of the mean potential is attractive for both $K$-types , while the vector part is slightly repulsive for $K$ ($V=20$–30 MeV at $\rho =\rho _0$ and ${\overrightarrow{p}}=\,0$) and yet attractive for $\bar{K}$ ($V=-$50–150 MeV at $\rho =\rho _0$ and ${\overrightarrow{p}}=\,0$). The effective $\bar{K}$ mass is then expected to undergo a substantial reduction in the presence of dense nuclear matter, up to the point where strangeness-violating decays of protons into neutrons, $\bar{K}$ and neutrinos occur. This process might set in starting at rather high baryonic densities ($\rho =3\hbox {--}4~\rho _0$) and could lead to the creation of an equilibrated condensate of $\bar{K}$ and neutrons in the interior of neutron stars.

The investigation of the kaon properties produced in heavy-ion collisions at intermediate energy was successfully carried out in the 1990s by the KaoS [2308] and FOPI [2309] collaborations and more recently by the HADES collaboration [2310] by measuring heavy-ion collisions with beam kinetic energies up to $\mathrm {1.9\,GeV}$. In this energy regime, strange hadrons are produced to a large extent below the nucleon–nucleon energy threshold and hence mainly by secondary collisions, which are used as reservoirs to gather the necessary energy to produce strange hadrons. These secondary collisions are more probable during the initial, denser phase of the collision, where the baryonic matter undergoes the highest compression and hence strange hadrons are highly sensitive to possible repulsion/attraction in dense baryonic matter [2311]. The nuclear matter EOS determined by comparing the $K$ multiplicities produced in heavy (Au+Au) and light (C $+$ C) colliding systems as a function of the kinetic energy is found to be rather soft [2312]. Indeed, the $K^+$s are produced in the initial dense phase of the collisions and since they do not undergo absorption, because the imaginary part of their spectral function is rather small, the scaling of the production rate with increasing incoming kinetic energy, which corresponds to an increasing compression of the system, can be used to tag the compressibility parameter and hence the EOS. Additionally to the effects linked to the compressibility of pure nuclear matter, by looking at the $K$ observable one should consider the effect of the repulsive potential between nucleons and $K$. It is very difficult to disentangle the properties of nuclear matter and its interaction with $K$ and $\bar{K}$ with the same observable. This is the reason why in addition to the $K$ yields other observables have also been taken into account.

The standard method to study the potential effects on $K$ and $\bar{K}$ production consists in analyzing either their collective flow or in the study of the $p_\mathrm{T}$ spectra^23^ in different rapidity intervals [2309, 2313]. First we discuss the flow observable. Taking as a reference the reaction plane formed by the distance of closest approach of the two colliding nuclei and the beam direction, the particle emission angle with respect to this plane is considered. The azimuthal anisotropies in the collective expansion, also called anisotropic flow, are usually characterized by a Fourier expansion of the azimuthal distribution of the produced particles:

7.2 $$\begin{aligned} v_n=\,\langle \langle \cos n(\phi -\Psi _R) \rangle \rangle , \end{aligned}$$

where $\Psi _R$ and $\phi$ represent the orientation of the reaction plane and the azimuthal angle of the particle with respect to the reaction plane. The two averages of the $\cos$ function run over the number of particle per event and over the total number of events. The resulting parameters $v_1$ and $v_2$ are known as direct and elliptic flow, respectively. Direct and elliptic flow for $K$ and $\bar{K}$ have been recently measured by the FOPI  [2314] collaboration in Ni+Ni collisions at $\mathrm {1.9\,GeV}$ kinetic energy and compared to the different transport models. Preliminary results show that the expected sensitivity of the direct flow $v_1$ in the target region is weaker than predicted by transport calculations including a strongly attractive $\bar{K}N$ potential. For $K$ the qualitative behavior of $v_1$ is described by the transport models including a slightly average repulsive potential of $20\,\mathrm {MeV}$. One has to mention here that the major limitation of the transport models in the description of $K$ and $\bar{K}$ flow is the fact that the momentum dependence of $v_1$ and $v_2$ is far from being well modelled for this energy regime [2301]. One has to consider that the approximation made so far by the transport models used for these comparisons, in which the interacting $K$-nucleus potential depends linearly upon the system density, is certainly much too simplistic. In this respect recent developments of the GiBUU [2315] model includes a more realistic chiral potential for the $K$, but first tests are only now being carried out with data extracted from proton-induced collisions. The next step would be to extend this model to heavy-ion collisions. To summarize the $K$ and $\bar{K}$ flow results a slight repulsive potential is confirmed for the $K$ produced in heavy-ion collisions at intermediate energies but unfortunately no evidence of a strongly attractive potential for $\bar{K}$ could be observed within the statistical sensitivity of the data.

The doubly differential analysis of the $p_\mathrm{T}$ spectra for $K$ shows a better consistency. There, by looking at the experimental $p_\mathrm{T}$ distributions for different rapidity intervals, a systematic shift towards higher momenta is observed [2310, 2316]. This shift is thought to be due to the repulsive potential felt by $K$ in the nuclear medium. So far, the experimental findings about the $p_\mathrm{T}$ distributions indicate a repulsive average potential for $\mathrm {K^0}$ and $\text {K}^+$, estimated to be between $\mathrm {20\,MeV}$ [2316] and $\mathrm {40\, MeV}$ [2310] at $\rho =\,\rho _0$ but stays rather controversial for $K^{-}$ [2305]. Further measurements with $\pi$-beams planned at GSI already in 2014 and at JPARC starting from 2013 should allow a more quantitative determination of this potential. In particular, elementary reactions are needed to provide the transport models with solid inputs for all reaction channels.

It has been mentioned that the understanding of the $\bar{K}$ properties in dense nuclear matter is still far from being properly tagged down. So far the scarce amount of data for $\bar{K}$ produced in heavy-ion collisions at intermediate energies has hampered this study. The existing data by the FOPI collaboration about the $\bar{K}$ flow are unfortunately not definitive, as mentioned above. More experiments are needed in this direction.

Meanwhile, new theoretical developments have been carried out towards a more realistic treatment of the $\bar{K}$ in-medium spectral function [2317]. There, unitarized theories in coupled channels based on chiral dynamics [2318, 2319] and meson-exchange models [2320] are discussed, with particular emphasis on the novel inclusions of higher-partial waves beyond the s-wave in the meson–baryon coupling [2321]. In such calculations all possible meson–baryon coupled channels are considered to compute the final $\bar{K}$ spectral function in the medium, including effects such as the Pauli blocking in medium, and the self-consistent consideration of the $\bar{K}$ self-energy, the self-energies of the mesons and baryons in the intermediate states. Within this approach, an attraction of the order of $-50\,\mathrm { MeV}$ at normal nuclear matter density is obtained. This kind of calculations should be implemented in transport models to extract predictions for the $\bar{K}$ properties as a function of the system density.

Unfortunately in other approaches the low density approximation is employed to describe the broadening of the imaginary part of the $\bar{K}$ spectral function. This approach does not suit the complex behavior expected for $\bar{K}$ in the medium [2319].

#### The $K$-nucleon interaction in vacuum

The issue of the $K$-nucleon interaction has also been addressed in an alternative way in recent years. Indeed, so-called kaonic bound states, formed from a $\bar{K}$ sticking to two or more nucleons, have been predicted by theory [2322] and shortly afterwards observed in experiments [2323, 2324]. This idea originates from the first studies that Dalitz and colleagues did on the intrinsic nature of the $\Lambda (1405)$ resonance in the 1960s [2325], when they proposed a description of this particle as a molecular state of a $\bar{K}$–p and $\mathrm {\pi }$–$\Sigma$ poles interfering with each other. Since the $\Lambda (1405)$ is, at least partially, a $\bar{K}$–p bound state, it was natural to investigate the possibility of adding one or more nucleons and still finding a bound state. The binding energy and the width of this so-called kaonic cluster would reveal the strength of the $\bar{K}$–nucleon interaction in vacuum. As far as the $\Lambda (1405)$ is concerned, several approaches based on chiral effective field theory do describe this resonance as a molecular system emerging naturally from coupled channels calculations of meson–baryon pairs with $\mathrm {S=\,-1}$ [2326, 2327]. These models are constrained above the $\bar{K}$N threshold by scattering data and by the very precise measurement of the $\bar{\mathrm {K}}p$ scattering length at the threshold extracted from the kaonic-hydrogen data measured by the SIDDHARTA collaboration [2328]. The underlying concept for such an experiment is to determine the shift and width of the ground levels in kaonic hydrogen and deuterium caused by the strong interaction between the $\bar{K}$ and the nuclei. Therefore, the X-rays emitted in transitions of the $\bar{K}$ to the ground level are measured. By comparing the measured X-ray energies with the values expected from QED only the strong interaction-induced shift and width are obtained. The measurement of the kaonic-deuterium is planned by the SIDDHARTA collaboration at DA$\Phi$NE after an upgrade of the experimental apparatus and will also be pursued at JPARC. These experiments will allow the determination of the isospin-dependent scattering lengths, currently strongly under debate from the side of theory.

To this end it is clear that within this approach the whole $\bar{K}$ dynamics in nuclear matter is strongly influenced by the presence of the $\Lambda (1405)$ resonance. Several experiments have been carried out, employing either stopped kaons and antikaons, or beams of these particles, real photons, and protons, to study the properties of the $\Lambda (1405)$ and to search for kaonic bound states. The molecular nature of the $\Lambda (1405)$ is supported by the observation of different spectral function distributions measured with different initial states [2329]. Different production mechanisms correspond indeed to different coupling strength of the poles leading to molecule formation.

The spectral shape of the $\Lambda (1405)$ resonance measured from its decay into $(\Sigma \pi )^0$ pairs has been reconstructed by the CLAS [2330] collaboration [511] for the reaction $\gamma +\text {p}\rightarrow \Lambda (1405) + \text {K}^+$ and for 9 different settings of the photon energy varying from $\mathrm {2}$ to $\mathrm {2.8\,GeV}$. The experimental data have been discussed in terms of phenomenological fits to test the possible forms and magnitude of the contributing amplitudes. Two $I=0$ amplitudes with an additional single $I=1$ amplitude parametrized with Breit–Wigner functions work very well to model all line shapes simultaneously. This comes as a surprise since the $I=0$ poles are very different than those obtained by coupled channel calculations [2326, 2327], and the existence of a bound state in the $I=1$ channel is very controversial. An alternative strategy has been proposed in [2331] to describe the CLAS data in the $\Sigma ^0\pi ^0$ decay channel by varying the chiral coefficients for the meson–baryon coupling amplitudes. This variation is motivated by the fact that higher-order calculations of the chiral amplitude (most of the models include only the Weinberg–Tomosawa term for the interaction) might lead to significant corrections. This empirical approach delivers a reasonable description of the data but does not explain the presence of the $I=1$ bound state in the charged decays.

The $\Lambda (1405)$ signal reconstructed from the reaction $\mathrm {p+p\rightarrow \Lambda (1405)+p+K^+}$ and the successive decay into the two charged channels $\Sigma ^{\pm }\pi ^{\mp }$ has been recently analyzed by the HADES collaboration [2332]. There, a shift of the spectral function associated to the $\Lambda (1405)$ has been observed and the maximum of the distribution is found $\mathrm {20\,MeV}$ lower than the nominal value of $1405\,\text {MeV}$. This effect, which strongly differs from the CLAS results, shows clearly the molecular character of the $\Lambda (1405)$ resonance and is yet to be fully understood from a theoretical point of view. It should be mentioned that the shifted pole towards lower masses might indicate that in the p+p entrance channel the $\pi \Sigma$ pole couples stronger to the $\Lambda (1405)$ formation [2333]. This is so far only a speculation that should be verified within a solid theoretical calculation, but could nevertheless strongly modify the $\bar{K}$ dynamic in the nuclear medium.

Following this line of thought and assuming that the $\bar{K}$–p pole dominates in the formation of the $\Lambda (1405)$, the smallest of the kaonic clusters ($\mathrm {ppK^{-}}$) could be obtained by adding an additional proton. The experimental evidence for kaonic bound states is partly strongly criticized but the signal measured with stopped $K$ by the FINUDA collaboration in the $\mathrm {\Lambda }$–p final state seems rather robust [2323]. One of the critical reviews of this work [2334] emphasizes the role played by the one nucleon and two nucleons absorption reactions (K$^{-} +\mathrm {N}\rightarrow \Lambda \hbox {--N--} \pi$ or K$^{-} +\mathrm {N}\rightarrow \Lambda \hbox {--N--N--}\pi$) and its contribution to the measured $\mathrm {\Lambda }\hbox {--}\hbox {p}$ final state. Recent measurement by the KLOE collaboration [2335] shows the feasibility of the exclusive measurement of the one-nucleon absorption. These results should be quantitatively compared to theoretical prediction and be included in the further analysis of the KEK [2336] and future JPARC experiments on this subject.

The findings by the FINUDA [2323] and DISTO [2324] collaborations about the signature of the smallest of the kaonic cluster $\mathrm {ppK^{-}}$ do agree on the reported value for the binding energy, which is found to be about $\mathrm {100\,MeV}$, but differ strongly on the state width ($60$ to $\mathrm {100\,MeV}$). The great majority of the theoretical models predict the existence of such cold and dense objects as well, but the landscape of the binding energies and widths is rather broad with intervals of 9–90 MeV and 35–110 MeV respectively [2337–2340].

Concerning the findings in p+p reactions, the contribution of the $\mathrm {N^*}$ resonance to the analyzed final state and the interference effects among different resonances has not yet been taken into account. A global study of the available $\mathrm {p+p \rightarrow p+\Lambda + K^+}$ data sets within a Partial Wave Analysis (PWA) should clarify the situation and quantify the contributions of the non-exotic and exotic sources to the final state. As far as the upcoming experiments at JPARC are concerned, two main issues should be mentioned. First of all, the one- and two nucleon absorption should be measured exclusively and, second, spin observables would be necessary to separate different contributions of the measured spectra.

Summarizing the situation for the $\bar{K}$ and its link to dense objects, the measured data show some evidence for a strong $\bar{\mathrm {K}}N$ binding but more quantitative information is still needed.

#### Hyperon–nucleon interaction

The hypothesis of a $\bar{K}$ condensate in neutron stars is complemented by a scenario that foresees hyperon production and coexistence with the neutron matter inside neutron stars. At present, the experimental data set on the $\Lambda$N and $\Sigma$N interactions consists of not more than 850 spin-averaged scattering events, in the momentum region from $200$ to $\mathrm {1500\, MeV}$, while no data are available for hyperon–hyperon scattering. This case can be approached by the measurement of hypernuclei. The $\Lambda$N effective interaction has been determined from reaction spectroscopy where hadronic final states are used to determine the hyper nuclei binding energies and $\gamma$-ray spectroscopy on the hyper nuclei decay. The reaction spectroscopy provides access to the central part of the $\Lambda$N potential at zero momentum while the fit of $\gamma$-ray data on p-shell hypernuclei allows the determination the contribution by the spin–spin term in the $\Lambda$N interaction [2341]. There, hypernuclei are produced employing secondary meson beams and primary electron beams, and the reaction spectroscopy results for several nuclei are consistent with calculations including an average attractive $\Lambda$-nucleus potential of $\mathrm {ReV_{\Lambda }\approx \,-30\,MeV}$, with $\mathrm {ReV_{\Lambda }}$ representing the real part of the optical potential. The spin-spin corrections depend on the nuclear species and amount to about $\mathrm {1\,MeV}$. Of particular interest in this context is the recent observation of the neutron-rich hypernucleus $^{6}_{\Lambda }H$ [2342]. Despite the fact that the measured yield amounts only to three events, the extracted binding energy allowed testing some models of the $\mathrm {\Lambda NN}$ interaction and excluding a strongly attractive one. Future measurements in this direction are planned at the JPARC facility where an unprecedented intensity of kaon-beams will be achieved in the next years. A different approach to study hypernuclei is to use projectile fragmentation reactions of heavy-ion beams. In such reaction, a projectile fragment can capture a hyperon produced in the hot participant region to produce a hypernucleus. Since a hypernucleus is produced from a projectile fragment, isospin and mass values of the produced hypernuclei can be widely distributed. The life-time and binding energies of the so-produced hypernuclei can be studied by the techniques developed in heavy-ion collisions experiments with fixed target set-ups. A pilot experiment reported in [2343] shows the feasibility of this technique. An extensive program based on this method is planned at FAIR (Facility for Antiproton and Ion Research). Sigma hypernuclei do not exist for times longer than $10^{-23}$ s, due to the strong $\mathrm {\Sigma -N\rightarrow \Lambda -N}$ conversion but the analysis of $\Sigma$ formation spectra [2344] shows that the average $\Sigma$–Nucleon potential is repulsive $\mathrm {ReV_{\Sigma }(\rho _0)\approx +(10-50) MeV}$. Moreover, that $\Lambda$ hyperons can be also analyzed in heavy-ion collisions too. The extracted kinematic variables can be then compared to transport models. So far, the results obtained at intermediate energies (Ni+Ni, $E_{\text {KIN}}=\,1.93$ AGeV) [2345] show that $\Lambda$-hyperons exhibit a different behavior compared to protons, if one looks at the flow pattern of the two particle species. Systematic studies as a function of the particle momentum and system density that could lead to the extraction of an interacting potential between the $\Lambda$-hyperon and the nucleons participating in the reaction have still to be carried out.

#### Implications for neutron stars

**Fig. 81:**
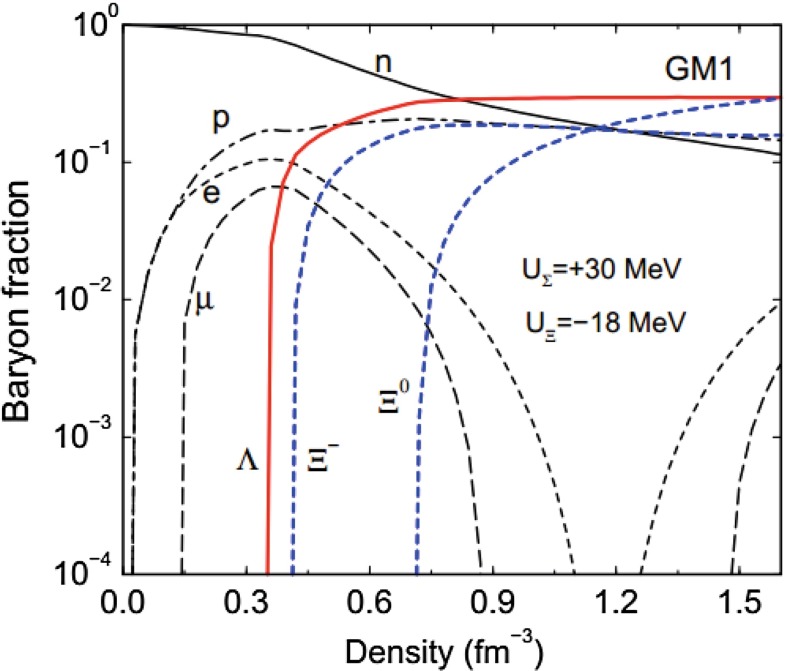
The fraction of baryons and leptons in neutron star matter for a RMF [2346] calculation with weak hyperon–hyperon interactions

Returning to neutron stars, the presence of $\bar{K}$s in their core would soften the EOS, leading to an upper limit for the maximal mass of the stars that is lower than the one observed. This way, we might also doubt the results about the compressibility of nuclear matter extracted from heavy-ion collisions. Indeed, most of the models used to describe these data do not contain an explicit dependence on the temperature of the system and in heavy-ion collisions the so called thermal contribution can influence the results. Two years ago the discovery of a neutron star of about two solar masses [2347] turned the situation upside down. Indeed, such massive neutron stars are neither compatible with a soft equation of state nor with the presence of a $\bar{K}$ condensate inside the star. In this context, a theoretical work [2348] suggests that the inner part of neutron stars is composed by normal nuclear matter and that the maximal densities reached for these objects do not exceed $4\,\rho _0$. Following this line of thought, a prediction of a rather stiff equation of state for normal nuclear matter has been put forward. The EOS for a finite system made only from neutrons and a small fraction of protons, including also three-body forces, has been calculated and the two solar-masses objects have been assigned a radius varying from $11$ to $\mathrm {14\, km}$ depending on the EOS constraints. The maximal density within neutron stars associated with this calculation does not exceed 3–4 $\rho _0$. Others suggest a transition from a soft to a stiffer EOS happening at densities between 3–5 $\rho _0$ [2349]. There the authors have stressed the importance of measuring the radius of small neutron stars, with mass near $\approx \,$1.4 solar masses, to verify the EOS for these systems, which density is supposed to be more compatible with conditions produced in heavy-ion collisions at intermediate energies. This scenario is very model dependent and mainly based on the compressibility extracted from kaon data. On the other hand, the hypothesis that only plain nuclear matter might constitute the core of the stars is in conflict with the very likely case that $\Lambda$ and $\Sigma$ hyperons might appear starting at densities around $\mathrm {3-\,\!-4\,\rho _0}$ and hence influence the EOS [2346]. Figure 81 shows the fraction of baryons and leptons as a function of the system density in neutron star matter. One can clearly see the appearance of the $\Lambda$ hyperons already at the density $\rho =\,\,2.3\,\rho _0$. Their presence should enhance the cooling of the neutron stars via direct Urca^24^ processes driven by hyperons, but in the case of large modifications of the hyperon mass in the dense environment a coexistence of neutrons and hyperons could be favored. The scenario with plain neutron-like matter up to large densities seems in this context rather improbable. On the other hand most probably, not antikaons but hyperons play a leading role in dense and cold systems, making the study of the interaction of the hyperons with nucleons as a function of the relative distance, temperature, and density of the surrounding system fundamental. Hyperons created in dense nuclear matter have already been studied extensively, but so far the kinematics of the hyperon reconstructed in heavy-ion collisions at intermediate energies [2303, 2345] was only compared to either the Boltzmann-like distribution to describe the kinematic freeze-out or to a statistical thermal model to infer upon the chemical freeze-out. The future perspectives, aside from the hypernuclei measurements, foresee a detailed analysis of the double differential kinematic observables ${p_\mathrm{T}},\,Y_{\mathrm{CM}}$ for $\Lambda$ hyperons produced in proton- and pion-induced reactions at kinetic energies around $2$ GeV and Au+Au collision at $1.25$ AGeV to extract the effect of the average $\Lambda$–nucleon interaction as a function of the system density. Moreover, $\Lambda$–p correlations can be studied in elementary reactions to infer on the distance dependence of the interaction which is so far not known at all.

#### Neutron-rich nuclei

**Fig. 82:**
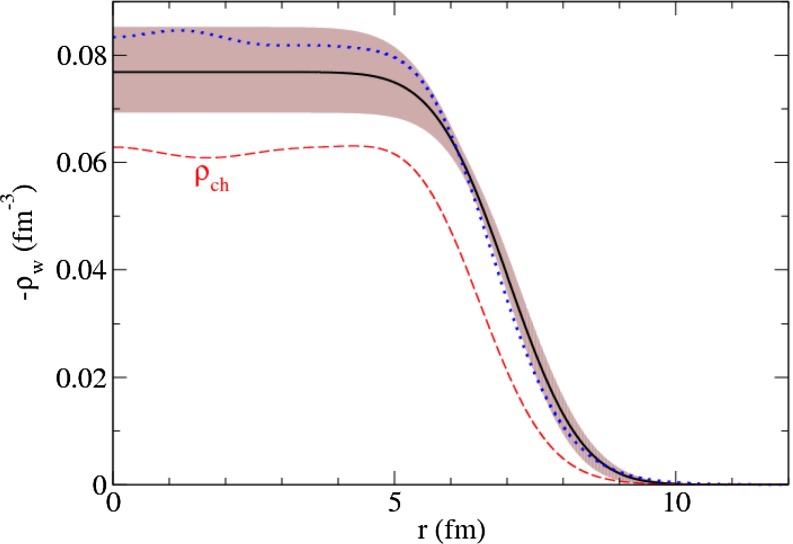
Weak charge density $\mathrm {\rho _W(r)}$ of $\mathrm {^{208}Pb}$ that is consistent with the PREX result (*solid black line*) [2352]. The *brown error band* shows the incoherent sum of experimental and model errors. The *red dashed curve* is the experimental (electromagnetic) charge density $\rho _{\text {ch}}$

The quest for the properties of neutron-rich matter and associated compact objects has also been addressed recently by parity violating scattering experiments with electron beams impinging on neutron-rich nuclei [2350]. This method has the advantage of being completely free from contributions by the strong interaction and provides a model independent probe of the neutron density in nuclei with a large neutron excess. By measuring the asymmetry in the scattering of electrons with different helicity, one can first extract the weak form factor. This is the Fourier transform of the weak charge density. Considering that the neutron weak charge is much larger than the proton weak charge, and applying corrections for the Coulomb distortion, the spatial distribution of the matter densities can be derived to the weak charge density. The black line in Fig. 82 shows the extracted weak charge density extracted within the Helm model [2351] on the base of the asymmetry measured by the PREX experiment at JLab [2352]. The brown error band shows the incoherent sum of experimental and model errors and the red dashed line shows the measured charge density [2353].

The point neutron density can be deduced from the weak charge density and the matter radius $R_{\text {n}}$ can be determined. The difference between the charge and matter radius of $\mathrm {^{208}Pb}$ and has found $R_{\text {n}}-R_{\text {p}}=\,0.33^{+0.16}_{-0.18}$ [2352], being $R_{\text {n}}$ and $R_{\text {n}}$ the matter and charge radii of the nucleus respectively. Future measurements are planned to reduce the error to $\mathrm {0.05\, fm}$. There is a strong correlation between the $R_{\text {n}}$ and the pressure in neutron stars at densities of $\frac{2}{3}\rho _0$, hence this measurement can constraint further the EOS of neutron-rich matter. Indeed a larger internal pressure in neutron stars would push the neutrons against the surface increasing $R_{\text {n}}$. For this reason an accurate measurement of the matter radius can better constraint the nuclear EOS, and in general, a larger value of $R_{\text {n}}$ is linked with a stiffer EOS. Moreover, the symmetry energy $s$ of nuclear matter, which plays an important role when one departs from a symmetric situation in the number of protons and neutrons, is also correlated with $R_{\text {n}}$. In particular, the variation of $s$ with the system density is found to be strongly correlated with $R_{\text {n}}$ [2350]. In the case of a large value of $s$ for large system densities, the non-negligible fraction of protons in the system would stimulate Urca processes and hence cool the neutron star more rapidly.

An alternative method to determine the matter radius was proposed in [2354], in which antiproton collisions with nuclei would be exploited. There the extraction of the matter radius is achieved by measuring the antiproton-neutron annihilation cross section, but it is found to be rather model dependent. The produced *A* $-$1 nucleus after the $\bar{\mathrm {p}}$ annihilation can be detected by exploiting the Schottky technique [2355] in a storage ring. Such an experimental method has been proposed as a part of the FAIR project [2356], where antiprotons of about $\mathrm {500\, Mev}$ can be stored and then collided with nuclei. The intact *A* $-$1 nuclei can further circulate in the storage ring and be detected via the Schottky technique. This method would be an interesting alternative to the parity violating measurements with electron beams.

### Nucleon–nucleon interactions and finite nuclei from QCD

As issues of the nuclear equation of state will be dealt with later, this subsection concentrates on finite nuclei and nucleon–nucleon interactions. Since electromagnetic and weak effects are typically rather small for light nuclei, the holy grail of theoretical nuclear physics is to understand nuclear phenomena in terms of QCD. This task is quite daunting because QCD is a difficult theory that must be treated using non-perturbative methods. However, lattice QCD has begun to emerge as a truly precision tool to deal with many non-perturbative problems in QCD. Unfortunately, nuclear phenomena are not yet in this class of problems. For a variety of technical reasons, problems in nuclear physics are particularly difficult to pursue on the lattice. While there has been significant recent effort in attacking nuclear problems using lattice methods, and insights into nuclear problems can be gleaned from the present day calculations, there remains some distance to go. Given this situation, there is an interest in seeing whether one can learn *something* about nuclear physics from QCD without solving the theory. One method which has been pursued over the decades is to use “QCD-motivated” models to attack problems in nuclear physics. This approach has one highly problematic feature—it is difficult or impossible to tell what parts of a result come from QCD and what parts from an *ad hoc* model. As such this approach will not be discussed here. An alternative way forward is to consider systematic expansions based on counting rules which encode basic features of QCD. Two approaches of this sort will be discussed here: an effective field theory approach which can, in principle, encode the underlying approximate chiral symmetry of QCD and the other is the large-$N_\mathrm{c}$ limit of QCD and the $1/N_\mathrm{c}$ expansion (where $N_\mathrm{c}$ is the number of colors). While both of these approaches are interesting they do have important limitations.

#### Lattice QCD and nuclear physics

As we have noted, nuclear physics problems are intrinsically difficult to compute on the lattice. There are numerous reasons for this. The natural energy scales in nuclear physics are much smaller than in hadronic physics, so that calculations need to be done with much higher accuracy than in hadronic physics to determine phenomenologically relevant results. For example, an energy measurement with an accuracy of 1 MeV is a 0.1 % measurement of the nucleon’s mass but a 50 % measurement of the deuteron’s binding energy. This means, for instance, that extrapolating to the physical point for the pion mass can have a particularly large effect. Moreover, since the systems are bigger than for single hadrons, finite volume effects can be significant unless large lattices are used. Furthermore, signal-to-noise problems are expected to be more severe for systems which require many propagators. Finally, these calculations are simply more involved than calculations for single hadrons since they involve a large number of contractions.

Despite these challenges, there are major efforts to study the nucleon–nucleon interaction and bound states from the lattice, and significant progress has been made. One of these is centered in the NPLQCD collaboration. Reference [2357] gives a good idea of the state of the art for this work. The goals of NPLQCD are to compute observables of relevance to nuclear physics directly from the lattice. The approach can be used to compute the binding energy of light nuclei directly. While this is intellectually straightforward, the problem is technically challenging. Since the most basic interaction in nuclear physics is between two nucleons, it is also of interest to extract information about the nucleon–nucleon interaction. The most natural observables associated with this interaction describe nucleon–nucleon scattering as well as properties of the deuteron bound state. Some scattering observables, such as phase shifts, can be obtained by the standard approach of relating scattering observables to the energy levels in a box [395, 396]. Another class of observables of interest are hypernuclei and hypernucleon–nucleon scattering. The method is equally suitable for the study of these. While the current state of the art does not yet allow for computations in the regime of physical pion masses, serious calculations of real interest are being done—for example, a computation of the nucleon–nucleon scattering lengths in a world of exact $\mathrm{SU}(3)$ flavor symmetry and the computation of the binding energies of light nuclei and hypernuclei [2357, 2358]. Moreover, there is a clear path forward for this line of research, and one might expect this approach in time to lead to results which are directly applicable to the physical world. The techniques that have been developed are interesting in part because they are applicable to problems where experiments are difficult. Reliable *a priori* calculations are critical for resolving such issues as whether a bound H-dibaryon exists [2357]. The principal open question with this approach is just how far it can be pushed in practice.

Another approach has been pushed by the HAL QCD collaboration. It is in many ways far more ambitious than the NPLQCD approach. However, the scope of its ambition pushes the approach to a more problematic premise. Unlike the NPLQCD approach, this does not strive to compute nuclear observables directly from the lattice calculations. Rather, the underlying philosophy is to attempt to extract a nucleon–nucleon interaction in the form of a non-local potential from QCD which is supposed to be usable in few-body and many-body calculations. A review of this approach can be found in [2359]. There is an important theoretical issue about the foundations of this approach. Namely, the extent to which the interaction so obtained is capable of accurately describing many-nucleon systems.

#### Effective field theory approach

The initial drive underlying this strand of research was to encode the underlying approximate chiral symmetry of QCD into nuclear calculations in a systematic way in much the same way that chiral perturbation theory is used in hadronic physics. However, in nuclear physics there are low-energy scales that are not the direct result of chiral symmetry; for example, the large scattering lengths in the nucleon–nucleon system which are not the direct result of chiral physics. Thus, underlying the approach is the idea that one can build both the result of chiral dynamics *and* the other light scales of nuclear physics into an effective field theory (EFT). The main challenge with this approach is the calculation of physical observables (such as nuclear binding energies) from the EFT since unlike in chiral perturbation theory, this EFT must be used in a non-perturbative context. A recent review of this approach can be found in [2360].

The principal new development in the last several years has been the development of a lattice based approach to calculations within chiral effective field theory [2360]. The approach requires novel numerical techniques which are quite different from those in lattice QCD. This has already been applied to nuclear systems as heavy as ${}^{12}C$ and has proved amenable to calculations of excited states, including the Hoyle state in ${}^{12}C$ [2361].

There are open issues in this field of both practical and theoretical significance. On the practical level, the principal issue is the extent to which this method can be pushed to describe heavier nuclei. The basic theoretical question concerns its status as an EFT reflecting the underlying chiral structure of QCD. While there is a power counting scheme at the level of the interaction, the non-perturbative nature of the calculations mixes the various powers. An interesting and important question is the extent to which one can estimate *a priori* the scale of the effects of neglected higher-order terms on the nuclear observables based on power counting principles.

#### Large $N_\mathrm{c}$ limit and the $1/N_\mathrm{c}$ expansion

The $1/N_\mathrm{c}$ expansion around the large-$N_\mathrm{c}$ limit of QCD has yielded qualitative insights and some semi-quantitative results in hadronic physics. It is natural to ask whether it will be useful in nuclear physics. It is probably true that its principal value is of theoretical rather than of phenomenological value for most problems in nuclear physics. The reason is that the nucleon–nucleon force is much larger in a larger $N_\mathrm{c}$ world than in the world of $N_\mathrm{c}=3$ and many of the delicate cancellations which occur in nuclear physics are spoiled. For example, nuclear matter is believed to be a crystal at large $N_\mathrm{c}$, while it is thought to be a Fermi liquid in the physical world. It is important to recognize these limitations since many models are justified, at least implicitly, only at large $N_\mathrm{c}$, where mean-field approaches become exact.

On the theoretical side, there is interest in understanding nuclear physics in QCD-like theories, even in domains which are rather far from the physical world. Large $N_\mathrm{c}$ gives access to such worlds. However, it is typically not possible to solve theories directly even given the simplifications due to the large-$N_\mathrm{c}$ limit. Recently, however, the total nucleon–nucleon total cross section in QCD at momenta far above the QCD scale was shown to be calculable [2362] and given by

7.3 $$\begin{aligned} \sigma ^\mathrm{total} = \frac{2 \pi \, \log ^2(N_\mathrm{c})}{m_{\pi }^2} \, . \end{aligned}$$

This result follows from the fact that the nucleon–nucleon interaction is strong in the large-$N_\mathrm{c}$ world and thus the cross section is fixed by the mass of the lightest particle in the theory which acts to fix that range at which this intrinsically large interaction becomes weak. Unfortunately, it is of little phenomenological relevance as the corrections are of relative order $1/\log (N_\mathrm{c})$; the predicted cross section is a factor of three to four larger than the phenomenological one at energies of a few GeV. There have also been recent results on nuclear matter which are valid in a world in which both the number of colors is large and the quark masses are well above the QCD scale [2363, 2364]. While the world in which we live is far from the combined large $N_\mathrm{c}$ and heavy quark limits, the study of such a world is interesting since this represents a system for which a QCD-like theory is tractable.

The principal open issue in the field is whether there are phenomenologically relevant predictions in nuclear physics which are obtainable in practice from large-$N_\mathrm{c}$ analysis.

### Dense matter: theory and astrophysical constraints

#### Ultra-dense QCD and color–flavor locking

We do not have much knowledge from rigorous first-principles calculations about the QCD phase diagram in the plane of temperature and baryon chemical potential. The region of cold and dense matter turns out to be especially challenging. In the extreme limit of infinite density the system becomes tractable because the average energies and momenta of the particles are large and asymptotic freedom allows us to use weak-coupling methods. This is analogous to the high-temperature regime discussed in Sect. 6. Since the quark-quark interaction is attractive in the anti-triplet channel, the standard Bardeen–Cooper–Schrieffer (BCS) argument for superconductivity then tells us that the quark Fermi surface is unstable with respect to the formation of a quark Cooper pair condensate (this is “color superconductivity”—for a review see [2365]).

If we consider 3-flavor quark matter,^25^ the ground state is the Color-Flavor Locked (CFL) state [2366], where the three flavors pair in a very symmetric way. The symmetry breaking pattern is

7.4 $$\begin{aligned}&\mathrm{SU}(3)_\mathrm{c} \times \mathrm{SU}(3)_L\times \mathrm{SU}(3)_R \times U(1)_B \nonumber \\&\quad \rightarrow \mathrm{SU}(3)_{c+L+R} \times \mathbb {Z}_2 \, , \end{aligned}$$

where $\mathrm{SU}(3)_\mathrm{c}$ is the color gauge group, $\mathrm{SU}(3)_L\times \mathrm{SU}(3)_R$ the chiral flavor group (which is an exact symmetry at ultra-high densities where the quark masses are negligible compared to the quark chemical potential, $m_u,m_d,m_\mathrm{s}\ll \mu$), and $U(1)_B$ is the symmetry associated with baryon number conservation. The unbroken symmetry is a global $\mathrm{SU}(3)$ of simultaneous rotations in color and flavor space, hence the name color–flavor locking. In particular, CFL breaks chiral symmetry by an order parameter in a manner very similar to that seen in the vacuum where the order parameter is a chiral quark–antiquark condensate. We conclude that chiral symmetry of QCD is spontaneously broken at low *and* high densities. The low-energy degrees of freedom in CFL quark matter are Goldstone modes: one exactly massless superfluid phonon and eight light pseudoscalar mesons analogous to the pions and kaons. The quarks are gapped by their Cooper pairing; so, the phenomenology of the CFL phase at low energies is dominated by the Goldstone bosons.

Because of the spontaneous breaking of the color gauge symmetry, the gluons in the CFL phase acquire a Meissner mass, just like the photon in an ordinary electronic superconductor. More precisely, seven of the gluons plus one combination of the eighth gluon and the photon become massive, while the orthogonal combination of the eighth gluon and the photon remains massless. In the gauge sector, the infrared physics of the CFL phase thus reduces to an Abelian theory.

The CFL phase has many interesting properties, some of which have been worked out and some of which should be determined in the future. The phenomenology of the CFL phase is relevant for compact stars; see the discussion below and in Sect. 7.3.4. Of course, the matter in a compact star is in a region of the QCD phase diagram that is far from being asymptotically dense. In fact, one can estimate that the perturbative weak-coupling calculation of the CFL energy gap is reliable only for $\mu \gtrsim 10^8\,\mathrm{MeV}$ [2367]. This corresponds to densities 15–16 orders of magnitude larger than those in the center of compact stars. It is thus important to ask what the ground state of dense quark matter at these much lower densities is. Finding the answer to this question is a major challenge, and the problem is currently unsolved. The difficulties of this problem and approaches that have been applied and may be applied in the future are explained in the next two subsections.

#### Moderately dense QCD

Phases of QCD at moderate densities can be studied from two different perspectives. Either “from below,” by investigating dense nuclear matter and extrapolating results to higher densities (see Sect. 7.1) or “from above,” starting from CFL and asking what are the next phases down in density. Here we shall take the latter approach. As we reduce the density, we encounter two complications. First, we leave the safe grounds of asymptotic freedom and have to deal with a strongly coupled theory. Currently, there are no reliable methods in QCD to apply to this problem, and we have to rely on the alternative approaches discussed below. Second, the particularly symmetric CFL state will be disrupted because at the densities of interest the strange quark mass can no longer be neglected because its density-dependent value, which lies between the current mass ${\sim } 100\,\mathrm{MeV}$ and the vacuum constituent mass ${\sim } 500\,\mathrm{MeV}$, is not small compared to quark chemical potentials of the order of $(400$–$500)\,\mathrm{MeV}$ inside a compact star. Thus the Fermi momenta of up, down, and strange quarks are no longer equal: it is energetically more costly now to have strange quarks in the system, and hence the strange quark Fermi momentum becomes smaller. In the standard BCS pairing, however, it is crucial that the Fermi momenta of the quarks that form Cooper pairs are identical. Since CFL pairing relies on the attractiveness of the pairing between quarks of different flavors, this Fermi momentum mismatch imposes a kind of stress on the pairing. A simplified version of this problem was already discussed in the context of electronic superconductivity by Clogston and Chandrasekhar in the 1960s [2368, 2369]. In this case, the superconducting state becomes disfavored with respect to the unpaired state when $\delta \mu > \Delta /\sqrt{2}$, where $\delta \mu$ is the difference in chemical potential of the two fermion species that form Cooper pairs and $\Delta$ the quasi-particle energy gap. In quark matter, $\delta \mu$ is determined by $m_\mathrm{s}^2/\mu$. However, the situation in a compact star is more complicated than in an electronic superconductor because we are dealing not with 2 (spin up and down) but with $2N_\mathrm{f} N_\mathrm{c}=18$ fermion species (antiparticles can be neglected since they are strongly blocked in the presence of the Fermi sea) and because the conditions of electric and color neutrality impose constraints on the system. Nevertheless, the general expectation that it becomes “harder” for the quarks to form Cooper pairs in the presence of a non-negligible strange quark mass remains true.

The most radical possibility for the system to respond to the stress would be not to form any Cooper pairs. There are other options, however, which constitute viable candidates for matter in the core of compact stars. First of all, CFL may survive in a modified version, by producing a $K^0$ condensate (relieving the stress by producing negative strangeness), where the $K^0$ is the lightest of the (pseudo-)Goldstone modes of the chiral symmetry breaking in CFL [2370, 2371]. The resulting phase, usually called CFL-$K^0$, has interesting phenomenological properties that are being worked out in a series of studies, see for instance [2372–2374]. In a way, CFL-$K^0$ is the “mildest” modification of the CFL phase. Larger values of the strange quark mass (more precisely of $m_\mathrm{s}^2/\mu$ compared to the energy gap $\Delta$) lead to more radical modifications and eventually to a breakdown of CFL. Continuing our journey down in density (at zero temperature) we next expect the Cooper pairs to break in certain directions in momentum space, spontaneously breaking rotational symmetry. In general, such a phase can be thought of as a compromise between the fully paired and fully unpaired phases: the energy cost of forming Cooper pairs with zero total momentum in all directions becomes too large, but it is still preferable to form Cooper pairs in certain directions, if the kinetic energy cost is sufficiently small. Counter-propagating currents arise, of the kaon condensate on the one hand and the unpaired fermions on the other hand; hence, this phase is termed curCFL-$K^0$ [2375–2377]. With even larger mismatches, counter-propagating currents appear in more than one direction; as a result the system spontaneously breaks translational invariance and crystalline structures become possible, where the gap $\Delta$ varies periodically in space and vanishes along certain surfaces [2378–2380]. Further increasing $m_\mathrm{s}^2/\mu$, the CFL pairing pattern may break down, and pairing only between up and down quarks (“2SC phase”) [2381] or single-flavor pairing in the spin-one channel [2382, 2383] become candidates for the ground state.

This journey down in density has been done by varying the “parameter” $m_\mathrm{s}^2/\mu$ and by relying on effective theories, phenomenological models, etc., but not on first-principles QCD calculations. In QCD, the only dimensionful parameter is $\mu$, while $m_\mathrm{s}$, $\Delta$, and the strong coupling constant are functions of $\mu$ that are unknown in the strongly coupled regime. In other words, it is currently not known how the above sequence of phases translates into the QCD phase diagram. It is conceivable that the CFL phase (or variations of it) persists down to densities where the hadronic phase takes over. In this case, the intriguing possibility of a quark-hadron crossover might be realized [2384–2386]. Or, there may be one or several of the above more exotic color superconductors between the CFL phase and the hadronic phase. It is fair to say that a major improvement of current theoretical tools is needed to settle these questions unambiguously. We shall discuss some of the tools used so far and promising theoretical directions for the future in the next subsection. A complementary line of research is provided by astrophysics, where candidate phases can be potentially ruled out from properties of compact stars, see Sect. 7.3.4.

#### Theoretical approaches and challenges

Let us discuss the theoretical tools available for studying dense QCD matter and their potential and perspective for future research. We start with the ones that have already been employed extensively to obtain the above sketched picture of the QCD phase structure and then turn to more novel approaches.

*a. Perturbative QCD* We have already mentioned the regime of applicability of perturbative QCD, which is limited to densities many orders of magnitude larger than the densities in the interior of compact stars. Although distant from the physically relevant regime, this is a secure base from which we extrapolate down in density just as we use results from nuclear physics to extrapolate up in density. An extrapolation over many orders of magnitude seems bold, but the value it gives for the energy gap of color superconductivity is comparable to the result obtained from a phenomenological Nambu–Jona-Lasinio (NJL) model whose parameters are fit to *low*-density properties. The extrapolation of perturbation theory down to $\mu \simeq 400\,\mathrm{MeV}$ yields $\Delta \simeq 20\,\mathrm{MeV}$ [2387, 2388] (using a strong coupling constant $g\simeq 3.5$, suggested by the two-loop QCD beta function), while NJL calculations suggest $\Delta \simeq$ (20–100) MeV. Given the completely different theoretical origins of these results, their approximate agreement is remarkable.

In perturbative calculations, the magnitude of the zero-temperature energy gap $\Delta$ translates into a critical temperature $T_\mathrm{c}$ for color-superconductivity via a BCS-like relation. In BCS theory, $T_\mathrm{c} = (e^\gamma /\pi )\,\Delta \simeq 0.57\,\Delta$, where $\gamma$ is the Euler–Mascheroni constant. In some color superconductors this relation is modified [2389], in the CFL phase $T_\mathrm{c} = 2^{1/3}(e^\gamma /\pi )\,\Delta$, but it is still true that the critical temperature is given by a numerical factor of order one times the zero-temperature gap. Therefore, one consequence of the above estimate of $\Delta$ is that the critical temperature of the CFL phase (and of other spin-zero color superconductors) is larger than the typical temperature of a neutron star.

*b. Effective theory of CFL* One can construct an effective Lagrangian for the low-energy degrees of freedom of CFL [2371], like the chiral Lagrangian for low-density mesons. This effective theory does not tell us if and at what density CFL is replaced by another phase, but it can be used to compute properties of CFL and CFL-$K^0$ in terms of a small number of unknown couplings in the Lagrangian. This has been done for transport properties such as bulk and shear viscosities [2373, 2390–2392]. Since its form is determined by the symmetries of CFL, the effective theory must be valid for all densities where CFL, or any other phase with the same symmetry breaking pattern, exists (at energies far below the critical temperature of CFL). Hence, if CFL persists down to densities of astrophysical interest we can determine, at least qualitatively, the properties of matter at these densities. Quantitative predictions are still subject to uncertainties since up to now the only way we can estimate the parameters of the Lagrangian is by matching to perturbative high-density results.

*c. Hydrodynamics* Efforts to connect neutron star observables with the properties of their interior often involve calculating transport properties that characterize the hydrodynamics of cold dense QCD matter. (The hydrodynamics of hot QCD matter is an active research field with relevance for heavy-ion collisions, see Sect. 6, and it will be interesting to see whether and how these two research lines can benefit from each other). In a neutron star, hydrodynamics becomes important for instance in the discussion of $r$-mode instability^26^ [2393], asteroseismology [2394], discussed below, and dynamical effects of the magnetic field [2395]. In particular, it is desirable to understand *superfluid* hydrodynamics since superfluidity appears in nuclear matter as well as in quark matter. In quark matter, only the CFL phase (and its variants) is superfluid, because of the spontaneous breaking of baryon number conservation, see (7.4). Superfluidity of the CFL phase manifests itself-for instance in the presence of three different bulk viscosity parameters [2392].

For applications to neutron stars one must deal with the fact that in some cases the mean free path of the superfluid phonons can be comparable to or even larger than the size of the star, as discussed in [2396, 2397] with emphasis on applications in cold atomic trapped gases.

It is also valuable to formulate the hydrodynamics of CFL in the hydrodynamical framework that is used by astrophysicists. A first step in this direction has been made recently in connecting the relativistic two-fluid formalism of superfluidity with microscopic physics [2398, 2399]. Like proton-neutron matter, CFL may also be a complicated multi-fluid system if kaons condense, i.e., in the CFL-$K^0$ phase. In this case it is not only $U(1)_B$ that is spontaneously broken (by the Cooper pair condensate), but also strangeness conservation (by the kaon condensate). Interesting fundamental questions regarding superfluidity arise because strangeness is not conserved when the weak interactions are taken into account, i.e., one has to understand whether some superfluid phenomena can persist even if the underlying $U(1)$ is only an approximate symmetry.

*d. Nambu–Jona-Lasinio (NJL) model* In the NJL model, the gluonic interaction between the quarks is replaced by a simple four-fermion interaction. Because of its relative simplicity, and because it is well suited to incorporate Cooper pairing (it was developed originally in this context) as well as the chiral condensate, it has been frequently used to gain some insight into the phase structure of dense quark matter; see for instance [2400–2403], and, for extensions including the Polyakov loop, Refs. [2404–2407]. These studies are very useful since they point out possible phases and phase transitions. However, they are ultimately of limited predictive power because their results depend strongly (even qualitatively) on the chosen values of the parameters such as the coupling constants and because the model is not a controlled limit of QCD.

*e. Ginzburg–Landau (GL) studies* A GL theory is an effective theory of the order parameter. It is like the low-energy effective theory described above, except that it includes the “radial” degree of freedom, which corresponds to the magnitude of the order parameter, hence it can describe the transition at which the order parameter becomes non-zero. Whereas GL theory is valid in the vicinity of the second-order thermal (“melting”) phase transition where the correlation length diverges, the aforementioned effective theory of CFL is valid away from this transition line so that these two approaches nicely complement each other. GL theories are commonly used to describe phase transitions in condensed matter physics, and, along with NJL models, have also been applied to phase transitions in dense quark matter. A GL theory has been used to show that gauge field fluctuations yield a correction to the critical temperature of color superconductivity and render this phase transition first order [2408]. Also, the significance of the axial anomaly for a possible crossover between nuclear and CFL quark matter has been investigated within GL theory [2385]. As with NJL models, the GL theory can be very useful as a guideline for the phases of dense QCD, especially since ordinary condensed matter physics tells us that the phase diagram can be expected to be very rich; however, if we are interested in full QCD, it can at best be a first step towards more elaborate studies.

We now discuss some theoretical approaches which have only recently been considered for the study of dense matter and which may shed light on the open problems from different angles, but which all have to deal with difficult theoretical challenges.

*f. Lattice QCD* Lattice gauge theory is currently the most powerful method to determine equilibrium properties of the QCD vacuum and its excitations (see Sect. 6). However, as explained in that chapter, at finite density the usual probabilistic sampling method fails because of the sign problem. Therefore, there is currently no input from lattice QCD to the questions we discuss here. Several groups are trying to find ways around the sign problem [2409–2411]. For instance, it has been shown that in a combined strong coupling and hopping expansion, an effective theory can be derived for which the sign problem is relatively harmless [2410]. Currently this method is restricted to unphysically large quark masses. In this limit, first indications for a nuclear matter onset have been obtained [2412]. It will be very interesting to see whether this approach can be extended to more realistic quark masses, and whether it can eventually tell us something about realistic, dense nuclear and quark matter from first principles. For instance, one might try to study quark and nucleon Cooper pairing and its phenomenological consequences.

*g. Large-* $N_\mathrm{c}$ QCD The number of colors $N_\mathrm{c}$ is a useful “knob” that, if set to a sufficiently large value, deforms QCD into a simpler (albeit not simple) theory; see for instance Sects. 7.2 and 9. For the study of moderately dense QCD, the $N_\mathrm{c}\rightarrow \infty$ limit is, like the asymptotic $\mu \rightarrow \infty$ limit discussed above, a more accessible regime from which we can extrapolate (admittedly with the chance of missing important physics) to the regime of interest. The difference is that for $N_\mathrm{c}\rightarrow \infty$ we leave the theory of interest, while for $\mu \rightarrow \infty$ we stay within QCD. The gross features of the large-$N_\mathrm{c}$ QCD phase diagram are known, and it has been argued that nuclear matter at large $N_\mathrm{c}$ (called “quarkyonic matter”) behaves quite differently from $N_\mathrm{c}=3$ nuclear matter [2413]. It is an interesting, unsolved question whether quarkyonic matter survives for $N_\mathrm{c}=3$ QCD, and several studies have addressed this question, for instance, within NJL-like models [2407]. It is also known that for very large $N_\mathrm{c}$ quark-hole pairing is favored over quark-quark pairing and thus the CFL phase is replaced by a so-called chiral density wave [2414]. These results seem to indicate that, at least for dense matter, $N_\mathrm{c}=3$ is very different from $N_\mathrm{c}=\infty$.

*h. Gauge/gravity correspondence* The gauge/gravity correspondence has become an extremely popular tool to study strong-coupling physics. It has relevance to heavy-ion physics (see Sect. 6) and to dense matter (see Sect. 9). One can introduce a chemical potential in a gauge/gravity calculation, and this provides a tractable system of dense matter with strongly coupled interactions. The model that currently comes closest to QCD is the Sakai–Sugimoto model [2415], which completely breaks supersymmetry and contains confinement/deconfinement and chiral phase transitions. In the context of dense matter, it has been used to compute phase structures in the presence of finite $\mu$ and $T$ [2416, 2417] and in a background magnetic field [2418, 2419]. For nuclear matter, however, its relevance for QCD is questionable, since it has been shown that holographic nuclear matter behaves quite differently from ordinary nuclear matter [2420, 2421]; in particular, the nuclear matter onset in the Sakai–Sugimoto model is second order, indicating the absence of a binding energy. One of the reasons for this and other differences to QCD is the large-$N_\mathrm{c}/N_\mathrm{f}$ limit to which most of these studies are constrained. Their relevance for dense QCD is thus debatable for reasons discussed in the previous paragraph. It would be very interesting, but also very challenging, to lift the constraint of large $N_\mathrm{c}/N_\mathrm{f}$ in these holographic studies; see [2422, 2423] for pioneering work in this direction. It would also be interesting to study color superconductivity in gauge/gravity duality. First steps in this direction within a “bottom-up” approach have been done [2424], resulting in phase diagrams that resemble qualitatively the expected phase structures of dense QCD.

#### Dense matter and observations of compact stars

Matter at densities of several times nuclear ground state density is very difficult to study experimentally (see Sect. 7.1). Dedicated collider experiments in the coming years at FAIR (Darmstadt) and NICA [2425] (Dubna) will help to extend our experimental reach further into the region of high densities, although it will remain a challenge to produce dense matter that is cold enough to exist in the deconfined quark phases discussed above. We therefore turn our attention to compact stars, which are the only place in the universe where we might find cold nuclear or even quark matter. After black holes, compact stars are the densest objects in nature. They are the remains of massive ordinary stars after the nuclear fusion process runs out of fuel, and the gravitational attraction in the collapsing core can only be compensated by the Pauli pressure of the strongly interacting constituents. They have masses of more than a solar mass at radii of the order of 10 km and can thereby reach up to 10 times the density reached in atomic nuclei, corresponding to a baryon chemical potential up to 1.5 GeV. These densities are large enough that they could contain phases of dense quark matter in their interior, but are far below the asymptotic densities described above.

To learn something about dense phases of QCD from astrophysical observations, we need to compute properties of candidate phases and see whether the astronomical observables are able to discriminate between these candidate phases. This would allow us to put constraints on the structure of the QCD phase diagram.^27^ For instance, we would like to understand whether compact stars are made of nuclear matter only (neutron stars), whether they contain a quark matter core with a nuclear mantle (hybrid star), or whether they are pure quark stars. Here we will limit ourselves to discussion of a few very interesting recent measurements which nicely demonstrate how we can obtain constraints on dense matter from compact stars and what is needed in the future to make these constraints more stringent. For a broader pedagogical review see for instance [2428].

**Fig. 83:**
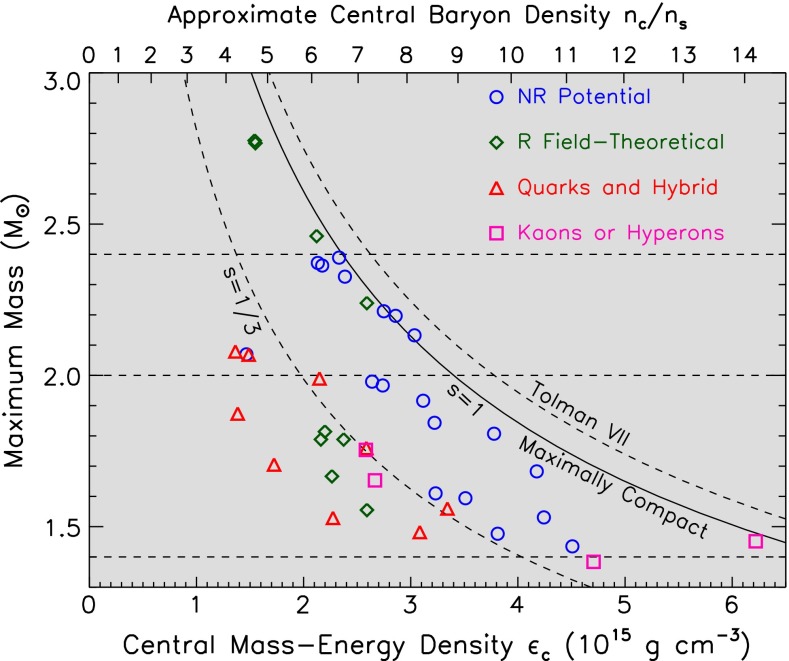
A given compact star mass (*vertical axis*) implies an upper bound for the energy density (*lower horizontal scale*) and the baryon density (*upper horizontal scale*) in the center of the star. For instance, $M\simeq 2\,M_\odot$ (see *middle horizontal dashed line*) allows a central baryon density of no more than about 9 times nuclear ground state density. An even heavier star would *decrease* this upper bound. The solid line that gives this bound is obtained by assuming a “maximally compact” equation of state of the form $P=s(\epsilon - \epsilon _0)$ with $s=1$. Independent of $\epsilon _0$ one finds $\epsilon _\mathrm{max}M_\mathrm{max}^2=1.358\times 10^{16}\,\mathrm{g}\,\mathrm{cm}^{-3}M_\odot ^2$, which defines the *solid line.* The various points are calculations within different models and matter compositions. They confirm the limit set by the *solid curve* and show that equations of state with pure nuclear matter tend to give larger maximal masses than more exotic equations of state. Details can be found in [2436], where this figure is taken from

*a. Mass-radius relation and the* $2\,M_\odot$ *compact star* The mass-radius function $M(R)$ of a compact star is determined, via the Tolman–Oppenheimer-Volkov equation, by the EOS of the dense matter of which it is made. Therefore measurements of masses and radii provide information about the EOS of nuclear and perhaps quark matter. The $M(R)$ curve has a maximum mass which is larger if the EOS is stiffer, i.e., has stronger repulsive interactions, and smaller if the EOS is soft. In order to use measurements of $M(R)$ to learn about dense matter, we need to calculate the EOS for the various different forms of matter that we think might be present. Such calculations are not well controlled, particularly at densities above nuclear density, and the results have large uncertainties. However, in general, one can say that matter with larger number of degrees of freedom tends to be softer and yield smaller maximal masses, or, more precisely, if one adds new degrees of freedom to the system, the interactions must become stronger in order to achieve the same maximal mass. Model calculations confirm that hyperons and/or meson condensates in nuclear matter decrease the maximum mass of neutron stars, see for instance Refs. [2429, 2430]. Also quark matter has more degrees of freedom than ordinary nuclear matter, suggesting a softer equation of state. However, it is not known whether this effect can be compensated by the strength of the interactions.

The heaviest neutron stars observed to date are the pulsars PSR J1614-2230 and PSR J0348+0432, which have been determined to have masses $M=(1.97\pm 0.04)M_\odot$ [2431] and $M=(2.01\pm 0.04)M_\odot$ [2432], respectively. Both results are remarkably precise (achieved by measuring Shapiro delay in a nearly edge-on binary system in the first case, and by a precise determination of the white dwarf companion mass in the second case).

The large value of the mass rules out several proposed EOS for dense matter [2433, 2434] and strongly constrains the quark matter EOS [2435].

In this area we look forward to both theoretical and observational improvements. Future observations may yield even heavier stars, and more accurate measurements of radius along with mass, giving a more accurate idea of the $M(R)$ curve for compact stars. In Fig. 83 we show some theoretical results for various equations of state for neutron stars, hybrid stars, and quark stars together with a general constraint for the maximal density in the center of the star that can be obtained from a given mass measurement [2436]. It is important for theorists to improve our understanding of cold, dense, strongly interacting quark matter, for instance with better perturbative calculations, such as in [2437, 2438] where the equation of state up to order $\alpha _\mathrm{s}^2$ has been worked out, or non-perturbative studies building on a Dyson–Schwinger approach [2439–2441].

*b. Cooling rate and the fast cooling of Cas A* While different phases of matter may have very similar equations of state, which is a bulk property, they may be distinguished by their neutrino emissivity, which is more sensitive to the low-energy excitations. Since neutrino emission is the dominant cooling mechanism of a neutron star less than a million years old, measurements of cooling give information about neutrino emissivity and hence about the phases present inside the star, in particular about superfluidity. Unpaired matter can more easily produce neutrinos and antineutrinos via beta decay: its emissivity varies as some power of temperature. In contrast, superfluid matter with an energy gap $\Delta$ in the quasi-particle spectrum shows an exponential suppression of the emissivity $\propto e^{-\Delta /T}$ for small temperatures $T\ll \Delta$ [2442–2444]. However, the emissivity of a superfluid can be enhanced—even compared to unpaired matter—at temperatures below, but close to, the critical temperature due to continual pair breaking and formation (PBF) of the Cooper pairs [2445–2447].

There has been a noteworthy recent observation of the isolated neutron star in the Cassiopeia A (Cas A) supernova remnant [2448]. It is the youngest known neutron star of the Milky Way with an age of 330 yr.

Recent analysis shows some evidence that the temperature of this star has decreased during 2000 to 2009, by $3\,\% \,\pm \, 1\,\% \,\pm \, 0.5\,\%$ [2448]. If this turns out to be a physically real effect, such fast cooling would imply a high neutrino emissivity during that time period.

It has been conjectured that the PBF process mentioned above might be responsible for the rapid cooling of the Cas A star [2449, 2450], in the following way. Before the rapid cooling began, the core of the star contained superconducting protons (ensuring that it cooled slowly) and unpaired neutrons. When the temperature in the core reached the critical temperature $T_\mathrm{c}$ for neutron superfluidity in the $^3P_2$ channel, the PBF process began to occur in that region, accelerating the cooling process. It is therefore conjectured that in Cas A we are observing the superfluid transition of neutrons in real time. This explanation assumes, as theorists have predicted [2451, 2452], that the critical temperature is strongly density dependent.

**Fig. 84:**
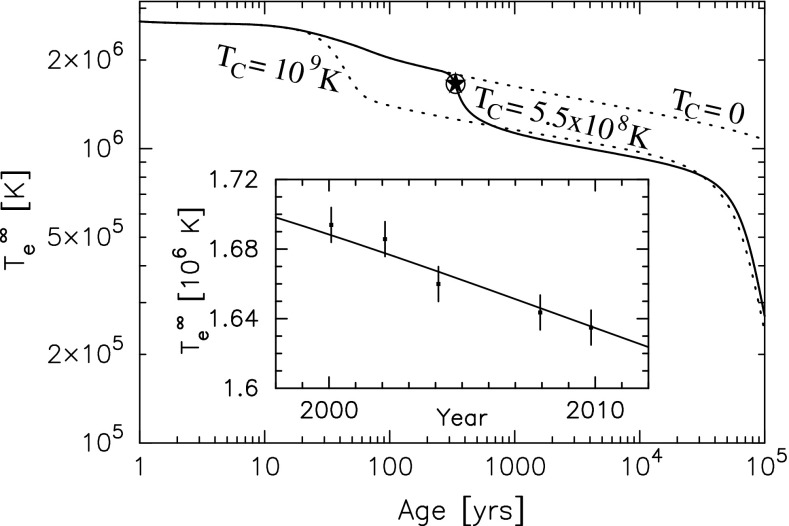
Red-shifted effective temperature versus age of the Cas A neutron star: data (*encircled star* and points with *error bars* in the zoom-in) and theoretical curves based on the PBF process for various critical temperatures $T_\mathrm{c}$ for neutron superfluidity (more precisely the maximal $T_\mathrm{c}$, since $T_\mathrm{c}$ depends on density). The *solid line*, also shown in the zoom-in matches the data points, while larger or smaller values for $T_\mathrm{c}$ would lead to an earlier or later start of the rapid cooling period. Figure taken from [2449]

There is then for an extended time period a slowly expanding shell in the core at which the temperature is close to $T_\mathrm{c}$ and where the efficient PBF cooling mechanism operates. In Fig. 84 we show how this can explain the data [2449].^28^

Although several assumptions go into this interpretation, it is a nice example how an astrophysical observation can yield constraints on microscopic parameters such as the critical temperature for neutron superfluidity. This “measurement of $T_\mathrm{c}$” becomes particularly interesting because of the enormous uncertainties in the theoretical calculation of $T_\mathrm{c}$ from nuclear physics [2453–2455].

Alternative scenarios have been discussed in the literature as well, and it is an interesting problem for future studies to either support or rule out the various possible explanations. For instance, taking into account certain medium corrections to the cooling process appears to explain the data without any transition into the superfluid phase, i.e., with a much smaller critical temperature for neutron superfluidity [2456, 2457]. It has also been suggested that a color-superconducting quark matter core may explain the cooling data [2458, 2459]. In the scenario of [2459], the rapid cooling is due to a transition from the so-called 2SC phase—plus a phenomenologically imposed gap for the blue quarks that usually remain unpaired in 2SC—to a crystalline color superconductor where there are unpaired fermions that enable efficient neutrino emission.

*c. Gravitational wave emission and compact star seismology* Another way to directly probe the interior composition of compact stars is to study how the dense matter inside damps global oscillation modes, using seismological methods similar to those employed to learn about the interior composition of the earth or the sun. Particularly relevant are “$r$-modes” [2393, 2460], which in the absence of damping are unstable and grow spontaneously until they reach their saturation amplitude. They then cause the star to spin down by emitting gravitational waves. The observable consequences are (a) direct detection of the waves [2461, 2462] in next-generation detectors such as advanced LIGO [2463] and VIRGO [2464] or the planned Einstein telescope; (b) stars should not be found in the “instability region” in spin-temperature space. Mapping the instability region would tell us about the interior viscosity, because it is the viscosity that limits the occurrence of $r$-modes to an instability region over a range of temperatures and sufficiently large frequencies. How quickly stars exit from the instability region is determined by the saturation amplitude of the $r$-modes, which is restricted by astrophysical observations [2465, 2466]. Several possible saturation mechanisms have been proposed [2467–2472]. Although the actual mechanism and amplitude remains uncertain, no currently proposed mechanism yields amplitudes that are low enough that a fast-spinning star would not spin down too fast to be consistent with the stringent astrophysical bounds. Thus no pulsars are expected to be found within the $r$-mode instability region in frequency-temperature space.

The current state of observations is that there are many millisecond pulsars in low mass X-ray binaries (LMXBs) which have been spun up by accretion from a companion star and now lie well within the instability region that is predicted for a standard neutron star without enhanced damping mechanisms such as crust-core rubbing [2473]. This conclusion is based on accurate measurements of their frequencies and reasonable estimates of their temperatures from a spectral fit to their quiescent X-ray radiation. This means there must be additional damping mechanisms that suppress the $r$-modes, such as structural aspects in the star’s crust or the presence in the star of novel phases such as a superfluid and/or superconductor, or quark or hyperonic matter [2474]. Semi-analytic expressions have now been derived that make it possible to estimate the many uncertainties in our predictions of the instability region, and it has been shown that many of the relevant macroscopic observables are remarkably insensitive to quantitative microphysical details, but can nevertheless distinguish between qualitatively different forms of dense matter [2475]. A promising recent development is that by a detailed understanding of the pulsar evolution the r-mode instability could be connected to timing data of radio pulsars [2461] using novel dynamic instability regions [2466] that confirmed the above picture. The extensive data for these old and very stable sources is among the most precise data in physics [2476], which could allow a clear distinction between different forms of dense matter. Finally, even if the saturation amplitude is very low so that $r$-modes would not affect the spindown evolution, they would still strongly heat the star [2466] so that this scenario could be falsified by future X-ray observations.

*d. X-ray bursts and the physics of the neutron star crust* Nuclear matter at lower densities is found in the crust of neutron stars, so surface phenomena that are sensitive to the behavior of the crust give information about this form of matter. A better understanding of the crust is also valuable for understanding the core, since measurements of the temperature of the star’s core are based on surface phenomena (quiescent X-ray spectra) combined with models of heat transport through the crust.

Observations of X-ray bursts are a valuable source of information about the crust. The bursts arise from light elements, which are accreted onto the neutron star surface, then gradually sink down and at a critical pressure and density are explosively converted into the heavier nuclei that form the star’s crust [2477]. Detailed observations of the resultant X-ray emission and subsequent cooling can then, in principle, be used to constrain the parameters of theoretical models of the crust. This requires calculations of the expected behavior using those models, including detailed dynamical understanding of the various transport properties within the crust. Recent progress in this direction includes the development of effective theories for the crust [2478], which describe the dynamics of the low energy degrees of freedom given by electrons, lattice phonons and (in the inner crust) Goldstone bosons arising from neutron superfluidity.

It is worth noting that understanding the nuclear fusion and capture reactions underlying X-ray bursts is also relevant for understanding the nucleosynthesis of heavy elements.

### Future directions

Throughout the chapter we have pointed out various important future directions for nuclear physics and dense (nuclear and quark) matter with applications to astrophysics. As far as the kaon–nucleon interaction is concerned new results of the $K$- and $\bar{K}$-nucleons interaction at $\rho _0$ are going to be delivered by the GSI and JPARC experiments, together with high-statistics and high-precision measurement of all the collective observables from heavy-ion collisions at 10–30 AGeV with CBM at FAIR which will test higher densities. Further experiments will be carried out mainly at JPARC to search for kaonic bound states and the planned precision measurement of kaonic–deuterium with SIDDHARTA in FRASCATI and at JPARC will deliver conclusive information on the isospin dependence of the kaon–nucleon scattering length. All these results together should deliver solid density dependent constraints for models that hypothesize the presence of antikaons in the inner-core of neutron stars. On the other hand, we have pointed out the importance of hyperons in understudying dense and compact objects. New measurements of single and double-$\Lambda$ hypernuclei at the upcoming JPARC facility and more data on hyperon production in hadron-hadron collisions are expected from HADES and the future FAIR experiments. As a complement to these measurements, a more precise determination of the matter radius of neutron-rich nuclei at JLab and MAMI are planned. These measurements impose important constraints on the thickness of the neutron crust in neutron stars and also on the boundaries of possible phase transition when going to the inner part of the star.

Possible ways towards a better theoretical understanding of dense matter include solving or mitigating the sign problem in lattice QCD as well as combining established theories (perturbative QCD, effective theory of CFL, hydrodynamics) or models (NJL, NJL with Polyakov loop, sigma models including both quarks and hadrons, etc) with more novel approaches (gauge/gravity correspondence). A major future direction for the field of theoretical nuclear physics is the continued push to obtain ab initio calculations of nuclear properties from QCD via lattice methods. In the near future, we can expect more, and more precise, astrophysical data from compact stars (mass, radius, temperature, X-ray bursts, possibly gravitational waves) which should be compared with predictions from QCD or effective theories/models (equation of state, transport properties).

## Vacuum structure and infrared QCD: confinement and chiral symmetry breaking

29The Standard Model of particle physics is formulated as a quantum theory of gauge fields, describing weak and electromagnetic interactions by electroweak theory and strong interactions by Quantum Chromodynamics (QCD). Quantum field theories have a very well-developed perturbation theory for weak couplings. Processes of elementary particles at high energies are characterized by asymptotic freedom, by decreasing strength of the strong interaction with increasing energy. This makes QCD a valuable tool to investigate the strong interaction: In the weak coupling regime of QCD the agreement of perturbative calculations with an enormous number of available measurements is truly impressive. This is the case despite the fact that QCD violates an essential basis of a perturbative description, namely field-particle duality. This duality assumes that each field in a quantum field theory is associated with a physical elementary particle.

It is evident that hadrons are not elementary particles. The partonic substructure of the nucleon has been determined to an enormous precision leaving no doubt that the parton picture emerges from quarks and gluons, the elementary fields of QCD. It is a well-known fact that these quarks and gluons have not been detected outside hadrons, which is known as confinement. Although this hypothesis was formulated decades ago, the understanding of the confinement mechanism(s) is still not satisfactory, see, e.g., [2479] for a recent discussion of the different aspects of the confinement problem.

Noting that the Kinoshita–Lee–Nauenberg theorem on infrared divergences [2480, 2481] applies to non-Abelian gauge theories in four dimensions, order by order in perturbation theory [2482], a description of confinement in terms of perturbation theory (at least in any naïve sense) is excluded. This finding corroborates the simple argument that, since confinement arises at small momentum scales, the relevant values of the strong coupling $\alpha _\mathrm{s}$ are too large to justify a perturbative treatment. Therefore non-perturbative methods are required to study the dynamics of confinement. Furthermore, the quest for the confining gluonic field configuration(s) has led to the anticipation that a possible picture of confinement is directly related to the vacuum structure of QCD.

In the first section of this chapter we will comment on our current understanding of the QCD vacuum as it is obtained from lattice gauge theory and its duality to string theory. In the second section we briefly review some aspects of confinement and dynamical breaking of chiral symmetry from the perspective of functional methods. In the third section, additional aspects of chiral symmetry breaking as inferred from lattice calculations are revisited.

### Confinement

Confinement is a fascinating phenomenon which precludes observation of free quarks in our world. Mathematically, the property of confinement is usually formulated in terms of the potential $V_{\bar{Q}Q}(R)$ between external heavy quarks. In the case of pure gluodynamics (i.e., without dynamical quarks) this potential grows at large distances $R$, thus not allowing the quarks to separate. Lattice simulations indicate that

8.1 $$\begin{aligned} \lim _{R\rightarrow \infty }{V_{\bar{Q}Q}(R)} = \sigma \cdot R+\frac{\text {const}}{R}, \end{aligned}$$

where $\sigma$ is a constant and $\text {const}/R$ is the leading correction. Equation (8.1) can be interpreted in terms of a string, of tension $\sigma$ stretched between the heavy quarks.

Despite many years of intense effort, there is no analytic solution yet to the problem of confinement in the case of non-Abelian gauge interactions in four dimensions, i.e., in the real world. There are examples, however, of Abelian theories where confinement is demonstrated analytically [2483–2485]. What is common to all these models is that confinement is associated with particular vacuum field configurations, or with the structure of the vacuum. Moreover, there is a strong correlation between confinement of some charges and condensation of the corresponding magnetic degrees of freedom [2483–2485]. For example, in the case of an Abelian charge, confinement of charged particles is due to the condensation of magnetic monopoles, and vice versa. An important example is provided by superconductors: it is a charged field which is condensed and (external, heavy) magnetic monopoles which are confined. Thus, observation of confinement probably indicates a kind of duality between electric and magnetic degrees of freedom. According to modern theoretical views, the vacuum of non-Abelian theories is populated by condensed, or percolating, magnetic degrees of freedom. By studying the vacuum structure we expect to observe the dual world of the magnetic degrees of freedom.

Studies using lattice gauge theory have produced strong support of this idea; for a review see, e.g., [2486]. The fact that one can observe and make measurements on vacuum fluctuations is far from trivial. Indeed, in the continuum-theory language one usually subtracts vacuum expectations of various operators, concentrating on the physical excitations. In this respect, the vacuum of the latticized space-time is rather similar to the “vacuum” of percolation theory.^30^ In the latter case the properties of the vacuum condensates, in the so-called overheated phase, are subject of theoretical predictions and measurements. For example, the phase transition is signaled by emergence of an infinite cluster of closed trajectories at some critical value $p_\text {cr}$ . At $p>p_\text {cr}, |p-p_\text {cr}|\ll 1$ the probability of a given link belonging to the infinite cluster is still small:

8.2 $$\begin{aligned} \theta _\text {inf.cluster} \sim (p-p_\text {cr})^{\alpha }, \end{aligned}$$

where $$\alpha > 0$$.

Lattice studies of the vacuum of the Yang–Mills theories revealed the existence of infinite clusters of trajectories and surfaces with remarkable scaling properties:

8.3 $$\begin{aligned}&\theta _\text {link} \approx (\text {const})(\Lambda _\text {QCD} a)^3, \nonumber \\&\theta _\text {plaquette} \approx (\Lambda _\text {QCD} a)^2~, \end{aligned}$$

where $\theta _\text {link}$ and $\theta _\text {plaquette}$ are the probabilities of a given link and plaquette, respectively, to belong to the infinite clusters; $a$ is the lattice spacing; and $\Lambda _\text {QCD}$ is the hadronic scale, $\Lambda _\text {QCD}\sim 100~\mathrm{MeV}$. Moreover, the trajectories are contained in the surfaces [2488–2490]. These lines and surfaces can be called defects of lower dimension. Indeed, the trajectories represent $D=1$ defects in the Euclidean $D=4$ space and surfaces represent $D=2$ defects. Removal of the defects, which occupy a vanishing fraction of the lattice in the continuum limit $a\rightarrow 0$ results in the loss of confinement (and of the spontaneous breaking of chiral symmetry). In terms of the non-Abelian fields, the defects are associated with an excess of action and topological charge.

A theory of confinement in terms of field-theoretic defects is an unfinished chapter. Although truly remarkable observations were made in lattice studies and illuminating theoretical insights were suggested there is no concise picture yet. Elaborating such a picture would be of great importance for the field theory in general. It resembles going from the Hooke’s law for continuum media to a theory of dislocations where the same law arises only after averaging over many defects. Whether a similar step can indeed be made in the case of confinement remains an open question, to be addressed in the future.

So far we have discussed temperature $T=0$ and Euclidean space-time. Thus, the defects percolate in all four dimensions. A remarkable phenomenon occurs at the temperature of the deconfining phase transition, $T_\mathrm{c}$: the defects become predominantly parallel to the time direction, while still percolating in three spatial dimensions, see, in particular [2486, 2491]. On the lattice, one studies geometrically defined asymmetries such as

8.4 $$\begin{aligned} A = \frac{N_{\tau }-\frac{1}{3}N_{x,y,z}}{N_{\tau }+\frac{1}{3}N_{x,y,z}}, \end{aligned}$$

where $N_{\tau }$ is the number of links (belonging to the 1D defects above) looking in the Euclidean time direction and $N_{x,y,z}$ is the number of links looking in one of the spatial directions. The asymmetry $A\approx 0$ below $T_\mathrm{c}$ and $A\approx 1$ at temperatures above $T_\mathrm{c}$.

It is worth mentioning that lower-dimensional defects in field theories have been discussed in many papers. One of the best known and early examples is [2492]. Moreover, the quantized vortices in rotating superfluids, known for about 70 years, can be thought of as 1D defects. Indeed, within the hydrodynamic approximation vortices introduce a singular flow of the liquid, with the singularity occupying a line, the axis of the vortex.

However, in the particular case of Yang–Mills (YM) theories, the only example of non-perturbative fluctuations which can be studied in the quasi-classical approximation is provided by instantons, and this example does not help to interpret the lattice data mentioned above. Probably, this is one of the reasons why the observations (see (8.3)) and their extensions did not have much feedback to the continuum theory. Also, the algorithm for the search of defects is formulated in a specific lattice language, and this makes the interpretation of data difficult. Actually, in the case of temperature $T>T_\mathrm{c}$ a well-known example of a field-theoretic operator exists, which might serve as a field theoretic image of the (Euclidean) time-independent defects. We have in mind the Polyakov line, or path-ordered exponent:

8.5 $$\begin{aligned} L=\mathrm {tr} P\left( \exp {i\int _0^{1/T}A_0({\mathbf {x}},\tau )d\tau }\right) , \end{aligned}$$

where $A_0$ is the gauge potential and $\tau$ is the Euclidean time, $1/T \ge \tau \ge 0$. The loop is an extended object defined in four dimensions. However, since it is parallel to the Euclidean time, we have in fact a 3D object. Note that condensation of the Polyakov lines at $T>T_\mathrm{c}$ has been discussed in many papers, for a review see, e.g., [2493]. If this is true, then the Polyakov lines could be considered as an example of lower-dimensional defects in the language used here.

It is worth mentioning that in the case of supersymmetric gauge theories, with elementary scalar fields, the theory of defects is developed much further, for a review see, e.g., [2494]. Moreover, some features of the defects present in SUSY YM theories are in striking accord with the lattice observations concerning pure Yang–Mills theories (with no elementary scalar fields). In particular, in both cases the fields of monopoles are locked onto the magnetic surfaces (defined independently):

8.6 $$\begin{aligned} \epsilon _{ijk}H^i\Sigma ^{jk} = 0, \end{aligned}$$

where $H^i$ is the magnetic field of monopoles and $\Sigma _{jk}$ are surface elements, constructed from the tangent vectors. However, these two approaches—lattice studies of the defects in pure Yang–Mills case and theoretical studies of defects in the supersymmetric case—have been developing independently, with almost no interaction between the corresponding mini-communities.

A new chapter in the theory of gauge interactions with strong coupling was opened with the formulation of the Maldacena duality, for a review see, e.g., [2495]. It was forcefully argued that the infrared completion of gauge theories is provided by string theories with extra dimensions and non-trivial geometry. The ordinary $4D$ space, where the gauge theories are defined, is assumed to constitute a boundary of the multi-dimensional space. There are certain rules to relate the stringy physics in the extra dimensions to the physics of gauge theories in ordinary four dimensions. For this reason one talks about the “holographic” approach to gauge theories. Exact results apply, however, only to supersymmetric gauge theories (with elementary scalars) in the limit of a large number of colors, $N_\mathrm{c}\rightarrow \infty$.

In the case of pure Yang–Mills theories, with no elementary scalar fields, the strongest claim was made quite some time ago [2415, 2496], and not much progress has been made since then. Namely, it was shown that in the *far infrared limit*, i.e., formally in the limit

$$\begin{aligned} R \gg \Lambda _\text {QCD}^{-1}, \end{aligned}$$

the pure, large-$N_\mathrm{c}$ Yang–Mills theory belongs to the same universality class as a particular string theory, specified in [2415, 2496]. However, this very string theory in the ultraviolet is dual to a supersymmetric *five-dimensional* Yang–Mills (YM) theory which is radically different from the YM theory in $4D$ in which we are interested. Thus, only large-distance, or non-perturbative physics of the gauge theories can be captured within this model. On the other hand, the separation between the perturbative and non-perturbative contributions is actually not uniquely defined at any distances, large distances included.

Nevertheless, it is just in the case of vacuum defects that the holographic approach can be tested. Indeed, from the lattice simulations we know that there are percolating defects which survive, therefore, in the far infrared. Remarkably, the holographic approach based on [2415, 2496] is able to explain the basic observations concerning the vacuum structure of pure Yang–Mills theories.

A nonexhaustive list of theoretical predictions looks as follows:

- The model incorporates, without any tuning, the confinement phenomenon [2415, 2496]; i.e., it reproduces the large-distance behavior of the heavy-quark potential (see (8.1)) with $\sigma \sim \Lambda _\text {QCD}^2$. Geometrically, confinement is related to the properties of a fifth, $z$-direction. The physical meaning of the coordinate $z$ is that it is conjugate to the resolution of measurements. In more detail, $z\rightarrow 0$ corresponds to the ultraviolet limit, or to measurement with fine resolution. Larger values of $z$ correspond to momentum transfer of order $\Delta p \sim 1/z$. One of the basic geometric properties of the theory considered is the existence of a horizon,
  $$\begin{aligned} z \le z_\mathrm{H} \sim \Lambda _\text {QCD}. \end{aligned}$$
  One can show that the existence of this horizon in the $z$-direction implies confinement. Moreover, there is indeed a string stretched between the heavy quarks.
- The stringy completion of gauge theories drastically extends the number of topologically stable classical solutions, see, e.g., [2497, 2498] and references therein. The geometric reason is that the model [2415, 2496] has two compact dimensions, Euclidean time (as usual) and one extra, sixth dimension $\theta$. Lower-dimensional defects correspond to D0-, D2-, D4-branes.^31^ If the branes wrap around at least one of the compact dimensions, the corresponding solutions are stable. The ordinary instantons correspond to D0-branes wrapped around the $\theta$-direction [2499]. Moreover, it is a general property that wrapping around the $\theta$-coordinate implies a non-trivial topological charge of the defect in terms of the Yang–Mills fields. In particular, there are D2 branes wrapped around $\theta$ which would match topologically charged strings in the vacuum of YM theories [2497, 2498].
- At low temperatures, the D2 branes just discussed are expected to percolate. The geometrical reason is that the radius of the sixth dimension, $R_{\theta }$, depends on the $z$-coordinate. Moreover, the crucial observation is that
  $$\begin{aligned}&R_{\theta }(z_\mathrm{H}) = 0. \end{aligned}$$
  Since the action associated with any defect is proportional to its (D+1)-dimensional volume, the action of defects wrapped around the $\theta$-coordinate vanishes at $z=z_\mathrm{H}$, or in the infrared. This implies vanishing of the action of the D2-branes, and this makes plausible their percolation.
- Holography predicts a phase transition to deconfinement at some $T_\mathrm{c}$ [2415, 2496]. In the geometric language this is a so-called Hawking–Page transition [2500], i.e., a transition between two geometries in general relativity. In the case considered, there are two similar compact directions, $\theta$- and $\tau$-directions. Below $T_\mathrm{c}$,
  8.7 $$\begin{aligned}&R_{\tau }=(2\pi T)^{-1}, \nonumber \\&R_{\theta }(z=0)=\text {const}_{\theta },R_{\theta }(z_\mathrm{H})=0, \end{aligned}$$
  while above $T_\mathrm{c}$ the roles of the two compact dimensions are interchanged:
  8.8 $$\begin{aligned}&R_{\theta }=\text {const}_{\theta }, \nonumber \\&R_{\tau }(z=0)=(2\pi T)^{-1},R_{\tau }(z_\mathrm{H})~=0, \end{aligned}$$
  and the phase transition occurs at $(2\pi T_\mathrm{c})^{-1} = \text {const}_{\theta }.$
- According to the holographic picture, the deconfining phase transition can be viewed as dimensional reduction at finite temperature, $T=T_\mathrm{c}$ [2497, 2498]:
  8.9 $$\begin{aligned}&(4D ~\text {percolation}, T<T_\mathrm{c}) \nonumber \\&\quad \rightarrow (3D~\text {percolation}, T>T_\mathrm{c}). \end{aligned}$$
  Indeed, because of the vanishing of the radius of the time circle at the horizon, $R_{\tau }(z_\mathrm{H})=0$, see (8.8), percolating defects are those which are wrapped around the compact $\tau$-direction at $T>T_\mathrm{c}$. The wrapping around the $\tau$-direction implies in turn that the non-perturbative physics in the infrared becomes three-dimensional, see discussion around (8.5) above.
- Generically, the holographic models predict a low value of the shear viscosity $\eta$ [1864]:
  8.10 $$\begin{aligned} \eta /s = 1/4\pi \!. \end{aligned}$$
  This prediction is shared by the models considered, see, e.g., [2501].

Thus, we can summarize that the holographic model based on [2415, 2496] does, in fact, reproduce all the basic observations concerning defects in pure Yang–Mills theories. However, the predictions are mostly qualitative in nature. Since the holographic model does not work in the ultraviolet, it is not possible to fix scales, such as the tensions associated with the defects.

Also, there is no established one-to-one correspondence between defects inherent to the holography and defects observed on the lattice. The reason is the proliferation of the defects in the holographic model. At the moment, there are a few possibilities open to accommodate the defects known from the lattice studies and new, not-yet-observed defects are predicted. Let us mention in this connection that it is only recently that it was observed that the defects called thermal monopoles in the lattice nomenclature are in fact dyons [2502]:

8.11 $$\begin{aligned} |{\mathbf {E}}^a| = |{\mathbf {B}}^a|, \end{aligned}$$

where ${\mathbf {E}}^a,{\mathbf {B}}^a$ are the color electric and magnetic fields associated with the thermal monopoles.

In recent years it was recognized that the phenomenon of confinement has much in common with superfluidity and superconductivity. This similarity is most explicit in holographic models; for reviews of applications of holography to condensed matter systems see, e.g., [2503, 2504]. Namely, basically similar holographic models describe confinement in four dimensions and superfluidity (superconductivity) in three dimensions.

From the technical point of view, however, this change in dimensionality of the space considered is quite crucial. Namely, the difficulty in solving the confinement problem is that it is reduced to string theory in terms of defects, and the string theory in $4D$ is poorly developed. Reduction by one dimension transforms 2D defects into 1D defects. The one-dimensional defects, in turn, correspond to field theory in the language of the quantum geometry, and field theory is much better understood than string theory. This is the reason why in the case of superfluidity and superconductivity the holographic approach allows us to get more detailed predictions than in the case of the confinement in $4D$. Another implication of this simple counting of dimensions is that in the deconfining phase one can expect to find superfluidity. Indeed, in the deconfining phase the $4D$ non-perturbative physics becomes 3D physics, see (8.9). And, indeed, the holographic models predict a relation (see (8.10)) which is, according to the modern view [1864], the lowest possible value of the shear viscosity.

In the deconfining phase one expects to find a dissipation-free electric current as well. We have in mind the so-called chiral magnetic effect (CME) [2285, 2288, 2505]. The effect is the induction of electric current flowing along the external magnetic field in the presence of a non-vanishing chiral chemical potential $\mu _5$:

8.12 $$\begin{aligned} \varvec{j}_\text {el} = \frac{\mu _5}{2\pi ^2}\varvec{B}_\text {ext}. \end{aligned}$$

There is an exciting possibility that the effect of charge separation with respect to the collision plane observed in experiments at RHIC [2506, 2507] and at ALICE [2282, 2508] is a manifestation of a (fluctuating) chiral chemical potential. For further discussion, see Sect. 6.

From the theoretical point of view, it is most exciting that the current (see (8.12)) is dissipation-free and can exist in equilibrium, provided that the chiral limit is granted [2509, 2510]. In this respect, the CME effect is similar to superconductivity. On the other hand, the current (see (8.12)) is carried by fermionic degrees of freedom and there is, unlike the superfluidity case, no coherent many-particle state. In this sense (8.12) rather describes ballistic transport, i.e., collisionless transport along the external magnetic field, and without any driving force. Unlike the ordinary ballistic transport (which refers simply to propagation at distances less than the mean free path), (8.12) is to be quantum and topological in nature. An explicit quantum state responsible for the dissipation-free flow (see (8.12)) has not been constructed yet.

Discussion of the dissipationless nature of the CME brings us to mention the, probably, most dramatic shift of direction of our studies which is taking place nowadays. We mean exploration of condensed-matter systems which are similar in their properties to relativistic chiral-invariant field theories. The spectrum of fermionic excitations in these systems is linear in the momentum $p$:

8.13 $$\begin{aligned} \epsilon \approx v_\mathrm{s}\cdot p, \end{aligned}$$

where $v_\mathrm{s}$ is the fermionic speed of sound. The spectrum (see (8.13)) is similar to the spectrum of a superfluid. However, now it refers to fermions. The implication is that in such materials there should exist a kind of chiral superconductivity, exhibited by (8.12). Moreover, for the condensed-matter systems the condition of validity of the chiral limit can be satisfied to a much better accuracy than in the case of QCD. This point could be crucial for applications. The best known example of such “chiral materials” is graphene. The analog of the chiral magnetic effect is expected to be observed in semi-metals which are also chiral materials; for details and references see, in particular, [2511].

Apart from the CME, there are other interesting phenomena which are expected to happen in strongly interacting gauge theories in external magnetic fields. In particular, it was argued in [2512] that at some critical value of the external magnetic field there is a phase transition of the ordinary vacuum of QCD to a superconducting state. The estimate of the critical field is

8.14 $$\begin{aligned} (B_\text {ext}^\text {crit})^2 \approx (\text {0.6~GeV})^2. \end{aligned}$$

Moreover, one expects that the new superconducting state represents a lattice-like structure of superconducting vortices, see [2513] and references therein.

To summarize, the most intuitive model of confinement, the so-called dual superconductor, appealed to the analogy with superconductivity [2483–2485, 2514–2518]. However, there is no complete implementation of this analogy so far because confinement in $4D$ gauge theories is rather related to string theory which is not developed enough yet. This relation to strings is manifested especially clearly once one turns to the study of defects responsible for the confinement [2486, 2488–2490]. However, above the critical temperature, $T>T_\mathrm{c}$, the non-perturbative physics of the gauge theories comes much closer to the physics of superconductivity [2497, 2498, 2501]. This time it is a relativistic, or chiral superconductivity [2285, 2288, 2505] which is a new chapter in theoretical physics. The phenomenon of the chiral superconductivity seems inherent not only to relativistic field theories but to some condensed-matter systems, like graphene and semimetals, as well.

### Functional methods

As we have seen above, the confining field configurations are, at least to our current understanding, given by lower-dimensional defects. Furthermore, the long-range correlations in between these defects are of crucial importance. This makes confinement a phenomenon based on the behavior of glue in the deep infrared.

It is evident that in such a situation, at least to complement lattice gauge theory, continuum methods are highly desirable. As stated in the introduction to this chapter, a perturbative description of confinement is excluded, leaving us with a need for non-perturbative tools in quantum field theory. One of the very few such approaches is given by the one of functional methods. We use here this term summarizing all those non-perturbative methods which are based on generating functionals and/or Green functions. The basic idea is to rewrite exact identities in between functionals such that they become amenable to an exact treatment in certain kinematical limits and controlled truncation schemes for general kinematics. The truncated set of equations is then subsequently solved numerically. Typically the cost of obtaining numerical solutions is then orders of magnitude less than for a lattice Monte Carlo calculation. In the last decade several of these methods have been used to study the infrared behavior of QCD, amongst them most prominently Dyson–Schwinger equations (see, e.g., [2519, 2520]), exact renormalization group equations (see, e.g., [2521]), $n$-particle irreducible actions (see, e.g., [2522]), and the so-called pinch technique (see, e.g., [2523]). Several recent investigations exploit possible synergies and use different functional methods in a sophisticatedly combined way. Before going into details a few general remarks are in order.

The challenge to describe confinement adequately is given by the fact that the physical Hilbert space of asymptotic (hadron) states does not contain any states with particles corresponding to the elementary fields in QCD, i.e., quarks and gluons. For a satisfactory description of color confinement within local quantum field theory, the elementary fields have to be disentangled completely from a particle interpretation. Within (non-perturbative) gauge field theories the elementary fields implement locality. Those fields are chosen according to the underlying symmetries and charge structure and reflect only indirectly the empirical spectrum of particles. Furthermore, to circumvent the production of colored states from hadrons, strong infrared singularities are anticipated. This expectation is supported by the fact that the absence of unphysical infrared divergences in Green functions of elementary fields would imply colored quark and gluon states in the spectrum of QCD to every order in perturbation theory [2524]. And, even more directly, the linearly rising static potential, discussed in the last section, indicates a strong “$1/k^4$-type” infrared singularity in four-point functions of heavy colored fields.

It will be useful for the following discussion to revisit the formal argument for the non-perturbative nature of the confinement scale in four-dimensional gauge field theories: In the chiral limit QCD is classically scale invariant. It therefore needs to dynamically generate the physical mass scale related to confinement. Furthermore, it is an asymptotically free theory with a Gaussian ultraviolet fixed point, and its renormalization group (RG) equations, in the presence of such a mass scale, imply (at least in expressions for physical quantities) an essential singularity in the coupling at $g =0$. The dependence of the RG invariant confinement scale on the coupling and the renormalization scale $\mu$ near the ultraviolet fixed point is determined by

8.15 $$\begin{aligned} \Lambda = \mu \exp \left( - \int ^g \frac{dg'}{\beta (g')} \right) \mathop {\rightarrow }\limits ^{g\rightarrow 0} \mu \exp \left( - \frac{1}{2\beta _0g^2} \right) , \end{aligned}$$

where with asymptotic freedom $\beta _0>0$. Since all RG invariant mass scales in QCD at the chiral limit will exhibit the behavior (see (8.15)) up to a multiplicative constant, this has, besides the inadequacy of a perturbative expansion for the problem at hand, another important consequence: in the chiral limit the ratios of all bound state masses do not depend on any parameter.

The objectives of the application of functional methods to QCD and hadron physics can be typically separated into two issues: One is the description of hadrons and their properties from elementary Green functions. This is described in Sect. 3. The other is the understanding of fundamental implications of QCD as, e.g., dynamical breaking of chiral symmetry or the axial anomaly. One should note that the formation of bound states with highly relativistic constituents provides hinge between the two types of investigations. But most prominently, a possible relation of the phenomenon of confinement to the infrared behavior of QCD amplitudes has been the focus of many studies.

Although one aims at the calculation of physical and therefore gauge-invariant quantities, functional methods (based on the Green functions of elementary fields) are required by mere definition to fix the gauge. Most investigations have been performed in Landau, Coulomb, or maximally Abelian gauge. The reasons for the respective choices are quite distinct. As studies in Landau gauge are in the majority, we discuss them first.

Some relations between different confinement scenarios become most transparent in a covariant formulation which includes the choice of a covariant gauge. First, we note that covariant quantum theories of gauge fields require indefinite metric spaces. Abandoning the positivity of the representation space already implies to give up one of the axioms of standard quantum field theory. Maintaining the much stronger principle of locality, gluon confinement then naturally relates to the violation of positivity in the gauge field sector. Therefore one of the main goals of corresponding lattice and functional studies of the Landau gauge gluon propagator was to test them for violation of positivity. As a matter of fact, convincing evidence has been found for this property, see, e.g., [2525] for a recent review.

Noting that positivity violation beyond the usual perturbative Gupta–Bleuler or Becchi–Rouet–Stora–Tyutin (BRST) quartet mechanism^32^ has been verified, the question arises how a physical positive-definite Hilbert space can be defined. If, in Landau gauge QCD, BRST symmetry is softly broken (as some recent investigations indicate, see below) the answer is unknown. On the other hand, for an unbroken BRST symmetry the cohomology of the BRST charge provides a physical Hilbert space as has been shown more than three decades ago [2527]. Given some well-defined assumptions, known as Kugo–Ojima confinement criteria, it has been subsequently proven that in this scenario the color charge of any physical state must vanish. As a corollary to these confinement criteria, it is shown that then the ghost propagator diverges more than a massless pole [2528]. Such a behavior is exactly the one found in one type of solutions of Dyson–Schwinger and Exact Renormalization Group studies. It now goes under the name of the scaling solution and is characterized by an infrared enhanced ghost and an infrared vanishing gluon propagator with correlated infrared exponents, see, e.g., [2525] and references therein.

Lattice calculations of Landau gauge propagators lead, however, seemingly to another conclusion, namely an infrared finite gluon propagator and a simple massless ghost propagator,^33^ see, e.g., [2532–2535]. Functional equations, on the other hand, also have such a type of solution, and as matter of fact, it turns out that these are actually a whole one-parameter family of solutions depending on the chosen renormalization constant for one of the propagators [2536–2538]. These are called either decoupling or massive solutions. The latter name should, however, be understood with some care. Of course, in Landau gauge the gluon propagator, although infrared finite, stays transverse. No degenerate longitudinal component of the gluon develops as it is the case in the Higgs phase of Yang–Mills theory with a massive gauge boson: Also for the decoupling or “massive” solution the gluon stays in the massless representation of the Poincaré group with only two polarizations attributed, and as already true on the perturbative level the timelike and the longitudinal gluon stay in the fundamental BRST quartet together with the Faddeev–Popov ghost and the antighost.

The relation between the one scaling and many decoupling solutions can be understood most easily if one chooses to renormalize the ghost propagator at vanishing four-momentum: Denoting by $G(p^2)$ the dressed ghost renormalization function, a non-vanishing choice for $G^{-1}(0)$ leads to one of the decoupling solutions, choosing $G^{-1}(0)=0$ to the scaling solution which then identifies itself as one of the two endpoints of the one-parameter family of solutions.

Recently, a verification of the multitude of propagator solutions has been obtained within a lattice calculation [2539]: On the lattice it turns out that the choice of eigenvalues of the Faddeev–Popov operator in between different Gribov copies of the same configurations (and all of them fixed to Landau gauge!) provides the discrimination in between different members of the decoupling solution family. Therefore one can conclude that the existence of several solutions of functional equations is related to the difficulties of fixing non-perturbatively the gauge in the presence of the Gribov ambiguity as has been already speculated in [2540] based on lattice calculations, where Gribov copies have been chosen on the basis of the infrared behavior of the ghost propagator.

Another well-investigated topic for the realization of confinement is the so-called Gribov–Zwanziger scenario. The generic idea is hereby to take into account only one gauge copy per gauge orbit. Within the state of all gauge field configurations the ones fulfilling the naïve Landau gauge, i.e., the transverse gauge fields, form a “hyperplane” $\Gamma = \{A:\partial \cdot A=0\}$. A gauge orbit intersects $\Gamma$ several times and therefore gauge fixing is not unique. The so-called minimal Landau gauge, obtained by minimizing $||A||^2$ along the gauge orbit, is usually employed in corresponding lattice calculations. It restricts the gauge fields to the Gribov region

8.16 $$\begin{aligned} \Omega&= \{A: ||A||^2 \, \text {minimal}\} \nonumber \\&= \{A: \partial \cdot A=0, -\partial \cdot D (A) \ge 0 \}, \end{aligned}$$

where the Faddeev operator $-\partial \cdot D (A)$ is strictly positive definite. Phrased otherwise, on the boundary of the Gribov region, the Gribov horizon, the Faddeev operator possesses at least one zero mode. Unfortunately, this is not the whole story. There are still Gribov copies contained in $\Omega$, therefore one needs to restrict the gauge field configuration space even further to the region of global minima of $||A||^2$, which is called the fundamental modular region. Usually, a restriction to the fundamental modular region can be obtained in neither lattice calculations nor functional methods. Note, however, that the restriction to the first Gribov region $\Omega$ is fulfilled when using functional equations as long as the ghost propagator does not change sign. To include contributions from field configurations which are exactly the ones being in $\Omega$ leads to the requirement that the ghost propagator is more singular in the infrared than a simple pole, i.e., one obtains the same condition as in the Kugo–Ojima approach.

In [2541] the relation of the Kugo–Ojima to the Gribov–Zwanziger scenario has been investigated showing that the occurrence of the same condition is not at all accidental but points to a deep connection in between these scenarios. Besides this positive result the authors of [2541] obtained the result that conventional BRST symmetry is softly broken by the introduced boundary terms. Unfortunately, it is not clear yet whether some modified symmetry might be left unbroken. If not, one has to face the disturbing fact that an analysis of the Kugo–Ojima picture leads to a contradiction to one of its basic prerequisites.

To allow within the Gribov–Zwanziger scenario for a less divergent ghost and an infrared non-vanishing gluon propagator the so-called refined Gribov–Zwanziger picture has been developed. Some details can be found in the recent review [2542] and references therein. Although the refined Gribov–Zwanziger scheme yields propagators in qualitative agreement with lattice results, it has not contributed to the question whether and, if so, how, the infrared behavior of Green functions is related to confinement. It is probably fair to say that with respect to the Kugo–Ojima and Gribov–Zwanziger pictures of confinement in linear covariant gauges the current understanding is inconclusive. In order to make progress several questions need to be answered: First, is BRST softly or dynamically broken in Landau gauge QCD? Second, are there other symmetries similar to BRST which need or should be considered? Third, is the multitude of possible infrared behaviors of QCD Green functions a failure of the employed methods, or are all these solutions correct ones in the sense that their existence is an issue of complete non-perturbative gauge fixing and all of them lead to identical gauge-invariant observables?

Much work on functional approaches to Coulomb gauge QCD has been performed over the last decades, see, e.g., [2543] and references therein. On the one hand, there is no confinement without Coulomb confinement [2544], and the strong infrared divergence of the time component of the gluon propagator seems to offer a relatively easy understanding of confinement. On the other hand, functional methods for Coulomb gauge QCD have proven to be utterly complicated and no definite conclusion can be reached yet. Given the fact that lattice results leave room for (but also do not show) the analog of the Gribov–Zwanziger scenario, it seems worthwhile to continue the corresponding efforts.

As explained in detail in the previous section, an intriguing scenario for confinement is the dual-superconductor picture. Intimately related to this picture is the use of the so-called maximally Abelian gauge. The corresponding gauge condition is such that it maximizes the diagonal part of the gluon field.^34^ This gauge keeps Poincaré invariance but breaks the covariance under gauge transformations. Quite general arguments allow to establish a connection between confinement, on the one hand, and the dominance of the Abelian gluon field components in the deep infrared on the other hand. Therefore it is encouraging that this picture has been verified in lattice calculations [2545] and in an exact infrared analysis of combined functional equations [2546]. Nevertheless, the provided evidence is not (yet) compelling. Progress has been made, however, with respect to the understanding of the Kugo–Ojima scenario in the maximally Abelian gauge: Whereas a naïve implementation of the Kugo–Ojima criteria fails [2547, 2548], a generalization of this confinement scenario to Coulomb and the maximally Abelian gauge has been constructed recently [2549].

All these studies described so far in this subsection concentrated on the Yang–Mills sector of QCD. They provide essential insights into color confinement (and hereby especially gluon confinement) but put the question of quark confinement aside. In several recent studies—see [2550] and references therein—the question of quark confinement has been addressed by computing the Polyakov loop potential from the fully dressed primitively divergent correlation functions. For static quarks with infinite masses the free energy of a single quark will become infinite as the following Gedanken experiment shows: Removing the antiquark in a colorless quark–antiquark pair to infinity requires an infinite amount of energy for a confined system. The gauge field part of the related operator is the Polyakov loop (see (8.5)), and the free energy of the “single” quark state, $F_q$, can be expressed with the help of the expectation value of $L$

8.17 $$\begin{aligned} \langle L \rangle \propto \exp (-F_q/T). \end{aligned}$$

Therefore, $\langle L \rangle$ is strictly vanishing in the confined phase (but will be nonzero in the deconfined phase). For gauge groups $\mathrm{SU}(N_\mathrm{c})$ this relates the question of confinement to the center symmetry $Z_{N_\mathrm{c}}$: In the center-symmetric phase the only possible value for $\langle L \rangle$ is zero, and one necessarily has confinement. Exploiting (i) $L[\langle A_0 \rangle ]\ge \langle L[A_0] \rangle$, and (ii) the fact that the full effective potential related to $L[\langle A_0 \rangle ]$ can be calculated in terms of propagators in constant $A_0$ background within functional methods, allows to derive a criterion for quark confinement in terms of the infrared behavior of the ghost and gluon propagators, see [2550] and references therein. Corresponding studies have been performed in the Landau gauge, the Polyakov gauge and in the Coulomb gauge hereby confirming the gauge independence of the formal results.^35^ The main result of these studies can be summarized as follows: infrared suppression of gluons but nonsuppression of ghosts is sufficient to confine static quarks.

A similar link of confinement to the infrared behavior of gauge-fixed correlation functions has been established in the last years with the help of so-called dual order parameters, see e.g., [2553] and references therein. These order parameters are, on the one hand, related to the spectral properties of the Dirac operator [2554] and therefore tightly linked to the quark correlation functions, on the other hand, they represent “dressed” Polyakov loops. Corresponding calculations have been extended to fully dynamical 2- and 2+1-flavor QCD at non-vanishing temperatures and densities. It turns out that the different classes of here discussed order parameters are closely related to each other, for a discussion see e.g., [2555].

Summarizing recent work on this topic one can conclude that, both on a quantitative and a qualitative level, confinement criteria have been developed further and one has gained more insight into the relation of confinement to the infrared properties of QCD with the help of functional approaches. These studies, however, provide only a basis to tackle the problem of the dynamical origin of confinement.

In this context one should note that the above discussion does not touch on the origin of the linearly rising potential between static quarks. First of all, one has to realize that the question whether and how such a linearly rising static potential can be encoded in the $n$-point Green functions of quenched QCD is a highly non-trivial one. In lattice gauge theory, this potential is extracted from the behavior of large Wilson loops. Due to the exponentiation of the gluon field the Wilson loop depends on infinitely many $n$-point functions. Therefore, the observed area law of the Wilson loop does not provide a compelling reason why a finite set of $n$-point functions should already lead to confinement in the sense of a linearly rising potential. On the other hand, one can show that an infrared singular quark interaction can provide such a linearly rising potential. The typical starting point for such an investigation is the hypothesis that some tensor components of the quark four-point function diverge like $1/k^4$ for small exchanged momentum $k$. If such an infrared divergence is properly regularized [2556] and then Fourier transformed, it leads, in the nonrelativistic limit, to a heavy quark potential with a term linear in the distance $r$, i.e., to the anticipated linearly rising potential. This provides an example how confinement can be encoded already in a single $n$-point function.

With the Landau gauge gluon being confined (instead of being confining) it is immediately clear that in Landau gauge QCD the quark–gluon vertex function needs to have some special properties if quark confinement is realized in the quark four-point function as described above. In this respect it is interesting to note that in the scaling solution of Dyson–Schwinger and Functional Renormalization Group Equations the quark–gluon vertex can be infrared singular such that the four-point function assumes the $1/k^4$ singularity [2557]. Furthermore, such an infrared singularity provides a possibility of a Witten–Venezanio realization of the $U_A(1)$ anomaly within a Green function approach [272]. As the origin of the pseudoscalar flavor singlet mass in the Witten–Veneziano relations is the topological susceptibility, these findings verify the deep connection of the infrared behavior of QCD Green functions to the topologically non-trivial properties of the QCD vacuum. Note that such a relation between Green functions and vacuum comes naturally in the Gribov–Zwanziger picture of confinement: In the deep infrared Green functions are dominated by the field configurations on the Gribov horizon which, on the other hand, are mostly (or maybe even completely) of a topologically non-trivial type.

Taken together all this motivates the idea that in Landau gauge QCD the quark–gluon vertex is of utter importance, and therefore it is a focus of several recent studies, see, e.g., [2558, 2559] and references therein. These are not only interesting with respect to confinement but show also some very important results for the understanding of dynamical chiral symmetry breaking. Usually one considers the generation of quark masses as the most important effect of chiral symmetry breaking. The recent studies of the quark–gluon vertex prove unambiguously that the dynamical generation of scalar and tensor components in this vertex takes place. In the deep infrared the chiral symmetry violating scalar and tensor interactions are as strong (if not even stronger) as the chiral symmetry respecting vector interactions: even in the light quark sector QCD generates, due to chiral symmetry breaking, scalar confinement, in addition to vector confinement, dynamically.

### Mechanism of chiral symmetry breaking

Already in 1960 Nambu [2560] concluded from the low value of the pion mass that the pion is a collective excitation (Nambu–Goldstone boson) of a spontaneously broken symmetry. He suggested that the breaking of chiral symmetry gives origin to a pseudoscalar zero-mass state, an idealized pion. After the formulation of the QCD Lagrangian it turned out that for massless quark fields $\psi$ (the chiral limit) left and right-handed species

8.18 $$\begin{aligned} \psi _r=\frac{1}{2}(1+\gamma _5)\psi ,\quad \psi _l=\frac{1}{2}(1-\gamma _5)\psi , \end{aligned}$$

are not coupled, they have independent $\mathrm{SU}(N_\mathrm{f})$ symmetries, $\mathrm{SU}(N_\mathrm{f})_l\times \mathrm{SU}(N_\mathrm{f})_r$, where $N_\mathrm{f}$ is the number of flavors. These symmetries can be decomposed into vector and axial vector symmetries, $\mathrm{SU}(N_\mathrm{f})_V\times \mathrm{SU}(N_\mathrm{f})_A$. The small pion mass $m_\pi =140~\mathrm{MeV}$ is an indication that in the ground state of QCD the axial vector symmetry is broken. In the chiral limit it is only broken by the dynamics of QCD and not by the Lagrangian. This spontaneous breaking of chiral symmetry (SB$\chi$S) is an effect which is strongly related to the structure of the non-perturbative vacuum of QCD. The only method at present available to tackle this non-perturbative problem is lattice QCD. As discussed in detail in Sect. 8.1, lattice studies of the vacuum of Yang–Mills theories revealed the existence of infinite clusters of surfaces with quantized magnetic fluxes (vortices) and of trajectories of magnetic monopoles localized on vortices. If monopoles or vortices are removed from the vacuum, both confinement and chiral symmetry breaking [2561] are gone. Whereas the vortex and monopole pictures give a consistent picture of quark confinement, the mechanism for chiral symmetry breaking is not yet clarified and therefore is under intensive discussion. There are several recent investigations possibly indicating where to search for this mechanism. The main questions to be addressed are:

- What are the configurations/degrees of freedom responsible for chiral symmetry breaking?
- How do we study them and what are the quantitative general results so far?
- Are quark confinement and chiral symmetry breaking related, and if yes, how?

The origin of chiral symmetry breaking may be described as an analog to magnetization, its strength is measured by the fermion condensate

8.19 $$\begin{aligned} \bar{\psi }\psi =\bar{\psi }_l\psi _r+\bar{\psi }_r\psi _l, \end{aligned}$$

which is an order parameter for chiral symmetry breaking. It is a vacuum condensate of bilinear expressions involving the quarks in the QCD vacuum, with an expectation value $\langle 0|\bar{\psi }\psi |0\rangle \approx -(250~\mathrm{MeV})^3$ given by phenomenology and confirmed by direct lattice evaluations (see, e.g., [2562]). The Banks–Casher equation [2563]

**Fig. 85:**
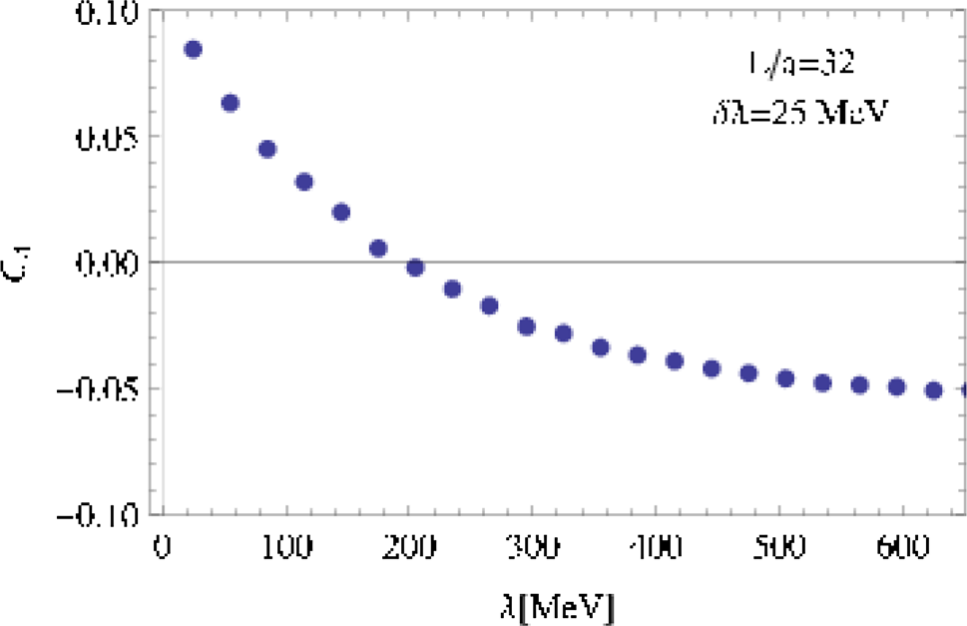
$C_A(\lambda )$ for an $L=32a$ lattice with $a=0.085~$fm of quenched QCD. From [2565, 2566]

8.20 $$\begin{aligned} \langle 0|\bar{\psi }\psi |0\rangle =-\pi \rho (0), \end{aligned}$$

relates this expectation value to the density $\rho (0)$ of near-zero Dirac eigenmodes, i.e., low-lying nonzero eigenmodes $\psi _\lambda$ of the Dirac equation $D\psi _\lambda =\lambda \psi _\lambda$, distributed around $\lambda =0$. Hence, the breaking of chiral symmetry should be imprinted in the chiral properties of the near-zero modes. Since the Dirac eigenmodes appear in pairs with eigenvalues $\pm \lambda$ and have opposite chiralities, there can be no preference for left or right modes, hence the modes have to have specific chiral properties locally. Reference [2564–2566] considers the left-right decomposition (see (8.18)) of the local value $\psi _{\lambda }(x)$ of Dirac modes. For an ensemble of gauge configurations they analyze a probability distribution $\mathcal {P}_\lambda (|\psi _r|,|\psi _l|)$ of these local values in some surrounding $\delta \lambda$. In order to determine whether the dynamics of QCD enhances or suppresses the polarization, they define an uncorrelated distribution $\mathcal {P}_\lambda ^\text {u}(|\psi _r|,|\psi _l|)= P_\lambda (|\psi _r|)P_\lambda (|\psi _l|)$ from $P_\lambda (|\psi _r|)=\int d\psi _l\mathcal {P}_\lambda (|\psi _r|,|\psi _l|)$. Then, they determine whether the correlation $C_A$ for a sample chosen from $\mathcal P_\lambda$ is more polarized than a sample chosen from $\mathcal P_\lambda ^\text {u}$, indicating enhanced polarization for $C_A>0$ and anticorrelation for $C_A<0$. The values of $C_A(\lambda )$ for an $L=32a$ lattice with $a=0.085~$fm of quenched QCD in Fig. 85 show that the lowest modes exhibit a dynamical tendency for chirality, while the higher modes dynamically suppress it.

**Fig. 86:**
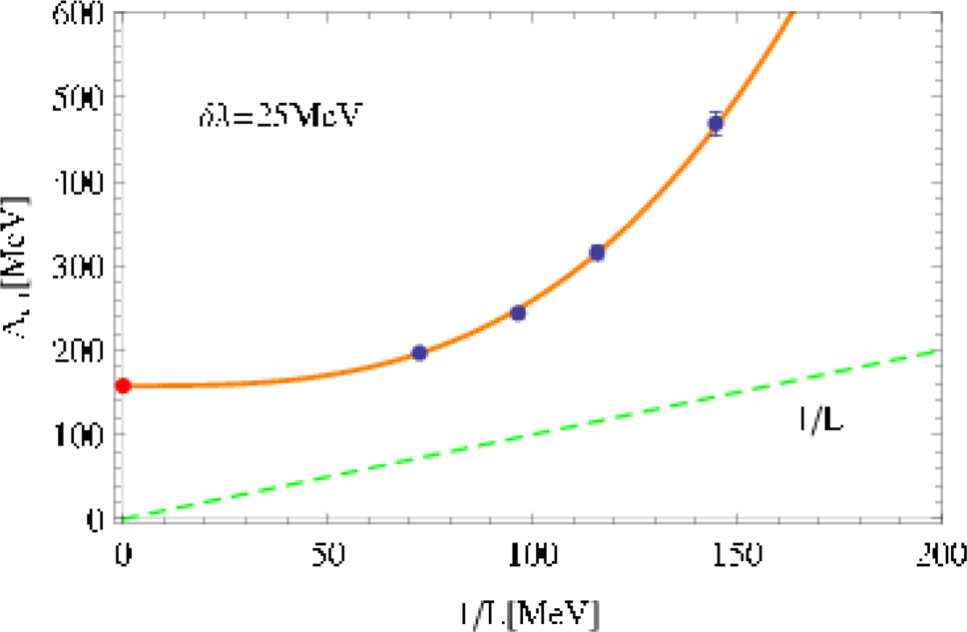
Infinite volume extrapolation of $\Lambda _\mathrm {ch}$. From [2565, 2566]

Chirally polarized low-energy modes condense and are thus carriers of the symmetry breaking. The width $\Lambda _\mathrm {ch}$ of the band of condensing modes provides a new dynamical scale as the dependence on the infrared cutoff in Fig. 86 indicates, where the numerical data are compared with a fit of the form $\Lambda _\text {ch}(1/L) = \Lambda _\text {ch}(0) + b\, (1/L)^3$ and the cutoff $1/L$ itself. This fit yields an infinite volume limit of $\Lambda _\text {ch}\approx 160~\mathrm{MeV}$.

Further, [2565, 2566] presents evidence that $\Lambda _\text {ch}$ is nonzero in the chiral limit of $N_\mathrm{f}=2+1$ QCD, and spontaneous breaking of chiral symmetry thus proceeds via chirally polarized modes, and $\Lambda _\text {ch}$ vanishes simultaneously with the density of near-zero modes when temperature is turned on.

This leads to the question of the origin of the near-zero modes. A first indication about the origin of the near-zero modes came from the instanton liquid model [2567–2569]. There is no unique perturbative vacuum of QCD, different vacua are characterized by a winding number. Instantons and anti-instantons are transitions between neighboring winding numbers. They have topological charge $Q=\pm 1$ and give rise to a single zero mode $\psi _0$ with eigenvalue $\lambda =0$ and definite chirality, i.e., they exhibit either $\psi _l$ or $\psi _r$. For field configurations with instantons and anti-instantons these (would-be) zero modes get small shifts of their eigenvalues and distribute around zero along the imaginary axis as the Dirac operator is anti-Hermitian, so that they become near-zero modes. Hence, overlapping would-be zero modes belonging to single instantons or anti-instantons split into low-lying nonzero modes and contribute to the above density of near-zero modes. The instanton liquid model provides a physical picture of chiral symmetry breaking by the idea of quarks “hopping” between random instantons and anti-instantons, changing their helicity each time. This process can be described by quarks propagating between quark-instanton vertices. In the random instanton ensemble one finds the value of the chiral condensate $\langle 0|\bar{\psi }\psi |0\rangle \approx -(253~\mathrm{MeV})^3$ [2570], which is quite close to the phenomenological value.^36^ Despite their striking success providing a mechanism for chiral symmetry breaking, instantons are not able to explain quark confinement. There are models where instantons may split into merons [2571], bions [2572], or at finite temperature into calorons [2573], which may provide a monopole-like confinement mechanism. Since the QCD-vacuum is strongly non-perturbative, it does not contain semiclassical instantons but is crowded with topologically charged objects that, after smooth reduction of the action (also known as cooling), may become instantons.

Reference [2574] demonstrates that the above mentioned smoothing procedures affect the dimensionality of the regions where the topological charge density $q(x)$ is localized. They measure the local density $q(x)$ of the topological charge with the trace of the zero-mass overlap operator $D(x,x)$ [2575, 2576]:

8.21 $$\begin{aligned} q(x)=-\mathrm {Tr}\left[ \gamma _5\left( 1-\frac{a}{2}D(x,x)\right) \right] , \end{aligned}$$

where the trace is taken over spinor and color indices. These investigations demonstrate that topological charge and zero modes are localized on low-dimensional fractal structures and tend to occupy a vanishing volume in the continuum limit. With the inverse participation ratio

8.22 $$\begin{aligned} \mathrm{IPR}=N\sum _x^N\alpha ^2(x),\quad \mathrm for \quad \sum _x\alpha (x)=1, \end{aligned}$$

for arbitrary normalized distributions $\alpha (x)$ they derive a fractal dimension of fermionic zero modes. Distributions localized on a single site get $\mathrm{IPR}=N$ and constant distributions $\mathrm{IPR}=1$. With the eigenfunctions $\psi _\lambda$ of the overlap Dirac operator to the eigenvalues $\lambda$ they measure the average over all zero modes and all measured gauge field configurations of the local chiral condensate

8.23 $$\begin{aligned} \rho _\lambda (x)=\psi _\lambda ^\dagger (x)\psi _\lambda (x). \end{aligned}$$

**Fig. 87:**
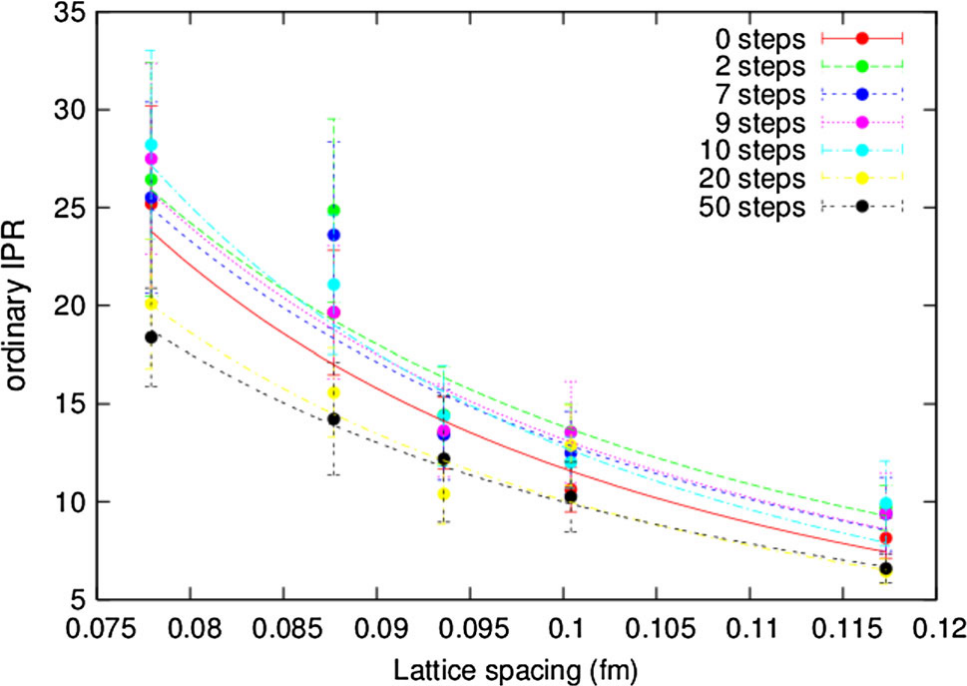
Ordinary IPR for zero modes, (8.23). From [2574]

Figure 87 shows how the localization depends on the lattice spacing $a$ and the number of cooling steps. The finer the lattice, the larger the IPR gets. This agrees very well with the idea that the volume occupied by the fermionic zero modes in the continuum limit approaches zero [2577]. Since zero modes, $\lambda =0$, have definite chirality the results for the local chirality agree with the local chiral condensate.

Performing a number of measurements with various lattice spacings $a$ [2574], one is able to define a fractal dimension $d$ by

8.24 $$\begin{aligned} \mathrm{IPR}(a)=\frac{\mathrm {const}}{a^d}, \end{aligned}$$

see Fig. 88.

**Fig. 88:**
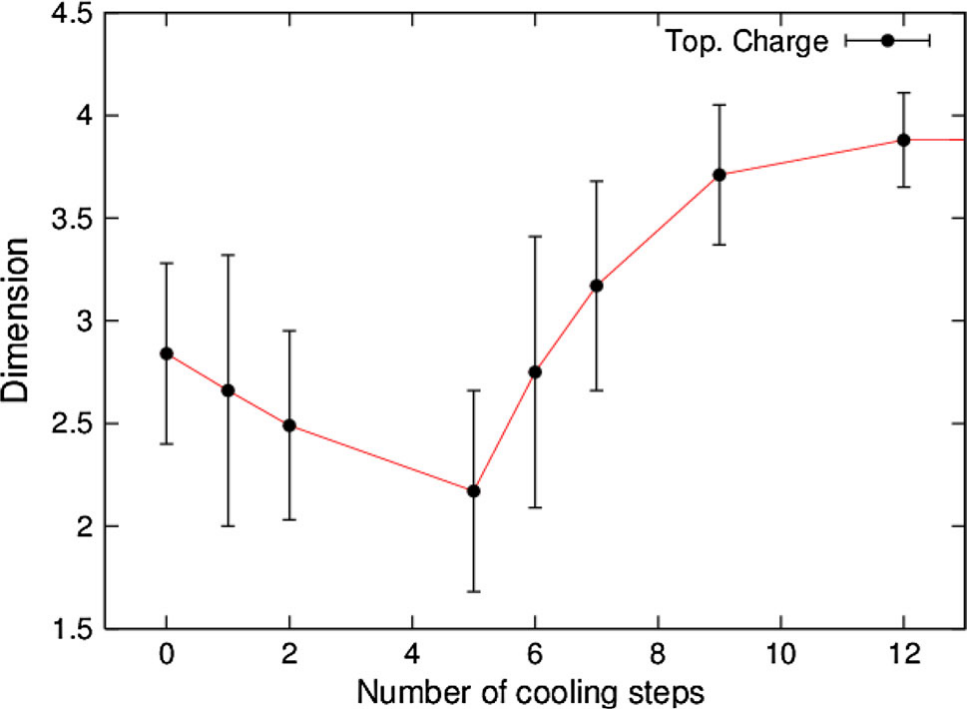
Fractal dimensions at various cooling stages. The *solid line* is shown to guide the eye. From [2574]

These results show that fermionic zero modes and chirality are localized on structures with fractal dimension $2\le D\le 3$, favoring the vortex/domain-wall nature of the localization [2578–2580]. The fractal dimension of these structures depends on the number of cooling steps. A long sequence of cooling iterations destroys the low-dimensional structures leading to gauge fields close to classical minima of the action where instantons dominate the properties of field configurations.

In [2578, 2581] it was shown that center vortices, quantized magnetic fluxes in the QCD vacuum, contribute to the topological charge by intersections with $Q_U=\pm 1/2$ and writhing points with a value of $\pm 1/16$.

Since it is expected that zero modes of the Dirac operator concentrate in regions of large topological charge density, a correlation between the location of vortex intersections and writhing points and the density $\rho _\lambda (x)=|\psi _\lambda (x)|^2$ of eigenmodes of the Dirac operator $D$, where $D\psi _\lambda =\lambda \psi _\lambda$ with $\lambda =0$ in the overlap formulation and $\lambda \approx 0$ in the asqtad formulation supports this picture [2582]. Reference [2583] proposed the observable

**Fig. 89:**
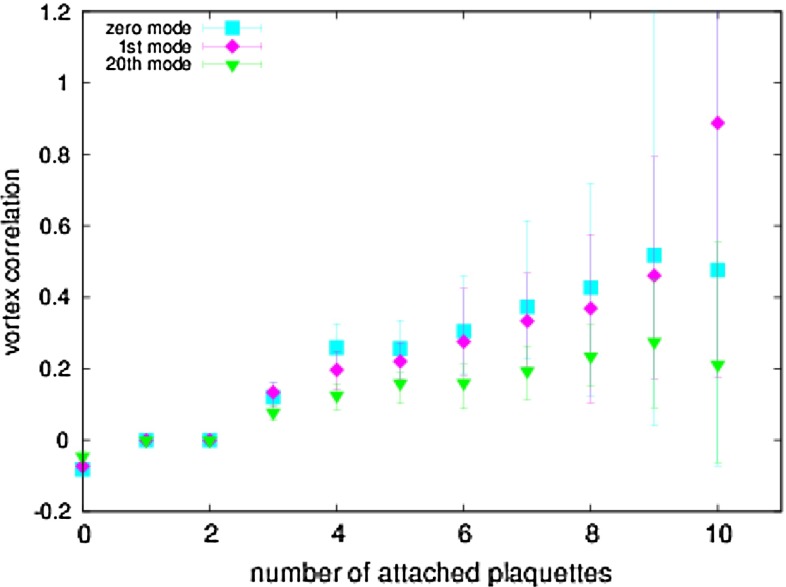
Vortex correlation $C_\lambda (N_v)$ for overlap eigenmodes on a $16^4$ lattice at $\beta _\mathrm {LW}=3.3$. From [2582]

8.25 $$\begin{aligned} C_\lambda (N_v)=\frac{\sum _{p_i}\sum _{x\in H}(V\rho _\lambda (x)-1)}{\sum _{p_i}\sum _{x\in H}1}, \end{aligned}$$

as a measure for the vortex-eigenmode correlation. To explain this formula we have to recall that center vortices are located by center projection in maximal center gauge [2584]. Plaquettes on the projected lattice, “P-plaquettes”, are either $+1$ or $-1$; they form closed surfaces on the dual lattice. Each point on the vortex surface belongs to $N_v$ P-plaquettes. $N_v=0$ we get for points which do not belong to a vortex surface, $N_v=1$ or $2$ is impossible since vortex surfaces are closed, for corner points or points where the surface is flat we get $N_v=3, 4$, or $5$, when the surface twists around a point $N_v=6$ or $7$, and at points where surfaces intersect $N_v\ge 8$. In Fig. 89, we display the data for $C_\lambda (N_v)$ versus $N_v$ computed for eigenmodes of the overlap Dirac operator. The lattice configurations are generated by Monte Carlo simulations of the Lüscher-Weisz action at $\beta _\mathrm {LW}=3.3$. The correlations for the first eigenmode and the twentieth Dirac eigenmode are shown. Since the correlator increases steadily with increasing $N_v$, we conclude that the Dirac eigenmode density is significantly enhanced in regions of large $N_v$.

By the Atiyah–Singer index theorem [2585–2588] zero modes are related to one unit of topological charge. Therefore, the question emerges, how vortex intersections and writhing points are related to these zero modes. Reference [2589] compares vortex intersections with the distribution of zero modes of the Dirac operator in the fundamental and adjoint representation using both the overlap and asqtad staggered fermion formulations in SU(2) lattice gauge theory. By forming arbitrary linear combinations of zero modes they prove that their scalar density peaks at least at two intersection points [2589].

In recent investigations a further source of topological charge was discovered. A contribution with one unit of topological charge comes from colorful center vortices [2590]. Vortices may have a color structure with a winding number and contribute to the topological charge. Covering of the full SU(2) color group leads to actions of a few instanton actions only and indicates that such configurations are possibly appearing in Monte-Carlo configurations. According to the index theorem and the Banks–Casher relation, interacting colorful vortices contribute to the density of near-zero modes.

These observations lead to a picture similar to the instanton liquid model. The lumps of topological charge appearing in Monte-Carlo configurations interact in the QCD-vacuum and determine the density of near-zero modes. Therefore, it is not the true zero modes deciding on the value of the topological charge of a field configuration which lead to the breaking of chiral symmetry. The number of these modes is small in the continuum limit. It is the density of interacting topological objects which leads to the density of modes around zero and, according to the Banks–Casher relation (see (8.20)), determines the strength of chiral symmetry breaking.

Due to the color screening by gluons the string tension of pairs of static color charges in $\mathrm{SU}(N)$ gauge theories depends on their $N$-ality. From the field perspective this $N$-ality dependence has its origin in the gauge field configurations which dominate the path integrals in the infrared. Center vortices are the only known configurations with appropriate properties. Concerning chiral symmetry breaking a remarkable result was found in [2561], namely removing vortices from lattice configurations leads to restoration of chiral symmetry. If one considers that a phase transition of the gauge field influences both gluons and fermions, then one would expect that deconfinement and chiral phase transition are directly related, as indicated by lattice calculations [2591].

It is an interesting check of this picture whether field configurations with restorations of chiral symmetry still have confinement. This problem was attacked recently from two different sides. Using the completeness of the Dirac mode basis and restricting the Dirac mode space by a transition to the corresponding projection operator

8.26 $$\begin{aligned} \sum _\lambda |\lambda \rangle \langle \lambda |=1\quad \rightarrow \quad \hat{P}_A=\sum _{\lambda >k}|\lambda \rangle \langle \lambda |. \end{aligned}$$

**Fig. 90:**
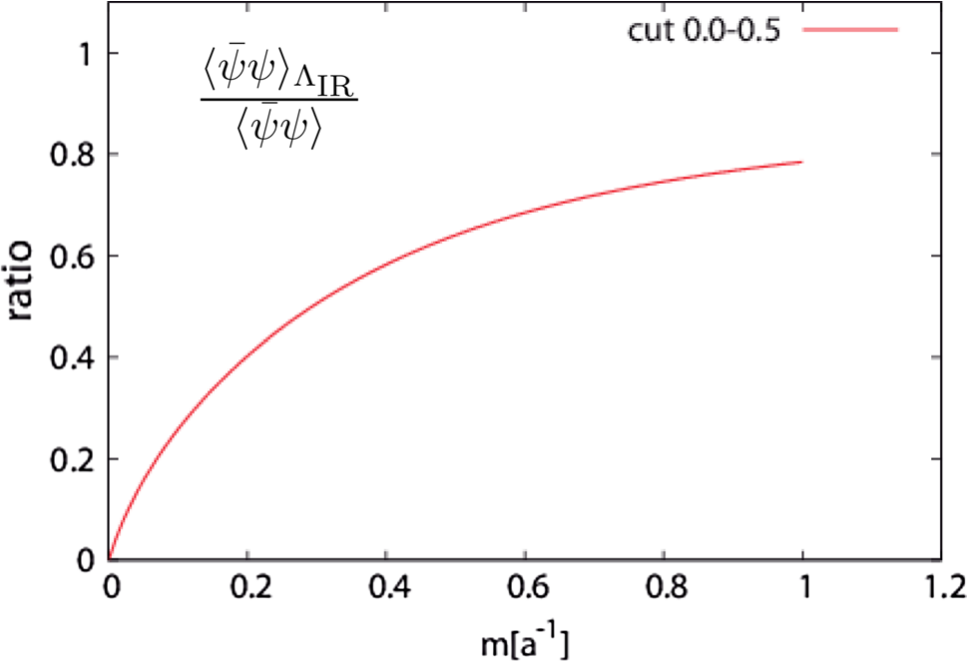
$\langle \bar{\psi }\psi \rangle _{\Lambda _\mathrm{IR}}/\langle \bar{\psi }\psi \rangle$ for an IR cut of $\Lambda _\mathrm{IR}=0.5a^{-1}$, plotted against the current quark mass $m$. A large reduction of $\langle \bar{\psi }\psi \rangle _{\Lambda _\mathrm{IR}}/\langle \bar{\psi }\psi \rangle \simeq 0.02$ is found in the physical case of $m\simeq 0.006a^{-1}\simeq 5~\mathrm{MeV}$. From [2596]

**Fig. 91:**
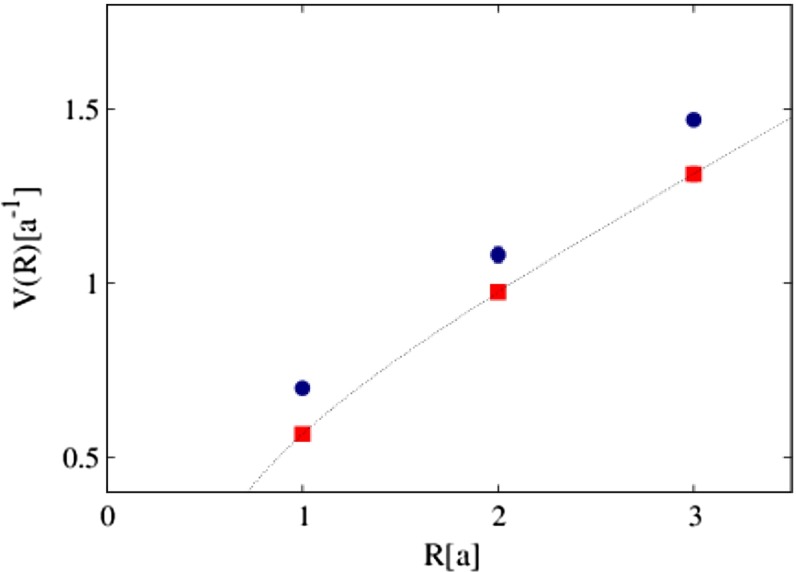
Inter-quark potential (*circles*) after removal of low-lying Dirac modes with the IR-cutoff $\Lambda _\mathrm{IR}=0.5a^{-1} \simeq 0.4~\mathrm{GeV}$ and original potential (*squares*), apart from an irrelevant constant. From [2596]

A manifestly gauge covariant Dirac-mode expansion and projection method was developed in [2592, 2593]. They had to deal with the technical difficulty to find all eigenvalues and eigenfunctions of huge matrices and used therefore the Dirac operator for staggered fermions [2594, 2595] in SU(3)-QCD and rather small $6^4$ lattices. After removing the lowest $k$ Dirac modes they got a strong reduction of the chiral condensate to $2~\%$ in the physical case of $m\simeq 0.006a^{-1}\simeq 5~\mathrm{MeV}$, see Fig. 90. This removal conserved the area law behavior of Wilson loops without modifying the slope. Besides an irrelevant constant the inter-quark potential is almost the same, see Fig. 91. The Polyakov loop remains almost zero indicating that the center symmetry is still unbroken [2596–2598].

The Graz group [522, 2599–2602] studied hadron spectra after cutting low-lying Dirac modes from the valence quark sector in a dynamical lattice QCD calculation. They expressed the valence quark propagators $S$ directly by the eigenfunctions of the Dirac operator and removed an increasing number $k$ of lowest Dirac modes $|\lambda \rangle$

**Fig. 92:**
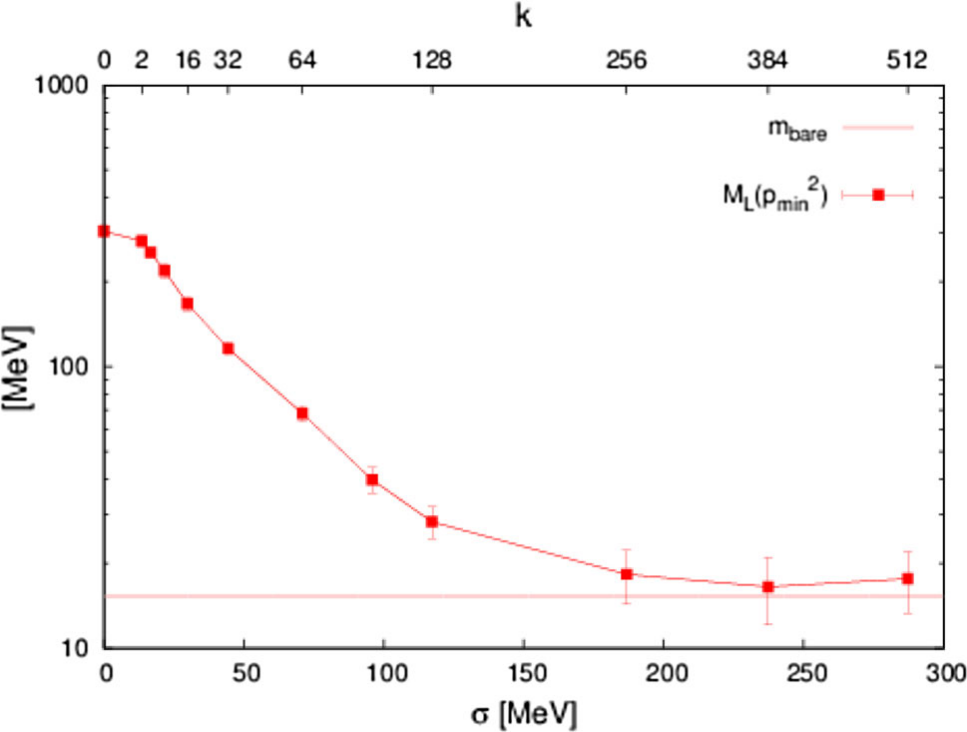
Lattice mass function $M_L(p_\mathrm {min}^2)$ for the smallest available momentum $p_\mathrm {min}=0.13~\mathrm{GeV}$ as a function of the truncation level. On the lower axis the level $k$ is translated to an energy scale. For comparison, the bare quark mass is plotted as a *horizontal line*. From [2603]

8.27 $$\begin{aligned} S_{\mathrm {red}(k)}=S-\sum _{\lambda \le k}\mu _\lambda ^{-1}|\lambda \rangle \langle \lambda |\gamma _5, \end{aligned}$$

with $\mu _\lambda$ the (real) eigenvalues of the Hermitian Dirac operator $D_5=\gamma _5D$. They extracted the mass function $M_L(p^2)$ from the reduced quark propagator (see (8.27)) for chirally improved fermions. In Fig. 92 the dynamical generated mass $M_L(p_\mathrm {min}^2)$ for the smallest available momentum $p_\mathrm {min}=0.13~\mathrm{GeV}$ is plotted as a function of the truncation level $k$. Removing the low-energy modes the dynamic mass generation ceases, and the bare quark mass is approached successively.

**Fig. 93:**
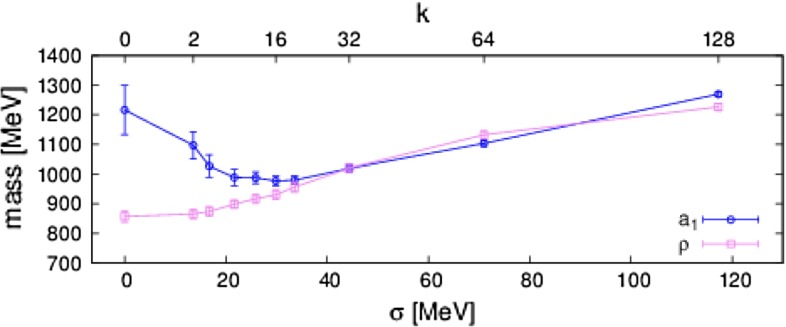
Influence of the removal of the lowest $k$ modes of the Dirac operator on the masses of chiral partner mesons, the vector meson $\rho$ and axial vector meson $a_1$. From [2603]

Except for the pion, the hadrons survive this artificial restoration of chiral symmetry through this truncation. The quality of the exponential decay of the correlators increases by this procedure indicating a state with the given quantum numbers. In Fig. 93 the influence of the truncation of the masses of two mesons which can be transformed into each other by a chiral rotation, the vector meson $\rho$ and the axial vector meson $a_1$ is shown. These would-be chiral partners become degenerate after restoration of chiral symmetry. Interestingly these meson masses increase with increasing truncation level $k$. These results demonstrate that even without a chiral symmetry breaking vacuum confined hadrons can exist, at least with rather large mass.

One obtains a picture for spontaneous chiral symmetry breaking ($\chi$SB) that can be called “kinematical”. Interacting lumps of topological charge lead to low-lying Dirac modes that induce $\chi$SB via the Banks–Casher relation (see (8.20)). Still, there is no clear answer to the question about the dynamics of $\chi$SB. A conjectured mechanism runs as follows. The low-momentum modes change chirality as they enter a combination of electric and magnetic fields present in regions of non-vanishing topological charge density. In such fields, slow color charges would move along spiraling paths changing their momentum and conserving their spin. Fast moving charges would be less influenced by such field combinations. This could explain the importance of low-lying Dirac modes for $\chi$SB and clarify why Goldstone bosons do not survive the removal of low-lying Dirac modes and heavy hadrons tend to increase their masses with increasing removal; see Fig. 93. Hence, field configurations with lumps of topological charge contributions increase the density of low-lying Dirac eigenmodes with pronounced local chiral properties producing a finite chiral condensate.

### Future directions

As described above there are many unsolved interesting problems concerning the vacuum structure of QCD, confinement, and chiral symmetry breaking. Most important, there is still no satisfactory solution to the confinement problem in non-Abelian gauge theories, and therefore no proof that continuum QCD confines. Some of the most interesting questions for future work to generate progress in this field are as follows:

- Can one treat confinement as a string theory of lower-dimensional topological defects in four dimensions? Is confinement related to percolation of these defects?
- Is the BRST symmetry of gauge-fixed non-Abelian Yang–Mills theory softly broken in the non-perturbative domain? If so, what is implied then for the Kugo–Ojima and Gribov–Zwanziger confinement scenarios?
- Is the existence of families of solutions for Green functions of elementary fields related to some yet not fully understood gauge degree of freedom?
- In the Coulomb gauge, what balances the “over-confinement” (i.e., the too large string tension) due the time-component of the gluon field?
- Is there Abelian dominance in the maximally Abelian gauge? Do chromomagnetic monopoles correlate along the lower-dimensional topological defects mentioned above? If so, is the picture of a dual superconductor still valid?
- Does the quark four-point function display the anticipated “$1/k^4$” infrared singularity? If so, in all gauges? Is then the cluster decomposition property violated?
- Can one construct an explicit quantum state responsible for a dissipation-free flow of an electric current along an external magnetic field (chiral magnetic effect)?
- Does there exist a kind of chiral superconductivity, inherent not only to relativistic field theories but to some condensed-matter systems, like graphene and semimetals, as well?
- Do chirally polarized low-energy modes condense? What is the physical origin of the band width $\Lambda _\mathrm {ch}$ of condensing modes?
- Does the result that fermionic zero modes and chirality are localized on structures with fractal dimension $D=2-3$ favor the vortex/domain-wall nature of the localization?
- Which kinds of effective quark–gluon interactions are generated by dynamically breaking chiral symmetry? Will this include a scalar confining force?
- Why do Goldstone bosons not survive the removal of low-lying Dirac modes?
- What is the relative contribution of the various interacting topological objects to the Dirac operator’s density of modes around zero virtuality?
- Do low-momentum modes change chirality in regions of non-vanishing topological charge density with electric **and** magnetic fields present and thus dynamically break chiral symmetry?

An answer to these questions may hold the key to understand infrared QCD and the related phenomena, most prominently, confinement and dynamical chiral symmetry breaking. Especially the confinement problem is one of the truly fundamental problems in contemporary physics. Until it is well understood something essential is lacking in our comprehension of particle and nuclear physics. Although the problems described in this section have proven to be very hard their solution is important, and they are certainly worth pursuing.

## Strongly coupled theories and conformal symmetry

37Most of the multifaceted physics of QCD and a good part of the theories of fundamental interactions beyond the standard model (BSM) are or have sectors that are strongly coupled. It is therefore of relevance to devise new and increasingly sophisticated theoretical approaches to study strongly coupled physics. The same methods often provide significant guidance in other branches of physics, from cosmology to material science. This chapter provides a short review of recent progress and present challenges in the theoretical formulation of gauge theories at strong coupling and their applications to particle and condensed matter physics.

Conformal symmetry has recently emerged as a key ingredient in this context and as a guide in the study of the many aspects of the phase diagram of non-Abelian gauge theories in four spacetime dimensions, as well as in the phenomenological search for models of new physics (NP) beyond the electroweak symmetry breaking (EWSB) scale and the standard model (SM) of particle interactions. The physics output has progressed jointly with the refinement of theoretical and computational approaches. Among the first, gauge–gravity duality and, in particular, the AdS/CFT correspondence [2604] between higher-dimensional string theories living in anti-de Sitter spacetime and conformal field theories (CFTs) living at their boundaries have introduced new classes of strong/weak coupling dualities and allowed predictions for (near)conformal strongly coupled systems that complement other effective field theory approaches, such as the large-$N$ expansion, the functional renormalization group, or methods to solve Schwinger–Dyson equations; examples of these approaches can be found in the rest of this document. Computational approaches essentially amount to lattice field theory, to date the only method we know that should provide the full non-perturbative solution of QCD, once the continuum limit is reached. Lattice field theory investigations have recently benefited from algorithmic advances and a huge step forward in supercomputer technology and performance, see also Sect. 1.

The interplay of conformal symmetry and the strongly coupled regime of quantum field theories has led to new paradigms and has highlighted the existence of families of gauge theories and regions of their phase diagram that might be relevant in describing high energy particle physics between the electroweak symmetry breaking scale and the Planck scale. The same theoretical advances have motivated the development of a number of methods for describing strongly coupled systems in condensed matter physics. Interesting examples in this context are the phase structure and transport properties of materials of the latest generation, such as graphene, non-Fermi liquids, and high-$T_\mathrm{c}$ superconductors.

This chapter is organized in four sections. Section 9.1 provides an overview of the most recent formal developments in quantum field theories with and without supersymmetry, with an emphasis on conformal field theories and the ways they connect to QCD at strong coupling. Section 9.2 discusses in more detail how conformal symmetry can be restored in non-Abelian gauge theories with matter content and outlines the theory of the conformal window. One interesting possibility is that theories close to the conformal window could be realized in nature and play a relevant role for new physics at the weak scale. Section 9.3 discusses electroweak symmetry breaking and BSM scenarios for its realization that involve strongly coupled dynamics and/or spontaneously broken conformal symmetry. In particular, we discuss the theoretical premises for a wide class of strongly coupled models, composite-Higgs or dilaton-Higgs, using a general effective field theory approach to constrain them with electroweak precision measurements and the recent discovery of a Higgs-like boson of about 126 GeV at the Large Hadron Collider (LHC). As an alternative to strongly coupled new dynamics, we revisit the appealing scenario of a minimally extended SM, where an underlying conformal symmetry would govern the dynamics from the Planck scale all the way down to the weak scale. Finally, Sect. 9.4 is devoted to recent advances in the study of condensed matter systems using lattice gauge theory and gauge–gravity duality. We discuss future prospects in Sect. 9.5.

### New exact results in quantum field theory

In this section we review recent developments in exact methods in quantum field theory (QFT), some of which were inspired by string theory and/or the AdS/CFT correspondence.

Many of these developments refer to quantum field theories in the *large* $N$ *limit*. As is well known [2605], gauge theories simplify by scaling the number of colors $N$ to infinity while at the same time sending the coupling constant $g$ to zero, keeping the combination $\lambda = g^2 N$, called the ’t Hooft coupling, fixed. In this limit Feynman diagrams acquire a topological classification, with *planar* diagrams providing the leading contribution to any given process, while the contribution of *non-planar* diagrams is suppressed by powers of $1/N$. Although QCD, whose gauge group is $\mathrm{SU}(3)$, corresponds to the value $N=3$, certain aspects are captured by the large-$N$ approximation.

The large-$N$ limit plays a central role in the AdS/CFT correspondence (gauge–gravity duality). The latter asserts that large-$N$ gauge theories admit a holographic description in terms of higher-dimensional string theories [2604, 2606, 2607]. In certain limits of the parameter space the higher-dimensional string theory can be well approximated by semi-classical supergravity, which allows computations to be performed in the strongly coupled regime of the gauge theory. The best studied example is the duality between the $\mathcal{N}=4$ Super-Yang–Mills (SYM) and type IIB string theory in AdS$_5\times$S$^5$. Several generalizations of the AdS/CFT correspondence have been developed, which attempt to describe gauge theories closer to QCD. For an entrée to the vast literature see the classic review [2608] and Sects. 9.1.1, 9.1.6, 9.2, and 9.4 of this chapter.

A large part of this section is focused on CFTs. While QCD is not conformal, the study of CFTs is important for several reasons. CFTs are the ultraviolet (UV) and infrared (IR) limits of renormalization group (RG) flows of other quantum field theories; so, any other well-defined quantum field theory can be understood as a UV CFT perturbed by relevant operators. Moreover, CFTs can be studied with more general methods (some of which are described below) than the usual perturbative expansion. This allows us to probe them in the strong coupling regime. Finally, CFTs have applications in string theory and condensed matter physics.

#### Integrability of planar $\mathcal{N}=4$ SYM

One of the most remarkable recent achievements in QFT is the proposed exact solution of planar $\mathcal{N}=4$ SYM using methods of integrability and input from the AdS/CFT correspondence. See Ref. [2609] for an extensive review and list of references. Unlike QCD, the $\mathcal{N}=4$ SYM is conformal and does not have asymptotic multiparticle states. Instead, the “spectrum” of the theory is encoded in the conformal dimensions of local, single trace operators. At small values of the ’t Hooft coupling $\lambda =g^2 N$ the conformal dimensions can be computed perturbatively by usual Feynman diagrams. As $\lambda$ is increased the computations quickly become intractable. Nevertheless, it is believed that for any value of the ’t Hooft coupling $\lambda$ the spectrum of the $\mathcal{N}=4$ SYM at large $N$ is governed by a $1+1$-dimensional integrable system. The exact S-matrix of this integrable system has been determined. Using this exact world-sheet S-matrix, the conformal dimensions of single trace operators can be determined by the solutions of complicated algebraic equations derived by the thermodynamic Bethe ansatz or Y-system. For instance, the anomalous dimension of the Konishi operator (a particular single trace operator) has been evaluated for all values of $\lambda$ by solving these equations numerically. As expected, the anomalous dimensions smoothly interpolate between the perturbative values at small $\lambda$ and the AdS/CFT predictions of type IIB string theory on AdS$_5\times$S$^5$ at large $\lambda$. The results from integrability constitute a notable non-trivial verification of the AdS/CFT correspondence. More recently, there have been promising attempts to extend the methods of integrability to the computation of correlation functions and to investigate the connections with scattering amplitudes in the $\mathcal{N}=4$ SYM. It would of course be exciting if integrability persists, in some form, in theories closer to QCD.

#### Scattering amplitudes

The computation of scattering amplitudes in perturbative QCD is of central importance both for theoretical and practical reasons—for instance, the analysis of backgrounds at the LHC. While straightforward in principle, the evaluation of scattering amplitudes using QCD Feynman diagrams grows very quickly in complexity as the number of external lines and/or number of loops is increased.

In the last decades we have seen remarkable progress in developing alternative methods to compute scattering amplitudes in QCD as well as in more general gauge theories, most prominently for the $\mathcal{N}=4$ SYM. These methods are based on “on-shell” techniques—generalized unitarity as well as input from the AdS/CFT correspondence. For a summary of these developments see Ref. [2610]. In the 1980s Parke and Taylor presented a compact formula for the tree-level maximally helicity violating (MHV) amplitudes of gluons in QCD [2611], which is vastly simpler than what appears in the intermediate steps of the computation via Feynman diagrams. More recently, a relation was conjectured between tree-level scattering amplitudes and a string theory in twistor space [2612], which eventually led to generalizations and the Cachazo-Svrcek-Witten rules [2613]. Another important step was the development of the Britto-Cachazo-Feng–Witten on-shell recursion relations [2614]. By considering a particular analytic continuation of tree-level amplitudes and exploiting the fact that, in certain theories, the resulting meromorphic function has simple behavior at infinity of the complex plane, higher-point amplitudes can be reconstructed by gluing together lower-point amplitudes. This technique simplifies the computation of tree level and, to some extent, higher-loop amplitudes. For outcomes of these developments we refer to Sect. 5 of this document.

Further insights are provided by the AdS/CFT correspondence and the work [2615], which relates scattering amplitudes of gluons at strong coupling in the $\mathcal{N}=4$ SYM to minimal area surfaces in AdS. The AdS/CFT computation of scattering amplitudes led to the discovery of a hidden symmetry of amplitudes called *dual conformal invariance*, which was also independently noticed in perturbative field theory computations at weak coupling. It also led to uncovering the relation between Wilson loops and scattering amplitudes; see Ref. [2610] for further discussions and references to the original literature.

These developments suggest that gluon scattering amplitudes, especially those for planar $\mathcal{N}=4$ SYM, may be governed by additional symmetries, such as the dual conformal invariance which together with ordinary conformal invariance closes into a larger “Yangian” symmetry, which may not be manifest in the Lagrangian formulation of the theory. This has led to an ambitious attempt at describing the all-loop scattering amplitudes of $\mathcal{N}=4$ SYM in terms of new mathematical structures; see Ref. [2616] for the latest developments in this direction.

#### Generalized unitarity and its consequences

The main inspiring idea behind generalized unitarity is that the only information needed to compute one-loop amplitudes, independently of the number of external legs, are the coefficients of a very well-known and tabulated set of 1-, 2-, 3-, and 4-point scalar integrals [2617–2619], plus rational parts which have to be added separately [2620]. Each coefficient is sitting in front of a unique combination of polydromic functions (logarithms and di-logarithms) which can be identified by looking at the discontinuities of the amplitude [2621], while the rational parts are not-polydromic in four dimensions. In the pioneering work of Refs. [2622, 2623], the discontinuities are determined analytically by combining different ways of putting on-shell two internal particles in the loop (two-particle cuts), and the rational parts are reconstructed from the soft/collinear limits of the full amplitude. In Ref. [2624] Britto, Cachazo, and Feng generalized, for the first time, this procedure by introducing the concept of a quadruple cut: all possible ways of putting four-loop particles on-shell completely determine the coefficients of the contributing 4-point scalar integrals (boxes), fully solving, at one loop, theories so symmetric that only boxes are present, such as $\mathcal{N}=4$ SYM. Since a quadruple cut factorizes the amplitude in four tree amplitudes, the box coefficients are simply computed in terms of the product of four tree amplitudes evaluated at values of the loop momenta for which the internal particles are on-shell. The solution for general theories—where also lower point functions contribute, such as triangles, bubbles, and tadpoles—is provided by the Ossola–Papadopoulos–Pittau (OPP) approach of Refs. [2625, 2626], in which the coefficients are directly inferred from the one-loop *integrand*. The advantage of this method is that, once the coefficients of the box functions are determined, a simple subtraction from the original integrand generates an expression from which the coefficients of the 3-point scalar functions can be computed by means of triple cuts [2627], and so on. The one-loop integrand can be either determined by gluing together tree-level amplitudes, as in the generalized unitarity methods [2628, 2629], or computed numerically [2630, 2631], the only relevant information being the value of the integrand at certain values of the would-be loop momentum. As for the missing rational parts, OPP uses special tree-level vertices (involving up to four fields and determined once for all for the theory at hand [2632–2634]) to include them, while they can be computed via their d-dimensional cuts in generalized unitarity [2635]. Alternatively, it is possible to construct the rational parts recursively in the number of legs [2628].

Since the integrand of a one-loop amplitude is a tree-level-like object, tree-level Feynman-diagrams-free recursion techniques [2636–2639] can be applied also in generalized unitarity and OPP. This has been dubbed a *“NLO revolution”* and made possible to calculate numerically 20-gluon amplitudes at NLO in QCD [2640] or six-photon amplitudes [2641] in QED, and to attack the NLO computation of complicated processes needed in the LHC phenomenology, such as $t\bar{t} b \bar{b}$ production [2642] (as an irreducible QCD background to the $Ht\bar{t}$ signal), $pp \rightarrow 4$ leptons [2643] (as a background to the Higgsstrahlung production mechanism), $H$ + 3 jets (using the effective $ggH$ coupling) [2644], $W$ + 5 jets [1233], and $pp \rightarrow 5$ jets [2645]. On the basis of the above computational progress, NLO Monte Carlo codes have been constructed in the last few years allowing the LHC experimental collaborations to analyze their data at NLO accuracy. Among them BlackHat [2628], GoSam [2646], HELAC-NLO [2647], and Madgraph5-aMC@NLO [2648–2650]. The last two Monte Carlo programs are general purpose ones: the user inputs the process to be simulated, and the programs provide the complete NLO answer by combining virtual and real contributions, including merging with parton shower and hadronization effects. For instance, realistic NLO simulations of $W$ + 2 jets [2651] production and $pp \rightarrow H t \bar{t}$ [2652] can be obtained in a completely automated fashion within the Madgraph5-aMC@NLO framework.^38^

The idea of getting the loop amplitude from its integrand (or equivalently from its cuts) can be generalized beyond one-loop [2653–2656], with the important difference that no minimal basis for multi-loop integrals is known. A particularly interesting approach is the multivariate polynomial division [2657, 2658], which generalized OPP to multi-loop integrands, although the field is still in its infancy compared with the full automation achieved at one loop.

#### Supersymmetric gauge theories

Several new results about strongly coupled supersymmetric field theories have been developed in the last several years. The work in [2659] showed how supersymmetric localization can be used to derive exact results in four-dimensional $\mathcal{N}=2$ and $\mathcal{N}=4$ supersymmetric gauge theories. The main point of this important result is that, under certain conditions, supersymmetric field theories can be placed on compact spheres while preserving the action of a supercharge. One can then demonstrate that the full path integral of the theory—even with the insertion of certain supersymmetric operators—reduces to a finite-dimensional integral (matrix model) over configurations preserving the unbroken supercharge. This makes possible the exact, non-perturbative computation of partition functions, Wilson and ’t Hooft loop expectation values, and other observables in several supersymmetric theories in two, three, and four spacetime dimensions.

In parallel, a large class of four-dimensional superconformal field theories with $\mathcal{N}=2$ supersymmetry was discovered [2660], which do not always have a weakly coupled Lagrangian description. These theories can be engineered in string theory by wrapping multiple M5 branes^39^ on Riemann surfaces, and they have interesting mathematical structure and dualities between them. This led to the discovery of the Alday–Galotto–Tachikawa correspondence [2661], which relates partition functions (and other observables) in four-dimensional supersymmetric field theory, to correlation functions in certain two-dimensional CFTs.

#### Conformal field theories

CFTs constitute an important class of quantum field theories. An ambitious long-standing goal is to study conformal field theories by the method of a *conformal bootstrap*. CFTs have the property that all correlation functions can be computed given the spectrum (dimensions and spins of local operators) and operator product expansion (OPE) coefficients. By performing successive OPEs, any correlation function can be computed in terms of these basic CFT data. Requiring the consistency of the OPE expansion in all possible channels leads to an infinite set of equations for the spectrum and OPE coefficients, which are known as the *conformal bootstrap* or *crossing symmetry* equations. These equations are exact and hold beyond perturbation theory. It is, however, difficult to extract useful data from them, as they are an infinite set of equations for an infinite number of variables.

In recent years progress has been made in extracting concrete, rigorous, and universal constraints for higher-dimensional CFTs from the conformal bootstrap equations. This work began with [2662], which demonstrated how—in certain CFTs—the conformal bootstrap can provide bounds for the conformal dimensions of certain operators. For this analysis, the explicit expressions for 4d conformal blocks, first discovered in [2663, 2664], played a crucial role. Subsequently, similar methods have been applied to derive bounds to OPE coefficients, central charges, and other aspects of the spectra of higher-dimensional CFTs. More recently, the conformal bootstrap has been applied towards solving the 3d Ising model [2665]. Interesting new constraints for the spectrum of CFTs can be found by considering the bootstrap equations in the Lorentzian regime [2666, 2667].

In a different direction, a remarkable new result has been the proof of the so-called $a$-theorem [2668]. By design, RG transformations integrate out degrees of freedom. Therefore, if two QFTs are connected by an RG flow, one expects the UV theory to contain more degrees of freedom than the IR theory. In two-dimensional theories this feature is expressed by the Zamolodchikov c-theorem [2669]. In 4d CFTs Cardy [2670] proposed that a certain coefficient $a$ in the trace anomaly be used to count the degrees of freedom, and he conjectured that $a$ would decrease into the IR. Over 20 years later, Komargodski and Schwimmer [2668] proved that $a$ (appropriately defined away from the conformal point) does indeed decrease under RG flow. The now established $a$-theorem strongly suggests the irreversibility of the RG flow and can be used to verify the consistency of conjectured RG-flow relations between different QFTs.

Another interesting development [2671] proved the analog of the Coleman–Mandula theorem for conformal field theories. It has been demonstrated that if a CFT contains a single higher spin conserved charge, then it necessarily has to contain an infinite tower of higher spin conserved currents and additionally, the correlators of those currents have the form of free-field correlators. In subsequent work [2672], it was further proven that weakly broken higher spin symmetry is sufficient to constrain the leading-order three-point functions.

#### 3d CFTs and higher spin symmetry

Significant progress has been made in the study of three-dimensional CFTs. A large class of such theories can be constructed by coupling Chern–Simons gauge theory to matter in various representations of the gauge group. Among them, of special importance are the Aharony–Bergman–Jafferis–Maldacena theories [2673], with matter in the bifundamental of the gauge group $U(N)_k\times U(N)_{-k}$, where $k$ is the Chern–Simons level. These theories describe the low-energy excitations of coincident M2 branes in M-theory. In the large-$N$ limit they are holographically dual to M-theory on AdS$_4\times$S$^7/Z_k$ (or type IIA string theory on AdS$_4\times \mathbb {CP}^3$).

Three-dimensional Chern–Simons theory coupled to matter in the fundamental representation has also attracted attention recently. In the large-$N$ limit these theories exhibit (slightly broken) higher-spin symmetry and interesting dualities between theories with bosons and fermions, a 3d version of “bosonization” [2674–2677]. These CFTs are important from a theoretical point of view, because they are believed to be holographically dual to higher-spin gravity (of Vasiliev type) in AdS$_4$ [2678–2680]. Vasiliev-type gravitational theories [2681], while more complicated than ordinary Einstein gravity in AdS, have vastly fewer fields than string theory and hence provide an example of AdS/CFT of intermediate complexity. Moreover, Chern–Simons fundamental matter theories provide examples of QFT-gravity (and QFT-QFT) dualities without any amount of supersymmetry. Finally, it has been proposed [2682] that there is a triality between a supersymmetric $\mathcal{N}=6$ version of Vasiliev gravity in AdS, the ABJ Chern–Simons-matter theory with gauge group $U(N)_k\times U(M)_{-k}$ and IIA string theory on AdS$_4\times \mathbb {CP}^3$ which might provide an understanding of closed strings in AdS as the flux tubes of (non-Abelian) Vasiliev theory.

Similar relations between higher-spin CFTs and higher-spin gravity have been discovered in lower dimensions. In [2683] an AdS$_3$/CFT$_2$ type of duality has been proposed between the two-dimensional $\mathcal{W}_\mathrm{N}$ minimal model CFTs and Vasiliev gravity in AdS$_3$. This duality is interesting because the boundary theory is exactly solvable and can serve as a useful toy-model for AdS/CFT. Further related developments are reviewed in [2684].

### Conformal symmetry, strongly coupled theories, and new physics

In this section we focus on non-Abelian gauge theories in four dimensions, and discuss the emergence of conformal symmetry when varying the matter content. In the realm of four dimensions, the amount of exact results based on duality arguments is still limited if compared with theories in lower dimensions. It is also generally true that most of the theoretical arguments require exact supersymmetry.

The existence of a conformal window, i.e., a family of theories that develop an attractive infrared fixed point (IRFP) at nonzero coupling and are deconfined with exact chiral symmetry at all couplings, has been long advocated for QCD with many flavors [2685, 2686] and for supersymmetric QCD (SQCD) [2687]. The structure of the perturbative beta function [2688, 2689] for non-Abelian gauge theories without supersymmetry and the Novikov–Shifman–Vainshtein–Zakharov [2690] beta function of SQCD suggest that a conformal window is a general feature of non-Abelian gauge theories with matter content, while its extent and location depend on the gauge group, the number of colors $N$, the number of flavors $N_\mathrm{f}$, and the representation of the gauge group to which they belong. The IRFP moves towards stronger coupling if the number of flavors is decreased, approaching the lower end of the conformal window. This is the reason why only a genuinely non-perturbative study, possibly complemented by the existence of duality relations, can establish the mechanism underlying its emergence or disappearance, its properties, and the differences between realizations with and without supersymmetry. The theory of the conformal window is further discussed in Sect. 9.2.1.

Interestingly, theories just below the conformal window may develop a precursor near-conformal behavior, characterized by a slower change of the running coupling with the energy scale (“walking”) and provide a potentially interesting class of candidates for BSM physics and the EWSB mechanism. The interplay of lattice field theory and AdS/CFT in this context will be considered in Sect. 9.2.2, while strongly coupled BSM candidates and LHC constraints will be more extensively discussed in Sect. 9.3. There, we also review the appealing possibility that conformal symmetry and its spontaneous breaking may play a role up to the Planck scale.

**Fig. 94:**
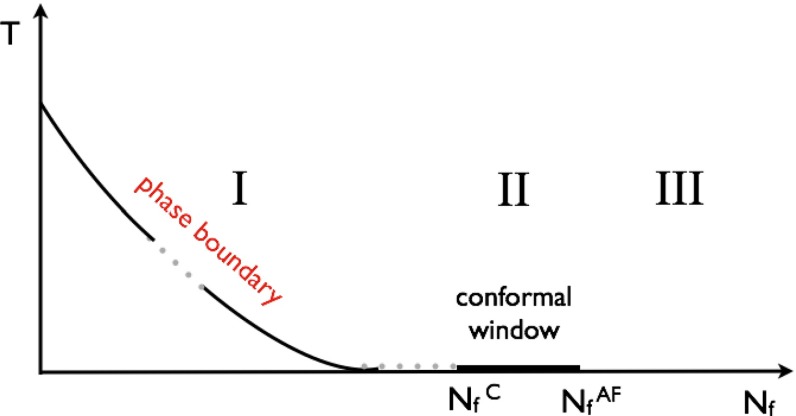
Temperature ($T$) and number of flavors ($N_\mathrm{f}$) phase diagram for a generic non-Abelian gauge theory at zero density. In region I, one or more phase boundaries separate a low-temperature region from a high-temperature region. The nature of the phase boundary and which symmetries identify the two phases, in particular the interplay of confinement and chiral symmetry breaking, depend on the fermion representation and the presence or absence of supersymmetry. Region II identifies the conformal window at zero temperature, for $N_\mathrm{f}^\mathrm{c}<N_\mathrm{f}<N_\mathrm{f}^\mathrm{AF}$, while region III is where the theory is no longer asymptotically free

#### Theory of the conformal window

Figure 94 summarizes the salient features of the phase diagram of non-Abelian gauge theories with massless fermions in the temperature–flavor-number ($T$–$N_\mathrm{f}$) plane. In particular, we identify three regions from left to right: region I describes one or more families of theories below the conformal window, region II identifies the conformal window above a critical flavor number and before the loss of asymptotic freedom $N_\mathrm{f}^\mathrm{c}<N_\mathrm{f}<N_\mathrm{f}^\mathrm{AF}$, while region III is where theories have lost asymptotic (UV) freedom for $N_\mathrm{f}>N_\mathrm{f}^\mathrm{AF}$. Details of region I depend on the way in which deconfinement and chiral symmetry restoration are realized at finite temperature. This realization in turn depends upon the transformation of the fermionic matter representations under the gauge group and the presence or absence of supersymmetry. The simplest realization of region I is provided by QCD, i.e., the case of fermions in the fundamental representation of the $\mathrm{SU}(N)$ gauge group with $N$ colors. In this case, chiral symmetry is broken at zero temperature for any $N_\mathrm{f}<N_\mathrm{f}^\mathrm{c}$, and a chiral phase boundary—a line of thermal chiral phase transitions (or crossovers)—separates the low-temperature chirally broken phase from the high-temperature chirally restored phase.

The region above the chiral phase boundary, at low $N_\mathrm{f}$, describes the strongly coupled quark–gluon plasma (QGP). Just above the phase boundary, QCD and $\mathcal{N}=4$ SYM carry similar features according to the AdS/CFT correspondence. Both predict the QGP to be a strongly coupled, nearly ideal fluid. The two descriptions should depart from each other at higher temperatures, where QCD becomes weakly coupled while $\mathcal{N}=4$ SYM remains strongly coupled. Properties of the QGP are reviewed in Sect. 6 of this document.

In QCD, the presence of a single true order parameter, i.e., the chiral condensate associated with chiral symmetry, suggests that the end point of the finite temperature chiral phase boundary in Fig. 94 should be identified with the lower end of the conformal window. A phase transition would signal its opening at some $N_\mathrm{f}^\mathrm{c}$ (region II in Fig. 94), and chiral symmetry is restored for theories inside the conformal window. Lattice studies [2691] support this scenario, where theories inside the conformal window appear to be chirally symmetric also away from the IRFP. Eventually, chiral symmetry is expected to be broken again at sufficiently strong coupling in the lattice theory. An interesting observation is that chiral symmetry could also be broken by the emergence of a UVFP at strong coupling, signaling the appearance of a new continuum field theory.

While the nature of the phase transition at $N_\mathrm{f}^\mathrm{c}$ is yet to be uncovered, it is natural to expect that the chiral dynamics plays a role in its appearance. In fact, it has been suggested [2685, 2686] that a phase transition of the Berezinskii–Kosterlitz–Thouless (BKT)-type (conformal phase transition) should be expected when the chiral dynamics is taken into account. Such a phase transition would be signaled by a preconformal scaling of chiral observables [2685, 2686, 2692, 2693] just below $N_\mathrm{f}^\mathrm{c}$, known as BKT or Miransky scaling.

Moving to different fermion representations, lattice results [2694] suggest that QCD with fermions in the adjoint representation develops an intermediate phase at finite temperature, for a given $N_\mathrm{f}$ in region I, where the theory is deconfined with broken chiral symmetry. In other words the restoration of chiral symmetry would occur at temperatures higher than the deconfinement temperature, i.e., $T_{\mathrm{ch}} > T_{\mathrm{dec}}$. In this scenario, it is plausible to expect that the two phase boundaries should merge at the lower end of the conformal window, thus $T_{\mathrm{ch}}=T_{\mathrm{dec}}=0$ for $N_\mathrm{f}=N_\mathrm{f}^\mathrm{c}$.

Supersymmetric QCD offers yet another realization of region I, where the dual, free magnetic phase for $N+2<N_\mathrm{f}<N_\mathrm{f}^\mathrm{c}=3N/2$ [2687] implies a confined electric phase, where chiral symmetry is not yet broken [2695]. A better understanding of the interplay of chiral symmetry and confinement in the presence of supersymmetry might also shed light into some aspects of the nonsupersymmetric case [2696, 2697]. Region I of nonsupersymmetric theories is being currently explored on the lattice [2698–2700].

As said before, the conformal window in region II identifies theories with $N_\mathrm{f}^\mathrm{c}<N_\mathrm{f}<N_\mathrm{f}^\mathrm{AF}$; they have a conformal IRFP and are deconfined with exact chiral symmetry at zero temperature. The existence of a conformal window and its properties can thus be established not only by directly probing the IRFP—a delicate task for lattice simulations—but also indirectly through the inspection of chiral observables, confinement indicators, the spectrum of low-lying states, and, more generally, by identifying the symmetry properties of the weak and strong coupling sides of the IRFP. The latter strategy was advocated in [2691], while the running gauge coupling has also been studied in [2701–2703], and strategies to directly probe conformality at the IRFP have been explored in [2704–2706]. Region III is where theories are no longer asymptotically free. The weak coupling beta function is now positive, and the theory is free in the infrared. The emergence of a UVFP at stronger coupling would make these theories interesting.

There are still many questions to be answered. What is the detailed nature of the finite temperature phase boundary, and what is the interplay of confinement and chiral symmetry breaking for theories in region I? What is the nature of the phase transition that opens the conformal window in region II? And what is the fate of the IRFP at $N_\mathrm{f}^\mathrm{c}$? The IRFP coupling can (i) flow to zero, (ii) flow to infinity, (iii) flow to a finite value at which a discontinuity occurs, see, e.g., Ref. [2707], or (iv) merge with a UVFP [2693]. The latter can only be realized if the UVFP is developed at strong coupling for theories inside region II, or simply at its lower-end. The AdS/CFT correspondence can in principle be a useful and complementary tool to explore these scenarios, for CFTs in four or lower dimensions. Preliminary attempts can be found in [2708–2712].

#### Lattice, AdS/CFT, and the electroweak symmetry breaking

Lattice studies of non-Abelian gauge theories just below the conformal window aim to establish or exclude the existence of a preconformal behavior, characterized by an almost zero beta function (to which we have referred previously as the walking regime) and a preconformal scaling of the finite temperature phase boundary and the chiral observables. These theories are expected to be rather strongly coupled, confining in a broad sense and have chiral symmetry spontaneously broken at zero temperature. Depending on their specific matter content (Goldstone bosons and resonances), they may be viable candidates for EWSB and BSM physics at the (multi) TeV scale.

The pattern of color $N$ and flavor $N_\mathrm{f}$ dependence of their beta functions is sufficient to infer that, for fixed $N$, the conformal window shifts to lower $N_\mathrm{f}$ and shrinks when increasing the Casimir of the fermion representation. Furthermore, lowering $N$ is qualitatively equivalent to increasing $N_\mathrm{f}$. Hence, a preconformal behavior with minimal fermionic content could be realized by gauge groups with $N=2$ or 3 and Dirac fermions in representations higher than the fundamental (adjoint, two-index symmetric and two-index antisymmetric), or mixed Weyl and Dirac fermions in the fundamental and nonfundamental representations; for example, the conformal window of $\mathrm{SU}(2)$ with adjoint fermions is expected to open at about $N_\mathrm{f}=2$. This theory and other variations are extensively studied on the lattice, see, e.g., Ref. [2713–2716]. The conformal window for $\mathrm{SU}(3)$ with fermions in the fundamental representation is, in contrast, expected to open in the surroundings of $N_\mathrm{f}=12$, and most results suggest the range between $N_\mathrm{f}=8$ and $N_\mathrm{f}=12$ [2691, 2702, 2717–2719]. This theory offers an optimal playground for the theoretical understanding of the emergence of conformality and its connection with QCD and QGP physics. Notice also that a preconformal regime with a lower fermionic content in the fundamental representation can be obtained by lowering the color content to $N=2$.

It is also important to observe that the most traditional lattice strategies, well tested and optimized in the context of QCD, can be far from optimal when studying the theory inside or close to the conformal window and at strong coupling. This is due to the different symmetry patterns and structure of the beta function for QCD as compared to theories inside the conformal window, and the fact that many optimization methods for lattice QCD have been devised to work close to the continuum limit, at rather weak coupling. It has recently been shown [2720–2723] how the Symanzik improvement program and its generalizations inherited from QCD can lead to exotic phases, genuine lattice artifacts, when used in the study of these systems at strong coupling. The same conclusions may be generalized to the lattice study of strongly coupled condensed matter systems such as graphene [2722]; the latter is a QED system with a chiral symmetry breaking transition at strong coupling, analogous in many respects to theories inside the conformal window in the QED-like region at the strong-coupling side of the IRFP. For reviews and a more complete list of references to recent work see, e.g., Refs. [2724–2727].

The genuinely non-perturbative nature of the lattice formulation for theories inside or just below the conformal window allows, in principle, exploring all salient aspects of their dynamics, in particular the mass ratio of the vector and scalar low-lying states, their first excitations, the pseudo-Goldstone boson decay constant, and the anomalous dimension of the fermion mass operator at the would-be IRFP. The relevance of higher-dimensional operators, such as four-fermion operators, can also be explored, as well as the Yukawa interaction with a scalar field and/or the addition of a dilaton. By varying the details of the interaction Lagrangian and of a Higgs-dilaton potential, one can explore the non-perturbative regime of an entire class of models, from Higgsless, to composite Higgs and dilaton-Higgs models. Also, the lattice study of Yukawa–Higgs models provides a genuinely non-perturbative information on the stability of the Higgs potential and the UV safety of the SM [2728–2731].

Exploiting the AdS/CFT correspondence beyond $\mathcal{N}=4$ SYM in four spacetime dimensions is not a straightforward task. The first steps in this direction aimed to find the AdS realizations that are approximately dual to $\mathcal{N}=1$ SYM [2708], or to SQCD with $N_\mathrm{f}$ dynamical flavors in the fundamental representation [2709–2711]. The beta functions of the approximately dual gauge theories can also be studied [2712], in order to explore possible realizations of the conformal window and the relevance of Kaluza–Klein excitations. The latter do not decouple in general, and give rise to higher-dimensional operators in the dual gauge theory. While still in their infancy, these studies may provide useful insights into the role of supersymmetry for the emergence of conformality and the interplay of chiral symmetry and confinement. Leaving aside AdS/CFT and supersymmetry, a recent attempt to derive the large-$N$ Yang–Mills beta function and the glueball spectrum from first principles [2732, 2733] may finally help to clarify the relevant differences between supersymmetric and nonsupersymmetric theories, and eventually suggest a new class of dualities for nonsupersymmetric gauge theories.

### Electroweak symmetry breaking

A new Higgs-like boson with mass $M_\mathrm{H} = 125.64 \pm 0.35$ GeV has been discovered at the LHC [1283, 1284, 2734, 2735], with a spin/parity consistent with the SM assignment $J^P=0^+$ [1287, 2736]. Although its properties are not yet precisely measured, it complies with the expected behavior, and therefore it is a very compelling candidate to be the SM Higgs [2737]. An obvious question to address is the extent to which alternative scenarios of EWSB remain viable. In particular, what are the implications for strongly coupled models in which the electroweak symmetry is broken dynamically? Alternatively, can a minimally extended SM be a valid theory up to the Planck scale?

#### Strongly coupled scenarios for EWSB 

Usually, strongly coupled theories do not contain a fundamental Higgs field, bringing instead resonances of different types as in QCD. For instance, Technicolor [2738–2740], the most studied strongly coupled model, introduces an asymptotically free QCD replica at TeV energies which breaks the electroweak symmetry in the infrared, in a similar way as chiral symmetry is broken in QCD. This gives rise to the appearance of a tower of heavy resonances in the scattering amplitudes. Other models consider the possibility that the ultraviolet theory remains close to a strongly interacting conformal fixed point over a wide range of energies (Walking Technicolor) [2741–2744]; recent work in this direction incorporates conformal field theory techniques (Conformal Technicolor) [2662, 2745, 2746]. Strongly coupled models in warped [2747] or deconstructed [2748–2750] extra dimensions [2751–2767] have been also investigated.

The recently discovered scalar boson could indeed be a first experimental signal of a new strongly interacting sector: the lightest state of a large variety of new resonances of different types. Among the many possibilities, the relatively light mass of the discovered Higgs candidate has boosted the interest [2768–2770] in strongly coupled scenarios with a composite pseudo-Goldstone Higgs boson [2771–2776], where the Higgs mass is protected by an approximate global symmetry and is only generated via quantum effects. Another possibility would be to interpret the Higgs-like scalar as a dilaton, the pseudo-Goldstone boson associated with the spontaneous breaking of scale (conformal) invariance [2777–2782].

In the absence of direct evidence of a particular ultraviolet completion, one should investigate the present phenomenological constraints, independently of any specific implementation of the EWSB. The precision electroweak data confirm the $\mathrm{SU}(2)_L\times \mathrm{SU}(2)_R\rightarrow \mathrm{SU}(2)_{L+R}$ pattern of symmetry breaking, giving rise to three Goldstone bosons which, in the unitary gauge, become the longitudinal polarizations of the gauge bosons. When the $U(1)_Y$ coupling $g'$ is neglected, the electroweak Goldstone dynamics is described at low energies by the same Lagrangian as the QCD pions, replacing the pion decay constant by the EWSB scale $v=(\sqrt{2}\, G_\mathrm{F})^{-1/2} = 246\,$GeV [2783]. Contrary to the SM, in strongly coupled scenarios the symmetry is nonlinearly realized.

The dynamics of Goldstones and massive resonance states can be analyzed in a generic way by using an effective Lagrangian based on a $\mathrm{SU}(2)_L\times \mathrm{SU}(2)_R$ symmetry, spontaneously broken to the diagonal subgroup $\mathrm{SU}(2)_{L+R}$. The theoretical framework is analogous to the Resonance Chiral Theory description of QCD at GeV energies [2784–2786]. Let us consider a low-energy effective theory containing the SM gauge bosons coupled to the electroweak Goldstone bosons and the light scalar state $S_1$ with mass $m_{S_1} = 126$ GeV, discovered at the LHC, which is assumed to be an $\mathrm{SU}(2)_{L+R}$ singlet. We also include the lightest vector and axial-vector triplet multiplets, $V_{\mu \nu }$ and $A_{\mu \nu }$, with masses $M_V$ and $M_A$, respectively. To lowest order in derivatives and number of resonance fields [2787–2789],

9.1 $$\begin{aligned} \mathcal {L}\;&=\;&\frac{v^2}{4}\,\langle u_{\mu } u^\mu \rangle \,\left( 1 + \frac{2\,\omega }{v}\, S_1\right) + \frac{F_A}{2\sqrt{2}}\, \langle A_{\mu \nu } f^{\mu \nu }_- \rangle \nonumber \\&\, + \frac{F_V}{2\sqrt{2}}\, \langle V_{\mu \nu } f^{\mu \nu }_+ \rangle + \frac{i\, G_V}{2\sqrt{2}}\, \langle V_{\mu \nu } [u^\mu , u^\nu ] \rangle \nonumber \\&\, + \sqrt{2}\, \lambda _1^{SA}\, \partial _{\mu } S_1 \, \langle A^{\mu \nu } u_\nu \rangle \, , \end{aligned}$$

plus the gauge boson and resonance kinetic terms. The electroweak Goldstone fields $\vec \varphi (x)$ are parameterized through the matrix $U=u^2= \exp {\left\{ i \vec {\sigma }\cdot \vec {\varphi } / v \right\} }$, $u^\mu = -i\, u^\dagger D^\mu U\, u^\dagger$, with $D^\mu$ the appropriate gauge-covariant derivative, and $\langle A\rangle$ stands for the trace of the $2\times 2$ matrix $A$. The first term in (9.1) gives the Goldstone Lagrangian, present in the SM, plus the scalar-Goldstone interactions. For $\omega =1$ one recovers the $S_1\rightarrow \varphi \varphi$ vertex of the SM. The $F_V$ and $F_A$ terms incorporate direct couplings of the vector and axial-vector resonances with the gauge fields through $f^{\mu \nu }_\pm = -\frac{g}{2}\, u^\dagger \varvec{\sigma }\,\varvec{W}^{\mu \nu } u \mp \frac{g'}{2} u \sigma _3 B^{\mu \nu } u^\dagger$.

The presence of massive states coupled to the gauge bosons modifies the $Z$ and $W^\pm$ self-energies, which are characterized by the so-called oblique parameters $S$ and $T$ [2790, 2791]. $S$ measures the difference between the off-diagonal $W^3B$ correlator and its SM value, while $T$ parameterizes the difference between the $W^3$ and $W^\pm$ self-energies, after subtracting the SM contribution. To define the SM correlators, one needs a reference value for the SM Higgs mass; taking it at $m_{S_1}= 126$ GeV, the global fit to electroweak precision data gives the constraints $S = 0.03\pm 0.10$ and $T=0.05\pm 0.12$ [1289].

The oblique parameter $S$ receives tree-level contributions from vector and axial-vector exchanges [2790, 2791], while $T$ is identically zero at lowest order (LO):

9.2 $$\begin{aligned} S_{\mathrm {LO}} = 4\pi \left( \frac{F_V^2}{M_V^2}\! -\! \frac{F_A^2}{M_A^2} \right) , \qquad \quad T_{\mathrm {LO}}=0 . \end{aligned}$$

Assuming that weak isospin and parity are good symmetries of the strong dynamics, the $W^3 B$ correlator is proportional to the difference of the vector and axial-vector two-point Green functions. In asymptotically free gauge theories this difference vanishes as $1/s^3$ at $s\rightarrow \infty$ [2792], implying two super-convergent sum rules, known as the first and second Weinberg sum rules (WSRs) [2793]. At LO they give the identities

9.3 $$\begin{aligned} F_{V}^2 - F_{A}^2 = v^2\, , \qquad \quad F_{V}^2 \,M_{V}^2 - F_{A}^2 \, M_{A}^2 = 0\, , \end{aligned}$$

which relate $F_V$ and $F_A$ to the resonance masses, leading to

9.4 $$\begin{aligned} S_{\mathrm {LO}}\; =\; \frac{4\pi v^2}{M_V^2}\, \left( 1 + \frac{M_V^2}{M_A^2} \right) \, . \end{aligned}$$

Since the WSRs also imply $M_A>M_V$, this prediction turns out to be bounded by [2787]

9.5 $$\begin{aligned} \frac{4\pi v^2}{M_V^2} \; < \; S_\mathrm{LO} \; < \; \frac{8 \pi v^2}{M_V^2} \, . \end{aligned}$$

It is likely that the first WSR is also true in gauge theories with non-trivial ultraviolet fixed points [2794, 2795], while the second WSR is questionable in some scenarios. If only the first WSR is considered, but still assuming the hierarchy $M_A>M_V$, the lower bound in (9.5) remains [2787].

The allowed experimental range for $S$ implies that $M_V$ is larger than 1.5 (2.4) TeV at 95 % (68 %) CL. Thus, strongly coupled models of EWSB should have a quite high dynamical mass scale. While this was often considered as an undesirable property, it fits very well with the LHC findings, which are pushing the scale of new physics beyond the TeV region. It also justifies our approximation of only considering the lightest resonance multiplets.

The experimental constraints on $S$ and $T$ depend on the chosen reference value for the SM Higgs mass, which we have taken at $m_{S_1}$. Since the SM Higgs contribution only appears at the one-loop level, there is a scale ambiguity when comparing a LO theoretical result with the experimental measurements, making necessary to consider NLO corrections [2787–2789, 2794, 2796–2800]. Imposing proper short-distance conditions on the vector and axial-vector correlators, the NLO contributions to $S$ from $\varphi \varphi$, $V\varphi$, and $A\varphi$ loops have been evaluated in [2787]. These corrections are small and strengthen the lower bound on the resonance mass scale slightly.

Much more important is the presence of a light scalar resonance with $m_{S_1}= 126$ GeV. Although it does not contribute at LO, there exist sizable $S_1 B$ ($S_1\varphi$) loop contributions to $T$ ($S$). Neglecting the mass-suppressed loop corrections from vector and axial-vector resonances and terms of $\mathcal {O}(m_{S_1}^2/M_{V,A}^2)$, one finds [2788, 2789]

9.6 $$\begin{aligned} T = \frac{3}{16\pi \cos ^2 \theta _W} \bigg [ 1 + \log \frac{m_{S_1}^2}{M_V^2} - \omega ^2 \left( 1 + \log \frac{m_{S_1}^2}{M_A^2} \right) \bigg ] \, . \end{aligned}$$

Enforcing the second WSR, one obtains the additional constraint $\omega = M_V^2/M_A^2$, which requires this coupling to be in the range $0\le \omega \le 1$, and [2788, 2789]

9.7 $$\begin{aligned} S&= \frac{4 \pi v^2}{M_{V}^2} \left( 1+\frac{M_V^2}{M_{A}^2}\right) + \frac{1}{12\pi } \bigg [ \log \frac{M_V^2}{m_{S_1}^2} -\frac{11}{6} \nonumber \\&+\;\frac{M_V^2}{M_A^2}\log \frac{M_A^2}{M_V^2} - \frac{M_V^4}{M_A^4}\, \bigg (\log \frac{M_A^2}{m_{S_1}^2}-\frac{11}{6}\bigg ) \bigg ] \, .\quad \end{aligned}$$

**Fig. 95:**
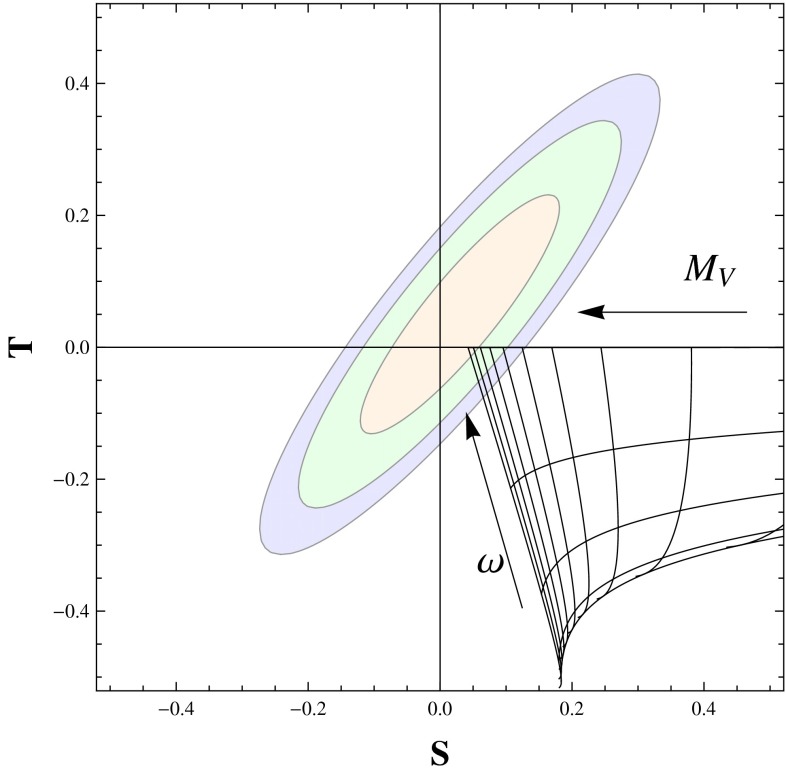
NLO determinations of $S$ and $T$, imposing the two WSRs. The approximately vertical (horizontal) lines correspond to values of $M_V$, from $1.5$ to $6.0$ TeV at intervals of $0.5$ TeV ($\omega = M_V^2/M_A^2$: $0.00, \, 0.25, 0.50, 0.75, 1.00$). The arrows indicate the directions of growing $M_V$ and $\omega$. The ellipses give the experimentally allowed regions at 68 % (*orange*), 95 % (*green*), and 99 % (*blue*) CL [2788]

These NLO predictions are compared with the experimental bounds in Fig. 95, for different values of $M_V$ and $\omega = M_V^2/M_A^2$. The line with $\omega = 1$ ($T=0$) coincides with the LO upper bound in (9.5). This figure demonstrates a very important result in the two-WSR scenario: the precision electroweak data require that the Higgs-like scalar should have a $WW$ coupling very close to the SM one. At 68 % (95 %) CL, one gets $\omega \in [0.97,1]$ ($[0.94,1]$) [2788, 2789], in nice agreement with the present LHC evidence [1283, 1284, 2734, 2735], but much more restrictive. Moreover, the vector and axial-vector states should be very heavy (and quite degenerate); one finds $M_A \approx M_V> 5$ TeV ($4$ TeV) at 68 % (95 %) CL [2788, 2789].

If the second WSR is dropped, one can still obtain a lower bound at NLO (assuming $M_V<M_A$):

9.8 $$\begin{aligned} S \!\ge \! \frac{4 \pi v^2}{M_{V}^2} \!+\! \frac{1}{12\pi } \bigg [ \log \frac{M_V^2}{m_{S_1}^2} \!-\! \frac{11}{6} - \omega ^2 \bigg (\!\log \frac{M_A^2}{m_{S_1}^2}-\frac{17}{6} \!+\! \frac{M_A^2}{M_V^2}\!\bigg ) \bigg ] . \end{aligned}$$

In the limit $\omega \rightarrow 0$, this lower bound reproduces the corresponding result in (9.7), which is excluded by Fig. 95. Thus, a vanishing scalar-Goldstone coupling would be incompatible with the data, independently of whether the second WSR is assumed.

**Fig. 96:**
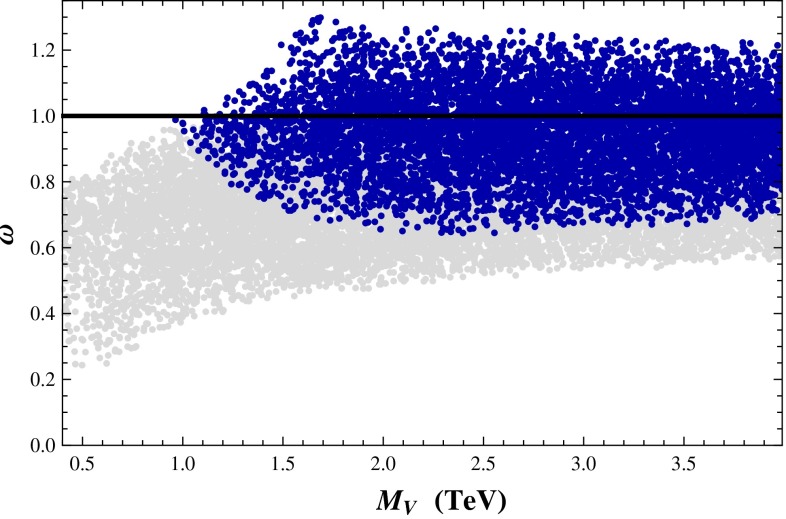
Scatter plot for the 68 % CL region, in the case when only the first WSR is assumed. The *dark blue* and *light gray* regions correspond, respectively, to $0.2<M_V/M_A<1$ and $0.02<M_V/M_A<0.2$ [2788]

Figure 96 shows the allowed 68 % CL region in the space of parameters $M_V$ and $\omega$, varying $M_V/M_A$ between 0 and 1. Values of $\omega$ very different from the SM and/or vector masses below the TeV scale can only be obtained with a large splitting of the vector and axial-vector masses, which looks quite unnatural. In general there is no solution for $\omega >1.3$. Requiring $0.2\, (0.5) <M_V/M_A<1$, leads to $1-\omega <0.4\, (0.16)$ and $M_V > 1\, (1.5)$ TeV [2788, 2789].

The tree-level exchanges of the light Higgs-like boson regulate quite well the high-energy behavior of the longitudinal gauge-boson scattering amplitudes:

9.9 $$\begin{aligned} \mathcal {M}(W_L^+W_L^{-}\rightarrow W_L^+W_L^{-})\,\sim \, (1-\omega ^2)\, u/v^2, \end{aligned}$$

where $u$ is the usual Mandelstam variable. With $\omega \approx 1$, the perturbative unitarity bounds can only be approached at very high energies, where the strongly coupled dynamics will restore the right behavior [2801–2803].

These conclusions are quite generic, only using mild assumptions about the ultraviolet behavior of the underlying strongly coupled theory, and they can be easily adapted to more specific models obeying the $\mathrm{SU}(2)_L\times \mathrm{SU}(2)_R\rightarrow \mathrm{SU}(2)_{L+R}$ pattern of EWSB. For instance, in the $SO(5)/SO(4)$ minimal composite Higgs model [2762, 2763], the $S$ and $T$ constraints are directly given by Fig. 95 with the identification $\omega =\cos \theta \le 1$, where $\theta$ is the $SO(4)$ vacuum angle [2804–2806]. A Higgs-like dilaton, associated with the spontaneous breaking of scale (conformal) invariance at the scale $f_\phi$ [2777–2782], would correspond to $\omega = v/f_\phi$. The experimental constraints on $\omega$ discussed above require $f_\phi \sim v$, making unlikely this light-dilaton scenario.

Thus, strongly coupled electroweak models are allowed by current data provided the resonance mass scale stays above the TeV scale and the light Higgs-like boson has a gauge coupling close to that of the SM. This has obvious implications for future LHC studies, since it leads to a SM-like scenario. A possible way out would be the existence of new light scalar degrees of freedom, sharing the strength of the SM gauge coupling; at available energies, this possibility could result in phenomenological signals similar to perturbative two-Higgs-doublet models [2807].

Future progress requires a thorough investigation of the fermionic sector. The couplings of the Higgs-like scalar with ordinary fermions are not well known yet and could show deviations from the SM Yukawa interactions. Generally, a proper understanding of the pattern of fermion masses and mixings is also missing; in particular, the huge difference between the top mass scale and the small masses of the light quarks or the tiny neutrino ones remains to be explained.

#### Conformal symmetry, the Planck scale, and naturalness

Should the LHC experiments ultimately discover no new particles, beyond the Higgs-like boson at about 126 GeV, then entire families of BSM theories would be excluded or would have to depart from naturalness [2740, 2808–2812] in a substantial way; it would be true for all scenarios that invoke a relevant new energy scale $\Lambda _{\mathrm{EW}}\lesssim \Lambda \ll \Lambda _{\mathrm{Planck}}$, such as most versions of weakly coupled supersymmetry or strongly coupled compositeness. One should, instead, aim to formulate a theoretically viable completion of the SM that does not imply a proliferation of new particles up to scales $\Lambda \lesssim \Lambda _{\mathrm{Planck}}$, possibly embedding gravity. In other words, to which extent is it possible to enhance the symmetries of the SM without enlarging its particle content?

It seems not accidental that a Higgs boson with a mass of about 126 GeV allows for a SM vacuum that is at least metastable, or perhaps stable [2813–2819], with the SM ultraviolet cutoff as high as the Planck scale. A precise determination of the boundary between the metastable and stable vacuum solution for the SM has become especially relevant after the discovery of the Higgs-like boson at the LHC. The full knowledge of the RG coefficients for all the SM parameters (gauge couplings, Yukawa couplings, masses, and Higgs sector parameters), from the weak scale to the Planck scale, is necessary to establish the fate of the SM vacuum. Most of the current predictions [2814–2817] suggest that the SM vacuum is at least metastable. Interestingly, the work in [2819] concludes for a stable solution, accompanied by a first-order phase transition at about  $7\times 10^{16}$ GeV, above which the system is in the unbroken phase, i.e., the Higgs VEV vanishes; in this analysis the phase transition is induced by the zero in the coefficient of the quadratic divergence of the Higgs mass counterterm. It is interesting to explore further the implications of this scenario for inflation and baryogenesis.

All the above mentioned results should be considered as work in progress, since predictions and their accuracy are still affected by theoretical uncertainties (such as higher-order contributions in the perturbative expansion, or inclusion of operators with dimension higher than four), and by the experimental uncertainty on the top quark mass, the running strong coupling $\alpha _\mathrm{s}$, and the Higgs mass itself. Calculations are currently done in perturbation theory, see, e.g., Refs. [2814–2817, 2819, 2820], and on the lattice [2818].

A vacuum that is at least metastable, with a lifetime longer than the age of the Universe, or stable would imply that the SM may be a valid effective field theory up to the Planck scale. It would not contradict the stringent bounds coming from flavor physics, on the contrary it would avoid the long-standing difficulties of most BSM models to produce tiny deviations from the SM predictions in all flavor sectors and for all relevant observables; among the latter are flavor changing neutral current processes, radiative decays such as $b\rightarrow s\gamma$, and CP-violating observables, such as permanent electric dipole moments, see Sect. 5. Ultimately, we would like to answer the first question of all: what is the symmetry, if any, that protects the Higgs mass from running all the way to the Planck mass, or, equivalently, what is the source of the large hierarchy $\Lambda _{\mathrm{EW}}/\Lambda _{\mathrm{Planck}}\simeq 10^{-16}$?

**Fig. 97:**
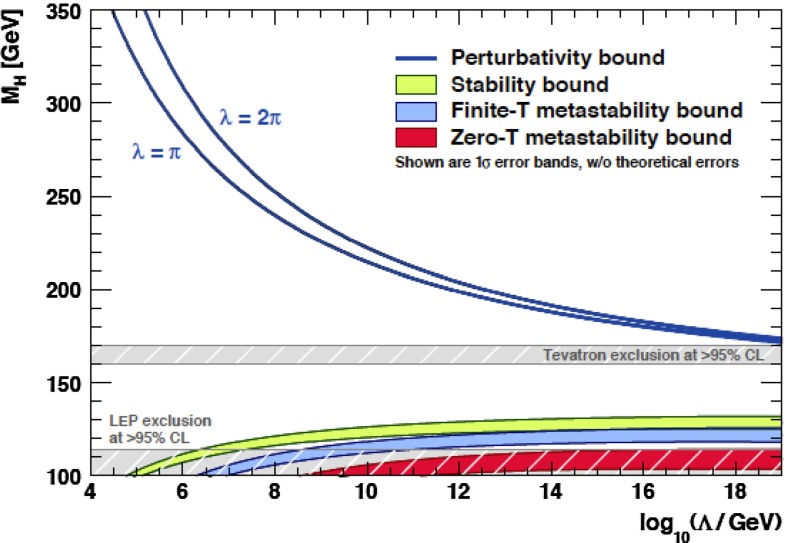
An illustration of the trend of the stability bounds (*lower curves*) and perturbativity bounds (*upper curves*) for the SM vacuum from [2813] as a function of the quartic self-coupling of the Higgs field and the Higgs boson mass. The determination of the boundary between the metastable and stable vacuum solution is work in progress, and depends on the experimental uncertainty on the top quark mass, the running strong coupling $\alpha _\mathrm{s}$, and the Higgs mass itself

The line of thought in [2819] would answer this question without invoking an underlying symmetry beyond the SM gauge group. Instead, it is the RG evolution of the quadratic divergences (treated as physical, for the theory with a finite UV cutoff) to protect the SM from instabilities [2819]. An alternative line of thought is to invoke an underlying symmetry beyond the SM gauge group. In line with ideas put forward more than a decade ago [2821] and ideas that inspired walking technicolor models [2741–2744], one could conceive that scale invariance (and the invariance under the full conformal group) is the symmetry underlying the RG evolution of the SM well above the TeV scale and up to the Planck scale. At the classical level, the SM Lagrangian is scale and conformally invariant, with the exception of the Higgs mass term. To maintain full conformal symmetry at the Lagrangian level, one can generate the Higgs mass through a Higgs-dilaton coupling [2822] and the spontaneous breaking of conformal symmetry [2823, 2824]. The dilaton, which is the Goldstone boson of the spontaneously broken symmetry, remains massless or may acquire a nonzero mass through terms that explicitly break conformal symmetry.

At the quantum level, the SM scale and conformal invariance is explicitly broken by the logarithmic running of the coupling constants, so that the divergence of the dilatation current, which is equal to the trace of the energy-momentum tensor, has the general form

$$\begin{aligned} \partial _{\mu } s^\mu = T_{\mu }^\mu = \sum _i\, \beta _i (\{g\},\{\lambda \} )\cdot O_i^{(d=4)} + {\text{ mass } \text{ terms }} , \end{aligned}$$

with $\beta _i$ the beta function of the SM coupling for the operator $O_i$, $\{g\}$ the set of gauge couplings, and $\{\lambda \}$ the set of scalar couplings. In the absence of mass terms, scale (and conformal) invariance will be restored at RG fixed points, where $\beta _i =0$. Approximate scale invariance, with $\beta _i\simeq 0$, might also be sufficient for the viability of the SM beyond the EWSB scale. Work dating before the discovery of the top quark [2825] pointed to the appealing possibility that SM physics at the weak scale is driven by the presence of infrared pseudo-fixed points for the SM couplings. The relevant observations can be summarized as follows: i) the top Yukawa coupling $g_t$ and the self-interaction Higgs coupling $\lambda$ develop a IRFP in the limit where the electroweak couplings $g,g^\prime$ are neglected with respect to the strong coupling $g_\mathrm{c}$, ii) the running of the light quark masses and charged leptons is unaffected by $g_t$, while the light down quarks receive small corrections from it, iii) the RG running of the ratio $m_b/m_\tau$ is dominated by $g_t$, (iv) the gauge couplings are unaffected by $g_t$ at one-loop order, and v) the CKM mixing angles and phase seem to have a IRFP at zero that is approached very slowly. Detailed studies of the RG equations of the SM and its supersymmetric extensions have followed during the years, essentially without changing the early conclusions. For a review and analysis of the fixed point and manifold structure of the SM see Ref. [2826].

The ultraviolet fate of the SM is not yet established, and the LHC has not yet provided hints of a specific BSM completion close to the TeV scale. In the context of RG studies, the reduction of parameters program introduced in [2827] may be resurrected and provide insights into possible ultraviolet behaviors in light of the most recent experimental findings. Recall that in the matter sector with $g=g^\prime =0$, the top and Higgs couplings in the top-Higgs–$g_\mathrm{c}$ subsector share asymptotic freedom and have a IRFP. Even if the SM cannot be taken to the limit $\mu \rightarrow \infty$ due to the Landau pole of $g^\prime$, nor to the limit $\mu \rightarrow 0$ due to the confinement of strong interactions, it might well be that the underlying conformal symmetry in one or more sectors of the SM is enough to drive its evolution from the electroweak to the Planck scale.

Conformal symmetry would also be able to avoid the source of the gauge hierarchy problem, since it can protect the mass of the Higgs boson from additive quantum corrections of the order of the ultraviolet cutoff of the theory. As an alternative to the most familiar SM extensions with and without supersymmetry, one can invoke conformal invariance at the quantum level and its spontaneous breaking at the Planck scale, see, e.g., Ref. [2823]. In the context of quantum gravity, the authors of [2824] conjectured that it is always possible to render a theory conformally invariant at the quantum level (at least perturbatively), if its action is conformally invariant in any $d$ spacetime dimensions—obtained via dilaton couplings—and if conformal symmetry is only spontaneously broken. In other words, there would exist conformally invariant counterterms to all orders in perturbation theory. Within the scalar sector of the SM, it was recently shown [2823] that a “Scale-Invariant” (SI) prescription does exist for which i) the theory is conformally invariant at the quantum level to all orders in perturbation theory, ii) it reproduces the low-energy running of the coupling constants, iii) it embeds unimodular gravity, and (iv) it protects the mass of the Higgs boson from additive ultraviolet corrections, i.e., $\delta m_\mathrm{H}^2 \propto m_\mathrm{H}^2$ and not $\Lambda _{\mathrm{Planck}}^2$.

It seems worthwhile to explore further the consequences of this program for the Yukawa and gauge sectors of the SM, taking as a reference starting point the spontaneously broken conformal symmetry at the Planck scale. It remains to be seen if the resulting theory is renormalizable and, most importantly, unitary, and to be established how unique the prescription is that both ensures conformality at the quantum level and reproduces the low-energy running of the SM couplings. In view of the most recent LHC findings, the scenario of a minimally extended SM up to the Planck scale with conformal invariance as an underlying symmetry, remains an appealing possibility. The next round of LHC data will hopefully provide further hints into a preferred high-energy completion of the SM.

### Methods from high-energy physics for strongly coupled, condensed matter systems

The investigation of QCD at low energies, a prototypical example of a strongly coupled quantum field theory, has lead to the development of a number of methods for describing strongly coupled theories also in other areas of physics. The example studied here is condensed matter physics, where methods developed for QCD are applied to strongly coupled theories of relevance for the study of systems such as graphene and high-$T_\mathrm{c}$ superconductors. Both lattice gauge theory and gauge–gravity duality methods have been applied to condensed matter systems. While lattice gauge theory is an established method for studying QCD at low energies, gauge–gravity duality was developed more recently as a generalization of the AdS/CFT correspondence of string theory. It has proved very useful in studies of transport processes and the calculation of spectral functions of the quark–gluon plasma and for QCD-like theories at high density, reviewed in Sect. 6 of this document.

As examples of lattice gauge theory and gauge–gravity duality applications to condensed matter physics, we review lattice gauge theory results for the conductivity in graphene as function of the coupling strength, as well as gauge–gravity duality results for the Green functions and conductivities in non-Fermi liquids and superconductors. These methods may be applied more generally to the description of strongly coupled systems in condensed matter physics, for which traditional methods are scarce. They may also be used to predict new phases of matter.

#### Lattice gauge theory results

Graphene is a material which displays a relativistic dispersion relation. Near the Fermi-Dirac points, the charge carriers display an energy spectrum similar to the one of free $2+1$-dimensional massless Dirac fermions. This leads to unusual transport properties which have recently been investigated using lattice gauge theory [2828–2830]. The lattice study of graphene was initiated in [2831], where evidence for a second-order semimetal–insulator transition was found, which is associated with spontaneous chiral symmetry breaking and the opening of a gap in the energy spectrum.

As in [2831], the starting point of [2828] is a $3+1$-dimensional Abelian lattice gauge field coupled to $2+1$-dimensional staggered lattice fermions. The conductivity calculated as a function of the inverse lattice gauge coupling $\beta$ is given by

**Fig. 98:**
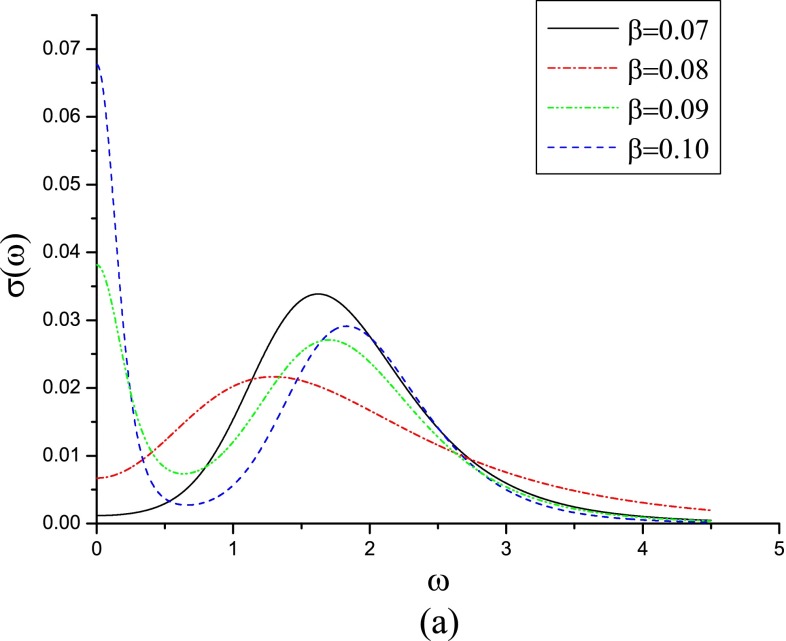
AC conductivity by varying the frequency $\omega$ from the lattice study in [2828] for different values of the inverse coupling $\beta$ in the strong coupling regime

9.10 $$\begin{aligned} \beta \equiv \frac{1}{g^2} = \frac{v_\mathrm{F}}{4 \pi e^2} \frac{\epsilon +1}{2} \, , \end{aligned}$$

where $\epsilon$ is the dielectric permittivity and $v_\mathrm{F}$ the Fermi velocity. It is found that at large values of the coupling $g$, a fermion condensate $\langle \bar{\psi }\psi \rangle$ forms. Simultaneously, the DC conductivity is smaller in the strong coupling regime ($g=4.5$) as compared to the weak coupling regime ($g < 3.5$) by three orders of magnitude. At small values of $\beta$, the AC conductivity as calculated from linear response theory shows the behavior displayed in Fig. 98. In the opposite limit of vanishing interaction (large $\beta$), the AC conductivity should develop a $\delta (\omega )$ contribution due to translational invariance from the absence of scattering. When the interaction is increased, thus for decreasing $\beta$ in Fig. 98, the peak becomes broader. The second peak in Fig. 98 is expected to correspond to the optical frequency range for graphene. These results have been obtained using the maximum entropy method, while a more refined analysis based on a tight-binding model can be found in [2829].

An alternative QCD-inspired strong-coupling approach to study graphene is to use the Schwinger–Dyson equations. The dynamical gap generation by long-range Coulomb interactions in suspended graphene has been investigated with this approach in [2832].

#### Gauge–gravity duality results

Generalizations of the AdS/CFT correspondence [2604], referred to as *gauge–gravity duality*, are naturally suited for describing strongly coupled systems. Gauge–gravity duality is a conjecture which states that strongly coupled $\mathrm{SU}(N)$ field theories with $N \rightarrow \infty$ in $d$ dimensions are mapped to weakly coupled gravity theories in $d+1$ dimensions. The two theories share the global symmetries and the number of degrees of freedom. Supersymmetry as well as conformal symmetry may be completely broken within gauge–gravity duality by considering more complicated metrics than the original anti-de Sitter space, and RG flows may be described in which the additional coordinate corresponds to the energy scale. Several non-trivial examples within QCD support the gauge–gravity duality conjecture, such as the result for the shear viscosity over entropy ratio [1864], results for jet quenching [2048], as well as for chiral symmetry breaking and the $\rho$ meson mass as function of the $\pi$ meson mass squared at large $N$ [2245] (see Sect. 6).

Of course, the microscopic degrees of freedom in a condensed matter system are very different from those described by a non-Abelian gauge theory at large $N$. Nevertheless, the idea is to make use of *universality* and to consider systems at second order phase transitions or, more generally, at renormalization group fixed points, where the microscopic details may not be important. A prototype example for this scenario are *quantum phase transitions*, i.e., phase transitions at zero temperature which are induced by quantum rather than thermal fluctuations [2833–2835]. These transitions generically appear when varying a parameter or coupling which is not necessarily small.

In many cases, the study of models relevant to condensed matter physics involves the introduction of a finite charge density in addition to finite temperature. This applies, for instance, to Fermi surfaces or condensation processes. In the gauge–gravity duality context, this is obtained in a natural way by considering charged black holes, the Reissner–Nordström black holes. Their gravity action involves additional gauge fields,

9.11 $$\begin{aligned} \mathcal{S} = \int \! \mathrm {d}^{d+1} x \, \sqrt{-g} \left[ \frac{1}{2 \kappa ^2} \left( R - 2 \Lambda \right) - \frac{1}{4 e^2} F^{m n}F_{m n} \right] .\nonumber \\ \end{aligned}$$

Here, $\kappa ^2$ is the gravitational constant in $d+1$ dimensions, $R$ is the Ricci scalar for the metric $g_{m n}$ with determinant $g$, $\Lambda$ is the negative cosmological constant associated with anti-de Sitter space, and $F_{mn}$ is the field strength for a $U(1)$ gauge field $A_m$ on the gravity side. According to the prescriptions of the AdS/CFT correspondence, this gauge field $A_m$ couples to a conserved global $U(1)$ current in the dual $\mathrm{SU}(N)$ gauge theory, for which it acts as a source,

9.12 $$\begin{aligned} \langle J_{\mu } \rangle = \frac{\delta W}{\delta A^\mu } \, , \end{aligned}$$

with $W$ the generating functional of connected Green’s functions. Similarly, the metric of the curved space is the source for the energy-momentum tensor in the dual field theory. A chemical potential and finite charge density are obtained from a non-trivial profile for the time component of the gauge field in (9.11). Within this approach, standard thermodynamic quantities such as the free energy and the entropy may be calculated. An important observable characterizing the properties of condensed matter systems, and already discussed in Sect. 9.4.1 in the context of lattice studies, is the frequency-dependent conductivity. This can be calculated in a straightforward way using gauge–gravity duality techniques. Below, we discuss examples for results obtained using this approach.

In several holographic models, instabilities may lead to new ground states with lower free energy. This includes models with properties of superfluids and superconductors [2836, 2837]. In addition to condensed matter physics, such new ground states occur also in models describing the quark–gluon plasma at finite isospin density and predict the frictionless motion of mesons through the plasma [2838, 2839]. In some cases, the new ground state is characterized by a spatially modulated condensate [2840–2843]. These findings have analogs also within QCD itself. For instance, an external magnetic field leads to a spatially modulated $\rho$ meson condensate [2844, 2845], similar to earlier results for Yang–Mills and electroweak fields [2846, 2847].

**Fig. 99:**
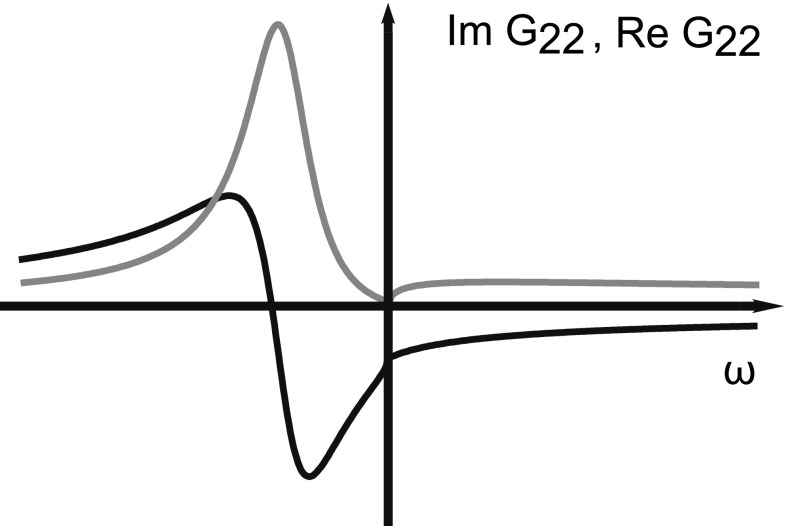
Typical frequency dependence of the real part (*black*) and imaginary part (*gray*) of the fermionic retarded Green function calculated from gauge–gravity duality. We display the 2–2-component in spin space

A further important aspect of condensed matter applications is the study of fermions in strongly coupled systems using gauge–gravity duality [2848, 2849]. The standard well-understood approach for describing fermions in weakly coupled systems is Landau-Fermi liquid theory. These systems have a Fermi surface, and the low-energy degrees of freedom are quasi-particle excitations around the Fermi surface. However, many systems have been observed in experiments which do not exhibit Landau-Fermi liquid behavior. Although they have a Fermi surface, their low-energy degrees of freedom do not correspond to weakly coupled quasi-particles. Nevertheless, the Fermi surface contains essential information about the physical properties also of strongly coupled systems. For instance for high-$T_\mathrm{c}$ superconductors, it reveals the $d$-wave symmetry structure. Gauge–gravity duality provides means for calculating Fermi surfaces and spectral functions for strongly coupled systems [2850]. An example for the real and imaginary parts of the retarded Green function is shown in Fig. 99. This result is obtained from the Reissner–Nordström black hole discussed above and corresponds to the Fermi surface of a strongly coupled non-Fermi liquid which is difficult to obtain using standard approaches. Note that for fermions, the Green function is a $2 \times 2$ matrix in spin space.

**Fig. 100:**
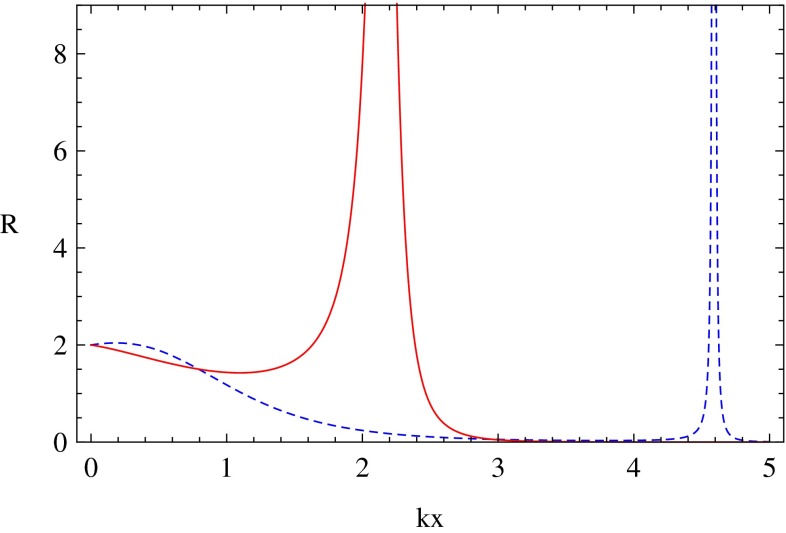
Spectral function from [2851] as function of the momentum $$k_x/ \pi T$$ from a gauge–gravity dual model showing non-Fermi liquid behavior. The two curves correspond to different components $${R}_{11} $$ (*solid red*) and $${R}_{22} $$ (*dashed blue*) of the fermion matrix in spin space, related by $${R}_{11} (k_x) = {R}_{22} (-k_x)$$

Within the gauge–gravity duality framework, an approach giving control over the microscopic degrees of freedom of the quantum field theory involved is to calculate Fermi surfaces for fermionic supergravity fields dual to composite gauge-invariant fermionic operators in the dual field theory [2851]. This requires starting from a ten-dimensional gravity action involving an internal manifold in addition to the asymptotically anti-de Sitter space. Due to the strong coupling, the resulting systems may be of marginal or non-Fermi liquid type. An example is shown in Fig. 100. The predicted dispersion relation and momentum dependence of the spectral function read [2848, 2851]

9.13 $$\begin{aligned} \omega - \omega _\mathrm{f}&\sim \left( k - k_\mathrm{f}\right) ^z \, \end{aligned}$$

9.14 $$\begin{aligned} {R_{ii}}&\sim \left( k - k_\mathrm{f} \right) ^{-\alpha }, \quad i=1,2, \end{aligned}$$

with

9.15 $$\begin{aligned} z = 1.00 \pm 0.01, \qquad \alpha = 2.0 \pm 0.1 \, . \end{aligned}$$

This result deviates substantially from the Landau-Fermi liquid theory, where $z = \alpha =1$. More recently, progress has been made towards holographically calculating the Fermi surfaces for the elementary fermions present in the dual field theory [2852].

For gauge–gravity dual models of superconductivity and superfluidity, the conductivity as obtained from the current-current correlator

**Fig. 101:**
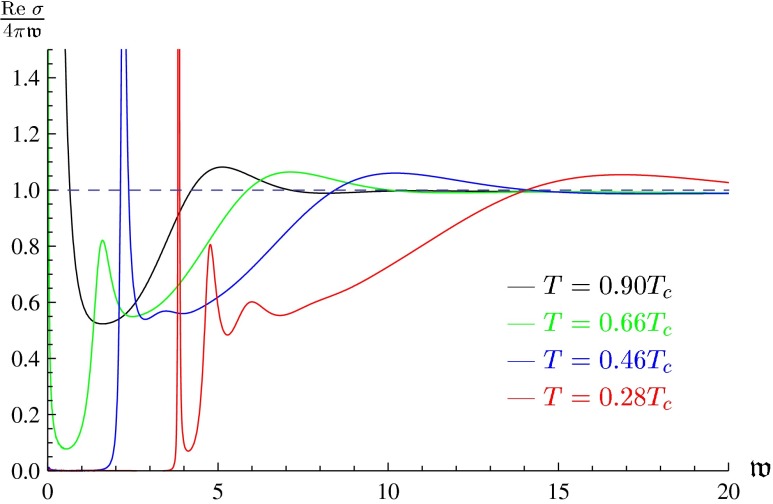
Frequency-dependent conductivity for the gauge–gravity superfluid from [2838]. The *horizontal axis* corresponds to the reduced frequency $\omega /(2 \pi T)$. This relativistic model involves a finite isospin density and the new ground state corresponds to a $\rho$ meson condensate. At low frequencies, a gap develops when lowering the temperature. The peaks at higher frequencies above the gap correspond to higher excited modes (similar to the $\rho ^*$) in this strongly coupled system

9.16 $$\begin{aligned} \sigma _{ij} (\omega ) = \frac{i}{\omega } G^R_{ij} (\omega ) \, , \qquad i,j \in \{1,2 \} , \end{aligned}$$

displays a gap as function of the frequency as expected. For the model of [2838, 2839], which corresponds to a relativistic superfluid at finite isospin density, this is shown in Fig. 101.

Since condensed matter systems are generically nonrelativistic, it is useful to consider extensions of gauge–gravity duality to spaces which share nonrelativistic symmetries [2853, 2854]. Some of these spaces have the additional advantage of naturally providing a zero ground state entropy. Moreover, in addition to the thermodynamic entropy, the quantum-mechanical entanglement entropy may also be realized within the gauge–gravity duality [2855], with significant consequences for the models considered. Generally, the entanglement entropy provides an order parameter, for instance, for topologically ordered states.

The examples given show that both lattice gauge theory and gauge–gravity duality have useful applications to strongly coupled systems also within condensed matter physics. Further new developments along this recent line of research are expected in the near future.

### Summary and future prospects

The study of strongly coupled systems, from particle to condensed matter physics, has recently acquired new prospects and directions. One important aspect of these developments, and partly a revival of ideas sketched in the past, is the realization of the utility of conformal symmetry because many properties of systems in Nature can be described in terms of small deviations from the conformal point. This happens to be particularly useful when trying to solve field theories in their non-perturbative regime, i.e., in strongly coupled systems.

As reviewed in Sect. 9.1, many exact methods have been recently refined to describe QFTs in the large $N$ limit, often inspired by string theory and its lower-dimensional realizations. The AdS/CFT correspondence, and, more generally, gauge–gravity dualities have become an inspiring tool for effective field theory realizations of strongly coupled systems in four or lower spacetime dimensions, by identifying their dual string theory realization. However, it will remain difficult to extend the use of duality arguments beyond the large $N$ limit, and to predict from first principles the size of deviations from the large $N$ and the conformal limits in this framework. Conformal bootstrap methods offer a powerful alternative, fully based on field theory arguments. Still it remains true that in some cases—properties of QGP, conductivities in graphene and superconductors—the predictions based on the gauge–gravity duality turn out to be promisingly close to the experimental results, as discussed in Sect. 9.4. Lattice field theory computations remain, as of today, the only genuinely non-perturbative description of these systems that is a priori able to provide the complete answer, from strong to weak coupling, once the continuum limit is reached.

As reviewed in Sect. 9.2, the combination of lattice computations and analytic field theoretical methods is being especially successful in uncovering the physics of the conformal window and, more generally, the approach to conformal symmetry in non-Abelian gauge theories with matter content, with and without supersymmetry. Strongly coupled theories close to the conformal window provide interesting candidates for BSM physics. A wide class of viable BSM theories have been discussed in Sects. 9.2 and 9.3. In particular, we have considered theories with a strongly coupled new sector, invoking compositeness at the multi-TeV scale with or without conformality, and minimal SM extensions where conformal symmetry is invoked at the Planck scale. Weakly coupled supersymmetric extensions of the SM have not been discussed here, and, except for the maximally constrained minimal supersymmetric SM (cMSSM), they remain a viable alternative to the mentioned scenarios.

All these attempts aim to accommodate a Higgs boson of 126 GeV and the absence of significant deviations from the SM, as in accordance with the LHC observations collected up to now. The next run of the LHC experiments will either confirm the validity of the SM by pushing all exclusion bounds to higher energies, or, in the most striking case, will find direct evidence of a new sector—be it, perhaps, a resonance of a composite strongly coupled extension, additional scalar(s), or supersymmetric partner(s). While awaiting further experimental signatures, the task of particle theorists is to rethink their models in light of the recent Higgs boson discovery and broaden their scope by exploring implications for cosmology and a possible unification with gravity.

Ultimately, the high-energy completion of the SM ought account for anything that is not yet embedded in it, i.e., neutrino masses and oscillations, baryo- and leptogenesis, dark matter and dark energy—or anything that identifies with that $27$ and $68~\%$, respectively, of our Universe beyond a tiny $5~\%$ [2856, 2857] of visible baryonic matter. In other words, it should account for the evolution of the Universe once gravity is embedded in the theory, and it ought to explain any possible deviation from the SM eventually observed at the LHC.

## Appendix: Acronyms

**Table:**

	**Accelerators**
AGS	Alternating Gradient Synchrotron
	http://www.bnl.gov/rhic/ags.asp
DA$\Phi$NE	Double Annular $\Phi$ Factory for Nice Experiments
	http://www.lnf.infn.it/acceleratori/
ELSA	ELectron Stretcher Accelerator
	http://www-elsa.physik.uni-bonn.de/elsa-facility_en.html
HERA	Hadron-Electron Ring Accelerator
	http://adweb.desy.de/mpy/hera/
LEP	Large Electron-Positron collider
	http://home.web.cern.ch/about/accelerators/large-electron-positron-collider
LHC	Large Hadron Collider
	http://home.web.cern.ch/topics/large-hadron-collider
MESA	Mainz Energy-Recovering Superconducting Accelerator
	http://www.prisma.uni-mainz.de/mesa.php
NICA	Nuclotron-based Ion Collider fAcility
	http://nica.jinr.ru/
RHIC	Relativistic Heavy Ion Collider
	http://www.bnl.gov/rhic/
SPS	Super Proton Synchrotron
	http://home.web.cern.ch/about/accelerators/super-proton-synchrotron
Tevatron	http://www.fnal.gov/pub/tevatron/

**Table:**

	**Experiments**
ACME	Advanced Cold Molecule Electron EDM
	http://laserstorm.harvard.edu/edm/
ACORN	A CORrelation in Neutron decay
	http://www.ncnr.nist.gov/expansion/individual_instruments/aCORN041911.html
ALEPH	Apparatus for LEP Physics at CERN
	http://home.web.cern.ch/about/experiments/aleph

**Table:**

ALICE	A Large Ion Collider Experiment
	http://aliceinfo.cern.ch/
ATLAS	A Toroidal LHC ApparatuS
	http://atlas.ch
BaBar	http://www-public.slac.stanford.edu/babar/
Belle	http://belle.kek.jp/
BES III	Beijing Spectrometer
	bes3.ihep.ac.cn/
BRAHMS	Broad RAnge Hadron Magnetic Spectrometers Experiment at RHIC
	http://www4.rcf.bnl.gov/brahms/WWW/brahms.html
CB	Crystal Ball (MAMI)
	http://wwwa2.kph.uni-mainz.de/internalpages/detectors-and-setup/cb-mami.html
CMD-3	Cryogenic Magnetic Detector
	cmd.inp.nsk.su/cmd3
CMS	Compact Muon Solenoid
	http://cern.ch/cms
COMPASS	COmmon Muon and Proton Apparatus for Structure and Spectroscopy
	http://www.compass.cern.ch/
CPLEAR	http://cplear.web.cern.ch/cplear/welcome.html
DØ	after its location on the Tevatron ring
	http://www-d0.fnal.gov/
DELPHI	DEtector with Lepton, Photon and Hadron Identification
	http://www.cern.ch/delphi
DIRAC	DImeson Relativistic Atom Complex
	dirac.web.cern.ch/DIRAC/
DISTO	Dubna-Indiana-Saclay-TOrino
	http://oldsite.to.infn.it/activities/experiments/disto/disto_overview.html
E158	E158 experiment (SLAC) A precision measurement of the Weak Mixing Angle in Moeller Scattering
	http://www.slac.stanford.edu/exp/e158/
E246	E246 experiment (KEK)
E549	E549 experiment (KEK)
E609	E609 experiment (Fermilab)
E653	E653 experiment (Fermilab)
E665	E665 experiment (Fermilab) Muon spectrometer
	http://www.nuhep.northwestern.edu/~schellma/e665/
E791	E791 experiment (Fermilab)
	http://ppd.fnal.gov/experiments/e791/welcome.html
E852	A Search for Exotic Mesons
	http://hadron.physics.fsu.edu/~e852/
E862	E862 experiment, Antihydrogen at Fermilab
	http://ppd.fnal.gov/experiments/hbar/
E989	E989 experiment (Fermilab)

**Table:**

emiT	A search for Time-reversal Symmetry Violation in Polarized Neutron Beta Decay
	http://ewiserver.npl.washington.edu/emit
FINUDA	Fisica NUcleare a DAFNE
	http://www.lnf.infn.it/esperimenti/finuda/finuda.html
FOCUS	http://www-focus.fnal.gov
FOPI	4$\pi$
	http://www.gsi.de/en/work/research/cbmnqm/fopi.htm
GRAAL	GRenoble Anneau Accelerateur Laser
	http://graal.ens-lyon.fr/
HADES	High Acceptance DiElectron Spectrometer
	http://www-hades.gsi.de/
HAPPEX	Hall A Precision Parity EXperiment
	http://hallaweb.jlab.org/experiment/HAPPEX
HERMES	http://www-hermes.desy.de/
H1	H1 detector (HERA)
	www.h1.desy.de/
JADE	JApan, Deutschland, and England
	https://wwwjade.mpp.mpg.de/
KaoS	Kaon Spectrometer
	http://www-aix.gsi.de/~kaos/html/kaoshome.html
KEDR	kedr.inp.nsk.su/
KLOE	K LOng Experiment
	http://www.lnf.infn.it/kloe/
LHCb	Large Hadron Collider beauty
	http://lhcb.web.cern.ch/lhcb/
MINER$\nu$A	Main Injector Experiment for $\nu$-A
	http://minerva.fnal.gov/
MiniBoone	BOOster Neutrino Experiment
	http://www-boone.fnal.gov/
MOLLER	after Møller scattering
	http://hallaweb.jlab.org/12GeV/Moller/
MuLan	MUON Lifetime ANalysis
	http://www.npl.uiuc.edu/exp/mulan//
MUSE	The MUon proton Scattering Experiment
	http://www.physics.rutgers.edu/~rgilman/elasticmup/
Muon $g-2$	http://www.g-2.bnl.gov/
NA10	NA10 experiment (CERN)
	http://greybook.cern.ch/programmes/experiments/NA10.html/
NA45	NA45 experiment (CERN)
	http://greybook.cern.ch/programmes/experiments/NA45.html
NA48	NA48 experiment (CERN) CP violation
	http://greybook.cern.ch/programmes/experiments/NA48.html/
NA49	NA49 experiment (CERN)
	http://na49info.web.cern.ch/na49info/
NA57	NA57 experiment (CERN)
	http://wa97.web.cern.ch/WA97/
NA60	NA60 experiment (CERN)

**Table:**

NA61/SHINE	SPS Heavy Ion and Neutrino Experiment (CERN)
	http://home.web.cern.ch/about/experiments/na61shine
NA62	NA62 experiment (CERN)
	http://na62.web.cern.ch/na62/Home/Aim.html/
Nab at ORNL	http://www.phy.ornl.gov/groups/neutrons/beta.html
nEDM-SNS	neutron EDM experiment at the Spallation Neutron Source
	http://www.phy.ornl.gov/nedm/
New Muon $g-2$	http://muon-g-2.fnal.gov/
NuSea	http://p25ext.lanl.gov/e866/e866.html
NuTeV	http://www-e815.fnal.gov/
OBELIX	http://www.fisica.uniud.it/~santi/OBELIX/OBELIX.html
OPAL	Omni-Purpose Apparatus at LEP
	http://www.cern.ch/opal
PANDA	anti-Proton ANnihilation at DArmstadt
	http://www-panda.gsi.de/
PAX	Polarized Antiproton eXperiment
	http://collaborations.fz-juelich.de/ikp/pax/index.shtml
PEN	http://pen.phys.virginia.edu/
PERC	Proton and Electron Radiation Channel
PERKEOIII	http://www.physi.uni-heidelberg.de/Forschung/ANP/Perkeo/perkeo3.php
PHENIX	Pioneering High Energy Nuclear Interaction eXperiment
	http://www.bnl.gov/rhic/PHENIX.asp
PHOBOS	http://www.phobos.bnl.gov/
PIBETA	PI BETA experiment
	http://pibeta.phys.virginia.edu
PLANCK	http://www.rssd.esa.int/index.php?project=planck
PNDME	Precision Calculation of Neutron-Decay Matrix Elements
	http://www.phys.washington.edu/users/hwlin/pndme/index.xhtml
PREX	208Pb Radius Experiment
	http://hallaweb.jlab.org/parity/prex/
PRIMEX	Primakoff experiment
	http://www.jlab.org/primex/
QWEAK	http://www.jlab.org/qweak/
REX-ISOLDE	Radioactive Beam EXperiment at ISOLDE
	http://isolde.web.cern.ch/rex-isolde
SIDDHARTA	SIlicon Drift Detectors for Hadronic Atom Research by Timing Application
	http://www.lnf.infn.it/esperimenti/siddharta/
SND	Spherical Neutral Detector
	wwwsnd.inp.nsk.su
SNO	Sudbury Neutrino Observatory
	http://www.sno.phy.queensu.ca/
STAR	Solenoid Tracker At RHIC
	http://www.star.bnl.gov/

**Table:**

ThO	ACME Thorium Oxide Electron EDM Experiment
	http://www.doylegroup.harvard.edu/wiki/index.php/ThO
TREK	http://trek.kek.jp
UCNA	Ultra Cold Neutron Apparatus
	http://www.krl.caltech.edu/research/ucn
UCNB	UltraCold Neutron source
	http://www.ne.ncsu.edu/nrp/ucns.html
UCNb	UltraCold Neutron source
	http://www.ne.ncsu.edu/nrp/ucns.html
VES	VErtex Spectrometer (IHEP)
	http://pcbench.ihep.su/ves/index2.shtml
WA102	WA102 experiment (CERN)
	http://www.ep.ph.bham.ac.uk/exp/WA102/
UCNb	UltraCold Neutron source
	http://www.ne.ncsu.edu/nrp/ucns.html
VES	VErtex Spectrometer (IHEP)
	http://pcbench.ihep.su/ves/index2.shtml
WA102	WA102 experiment (CERN)
	http://www.ep.ph.bham.ac.uk/exp/WA102/
WASA	Wide Angle Shower Apparatus
	http://collaborations.fz-juelich.de/ikp/wasa/index.shtml
WMAP	Wilkinson Microwave Anisotropy Probe
	http://map.gsfc.nasa.gov/
ZEUS	ZEUS experiment (HERA)
	www.zeus.desy.de

**Table:**

	**Laboratories**
BNL	Brookhaven National Laboratory
	http://www.bnl.gov
CERN	European Organization for Nuclear Research
	http://home.web.cern.ch/
CSSM	Centre for the Subatomic Structure of Matter
	http://www.physics.adelaide.edu.au/cssm/
DESY	Deutsches Elektronen-SYnchrotron
	http://www.desy.de/
FAIR	Facility for Antiproton and Ion Research
	http://www.fair-center.eu/
Fermilab	Fermi National Accelerator Laboratory
	http://www.fnal.gov/
FNAL	Fermi National Accelerator Laboratory
	http://www.fnal.gov/
FRM-II	Heinz Maier-Leibnitz research neutron source
	http://www.frm2.tum.de/
GSI	GSI Helmholtz Centre for Heavy Ion Research
	http://www.gsi.de/
ILL	Institut Laue-Langevin
	http://www.ill.eu/
JINR	Joint Institute for Nuclear Research
	http://www.jinr.ru/
JLab	Jefferson Lab
	http://www.jlab.org/

**Table:**

JPARC	Japan Proton Accelerator Research Complex
	http://j-parc.jp/index-e.html
KEK	High Energy Accelerator Research Organization
	http://legacy.kek.jp/
LANL	Los Alamos National Laboratory
	http://www.lanl.gov/
LBNL	Lawrence Berkeley National Laboratory
	http://www.lbl.gov/
MAMI	MAinz MIcrotron
	http://wwwkph.uni-mainz.de/B1/
NIST	National Institute of Standards and Technology
	http://www.nist.gov
ORNL	Oak Ridge National Laboratory
	http://www.ornl.gov/
RBRC	RIKEN BNL Research Center
	http://www.bnl.gov/riken
SLAC	SLAC National Accelerator Laboratory (originally Stanford Linear Accelerator Center)
	https://www6.slac.stanford.edu/

**Table:**

	**Lattice-QCD Collaborations**
ALPHA	ALPHA collaboration
	http://www.zeuthen.desy.de/alpha/
APE	Array Processor Experiment
	http://apegate.roma1.infn.it/mediawiki/index.php/Main_Page
BGR	Bern-Graz-Regensburg
BMW	Budapest-Marseille-Wuppertal
	http://www.bmw.uni-wuppertal.de/Home.html
CLS	Coordinated Lattice Simulations
	https://twiki.cern.ch/twiki/bin/view/CLS/WebHome
CSSM Lattice	Centre for the Subatomic Structure of Matter Lattice
	http://www.physics.adelaide.edu.au/cssm/lattice/
CP-PACS	Computational Physics by Parallel Array Computer Systems
	http://www.rccp.tsukuba.ac.jp/cppacs/project-e.html
DiRAC	Distributed Research utilising Advanced Computing
	http://ukqcd.swan.ac.uk/dirac/
ETM	European Twisted Mass
	http://www-zeuthen.desy.de/~kjansen/etmc/
Fermilab Lattice	http://inspirehep.net/search?ln=en&ln=en&p=find+cn+fermilab+lattice
FLAG	Flavour Lattice Averaging Group
	http://itpwiki.unibe.ch/flag/index.php, http://www.latticeaverages.org

**Table:**

Hadron Spectrum	http://usqcd.jlab.org/projects/Spectrum/
HotQCD	http://quark.phy.bnl.gov/~hotqcd/
HPQCD	High-precision QCD
	http://www.physics.gla.ac.uk/HPQCD/
JLQCD	Japanese Lattice QCD
	http://jlqcd.kek.jp
LHP	Lattice Hadron Physics
MILC	MIMD Lattice Computations
	http://www.physics.utah.edu/~detar/milc/
PACS-CS	Parallel Array Computer System for Computational Sciences
	http://www2.ccs.tsukuba.ac.jp/PACS-CS/
PNDME	Precision Calculation of Neutron-Decay Matrix Elements
	http://www.phys.washington.edu/users/hwlin/pndme/index.xhtml
QCD-Taro	
QCDSF	QCD Structure Function
	http://inspirehep.net/search?p=find+cn+qcdsf
RBC	RBRC-BNL-Columbia
	http://rbc.phys.columbia.edu
SPQcdR	Southampton-Paris-Rome QCD
SWME	Seoul-Washington Matrix Element
	http://lgt.snu.ac.kr/
UKQCD	United Kingdom QCD
	http://www.ukqcd.ac.uk
USQCD	United States QCD
	http://www.usqcd.org
WHOT-QCD	

**Table:**

	**Other**
CKMfitter	Global analysis of CKM matrix
	http://ckmfitter.in2p3.fr/
CODATA	Committee on Data for Science and Technology
	http://www.codata.org/
CTEQ	The Coordinated Theoretical-Experimental Project on QCD
	http://users.phys.psu.edu/~cteq/
HFAG	Heavy Flavor Averaging Group
	http://www.slac.stanford.edu/xorg/hfag/
NNPDF	Neural Network Parton Distribution Functions
	https://nnpdf.hepforge.org
PDG	Particle Data Group
	http://pdg.lbl.gov
PYTHIA	after an ancient Greek priestess
	http://home.thep.lu.se/~torbjorn/Pythia.html
QWG	Quarkonium Working Group
	http://www.qwg.to.infn.it
UTfit	Unitarity Triangle fits
	http://www.utfit.org

## Footnotes

1 Contributing authors: N. Brambilla, S. Eidelman, P. Foka, S. Gardner, A.S. Kronfeld.

2 Contributing authors: N. Brambilla, S. Eidelman, P. Foka, S. Gardner, X. Garcia i Tormo, A.S. Kronfeld, R. Vogt.

3 Contributing authors: R. Alkofer$^{\dagger }$, J. L. Goity$^{\dagger }$, B. Ketzer$^{\dagger }$, H. Sazdjian$^{\dagger }$, H. Wittig$^{\dagger }$, S. Eidelman, S. Gardner, A. S. Kronfeld, Felipe J. Llanes-Estrada, A. Pich, J.-W. Qiu, C. Salgado, N. G. Stefanis.

4 The quark masses introduce a flavor dependence into the quark-quark interaction. Furthermore, flavor independence of this interaction is in disagreement with experimental facts.

5 Other possibilities exist, such as making the Higgs an additional Goldstone boson of new physics, e.g., a dilaton.

6 Here we restrict ourselves to states used by the PDG in their averages [1], together with recent data from COMPASS not yet listed in the summaries.

7 Contributing authors: M. Butenschoen$^{\dagger }$, P. Pakhlov$^{\dagger }$, A. Vairo$^{\dagger }$, N. Brambilla, X. Garcia i Tormo, G. M. von Hippel, R. Mizuk, J.-W. Qiu, G. Ricciardi.

8 For theoretical aspects of threshold resummation in $B$ decays see, e.g., Refs. [941–946].

9 Contributing authors: S. Gardner$^{\dagger }$, H.-W. Lin$^{\dagger }$, Felipe J. Llanes-Estrada$^{\dagger }$, W.M. Snow$^{\dagger }$, X. Garcia i Tormo, A.S. Kronfeld.

10 The three-flavor PMNS matrix carries two additional phases if the neutrinos are Majorana.

11 Alternatively, a dimension-five operator, which is SM electroweak gauge invariant but L-violating, can be used [1370]. After electroweak symmetry breaking, this term yields a Majorana neutrino mass term $(Y^2 v^2/\Lambda ) \nu _L^T C \nu _L$, in which $Y$ is the hypercharge and $C$ is the charge-conjugation operator, and makes neutrinoless double-$\beta$ decay possible.

12 For example, nuclear beta decay $0^{+} \rightarrow 0^+$ transitions and other processes, such as the $\beta$ asymmetry in oriented $^{60}\text{ Co }$ decay, the longitudinal polarization ratio between the Fermi and Gamow–Teller transitions in $^{114}\text{ In }$ decay, the positron polarization in polarized $^{107}\text{ In }$ decay, and the beta-neutrino correlation parameters in nuclear transitions.

13 A recent determination of the Gamow–Teller matrix element in $^6$He [1527] decay is consistent with *ab initio* calculations—no quenching of the Gamow–Teller strength has been observed.

14 We note, too, that the neutron–proton mass difference has now been computed within a self-consistent lattice-QCD calculation [590]: $[M_n - M_p]_\mathrm{QCD} ({\overline{\mathrm{MS}}},\,2\,\mathrm{GeV}) = 2.9 \pm 0.6 \pm 0.2\,\mathrm{MeV}$.

15 Consequently charge quantization is not guaranteed in a three-generation SM because the difference in family lepton numbers is anomaly free [1547, 1550]. Nevertheless, the neutron and atomic hydrogen remain electrically neutral.

16 Indeed Ref. [45] typically declines to employ such averages.

17 Contributing authors: P. Foka$^{\dagger }$, H. Meyer$^{\dagger }$, R. Vogt$^{\dagger }$, A. Vuorinen$^{\dagger }$, P. Arnold, N. Brambilla, P. Christakoglou, P. Di Nezza, J. Erdmenger, Z. Fodor, M.A. Janik, A. Kalweit, D. Keane, E. Kiritsis, A. Mischke, G. Odyniec.

18 Exponential scaling at the quantum critical point.

19 Scale dependence of the difference between the vector and axial-vector vacuum polarization amplitudes in technicolor.

20 Contributing authors: M. Alford$^{\dagger }$, T. Cohen$^{\dagger }$, L. Fabbietti$^{\dagger }$, A. Schmitt$^{\dagger }$, K. Schwenzer.

21 AGeV $=$ GeV per nucleon.

22
$F=\frac{d \langle p_X \rangle /A}{dp_\mathrm{T}} \mid _{Y=Y_{\mathrm{CM}}}$, where $Y_{\mathrm{CM}}$ is the center of mass rapidity of the nucleus–nucleus system.

23
$p_\mathrm{T}$ is the momentum component perpendicular to the beam axis.

24 Urca is the name of the casino in Rio de Janeiro where G. Gamow and M. Schönberg discussed for the first time neutrino-emitting processes responsible for the cooling of neutron stars.

25 If we work at asymptotically large densities, we must in principle also consider the heavy $c$, $b$, and $t$ quarks. However, for compact star interiors we are interested in densities where the quark chemical potential is much lower than the masses of these quarks.

26 Oscillatory modes of a compact star are classified according to the restoring force. In the case of $r$-modes or rotational modes, this is the Coriolis force.

27 It is usually assumed that the general theory of relativity gives the correct description of the intense gravitational field of a neutron star, and mass-radius measurements are then used to constrain the equation of state. However, one can alternatively assume an equation of state and obtain constraints on the gravitational coupling [2426] or on deviations from general relativity [2427].

28 See also http://chandra.harvard.edu/photo/2011/casa/coolCANSv7_lg_ web.mov for a movie of the cooling process.

29 Contributing authors: M. Faber$^{\dagger }$, M. I. Polikarpov$^{\dagger }$, R. Alkofer, R. Höllwieser, V. I. Zakharov.

30 In its simplest form, the percolation theory introduces a probability $p$ of a link or of a plaquette to be “occupied” and studies the properties of the aggregate of the occupied links or plaquettes. More generally, quantum geometry provides alternative formulations of field theories and of string theories in terms of trajectories and surfaces, respectively, for an introduction see, e.g., [2487].

31 “D” for “Dirichlet”: points (D0), surfaces (D2), and $4D$ hypersurfaces (D4) on which strings end.

32 See, e.g, Chapter 16 of [2526].

33 Here we consider only the case of four spacetime dimensions. Note that for two spacetime dimensions there is only the scaling solution found in the continuum as well as on the lattice [2529–2531].

34 In mathematical terms, it maximizes the elements of the gluon field being in the Cartan subalgebra which then also gave the name to this gauge.

35 For recent lattice calculations of the Polyakov loop potential see [2551, 2552].

36 The above value depends on a scale given by the average instanton size.

37 Contributing authors: J. Erdmenger$^{\dagger }$, E. Pallante$^{\dagger }$, K. Papadodimas, A. Pich, R. Pittau.

38 See http://amcatnlo.cern.ch for more examples.

39 M5 branes are membranes of 5+1 spacetime dimensions in 11-dimensional M-theory.
